# Cu(ii)/SPDO complex catalyzed asymmetric Baeyer–Villiger oxidation of 2-arylcyclobutanones and its application for the total synthesis of eupomatilones 5 and 6†

**DOI:** 10.1039/d2sc02079c

**Published:** 2022-06-23

**Authors:** Chang-Sheng Zhang, Ya-Ping Shao, Fu-Min Zhang, Xue Han, Xiao-Ming Zhang, Kun Zhang, Yong-Qiang Tu

**Affiliations:** State Key Laboratory of Applied Organic Chemistry, College of Chemistry and Chemical Engineering, Lanzhou University Lanzhou 730000 P. R. China tuyq@lzu.edu.cn zhangfm@lzu.edu.cn; School of Biotechnology and Health Sciences, Wuyi University Jiangmen 529020 Guangdong P. R. China; School of Chemistry and Chemical Engineering, Shanghai Jiao Tong University Shanghai 200240 P. R. China

## Abstract

A novel classical kinetic resolution of 2-aryl-substituted or 2,3-disubstituted cyclobutanones of Baeyer–Villiger oxidation catalyzed by a Cu(ii)/SPDO complex is reported for the first time, producing normal lactones in excellent enantioselectivities (up to 96% ee) and regioselectivities (up to >20/1), along with unreacted ketones in excellent enantioselectivities (up to 99% ee). The current transformation features a wide substrate scope. Moreover, catalytic asymmetric total syntheses of natural eupomatilones 5 and 6 are achieved in nine steps from commercially available 3-methylcyclobutan-1-one.

## Abstract 2

A novel classical kinetic resolution of Baeyer–Villiger oxidation catalyzed by a Cu(ii)/SPDO complex with excellent enantioselectivity, regioselectivity and wide substrate scope is reported for the first time and explore the synthetic application.

## Graphical abstract

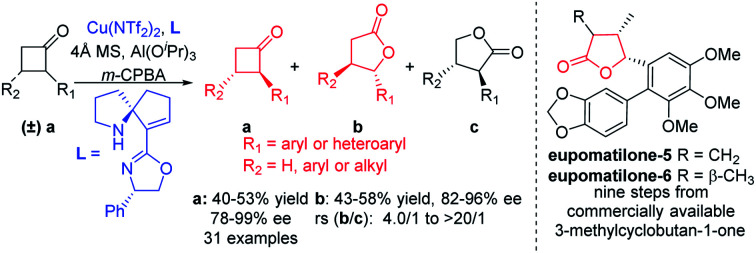

## Introduction

**Scheme 1:**
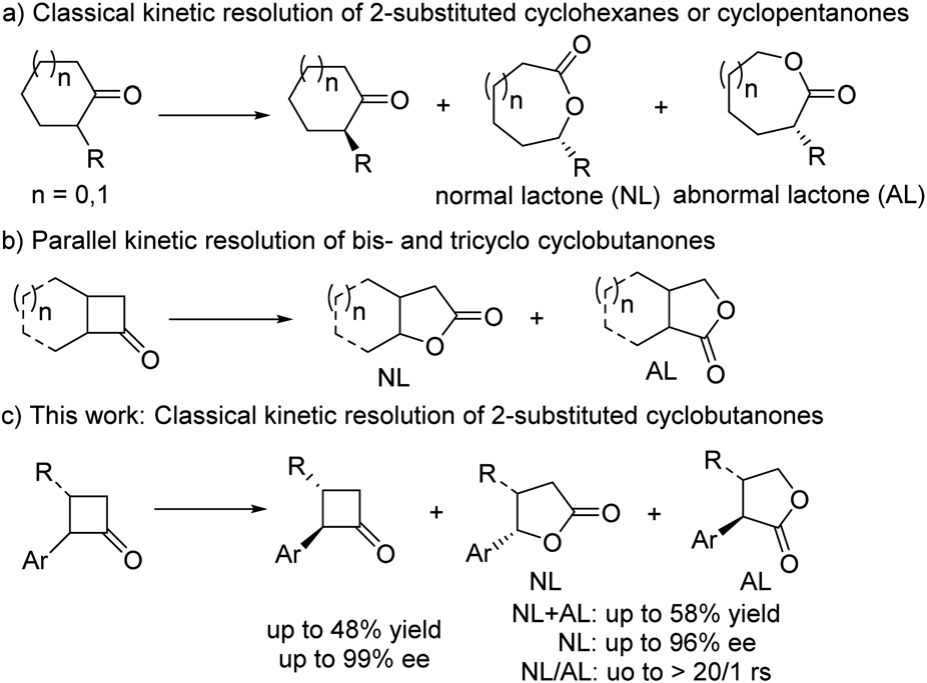
The overview of asymmetric B–V oxidations involving 2-substituted cycloketones.

Baeyer–Villiger (B–V) oxidation, which was first reported in 1898,^1^ is an important transformation in organic synthesis because it provides a concise and convenient approach toward esters from ketones or aldehydes, especially lactones from cycloketones.^2–5^ Asymmetric B–V oxidation using chiral substrates as the starting materials has been widely applied to prepare chiral lactones; however, its catalytic asymmetric version was explored by Bolm in the 1990s.^5*a*^ Since then, catalytic asymmetric B–V oxidation has received much attention from synthetic chemists, and chiral bio-,^3^ org-,^4^ and metal catalytic systems^5^ have been developed, delivering valuable synthetic building blocks such as γ-, δ-, and ε-lactones with excellent enantioselectivities and/or regioselectivities (rs). Although some achievements have been made in recent decades,^2^ catalytic asymmetric investigations still need to be fully developed; in particular, the scope of substrates is limited to two types, namely prochiral and cyclic meso-ketones,^4*a*–*j*,5*d*–*y*^ which were investigated through desymmetric B–V oxidation, and racemic cyclic ketones through the kinetic resolution of B–V oxidation.^4*i*,*k*,*n*,5*a*–*d*,*h*–*y*^ In the latter, 2-substituted or 3-substituted cyclohexanones and 2-substituted cyclopentanones were explored,^4*g*,*i*,*n*,5*v*–*y*^ resulting in ideal selectivity and excellent enantioselectivity. However, for their ring-strained cyclobutanone analogues, just limited bicyclic or tricyclic substrates could produce the desired γ-lactone with good enantiomeric excess (ee) (Scheme 1b),^4*i*,*m*,*n*,5*h*–*y*^ while 2-substituted cyclobutanones and more challenging 2,3-chain substituted cyclobutanones have remained an unsolved problem till now. To the best of our knowledge, only one example, *i.e.*, 2-phenylcyclobutanone, was reported by Feng and co-workers in 2014,^5*w*^ and a moderate ee value and poor rs ratio were observed using their own developed privileged chiral *N*,*N*′- dioxide/Sc(iii) system that exhibited excellent activities and enantioselectivities for a wide range of cyclohexanones and cyclopentanones in the desymmetric or kinetic resolution of B–V oxidations^5*v*–*y*^ and other diverse asymmetric transformations.^6^ From a synthetic perspective, there are some synthetic challenges in the classical kinetic resolution of 2-substituted cyclobutanones and their derivatives, including the following: (1) easy racemization of the unreacted 2-substituted enantiomer is inevitable due to the effect of the adjacent carbonyl group during its purification at room temperature; (2) both the regioselectivity and enantioselectivity of the reacted enantiomer can be simultaneously controlled in the subsequent transformation of its key Criegee intermediates;^7^ (3) the chiral catalyst could tolerate the corresponding oxidation conditions; and (4) the lack of appropriate functional groups in 2-substituted cyclobutanones which could interact with the chiral catalysts. However, this classical kinetic resolution through asymmetric B–V oxidation could consistently deliver two synthetically valuable products, namely chiral γ-lactone and 2-substituted cyclobutanone. Therefore, it is essential to develop a general B–V oxidation involving 2-substituted cyclobutanone and its derivatives.

Over the past few years, our group has developed structurally unique spiro-pyrrolidine oxazoline (SPDO)-derived catalysts,^8,9^ which exhibit excellent properties in some catalytic asymmetric transformations, especially aerobic oxidative coupling reactions.^9*a*,*c*^ Therefore, we wondered whether SPDO catalysts could be used as suitable ligands to catalyse the challenging classical kinetic resolution of 2-substituted cyclobutanone under B–V oxidation conditions.^5*w*^ If successful, this transformation could not only solve the above-mentioned synthetic challenges but also further expand this rarely reported Cu-catalyzed asymmetric B–V oxidation, which would simultaneously provide two synthetically useful chiral products. Herein, we wish to report our research results as a communication (Scheme 1c).

## Results and discussion

Initially, we selected 2-phenylcyclobutan-1-one^5*w*,10^ as a model substrate and 3-chloroperoxybenzoic acid (*m*-CPBA)^11^ as a stoichiometric oxidant in the presence of the Cu(OTf)_2_ complex and ligand L1 to evaluate our desired classical kinetic resolution procedure. The preliminary results indicate that unreacted 2-phenylcyclobutan-1-one 1a was isolated with moderate ee, along with normal lactone 1b and its regioisomer 1c in an almost equal ratio (entry 1, Table 1). Compared to other tested copper salts (entries 2–4, Table 1), Cu(NTf_2_)_2_ exhibited excellent enantioselectivity with unreacted 2-phenylcyclobutan-1-one 1a and better regioselectivity with lactones 1b and 1c (4.7 : 1). Subsequently, other chiral SPDO ligands L2 and L3 were used to replace L1, but no better result was obtained (entries 5 and 6, Table 1).^11^ To our delight, the improved regioselectivity of lactones b and c was obtained in different halogenated solvents (entries 7–9, Table 1). For comprehensive consideration of the enantioselectivity of the two desired products 1a and 1b and regioselectivity of 1b and 1c, different ratios of mixed solvent of tetrahydrofuran (THF) and haloalkane were further screened. Better results were observed when the mixed solvent (THF : CHBr_3_ = 1 : 1) was used (entries 10–12, Table 1).^11^ When the model reaction was performed at −40 °C, improvements in the ee value of lactone 1b to 92% and regioselectivity ratio of 1b/1c to 15/1 were obtained, although the ee value of unreacted ketone 1a was only 72% (entry 13, Table 1). Inspired by Feng's excellent work in the B–V oxidations of a variety of substrates,^5*v*–*y*^ various additives were introduced into the reaction mixture to further improve the enantioselectivity of ketone 1a, and the combination of Al(O^*i*^Pr)_3_ and 4 Å molecular sieves (MS) showed the best reaction results.^11^ In this case, the enantioselectivity of unreacted ketone 1a increased to 91% ee, and the enantioselectivity of lactone 1b and regioselectivity of 1b/1c were also less influenced (entry 15, Table 1). On further decreasing the reaction temperature to −50 °C, no better result was obtained (entry 16, Table 1). Notably, the ligand *ent*-L1 exhibited similar reaction results, providing the enantiomers *ent*-1a and *ent*-1b, respectively (entry 17, Table 1). Therefore, the reaction parameters listed in entry 15 (Table 1) were selected as the optimal reaction conditions for subsequent investigations.

**Table 1:** Optimization of the reaction conditions^a^

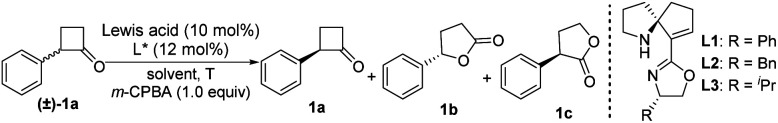								
Entry	Ligand	Lewis acid	Solvent (mL)	*T* (°C)	Time (h)	1a: yield^b^ (%)	1b + 1c: yield^b^ (%)	rs^d^
						ee^c^ (%)	1b: ee^c^ (%)	1b/1c
1	L1	Cu(OTf)_2_	THF	0	18	43/56	55/87	1.1/1
2	L1	Cu(NTf_2_)_2_	THF	0	10	45/70	52/74	4.7/1
3	L1	Cu(BF_4_)_2_·6H_2_O	THF	0	10	50/34	45/86	1.7/1
4	L1	Cu(ClO_4_)_2_·6H_2_O	THF	0	10	52/30	40/84	1.2/1
5	L2	Cu(NTf_2_)_2_	THF	0	18	48/35	50/45	3.8/1
6	L3	Cu(NTf_2_)_2_	THF	0	18	43/41	54/40	3.9/1
7	L1	Cu(NTf_2_)_2_	DCM	0	6	46/40	48/47	15.0/1
8	L1	Cu(NTf_2_)_2_	DCE	0	5	49/30	48/46	14.1/1
9	L1	Cu(NTf_2_)_2_	CHCl_3_	0	5	46/57	50/70	11.2/1
10^e^	L1	Cu(NTf_2_)_2_	THF/DCM	0	10	43/80	52/69	9.0/1
11^e^	L1	Cu(NTf_2_)_2_	THF/CHCl_3_	0	10	46/77	51/80	8.5/1
12^e^	L1	Cu(NTf_2_)_2_	THF/CHBr_3_	0	18	48/75	45/88	8.0/1
13^e^	L1	Cu(NTf_2_)_2_	THF/CHBr_3_	−40	36	54/72	45/92	15.0/1
14^e^^,^^f^	L1	Cu(NTf_2_)_2_	THF/CHBr_3_	−40	36	51/66	46/94	14.8/1
15^e^^,^^f^^,^^g^	L1	Cu(NTf_2_)_2_	THF/CHBr_3_	−40	36	43/91	52/92	12.5/1
16^e^^,^^f^^,^^g^	L1	Cu(NTf_2_)_2_	THF/CHBr_3_	−50	60	48/85	48/90	13.0/1
17^e^^,^^f^^,^^g^	*ent*-L1	Cu(NTf_2_)_2_	THF/CHBr_3_	−40	36	44/−91	52/−92	12.5/1

a Reaction conditions: unless otherwise noted, the reactions were performed with 1a (0.2 mmol), Cu(NTf_2_)_2_ (10 mol%), ligand (12 mol%) and *m*-CPBA (1.0 equiv.) in THF (2.0 mL) at 0 °C.

b Isolated yield.

c Determined by UPC^2^ analysis.

d The regioselectivity (rs) of b/c was determined by ^1^H NMR of crude products.

e 2 mL THF and 2 mL haloalkane were used.

f 4 Å MS (60 mg) was added.

g Al(O^*i*^Pr)_3_ (50 mol%) was used.

With the optimal reaction conditions in hand, the substrate scope of 2-aryl cyclobutanone was investigated (Table 2). In most cases, high ee of γ-lactone b (82–94%) and unreacted ketone a (78–99%), decent rs of γ-lactones b/c, and good selectivity factor (*s*-factor) were obtained.^5*v*,12^ For the substrates bearing mono-substituents on the aryl ring, some experimental phenomena were observed from the reaction results, including the following: (1) The substituents on the aromatic ring at the *para*-position affected the reaction results, that is, the steric hindrance of substituents on the aromatic ring was greater, reaction time was longer, and ratio of γ-lactones b/c was lower (entries 1–5, Table 2). (2) The substituents at the *ortho*-position dramatically affected the desired B–V oxidation, and poor results were obtained even at room temperature (entry 3, Table 2). (3) An electron-donating group (EDG) on the arenes accelerated the reaction and improved the ratio of b/c (entries 7–10, Table 2), while electron-withdrawing groups (EWGs) exhibited the opposite effect on the reaction results (entries 11–15, Table 2), especially regarding the rs of γ-lactones (entries 7–10 & 11–14, Table 2). For bis-substituted aryl substrates, ketones 16a–19a or lactones 16b–19b were concisely isolated with excellent selectivity (entries 16–19, Table 2), and substituents at the 3,4-positions had less effect on the results. More importantly, the 3,4,5-tri-OMe-substituted substrate reacted well in terms of the resulting enantioselectivity and rs (entries 17 & 20, Table 2). Although 1-naphthyl cyclobutanone showed moderate rs with an excellent ee (entry 22, Table 2), the greater steric hindrance of the 2-naphthyl and 1-pyrenyl analogues improved the rs, enantioselectivities, and reactivities, and just 0.6 equiv. of *m*-CPBA was needed (21a, 23a). Notably, the absolute configuration of lactones 6b, 16b, and 21b was further determined by X-ray analysis. Additionally, substrates with heteroaromatic benzothiophenyl (24a), thienyl (25a, 26a), and benzofuranyl (27a) substituents were well tolerated, resulting in ketones 24a–27a and lactones 24b–27b with slightly decreased enantioselectivities; unsatisfactorily, substrate 24a showed moderate regioselectivity (4.0/1). Overall, whether for aryl rings with a mono-substituent or multi-substituents or for heteroaryl rings, the desired B–V oxidation reactions proceeded well in most cases, providing two valuable synthetic building blocks (2-aryl-substituted cycloketones and γ-lactones) with excellent enantioselectivities.

**Table 2:** Substrate scope of 2-substituted cyclobutanones^a^

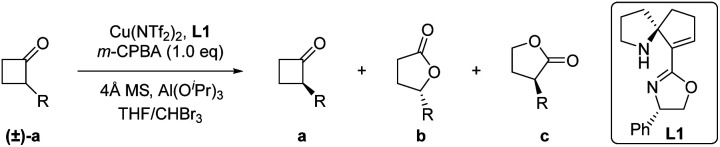						
Entry	Substituents	Time	a: yield/ee	b + c: yield/b: ee	b/c rs	*s*-factor
1	1a: R = Ph	36 h	43%/91%	52%/93%	12.5/1	88
2	2a: R = 4-Me-Ph	48 h	43%/98%	57%/90%	10.0/1	87
3	3a: R = 2-Me-Ph	72 h	52%/7%	44%/61%	1.5/1^b^^,^^c^	4.4
4	4a: R = 4-^*i*^Pr-Ph	50 h	44%/90%	52%/91%	9.2/1^c^	65
5	5a: R = 4-^*t*^Bu-Ph	50 h	53%/78%	43%/92%	10.0/1^c^	57
6	6a: R = 4-Ph-Ph	42 h	45%/90%	54%/91%	12.5/1^g^	65
7	7a: R = 4-MeO-Ph	28 h	44%/95%	54%/91%	16.6/1	79
8	8a: R = 3-MeO-Ph	38 h	47%/95%	51%/91%	12.4/1	79
9	9a: R = 4-PhO-Ph	36 h	45%/99%	48%/93%	12.5/1	145
10	10a: R = 4-BnO-Ph	39 h	43%/99%	52%/88%	13.0/1	82
11	11a: R = 4-F-Ph	39 h	49%/82%	50%/92%	6.3/1^c^	61
12	12a: R = 3-F-Ph	39 h	48%/88%	50%/91%	5.0/1^c^	62
13	13a: R = 4-Cl-Ph	36 h	43%/87%	56%/92%	5.9/1^c^	68
14	14a: R = 4-Br-Ph	38 h	45%/85%	50%/92%	6.3/1^c^	65
15	15a:R = 3-MeCO_2_Ph	38 h	45%/92%	53%/91%	6.1/1	70
16	16a: R = 2,3-dihydrobenzofuranyl	38 h	40%/95%	56%/86%	16.7/1^g^	49
17	17a: R = 3,4-diMeO-Ph	39 h	40%/99%	58%/87%	14.3/1	75
18	18a: R = 3-F-4-MeO-Ph	38 h	47%/94%	49%/89%	12.5/1	61
19	19a: R = 3-Cl-4-MeO-Ph	38 h	46%/97%	52%/90%	11.1/1	80
20	20a: R = 3,4,5-triMeO-Ph	48 h	48%/91%	48%/85%	>20/1	39
21	21a: R = 2-naphthyl	42 h	48%/91%	51%/92%	>20/1^d^^,^^g^	76
22	22a: R = 1-naphthyl	38 h	41%/98%	56%/94%	6.0/1^e^	149
23	23a: R = 1-pyrenyl	42 h	44%/96%	55%/89%	>20/1^d^	67
24	24a: R = 3-benzothiophenyl	48 h	42%/84%	57%/90%	4.0/1^c^^,^^f^	50
25	25a: R = 3-thienyl	36 h	45%/90%	51%/90%	17.1/1^c^	58
26	26a: R = 2-thienyl	48 h	40%/98%	54%/86%	12.0/1	60
27	27a: R = 2-benzofuranyl	36 h	42%/96%	53%/82%	10.0/1	39

a Reaction conditions: unless otherwise noted, the reactions were performed with a (0.2 mmol), Cu(NTf_2_)_2_ (10 mol%), L1 (12 mol%), 60 mg 4 Å MS and *m*-CPBA (1.0 equiv.) in THF/CHBr_3_ (2.0/2.0 mL) for the indicated time at −40 °C. Isolated yield. ee was determined by UPC^2^ analysis. The rs of b/c was determined by crude ^1^H NMR.

b The reaction was performed at 20 °C, and the ee value of 3c was 44.8%.

c THF/Et_2_O/CHBr_3_ (1.0/1.0/2.0 mL) was used.

d 0.6 equiv. of *m*-CPBA was used.

e The ee value of 22c was 95%.

f The ee value of 24c was 85%.

g The absolute configuration of the products was confirmed by X-ray analysis.

After expansion of the substrate scope of 2-substituted cyclobutanones, we turned our attention to more synthetically challenging 2,3-disubstituted cyclobutanones with an additional functional group at the ketone ring (Table 3). The reaction of 2,3-disubstituted cyclobutanones also proceeded well (28a–32a). With respect to 3-aryl-substituted substrates, excellent enantioselectivities (94–98% ee) and good rs (8.3/1–12.5/1 rs) were obtained with either EDG (OMe) or EWG (CF_3_) substituents at the *para*-position of aryl substrates. Notably, this reaction was performed on the gram scale, and products 28a and 28b were isolated without a decrease in the yield and selectivity (entry 2, Table 3). For the 3-methyl-2-aryl-substituted substrate, the reaction also proceeded well under the optimal reaction conditions; both an excellent ee of ketone 32a (91% ee) and excellent rs of 32b and 32c (>20/1) were observed, although with a moderate ee of γ-lactone 32b. Further improvement in the ee value of lactone *ent*-32b will be discussed in the asymmetric total synthesis of natural products.

**Table 3:** Substrate scope of 2,3-bis-substituted cyclobutanones^a^

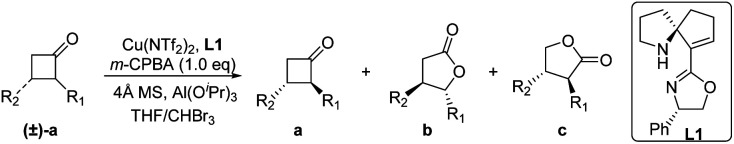						
Entry	Substituents	Time	a: yield/ee	b + c: yield/b: ee	b/c rs	*s*-factor
1	28a: R_1_ = Ph, R_2_ = Ph	36 h	46%/96%	52%/94%	12.5/1^e^	127
2	28a: R_1_ = Ph, R_2_ = Ph	38 h	48%/95%	52%/94%	12.5/1^b^	121
3	29a: R_1_ = Ph, R_2_ = 4-F-Ph	37 h	49%/96%	50%/94%	8.3/1	127
4	30a: R_1_ = Ph, R_2_ = 4-CF_3_-Ph	37 h	48%/95%	50%/93%	9.1/1	103
5	31a: R_1_ = Ph, R_2_ = 4-MeO-Ph	37 h	48%/98%	51%/96%	12.5/1	226
6	32a: R_1_ = 3,4,5-triMeO-Ph, R_2_ = Me	26 h	40%/91%	50%/86%	>20/1^c^	42
7	32a: R_1_ = 3,4,5-triMeO-Ph, R_2_ = Me	32 h	48%/91%	48%/92%	19.0/1^c^^,^^d^	121

a Reaction conditions: unless otherwise noted, the reactions were performed with a (0.2 mmol), Cu(NTf_2_)_2_ (10 mol%), L1 (12 mol%), 60 mg 4 Å MS. and *m*-CPBA (1.0 equiv.) in THF/CHBr_3_ (2.0/2.0 mL) for the time indicated at −40 °C. Isolated yield. ee was determined by UPC^2^ analysis. The rs of b/c was determined by crude ^1^H NMR.

b 1.0 g 28a was used.

c *ent*-L1 (12% mol) was applied.

d Cu(OTf)_2_ (10 mol%) was applied to replace Cu(NTf_2_)_2_.

e The absolute configuration of 28b was confirmed by X-ray analysis.

**Scheme 2:**
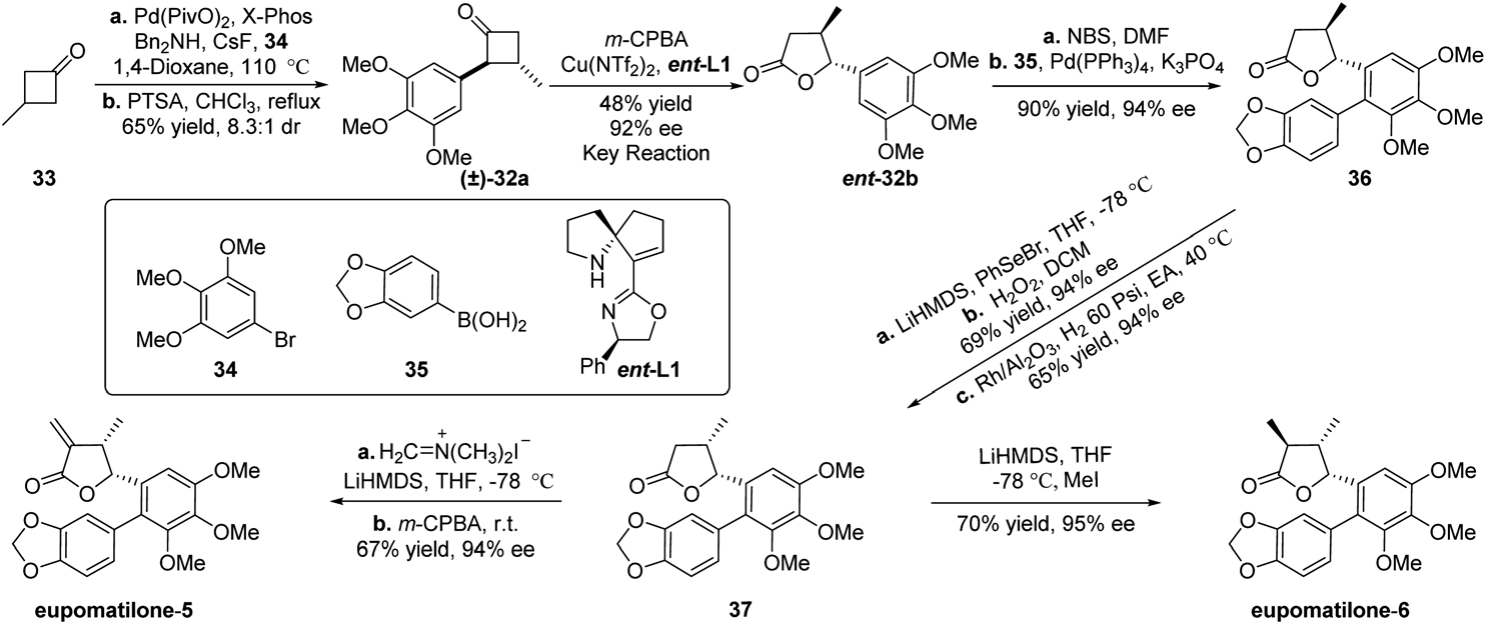
Asymmetric total syntheses of eupomatilones 5 and 6.

After completing the study of generality of this classical kinetic resolution of 2-aryl-substituted and 2,3-disubstituted cyclobutanones, we focused our efforts on exploring the synthetic application of this unexplored methodology and targeted eupomatilones 5 and 6 isolated from the Australian shrub *Eupomatia* (Scheme 2).^13,14^ Starting from commercially available 3-methylcyclobutan-1-one (33), Pd-catalyzed α-arylation of cyclobutanone with 1-bromo-3,4,5-trimethoxybenzene (34) was carried out to prepare racemic precursor 32a for the B–V reaction. However, poor yield and/or diastereoisomeric ratio (dr) was observed under the tested reaction conditions.^11^ To our delight, after extensive investigation, the desired racemate 32a was obtained in 5.6 : 1 dr using a more sterically hindered ligand (X-Phos), and the dr of (±)-32a was further increased to 8.3 : 1 by treating with *p*-toluenesulfonic acid in refluxing chloroform. To improve the dissatisfactory results of the desired lactone 32b achieved under the optimal reaction conditions (entry 6, Table 3), slightly adjusted reaction conditions (Cu(OTf)_2_ and *ent*-L1 were applied)^11^ were used to furnish the expected lactone *ent*-32b in 48% yield with an excellent ee of 92% (entry 7, Table 3). With chiral lactone *ent*-32b in hand, mono-bromination of the aryl ring with *N*-bromosuccinimide (NBS) and the subsequent Pd-catalyzed Suzuki–Miyaura reaction with 1,3-benzodioxole-5-boronic acid (35) gave biaryl compound 36^13*b*,15^in 90% yield and 94% ee (two steps). To reverse the configuration of the methyl group at the β-position of γ-lactone, compound 36 was treated with phenylselenyl bromide (PhSeBr) and lithium bis(trimethylsilyl)amide (LiHMDS) at −78 °C and then oxidized with 30% H_2_O_2_ to yield an unsaturated lactone. The subsequent catalytic hydrogenation of the resulting lactone with Rh/Al_2_O_3_ in ethyl acetate (EA) at 40 °C afforded γ-butyrolactone 37 with high diastereoselectivity.^16^ Finally, the total synthesis of eupomatilones 5 and 6 was concisely completed in just a one-step transformation. Treatment of 37 with Eschenmoser's salt in THF at −78 °C and subsequent elimination produced eupomatilone-5 in 67% yield and 94% ee, while eupomatilone-6 was obtained through stereoselective methylation using LiHMDS and MeI at −78 °C (70% yield, 95% ee).^13*e*^ The spectral data of two synthetic natural products were consistent with the reported literature.^13*b*,*d*–*g*^

## Conclusions

In summary, we developed an efficient Cu–SPDO catalytic system that exhibits excellent activity in the classical B–V oxidation of 2-aryl-substituted or 2,3-disubstituted cyclobutanones for the first time. The current transformation features a wide substrate scope and excellent enantioselectivity and regioselectivity, providing an alternative and concise approach for the simultaneous preparation of chiral γ-lactones and chiral aryl-substituted cyclobutanones. Additionally, asymmetric total syntheses of natural eupomatilones 5 and 6 were completed using this newly developed methodology as a key step. Other asymmetric reactions catalyzed using metal/SPDO complexes, and their synthetic applications are underway in our group.

## Data availability

The datasets supporting this article have been uploaded as part of the ESI.†

## Author contributions

C.-S. Zhang, F.-M. Zhang and Y.-Q. Tu designed this project. C.-S. Zhang performed the main experiments and prepared the ESI; Y.-P. Shao performed part of the experiments. X. Han synthesised some substrates. X.-M. Zhang, F.-M. Zhang and Y.-Q. Tu supervised and directed the project. C.-S. Zhang, F.-M. Zhang and Y.-Q. Tu wrote the manuscript. All authors discussed the finalized manuscript.

## Conflicts of interest

There are no conflicts to declare.

## Supplementary Material

SC-013-D2SC02079C-s001

SC-013-D2SC02079C-s002

SC-013-D2SC02079C-s003
