# Supplementary material for: Cu(ii)/SPDO complex catalyzed asymmetric Baeyer–Villiger oxidation of 2-arylcyclobutanones and its application for the total synthesis of eupomatilones 5 and 6
Source: Chem Sci. 2022 Jun 23;13(28):8429–35. doi: 10.1039/d2sc02079c (PMC9297696; doi:10.1039/d2sc02079c)

Electronic Supplementary Material (ESI) for Chemical Science.
This journal is © The Royal Society of Chemistry 2022

[Electronic supplementary information]

Cu(II)/SPDO complex-catalyzed asymmetric Baeyer–Villiger oxidation of 2-arylcyclobutanones and its application for the total synthesis of eupomatilones 5 and 6

Chang-Sheng Zhang,[a] Ya-Ping Shao,[a] Fu-Min Zhang,*[a] Xue Han,[a] Xiao-Ming Zhang,[a] Kun Zhang,[b] Yong-Qiang Tu,*[ac]

[a]State Key Laboratory of Applied Organic Chemistry and College of Chemistry and Chemical Engineering, Lanzhou University, Lanzhou 730000, P. R. China.

[b]School of Biotechnology and Health Sciences, Wuyi University, Jiangmen, Guangdong, 529020, P. R. China

[c]School of Chemistry and Chemical Engineering, Shanghai Jiao Tong University, Shanghai, 200240, P. R. China.

E-mail: tuyq@lzu.edu.cn (Y.-Q. Tu); zhangfm@lzu.edu.cn (F.-M. Zhang)

Table of Contents

1. **General information** ... 1
2. **Tables for optimization of the reaction conditions** ... 2
3. **Preparation of Ligands.** ... 3
4. **Synthesis of Substituted (phenylmethylene)cyclopropanes** ... 6
5. **General procedure for the synthesis of cyclobutanones a** ... 11
6. **[2+2] Synthesis of Heteroaromatic substituted Cyclobutanone** ... 16
7. **Synthesis of 2,3-disubstituted Cyclobutanone** ... 18
8. **Characterization of the products and unreacted Cyclobutanone** ... 19
9. **Optimization of direct α-arylation of cyclobutanone (Table S4)** ... 72
10. **Synthesis of eupomatilone-5 and eupomatilone-6** ... 73
11. **A possible mechanism for Cu/SPDO catalyzed Baeyer–Villiger oxidation** ... 83
12. **References** ... 84
13. **X-ray Crystallographic Data** ... 85
14. **^{1}H NMR, ^{13}C NMR and ^{19}F NMR Spectra of the Compounds** ... 89

1. General information

Unless stated otherwise, all glassware was dried under 110 ℃ in drying oven, and cooled down to room temperature in an atmosphere of argon. In addition to commercially available extra dry solvents, all solvents were purified by standard operating method. Tetrahydrofuran (THF), toluene, and diethyl ether (Et_2O) were distilled from sodium; dichloromethane (DCM), 1,2-dichloroethane (DCE) and chloroform ($CHCl_3$) were dried by calcium hydride. **Bromoform** ($CHBr_3$) was washed by water, dried by K_2CO_3 and then vacuum distillation under 60 ℃/45 mbar. 4Å molecular sieve (M.S.) do not need activation and drying. **3-chloroperoxybenzoic acid** (***m*-CPBA**) can be used for the reaction after washing with a phosphate buffer PH = 7.5 solution, and drying the residue under reduced pressure.

All reactions under standard conditions were monitored by thin layer chromatography (TLC) on gel F_{254} plates, visualized by ultraviolet light (254 nm). The products were purified by column chromatography on silica gel (200~300 mesh).

^{1}H NMR, **^{13}C NMR** and **^{19}F NMR** spectra were obtained from AVANCE III HD 400 or AVANCE NEO 600. Chemical shifts were recorded relative to tetramethylsilane: δ 0.00 or $CDCl_3$: 7.26 ppm for **^{1}H NMR**, 77.0 ppm for **^{13}C NMR**; The following abbreviations were used to indicate the multiplicity in NMR spectra: s = singlet; d = doublet; t = triplet; q = quartet; dd = doublet of doublets; td = triplet of doublets; m = multiplet.

High-resolution mass spectral analysis (**HRMS**) data were measured on the Bruker ApexII with ESI resource.

Infrared (**IR**) spectra were recorded on Nicolet is5 spectrometer.

The enantiomeric excesses (**ee**) value of the products was determined by Ultra Performance Convergence Chromatography (UPC^2) equipped with Waters 2998 Photodiode Array Detector or High Performance Liquid Chromatography (**HPLC**) analysis on the 2998 PDA Detector instruments.

Electron ionization mass (EI-MS) spectra with direct inlet at 70 eV were measured on a Shimadzu GCMSQP2010SE spectrometer, and the corresponding signals were given in m/z with relative intensity (%) in brackets.

The X-ray single-crystal determination was performed on a Bruker APEX II X-ray single crystal diffractometer.

Melting points (m. p.) were measured on a melting point apparatus and were uncorrected.

2. Tables for optimization of the reaction conditions

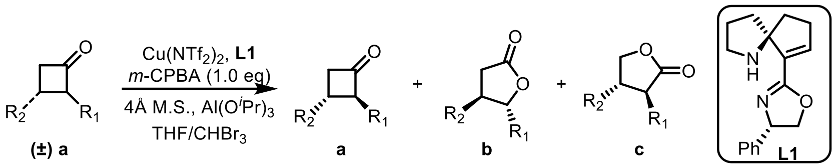

General procedure: the mixture of anhydrous $Cu(NTf_2)_2$ (12.5 mg, 0.02 mmol), **L1** (6.4 mg, 0.024 mmol) was stirred in THF (1 mL) for 1 h at room temperature (r. t.). The 4Å molecular sieves (M.S., 60 mg), $Al(O^iPr)_3$ (20.4 mg, 50% mol) and **1a** was added to an oven-dried reaction tube in batches, then the chiral catalyst solution prepared beforehand, THF (1 mL) and $CHBr_3$ (2 mL) were added, the reaction mixture was cooled to -40 °C for 20 min. Then, the *m*-CPBA (0.2 mmol) was added to the reaction mixture at -40 °C. The Na_2SO_3 (126 mg, 1.0 mmol) was added after the reaction mixture had been stirred for the time indicated at -40 °C, and stirred for additional 2 h at -40 °C. The DCM (10 mL) was added, the solution was extracted with DCM. The combined organic layers were washed with brine, dried ($NaSO_4$), concentrated and the crude product was purified by flash column chromatography on silica gel at **-15 °C** to afford the desired lactones **b/c** and recover the unreacted ketones **a**.

Table S1: Investigation of the Ligands when $Cu(NTf_2)_2$ as Lewis acid[a].

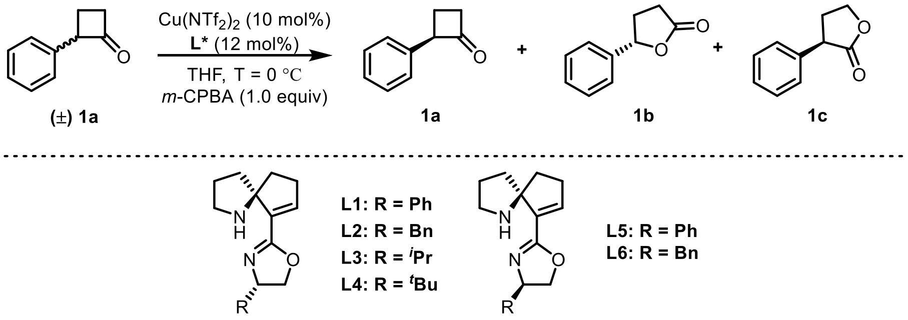

entry	ligand	Time/h	**a**: yield/%[b] ee/%[c]	**b**+**c**: yield/%[b] **c**: ee/%[c]	rs[d] **1b**/**1c**
1	**L1**	10	29/92	70/63	4.7/1
2	**L2**	18	48/35	50/45	3.8/1
3	**L3**	18	39/24	58/33	3.5/1
4	**L4**	36	57/2	40/3	1.6/1
5	**L5**	18	47/-7	46/-31	1.8/1
6	**L6**	18	42/28	52/32	3.0/1

[a] Reaction conditions: **1a** (0.2 mmol), $Cu(NTf_2)_2$ (10 mol%), Ligand (12 mol%) and *m*-CPBA (1.0 equiv) in THF (2.0 mL) at 0 °C for indicated time. [b] Isolated yield. [c] Determined by UPC^2 analysis. [d] The regioselectivity of **b/c** was determined by crude ^1H NMR.

Table S2: Investigation of the component solvent[a].

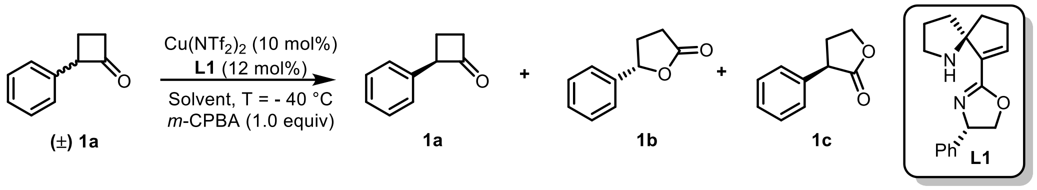

entry	Solvent	Time/h	**a:** yield/%[b] ee/%[c]	**b+c:** yield/%[b] **b**: ee/%[c]	rs[d] **1b**/**1c**
1	THF	48	53/43	44/79	6.1/1
2	THF/DCM	36	56/70	41/89	19/1
3	THF/DCE	32	55/66	43/80	18/1
4	THF/$CHCl_3$	36	56/70	41/85	19/1
5	THF/$CHBr_3$	36	54/72	45/92	15/1

[a] Reaction conditions: **1a** (0.2 mmol), $Cu(NTf_2)_2$ (10 mol%), Ligand (12 mol%) and *m*-CPBA (1.0 equiv) in component solvent (4.0 mL) at -40 °C for indicated time. [b] Isolated yield. [c] Determined by UPC^2 analysis. [d] The regioselectivity (rs) of **b/c** was determined by crude ^1H NMR.

Table S3: Investigation of the Additives[a].

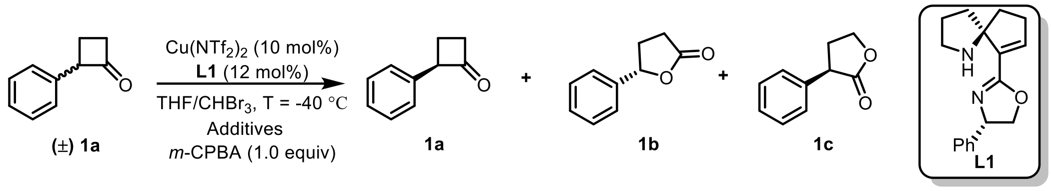

entry	additives	Time /h	**a:** yield/%[b] ee/%[c]	**b+c:** yield/%[b] **b:** ee/%[c]	rs **1b**/**1c**
1	3Å M.S.[e]	36	51/68	46/89	16.9/1
2	4Å M.S.[e]	36	51/66	46/94	14.8/1
3	5Å M.S.[e]	30	43/65	55/87	19.1/1
4	4Å M.S.[e], 2,4,6-tris(1-methylethyl)benzoic acid	36	48/68	50/94	16.1/1
5	4Å M.S.[e], $Al(OEt)_3$	36	50/78	46/90	11.0/1
6	4Å M.S.[e], $Al(O^iPr)_3$	36	43/91	52/92	12.5/1
7	4Å M.S.[e], $Al(O^tBu)_3$	72	55/55	40/90	15.0/1
8	4Å M.S.[e], $NaBAr^F$[f]	10	40/25	54/41	>20/1

[a] Reaction conditions: **1a** (0.2 mmol), $Cu(NTf_2)_2$ (10 mol%), Ligand (12 mol%) and *m*-CPBA (1.0 equiv) in THF/$CHBr_3$ (2 mL/2 mL) at -40 °C for indicated time. [b] Isolated yield. [c] Determined by UPC^2 analysis. [d] The regioselectivity of **c**/**d** was determined by crude ^1H NMR. [e] M.S.: 60 mg. [f] $NaBAr^F$ = Sodium tetrakis[3,5-bis(trifluoromethyl)phenyl]borate

3. Preparation of Ligands.

General procedure for Ligands **L1**-**L6** according to the literature.[1]

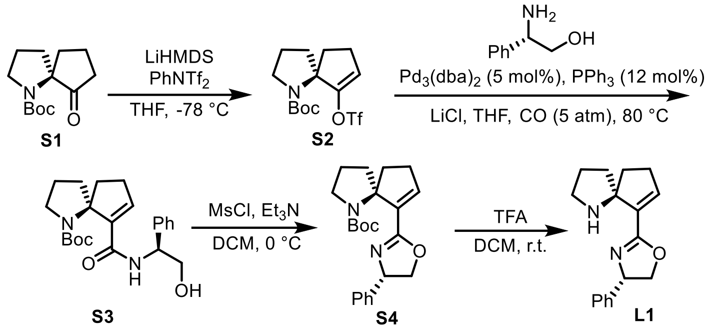

Scheme S1. General procedure for Ligands **L1**

Preparation of **L1**:

S2: **S1** (1.0 g, 4.18 mmol) was dissolved in dry THF (42 mL) under argon atmosphere at room temperature and then cooled down to -78 °C. Lithium bis(trimethylsilyl)amide (1.0 M in THF, 4.6 mL, 1.1 equiv) was added dropwise via a syringe. The resulted solution was stirred at -78 °C for 1 h. And then *N,N*-Bis(trifluoromethylsulfonyl)aniline (1.64 g, 4.6 mmol, 1.1 equiv) was added portionwise and the reaction mixture was stirred at room temperature for additional 4 h. Then the reaction mixture was quenched by the addition of saturated NH_4Cl aqueous solution (10 mL) after full conversion, and the resultant mixture was extracted with EtOAc. The combined organic layers were washed again with brine, dried ($NaSO_4$) and concentrated to give crude product **S2** which can be used in the next step without further purification.

S4: To a solution of S2 in dry THF (10 mL) was added Pd_2dba_3 (191 mg, 0.21 mmol, 5 mol%), PPh_3 (131 mg, 0.5 mmol, 12 mol%), LiCl (532 mg, 12.5 mmol, 3.0 equiv), (*S*)-2-Phenylglycinol (1.26 g, 4.4 mmol, 2.0 equiv). And then the reaction mixture stirred under CO (5 atm) at 80 °C for 12 h, filtered through celite and concentrated under vacuum to give crude product **S3.** To a solution of crude product in dry DCM (21 mL) at 0 °C, Et_3N (2.9 mL, 20.9 mmol, 5.0 equiv), MsCl (0.97 mL, 12.5 mmol, 3.0 equiv). The reaction mixture was stirred for 5 h at room temperature, then quenched with saturated NH_4Cl aqueous solution, and the resultant mixture was extracted with EtOAc three times. The combined organic layers were washed again with brine, dried ($NaSO_4$) and concentrated to give crude product **S4**.

L1: To a solution of products in dry DCM (31 mL), was added TFA (3.1 mL, 41.8 mmol, 10.0 equiv), and stirred for 5 h. The reaction mixture was concentrated under vacuum and then diluted with DCM, neutralized with saturated K_2CO_3 aqueous solution, and the resultant mixture was extracted with DCM three times. The combined organic layers were washed again with brine, dried ($NaSO_4$), concentrated to give crude products. The crude products were purified directly by column chromatography on silica gel (DCM: MeOH = 30:1 as eluent with 1% Et_3N) to afford the desired products **L1-L6**.

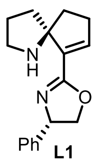

(*S*)-4-phenyl-2-((*R*)-1-azaspiro [4.4] non-6-en-6-yl)-4,5-dihydrooxazole (L1)

This compound was synthesized according to the procedure above, 70% yield, colorless liquid.

$[\alpha]^{27}_D$ = -13.0 (c = 1.0, $CHCl_3$).

^{1}H NMR (400 MHz, $CDCl_3$) δ 7.37-7.31 (m, 2H), 7.31-7.24 (m, 1H), 7.23-7.17 (m, 2H), 6.69 (t, *J* = 2.6 Hz, 1H), 5.27 (dd, *J* = 10.0, 8.0 Hz, 1H), 4.57 (dd, *J* = 9.8, 8.2 Hz, 1H), 4.44 (s, 1H), 4.00 (t, *J* = 8.2 Hz, 1H), 3.22-3.12 (m, 1H), 2.97-2.88 (m, 1H), 2.64-2.52 (m, 1H), 2.49-2.37 (m, 1H), 2.18-2.05 (m, 2H), 2.01-1.81 (m, 3H), 1.81-1.73 (m, 1H).

^{13}C NMR (100 MHz, $CDCl_3$) δ 160.9, 142.3, 142.0, 133.8, 128.5, 127.3, 126.2, 74.8, 73.2, 69.6, 46.0, 38.9, 35.7, 30.1, 25.4.

HRMS (ESI) calculated For $C_{17}H_{20}N_2O$ [M+H$^+$]: 269.1648, found 269.1647.

FT-IR (cm^{-1}): 3355, 2958, 1656, 1598, 1384, 1023, 700.

MS (EI) *m/z* (%): 226 (100), 107 (32), 120 (20), 269 (15), 134 (10), 147 (4).

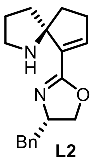

L2

(*S*)-4-benzyl-2-((*R*)-1-azaspiro[4.4]non-6-en-6-yl)-4,5-dihydrooxazole (L2)

This compound was synthesized according to the procedure above, 60% yield, colorless liquid.

[α]^{16}$_D$ = -26.0 (c = 1.0, $CHCl_3$).

^{1}H NMR (600 MHz, $CDCl_3$) δ 7.29 (t, *J* = 7.2 Hz, 2H), 7.22 (t, *J* = 7.5 Hz, 1H), 7.19 (d, *J* = 7.2 Hz, 2H), 6.59 (t, *J* = 2.7 Hz, 1H), 5.39 (br, 1H), 4.45 (p, *J* = 7.7 Hz, 1H), 4.19 (t, *J* = 8.7 Hz, 1H), 3.97 (dd, *J* = 8.4, 7.2 Hz, 1H), 3.20-3.13 (m, 1H), 3.07-3.01 (m, 1H), 2.98 (dd, *J* = 13.5, 6.3 Hz, 1H), 2.72 (dd, *J* = 13.5, 7.5 Hz, 1H), 2.60-2.52 (m, 1H), 2.45-2.36 (m, 1H), 2.24-2.17 (m, 1H), 2.09-2.01 (m, 1H), 2.00-1.94 (m, 2H), 1.94-1.87 (m, 1H), 1.78-1.73 (m, 1H).

^{13}C NMR (100 MHz, $CDCl_3$) δ 160.1, 141.0, 138.0, 134.9, 129.3, 128.4, 126.4, 74.6, 70.2, 67.8, 46.5, 41.78, 39.8, 35.8, 30.1, 25.9.

HRMS (ESI) calculated for $C_{18}H_{23}N_2O$ [M+H$^+$]: 283.1805, found: 283.1803.

FT-IR (cm^{-1}): 3395, 2957, 1657, 1599, 1453, 1386, 1051, 925, 754, 703.

MS (EI) *m/z* (%): 240 (100), 241 (18), 120 (15), 148 (14), 191 (13), 163 (7), 282 (4), 267 (3).

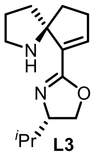

L3

(*S*)-4-isopropyl-2-((*R*)-1-azaspiro[4.4]non-6-en-6-yl)-4,5-dihydrooxazole (L3)

This compound was synthesized according to the procedure above, 45% yield, colorless liquid.

[α]^{28}$_D$ = -34.0 (c = 1.0, $CHCl_3$).

^{1}H NMR (600 MHz, $CDCl_3$) δ 6.59 (t, *J* = 2.7 Hz, 1H), 5.84 (br, 1H), 4.28-4.17 (m, 1H), 4.03-3.92 (m, 2H), 3.29-3.20 (m, 1H), 3.17-3.09 (m, 1H), 2.64-2.54 (m, 1H), 2.47-2.38 (m, 1H), 2.38-2.28 (m, 1H), 2.09-1.88 (m, 4H), 1.84-1.68 (m, 2H), 0.93 (d, *J* = 6.6 Hz, 3H), 0.89 (d, *J* = 7.2 Hz, 3H).

^{13}C NMR (150 MHz, $CDCl_3$) δ 159.7, 141.6, 132.8, 75.6, 72.0, 69.1, 46.0, 38.2, 35.7, 32.8, 30.2, 25.2, 18.5, 18.4.

HRMS (ESI) calculated for $C_{14}H_{23}N_2O$ [M+H$^+$]: 235.1805, found: 235.1804.

FT-IR (cm^{-1}): 3407, 2958, 2871, 1660, 1600, 1465, 1384, 1102, 1027, 924.

MS (EI) *m/z* (%): 192 (100), 120 (48), 148 (28), 191 (20), 163 (10).

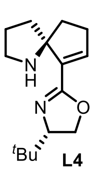

L4

(*S*)-4-(tert-butyl)-2-((*R*)-1-azaspiro[4.4]non-6-en-6-yl)-4,5-dihydrooxazole (L4)

This compound was synthesized according to the procedure above, 35% yield, colorless liquid.

[α]^{17}$_D$ = -37.0 (c = 1.0, $CHCl_3$).

^{1}H NMR (600 MHz, $CDCl_3$) δ 6.55 (t, *J* = 2.4 Hz, 1H), 5.07 (br, 1H), 4.14 (dd, *J* = 9.9, 8.7 Hz, 1H), 4.04 (dd, *J* = 8.4, 7.8 Hz, 1H), 3.92 (dd, *J* = 10.2, 7.8 Hz, 1H), 3.22 (dt, *J* = 11.0, 6.8 Hz, 1H), 3.03-2.97 (m, 1H), 2.59-2.51 (m, 1H), 2.44-2.36 (m, 1H), 2.20-2.13 (m, 1H), 2.06-1.99 (m, 1H), 1.99-1.92 (m, 2H), 1.92-1.85 (m, 1H), 1.78-1.71 (m, 1H), 0.88 (s, 9H).

^{13}C NMR (150 MHz, $CDCl_3$) δ 159.5, 140.9, 133.9, 75.7, 75.1, 67.3, 46.4, 39.2, 36.0, 33.9, 30.1, 25.8.

HRMS (ESI) calculated for $C_{15}H_{25}N_2O$ [M+H$^+$]: 249.1961, found: 249.1971.

FT-IR (cm^{-1}): 3415, 2956, 1661, 1601, 1384, 1363, 771.

MS (EI) *m/z* (%): 206 (100), 120 (48), 148 (28), 191 (20), 163 (13), 233 (5), 248 (5), 234 (6), 219 (5).

(*R*)-4-phenyl-2-((*R*)-1-azaspiro [4.4] non-6-en-6-yl)-4,5-dihydrooxazole (L5)

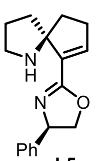

L5

This compound was synthesized according to the procedure above, 70% yield, colorless liquid.

[α]^{27}D = +44.0 (c = 1.0, $CHCl_3$).

^{1}H NMR (600 MHz, $CDCl_3$) δ 7.37-7.32 (m, 2H), 7.30-7.26 (m,1H), 7.24-7.20 (m, 2H), 6.72 (t, *J* = 3.0Hz, 1H), 5.25 (t, *J* = 9.3 Hz, 1H), 4.81-4.65 (br, 1H), 4.60 (dd, *J* = 9.9, 8.1Hz, 1H), 4.02 (t, *J* = 8.4 Hz, 1H), 3.26-3.16 (m, 1H), 3.05-2.97 (m, 1H), 2.66-2.56 (m, 1H), 2.51-2.41 (m, 1H), 2.26-2.14 (m, 1H), 2.06-1.97 (m, 2H), 1.97-1.84 (m, 2H), 1.80-1.73 (m, 1H).

^{13}C NMR (150 MHz, $CDCl_3$) δ 161.3, 142.5, 141.9, 133.3, 128.8, 127.6, 126.6, 75.3, 73.6, 70.0, 46.1, 38.5, 35.5, 30.2, 25.1.

HRMS (ESI) calculated for $C_{17}H_{21}N_2O$ [M+H^{+}]: 269.1648, found 269.1646.

FT-IR (cm^{-1}): 3284, 2959, 1656, 1598, 1452, 1384, 1260, 1023, 699.

MS (EI) *m/z* (%): 226 (100), 107 (32), 120 (20), 269 (15), 134 (10), 147 (4).

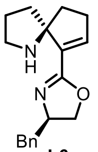

L6

(*R*)-4-benzyl-2-((*R*)-1-azaspiro[4.4]non-6-en-6-yl)-4,5-dihydrooxazole (L6)

This compound was synthesized according to the procedure above, 55% yield, colorless liquid.

[α]^{28}D = +54.0 (c = 1.0, $CHCl_3$).

^{1}H NMR (600 MHz, $CDCl_3$) δ 7.34-7.29 (m, 2H), 7.25-7.22 (m, 1H), 7.22-7.18 (m, 2H), 6.62 (t, *J* = 2.4 Hz, 1H), 5.98 (br, 1H), 4.42 (p, *J* = 7.8 Hz, 1H), 4.21 (dd, *J* = 9.0, 8.4 Hz, 1H), 3.97 (dd, *J* = 8.4, 7.8 Hz, 1H), 3.22-3.16 (m, 1H), 3.16-3.10 (m, 1H), 3.04 (dd, *J* = 13.5, 6.3 Hz, 1H), 2.71 (dd, *J* = 13.8, 8.4 Hz, 1H), 2.63-2.55 (m, 1H), 2.47-2.39 (m, 1H), 2.35-2.27 (m, 1H), 2.02-1.87 (m, 4H), 1.79-1.71 (m, 1H).

^{13}C NMR (150 MHz, $CDCl_3$) δ 160.2, 142.2, 137.9, 132.6, 129.1, 128.5, 126.5, 75.6, 70.8, 67.7, 45.7, 41.7, 37.8, 35.4, 30.2, 24.8.

HRMS (ESI) calculated for $C_{18}H_{23}N_2O$ [M+H^{+}]: 283.1805, found: 283.1801.

FT-IR (cm^{-1}): 3283, 2958, 1657, 1598, 1453, 1386, 1022, 703.

MS (EI) *m/z* (%): 240 (100), 241 (18), 120 (15), 148 (14), 191 (13), 163 (7), 282 (4), 267 (3).

4. Synthesis of Substituted (phenylmethylene)cyclopropanes[2]

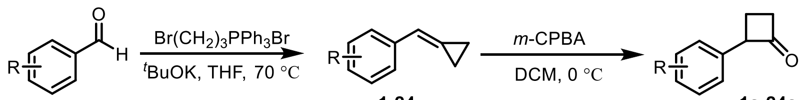

1-24 1a-24a

The substrate **1** was synthesized according to the literature.[2]

General procedure for the synthesis of (phenylmethylene)cyclopropanes 1

To a solution of (3-bromopropyl) triphenyl phosphonium bromide (5.1 g, 11.0 mmol, 1.1 equiv) in dry THF (35 mL) under argon atmosphere at 0 °C, a solution of Potassium tert-butoxide (22 ml, 22 mmol, 2.2 equiv) in THF (1 M) was added dropwise and then stirred at 70 °C for 1 h. Then a THF solution of benzaldehyde (10 mmol, 1.06 g, 1.0 equiv, 2.0 M in THF) was added dropwise at 0 °C and the mixture was refluxed again for 1 h. After full conversion, the mixture was cooled down to room temperature, layered with petroleum ether (50 mL) and the suspension filtered over celite washing thoroughly with petroleum ether. The filtrate was concentrated and the residue purified by chromatography on silica gel eluting with petroleum ether and ethyl acetate to afford various (phenylmethylene)cyclopropanes.

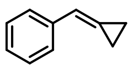
1

This compound was synthesized according to the procedure above, 94% yield, colorless liquid.

^{1}H NMR (400 MHz, $CDCl_3$) δ 7.53 (d, *J* = 7.6 Hz, 2H), 7.32 (t, *J* = 7.6 Hz, 2H), 7.28-7.12 (m, 1H), 6.74 (s, 1H), 1.48-1.34 (m, 2H), 1.26-1.08 (m, 2H).

^{13}C NMR (100 MHz, $CDCl_3$) δ 138.2, 128.4, 126.7 126.6, 124.3, 118.2, 4.2, 0.5.

HRMS (ESI) calculated for $C_{10}H_{11}$ [M+H$^+$]: 131.0855; found: 131.0854.

FT-IR (cm^{-1}) 3060, 2975, 1764, 1451, 740, 694, 509.

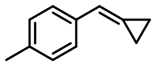
2

This compound was synthesized according to the procedure above, 96% yield, colorless liquid.

^{1}H NMR (600 MHz, $CDCl_3$) δ 7.46-7.39 (m, 2H), 7.12 (d, *J* = 7.8 Hz, 2H), 6.71 (t, *J* = 2.1 Hz, 1H), 2.33 (s, 3H), 1.42-1.36 (m, 2H), 1.18-1.12 (m, 2H).

^{13}C NMR (150 MHz, $CDCl_3$) δ 136.3, 135.5, 129.1, 126.5, 123.0, 118.0, 21.2, 4.1, 0.5.

HRMS (ESI) calculated for $C_{11}H_{13}$ [M+H$^+$]: 145.1012; found: 145.1005.

FT-IR (cm^{-1}): 3444, 3005, 2921, 1782, 1704, 1513, 1176, 1020, 812, 514.

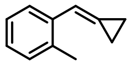
3

This compound was synthesized according to the procedure above, 88% yield, colorless liquid.

^{1}H NMR (400 MHz, $CDCl_3$) δ 7.73 (d, *J* = 8.0 Hz, 1H), 7.20-7.06 (m, 3H), 6.95 (p, *J* = 2.1 Hz, 1H), 2.38 (s, 3H), 1.42-1.35 (m, 2H), 1.19-1.12 (m, 2H).

^{13}C NMR (100 MHz, $CDCl_3$) δ 136.5, 134.8, 130.2, 126.6, 125.8, 125.8, 124.9, 115.4, 19.7, 4.1, 0.8.

HRMS (ESI) calculated for $C_{11}H_{13}$ [M+H$^+$]: 145.1012; found: 145.1011.

FT-IR (cm^{-1}): 3444, 3003, 2917, 1777, 1704, 1510, 1173, 810, 517.

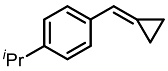

4

This compound was synthesized according to the procedure above, 76% yield, yellow liquid.

^{1}H NMR (600 MHz, $CDCl_3$) δ 7.46 (d, *J* = 8.4 Hz, 2H), 7.19 (d, *J* = 8.4 Hz, 2H), 6.74-6.70 (m, 1H), 2.89 (hept, *J* = 6.9 Hz, 1H), 1.42-1.37 (m, 2H), 1.25 (d, *J* = 6.6 Hz, 6H), 1.18-1.13 (m, 2H).

^{13}C NMR (150 MHz, $CDCl_3$) δ 147.4, 135.9, 126.6, 126.5, 123.2, 118.0, 33.8, 24.0, 4.1, 0.5.

HRMS (ESI) calculated for $C_{13}H_{17}$ [M+H$^+$]: 173.1325, found: 173.1325.

FT-IR (cm^{-1}): 2959, 2869, 1785, 1769, 1512, 1459, 1054, 844, 549.

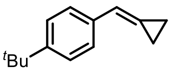

5

This compound was synthesized according to the procedure above, 91% yield, white solid, m. p.: 51-53 °C.

^{1}H NMR (600 MHz, $CDCl_3$) δ 7.50-7.44 (m, 2H), 7.38-7.33 (m, 2H), 6.72 (p, *J* = 2.1 Hz, 1H), 1.42-1.38 (m, 2H), 1.32 (s, 9H), 1.18-1.14 (m, 2H).

^{13}C NMR (150 MHz, $CDCl_3$) δ 149.6, 135.5, 126.3, 125.3, 123.3, 117.9, 34.5, 31.3, 4.1, 0.5.

HRMS (ESI) calculated for $C_{14}H_{19}$ [M+H$^+$]: 187.1481, found: 187.1479.

FT-IR (cm^{-1}): 3466, 2963, 2868, 1783, 1363, 1268, 1108, 843, 571.

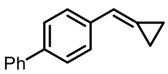

6

This compound was synthesized according to the procedure above, 89% yield, white solid, m. p.: 64-68 °C.

^{1}H NMR (600 MHz, $CDCl_3$) δ 7.67-7.53 (m, 6H), 7.46-7.39 (m, 2H), 7.35-3.30 (m, 1H), 6.19 (p, *J* = 2.0 Hz, 1H), 1.50-1.39 (m, 2H), 1.25-1.12 (m, 2H).

^{13}C NMR (150 MHz, $CDCl_3$) δ 140.9, 139.4, 137.3, 128.7, 127.1, 127.1, 127.0, 126.9, 124.6, 117.9, 4.2, 0.6.

HRMS (ESI) calculated for $C_{16}H_{15}$ [M+H$^+$]: 207.1168, found: 207.1169.

FT-IR (cm^{-1}): 3437, 3031, 2972, 1486, 1426, 847, 757, 692, 493.

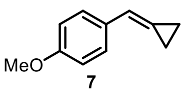

7

This compound was synthesized according to the procedure above, 92% yield, yellow liquid.

^{1}H NMR (400 MHz, $CDCl_3$) δ 7.45 (d, *J* = 8.4 Hz, 2H), 6.90-6.81 (m, 2H), 6.68 (t, *J* = 2.0 Hz, 1H), 3.77 (s, 3H), 1.41-1.31 (m, 2H), 1.19-1.06 (m, 2H).

^{13}C NMR (100 MHz, $CDCl_3$) δ 158.5, 131.1, 127.6, 121.5, 117.5, 113.8, 55.1, 3.9, 0.4.

HRMS (ESI) calculated for $C_{11}H_{12}NaO$ [M+Na$^+$]: 183.0780, found: 183.0779.

FT-IR (cm^{-1}): 3467, 3000, 2836, 1718, 1608, 1511, 1441, 1249, 1173, 1033, 838, 541.

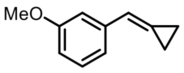

8

This compound was synthesized according to the procedure above, 93% yield, yellow liquid.

^{1}H NMR (400 MHz, $CDCl_3$) δ 7.24 (t, *J* = 8.0 Hz, 1H), 7.11 (d, *J* = 8.0 Hz, 2H), 6.80-6.74 (m, 1H), 6.72 (t, *J* = 1.8 Hz, 1H), 3.81 (s, 3H), 1.46-1.37 (m, 2H), 1.21-1.12 (m, 2H).

^{13}C NMR (100 MHz, $CDCl_3$) δ 159.7, 139.7, 129.3, 124.7, 119.4, 118.1, 112.2, 111.9, 55.1, 4.2, 0.5.

HRMS (ESI) calculated for $C_{11}H_{12}NaO$ [M+Na$^+$]: 183.0780, found: 183.0786.

FT-IR (cm^{-1}): 2956, 2834, 1780, 1735, 1598, 1490, 1263, 1155, 1043, 774, 690.

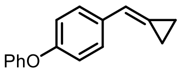

9

This compound was synthesized according to the procedure above, 83% yield, yellow liquid.

^{1}H NMR (400 MHz, $CDCl_3$) δ 7.49 (d, *J* = 8.8 Hz, 2H), 7.40-7.27 (m, 2H), 7.08 (t, *J* = 7.4 Hz, 1H), 7.04-6.92 (m, 4H), 6.72 (t, *J* = 1.8 Hz, 1H), 1.44-1.32 (m, 2H), 1.21-1.07 (m, 2H).

^{13}C NMR (100 MHz, $CDCl_3$) δ 157.4, 155.9, 133.6, 129.7, 127.8, 123.2, 123.1, 119.0, 118.6, 117.4, 4.0, 0.5.

HRMS (ESI) calculated for $C_{16}H_{15}O$ [M+H$^+$]: 223.1117, found: 223.1124.

FT-IR (cm^{-1}): 3447, 3039, 1782, 1588, 1488, 1240, 1165, 871, 692.

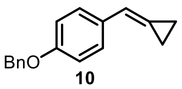

10

This compound was synthesized according to the procedure above, 94% yield, white solid, m. p.: 67-70 °C.

^{1}H NMR (600 MHz, $CDCl_3$) δ 7.48-7.44 (m, 2H), 7.44-7.41 (m, 2H), 7.39-7.35 (m, 2H), 7.34-7.28 (m, 1H), 6.98-6.89 (m, 2H), 6.68 (t, *J* = 2.1 Hz, 1H), 5.05 (s, 2H), 1.44-1.31 (m, 2H), 1.14 (m, 2H).

^{13}C NMR (150 MHz, $CDCl_3$) δ 157.7, 137.0, 131.4, 128.5, 127.9, 127.7, 127.4, 121.8, 117.5, 114.8, 70.0, 4.0, 0.5.

HRMS (ESI) calculated for $C_{17}H_{16}NaO$ [M+Na$^+$]: 259.1093, found: 259.1084.

FT-IR (cm^{-1}): 3437, 2968, 1606, 1510, 1249, 1012, 837, 741, 695, 529.

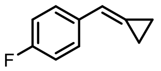

11

This compound was synthesized according to the procedure above, 77% yield, colorless liquid.

^{1}H NMR (400 MHz, $CDCl_3$) δ 7.52-7.41 (m, 2H), 7.05-6.93 (m, 2H), 6.69 (p, *J* = 2.0 Hz, 1H), 1.42-1.29 (m, 2H), 1.24-1.10 (m, 2H).

^{13}C NMR (100 MHz, $CDCl_3$) δ 161.8 (d, *J* = 244 Hz), 134.4 (d, *J* = 3.4 Hz), 127.9 (d, *J* = 7.9 Hz), 123.8 (d, *J* = 2.6 Hz), 117.1, 115.2 (d, *J* = 21.4 Hz), 4.0, 0.5.

^{19}F NMR (376 MHz, $CDCl_3$) δ -115.73.

HRMS (ESI) calculated for $C_{10}H_{10}F$ [M+H$^+$]: 149.0761, found: 149.0760.

FT-IR (cm^{-1}): 3075, 2926, 1765, 1600, 1509, 1226, 1157, 836.

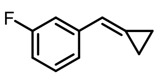

12

This compound was synthesized according to the procedure above, 72% yield, colorless liquid.

^{1}H NMR (400 MHz, $CDCl_3$) δ 7.27-7.19 (m, 3H), 6.92-6.83(m, 1H), 6.70 (s, 1H), 1.45-1.34 (m, 2H), 1.23-1.09 (m, 2H).

^{13}C NMR (100 MHz, $CDCl_3$) δ 163.1 (d, *J* = 242.9 Hz), 140.5 (d, *J* = 7.6 Hz), 129.8 (d, *J* = 8.4 Hz), 126.0, 122.45 (d, *J* = 2.6 Hz), 117.3 (d, *J* = 2.7 Hz), 113.4 (d, *J* = 21.4 Hz), 112.8 (d, *J* = 21.6 Hz), 4.2, 0.6.

^{19}F NMR (376 MHz, $CDCl_3$) δ -113.76.

HRMS (ESI) calculated for $C_{10}H_{10}F$ [M+H$^+$]: 149.0761, found: 149.0760.

FT-IR (cm^{-1}): 3073, 2925, 1761, 1590, 1523, 1488, 1448, 1250, 1162, 1142, 785.

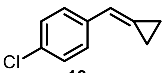

13

This compound was synthesized according to the procedure above, 79% yield, colorless liquid.

^{1}H NMR (600 MHz, $CDCl_3$) δ 7.42 (d, *J* = 8.4 Hz, 2H), 7.26 (d, *J* = 8.4 Hz, 2H), 6.68 (t, *J* = 1.8 Hz 1H), 1.44-1.32 (m, 2H), 1.26-1.12 (m, 2H).

^{13}C NMR (150 MHz, $CDCl_3$) δ 136.7, 132.2, 128.5, 127.7, 125.1, 117.2, 4.1, 0.6.

HRMS (ESI) calculated for $C_{10}H_{10}Cl$ [M+H$^+$] 165.0466, found: 165.0465.

FT-IR (cm^{-1}): 3466, 2925, 1743, 1491, 1091, 1014, 831.

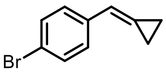

14

This compound was synthesized according to the procedure above, 91% yield, white solid, m. p.: 50-53 °C.

^{1}H NMR (600 MHz, $CDCl_3$) δ 7.42 (d, *J* = 8.4 Hz, 2H), 7.37 (d, *J* = 8.4 Hz, 2H), 6.68 (p, *J* = 2.1 Hz, 1H), 1.45-1.34 (m, 2H), 1.23-1.11 (m, 2H).

^{13}C NMR (150 MHz, $CDCl_3$) δ 137.1, 131.5, 128.1, 125.3, 120.3, 117.2, 4.2, 0.6.

HRMS (ESI) calculated for $C_{10}H_{10}Br$ [M+H$^+$]: 208.9960, 210.9940, found: 208.9960, 210.9942.

FT-IR (cm^{-1}): 3455, 3044, 2967, 1745, 1486, 1401, 1105, 840, 511.

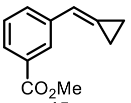

15

This compound was synthesized according to the procedure above, 77% yield, yellow liquid.

^{1}H NMR (600 MHz, $CDCl_3$) δ 8.16 (s, 1H), 7.87 (d, *J* = 7.8 Hz, 1H), 7.73 (d, *J* = 7.8 Hz, 1H), 7.38 (t, *J* = 7.8 Hz, 1H), 6.6.78 (p, *J* = 1.8 Hz, 1H), 3.92 (s, 3H), 1.56-1.39 (m, 2H), 1.23-1.16 (m, 2H).

^{13}C NMR (150 MHz, $CDCl_3$) δ 167.2, 138.5, 130.7, 130.3, 128.4, 127.7, 127.7, 125.9, 117.4, 52.1, 4.2, 0.6.

HRMS (ESI) calculated for $C_{12}H_{13}O_2$ [M+H$^+$]: 189.0910, found: 189.0909.

FT-IR (cm^{-1}): 2976, 2950, 1723, 1445, 1292, 1204, 1107, 747.

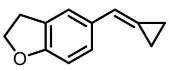

16

This compound was synthesized according to the procedure above, 92% yield, orange solid, m. p.: 80-83 °C.

^{1}H NMR (400 MHz, $CDCl_3$) δ 7.42 (s, 1H), 7.26 (d, *J* = 8.0 Hz, 1H), 6.75 (d, *J* = 8.0 Hz, 1H), 6.68 (*J* = 2.0 Hz, 1H), 4.57 (t, *J* = 8.8 Hz, 2H), 3.21 (t, *J* = 8.8 Hz, 3H), 1.46-1.33 (m, 2H), 1.23-1.06 (m, 2H).

^{13}C NMR (100 MHz, $CDCl_3$) δ 159.1, 131.2, 127.2, 126.8, 122.8, 120.9, 117.9, 109.1, 71.3, 29.7, 4.1, 0.5.

HRMS (ESI) calculated for $C_{12}H_{13}O$ [M+H$^+$]: 173.0961, found: 173.0961.

FT-IR (cm^{-1}): 3437, 2967, 2895, 1607, 1492, 1237, 937, 826, 804, 425.

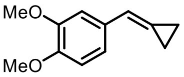

17

This compound was synthesized according to the procedure above, 91% yield, yellow solid, m. p.: 61-64 °C.

^{1}H NMR (600 MHz, $CDCl_3$) δ 7.14 (d, *J* = 1.8 Hz, 1H), 7.03 (dd, *J* = 8.1, 2.1 Hz, 1H), 6.83 (d, *J* = 7.8 Hz, 1H), 6.68 (p, *J* = 2.1 Hz, 1H), 3.89 (s, 3H), 3.87 (s, 3H), 1.43-1.36 (m, 2H), 1.19-1.14 (m, 2H).

^{13}C NMR (150 MHz, $CDCl_3$) δ 148.9, 148.0, 131.5, 121.8, 119.3, 117.7, 111.0, 109.2, 55.8, 55.6, 3.8, 0.4.

HRMS (ESI) calculated for $C_{12}H_{14}NaO_2$ [M+Na$^+$]: 213.0886, found: 213.0881.

FT-IR (cm^{-1}): 3443, 2968, 2835, 1779, 1514, 1264, 1232, 1138, 1026.

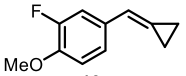

18

This compound was synthesized according to the procedure above, 98% yield, colorless liquid.

^{1}H NMR (600 MHz, $CDCl_3$) δ 7.31 (dd, *J* = 12.6, 1.8 Hz, 1H), 7.14 (d, *J* = 8.4 Hz, 1H), 6.89 (t, *J* = 8.7 Hz, 1H), 6.63 (s, 1H), 3.86 (s, 3H), 1.40-1.34 (m, 2H), 1.18-1.13 (m, 2H).

^{13}C NMR (150 MHz, $CDCl_3$) δ 152.5 (d, *J* = 161.9 Hz), 146.4 (d, *J* = 10.8 Hz), 132.0 (d, *J* = 6.8 Hz), 123.3, 122.6 (d, *J* = 3.0 Hz), 116.8 (d, *J* = 2.0 Hz), 113.5 (d, *J* = 19.1 Hz), 113.2, 56.2, 3.9, 0.5.

^{19}F NMR (376 MHz, $CDCl_3$) δ -135.66.

HRMS (ESI) calculated for $C_{11}H_{12}FO$ [M+H$^+$]: 179.0867, found: 179.0860.

FT-IR (cm^{-1}): 3048, 2976, 1784, 1490, 1090, 1068, 841, 511.

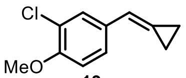

19

This compound was synthesized according to the procedure above, 98% yield, yellow liquid.

^{1}H NMR (400 MHz, $CDCl_3$) δ 7.56 (d, *J* = 2.0 Hz, 1H), 7.32 (dd, *J* = 8.6, 2.2 Hz, 1H), 6.86 (d, *J* = 8.4 Hz, 1H), 6.62 (t, *J* = 2.0 Hz, 1H), 3.88 (s, 3H), 1.54-1.34 (m, 2H), 1.25-1.04 (m, 2H).

^{13}C NMR (100 MHz, $CDCl_3$) δ 153.7, 132.1, 127.9, 125.9, 123.3, 122.5, 116.5, 111.9, 56.1, 4.0, 0.5.

HRMS (ESI) calculated for $C_{11}H_{12}ClO$ [M+H$^+$]: 195.0571, found: 195.0573.

FT-IR (cm^{-1}): 2974, 1600, 1500, 1281, 1258, 1062, 1023, 819.

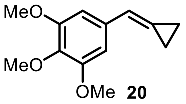

20

This compound was synthesized according to the procedure above, 96% yield, yellow solid, m. p.: 64-67 °C.

^{1}H NMR (400 MHz, $CDCl_3$) δ 6.78 (s, 2H), 6.66 (p, *J* = 2.0 Hz, 1H), 3.88 (s, 6H), 3.85 (s, 3H), 1.45-1.37 (m, 2H), 1.22-1.15 (m, 2H).

^{13}C NMR (100 MHz, $CDCl_3$) δ 153.1, 137.0, 134.0, 123.5, 118.0, 103.5, 60.8, 55.9, 3.9, 0.4.

HRMS (ESI) calculated for $C_{13}H_{16}NaO_3$ [M+Na$^+$]: 243.0992, found: 243.0983.

FT-IR (cm^{-1}): 2966, 2835, 1583, 1506, 1415, 1236, 1129, 1005, 842.

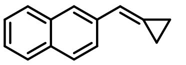

21

This compound was synthesized according to the procedure above, 89.3% yield, yellow solid, m. p.: 64-69 °C.

^{1}H NMR (400 MHz, $CDCl_3$) δ 7.88-7.71 (m, 5H), 7.48-7.35 (m, 2H), 6.91 (t, *J* = 1.8 Hz 1H), 1.57-1.43 (m, 2H), 1.28-1.14 (m, 2H).

^{13}C NMR (100 MHz, $CDCl_3$) δ 135.9, 133.7, 132.6, 127.9, 127.9, 127.6, 126.0, 125.7, 125.4, 124.9, 124.5, 118.5, 4.4, 0.7.

HRMS (ESI) calculated for $C_{14}H_{13}$ [M+H$^+$]: 181.1012, found: 181.1012.

FT-IR (cm^{-1}): 3051, 2969, 970, 900, 860, 739, 478.

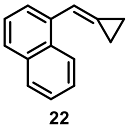

22

This compound was synthesized according to the procedure above, 89% yield, yellow liquid.

^{1}H NMR (400 MHz, $CDCl_3$) δ 8.19 (d, *J* = 8.4 Hz, 1H), 7.87-7.78 (m, 2H), 7.71 (d, *J* = 8.0 Hz, 1H), 7.53-7.40 (m, 4H), 1.41-1.37 (m, 2H), 1.28-1.18 (m, 2H).

^{13}C NMR (100 MHz, $CDCl_3$) δ 134.6, 133.8, 131.0, 128.5, 127.1, 126.7, 125.7, 125.5, 125.5, 123.8, 123.5, 114.6, 4.1, 1.5.

HRMS (ESI) calculated for $C_{14}H_{13}$ [M+H$^+$]: 181.1012, found: 181.1011.

FT-IR (cm^{-1}): 3404, 3056, 2971, 1554, 1360, 970, 907, 860, 743, 471.

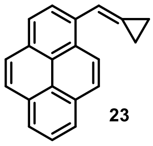
23

This compound was synthesized according to the procedure above, 82% yield, yellow solid, m. p.: 126-129 ℃.

^{1}H NMR (400 MHz, $CDCl_3$) δ 8.42 (d, J = 8.8 Hz, 1H), 8.10 (t, J = 7.8 Hz, 3H), 8.04 (d, J = 9.2 Hz, 1H), 7.98 (s, 2H), 7.94 (t, J = 7.6 Hz, 1H), 7.79 (t, J = 1.8 Hz, 1H), 1.61-1.51 (m, 2H), 1.36-1.28 (m, 2H).

^{13}C NMR (100 MHz, $CDCl_3$) δ 132.1, 131.5, 130.9, 130.1, 127.6, 127.5, 127.3, 127.2, 126.8, 125.8, 125.0, 125.0, 125.0, 124.7, 124.1, 122.9, 115.0, 4.5, 1.4.

HRMS (ESI) calculated for $C_{20}H_{15}$ [M+H$^+$]: 255.1168, found: 255.1166.

FT-IR (cm^{-1}): 3439, 3039, 2968, 1600, 1260, 843, 796, 711.

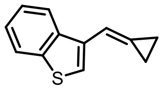
24

This compound was synthesized according to the procedure above, 87% yield, yellow liquid.

^{1}H NMR (600 MHz, $CDCl_3$) δ 7.96 (d, J = 8.4 Hz, 1H), 7.84 (dt, J = 8.0, 0.9 Hz, 1H), 7.53 (s, 1H), 7.41-7.37 (m, 1H), 7.36-7.31 (m, 1H), 7.07 (p, J = 2.0 Hz, 1H), 1.42-1.37 (m, 2H), 1.30-1.26 (m, 2H).

^{13}C NMR (150 MHz, $CDCl_3$) δ 140.2, 137.9, 134.1, 125.3, 124.3, 124.0, 122.8, 121.8, 120.8, 110.5, 4.2, 2.1.

HRMS (ESI) calculated for $C_{12}H_{11}S$ [M+H$^+$]: 188.0576, found: 187.0580.

FT-IR (cm^{-1}) 3426, 3009, 1615, 1265, 841, 797, 762, 711, 732, 421.

5. General procedure for the synthesis of cyclobutanones a[3]

To a solution of (cyclopropylidenemethyl)benzene (1.04 g, 8 mmol, 1.0 equiv) in DCM (80 mL, 0.1 M) at 0 °C was added a DCM (27 mL, 0.3 M) solution of *m*-CPBA (1.62 g, 85%, 8 mmol, 1.0 equiv) dropwise and stirred for 2-8 h. After full conversion, the solution was diluted with a saturated solution of aqueous Na_2SO_3 (15 mL) and extracted with DCM three times. The combined organic layers were washed successively with a saturated solution of aqueous Na_2CO_3 three times and brine, dried over Na_2SO_4 and concentrated in vacuum. The crude products were purified directly by column chromatography on silica gel with a mixture of petroleum ether and ethyl acetate to give various cyclobutanones.

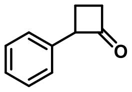
1a

This compound was synthesized according to the procedure above, 65% yield, colorless liquid.

^{1}H NMR (400 MHz, $CDCl_3$) δ 7.31-7.22 (m, 2H), 7.22-7.09 (m, 3H), 4.53-4.39 (m, 1H), 3.24-3.06 (m, 1H), 3.03-2.87 (m, 1H), 2.46 (qd, J = 10.7, 4.9 Hz, 1H), 2.25-2.03 (m, 1H).

^{13}C NMR (100 MHz, $CDCl_3$) δ 207.8, 136.4, 128.6, 126.9, 126.9, 64.5, 44.8, 17.6.

HRMS (ESI) calculated for $C_{10}H_{10}NaO$ [M+Na$^+$]: 169.0624, found: 169.0624.

FT-IR (cm^{-1}) 3027, 2962, 1781, 1495, 1070, 1031, 746, 697.

MS (EI) *m/z* (%): 104 (100), 105 (32), 103 (20), 117 (16), 115 (13), 146 (5)

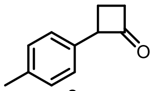
2a

This compound was synthesized according to the procedure above, 64% yield, colorless liquid.

^{1}H NMR (400 MHz, $CDCl_3$) δ 7.10 (s, 4H), 4.49-4.36 (m, 1H), 3.20-3.07 (m, 1H), 3.01-2.88 (m, 1H), 2.44 (qd, J = 10.6, 4.9 Hz, 1H), 2.29 (s, 3H), 2.13 (m, 1H).

^{13}C NMR (100 MHz, $CDCl_3$) δ 207.7, 136.3, 133.4, 129.0, 126.6, 64.0, 44.5, 20.8, 17.5.

HRMS (ESI) calculated for $C_{11}H_{12}NaO$ [M+Na$^+$]: 183.0780, found: 183.0784.

FT-IR (cm^{-1}): 2923, 1781, 1514, 1070, 812, 476.

MS (EI) *m/z* (%): 118 (100), 117 (65), 119 (40), 61 (36), 91 (30), 70 (30), 132 (7), 160 (3).

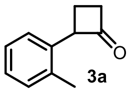
3a

This compound was synthesized according to the procedure above, 77% yield, colorless liquid.

^{1}H NMR (400 MHz, $CDCl_3$) δ 7.28-7.23 (m, 1H), 7.19-7.11 (m, 3H), 4.72-4.59 (m, 1H), 3.25-3.13 (m, 1H), 3.07-2.96 (m, 1H), 2.51 (qd, *J* = 10.7, 5.0 Hz, 1H), 2.30 (s, 3H), 2.21-2.09 (m, 1H).

^{13}C NMR (100 MHz, $CDCl_3$) δ 208.2, 135.9, 135.2, 130.4, 127.0, 126.0, 62.5, 44.5, 19.7, 17.7.

HRMS (ESI) calculated for $C_{11}H_{12}NaO$ [M+Na$^+$]: 183.0780, found: 183.0782.

FT-IR (cm^{-1}): 2917, 1777, 1514, 1075, 810, 513, 477.

MS (EI) *m/z* (%): 118 (100), 117 (65), 119 (40), 61 (36), 91 (30), 70 (30), 132 (7), 160 (3).

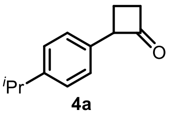
4a

This compound was synthesized according to the procedure above, 89% yield, colorless liquid.

^{1}H NMR (400 MHz, $CDCl_3$) δ 7.23-7.13 (m, 4H), 4.55-4.44 (m, 1H), 3.28-3.13 (m, 1H), 3.09-2.94 (m, 1H), 2.88 (hept, *J* = 6.9 Hz, 1H), 2.51 (qd, *J* = 10.6, 5.0 Hz, 1H), 2.28-2.15 (m, 1H), 1.23 (d, *J* = 7.2 Hz, 6H).

^{13}C NMR (100 MHz, $CDCl_3$) δ 208.1, 147.6, 133.8, 126.9, 126.7, 64.3, 44.8, 33.7, 23.9, 17.7.

HRMS (ESI) calculated for $C_{13}H_{17}O$ [M+H$^+$]: 189.1274, found: 189.1277.

FT-IR (cm^{-1}): 2961, 2871, 1782, 1683, 1605, 1183, 1056, 826.

MS (EI) *m/z* (%): 131 (100), 146 (78), 91 (23),145 (23), 115 (21), 117 (19), 188 (3).

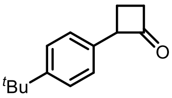
5a

This compound was synthesized according to the procedure above, 78% yield, white solid, m. p.: 42-45 °C.

^{1}H NMR (400 MHz, $CDCl_3$) δ 7.41-7.29 (m, 2H), 7.18 (d, *J* = 8.4 Hz, 2H), 4.55-4.44 (m, 1H), 3.27-3.13 (m, 1H), 3.09-2.95 (m, 1H), 2.50 (qd, *J* = 10.6, 5.0 Hz, 1H), 2.30-2.15 (m, 1H), 1.30 (s, 9H).

^{13}C NMR (100 MHz, $CDCl_3$) δ 208.1, 149.8, 133.4, 126.6, 125.5, 64.2, 44.8, 34.4, 31.3, 17.7.

HRMS (ESI) calculated for $C_{14}H_{18}NaO$ [M+Na$^+$]: 225.1250, found: 225.1253.

FT-IR (cm^{-1}): 2962, 2868, 1784, 1512, 1070, 826, 574.

MS (EI) *m/z* (%): 145 (100), 160 (24), 146 (22), 117 (18), 115 (12), 131 (8), 202 (2).

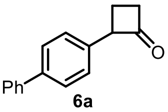
6a

This compound was synthesized according to the procedure above, 72% yield, white solid, m. p.: 86-87 °C.

^{1}H NMR (400 MHz, $CDCl_3$) δ 7.60-7.51 (m, 4H), 7.47-7.38 (m, 2H), 7.36-7.28 (m, 3H), 4.66-4.46 (m, 1H), 3.34-3.15 (m, 1H), 3.12-2.94 (m, 1H), 2.55 (qd, *J* = 10.6, 5.0 Hz, 1H), 2.35-2.15 (m, 1H).

^{13}C NMR (100 MHz, $CDCl_3$) δ 207.7, 140.7, 139.9, 135.4, 128.7, 127.3, 127.3, 127.2, 127.0, 64.2, 44.9, 17.7.

HRMS (ESI) calculated for $C_{16}H_{14}NaO$ [M+Na$^+$]: 245.0937, found: 245.0937.

FT-IR (cm^{-1}): 3431, 3011, 2977, 1770, 1405, 1074, 833, 744, 688.

MS (EI) *m/z* (%): 180 (100), 181 (54), 152 (43), 165 (38), 178 (33), 179 (25), 115 (18), 194 (10), 198 (8), 222 (7).

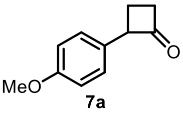
7a

This compound was synthesized according to the procedure above, 68% yield, yellow liquid.

^{1}H NMR (600 MHz, $CDCl_3$) δ 7.15 (d, *J* = 8.4 Hz, 2H), 6.86 (d, *J* = 9.0 Hz, 2H), 4.46 (m, 1H), 3.77 (s, 3H), 3.25-3.15 (m, 1H), 3.05-2.96 (m, 1H), 2.50 (qd, *J* = 10.8, 4.8 Hz, 1H), 2.21-2.12 (m, 1H).

^{13}C NMR (100 MHz, $CDCl_3$) δ 208.3, 158.5, 128.7, 128.0, 114.0, 63.8, 55.2, 44.6, 17.9.

HRMS (ESI) calculated for $C_{11}H_{12}NaO_2$ [M+Na$^+$]: 199.0730, found: 199.0725.

FT-IR (cm^{-1}): 2959, 2836, 1781, 1513, 1282, 1248, 1178, 1072, 828.

MS (EI) *m/z* (%): 134 (100), 91 (43), 119 (30), 135 (28), 77 (25), 61 (22), 147 (17), 176 (4).

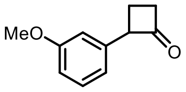

8a

This compound was synthesized according to the procedure above, 70% yield, yellow liquid.

^{1}H NMR (400 MHz, $CDCl_3$) δ 7.24 (t, *J* = 7.8 Hz, 1H), 6.89-6.73 (m, 3H), 4.56-4.41 (m, 1H), 3.78 (s, 3H), 3.27-3.12 (m, 1H), 3.09-2.92 (m, 1H), 2.50 (qd, *J* = 10.6, 5.0 Hz, 1H), 2.30-2.13 (m, 1H).

^{13}C NMR (100 MHz, $CDCl_3$) δ 207.5, 159.7, 137.9, 129.5, 119.1, 112.6, 112.2, 64.3, 55.1, 44.7, 17.5.

HRMS (ESI) calculated for $C_{11}H_{12}NaO_2$ [M+Na$^+$]: 199.0730, found: 199.0726.

FT-IR (cm^{-1}): 2961, 2836, 1780, 1600, 1491, 1251, 1154, 1071, 783, 694.

MS (EI) *m/z* (%): 134 (100), 135 (45), 91 (33), 77 (22), 176 (7).

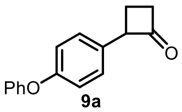

9a

This compound was synthesized according to the procedure above, 63% yield, yellow liquid.

^{1}H NMR (400 MHz, $CDCl_3$) δ 7.36-7.28 (m, 2H), 7.24-7.17 (m, 2H), 7.14-7.06 (m, 1H), 7.02-6.93 (m, 4H), 4.62-4.39 (m, 1H), 3.35-3.15 (m, 1H), 3.10-2.93 (m, 1H), 2.54 (qd, *J* = 10.6, 5.0 Hz, 1H), 2.29-2.12 (m, 1H).

^{13}C NMR (100 MHz, $CDCl_3$) δ 207.9, 157.2, 156.1, 131.3, 129.7, 128.3, 123.2, 119.0, 118.7, 63.9, 44.8, 17.9.

HRMS (ESI) calculated for $C_{16}H_{14}NaO_2$ [M+Na$^+$]: 261.0886, found: 261.0888.

FT-IR (cm^{-1}): 3062, 1782, 1588, 1506, 1488, 1238, 1071, 868, 755, 693.

MS (EI) *m/z* (%): 196 (100), 77 (46), 117 (25), 135 (25), 115 (23), 197 (22), 210 (7), 238 (3).

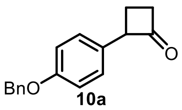

10a

This compound was synthesized according to the procedure above, 63% yield, white solid, m. p.: 56-57 °C.

^{1}H NMR (400 MHz, $CDCl_3$) δ 7.45-7.34 (m, 4H), 7.33 (m, 1H), 7.18-7.13 (m, 2H), 7.00-6.84 (m, 2H), 5.04 (s, 2H), 4.56-4.37 (m, 1H), 3.33-3.12 (m, 1H), 3.07-2.93 (m, 1H), 2.51 (qd, *J* = 10.6, 4.9 Hz, 1H), 2.28-2.07 (m, 1H).

^{13}C NMR (100 MHz, $CDCl_3$) δ 208.4, 157.7, 136.9, 128.9, 128.5, 128.0, 127.9, 127.4, 114.9, 69.9, 63.8, 44.7, 18.0.

HRMS (ESI) calculated for $C_{17}H_{16}NaO_2$ [M+Na$^+$]: 275.1043, found: 275.1034.

FT-IR (cm^{-1}): 3535, 3033, 2870, 1780, 1609, 1511, 1244, 1201, 1072, 826, 698.

MS (EI) *m/z* (%): 91 (100), 65 (12), 92 (7), 77 (6), 90 (6), 210 (6), 252 (2).

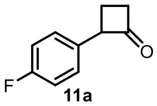

11a

This compound was synthesized according to the procedure above, 85% yield, colorless liquid.

^{1}H NMR (400 MHz, $CDCl_3$) δ 7.20 (dd, *J* = 8.4, 5.6 Hz, 2H), 7.08-6.91 (m, 2H), 4.69-4.34 (m, 1H), 3.31-3.11 (m, 1H), 3.10-2.90 (m, 1H), 2.53 (qd, *J* = 10.8, 4.8 Hz, 1H), 2.31-2.08 (m, 1H).

^{13}C NMR (100 MHz, $CDCl_3$) δ 207.5, 161.7 (d, *J* = 243.7Hz), 132.2 (d, *J* = 3.2 Hz), 128.4 (d, *J* = 7.9 Hz), 115.4 (d, *J* = 21.3 Hz), 63.5, 44.7, 17.8.

^{19}F NMR (376 MHz, $CDCl_3$) δ -115.67.

HRMS (ESI) calculated for $C_{10}H_{10}FO$ [M+H$^+$]: 165.0710, found: 161.0702.

FT-IR (cm^{-1}): 3540, 2963,2928, 1783, 1602, 1510, 1224, 1098, 1072, 831.

MS (EI) *m/z* (%): 122 (100), 121 (18), 135 12), 108 (12), 96 (12), 164 (4).

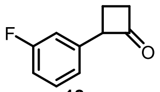

12a

This compound was synthesized according to the procedure above, 67% yield, colorless liquid.

^{1}H NMR (400 MHz, $CDCl_3$) δ 7.33-7.22 (m, 1H), 7.05-6.87 (m, 3H), 4.67-4.40 (m, 1H), 3.34-3.15 (m, 1H), 3.15-2.91 (m, 1H), 2.53 (qd, *J* = 10.8, 5.0 Hz,1H), 2.35-1.98 (m, 1H).

^{13}C NMR (100 MHz, $CDCl_3$) δ 206.7, 162.8 (d, *J* = 244.7 Hz), 138.7 (d, *J* = 7.5 Hz), 130.0 (d, *J* = 8.3 Hz), 122.5 (d, *J* = 2.9 Hz), 113.9 (d, *J* = 11.2 Hz), 113.7 (d, *J* = 10.3 Hz), 63.8 (d, *J* = 1.7 Hz), 44.8, 17.4.

^{19}F NMR (376 MHz, $CDCl_3$) δ -112.83.

HRMS (ESI) calculated for $C_{10}H_{10}FO$ [$M+H^+$]: 165.0710, found: 165.0705.

FT-IR (cm^{-1}): 3073, 2927, 1782, 1588, 1444, 1247, 1147, 787.

MS (EI) *m/z* (%): 122 (100), 121 (15), 96 (13), 135 (8), 164 (5).

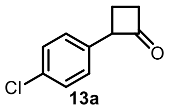

13a

This compound was synthesized according to the procedure above, 77% yield, colorless liquid.

^{1}H NMR (600 MHz, $CDCl_3$) δ 7.32-7.26 (m, 2H), 7.21-7.15 (m, 2H), 4.54-4.44 (m, 1H), 3.29-3.18 (m, 1H), 3.05-2.95 (m, 1H), 2.53 (qd, *J* = 10.5, 5.0 Hz, 1H), 2.25-2.11 (m, 1H).

^{13}C NMR (150 MHz, $CDCl_3$) δ 207.0, 134.8, 132.7, 128.6, 128.2, 63.6, 44.8, 17.6.

HRMS (ESI) calculated for $C_{10}H_{10}ClO$ [$M+H^+$]: 181.0415, found: 181.0414.

FT-IR (cm^{-1}): 3541, 2962, 1784, 1491, 1397, 1199, 1069, 1030, 821.

MS (EI) *m/z* (%): 138 (100), 140 (35), 103 (26), 115 (20), 89 (18), 180 (3), 182 (1).

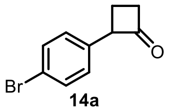

14a

This compound was synthesized according to the procedure above, 63% yield, white solid, m. p.: 37-40 ℃.

^{1}H NMR (400 MHz, $CDCl_3$) δ 7.54-7.36 (m, 2H), 7.23-7.05 (m, 2H), 4.55-4.38 (m, 1H), 3.32-3.15 (m, 1H), 3.08-2.94 (m, 1H), 2.52 (qd, *J* = 10.7, 4.9 Hz, 1H), 2.28-2.08 (m, 1H).

^{13}C NMR (100 MHz, $CDCl_3$) δ 206.8, 135.3, 131.6, 128.6, 120.7, 63.6, 44.8, 17.5.

FT-IR (cm^{-1}): 3539, 2924, 1782, 1485, 1395, 1070, 1011, 816, 727.

MS (EI) *m/z* (%): 184 (100), 182 (95), 103 (53), 115 (48), 89 (35), 77 (28), 63 (23), 51 (13), 224 (3), 226 (3).

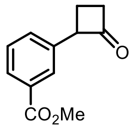

15a

This compound was synthesized according to the procedure above, 67% yield, colorless liquid.

^{1}H NMR (400 MHz, $CDCl_3$) δ 7.94 (d, *J* = 8.0 Hz, 1H), 7.53-7.44 (m, 1H), 7.40-7.28 (m, 2H), 5.43-5.23 (m, 1H), 3.89 (s, 2H), 3.30-3.13 (m, 1H), 3.12-2.94 (m, 1H), 2.58 (qd, *J* = 10.7, 4.4 Hz, 1H), 2.16 (p, *J* = 9.3 Hz, 1H).

^{13}C NMR (100 MHz, $CDCl_3$) δ 207.0, 167.7, 137.6, 132.3, 130.9, 128.9, 128.4, 127.0, 63.1, 52.1, 44.4, 18.8.

HRMS (ESI) calculated for $C_{12}H_{12}NaO_3$ [$M+Na^+$]: 227.0679, found: 226.0678.

FT-IR (cm^{-1}): 2953, 1781, 1720, 1443, 1273, 1199, 1085, 747.

MS (EI) *m/z* (%): 131 (100), 162 (92), 115 (28), 103 (27), 77 (22), 145 (17), 173 8), 204 (2).

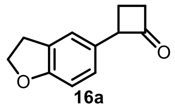

16a

This compound was synthesized according to the procedure above, 80% yield, yellow liquid.

^{1}H NMR (400 MHz, $CDCl_3$) δ 7.08 (s, 1H), 7.01-6.89 (m, 1H), 6.73 (d, *J* = 8.4 Hz, 1H), 4.54 (t, *J* = 8.6 Hz, 1H), 4.50-4.40 (m, 1H), 3.35-3.12 (m, 3H), 3.08-2.92 (m, 1H), 2.51 (qd, *J* = 10.8, 5.1 Hz, 1H), 2.24-2.07 (m, 1H).

^{13}C NMR (100 MHz, $CDCl_3$) δ 208.6, 159.1, 128.5, 127.4, 126.6, 123.6, 109.2, 71.2, 64.2, 44.6, 29.6, 18.2.

HRMS (ESI) calculated for $C_{12}H_{12}NaO_2$ [M+Na$^+$]: 211.0730, found: 211.0729.
FT-IR (cm^{-1}): 2961, 2895, 1779, 1492, 1238, 1071, 982, 942, 817.
MS (EI) *m/z* (%): 146 (100), 51 (35), 91 (32), 117 (32), 77 (25), 115 (25), 131 (23), 159 (22), 188 (5).

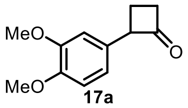

17a

This compound was synthesized according to the procedure above, 72% yield, yellow solid, m. p.: 74-77 ℃.
^{1}H NMR (600 MHz, $CDCl_3$) δ 6.88-6.81 (m, 1H), 6.81-6.71 (m, 2H), 4.54-4.44 (m, 1H), 3.88 (s, 3H), 3.86 (s, 3H), 3.28-3.15 (m, 1H), 3.11-2.95 (m, 1H), 2.53 (qd, J = 10.8, 4.8 Hz, 1H), 2.25-2.14 (m, 1H).
^{13}C NMR (150 MHz, $CDCl_3$) δ 208.1, 149.0, 148.0, 129.1, 118.9, 111.3, 110.3, 64.0, 55.9, 55.8, 44.6, 17.9.
HRMS (ESI) calculated for $C_{12}H_{14}NaO_3$ [M+Na$^+$]: 229.0835, found: 229.0839.
FT-IR (cm^{-1}): 3441, 2959, 1779, 1516, 1253, 1141, 1026.
MS (EI) *m/z* (%): 57 (100), 55 (87), 71 (68), 69 (66), 164 (53), 149 (46), 165 (38), 206 (8).

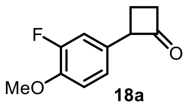

18a

This compound was synthesized according to the procedure above, 76% yield, yellow liquid.
^{1}H NMR (600 MHz, $CDCl_3$) δ 7.05-6.86 (m, 3H), 4.54-4.37 (m, 1H), 3.86 (s, 3H), 3.29-3.15 (m, 1H), 3.10-2.88 (m, 1H), 2.52 (qd, J = 10.8, 4.8 Hz, 1H), 2.23-2.11 (m, 1H).
^{13}C NMR (150 MHz, $CDCl_3$) δ 207.4, 152.3 (d, J = 244.8 Hz), 146.5 (d, J = 11.0 Hz), 129.4 (d, J = 5.9 Hz), 122.6 (d, J = 4.4 Hz), 114.8 (d, J = 19.1 Hz), 113.5 (d, J = 0.9 Hz), 63.4, 56.2, 44.7, 17.8.
^{19}F NMR (376 MHz, $CDCl_3$) δ -134.84.
HRMS (ESI) calculated for $C_{11}H_{12}FO_2$ [M+H$^+$]: 195.0816, found: 195.0813.
FT-IR (cm^{-1}): 2962, 2841, 1781, 1518, 1276, 1128, 1072, 1026, 760.
MS (EI) *m/z* (%): 139 (100), 141 (64), 125 (36), 57 (36), 111 (35), 152 (30), 195 (5).

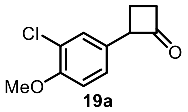

19a

This compound was synthesized according to the procedure above, 76% yield, white solid, m. p.: 57-59 ℃.
^{1}H NMR (600 MHz, $CDCl_3$) δ 7.29-7.21 (m, 1H), 7.17-7.04 (m, 1H), 6.88 (d, J = 8.4 Hz, 1H), 4.54-4.34 (m, 1H), 3.87 (s, 3H), 3.32-3.13 (m, 1H), 3.08-2.89 (m, 1H), 2.52 (qd, J = 10.8, 4.8 Hz, 1H), 2.25-2.08 (m, 1H).
^{13}C NMR (150 MHz, $CDCl_3$) δ 207.4, 153.8, 129.5, 128.6, 126.3, 122.3, 112.0, 63.1, 56.0, 44.7, 17.7.
HRMS (ESI) calculated for $C_{11}H_{12}ClO_2$ [M+H$^+$]: 211.0520, found: 211.0518.
FT-IR (cm^{-1}): 2963, 1779, 1503, 1255, 1065, 1022, 810.
MS (EI) *m/z* (%): 168 (100), 84 (90), 57 (80), 153 (60), 159 (56), 210 (8).

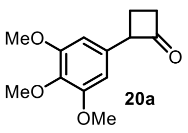

20a

This compound was synthesized according to the procedure above, 65% yield, yellow solid, m. p.: 49-54 ℃.
^{1}H NMR (600 MHz, $CDCl_3$) δ 6.48 (s, 2H), 4.48 (m, 1H), 3.86 (s, 6H), 3.82 (s, 3H), 3.27-3.16 (m, 1H), 3.08-2.98 (m, 1H), 2.54 (qd, J = 10.7, 4.8 Hz, 1H), 2.27-2.14 (m, 1H).
^{13}C NMR (150 MHz, $CDCl_3$) δ 207.6, 153.2, 136.8, 132.0, 103.8, 64.2, 60.6, 55.9, 44.5, 17.8.
HRMS (ESI) calculated for $C_{13}H_{16}NaO_4$ [M+Na$^+$]: 259.0941, found: 259.0933.
FT-IR (cm^{-1}): 2940, 2838, 1780, 1587, 1508, 1463, 1241, 1127, 1007.
MS (EI) *m/z* (%): 194 (100), 236 (80), 179 (78), 193 (62), 177(30), 195 (23), 208 (22), 237 (10).

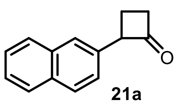

21a

This compound was synthesized according to the procedure above, 74% yield, white solid, m. p.: 82-85 ℃.
^{1}H NMR (400 MHz, $CDCl_3$) δ 7.84-7.75 (m, 3H), 7.69 (s, 1H), 7.51-7.39 (m, 2H),

7.35 (dd, J = 8.6, 0.8 Hz, 1H), 4.83-4.56 (m, 1H), 3.25 (s, 1H), 3.17-2.95 (m, 1H), 2.58 (qd, J = 10.7, 5.0 Hz, 1H), 2.39-2.19 (m, 1H).

^{13}C NMR (100 MHz, $CDCl_3$) δ 207.8, 133.9, 133.3, 132.4, 128.3, 127.7, 127.6, 126.2, 125.7, 125.3, 125.3, 64.6, 44.9, 17.7.

HRMS (ESI) calculated for $C_{14}H_{12}NaO$ [M+Na$^+$]: 219.0780, found: 219.0779.

FT-IR (cm^{-1}): 3429, 2964, 1775, 1598, 1074, 1031, 483.

MS (EI) *m/z* (%): 154 (100), 153 (28), 152 (17), 155 (16), 139 (13), 115 (12), 128 (10), 196 (5).

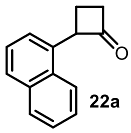

This compound was synthesized according to the procedure above, 62% yield, yellow solid, m. p.: 54-57 °C.

^{1}H NMR (400 MHz, $CDCl_3$) δ 7.83 (d, J = 8.0 Hz, 1H), 7.80-7.74 (m, 1H), 7.67 (d, J = 8.4 Hz, 1H), 7.51-7.39 (m, 3H), 7.33 (t, J = 7.6 Hz, 1H), 4.98 (t, J = 8.6 Hz, 1H), 3.22-3.04 (m, 1H), 3.04-2.86 (m, 1H), 2.49 (m, 1H), 2.31-2.01 (m, 1H).

^{13}C NMR (100 MHz, $CDCl_3$) δ 207.6, 133.7, 132.8, 131.0, 128.4, 127.4, 125.9, 125.6, 125.2, 123.7, 123.0, 61.7, 44.4, 18.2.

HRMS (ESI) calculated for $C_{14}H_{12}NaO$ [M+Na$^+$]: 219.0780, found: 219.0778.

FT-IR (cm^{-1}): 3404, 3047, 2960, 1780, 1396, 1076, 777, 426.

MS (EI) *m/z* (%): 154 (100), 153 (80), 127 (24), 139 (20), 165 (8), 196 (7).

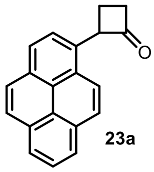

This compound was synthesized according to the procedure above, 57% yield, yellow solid, m. p.: 126-129 °C.

^{1}H NMR (400 MHz, $CDCl_3$) δ 8.20-7.90 (m, 9H), 5.52-5.29 (m, 1H), 3.41-3.26 (m, 1H), 3.22-3.07 (m, 1H), 2.74 (qd, J = 10.7, 5.0 Hz, 1H), 2.49-2.26 (m, 1H).

^{13}C NMR (100 MHz, $CDCl_3$) δ 208.1, 131.2, 130.7, 130.4, 130.4, 128.3, 127.6, 127.3, 127.1, 125.9, 125.2, 125.0, 124.8, 124.6, 123.7, 123.1, 62.3, 44.8, 18.9.

HRMS (ESI) calculated for $C_{20}H_{14}NaO$ [M+Na$^+$]: 293.0937, found: 293.0934.

FT-IR (cm^{-1}): 3442, 3039, 2962, 1776, 1601, 1071, 843, 715.

MS (EI) *m/z* (%): 228 (100), 113 (65), 227 (62), 239 (53), 253 (50), 119 (48), 126(40), 270 (25).

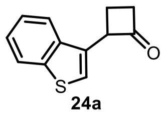

This compound was synthesized according to the procedure above, 26% yield, brown solid, m. p.: 53-55 °C.

^{1}H NMR (600 MHz, $CDCl_3$) δ 7.85 (d, J = 7.8 Hz, 1H), 7.74 (d, J = 7.8 Hz, 1H), 7.42-7.32 (m, 3H), 4.84-4.78 (m, 1H), 3.35-3.26 (m, 1H), 3.19-3.10 (m, 1H), 2.66 (qd, J = 10.7, 5.0 Hz, 1H), 2.29-2.21 (m, 1H).

^{13}C NMR (150 MHz, $CDCl_3$) δ 206.8, 140.4, 137.9, 131.3, 124.6, 124.1, 122.9, 122.0, 121.7, 59.1, 45.2, 17.6.

HRMS (ESI) calculated for $C_{12}H_{11}OS$ [M+H$^+$]: 203.0525, found: 203.0526.

FT-IR (cm^{-1}) 3069, 2962, 1780, 1725, 1428, 1258, 1071, 762, 732, 421.

MS (EI) *m/z* (%): 161 (100), 115 (62), 139 (56), 173 (52), 147 (48), 202 (20).

6. [2+2] Synthesis of Heteroaromatic substituted Cyclobutanone[4]

The substrate **25a-27a** was synthesized according to the literature

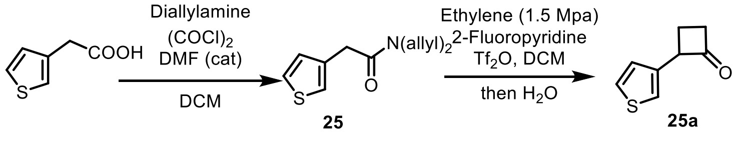

To a solution of 3-thiopheneacetic acid (2.84 g, 20 mmol) in DCM at 0 °C was added oxalyl chloride (2.0 ml, 24 mmol, 1.2 equiv) dropwise carefully. And the reaction mixture was stirred for 30 min at room temperature. The mixture was concentrated in vacuum, dissolved in DCM again and cooled to 0 °C. Diallyamine (7.4 mL, 60 mmol, 3.0 equiv) was added dropwise and stirred until full conversion monitored by TLC. The solution was diluted with a saturated solution of aqueous Na_2CO_3 (15 mL) and extracted with DCM three times. The combined organic layers were washed with brine, dried (Na_2SO_4) and concentrated in vacuum. The crude product can be used in the next step without further purification.

To a solution of the crude product (2.21 g, 10 mmol) in DCM (0.2 M, 50 mL) at 0 °C was added 2-Fluoropyridine (1.12 mL, 13.0 mmol, 1.3 equiv), Tf_2O (2.02 mL, 12.0 mmol, 1.2 equiv). And then the reaction mixture stirred under ethyleme (1.5 MPa) at 60 ℃ for 30 min, then the solution was diluted with H_2O (20 mL), and stirred overnight. The organic layers were separated, and the aqueous layer was extracted with DCM. The combined organic layers were washed with saturated aqueous $NaHCO_3$ and brine, dried (Na_2SO_4), and concentrated in vacuo. The residue was purified by column chromatography on silica gel on silica gel to afford cyclobutanone **25a**.

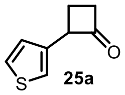

25a

This compound was synthesized according to the procedure above, 31% yield, red liquid.

^{1}H NMR (600 MHz, $CDCl_3$) δ 7.29 (dd, *J* = 4.8, 3.0 Hz, 1H), 7.10 (d, *J* = 3.0 Hz, 1H), 6.99 (dd, *J* = 4.8, 1.2 Hz, 1H), 4.58-4.49 (m, 1H), 3.26-3.15 (m, 1H), 3.10-2.99 (m, 1H), 2.52 (qd, *J* = 10.7, 5.2 Hz, 1H), 2.20-2.10 (m, 1H).

^{13}C NMR (150 MHz, $CDCl_3$) δ 207.2, 136.8, 126.4, 126.0, 120.6, 60.2, 44.9, 18.2.

HRMS (ESI) calculated for C_8H_9OS [M+H$^+$]: 153.0369, found: 153.0374.

FT-IR (cm^{-1}) 3103, 2961, 1779, 1259, 1072, 845, 784, 752, 628.

MS (EI) *m/z* (%): 111 (100), 139 (50), 124 (10), 152 (10).

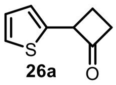

26a

This compound was synthesized according to the procedure above, 15% yield, red liquid.

^{1}H NMR (600 MHz, $CDCl_3$) δ 7.21 (dd, *J* = 5.1, 0.9 Hz, 1H), 6.97 (dd, *J* = 5.4, 3.6 Hz, 1H), 6.91 (d, *J* = 3.6 Hz, 1H), 4.71-4.66 (m, 1H), 3.29-3.19 (m, 1H), 3.14-3.05 (m, 1H), 2.61 (qd, *J* = 10.8, 4.8 Hz, 1H), 2.25-2.16 (m, 1H).

^{13}C NMR (150 MHz, $CDCl_3$) δ 205.7, 138.5, 127.0, 124.3, 124.2, 59.7, 44.9, 19.7.

HRMS (ESI) calculated for C_8H_9OS [M+H$^+$]: 153.0369, found: 153.0370.

FT-IR (cm^{-1}) 3105, 2962, 2926, 1783, 1727, 1665, 1415, 1258, 1068, 701.

MS (EI) *m/z* (%): 111 (100), 139 (50), 124 (10), 152 (10).

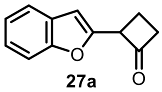

27a

This compound was synthesized according to the procedure above, 27.3% yield, brown solid, m. p.: 34-36 ℃.

^{1}H NMR (600 MHz, $CDCl_3$) δ 7.56 (d, *J* = 1.2 Hz, 1H), 7.53 (dd, *J* = 7.8, 1.2 Hz, 1H), 7.47 (d, *J* = 8.4 Hz, 1H), 7.30 (td, *J* = 7.5, 1.2 Hz, 1H), 7.24 (td, *J* = 7.5, 1.0 Hz, 1H), 4.68-4.58 (m, 1H), 3.36-3.25 (m, 1H), 3.18-3.10 (m, 1H), 2.59 (qd, *J* = 10.8, 4.8 Hz, 1H), 2.24-2.10 (m, 1H).

^{13}C NMR (150 MHz, $CDCl_3$) δ 206.6, 155.4, 141.1, 126.6, 124.6, 122.6, 119.9, 116.2, 111.6, 55.1, 45.5, 17.3.

HRMS (ESI) calculated for $C_{12}H_{11}O_2$ [M+H$^+$]: 187.0754, found: 187.0749.

FT-IR (cm^{-1}) 2962, 2929, 1788, 1724, 1599, 1453, 1255, 803, 750.

MS (EI) *m/z* (%): 145 (100), 144 (87), 115 (40), 131 (15), 157 (12), 102 (10), 186 (7).

7. Synthesis of 2,3-disubstituted Cyclobutanone[5]

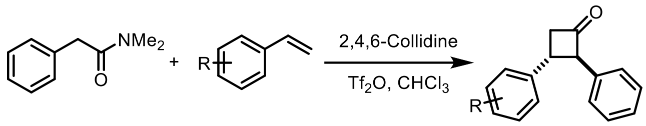

28a-31a

The substrate **28a-31a** was synthesized according to the literature.[5]

General procedure for the synthesis of 2,3-diphenyl Cyclobutanone 28a

To a refluxing mixture of *N*, *N*-Dimethyl-2-phenyl-acetamide (1.63 g, 10 mmol, 1.0 equiv), 2,4,6-Collidine (1.6 mL, 12 mmol, 1.2 equiv) and styrene (5.8 ml, 50 mmol, 5.0 equiv) in $CHCl_3$ 30 mL under argon atmosphere was added the $CHCl_3$ solution of Tf_2O (2,0 mL, 12 mmol, 1.2 equiv) dropwise over 12 h by syringe pump. After full conversion, the reaction mixture was cooled to room temperature, then diluted with CH_2Cl_2 and water. The biphasic mixture was stirred for 30 min, then separated and the aqueous layer extracted with CH_2Cl_2, combined organic layers were washed with brine, dried by Na_2SO_4, concentrated in vacuum. The crude products were purified directly by column chromatography on silica gel with a mixture of petroleum ether and ethyl acetate to give cyclobutanone **28a**.

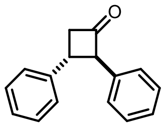

28a

This compound was synthesized according to the procedure above, 67% yield, white solid, m.p.: 49-51 °C.

^{1}H NMR (400 MHz, $CDCl_3$) δ 7.42-7.19 (m, 10H), 4.55 (dt, *J* = 8.8, 1.6 Hz, 1H), 3.78 (q, *J* = 8.9 Hz, 1H), 3.47-3.24 (m, 2H).

^{13}C NMR (100 MHz, $CDCl_3$) δ 204.9, 142.4, 135.6, 128.7, 128.7, 127.2, 127.0, 126.8, 126.5, 71.3, 51.3, 36.7.

HRMS (ESI) calculated for $C_{16}H_{14}NaO$ [$M+Na^+$]: 245.0937, found: 245.0932.

FT-IR (cm^{-1}): 3451, 2923, 1778, 1452, 1074, 6950.

MS (EI) *m/z* (%): 134 (100), 148 (47), 118 (34), 91 (25), 77 (22), 180 (14), 165 (5), 223 (1).

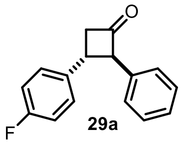

29a

This compound was synthesized according to the procedure above, 69% yield, colorless liquid.

^{1}H NMR (400 MHz, $CDCl_3$) δ 7.41-7.19 (m, 7H), 7.11-6.99 (m, 2H), 4.52 (dt, *J* = 8.9, 2.1 Hz, 1H), 3.77 (q, *J* = 8.8 Hz, 1H), 3.48-3.23 (m, 2H).

^{13}C NMR (100 MHz, $CDCl_3$) δ 204.5, 161.7 (d, *J* = 244 Hz), 138.1 (d, *J* = 3.2 Hz), 135.4, 128.7, 128.1 (d, *J* = 7.9 Hz), 127.3, 127.0, 115.6 (d, *J* = 21.4 Hz), 71.6, 51.4, 36.2.

^{19}F NMR (376 MHz, $CDCl_3$) δ -115.61.

HRMS (ESI) calculated for $C_{16}H_{14}FO$ [$M+H^+$]: 241.1023, found: 241.1030.

FT-IR (cm^{-1}): 3029, 1781, 1510, 1225, 1174, 831, 697, 525.

MS (EI) *m/z* (%): 198 (100), 118 (96), 90 (48), 183 (22), 133 (10), 240 (1).

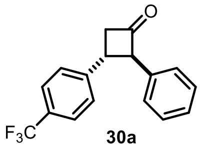

30a

This compound was synthesized according to the procedure above, 69% yield, yellow solid, m.p.: 35-37 °C.

^{1}H NMR (400 MHz, $CDCl_3$) δ 7.62 (d, *J* = 8.0 Hz, 2H), 7.47 (d, *J* = 8.8 Hz, 2H), 7.40-7.31 (m, 2H), 7.31-7.24 (m, 3H), 4.58 (d, *J* = 8.8 Hz, 1H), 3.86 (q, *J* = 8.9 Hz, 1H), 3.42 (m, 2H).

^{13}C NMR (100 MHz, $CDCl_3$) δ 203.9, 146.4 (d, *J* = 1.1 Hz), 135.2, 129.2 (q, *J* = 32.3 Hz), 128.8, 127.5, 127.0, 127.0, 125.7 (q, *J* = 3.7 Hz), 71.6, 51.2, 36.8.

^{19}F NMR (376 MHz, $CDCl_3$) δ -62.39.

HRMS (ESI) calculated for $C_{17}H_{14}F_3O$ [M+H$^+$]: 291.0991, found: 291.0992.
FT-IR (cm^{-1}): 3030, 1784, 1619, 1326, 1165, 1122, 1068, 835, 697.
MS (EI) *m/z* (%): 248 (100), 118 (68), 179 (66), 89 (54), 90 (50), 262 (25), 290 (5).

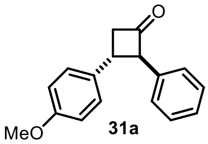
31a

This compound was synthesized according to the procedure above, 39% yield, yellow liquid.
^{1}H NMR (600 MHz, $CDCl_3$) δ 7.37-7.31 (m, 2H), 7.29-7.24 (m, 5H), 6.98-6.85 (m, 2H), 4.53 (dt, *J* = 9.0, 2.1 Hz, 1H), 3.80 (s, 3H), 3.78-3.69 (m, 1H), 3.43-3.26 (m, 2H).
^{13}C NMR (150 MHz, $CDCl_3$) δ 205.4, 158.5, 135.7, 134.5, 128.7, 127.6, 127.2, 127.0, 114.1, 71.5, 55.3, 51.5, 36.1.
HRMS (ESI) calculated for $C_{17}H_{17}O$ [M+H$^+$]: 253.1223, found: 253.1223.
FT-IR (cm^{-1}): 3084, 2933, 2835, 1777, 1513, 1249, 1179, 1033, 829, 698.
MS (EI) *m/z* (%): 134 (100), 105 (50), 136 (24), 119 (10), 179 (10), 210 (5), 252 (1).

8. Characterization of the products and unreacted Cyclobutanone

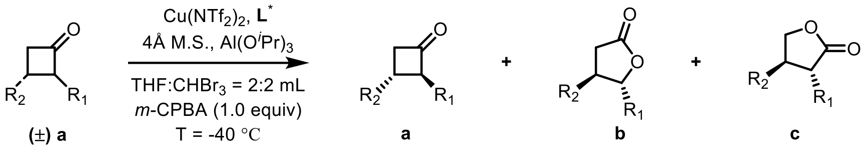

General procedure: the mixture of anhydrous $Cu(NTf_2)_2$ (12.5 mg, 0.02 mmol), **L1** (6.4 mg, 0.024 mmol) was stirred in THF (1 mL) for 1 h at room temperature (r. t.). The 4Å molecular sieves (M.S., 60 mg), Al(O^{i}Pr)$_3$ (20.4 mg, 50% mol) and **1a** was added to an oven-dried reaction tube in batches, then the chiral catalyst solution prepared beforehand, THF (1 mL) and $CHBr_3$ (2 mL) were added, the reaction mixture was cooled to -40 °C for 20 min. Then, the *m*-CPBA (0.2 mmol) was added to the reaction mixture at -40 °C. The Na_2SO_3 (126 mg, 1.0 mmol) was added after the reaction mixture had been stirred for the time indicated at -40 °C, and stirred for additional 2 h at -40 °C. The DCM (10 mL) was added, the solution was extracted with DCM. The combined organic layers were washed with brine, dried ($NaSO_4$), concentrated and the crude product was purified by flash column chromatography on silica gel at **-15 °C** to afford the desired lactones **b/c** and recover the unreacted ketones **a**.
Indicated procedure a: The reaction was performed at 20 °C.
Indicated procedure b: The THF/Et_2O/$CHBr_3$ (1.0/1.0/2.0 mL) was used.
Indicated procedure c: 0.6 equiv of *m*-CPBA was used.
Indicated procedure d: $Cu(OTf)_2$ and ***ent*-L1** was used.

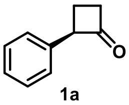
1a

(-)-2-phenylcyclobutan -1-one (1a):
Prepared according to **general procedure**: 36 h, 43% yield, 91.2% ee
[α]$_D^{22}$ = -15.0 (c = 1.0, $CHCl_3$).
The ee value was determined by the chiral UPC^2 analysis (CHIRALPAK® IG-3, CO_2/MeOH = 95/05, v = 2.0 mL/min, λ = 205.0 nm, t (major) = 1.041 min, t (minor) = 1.190 min.

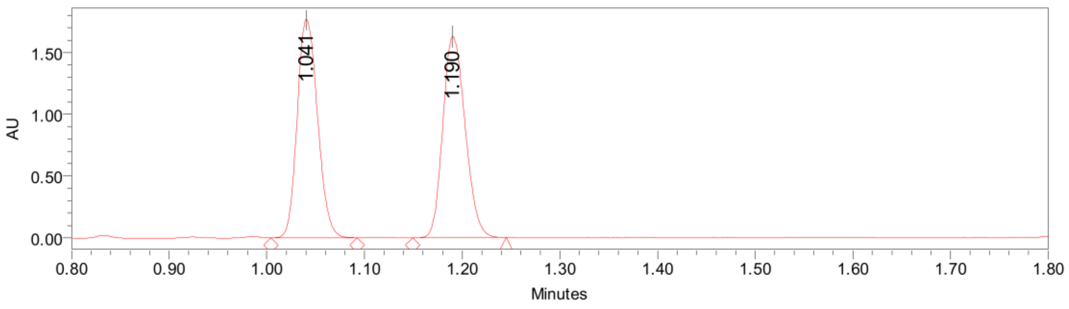

	RT	Area	% Area	Height
1	1.041	2573419	49.75	1773586
2	1.190	2599177	50.25	1631409

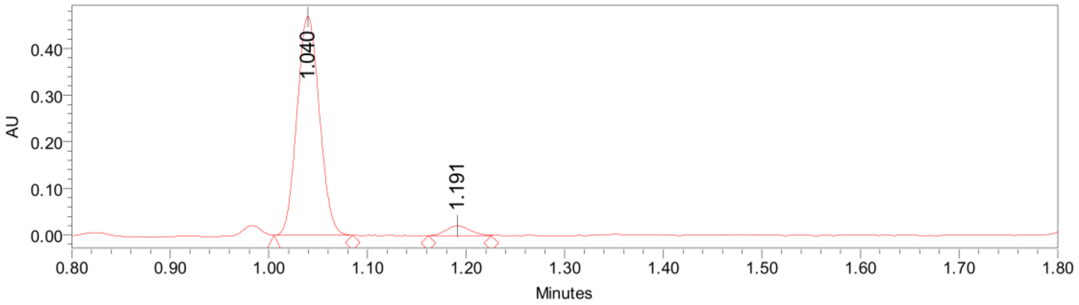

	RT	Area	% Area	Height
1	1.040	743230	95.61	470195
2	1.191	34104	4.39	20535

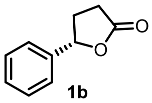

1b

(-)-5-phenyldihydrofuran-2(3H)-one (1b):

Prepared according to **general procedure**: 36 h, 52% yield, 92.6% ee, **1b/1c** = 12.5/1, colorless liquid.

$[\alpha]_D^{23}$ = -27.0 (c = 1.0, $CHCl_3$).

^{1}H NMR (400 MHz, $CDCl_3$) δ 7.42-7.30 (m, 5H), 5.53-5.46 (m, 1H), 2.70-2.60 (m, 3H), 2.23-2.11 (m, 1H).

^{13}C NMR (100 MHz, $CDCl_3$) δ 176.8, 139.3, 128.6, 128.3, 125.2, 81.1, 30.8, 28.8.

HRMS (ESI) calculated for $C_{10}H_{10}NaO_2$ $[M+Na^+]$: 185.0573, found: 185.0570.

FT-IR (cm^{-1}): 2926, 1173, 1216, 1176, 1141, 1025, 940, 699.

MS (EI) *m/z* (%): 84 (100), 56 (74), 51 (68), 107 (66), 117 (65), 105 (56), 162 (42).

The ee value was determined by the chiral UPC^2 analysis (CHIRALPAK® IC-3, CO_2/MeOH = 95/05, v = 2.0 mL/min, λ = 205.0 nm), t_b (minor) = 2.304 min, t_b (major) = 2.476 min, t_c (minor) = 3.102 min, t_c (major) = 3.240 min.

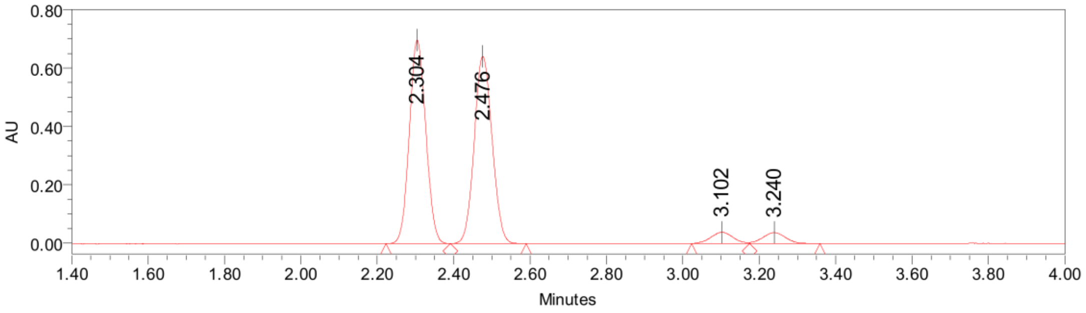

	RT	Area	% Area	Height
1	2.304	2085191	46.59	697184
2	2.476	2090832	46.71	641540
3	3.102	149444	3.34	38233
4	3.240	150501	3.36	36944

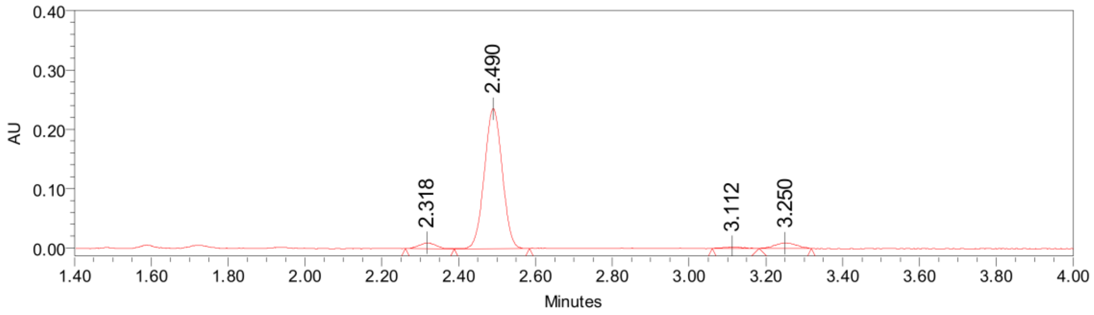

	RT	Area	% Area	Height
1	2.318	29242	3.50	9795
2	2.490	764873	91.46	236179
3	3.112	7537	0.90	2429
4	3.250	34597	4.14	9074

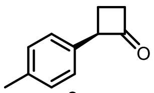

2a

(-)-2-(p-tolyl)cyclobutan-1-one (2a)

Prepared according to **general procedure**: 48 h, 43% yield, 98.4% ee.

$[\alpha]_D^{22}$ = -15.0 (c = 1.0, $CHCl_3$).

The ee value was determined by the chiral UPC[2] analysis (CHIRALPAK® IG-3, CO_2/MeOH = 95/05, v = 2.0 mL/min, λ = 205.0 nm, t (major) = 1.213 min, t (minor) = 1.402 min.

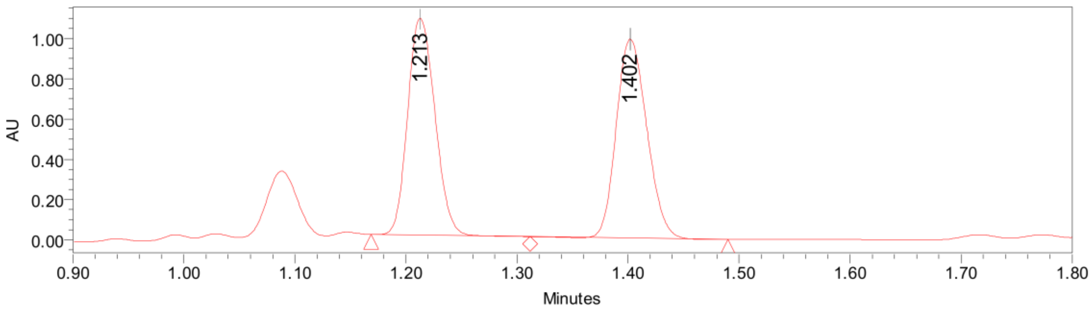

	RT	Area	% Area	Height
1	1.213	1845000	49.41	1076762
2	1.402	1889430	50.59	986704

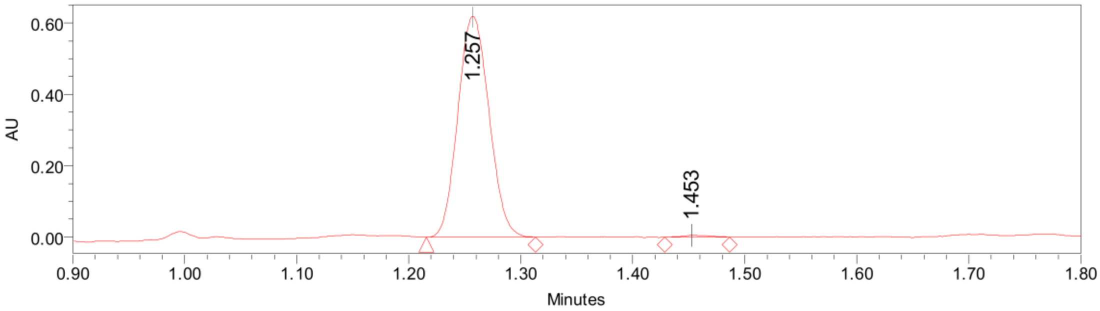

	RT	Area	% Area	Height
1	1.257	1199679	99.19	620090
2	1.453	9815	0.81	5446

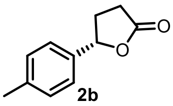
2b

(-)-5-(p-tolyl)dihydrofuran-2(3H)-one (2b)

Prepared according to **general procedure**: 48 h, 57% yield, 89.8% ee, **2b**/**2c** = 10.0/1, colorless liquid.

[α]$_D$^{25} = -10.0 (c = 1.0, $CHCl_3$).

^{1}H NMR (600 MHz, $CDCl_3$) δ 7.21 (q, *J* = 8.6 Hz, 4H), 5.51-5.44 (m, 1H), 2.67-2.61 (m, 3H), 2.36 (s, 3H), 2.21-2.15 (m, 1H).

^{13}C NMR (150 MHz, $CDCl_3$) δ 176.9, 138.3, 136.3, 129.4, 125.3, 81.3, 30.9, 29.0, 21.1.

HRMS (ESI) calculated for $C_{11}H_{12}NaO_2$ [M+Na$^+$]: 199.0730, found: 199.0723.

FT-IR (cm^{-1}): 3407, 2923, 1772, 1175, 1141, 1025, 938, 803.

MS (EI) *m/z* (%): 121 (100), 176 (69), 119 (54), 117 (52), 161 (34), 132 (20).

The ee value was determined by the chiral UPC^2 analysis (CHIRALPAK® IG-3, CO_2/MeOH = 95/05, v = 2.0 mL/min, λ = 205.0 nm), t_b (major) = 3.438 min, t_b (minor) = 4.193 min, t_c = 3.673 min.

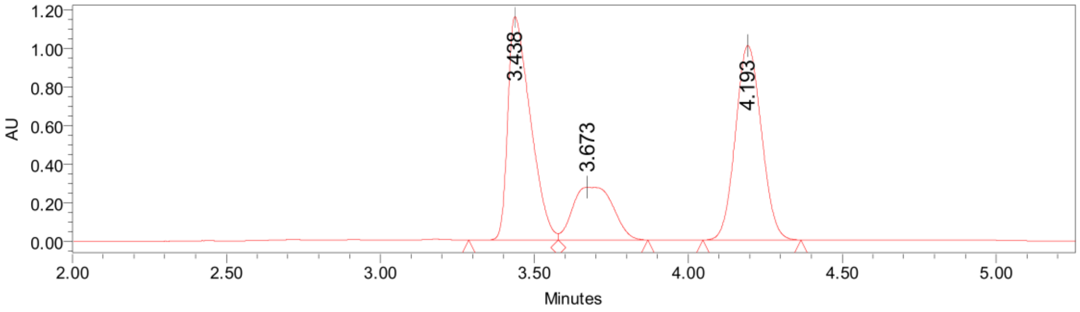

	RT	Area	% Area	Height
1	3.438	5907715	41.28	1157066
2	3.673	2492912	17.42	272842
3	4.193	5911667	41.30	1007212

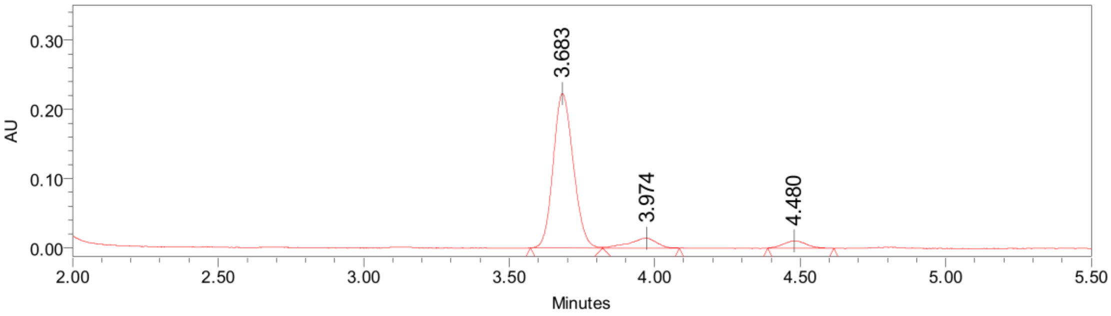

	RT	Area	% Area	Height
1	3.683	1068463	87.50	222938
2	3.974	95045	7.78	14253
3	4.480	57646	4.72	10360

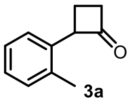
3a

(-)-2-(o-tolyl)cyclobutan-1-one (3a)

Prepared according to **indicated procedure a**: 72 h, 52% yield, 7.3% ee, colorless liquid.

[α]$_D$^{22} = -3.0 (c = 1.0, $CHCl_3$).

The ee value was determined by the chiral UPC[2] analysis (CHIRALPAK® IG-3, CO_2/MeOH = 95/05, v = 2.0 mL/min, λ = 205.0 nm, t (major) = 0.989min, t (minor) = 1.186 min).

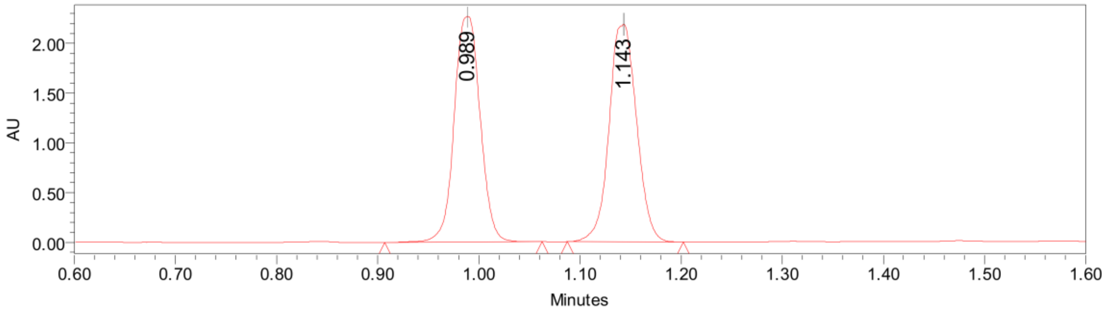

	RT	Area	% Area	Height
1	0.989	4043020	49.45	2269209
2	1.143	4133217	50.55	2188244

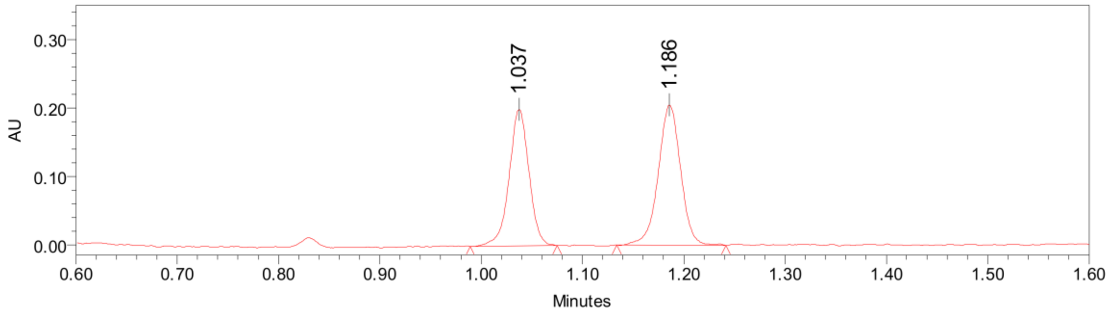

	RT	Area	% Area	Height
1	1.037	271554	46.33	199282
2	1.186	314581	53.67	204793

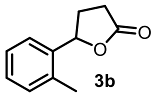
3b

(-)-5-(o-tolyl)dihydrofuran-2(3H)-one (3b)

Prepared according to **indicated procedure a**: 72 h, 44% yield, 61.5% ee (**3c**: 44.8% ee), **3b**/**3c** = 1.5/1, colorless liquid.

$[\alpha]_D^{22}$ = -8.0 (c = 1.0, $CHCl_3$).

^{1}H NMR (600 MHz, $CDCl_3$) δ 7.39-7.32 (m, 1H), 7.26-7.23 (m, 2H), 7.22-7.15 (m, 1H), 5.76-5.67 (m, 1H), 2.73-2.63 (m, 3H), 2.34 (s, 3H), 2.17-2.09 (m, 1H).

^{13}C NMR (150 MHz, $CDCl_3$) δ 177.1, 137.5, 134.1, 130.7, 128.1, 126.4, 124.1, 78.9, 29.5, 28.7, 19.0.

HRMS (ESI) calculated for $C_{11}H_{12}O_2Na$ $[M+Na^+]$: 199.0730, found: 199.0731.

FT-IR (cm^{-1}): 2923, 1772, 1175, 1143, 1023, 937, 801, 717.

MS (EI) *m/z* (%): 176 (100), 117 (87), 121 (86%), 119 (80), 131 (60), 132 (18), 120 (18).

The ee value was determined by the chiral UPC[2] analysis (CHIRALPAK® IC-3, CO_2/MeOH = 95/05, v = 2.0 mL/min, λ = 205.0 nm), t_b (minor) = 2.462 min, t_b (major) = 2.782 min, t_c (major) = 3.089 min t_c (minor) = 3.349 min.

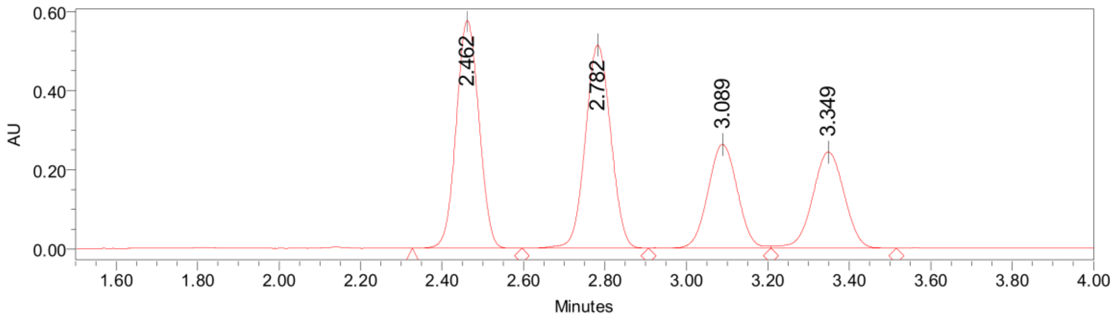

	RT	Area	% Area	Height
1	2.462	2226644	31.47	574956
2	2.782	2224117	31.44	513060
3	3.089	1311916	18.54	262297
4	3.349	1311711	18.54	243108

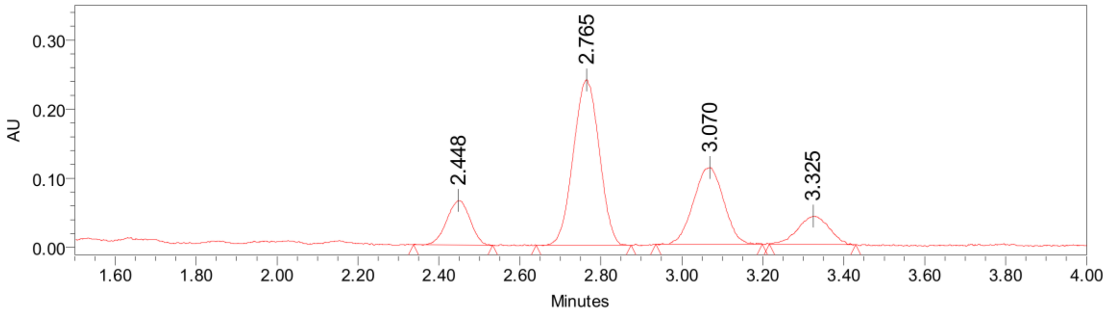

	RT	Area	% Area	Height
1	2.448	254292	12.07	64166
2	2.765	1065289	50.55	239390
3	3.070	570449	27.07	110259
4	3.325	217397	10.32	40716

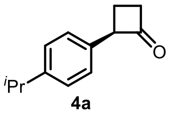

4a

(-)-2-(4-isopropylphenyl)cyclobutan-1-one (4a)

Prepared according to **indicated procedure b**: 50 h, 44% yield, 90.2% ee.

[α]$_D$^{24} = -25.0 (c = 1.0, $CHCl_3$).

The ee value was determined by the chiral UPC^2 analysis (CHIRALPAK® IG-3, CO_2/MeOH = 98/02, v = 2.0 mL/min, λ = 205.0 nm), t (major) = 1.819 min, t (minor) = 2.434 min.

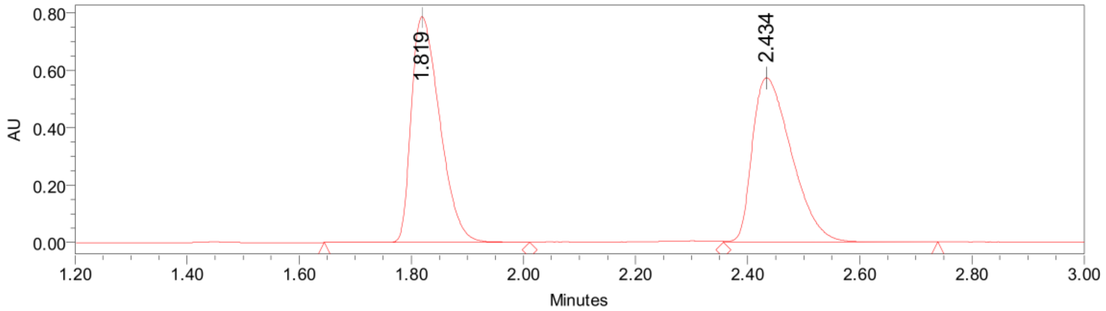

	RT	Area	% Area	Height
1	1.819	2774264	49.96	787186
2	2.434	2779124	50.04	572559

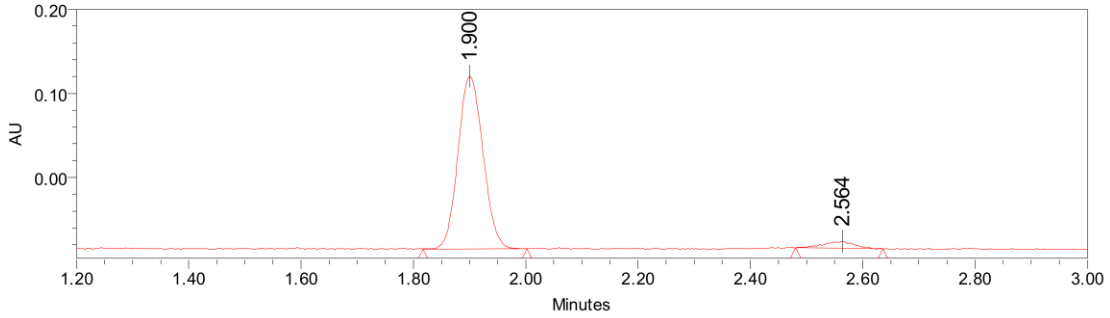

	RT	Area	% Area	Height
1	1.900	634363	95.12	205209
2	2.564	32531	4.88	8219

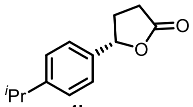

4b

(-)-5-(4-isopropylphenyl)dihydrofuran-2(3H)-one (4b)

Prepared according to **indicated procedure b**: 50 h, 52% yield, 90.6% ee, **4b**/**4c** = 9.2/1, white solid, m. p.: 39-44 ℃.

$[\alpha]_D^{24}$ = -11.0 (c = 1.0, $CHCl_3$).

^{1}H NMR (400 MHz, $CDCl_3$) δ 7.29-7.24 (m, 4H), 5.53-5.43 (m, 1H), 2.91 (hept, *J* = 6.9 Hz, 1H), 2.69-2.59 (m, 3H), 2.26-2.16 (m, 1H), 1.25 (s, 3H), 1.24 (s, 3H).

^{13}C NMR (100 MHz, $CDCl_3$) δ 176.9, 149.2, 136.6, 126.7, 125.4, 81.3, 33.8, 30.8, 29.0, 23.9.

HRMS (ESI) calculated for $C_{13}H_{17}O_2$ $[M+H^+]$: 225.1223, found: 225.1222.

FT-IR (cm^{-1}): 2960, 1773, 1176, 1142, 1018, 938, 808.

MS (EI) *m/z* (%): 149 (100), 161 (96), 105 (60), 91 (60), 201 (58), 145 (50), 119 (44), 189 (42).

The ee value was determined by the chiral UPC^2 analysis (CHIRALPAK® IG-3, CO_2/MeOH = 95/05, v = 2.0 mL/min, λ = 216.7 nm), t_b (major) = 4.185 min, t_b (minor) = 4.988 min, t_c (minor) = 3.348 min, t_c (major) = 3.436 min.

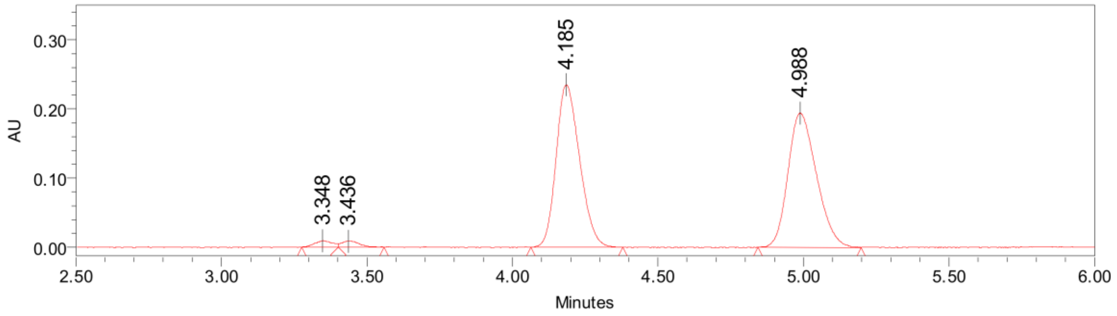

	RT	Area	% Area	Height
1	3.348	41522	1.52	9448
2	3.436	38381	1.41	8922
3	4.185	1324877	48.57	235008
4	4.988	1322940	48.50	194411

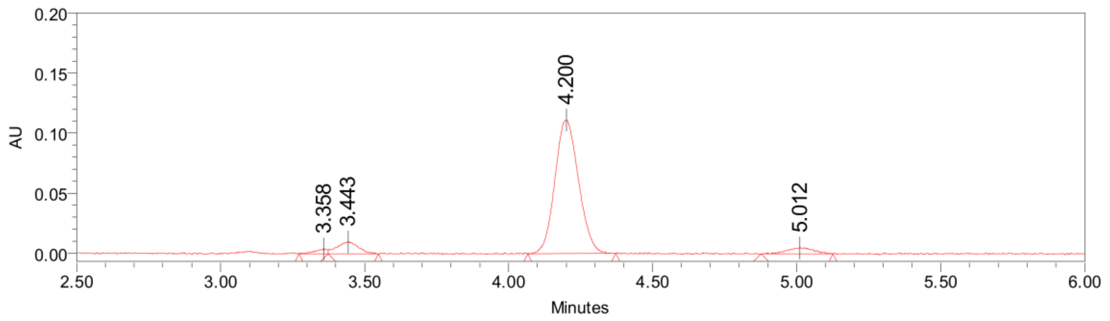

	RT	Area	% Area	Height
1	3.358	10534	1.44	3911
2	3.443	51056	6.99	9797
3	4.200	637729	87.27	111679
4	5.012	31447	4.30	4789

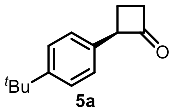

5a

(-)-2-(4-(tert-butyl)phenyl)cyclobutan-1-one (5a)

Prepared according to **indicated procedure b**: 50 h, 53% yield, 78.3% ee.

$[\alpha]_D^{26}$ = -28.0 (c = 1.0, $CHCl_3$).

The ee value was determined by the chiral UPC^2 analysis (CHIRALPAK® IC-3, CO_2/MeOH = 98/02, v = 2.0 mL/min, λ = 219.1 nm), t (major) = 2.897 min, t (minor) = 3.729 min.

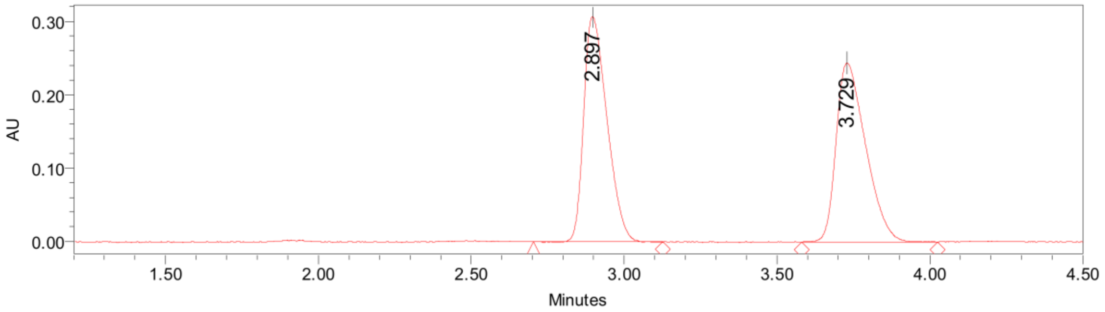

	RT	Area	% Area	Height
1	2.897	1622749	49.77	307125
2	3.729	1638036	50.23	244106

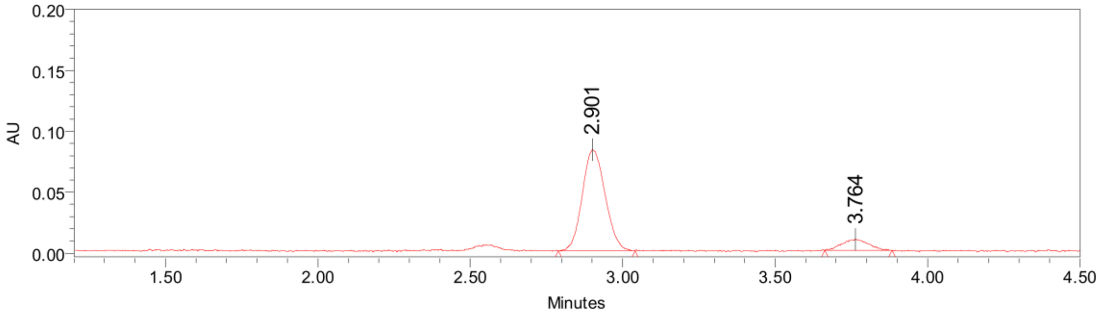

	RT	Area	% Area	Height
1	2.901	433013	89.13	82995
2	3.764	52815	10.87	8898

(-)-5-(4-(tert-butyl)phenyl)dihydrofuran-2(3H)-one (5b)

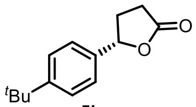

5b

Prepared according to **indicated procedure b**: 50 h, 43% yield, 92.2% ee, **5b**/**5c** = 10.0/1, colorless liquid.

[α]$_D^{26}$ = -15.0 (c = 1.0, $CHCl_3$)

^{1}H NMR (600 MHz, $CDCl_3$) δ 7.41 (d, *J* = 7.8 Hz, 2H), 7.27 (d, *J* = 8.4 Hz, 2H), 5.52-5.48 (m, 1H), 2.68-2.61 (m, 3H), 2.26-2.17 (m, 1H), 1.32 (s, 9H).

^{13}C NMR (150 MHz, $CDCl_3$) δ 177.0, 151.6, 136.2, 125.6, 125.1, 81.2, 34.6, 31.3, 30.8, 29.0.

HRMS (ESI) calculated for $C_{14}H_{18}NaO_2$ [M+Na$^+$]: 241.1199, found: 241.1191.

FT-IR (cm^{-1}): 3396, 2961, 1767, 1410, 1170, 1141, 1031, 942.

MS (EI) *m/z* (%): 203 (100), 218 (20), 85 (28), 91 (18), 57 (13), 115 (11), 175 (10), 159 (10),128 (10).

The ee value was determined by the chiral UPC^2 analysis (CHIRALPAK® AD-3, CO_2/MeOH = 95/05, v = 2.0 mL/min, λ = 216.7 nm), t_b (major) = 2.455 min, t_b (minor) = 2.851 min, t_c (major) = 1.854 min, t_c (minor) = 2.052 min.

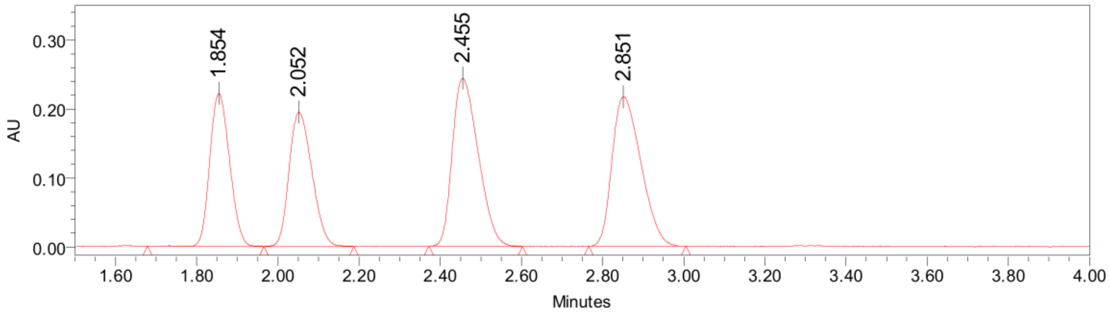

	RT	Area	% Area	Height
1	1.854	752966	20.53	221618
2	2.052	760230	20.73	194598
3	2.455	1078612	29.41	243406
4	2.851	1075936	29.34	216800

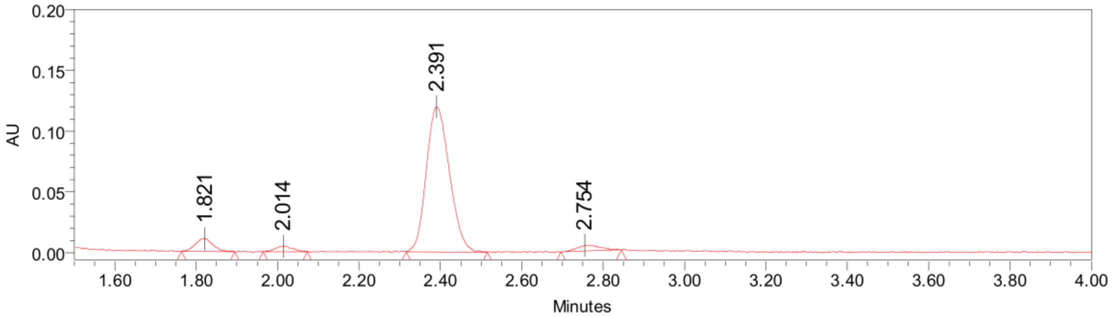

	RT	Area	% Area	Height
1	1.821	31785	5.95	10600
2	2.014	12790	2.40	4392
3	2.391	470095	88.06	119258
4	2.754	19167	3.59	4666

(-)-2-([1,1'-biphenyl]-4-yl)cyclobutan-1-one (6a)

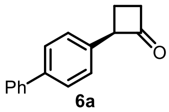

6a

Prepared according to **general procedure**: 42 h, 45% yield, 90.2% ee.

[α]$_D^{26}$ = -30.0 (c = 1.0, $CHCl_3$)

The ee value was determined by the chiral UPC^2 analysis (CHIRALPAK® AD-3, CO_2/MeOH = 95/05, v = 2.0 mL/min, λ = 251.0 nm), t (major) = 4.596 min, t (minor) = 6.211 min.

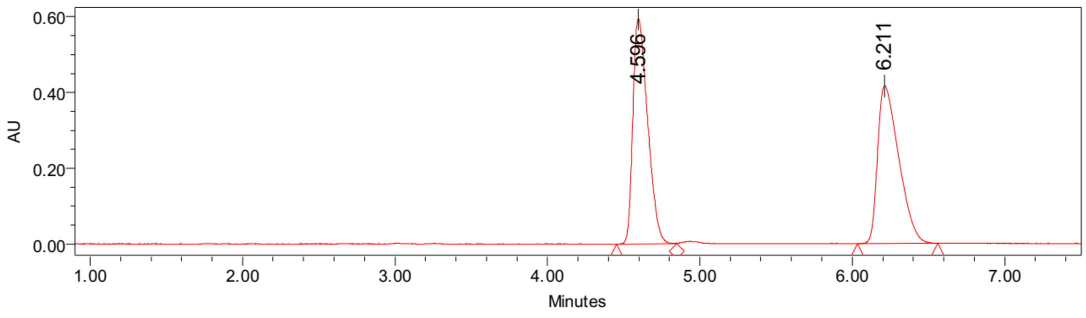

	RT	Area	% Area	Height
1	4.596	4011514	49.95	594170
2	6.211	4019661	50.05	416074

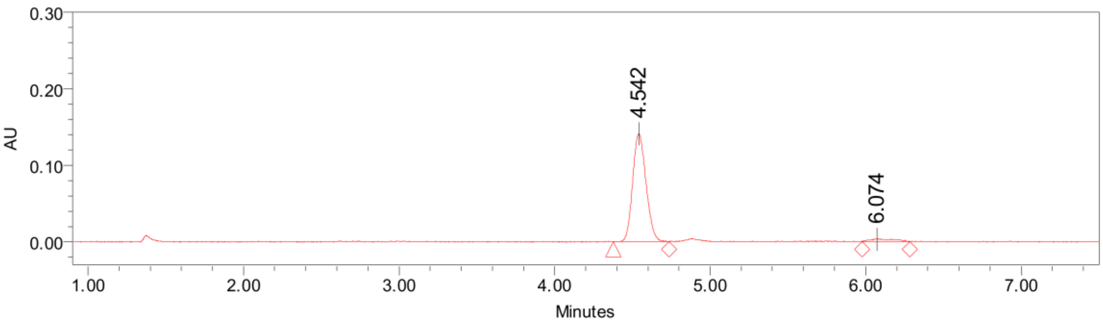

	RT	Area	% Area	Height
1	4.542	833524	95.13	140835
2	6.074	42635	4.87	3505

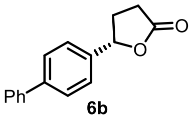

6b

(*S*)-5-([1,1'-biphenyl]-4-yl)dihydrofuran-2(3H)-one (6b)

Prepared according to **general procedure**: 42 h, 54% yield, 91.1% ee, **6b**/**6c** = =12.5/1, white solid, m. p.: 105-107 °C.

$[\alpha]_D^{30}$ = -24.0 (c = 1.0, $CHCl_3$)

^{1}H NMR (400 MHz, $CDCl_3$) δ 7.65-7.55 (m, 4H), 7.49-7.33 (m, 5H), 5.59-5.51 (m, 1H), 2.74-2.63 (m, 3H), 2.31-2.15 (m, 1H).

^{13}C NMR (100 MHz, $CDCl_3$) δ 176.9, 141.4, 140.3, 138.2, 128.8, 127.5, 127.4, 127.0, 125.7, 81.0, 30.9, 28.9.

HRMS (ESI) calculated for $C_{16}H_{14}NaO_2$ [M+Na$^+$]: 261.0886, found: 261.0880.

FT-IR (cm^{-1}): 2921, 1698, 1179, 1141, 1031, 760, 688.

MS (EI) *m/z* (%): 238 (100), 183 (60), 152(48), 194 (26), 115 (18), 165 (14), 127 (8), 139 (5), 209 (3).

The ee value was determined by the chiral UPC^2 analysis (CHIRALPAK® OD-3, CO_2/MeOH = 90/10, v = 2.0 mL/min, λ = 205.0 nm), t_b (minor) =2.398 min, t_b (major) = 2.620 min, t_c (major) = 2.774 min, t_c (minor) = 3.294 min

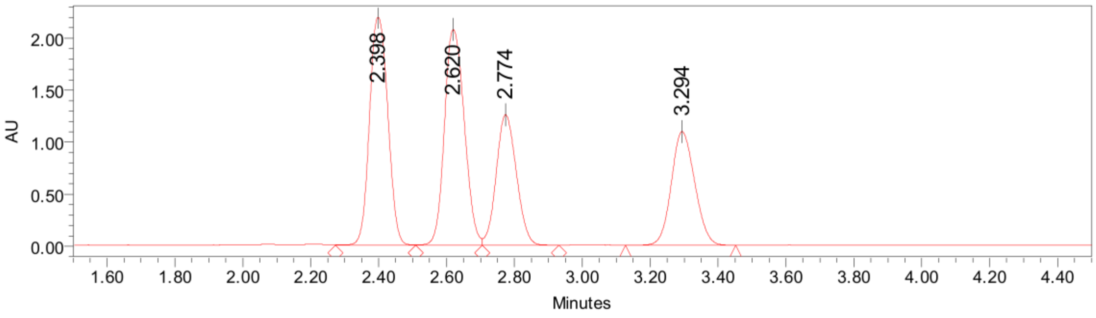

	RT	Area	% Area	Height
1	2.398	8208630	30.36	2190424
2	2.620	8437066	31.20	2071419
3	2.774	5180120	19.16	1254279
4	3.294	5215850	19.29	1093232

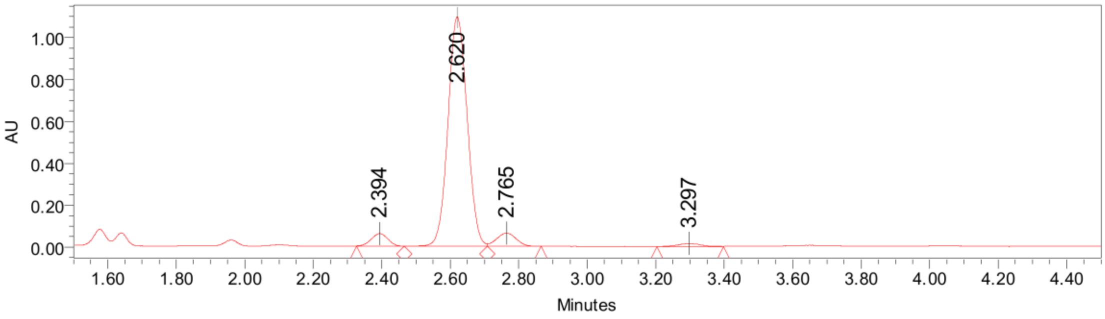

	RT	Area	% Area	Height
1	2.394	197317	4.16	59315
2	2.620	4240316	89.33	1094539
3	2.765	246361	5.19	62622
4	3.297	62562	1.32	13649

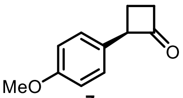

7a

(-)-2-(4-methoxyphenyl)cyclobutan-1-one (7a)

Prepared according to **general procedure**: 28 h, 44% yield, 95.1% ee.

$[\alpha]_D$^{27} **=** -15.0 (c = 1.0, $CHCl_3$)

The ee value was determined by the chiral UPC[2] analysis (CHIRALPAK® IG-3, CO_2/MeOH = 98/02, v = 2.0 mL/min, λ = 225.0 nm), t (major) = 3.039 min, t (minor) = 3.742 min.

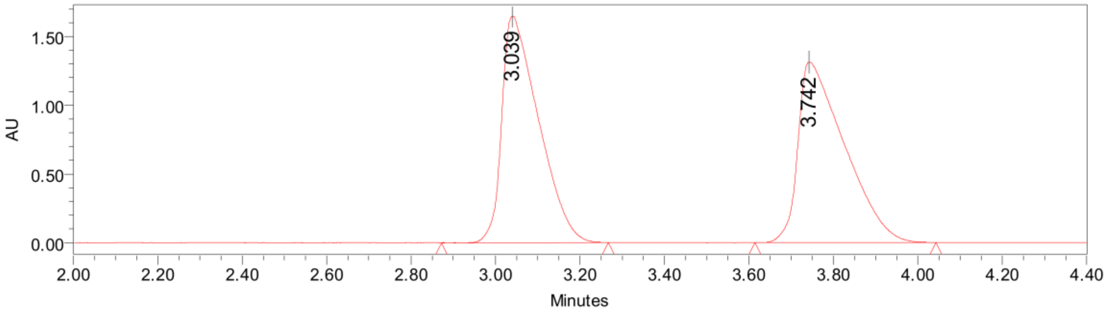

	RT	Area	% Area	Height
1	3.039	9977391	49.78	1647241
2	3.742	10064411	50.22	1311270

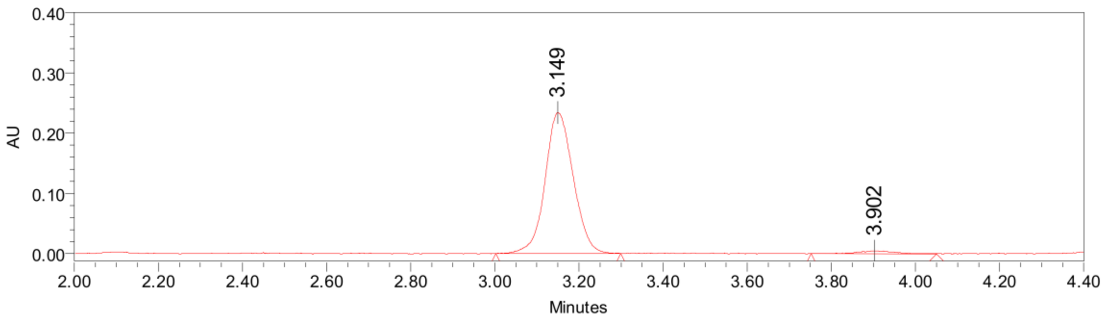

	RT	Area	% Area	Height
1	3.149	1090260	97.55	233691
2	3.902	27386	2.45	4410

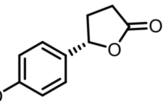

(-)-5-(4-methoxyphenyl)dihydrofuran-2(3H)-one (7b)

Prepared according to **general procedure**: 28 h, 54% yield, 90.8% ee, **7b**/**7c** = =16.6/1, white solid, m. p.: 51-52 ℃.

$[\alpha]_D^{27}$ = -10.0 (c = 1.0, $CHCl_3$)

^{1}H NMR (400 MHz, $CDCl_3$) δ 7.30-7.23 (m, 2H), 6.95-6.88 (m, 2H), 5.46 (dd, *J* = 8.2, 6.2 Hz, 1H), 3.81 (s, 3H), 2.70-2.56 (m, 3H), 2.28-2.13 (m, 1H).

^{13}C NMR (100 MHz, $CDCl_3$) δ 176.9, 159.7, 131.1, 126.9, 114.0, 81.3, 55.3, 30.9, 29.2.

HRMS (ESI) calculated for $C_{11}H_{12}NaO_3$ [M+Na$^+$]: 215.0679, found: 215.0676.

FT-IR (cm^{-1}): 2957, 2838, 1770, 1516, 1250, 1175, 1142, 1031, 937.

MS (EI) *m/z* (%): 137 (100), 135 (50), 148 (48), 192 (48), 109 (20), 117 (12), 161 (6).

The ee value was determined by the chiral UPC^2 analysis (Trefoil® CEL 1, CO_2/MeOH = 97/03, v = 2.0 mL/min, λ = 225.0 nm), t_b (minor) = 3.595 min, t_b (major) = 3.815 min, t_c = 4.148 min.

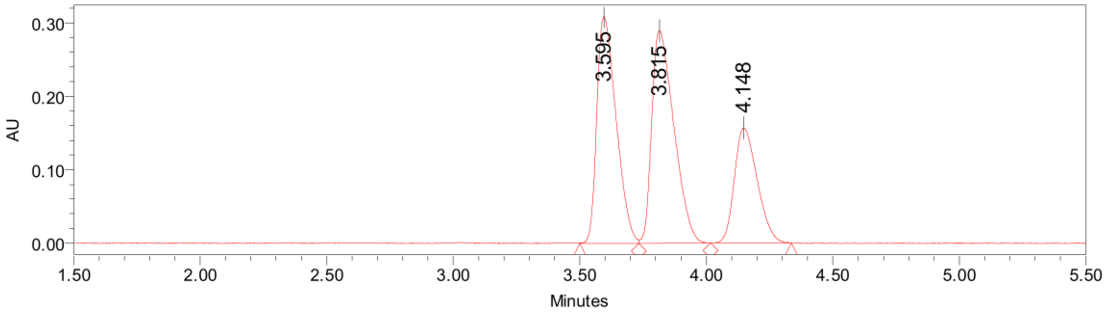

	RT	Area	% Area	Height
1	3.595	1636959	37.29	308895
2	3.815	1762993	40.17	289510
3	4.148	989362	22.54	157271

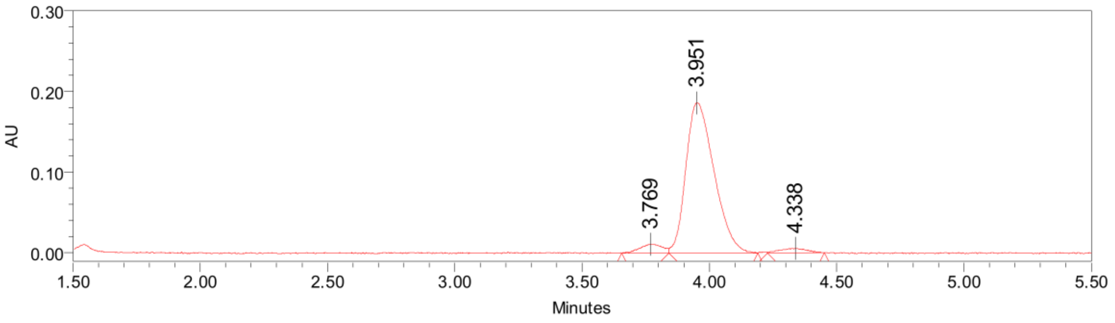

	RT	Area	% Area	Height
1	3.769	69225	4.47	11067
2	3.951	1437893	92.88	186465
3	4.338	40918	2.64	5465

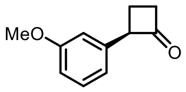

8a

(-)-2-(3-methoxyphenyl)cyclobutan-1-one (8a)

Prepared according to **general procedure**: 38 h, 47% yield, 95.3% ee.

[α]$_D^{25}$ = -9.0 (c = 1.0, $CHCl_3$)

The ee value was determined by the chiral UPC^2 analysis (CHIRALPAK® IG-3, CO_2/MeOH = 98/02, v = 2.0 mL/min, λ = 205.0 nm), t (major) = 3.299 min, t (minor) = 3.738 min.

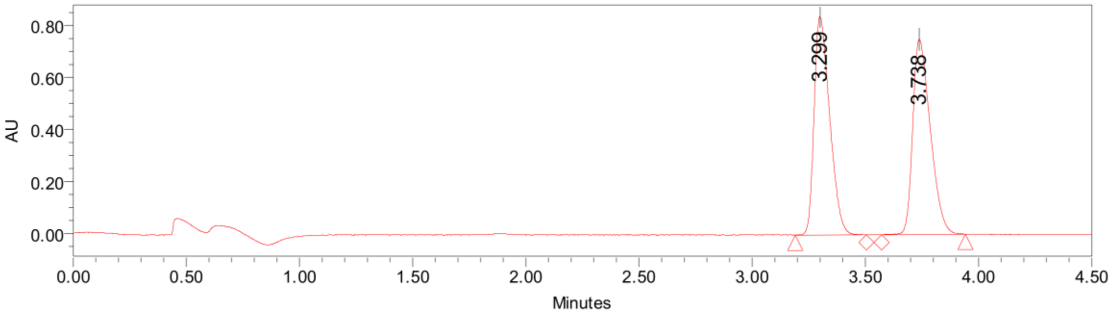

	RT	Area	% Area	Height
1	3.299	4011830	49.75	839849
2	3.738	4052192	50.25	750403

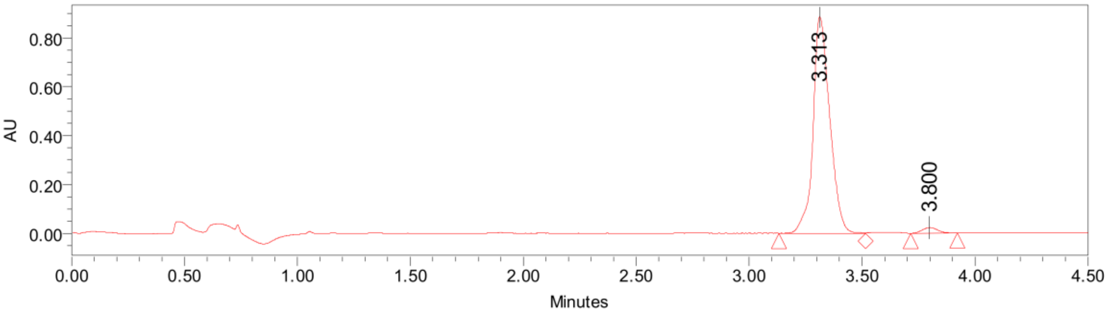

	RT	Area	% Area	Height
1	3.313	4560918	97.64	887731
2	3.800	110220	2.36	23407

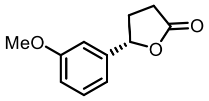

8b

(-)-5-(3-methoxyphenyl)dihydrofuran-2(3H)-one (8b)

Prepared according to **general procedure**: 38 h, 51% yield, 90.7% ee, **8b**/**8c** = 12.4/1, colorless liquid.

[α]$_D^{28}$ = -27.0 (c = 1.0, $CHCl_3$)

^{1}H NMR (400 MHz, $CDCl_3$) δ 7.34-7.28 (m, 1H), 6.93-6.85 (m, 3H), 5.53-5.45 (m, 1H), 3.82 (s, 3H), 2.72-2.60 (m, 3H), 2.26-2.12 (m, 1H).

^{13}C NMR (100 MHz, $CDCl_3$) δ 176.9, 159.9, 141.0, 129.8, 117.3, 113.8, 110.7, 81.0, 55.3, 30.9, 28.9.

HRMS (ESI) calculated for $C_{11}H_{12}NaO_3$ [M+Na$^+$]: 215.0679, found: 215.0674.

FT-IR (cm^{-1}): 3405, 2951, 2837, 1774, 1603, 1457, 1299, 1180, 1140, 1029, 907.

MS (EI) *m/z* (%): 192 (100), 137 (82), 135 (70), 109 (40), 148 (25), 161 (15), 117 (15).

The ee value was determined by the chiral UPC^2 analysis (CHIRALPAK® IC-3, CO_2/MeOH = 95/05, v = 2.0 mL/min, λ = 205.0 nm), t_b (minor) = 3.287 min, t_b (major) = 3.464 min, t_c (minor) = 4.836 min, t_c (major) = 5.274 min.

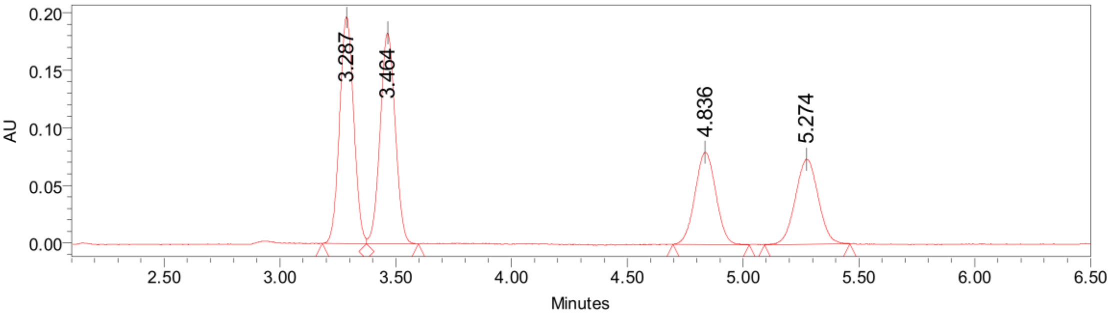

	RT	Area	% Area	Height
1	3.287	834967	31.23	197127
2	3.464	830035	31.05	182979
3	4.836	503359	18.83	80080
4	5.274	504917	18.89	73811

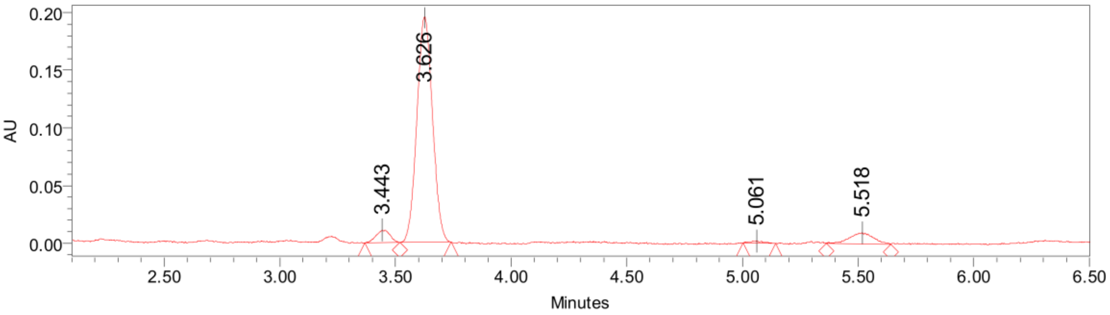

	RT	Area	% Area	Height
1	3.443	44932	4.32	10984
2	3.626	917499	88.23	195221
3	5.061	8063	0.78	1920
4	5.518	69429	6.68	9664

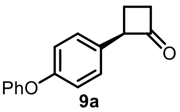

9a

(-)-2-(4-phenoxyphenyl)cyclobutan-1-one (9a)

Prepared according to **general procedure**: 36 h, 45% yield, 99.9% ee.

$[\alpha]_D^{29}$ = -16.0 (c = 1.0, $CHCl_3$)

The ee value was determined by the chiral UPC^2 analysis (CHIRALPAK® IG-3, CO_2/MeOH = 95/05, v = 2.0 mL/min, λ = 214.5 nm), t (minor) = 3.549 min, t (major) = 3.745 min.

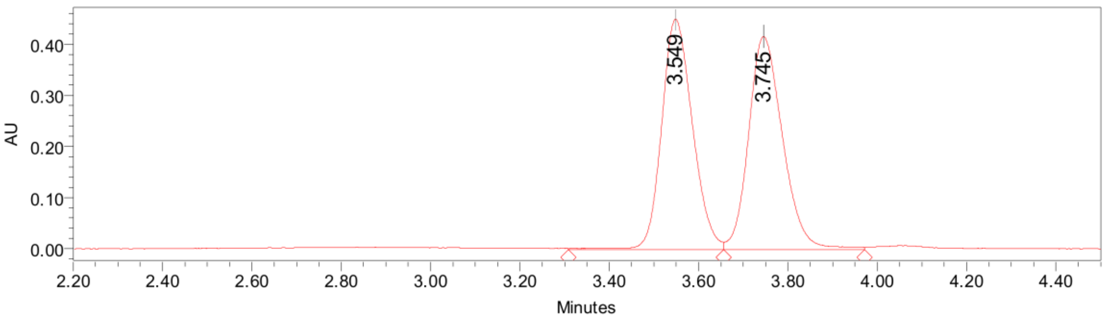

	RT	Area	% Area	Height
1	3.549	2166847	49.60	450865
2	3.745	2201742	50.40	416480

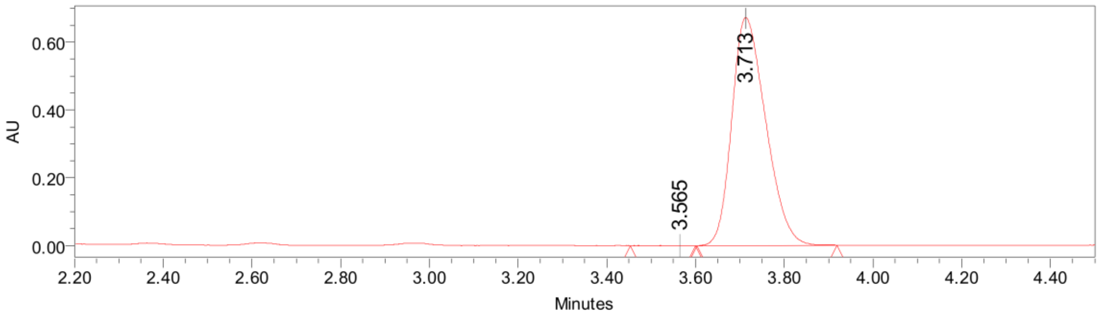

	RT	Area	% Area	Height
1	3.565	1538	0.04	-375
2	3.713	3569582	99.96	672108

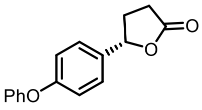

9b

(-)-5-(4-phenoxyphenyl)dihydrofuran-2(3H)-one (9b)

Prepared according to **general procedure**: 36 h, 48% yield, 93.2% ee, **9b**/**9c** = 12.5/1, white solid, m. p.: 59-61 °C.

$[\alpha]_D^{29}$ = -6.0 (c = 1.0, $CHCl_3$)

^{1}H NMR (600 MHz, $CDCl_3$) δ 7.35 (t, *J* = 7.8 Hz, 2H), 7.30 (d, *J* = 9.0 Hz, 2H), 7.13 (t, *J* = 7.2 Hz, 1H), 7.01 (d, *J* = 9.0 Hz, 4H), 5.52-5.45 (m, 1H), 2.69-2.60 (m, 3H), 2.26-2.14 (m, 1H).

^{13}C NMR (150 MHz, $CDCl_3$) δ 176.7, 157.6, 156.7, 133.8, 129.8, 127.0, 123.6, 119.1, 118.8, 81.0, 30.9, 29.1.

HRMS (ESI) calculated for $C_{16}H_{14}NaO_3$ $[M+Na^+]$: 277,0835, found: 77.0826.

FT-IR (cm^{-1}): 3440, 2924, 1773, 1589, 1489, 1238, 1168, 1022.

MS (EI) *m/z* (%): 254 (100), 199 (30), 255 (16), 210 (10), 117 (9), 115 (7).

the ee value was determined by the chiral UPC^2 analysis (CHIRALPAK® IG-3, CO_2/MeOH = 90/10, v = 2.0 mL/min, λ = 205 nm), t_b (minor) = 6.443 min, t_b (major) = 6.836 min, t_c = 5.544 min.

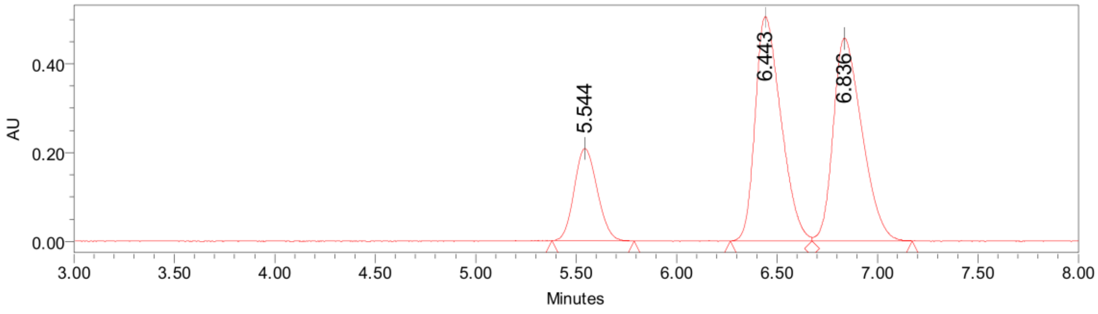

	RT	Area	% Area	Height
1	5.544	1668475	15.49	208570
2	6.443	4545561	42.21	506264
3	6.836	4555779	42.30	456865

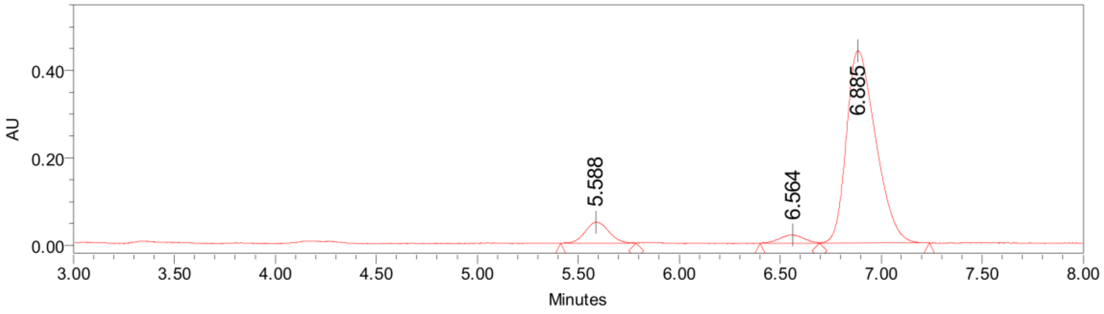

	RT	Area	% Area	Height
1	5.588	399678	8.02	48530
2	6.564	155119	3.11	18423
3	6.885	4427498	88.86	440377

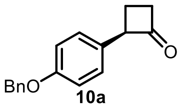

10a

(-)-2-(4-(benzyloxy)phenyl)cyclobutan-1-one (10a)

Prepared according to **general procedure**: 39 h, 43% yield, 99.4% ee.

$[\alpha]_D^{29}$ = -11.0 (c = 1.0, $CHCl_3$)

The ee value was determined by the chiral UPC^2 analysis (CHIRALPAK® IG-3, CO_2/MeOH = 90/10, v = 2.0 mL/min, λ = 205.0 nm), t (major) = 4.778 min, t (minor) = 5.934 min.

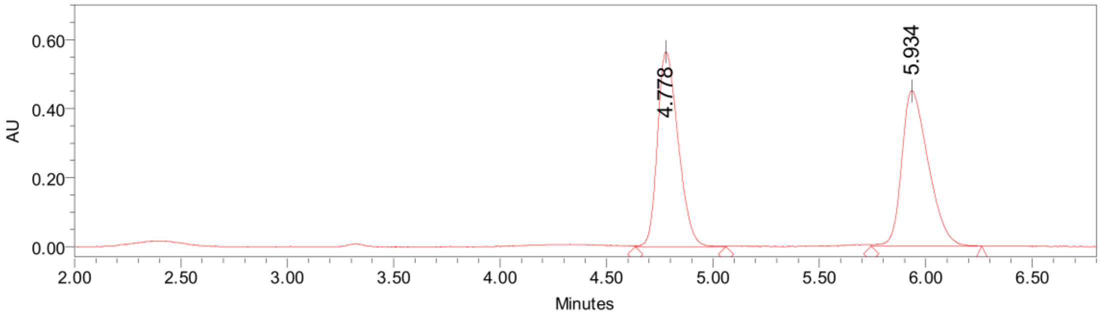

	RT	Area	% Area	Height
1	4.778	3821345	49.49	564208
2	5.934	3900496	50.51	449052

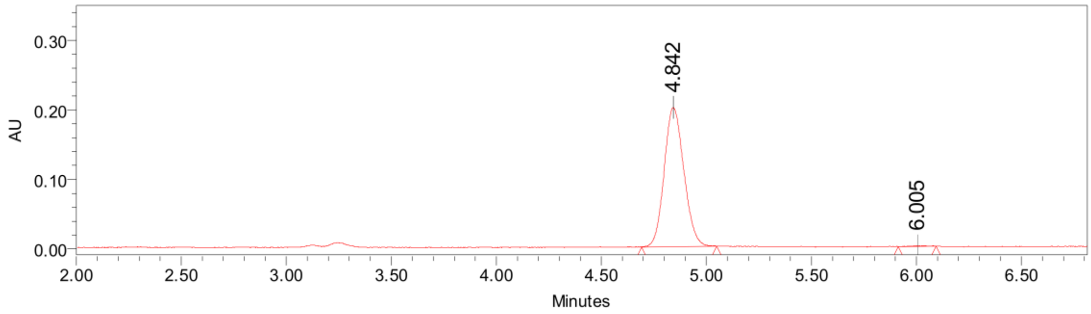

	RT	Area	% Area	Height
1	4.842	1301146	99.70	200114
2	6.005	3884	0.30	886

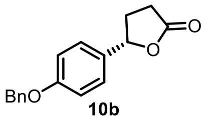

10b

(-)-5-(4-(benzyloxy)phenyl)dihydrofuran-2(3H)-one (10b)

Prepared according to **general procedure**: 39 h, 52% yield, 87.5% ee, **10b**/**10c** = 13.0/1, white solid, m. p.: 86-90 °C.

$[\alpha]_D^{29}$ = -9.0 (c = 1.0, $CHCl_3$)

^{1}H NMR (600 MHz, $CDCl_3$) δ 7.42 (d, *J* = 7.2 Hz, 2H), 7.38 (t, *J* = 7.5 Hz, 2H), 7.32 (t, *J* = 7.2 Hz, 1H), 7.25 (d, *J* = 8.4 Hz, 2H), 7.01-6.95 (m, 2H), 5.44 (dd, *J* = 8.1, 6.3 Hz, 1H), 5.06 (s, 2H), 2.67-2.55 (m, 3H), 2.23-2.13 (m, 1H).

^{13}C NMR (150 MHz, $CDCl_3$) δ 176.9, 158.9, 136.7, 131.4, 128.6, 128.0, 127.4, 126.9, 115.0, 81.2, 70.0, 30.8, 29.1.

HRMS (ESI) calculated for $C_{17}H_{16}NaO_3$ [M+Na$^+$]: 291.0992, found: 291.0984.

FT-IR (cm^{-1}): 3406, 2924, 1614, 1516, 1314, 1175, 1155, 1013, 936, 740.

MS (EI) *m/z* (%): 268 (100), 103 (14), 115 (13), 137 (10), 192 (10), 254 (4).

The ee value was determined by the chiral UPC2 analysis (CHIRALPAK® IC-3, CO_2/MeOH = 95/05, v = 2.0 mL/min, λ = 204.3 nm), t_b (minor) = 4.998 min, t_b (major) = 6.000 min, t_c (minor) = 5.326 min, t_c (major) = 5.768 min.

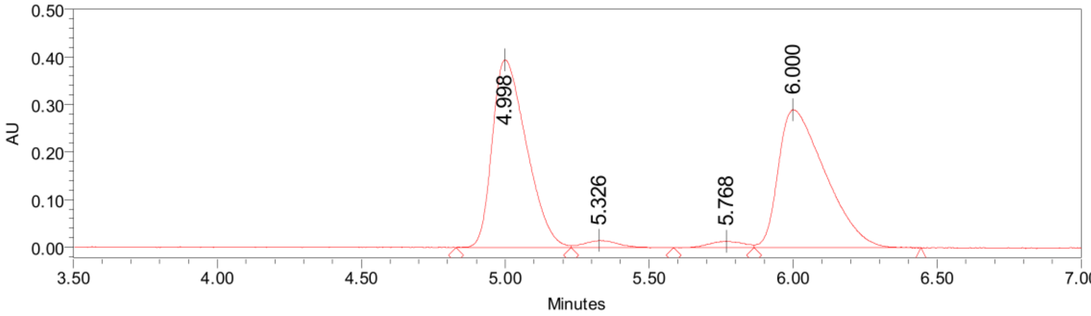

	RT	Area	% Area	Height
1	4.998	3267246	48.16	394477
2	5.326	122795	1.81	14972
3	5.768	109954	1.62	13450
4	6.000	3284089	48.41	290156

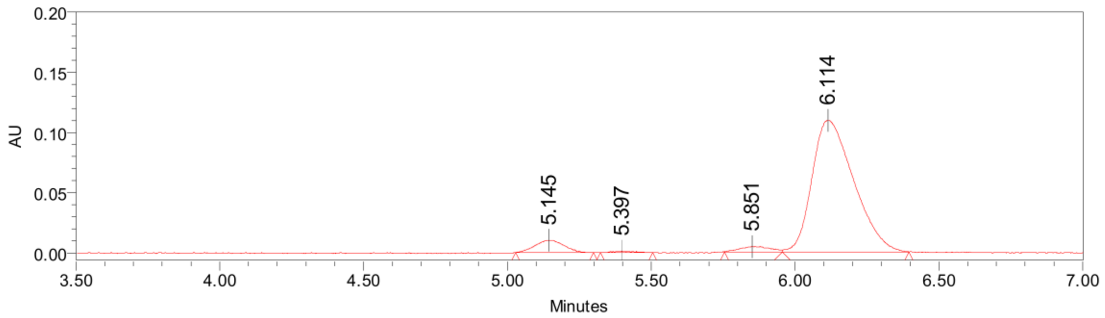

	RT	Area	% Area	Height
1	5.145	72882	6.03	10330
2	5.397	5027	0.42	992
3	5.851	31870	2.64	4350
4	6.114	1098965	90.92	109402

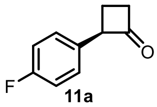

11a

(-)-2-(4-fluorophenyl)cyclobutan-1-one (11a)

Prepared according to **indicated procedure b**: 39 h, 49% yield, 81.6% ee.

$[\alpha]_D^{24}$ = -16.0 (c = 1.0, $CHCl_3$)

The ee value was determined by the chiral UPC[2] analysis (CHIRALPAK® IG-3, CO_2/MeOH = 98/02, v = 2.0 mL/min, λ = 215.0 nm), t (major) = 1.155 min, t (minor) = 1.324 min.

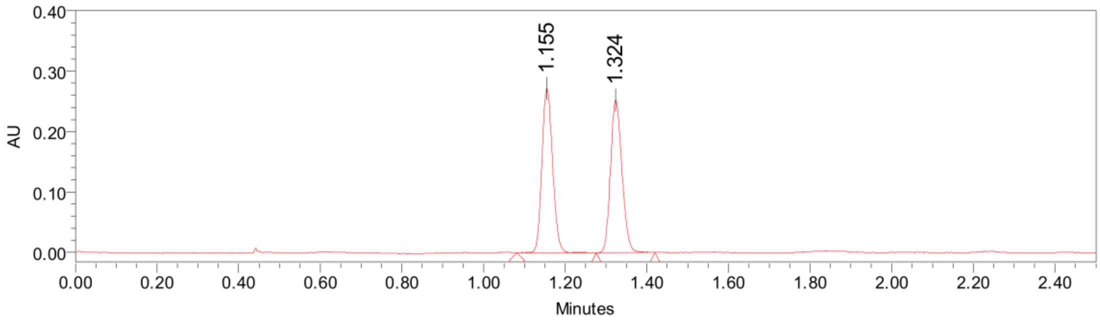

	RT	Area	% Area	Height
1	1.155	500518	50.34	272168
2	1.324	493711	49.66	252697

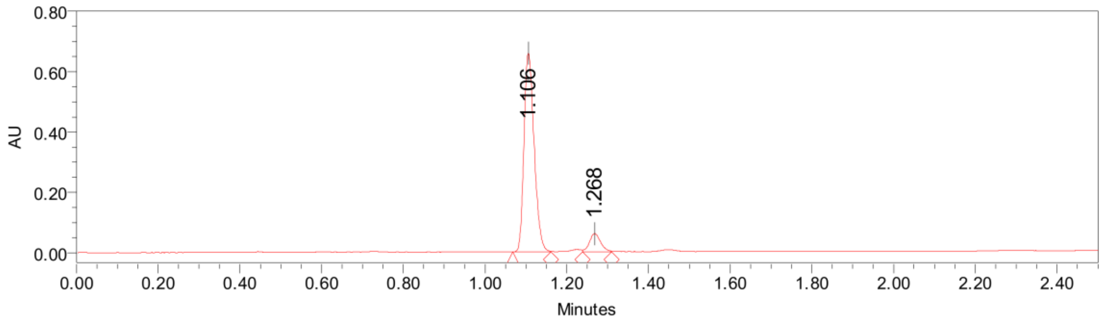

	RT	Area	% Area	Height
1	1.106	1132794	90.83	658774
2	1.268	114335	9.17	60073

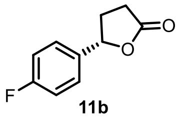

11b

(*S*)-5-(4-fluorophenyl)dihydrofuran-2(3H)-one (11b)

Prepared according to **indicated procedure b**: 39 h, 50% yield, 91.7% ee, **11b/11c** = 6.3/1, colorless liquid.

[α]$_D$^{24} = -15.0 (c = 1.0, $CHCl_3$)

^{1}H NMR (600 MHz, $CDCl_3$) δ 7.36-7.29 (m, 2H), 7.15-7.04 (m, 2H), 5.49 (dd, *J* = 8.1, 6.3 Hz, 1H), 2.74-2.61 (m, 3H), 2.25-2.11 (m, 1H).

^{13}C NMR (150 MHz, $CDCl_3$) δ 176.6, 162.6 (d, *J* = 246 Hz), 135.1 (d, *J* = 3.2 Hz), 127.2 (d, *J* = 8.6 Hz), 115.7 (d, *J* = 21.5 Hz), 80.6, 31.0, 29.0.

^{19}F NMR (376 MHz, CDCl3) δ -113.31.

HRMS (ESI) calculated for $C_{10}H_9NaO_2F$ [M+Na$^+$]: 203.0479, found: 203.0479.

FT-IR (cm^{-1}): 2924, 1770, 1513, 1226, 1140, 1022, 939, 837.

MS (EI) *m/z* (%): 56 (100), 125 (60), 135 (50), 95 (45), 180 (38), 109 (34).

The ee value was determined by the chiral UPC^2 analysis (CHIRALPAK® IC-3, CO_2/MeOH = 98/02, v = 2.0 mL/min, λ = 210.0 nm), t_b (minor) = 4.155 min, t_b (major) = 4.430 min, t_c (minor) = 5.099 min, t_c (major) = 5.263 min.

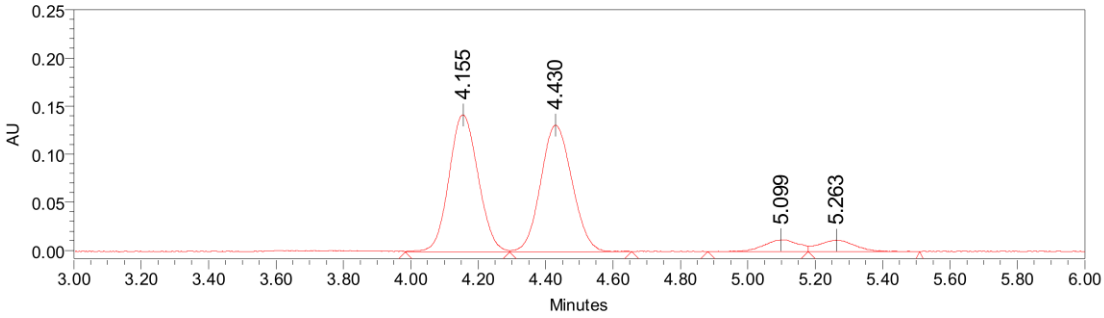

	RT	Area	% Area	Height
1	4.155	857264	45.32	142544
2	4.430	854291	45.16	131876
3	5.099	89846	4.75	12446
4	5.263	90245	4.77	11992

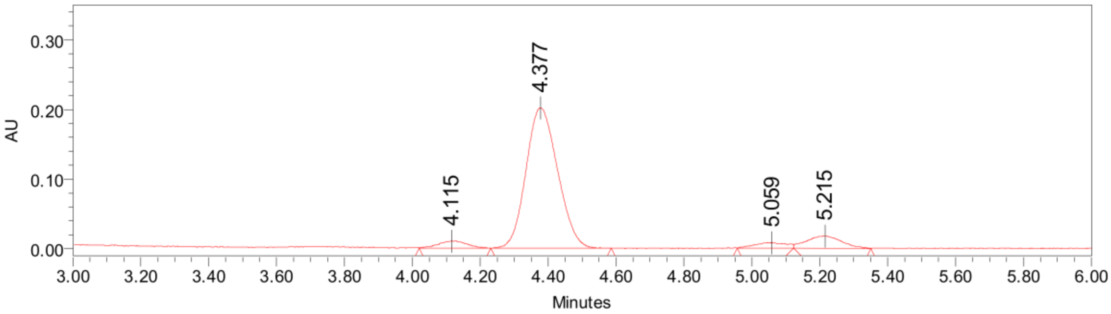

	RT	Area	% Area	Height
1	4.115	56831	3.69	10018
2	4.377	1306970	84.76	201630
3	5.059	51205	3.32	7709
4	5.215	126941	8.23	17655

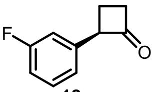

12a

(-)-2-(3-fluorophenyl)cyclobutan-1-one (12a)

Prepared according to **indicated procedure b**: 39 h, 48% yield, 88.4% ee.

[α]$_D$^{24} = -39.0 (c = 1.0, $CHCl_3$)

The ee value was determined by the chiral UPC^2 analysis (CHIRALPAK® IG-3, CO_2/MeOH = 98/02, v = 2.0 mL/min, λ = 215.0 nm), t (major) = 1.240 min, t (minor) = 1.390 min.

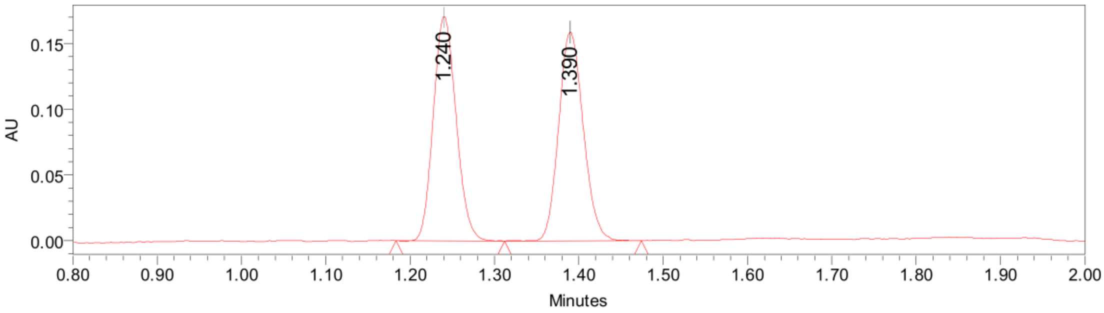

	RT	Area	% Area	Height
1	1.240	321280	49.88	170652
2	1.390	322869	50.12	158953

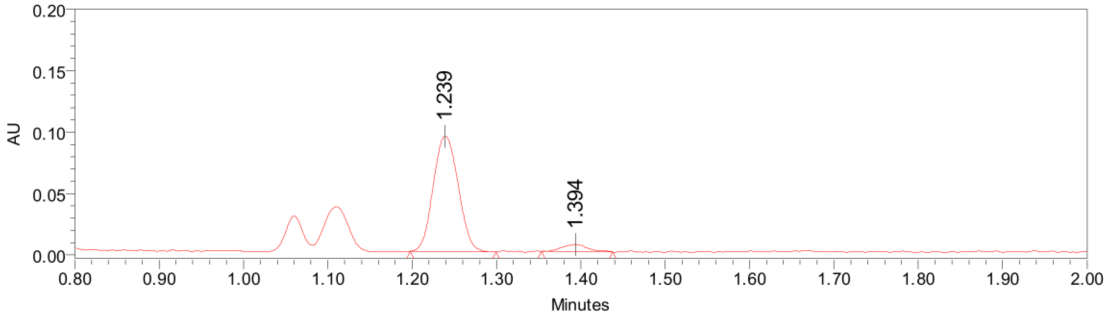

	RT	Area	% Area	Height
1	1.239	193588	94.18	94023
2	1.394	11974	5.82	5820

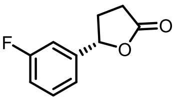

12b

(-)-5-(3-fluorophenyl)dihydrofuran-2(3H)-one (12b)

Prepared according to **indicated procedure b**: 39 h, 50% yield, 90.8% ee, **12b**/**12c** = 5.0/1, yellow liquid.

[α]$_D$^{24} = - 19.0 (c = 1.0, $CHCl_3$)

^{1}H NMR (400 MHz, $CDCl_3$) δ 7.42-7.32 (m, 1H), 7.15-6.99 (m, 3H), 5.57-5.45 (m, 1H), 2.78-2.61 (m, 3H), 2.25-2.11 (m, 1H).

^{13}C NMR (150 MHz, $CDCl_3$) δ 176.5, 162.9 (d, *J* = 245.7 Hz), 142.0 (d, *J* = 7.2 Hz), 130.5 (d, *J* = 3.8 Hz), 120.72 (d, *J* = 3.0 Hz), 115.3 (d, *J* = 20.9 Hz), 112.3 (d, *J* = 22.7 Hz), 80.2, 30.8, 28.7.

^{19}F NMR (376 MHz, $CDCl_3$) δ -111.93.

HRMS (ESI) calculated for $C_{10}H_{10}O_2F$ [M+H$^+$]: 181.0659, found: 181.0659.

FT-IR (cm^{-1}): 2924, 1773, 1292, 1181, 1140, 1024, 911, 798.

MS (EI) *m/z* (%): 56 (100), 123 (66), 180 (180), 135 (40), 95 (38), 109 (24), 115 (18).

The ee value was determined by the chiral UPC^2 analysis (CHIRALPAK® IC-3, CO_2/MeOH = 97/03, v = 2.0 mL/min, λ = 213.0 nm), t_b (minor) = 2.479 min, t_b (major) = 2.718 min, t_c (minor) = 3.261 min, t_c (major) = 3.396 min.

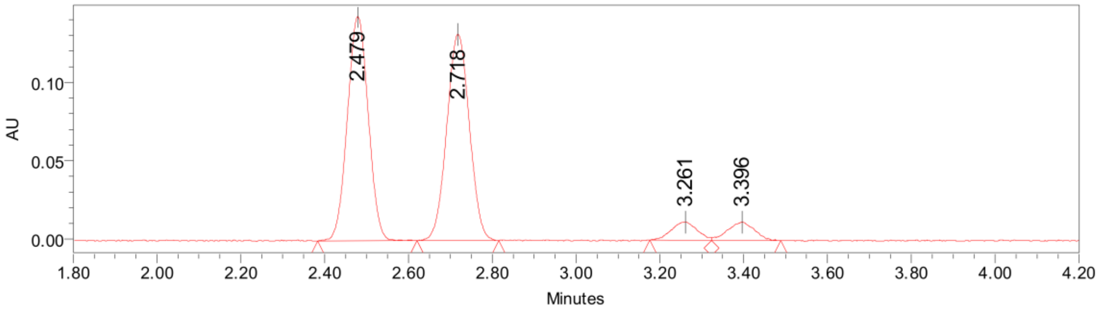

	RT	Area	% Area	Height
1	2.479	495979	45.38	142961
2	2.718	494122	45.21	131418
3	3.261	50005	4.58	11640
4	3.396	52753	4.83	11841

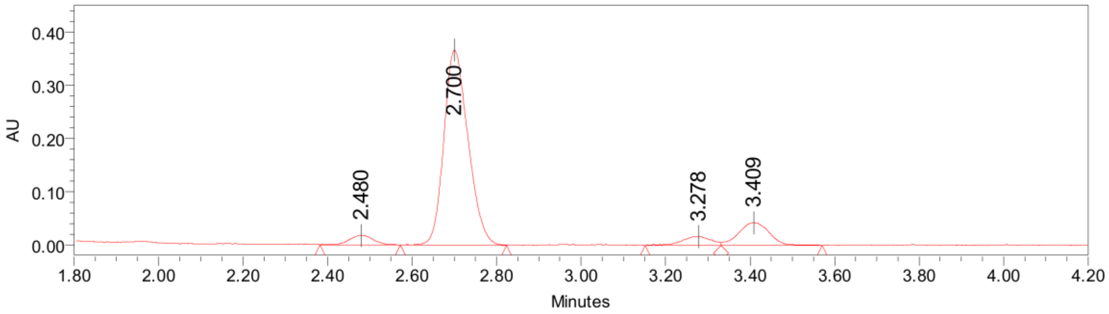

	RT	Area	% Area	Height
1	2.480	70187	3.86	17818
2	2.700	1456352	80.06	366444
3	3.278	79368	4.36	16166
4	3.409	213132	11.72	42503

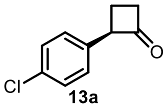

13a

(-)-2-(4-chlorophenyl)cyclobutan-1-one (13a)

Prepared according to **indicated procedure b**: 36 h, 43% yield, 86.7% ee.

$[\alpha]_D^{24}$ = -23.0 (c = 1.0, $CHCl_3$)

The ee value was determined by the chiral UPC^2 analysis (CHIRALPAK® IG-3, CO_2/MeOH = 98/02, v = 2.0 mL/min, λ = 214.7 nm), t (major) = 2.179 min, t (minor) = 2.782 min.

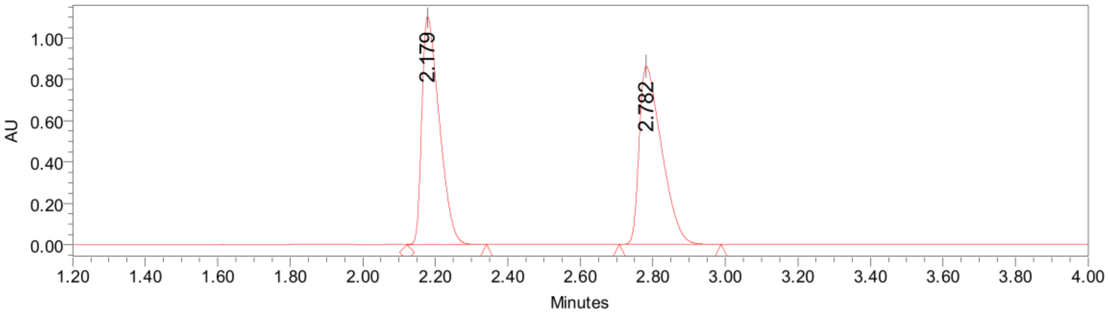

	RT	Area	% Area	Height
1	2.179	3694403	49.95	1099739
2	2.782	3701462	50.05	860402

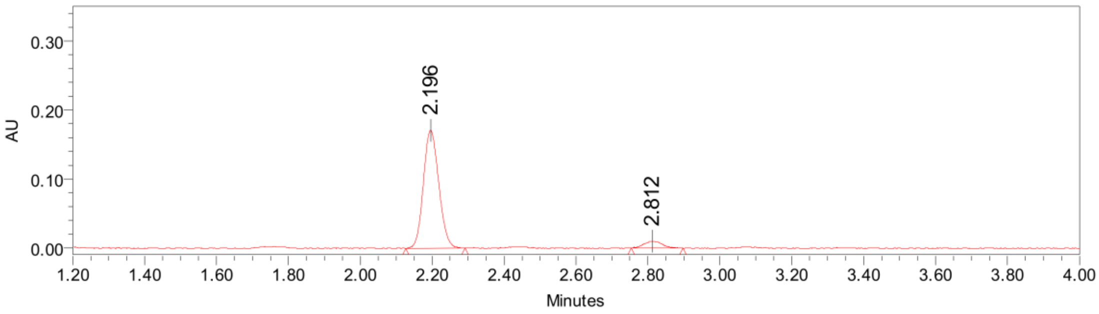

	RT	Area	% Area	Height
1	2.196	508047	93.35	170870
2	2.812	36179	6.65	9976

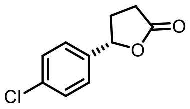

13b

(-)-5-(4-chlorophenyl)dihydrofuran-2(3H)-one (13b)

Prepared according to **indicated procedure b**: 36 h, 56% yield, 92.3% ee, **13b/13c** = 5.9/1, white solid, m. p.: 47-49 °C.

$[\alpha]_D^{24}$ = -28.0 (c = 1.0, $CHCl_3$)

^{1}H NMR (400 MHz, $CDCl_3$) δ 7.43-7.33 (m, 2H), 7.34-7.22 (m, 2H), 5.48 (dd, *J* = 8.2, 6.2 Hz, 1H), 2.76-2.59 (m, 3H), 2.26-2.05 (m, 1H).

^{13}C NMR (100 MHz, $CDCl_3$) δ 176.5, 137.8, 134.2, 128.9, 126.6, 80.4, 30.9, 28.8.

HRMS (ESI) calculated for $C_{10}H_{10}O_2Cl$ [M+H$^+$]: 197.0364, found: 197.0363.

FT-IR (cm^{-1}): 2924, 1770, 1493, 1173, 1139, 1013, 938, 806.

MS (EI) *m/z* (%): 56 (100), 141 (80), 161 (40), 117 (14), 115 (40), 196 (30), 197 (10).

The ee value was determined by the chiral UPC^2 analysis (Trefoil® CEL 1, CO_2/MeOH = 98/02, v = 2.0 mL/min, λ = 219.0 nm), t_b (minor) = 4.472 min, t_b (major) = 4.770 min, t_c (minor) = 5.903 min, t_c (major) = 6.374 min.

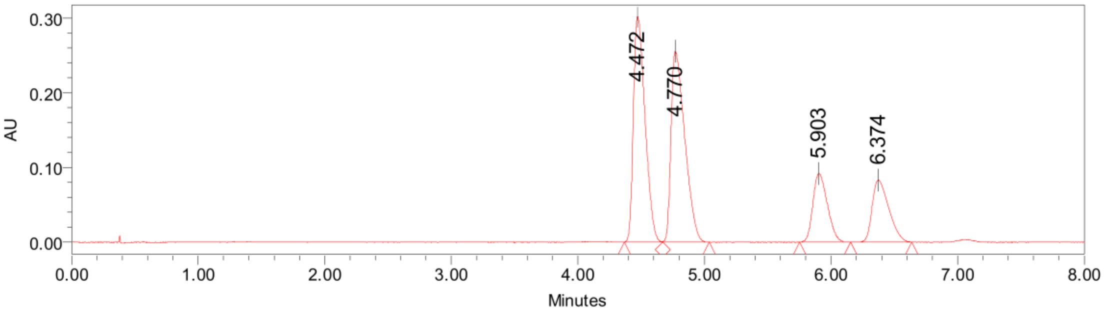

	RT	Area	% Area	Height
1	4.472	1919902	35.98	302153
2	4.770	1922332	36.03	255519
3	5.903	730506	13.69	91928
4	6.374	762840	14.30	83269

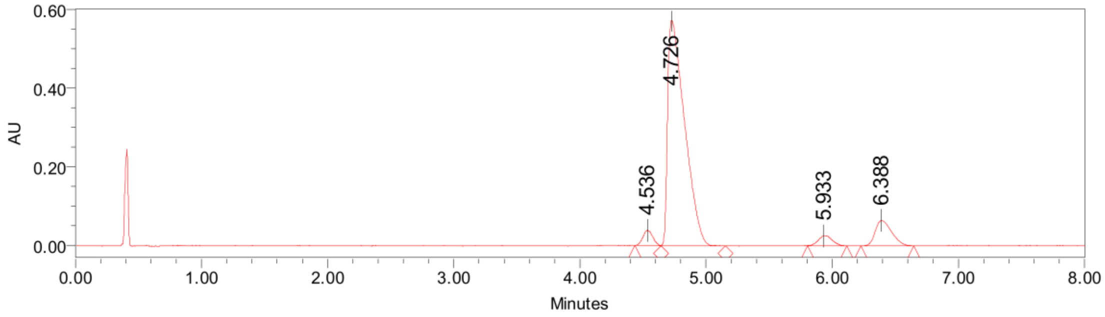

	RT	Area	% Area	Height
1	4.536	210529	3.34	38680
2	4.726	5311576	84.21	572443
3	5.933	197564	3.13	25475
4	6.388	587747	9.32	64711

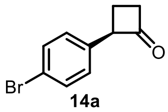

14a

(-)-2-(4-bromophenyl)cyclobutan-1-one (14a)

Prepared according to **indicated procedure b**: 38 h, 45% yield, 85.3% ee.

$[\alpha]_D^{24}$ = -22.0 (c = 1.0, $CHCl_3$)

The ee value was determined by the chiral UPC[2] analysis (CHIRALPAK® IG-3, CO_2/MeOH = 99/01, v = 2.0 mL/min, λ = 225.0 nm), t (major) = 4.954 min, t (minor) = 6.782min.

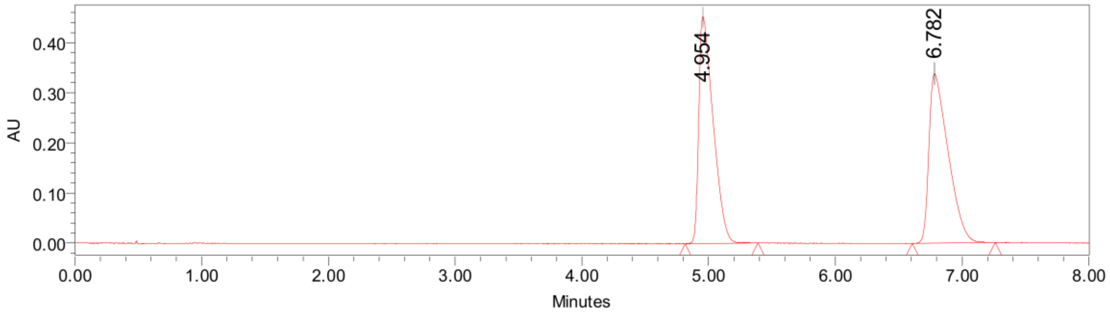

	RT	Area	% Area	Height
1	4.954	3559735	49.98	453550
2	6.782	3563232	50.02	338465

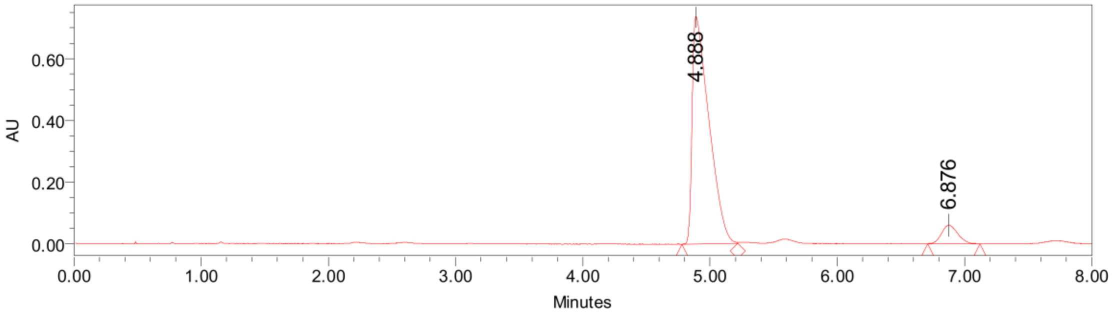

	RT	Area	% Area	Height
1	4.888	6775718	92.68	738203
2	6.876	535540	7.32	60534

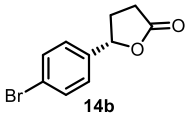

14b

(-)-5-(4-bromophenyl)dihydrofuran-2(3H)-one (14b)

Prepared according to **indicated procedure b**: 38 h, 50% yield, 92.4% ee, **14b**/**14c** = 6.3/1, white solid, m. p.: 79-82 °C.

[α]D²⁴ = -24.0 (c = 1.0, $CHCl_3$)

^{1}H NMR (400 MHz, $CDCl_3$) δ 7.57-7.47 (m, 2H), 7.26-7.17 (m, 2H), 5.52-5.41 (m, 1H), 2.75-2.58 (m, 3H), 2.24-2.07 (m, 1H).

^{13}C NMR (100 MHz, $CDCl_3$) δ 176.5, 138.4, 131.9, 126.9, 122.3, 80.4, 30.9, 28.8.

HRMS (ESI) calculated for $C_{10}H_{10}O_2Br$ [M+H$^+$]: 240.9859, 242.9838, found: 240.9858, 242.9838.

FT-IR (cm^{-1}): 2914, 1760, 1698, 1415, 1176, 1008, 804, 746.

MS (EI) *m/z* (%): 56 (100), 161 (70), 185 (40), 115 (40), 242 (30), 240 (20), 157 (20).

The ee value was determined by the chiral UPC^2 analysis (Trefoil® CEL 1, CO_2/MeOH = 95/05, v = 2.0 mL/min, λ = 220.0 nm), t_b (minor) = 3.070 min, t_b (major) = 3.272 min, t_c (minor) = 3.804 min, t_c (major) = 4.214 min.

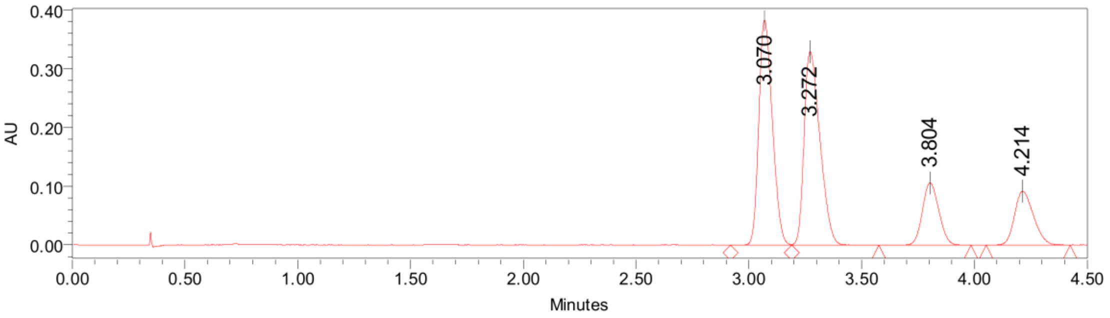

	RT	Area	% Area	Height
1	3.070	1602654	37.42	383291
2	3.272	1603435	37.44	329410
3	3.804	540253	12.61	106243
4	4.214	536554	12.53	91705

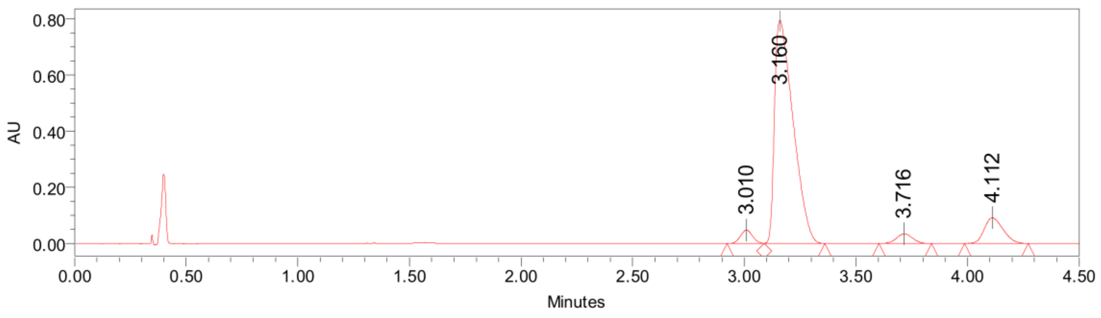

	RT	Area	% Area	Height
1	3.010	181312	3.31	48049
2	3.160	4585115	83.74	794665
3	3.716	175397	3.20	34646
4	4.112	533756	9.75	91785

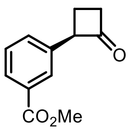

15a

(-)-methyl (*R*)-3-(2-oxocyclobutyl)benzoate (15a)

Prepared according to **general procedure**: 45% yield, 92.1% ee.

[α]D²⁴ = -24.0 (c = 1.0, $CHCl_3$)

The ee value was determined by the chiral UPC^2 analysis (CHIRALPAK® IG-3, CO_2/MeOH = 95/05, v = 2.0 mL/min, λ = 210.0 nm), t (major) = 2.542 min, t (minor) = 2.756min.

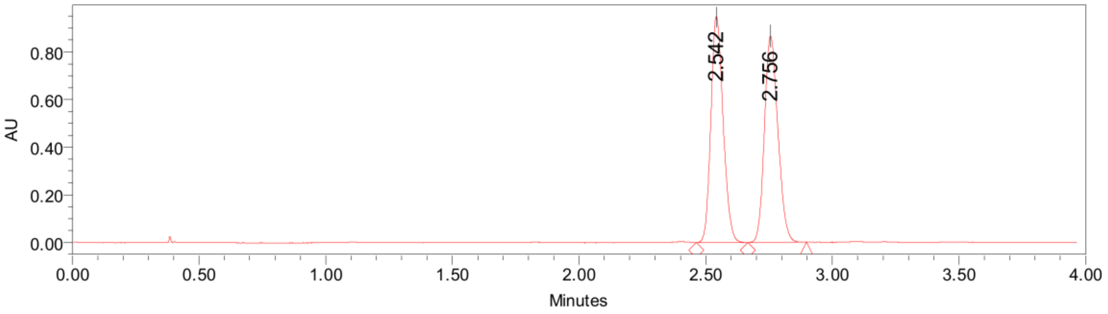

	RT	Area	% Area	Height
1	2.542	3207620	49.93	949786
2	2.756	3216437	50.07	866855

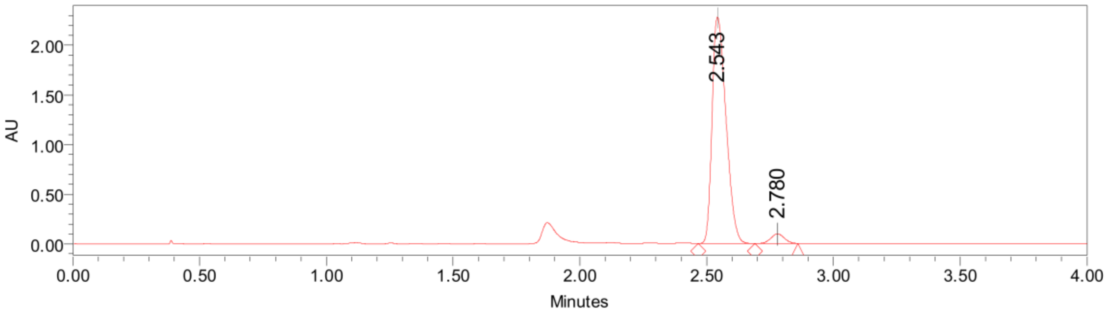

	RT	Area	% Area	Height
1	2.543	8707633	96.04	2280884
2	2.780	359070	3.96	96670

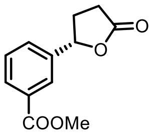

15b

(-)-methyl (*S*)-3-(5-oxotetrahydrofuran-2-yl)benzoate (15b)

Prepared according to **general procedure**: 53.1% yield, 90.5% ee, **15b**/**15c** = 6.1/1, colorless liquid.

$[\alpha]_D^{24}$ = -18.0 (c = 1.0, $CHCl_3$)

^{1}H NMR (600 MHz, $CDCl_3$) δ 8.04-7.99 (m, 2H), 7.55 (d, *J* = 7.8 Hz, 1H), 7.48 (t, *J* = 7.8 Hz, 1H), 5.56 (dd, *J* = 8.1, 6.3 Hz, 1H), 3.93 (s, 3H), 2.76-2.66 (m, 3H), 2.25-2.14 (m, 1H).

^{13}C NMR (150 MHz, $CDCl_3$) δ 176.5, 166.5, 139.8, 130.7, 129.7, 129.5, 128.9, 126.3, 80.5, 52.2, 30.9, 28.8.

HRMS (ESI) calculated for $C_{12}H_{12}NaO_4$ [M+Na$^+$]: 243.0628, found: 243.0628.

FT-IR (cm^{-1}): 2953, 1777, 1722, 1291, 1211, 1109, 753, 698.

MS (EI) *m/z* (%): 56 (100), 165 (92), 115 (74), 133 (70), 161 (60), 105 (52), 189 (30), 220 (25).

The ee value was determined by the chiral UPC^2 analysis (CHIRALPAK® IG-3, CO_2/MeOH = 95/05, v = 2.0 mL/min, λ = 227.0 nm), t_b (major) = 7.834 min, t_b (minor) = 9.882 min, t_c = 8.663.

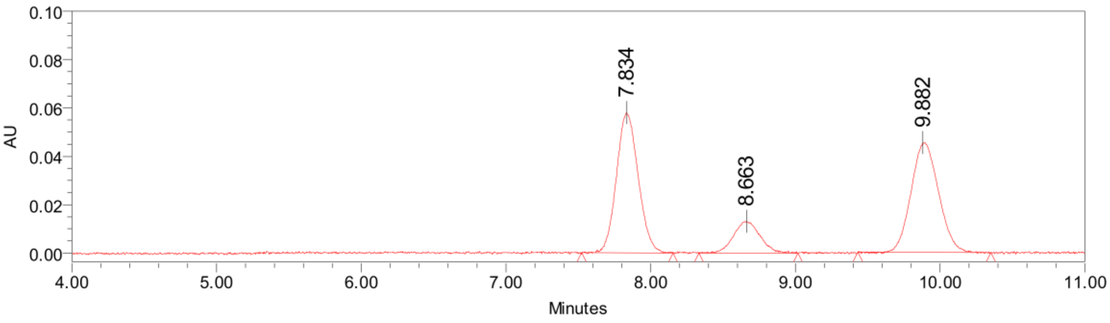

	RT	Area	% Area	Height
1	7.834	606765	44.06	58052
2	8.663	167595	12.17	13091
3	9.882	602905	43.78	45396

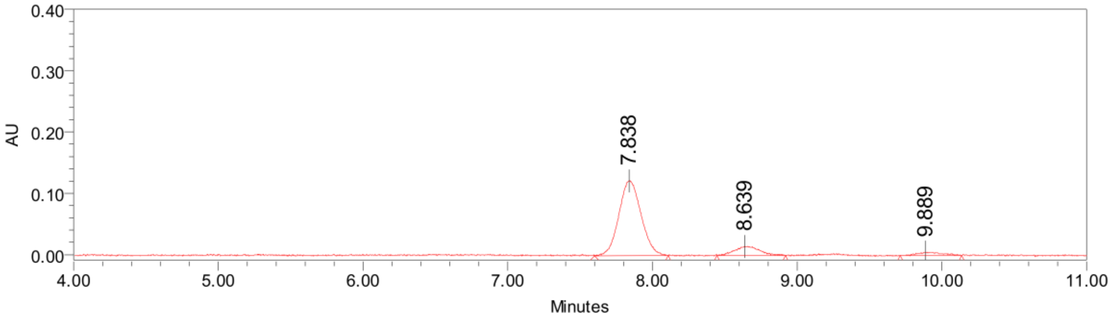

	RT	Area	% Area	Height
1	7.838	1298734	84.14	121939
2	8.639	180369	11.69	14836
3	9.889	64483	4.18	5414

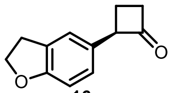

16a

(-)-2-(2,3-dihydrobenzofuran-5-yl)cyclobutan-1-one (16a)

Prepared according to **general procedure**: 38 h, 40% yield, 94.7% ee.

[α]$_D^{29}$ = -5.0 (c = 1.0, $CHCl_3$)

The ee value was determined by the chiral UPC^2 analysis (CHIRALPAK® IG-3, CO_2/MeOH = 95/05, v = 2.0 mL/min, λ = 202.6 nm), t (major) = 2.468 min, t (minor) = 2.891 min.

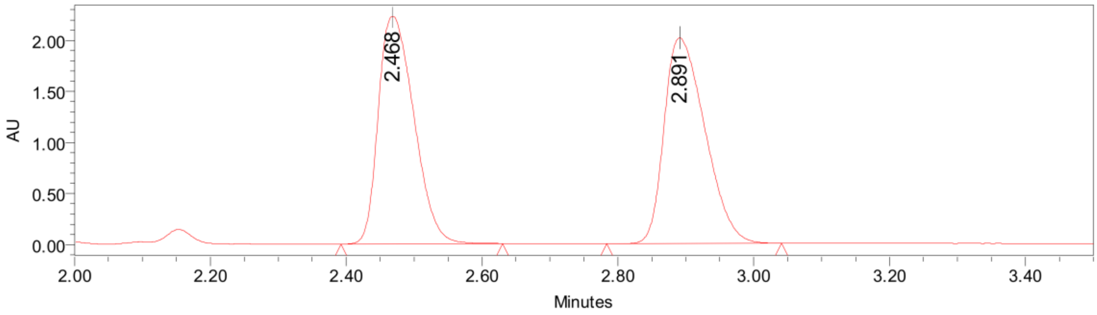

	RT	Area	% Area	Height
1	2.468	8129225	49.15	2225875
2	2.891	8410340	50.85	2012880

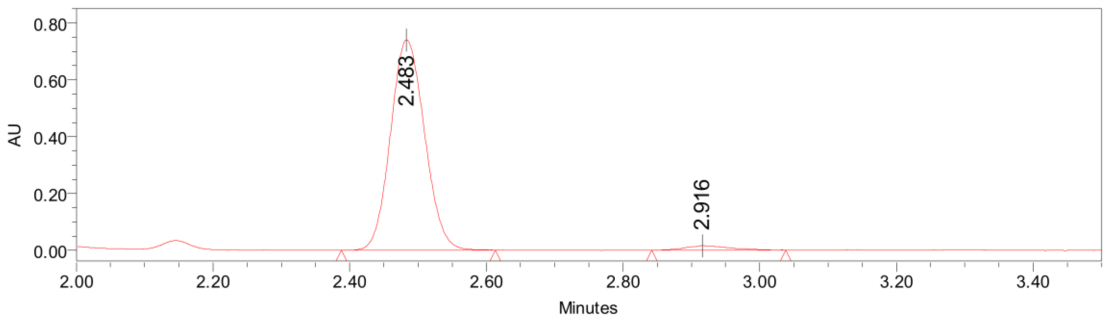

	RT	Area	% Area	Height
1	2.483	2575710	97.35	739385
2	2.916	70071	2.65	15508

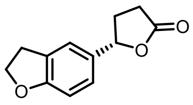

16b

(*S*)-5-(2,3-dihydrobenzofuran-5-yl)dihydrofuran-2(3H)-one (16b)

Prepared according to **general procedure**: 38 h, 56% yield, 86.5% ee, **16b**/**16c** = 16.7/1, colorless liquid.

$[\alpha]_D^{29}$ = -9.0 (c = 1.0, $CHCl_3$)

^{1}H NMR (600 MHz, $CDCl_3$) δ 7.19 (s, 1H), 7.06 (dd, *J* = 8.1, 0.9 Hz, 1H), 6.77 (d, *J* = 8.4 Hz, 1H), 5.44 (dd, *J* = 8.4, 6.6 Hz, 1H), 4.59 (t, *J* = 8.7 Hz, 2H), 3.21 (t, *J* = 8.7 Hz, 2H), 2.69-2.63 (m, 2H), 2.63-2.56 (m, 1H), 2.24-2.15 (m, 1H).

^{13}C NMR (150 MHz, $CDCl_3$) δ 176.9, 160.4, 131.1, 127.7, 125.8, 122.4, 109.2, 81.7, 71.5, 31.0, 29.5, 29.3.

HRMS (ESI) calculated for $C_{12}H_{12}NaO_3$ [M+Na$^+$]: 227.0679, found: 227.0673.

FT-IR (cm^{-1}): 3441, 2921, 1771, 1494, 1178, 1143, 982, 909, 806.

MS (EI) *m/z* (%): 84 (100), 86 (66), 51 (64), 69 (50), 149 (44), 204 (40).

The ee value was determined by the chiral UPC2 analysis (CHIRALPAK® IG-3, CO_2/MeOH = 90/10, v = 2.0 mL/min, λ = 202.6 nm), t_b (minor) = 3.921 min, t_b (major) = 4.222 min.

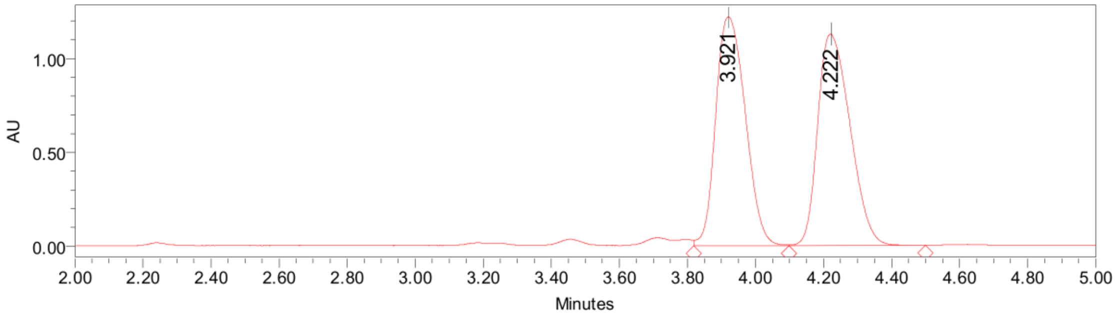

	RT	Area	% Area	Height
1	3.921	7357680	49.70	1220034
2	4.222	7446361	50.30	1127800

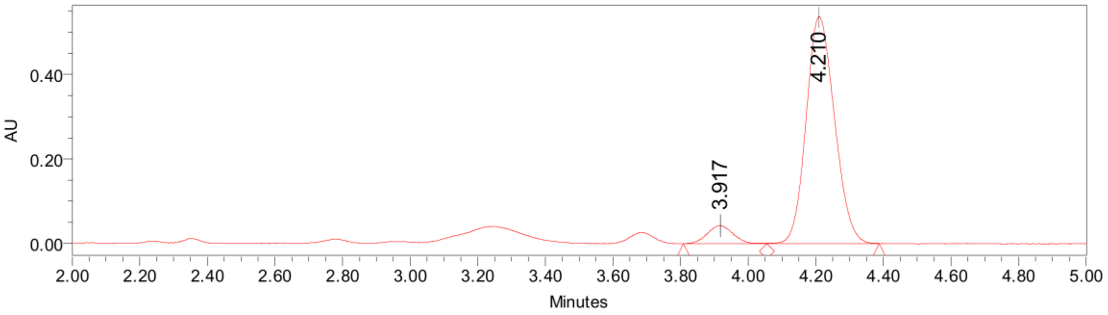

	RT	Area	% Area	Height
1	3.917	226161	6.72	42840
2	4.210	3139486	93.28	537285

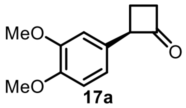

17a

(-)-2-(3,4-dimethoxyphenyl)cyclobutan-1-one (17a)

Prepared according to **general procedure**: 39 h, 40% yield, 98.5% ee.

$[\alpha]_D^{30}$ = -17.0 (c = 1.0, $CHCl_3$)

The ee value was determined by the chiral UPC^2 analysis (CHIRALPAK® IC-3, CO_2/MeOH = 90/10, v = 2.0 mL/min, λ = 205.0 nm), t (major) = 3.615 min, t (minor) = 3.956 min.

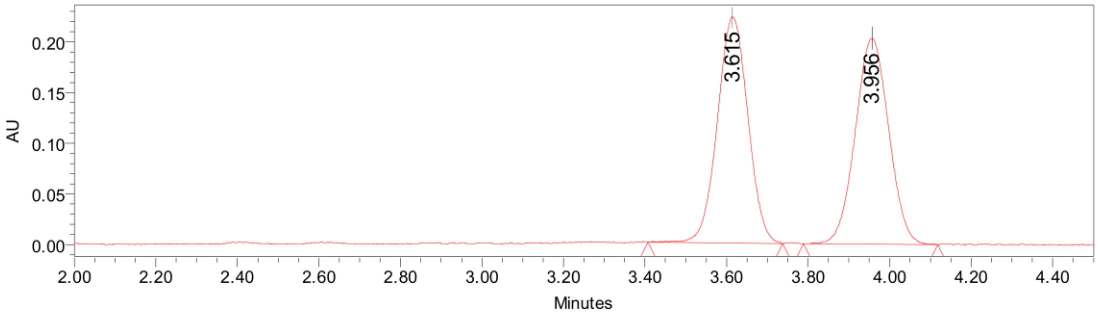

	RT	Area	% Area	Height
1	3.615	1164523	50.08	223371
2	3.956	1160982	49.92	203478

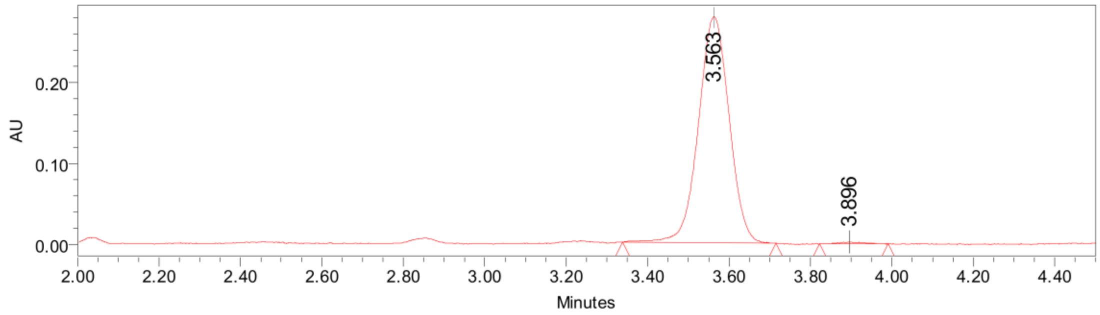

	RT	Area	% Area	Height
1	3.563	1462214	99.26	279126
2	3.896	10971	0.74	2279

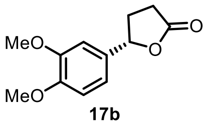

17b

(-)-5-(3,4-dimethoxyphenyl)dihydrofuran-2(3H)-one (17b)

Prepared according to **general procedure**: 39 h, 58% yield, 87.0% ee, **17b**/**17c** = 14.3/1, white solid, m. p.: 114-116 °C.

$[\alpha]_D^{30}$ = - 15.0 (c = 1.0, $CHCl_3$)

^{1}H NMR (600 MHz, $CDCl_3$) δ 6.87 (s, 2H), 6.86 (s, 1H), 5.46 (dd, *J* = 8.4, 6.0 Hz, 1H), 3.90 (s, 3H), 3.89 (s, 3H), 2.69-2.65 (m, 2H), 2.65-2.60 (m, 1H), 2.25-2.17 (m, 1H).

13C NMR (150 MHz, CDCl3) δ 176.9, 149.2, 149.1, 131.6, 117.9, 111.0, 108.5, 81.4, 55.9, 30.9, 29.2.

HRMS (ESI) calculated for $C_{12}H_{14}NaO_4$ [M+Na$^+$]: 245.0784, found: 245.0775.

FT-IR (cm^{-1}): 3440, 2937, 2837, 1518, 1464, 1262, 1144, 1025, 910.

MS (EI) *m/z* (%): 222 (100), 139 (55), 167 (50), 163 (35), 147 (34), 107 (30), 178 (23), 191 (14), 195 (5).

The ee value was determined by the chiral UPC^2 analysis (CHIRALPAK® IC-3, CO_2/MeOH = 90/10, v = 2.0 mL/min, λ = 205 nm), t_b (minor) = 4.863 min, t_b (major) = 5.582 min, t_c (minor) = 11.517 min, t_c (major) = 11.734 min.

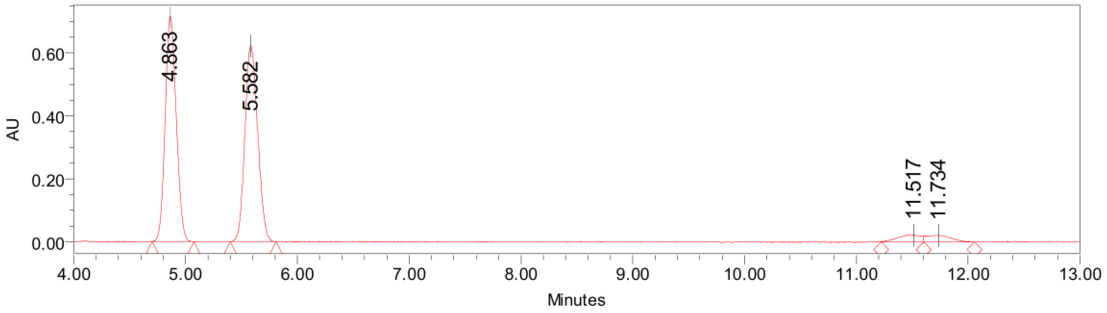

	RT	Area	% Area	Height
1	4.863	4912809	46.63	715127
2	5.582	4955771	47.04	621529
3	11.517	330020	3.13	22235
4	11.734	336508	3.19	21226

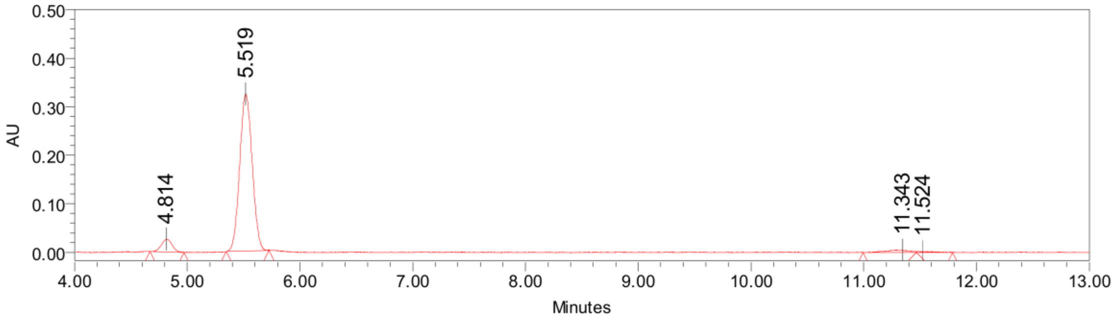

	RT	Area	% Area	Height
1	4.814	172898	6.32	26487
2	5.519	2480728	90.73	323910
3	11.343	66188	2.42	4594
4	11.524	14266	0.52	1713

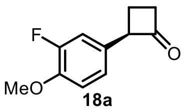

18a

(-)-2-(3-fluoro-4-methoxyphenyl)cyclobutan-1-one (18a)

Prepared according to **general procedure**: 38 h, 47% yield, 93.6% ee.

$[\alpha]_D^{27}$ = -1.0 (c = 1.0, $CHCl_3$)

The ee value was determined by the chiral UPC^2 analysis (CHIRALPAK® IG-3, CO_2/MeOH = 98/02, v = 2.0 mL/min, λ = 223.8 nm), t (major) = 2.725 min, t (minor) = 3.119 min.

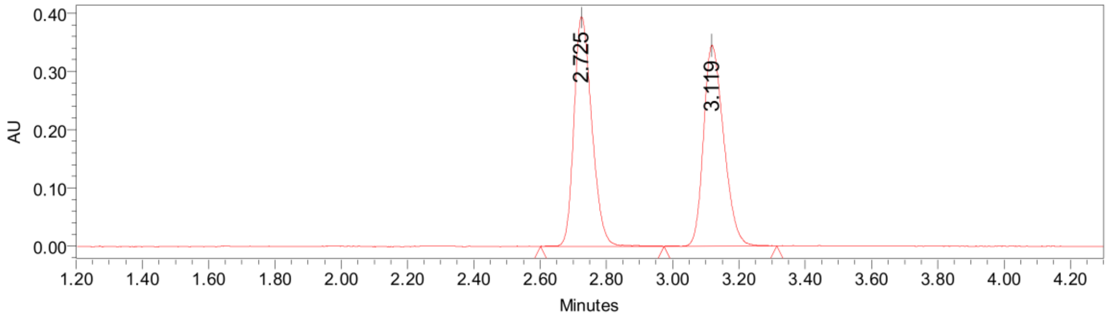

	RT	Area	% Area	Height
1	2.725	1475982	50.23	394249
2	3.119	1462340	49.77	344869

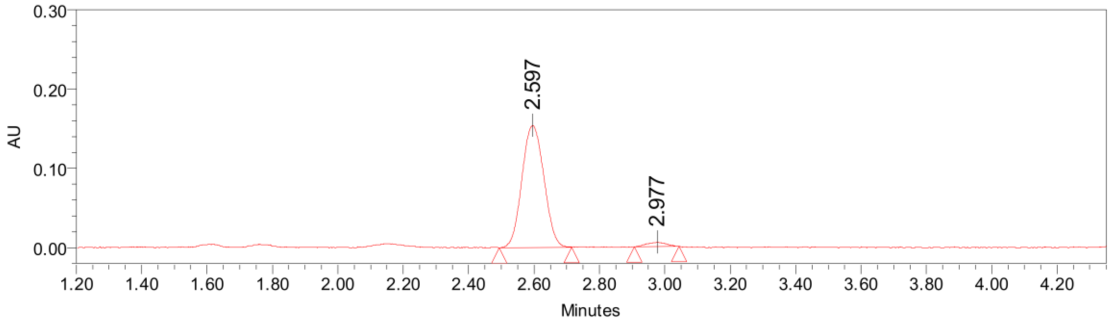

	RT	Area	% Area	Height
1	2.597	719066	96.79	154085
2	2.977	23884	3.21	5795

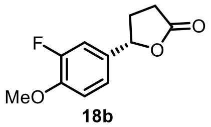

18b

(-)-5-(3-fluoro-4-methoxyphenyl)dihydrofuran-2(3H)-one (18b)

Prepared according to **general procedure**: 38 h, 49% yield, 89.4% ee, **18b**/**18c** = 12.5/1, yellow liquid.

$[\alpha]_D^{27}$ = -11.0 (c = 1.0, $CHCl_3$)

^{1}H NMR (400 MHz, $CDCl_3$) δ 7.12-7.03 (m, 2H), 7.00-6.92 (m, 1H), 5.49-5.39 (m, 1H), 3.90 (s, 3H), 2.71-2.57 (m, 3H), 2.27-2.10 (m, 1H).

^{13}C NMR (100 MHz, $CDCl_3$) δ 176.6, 152.3 (d, *J* = 245.2 Hz), 147.7 (d, *J* = 10.6 Hz), 132.1 (d, *J* = 5.9 Hz), 121.4 (d, *J* = 3.7 Hz), 113.4 (d, *J* = 19.2 Hz), 113.4 (d, *J* = 2.2 Hz), 80.4, 56.3, 30.8, 28.9.

^{19}F NMR (376 MHz, $CDCl_3$) δ -133.96.

HRMS (ESI) calculated for $C_{11}H_{11}NaO_3F$ [M+Na$^+$]: 233.0584, found: 233.0583.

FT-IR (cm^{-1}): 2938, 1773, 1522, 1278, 1180, 1142, 1025, 913, 758.

MS (EI) *m/z* (%): 139 (100), 84 (72), 57 (68), 69 (64), 111 (62), 85 (58), 125 (48), 155 (48), 169 (48), 210 (20).

The ee value was determined by the chiral UPC^2 analysis (CHIRALPAK® IG-3, CO_2/MeOH = 90/10, v = 2.0 mL/min, λ = 223.8 nm), t_b (major) = 2.779 min, t_b (minor) = 3.081 min, t_c (major) = 2.542 min, t_c (minor) = 2.623 min.

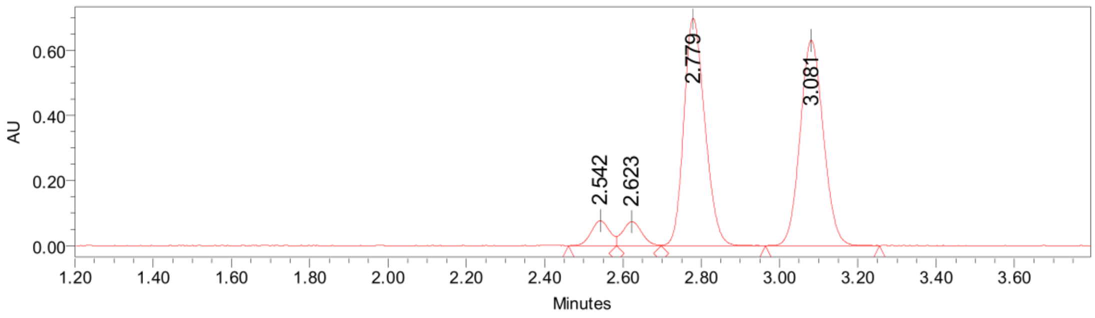

	RT	Area	% Area	Height
1	2.542	249753	4.38	76495
2	2.623	250094	4.38	73394
3	2.779	2603742	45.62	698461
4	3.081	2603692	45.62	630557

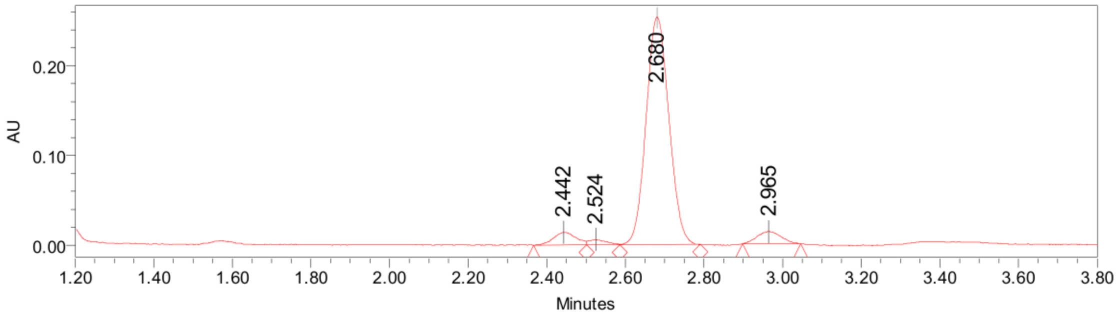

	RT	Area	% Area	Height
1	2.442	57228	4.95	14249
2	2.524	18134	1.57	5882
3	2.680	1023721	88.51	253473
4	2.965	57505	4.97	13607

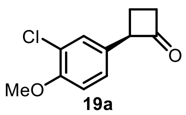

19a

(-)-2-(3-chloro-4-methoxyphenyl)cyclobutan-1-one (19a)

Prepared according to **general procedure**: 38 h, 46% yield, 96.6% ee.

$[\alpha]_D^{28}$ = -33.0 (c = 1.0, $CHCl_3$)

The ee value was determined by the chiral UPC^2 analysis (CHIRALPAK® IC-3, CO_2/MeOH = 95/05, v = 2.0 mL/min, λ = 205.0 nm), t (major) = 2.073 min, t (minor) = 2.271 min.

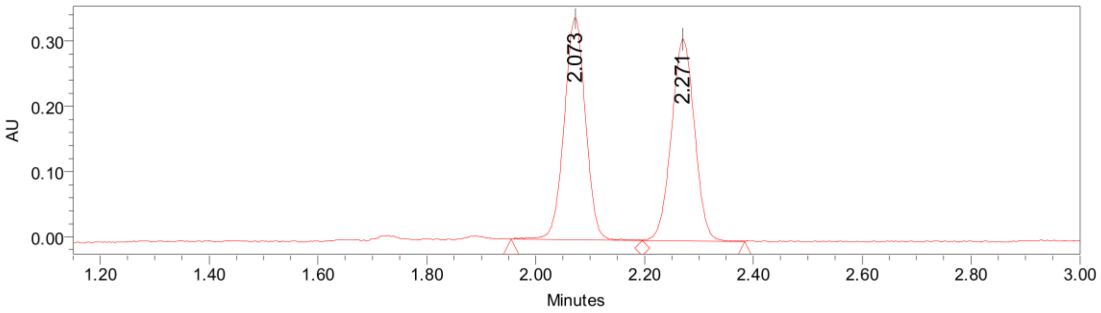

	RT	Area	% Area	Height
1	2.073	923974	50.43	340005
2	2.271	908369	49.57	308876

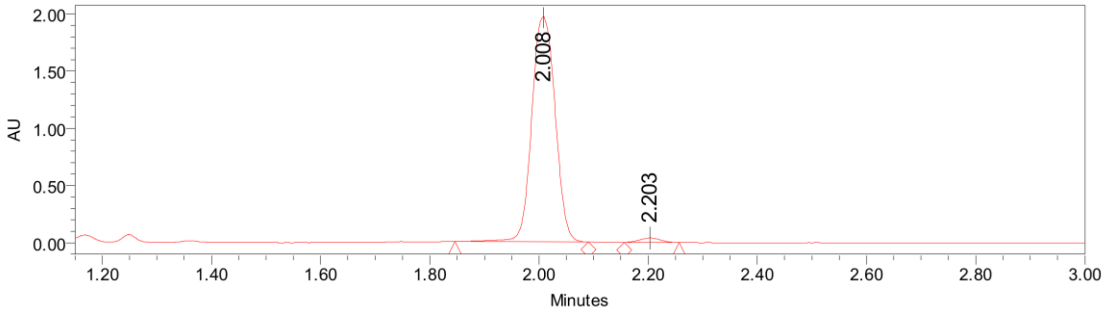

	RT	Area	% Area	Height
1	2.008	5864118	98.29	1968516
2	2.203	102140	1.71	38535

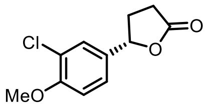

19b

(-)-5-(3-chloro-4-methoxyphenyl)dihydrofuran-2(3H)-one (19b)

Prepared according to **general procedure**: 38 h, 52% yield, 89.8% ee, **19b**/**19c** = 11.1/1, white solid, m. p.: 57-59 °C.

$[\alpha]_D^{28}$ = -28.0 (c = 1.0, $CHCl_3$)

^{1}H NMR (400 MHz, $CDCl_3$) δ 7.35 (d, *J* = 2.0 Hz, 1H), 7.21 (dd, *J* = 8.6, 2.2 Hz, 1H), 6.94 (d, *J* = 8.4 Hz, 1H), 5.47-5.37 (m, 1H), 3.91 (s, 3H), 2.75-2.56 (m, 3H), 2.25-2.10 (m, 1H).

^{13}C NMR (100 MHz, $CDCl_3$) δ 176.5, 155.0, 132.2, 127.4, 125.0, 122.7, 112.0, 80.3, 56.2, 30.8, 28.9.

HRMS (ESI) calculated for $C_{11}H_{12}O_3Cl$ [M+H$^+$]: 227.0469, found: 227.0469.

FT-IR (cm^{-1}): 2950, 1776, 1506, 1261, 1179, 1064, 1022.

MS (EI) *m/z* (%): 171 (100), 226 (60), 169 (58), 147 (46), 103 (40), 182 (30), 191 (18), 228 (18).

The ee value was determined by the chiral UPC^2 analysis (CHIRALPAK® IC-3, CO_2/MeOH = 95/05, v = 2.0 mL/min, λ = 205.0 nm), t_b (minor) = 6.266 min, t_b (major) = 6.643 min, t_c (major) = 8.362 min, t_c (minor) = 8.751 min.

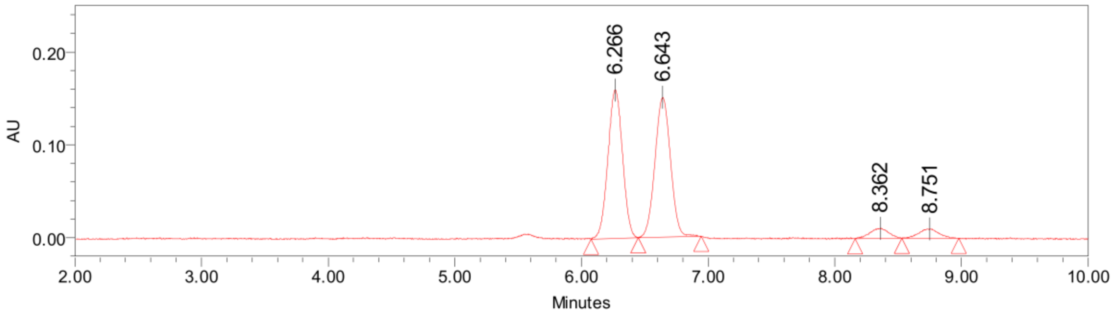

	RT	Area	% Area	Height
1	6.266	1281415	45.85	160027
2	6.643	1285213	45.99	150898
3	8.362	114981	4.11	11084
4	8.751	113001	4.04	10163

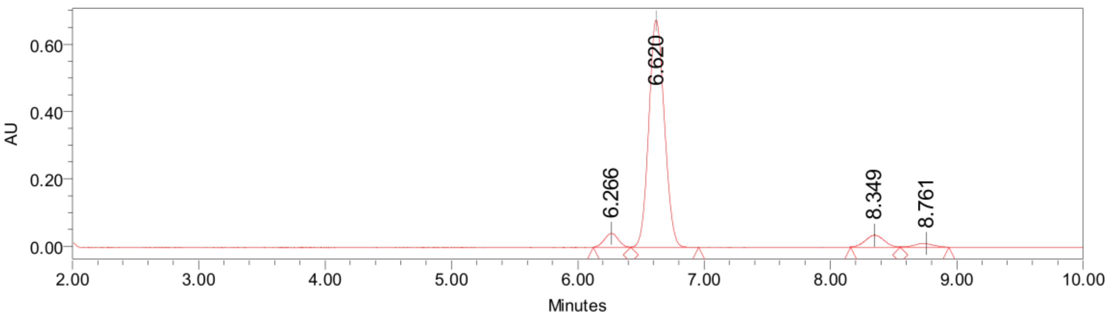

	RT	Area	% Area	Height
1	6.266	312281	4.75	41050
2	6.620	5778956	87.97	675765
3	8.349	363948	5.54	35503
4	8.761	114085	1.74	10788

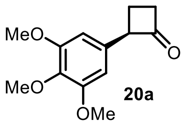

20a

(-)-2-(3,4,5-trimethoxyphenyl)cyclobutan-1-one (20a)

Prepared according to **general procedure**: 48 h, 48% yield, 90.5% ee.

$[\alpha]_D^{30}$ = -7.0 (c = 1.0, $CHCl_3$)

The ee value was determined by the chiral UPC^2 analysis (CHIRALPAK® IC-3, CO_2/MeOH = 90/10, v = 2.0 mL/min, λ = 205.0 nm), t (major) = 2.560 min, t (minor) = 3.770 min.

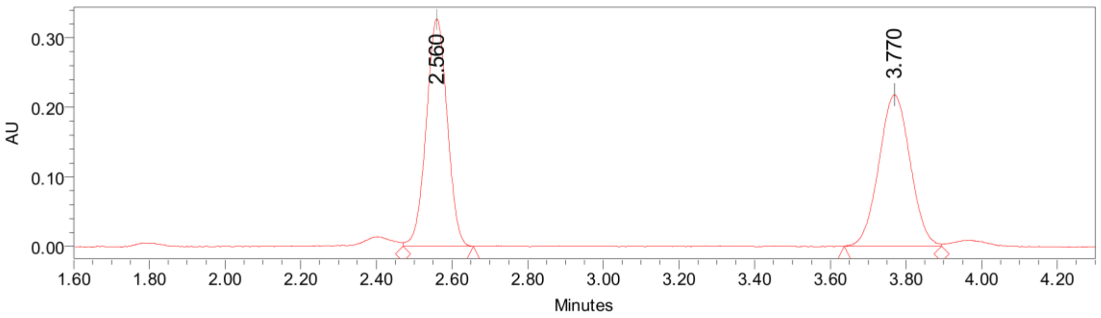

	RT	Area	% Area	Height
1	2.560	1261558	50.07	327123
2	3.770	1257781	49.93	218327

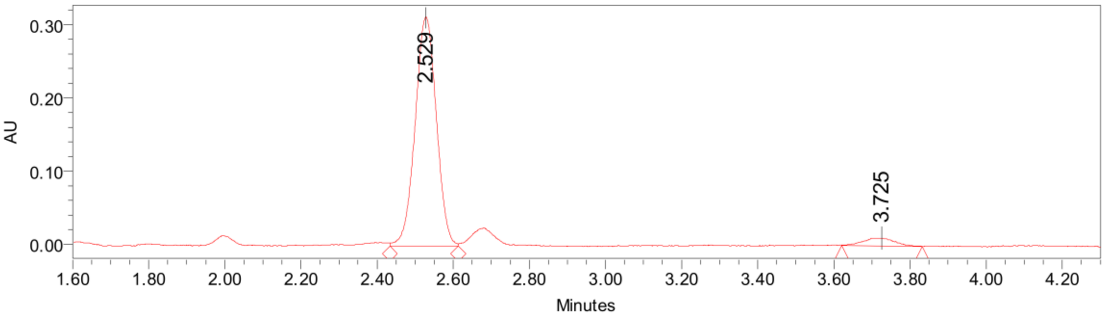

	RT	Area	% Area	Height
1	2.529	1209777	95.26	312752
2	3.725	60210	4.74	10644

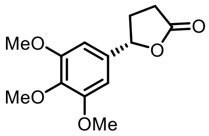

20b

(-)-5-(3,4,5-trimethoxyphenyl)dihydrofuran-2(3H)-one (20b)

Prepared according to **general procedure**: 48 h, 48% yield, 84.8% ee, **20b**/**20c** = >20/1, colorless liquid.

$[\alpha]_D^{30}$ = -7.0 (c = 1.0, $CHCl_3$)

^{1}H NMR (600 MHz, $CDCl_3$) δ 6.54 (s, 2H), 5.45 (dd, *J* = 8.4, 6.0 Hz, 1H), 3.87 (s, 6H), 3.85 (s, 3H), 2.70-2.62 (m, 3H), 2.24-2.14 (m, 1H).

^{13}C NMR (150 MHz, $CDCl_3$) δ 176.8, 153.5, 137.9, 135.0, 102.1, 81.2, 60.8, 56.2, 31.1, 29.0.

HRMS (ESI) calculated for $C_{13}H_{16}NaO_5$ [M+Na$^+$]: 275.0890, found: 275.0883.

FT-IR (cm^{-1}): 2941, 1773, 1593, 1464, 1240, 1179, 1126.

MS (EI) *m/z* (%): 252 (100), 193 (36), 177 (20), 169 (18), 237 (15), 154 (10), 138 (10), 125 (10), 105 (10).

The ee value was determined by the chiral UPC^2 analysis (CHIRALPAK® IC-3, CO_2/MeOH = 90/10, v = 2.0 mL/min, λ = 205 nm), t_b (minor) = 4.531 min, t_b (major) = 5.426 min, t_c (major) = 6.924 min, t_c (minor) = 7.688 min.

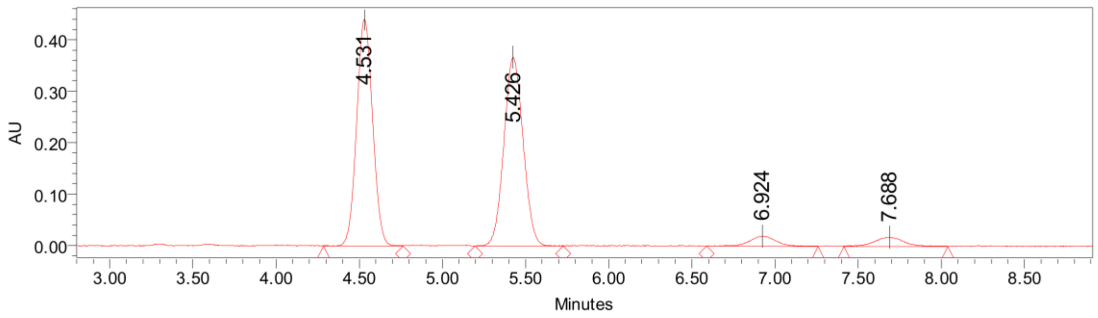

	RT	Area	% Area	Height
1	4.531	2942762	46.51	440422
2	5.426	2948368	46.59	366656
3	6.924	222327	3.51	19919
4	7.688	214325	3.39	17819

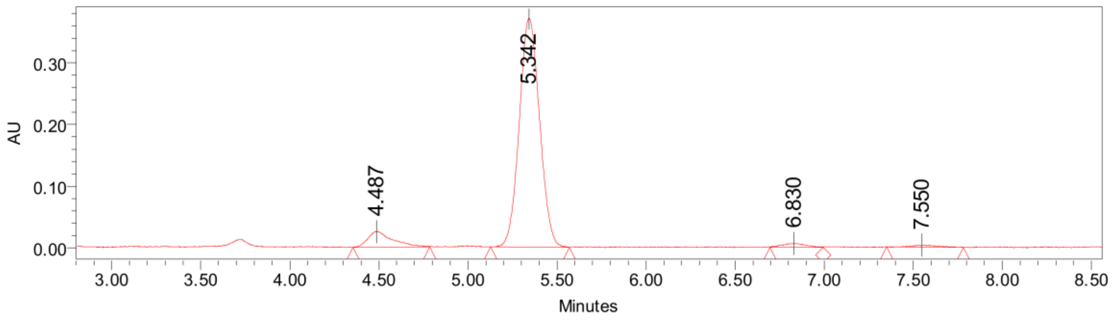

	RT	Area	% Area	Height
1	4.487	242701	7.39	25396
2	5.342	2954978	89.98	371922
3	6.830	54841	1.67	6329
4	7.550	31422	0.96	3076

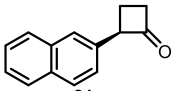

21a

(-)-2-(naphthalen-2-yl)cyclobutan-1-one (21a)

Prepared according to **indicated procedure c**: 42 h, 48% yield, 91.0% ee.

[α]$_D$^{23} = - 12.0 (c = 1.0, $CHCl_3$)

The ee value was determined by the chiral UPC^2 analysis (CHIRALPAK® IG-3, CO_2/MeOH = 95/05, v = 2.0 mL/min, λ = 220.0 nm), t (major) = 3.180 min, t (minor) = 3.843 min.

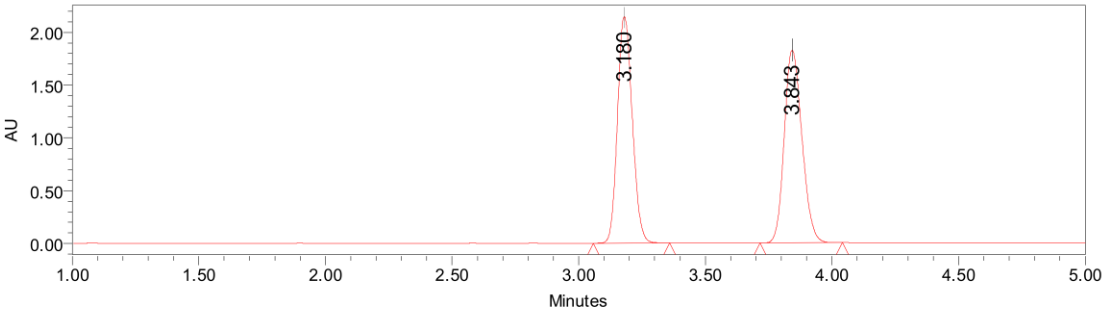

	RT	Area	% Area	Height
1	3.180	8707652	49.56	2144050
2	3.843	8861954	50.44	1824480

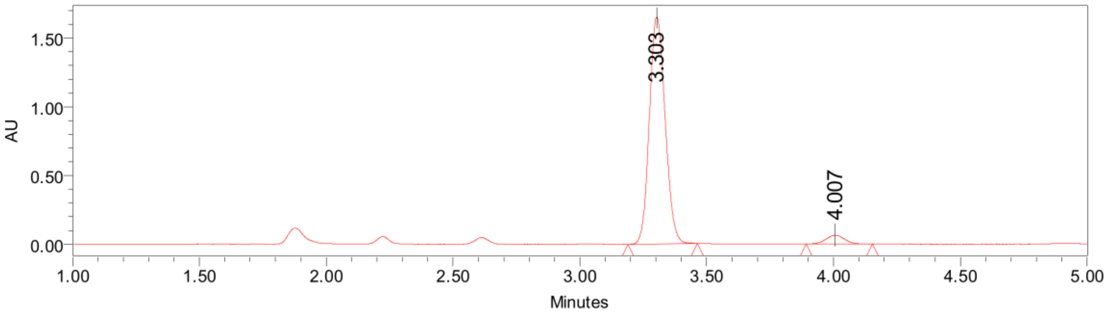

	RT	Area	% Area	Height
1	3.303	7118425	95.52	1650716
2	4.007	334052	4.48	63914

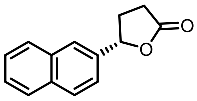

21b

(*S*)-5-(naphthalen-2-yl)dihydrofuran-2(3H)-one (21b)

Prepared according to **indicated procedure c**: 42 h, 51% yield, 91.8% ee, **21b**/**21c** = >20/1, white solid, m. p.: 115-117 °C.

[α]$_D$^{24} = - 18.0 (c = 1.0, $CHCl_3$)

^{1}H NMR (400 MHz, $CDCl_3$) δ 7.93-7.76 (m, 4H), 7.55-7.46 (m, 2H), 7.40 (dd, *J* = 8.4, 1.6 Hz, 1H), 5.74-5.61 (m, 1H), 2.78-2.63 (m, 3H), 2.35-2.18 (m, 1H).

^{13}C NMR (100 MHz, $CDCl_3$) δ 176.9, 136.6, 133.1, 133.0, 128.8, 128.0, 127.7, 126.6, 126.4, 124.2, 122.8, 81.2, 30.9, 28.9.

HRMS (ESI) calculated for $C_{14}H_{12}NaO_2$ [M+Na$^+$]: 235.0730, found: 235.0722.

FT-IR (cm^{-1}): 3420, 2951, 1768, 1191, 1013, 933, 755, 491.

MS (EI) *m/z* (%): 212 (100), 128 (65), 157 (54), 167 (30), 141 (12), 115 (12), 183 (5).

The ee value was determined by the chiral UPC^2 analysis (CHIRALPAK®OD-3, CO_2/MeOH = 95/05, v = 2.0 mL/min, λ = 220 nm), t_b (minor) = 3.502 min, t_b (major) = 3.813 min, t_c (major) = 4.799 min, t_c (minor) = 5.501 min.

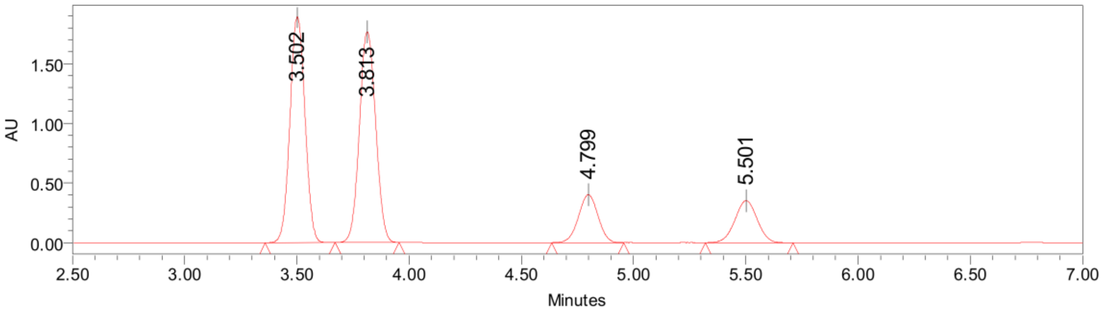

	RT	Area	% Area	Height
1	3.502	8804609	39.07	1890894
2	3.813	8875516	39.38	1764657
3	4.799	2421633	10.74	402007
4	5.501	2436155	10.81	352052

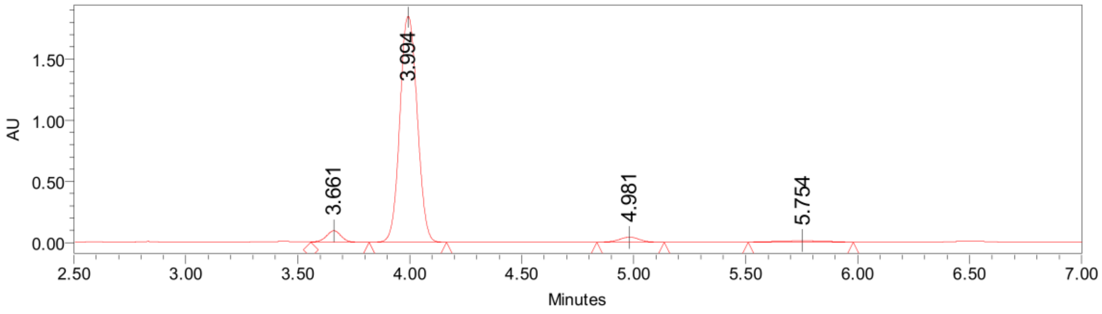

	RT	Area	% Area	Height
1	3.661	423080	3.92	92584
2	3.994	9950222	92.28	1846356
3	4.981	250129	2.32	40678
4	5.754	159719	1.48	12366

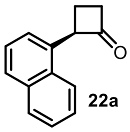

22a

(+)-2-(naphthalen-1-yl)cyclobutan-1-one (22a)

Prepared according to **general procedure**: 38 h, 41% yield, 97.8% ee.

[α]D^{25} = +48.0 (c = 1.0, $CHCl_3$)

The ee value was determined by the chiral UPC^2 analysis (CHIRALPAK® IG-3, CO_2/MeOH = 95/05, v = 2.0 mL/min, λ = 217.0 nm), t (major) = 2.586 min, t (minor) = 2.832 min.

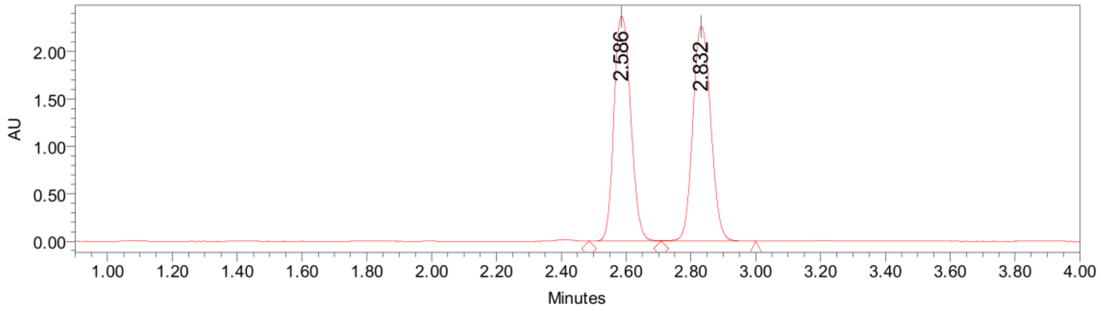

	RT	Area	% Area	Height
1	2.586	8510333	49.46	2367128
2	2.832	8697014	50.54	2260464

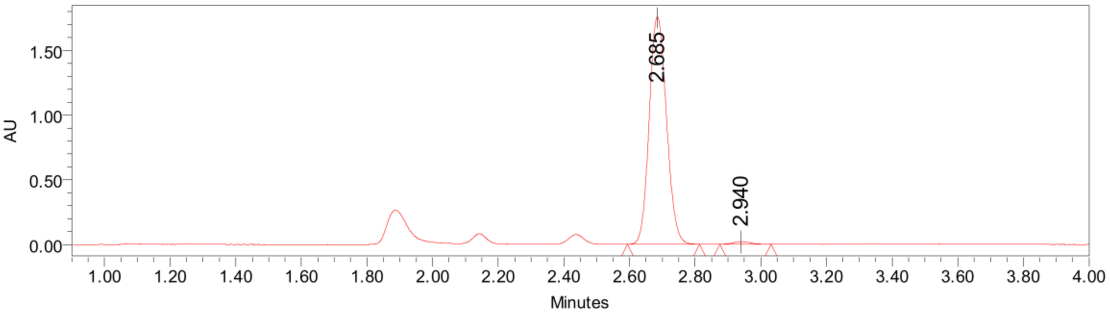

	RT	Area	% Area	Height
1	2.685	6424718	98.89	1757725
2	2.940	71966	1.11	19249

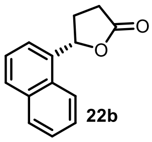

(-)-5-(naphthalen-1-yl)dihydrofuran-2(3H)-one (22b)

Prepared according to **general procedure**: 38 h, 56% yield, 94.4% ee (**c**: 95.3% ee), **22b**/**22c** = 6.0/1, orange liquid.

$[\alpha]_D^{26}$ = -110.0 (c = 1.0, $CHCl_3$)

^{1}H NMR (400 MHz, $CDCl_3$) δ 7.96-7.88 (m, 1H), 7.87-7.80 (m, 2H), 7.61-7.52 (m, 3H), 7.52-7.44 (m, 1H), 6.26 (t, *J* = 7.0 Hz, 1H), 2.96-2.83 (m, 1H), 2.78-2.58 (m, 2H), 2.35-2.23 (m, 1H).

^{13}C NMR (100 MHz, $CDCl_3$) δ 177.1, 134.9, 133.7, 129.4, 129.1, 128.7, 126.5, 125.9, 125.3, 122.4, 121.5, 78.6, 29.9, 28.3.

HRMS (ESI) calculated for $C_{14}H_{12}NaO_2$ [M+Na$^+$]: 235.0730, found: 235.0726.

FT-IR (cm^{-1}): 3426, 2925, 1776, 1511, 1184, 1162, 930, 779.

MS (EI) *m/z* (%): 128 (100), 212 (92), 157 (54), 153 (30), 167 (20), 115 (15), 141 (10).

The ee value was determined by the chiral UPC^2 analysis (CHIRALPAK® IC-3, CO_2/MeOH = 95/05, v = 2.0 mL/min, λ = 219 nm), t_b (minor) = 5.536 min, t_b (major) = 7.857 min, t_c (major) = 9.693 min, t_c (minor) = 10.255 min.

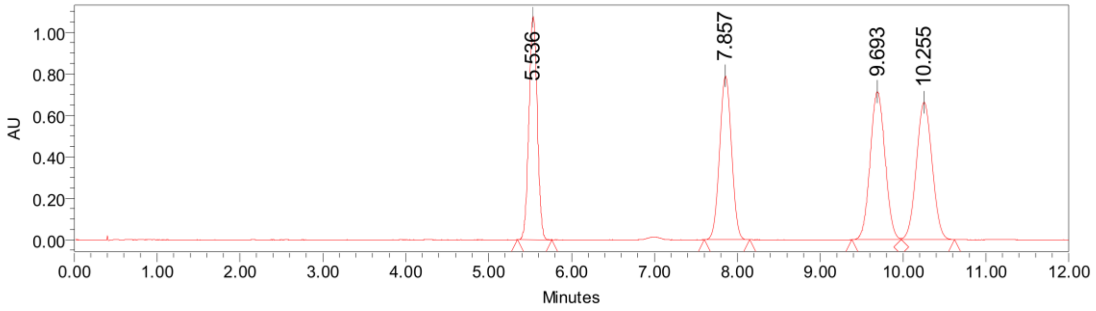

	RT	Area	% Area	Height
1	5.536	7539214	22.82	1077212
2	7.857	7885523	23.87	789656
3	9.693	8890567	26.91	714665
4	10.255	8723493	26.40	662116

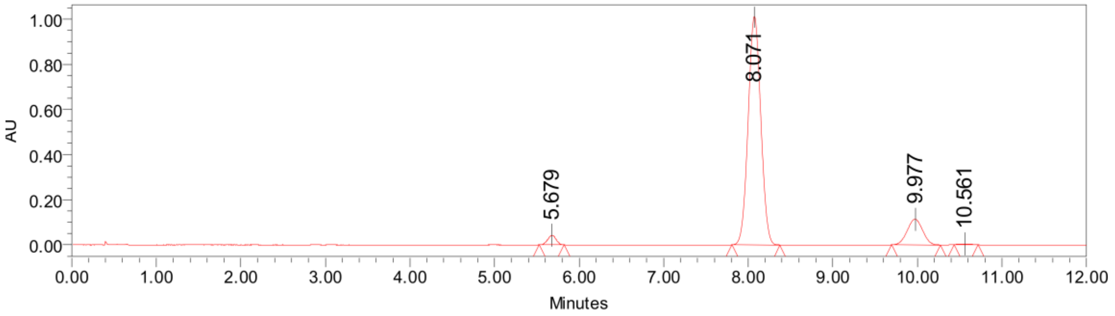

	RT	Area	% Area	Height
1	5.679	299946	2.44	42655
2	8.071	10498981	85.52	1012827
3	9.977	1442499	11.75	113721
4	10.561	34578	0.28	3607

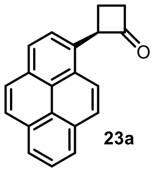

23a

(-)-2-(pyren-1-yl)cyclobutan-1-one (23a)

Prepared according to **indicated procedure c**: 42 h, 44% yield, 96.5% ee.

$[\alpha]_D^{30}$ = -42.0 (c = 1.0, $CHCl_3$)

The ee value was determined by the chiral UPC^2 analysis (CHIRALPAK® IC-3, CO_2/MeOH = 80/20, v = 2.0 mL/min, λ = 240.0 nm), t (major) = 4.539 min, t (minor) = 6.121 min.

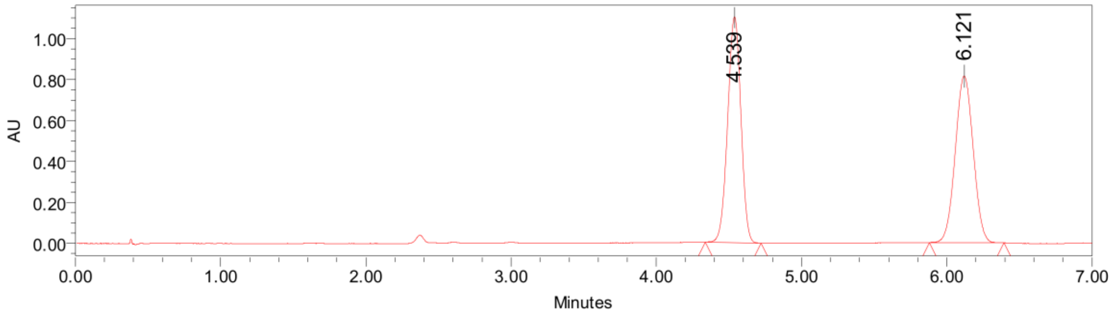

	RT	Area	% Area	Height
1	4.539	7007808	49.99	1102414
2	6.121	7010802	50.01	814628

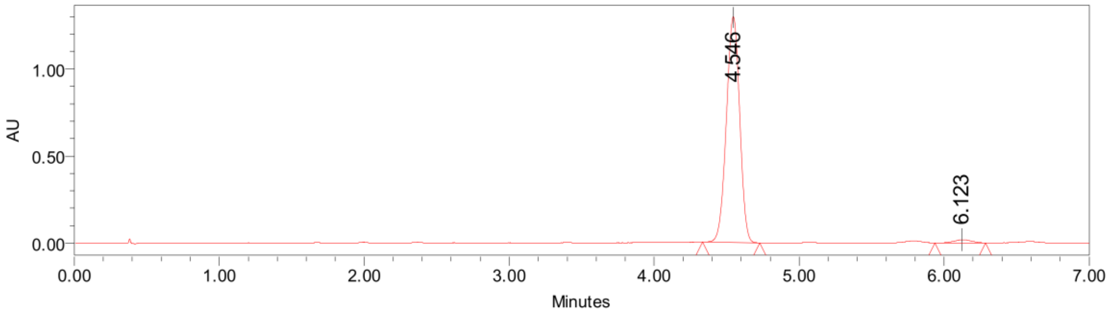

	RT	Area	% Area	Height
1	4.546	8295588	98.28	1297292
2	6.123	144843	1.72	17328

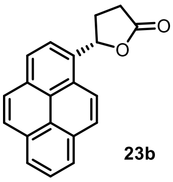

23b

(-)-5-(pyren-2-yl)dihydrofuran-2(3H)-one (23b)

Prepared according to **indicated procedure c**: 42 h, 55% yield, 88.6% ee, **23b/23c** = >20/1, white solid, m. p.: 142-144 °C.

[α]$_D$^{30} = -131.0 (c = 1.0, $CHCl_3$)

1H NMR (400 MHz, $CDCl_3$) δ 8.26-8.12 (m, 4H), 8.12-7.98 (m, 5H), 6.55 (t, J = 7.2 Hz, 1H), 3.06-2.92 (m, 1H), 2.86-2.68 (m, 2H), 2.46-2.30 (m, 1H).

^{13}C NMR (100 MHz, $CDCl_3$) δ 177.2, 132.4, 131.3, 131.2, 130.4, 128.3, 127.7, 127.4, 127.0, 126.2, 125.7, 125.4, 125.0, 124.8, 124.6, 121.7, 121.5, 79.0, 30.9, 28.7.

HRMS (ESI) calculated for $C_{20}H_{14}NaO_2$ [M+Na$^+$]: 309.0886, found: 309.0874.

FT-IR (cm^{-1}): 3042, 2953, 1774, 1182, 1160, 844, 717.

MS (EI) *m/z* (%): 286 (100), 202 (64), 241 (48), 227 (30), 120 (25), 100 (25), 214 (14), 254 (5).

The ee value was determined by the chiral UPC^2 analysis (CHIRALPAK® IC-3, CO_2/MeOH = 80/20, v = 2.0 mL/min, λ = 240.0 nm), t_b (minor) = 6.940 min, t_b (major) = 9.071 min, t_c = 5.571 min, t_c = 9.071 min.

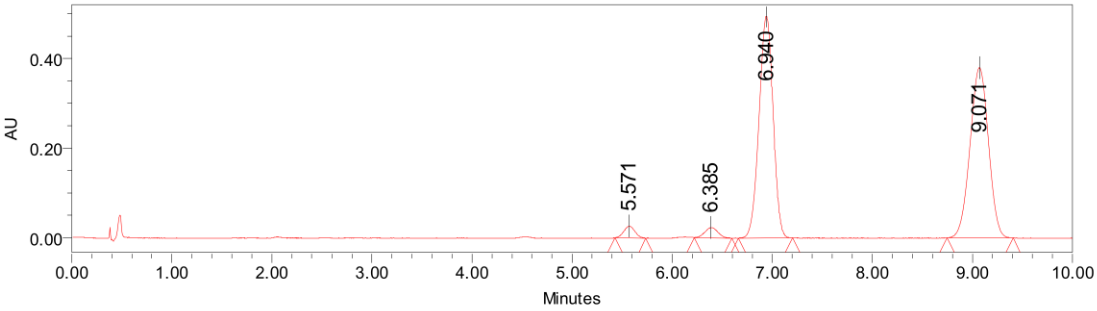

	RT	Area	% Area	Height
1	5.571	205391	2.03	26390
2	6.385	207272	2.05	23091
3	6.940	4848549	47.87	496089
4	9.071	4868298	48.06	380744

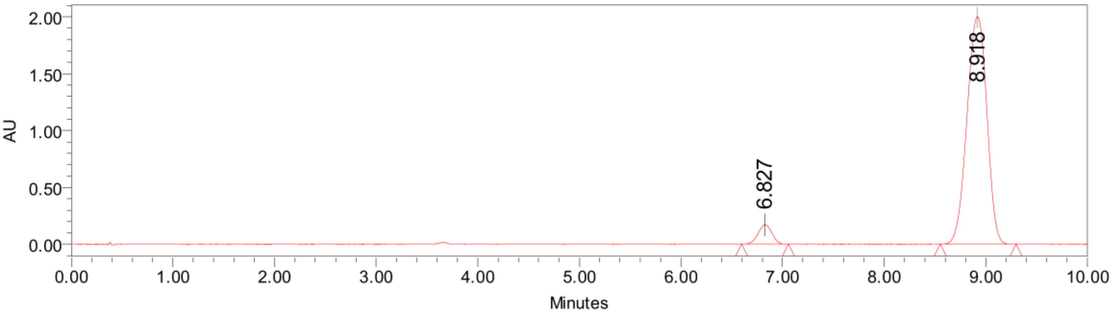

	RT	Area	% Area	Height
1	6.827	1640006	5.69	172203
2	8.918	27177013	94.31	2002102

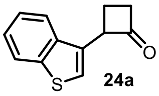

24a

(+)-2-(benzo[b]thiophen-3-yl)cyclobutan-1-one (24a)

Prepared according to **indicated procedure b**: 48 h, 40% yield, 83.7% ee.

[α]$_D$^{25} = +37.0 (c = 1.0, $CHCl_3$)

The ee value was determined by the chiral UPC^2 analysis (CHIRALPAK® IC-3, CO_2/MeOH = 95/05, v = 2.0 mL/min, λ = 226.0 nm), t (major) = 2.252 min, t (minor) = 2.570min.

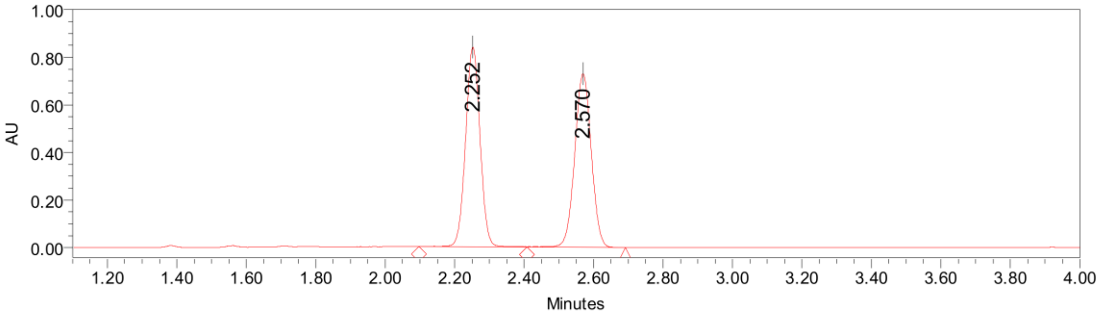

	RT	Area	% Area	Height
1	2.252	2426113	50.00	839209
2	2.570	2426111	50.00	729770

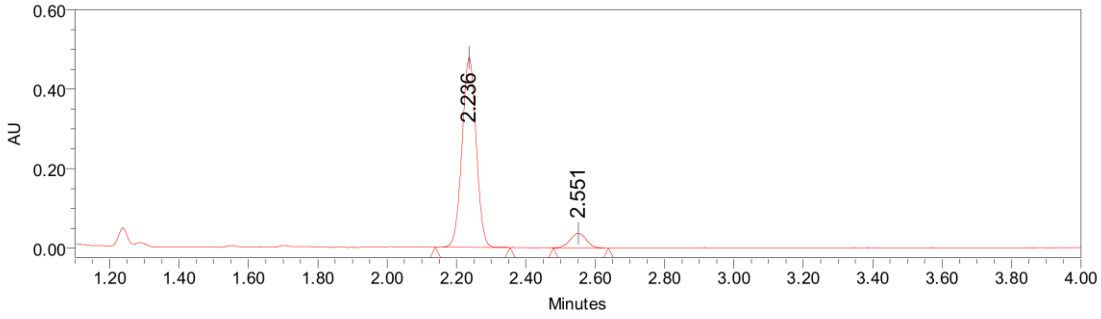

	RT	Area	% Area	Height
1	2.236	1348137	91.84	477126
2	2.551	119789	8.16	36792

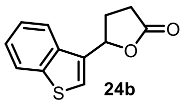

24b

(-)-5-(benzo[b]thiophen-3-yl)dihydrofuran-2(3H)-one (24b)

Prepared according to **indicated procedure b**: 48 h, 58% yield, 90.1% ee (**d**: 84.5% ee), **24b**/**24c** = 4.0/1, orange solid, m. p.: 89-91 °C.

$[\alpha]_D^{25}$ = -27.0 (c = 1.0, $CHCl_3$)

^{1}H NMR (600 MHz, $CDCl_3$) δ 7.88 (dd, *J* = 6.6, 1.8 Hz, 1H), 7.73 (dd, *J* = 6.9, 2.1 Hz, 1H), 7.33-7.48 (m, 3H), 5.85 (t, *J* = 7.2 Hz, 1H), 2.88-2.62 (m, 3H), 2.46-2.36 (m, 1H).

^{13}C NMR (150 MHz, $CDCl_3$) δ 176.6, 140.9, 136.4, 133.9, 124.8, 124.4, 123.0, 122.8, 121.7, 28.4, 28.3.

HRMS (ESI) calculated for $C_{12}H_{10}NaO_2S$ $[M+Na^+]$: 242.0294, found: 242.0295.

FT-IR (cm-1) 3081, 2961, 1773, 1704, 1429, 1184, 1020, 919, 747, 733, 421.

MS (EI) *m/z* (%): 218 (100), 168 (80), 173 (66), 147 (60), 134 (50), 115 (28).

The ee value was determined by the chiral UPC^2 analysis (CHIRALPAK® IC-3, CO_2/MeOH = 95/05, v = 2.0 mL/min, λ = 226.0 nm), t_b (minor) = 6.658 min, t_b (major) = 6.309 min, t_c (major) = 9.088 min, t_c (minor) = 10.135 min.

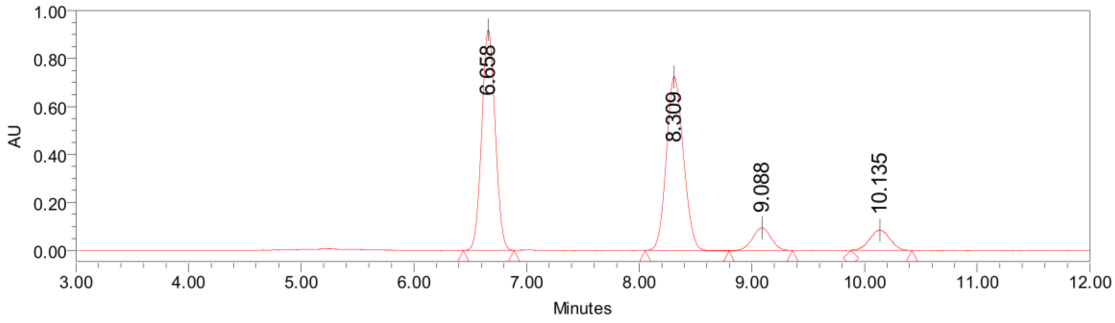

	RT	Area	% Area	Height
1	6.658	7601706	43.69	920396
2	8.309	7636650	43.89	724852
3	9.088	1087447	6.25	94508
4	10.135	1074740	6.18	85258

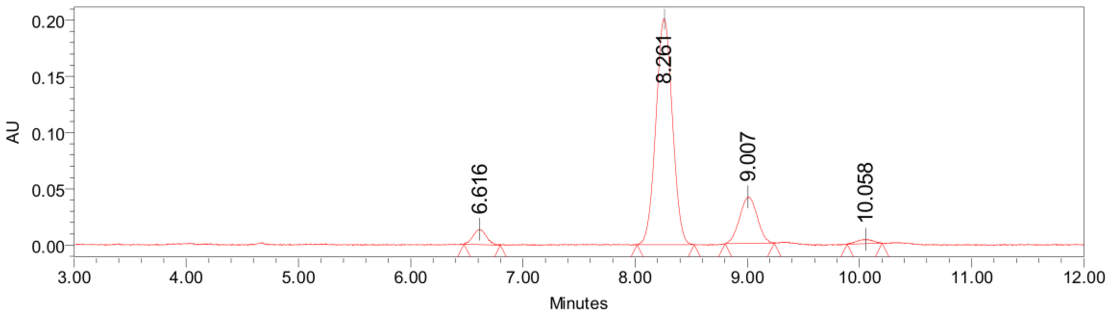

	RT	Area	% Area	Height
1	6.616	107863	4.06	13380
2	8.261	2062542	77.59	201349
3	9.007	449850	16.92	41381
4	10.058	37833	1.42	3909

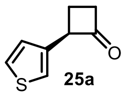

25a

(-)-2-(thiophen-3-yl)cyclobutan-1-one (25a)

Prepared according to **indicated procedure b**: 36 h, 45% yield, 90.4% ee.

$[\alpha]_D^{26}$ = - 13.0 (c = 1.0, $CHCl_3$)

The ee value was determined by the chiral UPC^2 analysis (CHIRALPAK® IG-3, CO_2/MeOH = 98/02, v = 2.0 mL/min, λ = 234.0 nm), t (major) = 1.959 min, t (minor) = 2.294 min.

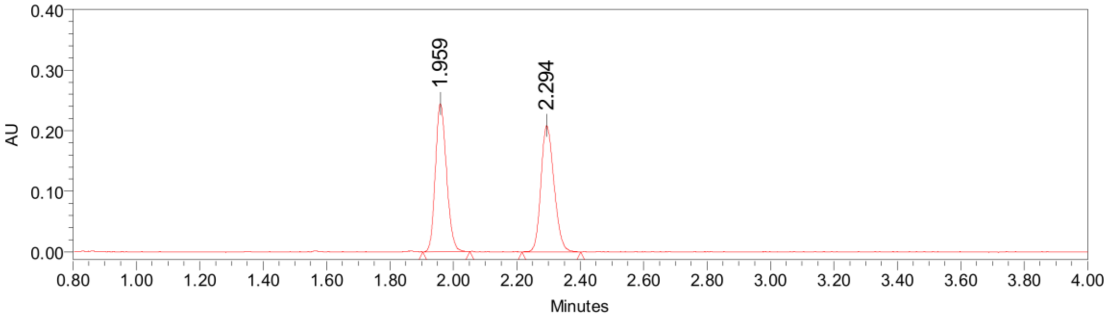

	RT	Area	% Area	Height
1	1.959	596113	49.86	244547
2	2.294	599474	50.14	208613

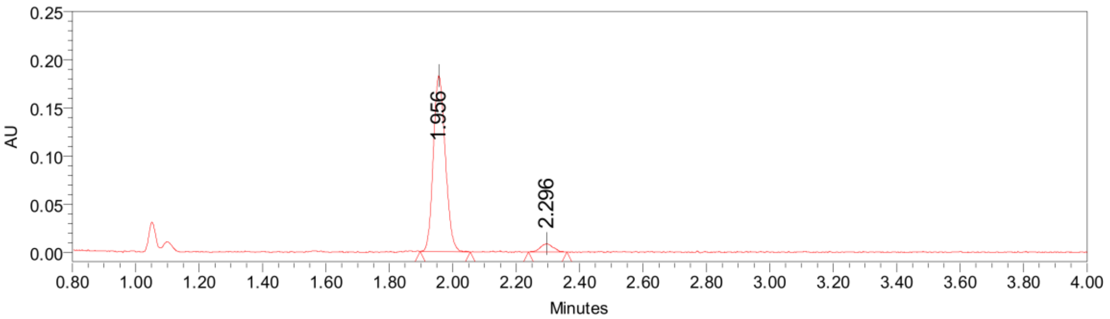

	RT	Area	% Area	Height
1	1.956	479104	95.26	182669
2	2.296	23863	4.74	8531

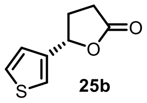

25b

(+)-5-(thiophen-3-yl)dihydrofuran-2(3H)-one (25b)

Prepared according to **indicated procedure b**: 36 h, 51% yield, 89.8% ee, **25b**/**25c** = 17.1/1, orange liquid.

$[\alpha]_D^{26}$ = +5.0 (c = 1.0, $CHCl_3$)

^{1}H NMR (600 MHz, CDCl3) δ 7.37 (dd, *J* = 5.4, 3.0 Hz, 1H), 7.28 (d, *J* = 3.0 Hz, 1H), 7.07 (dd, *J* = 5.1, 1.5 Hz, 1H), 5.59 (t, *J* = 6.6 Hz, 1H), 2.71-2.59 (m, 3H), 2.32-2.21 (m, 1H).

^{13}C NMR (150 MHz, $CDCl_3$) δ 176.7, 140.3, 127.0, 125.1, 121.9, 78.0, 29.9, 28.7.

HRMS (ESI) calculated for $C_8H_8NaO_2S$ [M+Na$^+$]: 191.0137, found: 191.0138.

FT-IR (cm-1) 3101, 2958, 1771, 1709, 1168, 1159, 911, 789, 747, 678.

MS (EI) *m/z* (%): 139 (100), 111 (98), 105 (66), 156 (50), 168 (20).

The ee value was determined by the chiral UPC^2 analysis (CHIRALPAK® IG-3, CO_2/MeOH = 95/05, v = 2.0 mL/min, λ = 226.0 nm), t_b (major) = 4.560 min, t_b (minor) = 4.882 min.

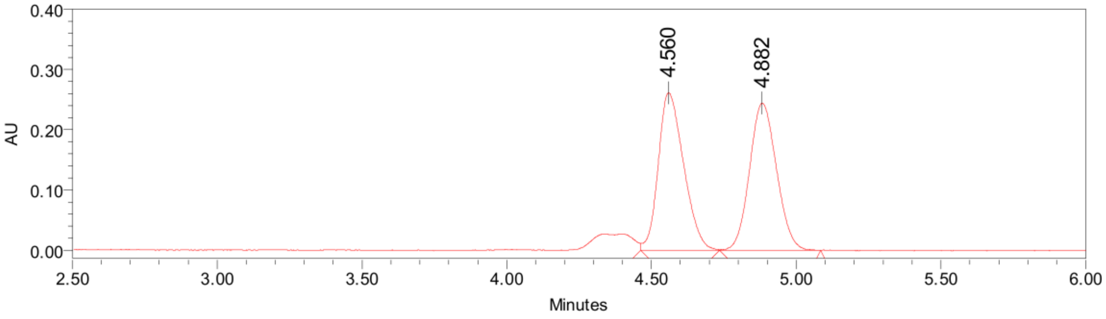

	RT	Area	% Area	Height
1	4.560	1594903	50.05	261583
2	4.882	1591683	49.95	244763

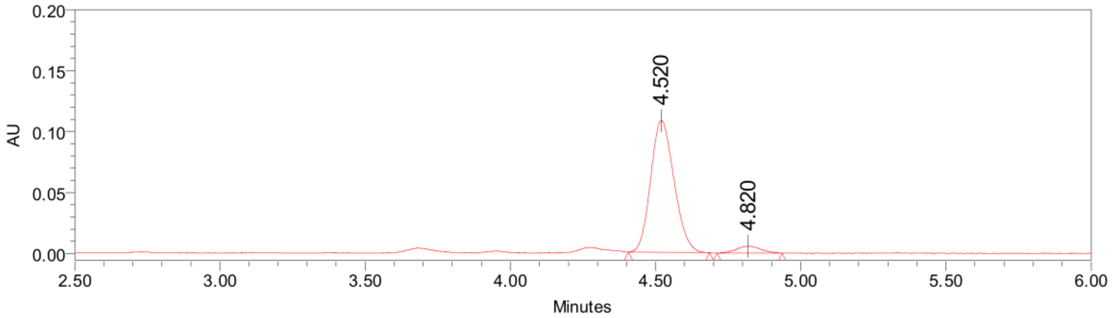

	RT	Area	% Area	Height
1	4.520	604638	94.91	108150
2	4.820	32417	5.09	5657

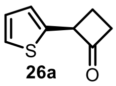

26a

(-)-2-(thiophen-2-yl)cyclobutan-1-one (26a)

Prepared according to **general procedure**: 48 h, 40% yield, 98.5% ee.

[α]$_D$^{26} = -15.0 (c = 1.0, $CHCl_3$)

The ee value was determined by the chiral UPC^2 analysis (CHIRALPAK® IG-3, CO_2/MeOH = 98/02, v = 2.0 mL/min, λ = 235.6 nm), t (major) = 1.766 min, t (minor) = 1.884 min.

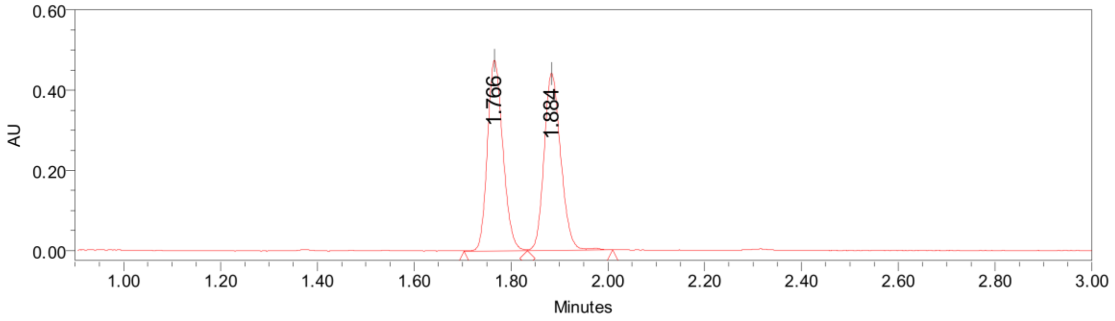

	RT	Area	% Area	Height
1	1.766	1047346	49.84	474513
2	1.884	1053899	50.16	441150

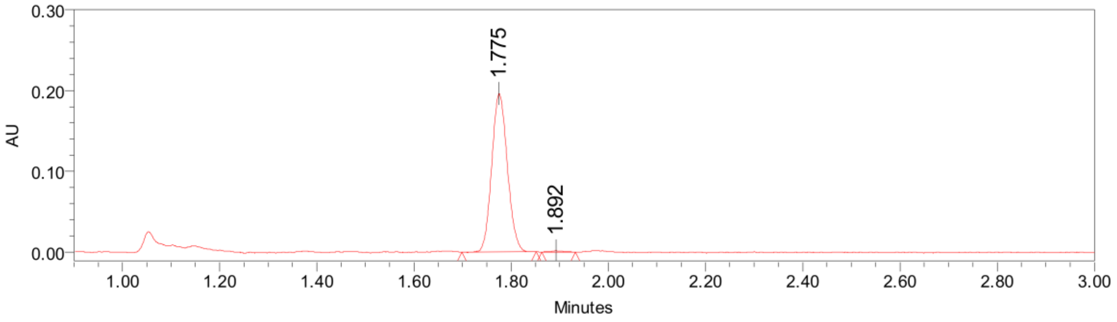

	RT	Area	% Area	Height
1	1.775	437402	99.27	196029
2	1.892	3237	0.73	1645

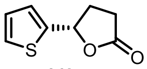

26b

(+)-5-(furan-2-yl)dihydrofuran-2(3H)-one (26b)

Prepared according to **general procedure**: 48 h, 54% yield, 85.9% ee, **26b**/**26c** = 12.0/1, yellow liquid.

[α]$_D$^{26} = +21.0 (c = 1.0, $CHCl_3$)

^{1}H NMR (600 MHz, $CDCl_3$) δ 7.34 (d, *J* = 5.4 Hz, 1H), 7.09 (d, *J* = 3.6 Hz, 1H), 7.01 (dd, *J* = 5.1, 3.9 Hz, 1H), 5.80-5.70 (m, 1H), 2.75-2.61 (m, 3H), 2.43-2.33 (m, 1H).

^{13}C NMR (150 MHz, $CDCl_3$) δ 176.1, 141.7, 126.9, 126.2, 125.9, 77.3, 30.7, 28.9.

HRMS (ESI) calculated for $C_8H_9O_2$ [M+H$^+$]: 169.0318, found: 169.0320.

FT-IR (cm-1) 3907, 2960, 1777, 1706, 1297, 1172, 1014, 920, 718.

MS (EI) *m/z* (%): 111 (100), 139 (78), 105 (48), 156 (40), 168 (20).

The ee value was determined by the chiral UPC^2 analysis (CHIRALPAK® IC-3, CO_2/MeOH = 95/05, v = 2.0 mL/min, λ = 230.0 nm), t$_b$ (minor) = 3.023 min, t$_b$ (major) = 3.269 min.

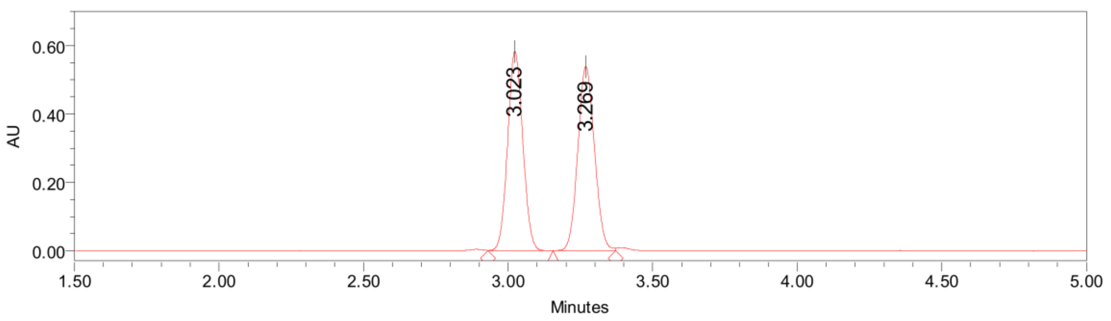

	RT	Area	% Area	Height
1	3.023	2133282	49.63	583109
2	3.269	2164971	50.37	538949

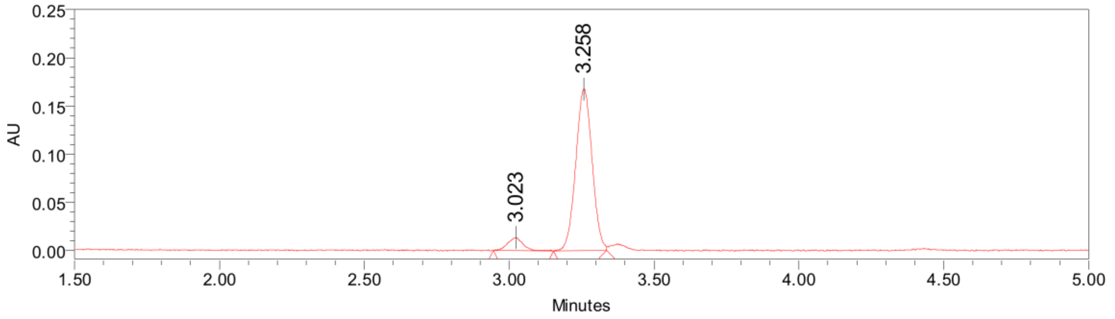

	RT	Area	% Area	Height
1	3.023	50184	7.06	13563
2	3.258	660307	92.94	167650

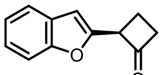

27a

(+)-2-(benzofuran-2-yl)cyclobutan-1-one (27a)

Prepared according to **general procedure**: 36 h, 42% yield, 95.7% ee.

[α]$_D$^{25} = +2.0 (c = 1.0, $CHCl_3$)

The ee value was determined by the chiral UPC^2 analysis (CHIRALPAK® OD-3, CO_2/MeOH = 98/02, v = 2.0 mL/min, λ = 246.0 nm), t (major) = 1.688 min, t (minor) = 2.011 min.

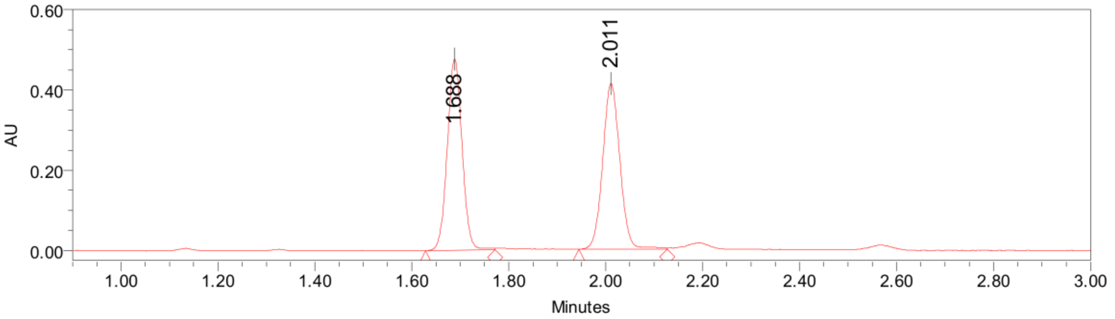

	RT	Area	% Area	Height
1	1.688	1006897	49.70	476279
2	2.011	1018911	50.30	412773

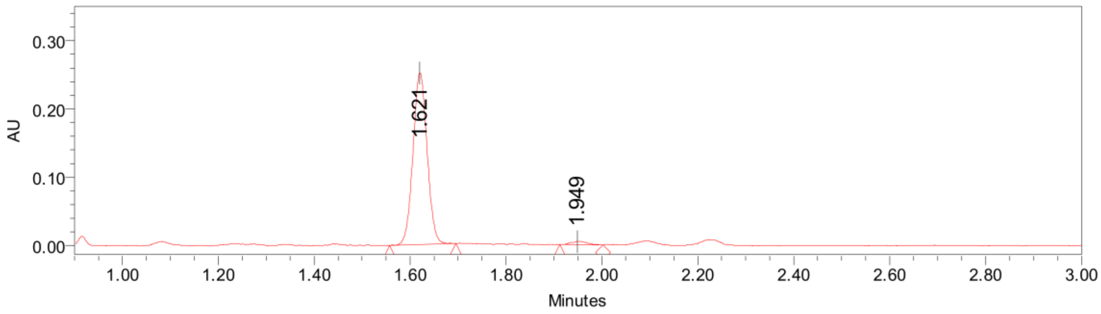

	RT	Area	% Area	Height
1	1.621	513675	97.85	251449
2	1.949	11308	2.15	4711

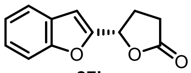

27b

(+)-5-(benzofuran-2-yl)dihydrofuran-2(3H)-one (27b)

Prepared according to **general procedure**: 48 h, 53% yield, 81.5% ee, **27b**/**27c** = 10.0/1, yellow solid, m. p.: 44-46 °C.

$[\alpha]_D^{25}$ = +2.0 (c=1.0, $CHCl_3$)

^{1}H NMR (600 MHz, $CDCl_3$) δ 7.56 (d, *J* = 7.8 Hz, 1H), 7.46 (d, *J* = 7.8 Hz, 1H), 7.31 (t, *J* = 7.2 Hz, 1H), 7.24 (t, *J* = 7.5 Hz, 1H), 6.77 (s, 1H), 5.62 (t, *J* = 6.6 Hz, 1H), 2.87-2.76 (m, 1H), 2.70-2.52 (m, 3H).

^{13}C NMR (150 MHz, $CDCl_3$) δ 176.3, 155.1, 153.4, 127.4, 125.1, 123.1, 121.4, 111.4, 105.5, 74.6, 28.1, 26.6.

HRMS (ESI) calculated for $C_{12}H_{10}NaO_3$ [M+Na$^+$]: 225.0522, found: 225.0524.

FT-IR (cm-1) 3435, 2927, 1777, 1701, 1453, 1254, 1174, 1142, 919, 747.

MS (EI) *m/z* (%): 147 (100), 145 (60), 157 (58), 131 (36), 210 (34).

The ee value was determined by the chiral UPC^2 analysis (CHIRALPAK® OD-3, CO_2/MeOH = 97/03, v = 2.0 mL/min, λ = 243.0 nm), t_b (minor) = 2.706 min, t_b (major) = 3.162 min, t_c (major) = 3.309 min, t_c (minor) = 3.398 min.

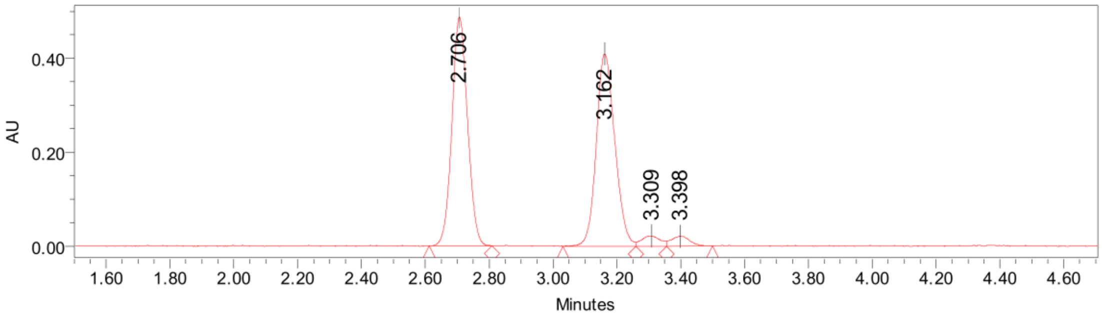

	RT	Area	% Area	Height
1	2.706	1657773	47.52	487535
2	3.162	1656171	47.47	409460
3	3.309	88843	2.55	21832
4	3.398	85810	2.46	20984

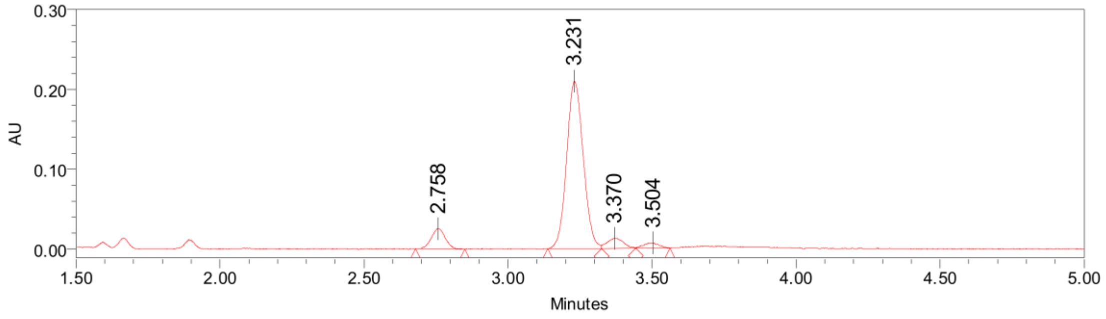

	RT	Area	% Area	Height
1	2.758	85359	8.55	25244
2	3.231	838959	84.03	209826
3	3.370	49816	4.99	12396
4	3.504	24234	2.43	6435

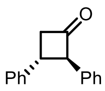

28a

(-)-2,3-diphenylcyclobutan-1-one (28a)

Prepared according to **general procedure**: 36 h, 46% yield, 95.6% ee.

$[\alpha]_D^{23}$ = -131.0 (c = 1.0, $CHCl_3$)

The ee value was determined by the chiral UPC^2 analysis (CHIRALPAK® IG-3, CO_2/MeOH = 95/05, v = 2.0 mL/min, λ = 205.0 nm), t (minor) = 2.343 min, t (major) = 2.645 min.

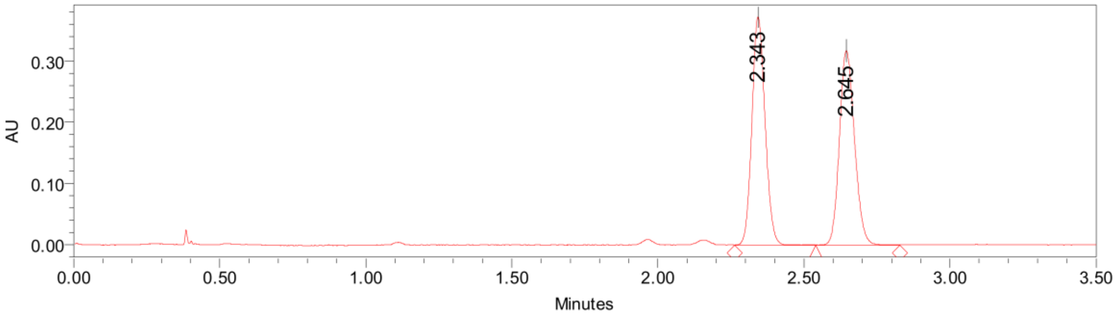

	RT	Area	% Area	Height
1	2.343	1177717	50.73	373975
2	2.645	1143703	49.27	317688

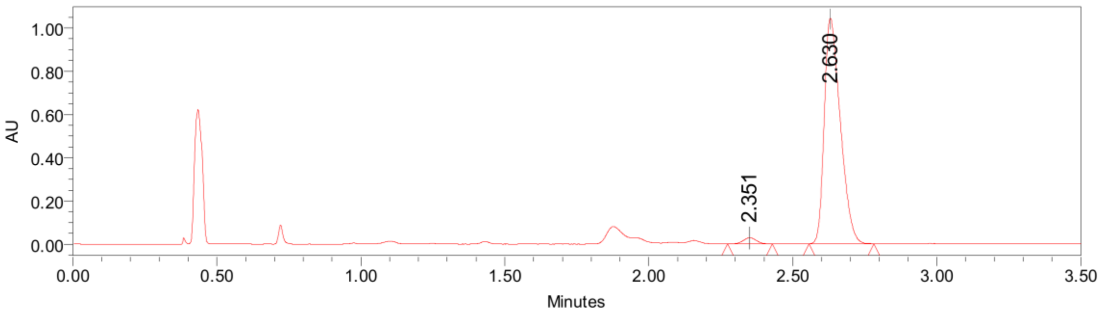

	RT	Area	% Area	Height
1	2.351	90351	2.19	28489
2	2.630	4039982	97.81	1043397

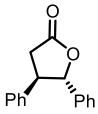

28b

(4*R*,5*S*)-4,5-diphenyldihydrofuran-2(3H)-one (28b)

Prepared according to **general procedure**: 36 h, 52% yield, 93.6% ee, **28b**/**28c** = 12.5/1, white solid, m. p.: 108-109 °C.

[α]D[23] = +114.0 (c = 1.0, $CHCl_3$)

^{1}H NMR (600 MHz, $CDCl_3$) δ 7.40-7.27 (m, 6H), 7.22-7.14 (m, 4H), 5.42 (d, *J* = 8.5 Hz, 1H), 3.59 (dt, *J* = 10.4, 9.0 Hz, 1H), 3.05 (dd, *J* = 17.4, 8.4 Hz, 1H), 2.92 (dd, *J* = 17.4, 10.8 Hz, 1H).

^{13}C NMR (150 MHz, $CDCl_3$) δ 175.2, 137.9, 137.7, 129.1, 128.6, 127.8, 127.3, 125.6, 87.4, 50.5, 37.1.

HRMS (ESI) calculated for $C_{16}H_{15}O_2$ [M+H$^+$]: 239.1067, found: 239.1066.

FT-IR (cm^{-1}): 3032, 2924, 1783, 1455, 1269, 1195, 1141, 1000, 765, 698.

MS (EI) *m/z* (%): 104 (100), 77 (60), 132 (55), 51 (54), 178 (45), 115 (43), 238 (40).

The ee value was determined by the chiral UPC^2 analysis (CHIRALPAK® IG-3, CO_2/MeOH = 95/05, v = 2.0 mL/min, λ = 205.0 nm), t_b (major) = 3.735 min, t_b (minor) = 6.154 min.

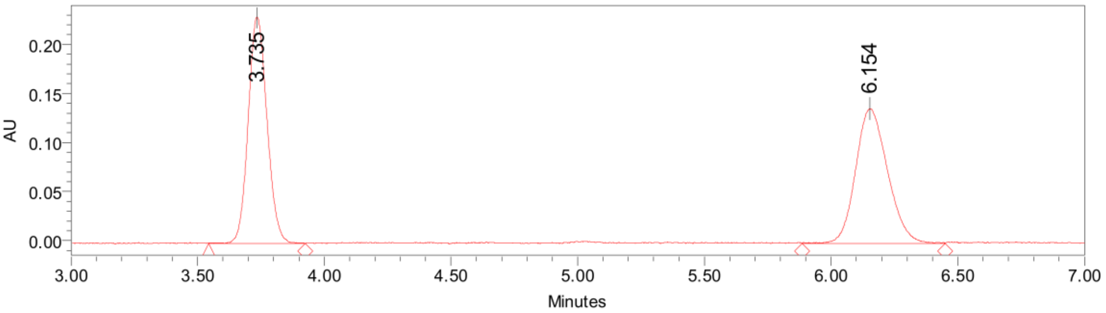

	RT	Area	% Area	Height
1	3.735	1191141	49.71	230722
2	6.154	1204798	50.29	137462

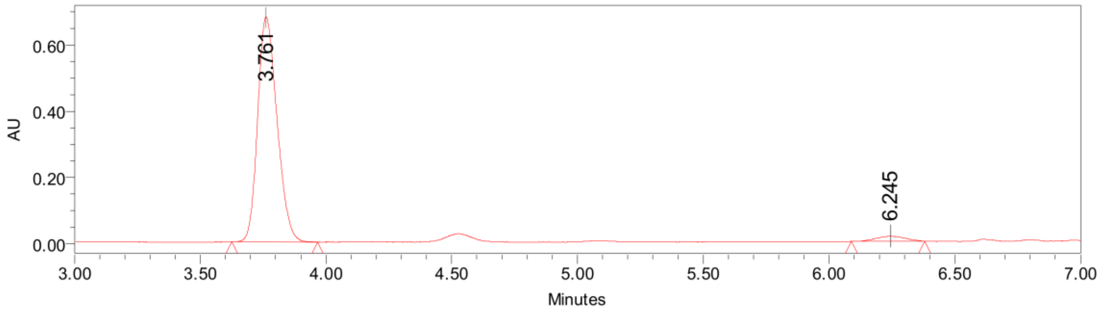

	RT	Area	% Area	Height
1	3.761	3643873	96.81	680090
2	6.245	120197	3.19	15175

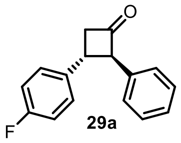

29a

(-)-3-(4-fluorophenyl)-2-phenylcyclobutan-1-one (29a)

Prepared according to **general procedure**: 37 h, 49% yield, 96.0% ee.

[α]D[24] = -44.0 (c = 1.0, $CHCl_3$)

The ee value was determined by the chiral UPC^2 analysis (CHIRALPAK® AD-3, CO_2/MeOH = 95/05, v = 2.0 mL/min, λ = 205.0 nm), t (minor) = 1.400 min, t (major) = 1.782 min.

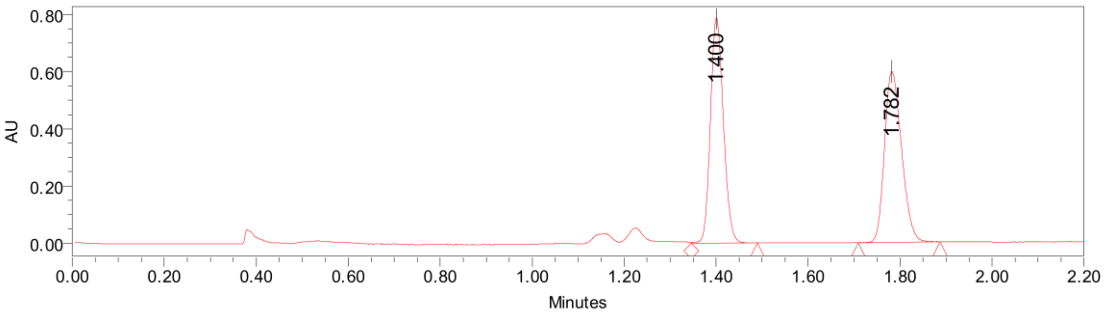

	RT	Area	% Area	Height
1	1.400	1549261	50.04	788175
2	1.782	1546619	49.96	597606

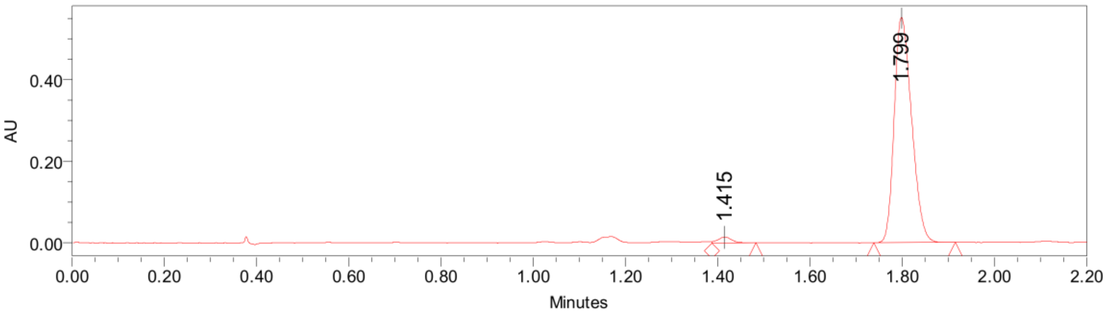

	RT	Area	% Area	Height
1	1.415	29851	2.01	14080
2	1.799	1454547	97.99	551957

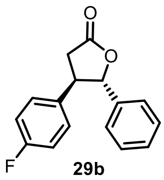

29b

(+)-4-(4-fluorophenyl)-5-phenyldihydrofuran-2(3H)-one (29b)

Prepared according to **general procedure**: 37 h, 50% yield, 93.8% ee, **29b**/**29c** = 8.3/1, white solid, m. p.: 87-89 °C.

[α]$_D$^{24} = +56.0 (c = 1.0, $CHCl_3$)

^{1}H NMR (600 MHz, $CDCl_3$) δ 7.38-7.31 (m, 3H), 7.21-7.16 (m, 2H), 7.16-7.11 (m, 2H), 7.06-7.00 (m, 2H), 5.36 (d, *J* = 8.4 Hz, 1H), 3.58 (dt, *J* = 10.8, 8.4 Hz, 1H), 3.05 (dd, *J* = 17.4, 8.4 Hz, 1H), 2.89 (dd, *J* = 17.4, 10.8 Hz, 1H).

^{13}C NMR (150 MHz, $CDCl_3$) δ 174.9, 162.2 (d, *J* = 245.4 Hz), 137.4, 133.4 (d, *J* = 3.2 Hz), 128.9 (d, *J* = 8.0 Hz), 128.8, 128.7, 125.6, 116.0 (d, *J* = 21.9 Hz), 87.4, 49.9, 37.1.

^{19}F NMR (376 MHz, $CDCl_3$) δ -114.11.

HRMS (ESI) calculated for $C_{16}H_{13}NaO_2F$ [M+Na$^+$]: 279.0792, found: 279.0793.

FT-IR (cm^{-1}): 3035, 2925, 1785, 1512, 1270, 1144, 1000, 840, 700, 522.

MS (EI) *m/z* (%): 122 (100), 121 (70), 96 (57), 77 (56), 101 (55), 51 (53), 228 (40), 256 (30).

The ee value was determined by the chiral UPC^2 analysis (CHIRALPAK® AD-3, CO_2/MeOH = 95/05, v = 2.0 mL/min, λ = 205.0 nm), t_b (major) = 2.430 min, t_b (minor) = 4.063 min, t_c (minor) = 2.827 min, t_c (major) = 6.994 min.

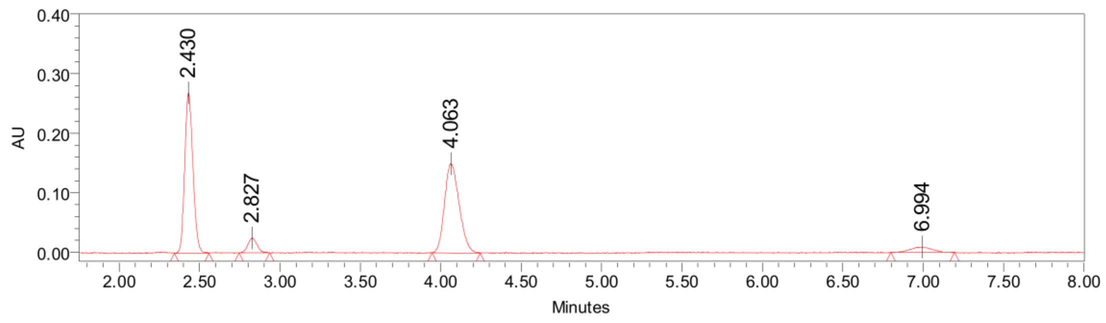

	RT	Area	% Area	Height
1	2.430	951795	45.61	268839
2	2.827	99399	4.76	24900
3	4.063	948675	45.46	150041
4	6.994	86947	4.17	9286

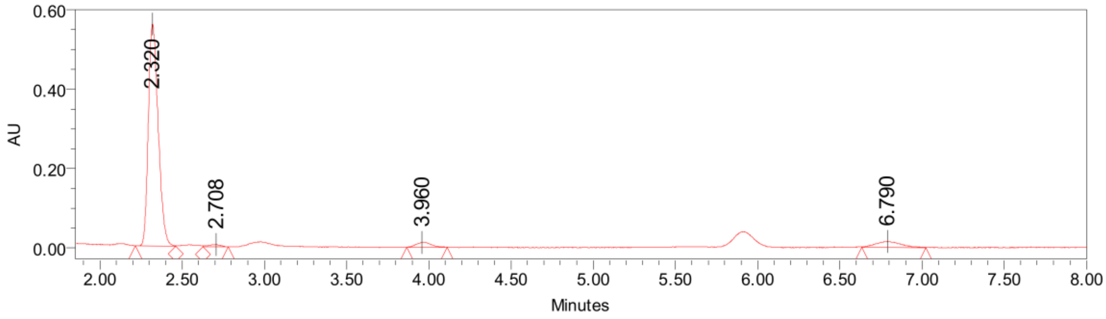

	RT	Area	% Area	Height
1	2.320	2325046	90.18	559909
2	2.708	26379	1.02	5644
3	3.960	74106	2.87	12171
4	6.790	152829	5.93	14894

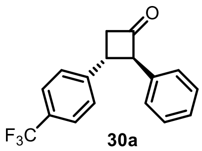

30a

(-)-2-phenyl-3-(4-(trifluoromethyl)phenyl)cyclobutan-1-one (30a)

Prepared according to **general procedure**: 37 h, 48% yield, 95.2% ee.

$[\alpha]_D^{24}$ = -75.0 (c = 1.0, $CHCl_3$)

The ee value was determined by the chiral UPC^2 analysis (CHIRALPAK® AD-3, CO_2/MeOH = 98/02, v = 2.0 mL/min, λ = 220.0 nm), t (minor) = 1.592 min, t (major) = 1.843 min.

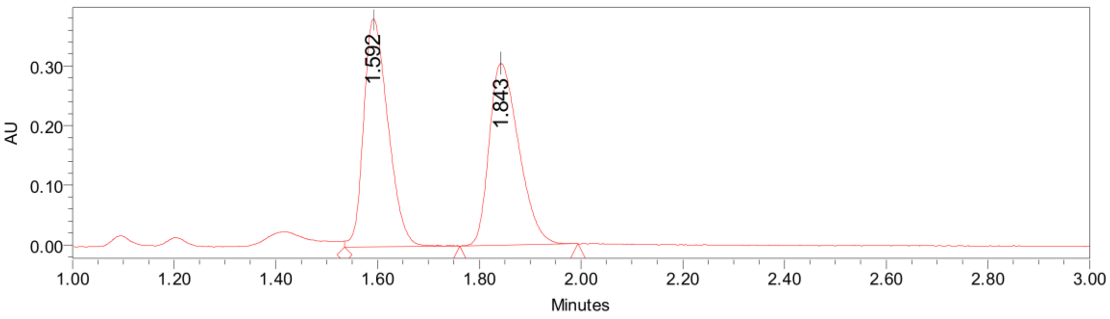

	RT	Area	% Area	Height
1	1.592	1273554	50.79	381738
2	1.843	1234137	49.21	304484

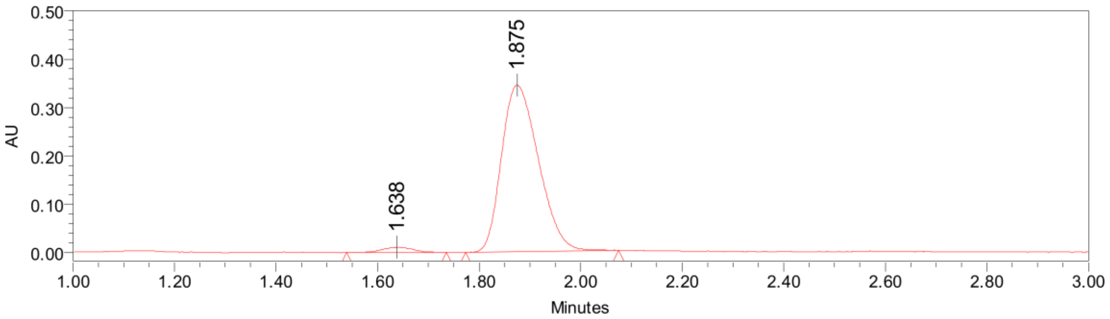

	RT	Area	% Area	Height
1	1.638	42718	2.38	10497
2	1.875	1749222	97.62	344740

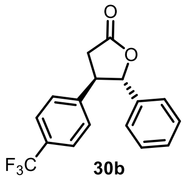

30b

(+)-5-phenyl-4-(4-(trifluoromethyl)phenyl)dihydrofuran-2(3H)-one (30b)

Prepared according to **general procedure**: 37 h, 50% yield, 93.1% ee, **30b**/**30c** = 9.1/1, white solid, m. p.: 99-100 °C.

$[\alpha]_D^{24}$ = +78.0 (c = 1.0, $CHCl_3$)

^{1}H NMR (600 MHz, $CDCl_3$) δ 7.61 (d, *J* = 7.8 Hz, 2H), 7.37-7.33 (m, 3H), 7.31 (d, *J* = 7.8 Hz, 2H), 7.23-7.16 (m, 2H), 5.41 (d, *J* = 8.4 Hz, 1H), 3.68 (dt, *J* = 10.2, 8.5 Hz, 1H), 3.10 (dd, *J* = 17.4, 8.4 Hz, 1H), 2.93 (dd, *J* = 17.4, 10.8 Hz, 1H).

^{13}C NMR (150 MHz, $CDCl_3$) δ 174.6, 141.9, 137.2, 130.0 (q, *J* = 32.4 Hz), 128.9, 128.8, 127.8, 126.0 (q, *J* = 3.7 Hz), 125.6, 123.8 (q, *J* = 270.4 Hz), 87.0, 50.3, 36.8.

^{19}F NMR (376 MHz, $CDCl_3$) δ -62.62.

HRMS (ESI) calculated for $C_{17}H_{13}NaO_2F_3$ [M+Na$^+$]: 329.0760, found: 329.0762.

FT-IR (cm^{-1}): 3035, 1788, 1327, 1123, 1070, 700.

MS (EI) *m/z* (%): 172 (100), 107 (60), 105 (55), 77 (50), 151 (50), 180 (45), 286 (45), 306 (30).

The ee value was determined by the chiral UPC^2 analysis (CHIRALPAK® OD-3, CO_2/MeOH = 98/02, v = 2.0 mL/min, λ = 205.0 nm), t_b (minor) = 3.888 min, t_b (major) = 4.188 min, t_c (major) = 4.546 min, t_c (minor) = 5.117 min.

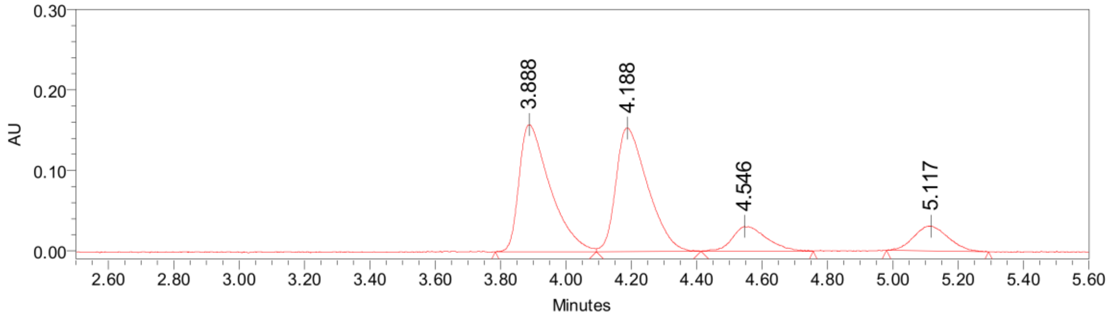

	RT	Area	% Area	Height
1	3.888	1048107	41.20	158253
2	4.188	1039228	40.85	153519
3	4.546	226233	8.89	30761
4	5.117	230548	9.06	31099

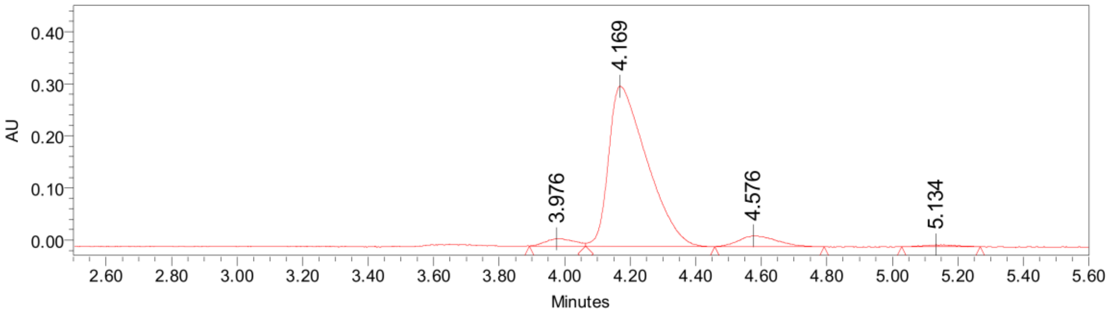

	RT	Area	% Area	Height
1	3.976	89266	3.20	14560
2	4.169	2497288	89.60	307317
3	4.576	173538	6.23	20730
4	5.134	27105	0.97	3837

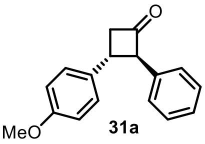

31a

(-)-3-(4-methoxyphenyl)-2-phenylcyclobutan-1-one (31a)

Prepared according to **general procedure**: 37 h, 48% yield, 98.1% ee.

$[\alpha]_D^{24}$ = -178.0 (c = 1.0, $CHCl_3$)

The ee value was determined by the chiral UPC[2] analysis (CHIRALPAK® IG-3, CO_2/MeOH = 90/10, v = 2.0 mL/min, λ = 225.0 nm), t (minor) = 2.576 min, t (major) = 3.524 min.

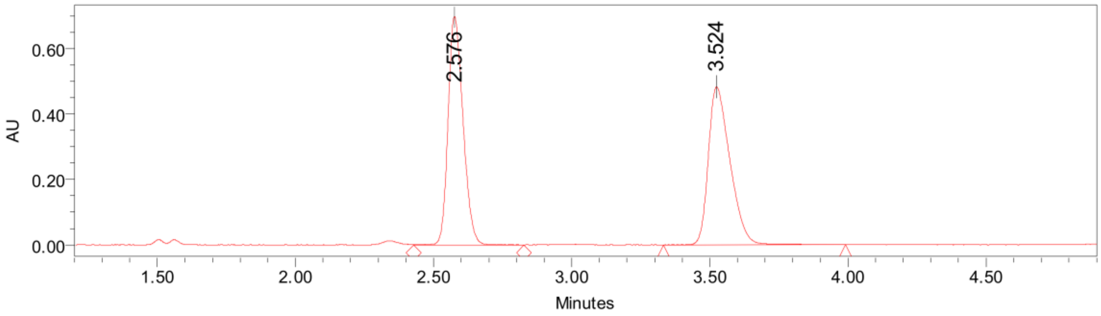

	RT	Area	% Area	Height
1	2.576	2631136	49.83	698890
2	3.524	2649499	50.17	483460

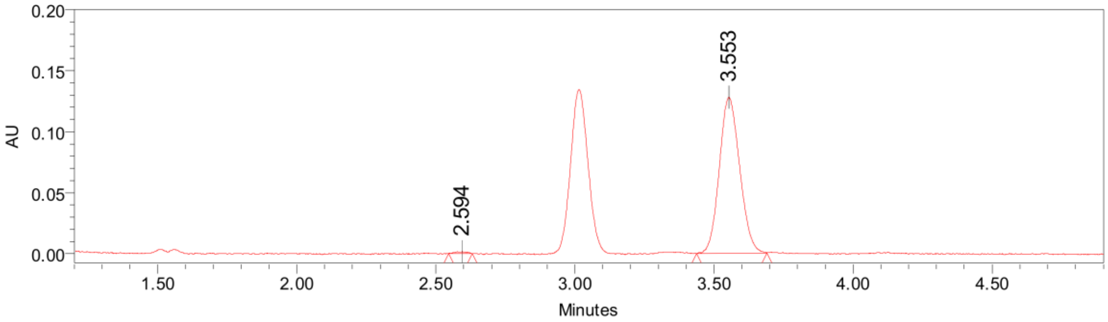

	RT	Area	% Area	Height
1	2.594	4053	0.62	1418
2	3.553	650909	99.38	127806

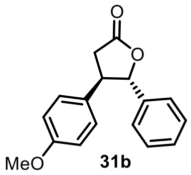
31b

(+)-4-(4-methoxyphenyl)-5-phenyldihydrofuran-2(3H)-one (31b)

Prepared according to **general procedure**: 37 h, 51% yield, 95.6% ee, **31b**/**31c** = 12.5/1, colorless liquid.

$[\alpha]_D^{24}$ = +148.0 (c = 1.0, $CHCl_3$)

^{1}H NMR (600 MHz, $CDCl_3$) δ 7.36-7.29 (m, 3H), 7.23-7.14 (m, 2H), 7.08 (d, *J* = 8.4 Hz, 2H), 6.87 (d, *J* = 8.4 Hz, 2H), 5.36 (d, *J* = 8.4 Hz, 1H), 3.79 (s, 3H), 3.53 (dt, *J* = 10.7, 8.6 Hz, 1H), 3.02 (dd, *J* = 17.4, 8.4 Hz, 1H), 2.88 (dd, *J* = 17.4, 11.1 Hz, 1H).

^{13}C NMR (150 MHz, $CDCl_3$) δ 175.3, 159.1, 137.7, 129.6, 128.6, 128.6, 128.4, 128.4, 125.6, 125.6, 114.4, 87.6, 55.2, 49.9, 37.2.

HRMS (ESI) calculated for $C_{17}H_{17}O_3$ [M+H$^+$]: 269.1172, found: 269.1171.

FT-IR (cm^{-1}): 2933, 2836, 1785, 1515, 1253, 1180, 1034, 1000, 700.

MS (EI) *m/z* (%): 134 (100), 91 (60), 119 (58), 135 (55), 77 (50), 152 (45), 165 (43), 268 (30).

The ee value was determined by the chiral UPC^2 analysis (CHIRALPAK® IG-3, CO_2/MeOH = 90/10, v = 2.0 mL/min, λ = 225.0 nm), t_b (major) = 3.009 min, t_b (minor) = 5.390 min, t_c (major) = 11.644 min, t_c (minor) = 5.117 min.

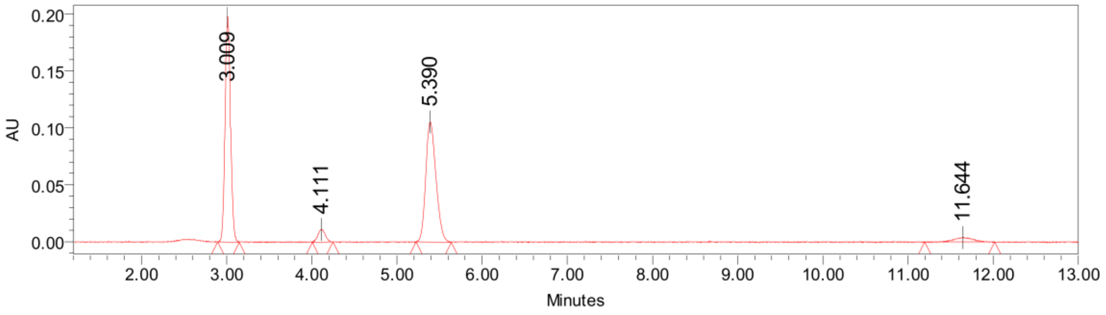

	RT	Area	% Area	Height
1	3.009	879238	46.54	198297
2	4.111	67608	3.58	11172
3	5.390	876783	46.41	105749
4	11.644	65574	3.47	4127

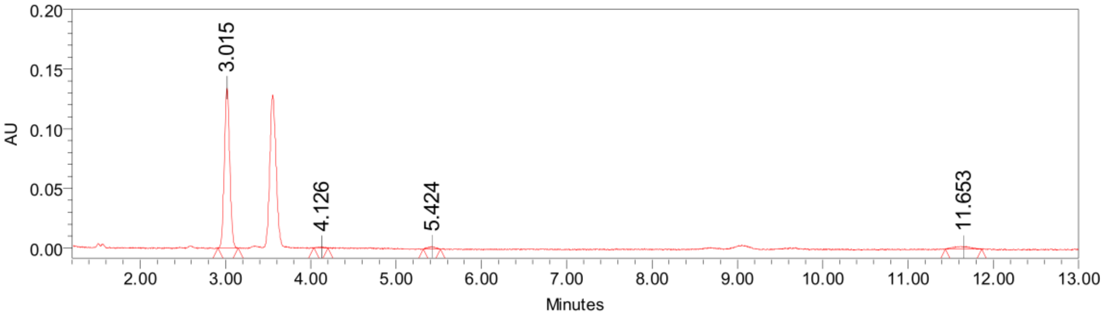

	RT	Area	% Area	Height
1	3.015	589783	92.39	134722
2	4.126	4970	0.78	1185
3	5.424	14241	2.23	2303
4	11.653	29340	4.60	2139

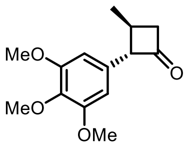

***ent*-32a**

(+)-3-methyl-2-(3,4,5-trimethoxyphenyl)cyclobutan-1-one (*ent*-32a)

Prepared according to **indicated procedure d**: 32 h, 40% yield, 91.0% ee, colorless liquid.

[α]$_D$^{26} = + 45.0 (c=1.0, $CHCl_3$)

The ee value was determined by the chiral UPC^2 analysis (Trefoil® CEL 1, CO_2/MeOH = 95/05, v = 2.0 mL/min, λ = 205.0 nm), t (minor) = 1.627 min, t (major) = 2.137 min.

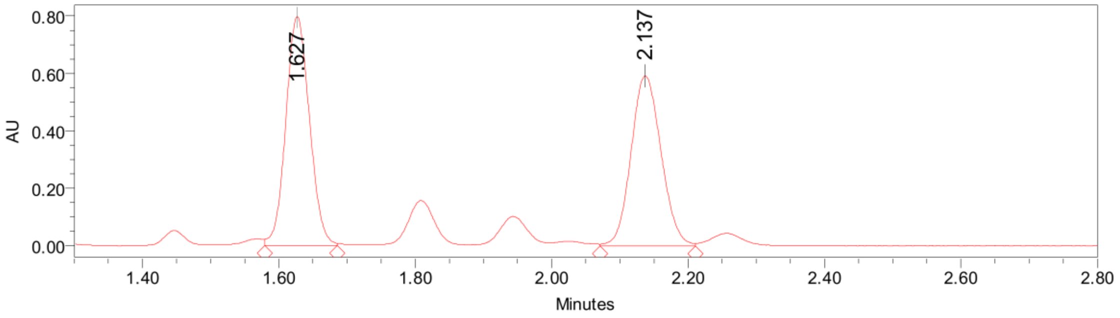

	RT	Area	% Area	Height
1	1.627	1924233	51.44	798179
2	2.137	1816561	48.56	592208

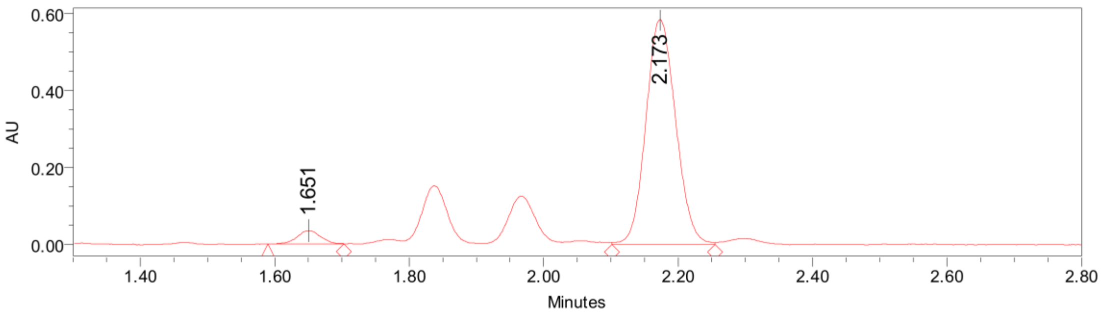

	RT	Area	% Area	Height
1	1.651	85087	4.51	34255
2	2.173	1801260	95.49	583849

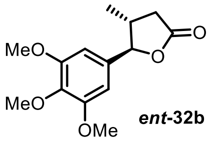

***ent*-32b**

(-)-4-methyl-5-(3,4,5-trimethoxyphenyl)dihydrofuran-2(3H)-one (*ent*-32b)

Prepared according to **indicated procedure d**: 32 h, 48% yield, 92.4% ee, **32b**/**32c** = 19/1, yellow liquid.

[α]$_D$^{26} = -5.0 (c = 1.0, $CHCl_3$)

The ee value was determined by the chiral UPC^2 analysis (CHIRALPAK® IG-3, CO_2/MeOH = 90/10, v = 2.0 mL/min, λ = 205.0 nm), t_b (major) = 2.837 min, t_b (minor) =4.250 min, t_c (major) = 3.844 min, t_c (minor) = 4.647 min.

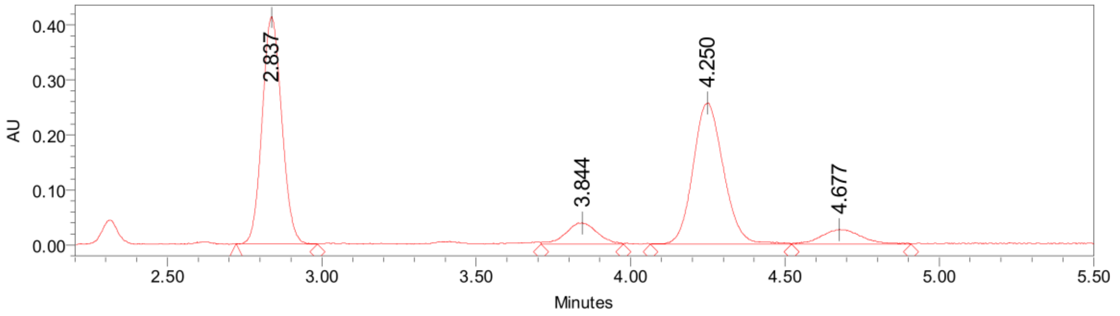

	RT	Area	% Area	Height
1	2.837	1822597	44.49	413263
2	3.844	276543	6.75	38775
3	4.250	1758816	42.93	256854
4	4.677	238914	5.83	26477

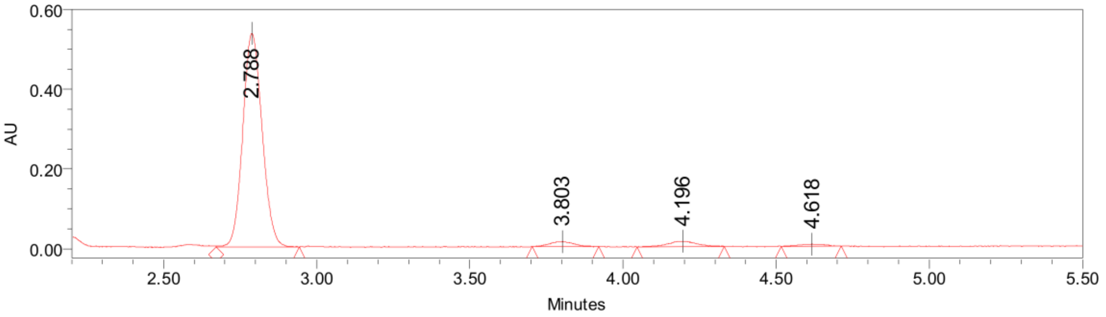

	RT	Area	% Area	Height
1	2.788	2358562	92.33	536067
2	3.803	69091	2.70	12399
3	4.196	93119	3.65	13719
4	4.618	33651	1.32	5618

9. Optimization of direct α-arylation of cyclobutanone (Table S4)[a]

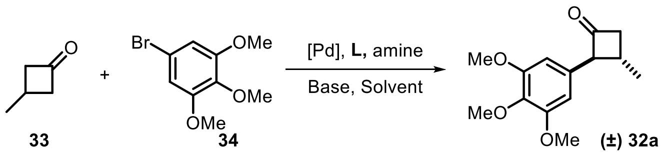

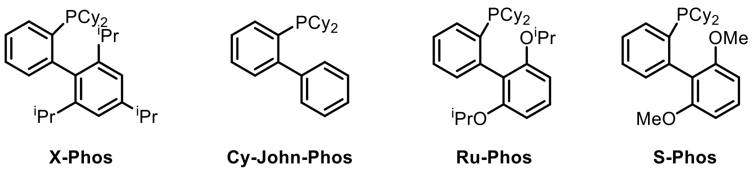

entry	[pd]	Ligand	Amine	Base	Solvent	Yield%	dr
1	$Pd(OAc)_2$	X-Phos	Pyrrolidine	K_3PO_4	1,4-Dioxane	24	3.2:1
2	$Pd(CF_3CO_2)_2$	X-Phos	Pyrrolidine	K_3PO_4	1,4-Dioxane	17	3.0:1
3	$Pd(PivO)_2$	X-Phos	Pyrrolidine	K_3PO_4	1,4-Dioxane	48	4.1:1
4	$Pd(PPh_3)_4$	X-Phos	Pyrrolidine	K_3PO_4	1,4-Dioxane	N.P.	-
5	$Pd(PivO)_2$	Cy-John-Phos	Pyrrolidine	K_3PO_4	1,4-Dioxane	trace	-
6	$Pd(PivO)_2$	Ru-Phos	Pyrrolidine	K_3PO_4	1,4-Dioxane	34	3.2:1
7	$Pd(PivO)_2$	Sphos	Pyrrolidine	K_3PO_4	1,4-Dioxane	42	3.4:1
8[b]	$Pd(PivO)_2$	X-Phos	Pyrrolidine	K_3PO_4	THF	41	4.1:1
9	$Pd(PivO)_2$	X-Phos	Pyrrolidine	K_3PO_4	Toluene	26	3.7:1
10	$Pd(PivO)_2$	X-Phos	Pyrrolidine	$K_3PO_3{\bullet}3H_2O$	1,4-Dioxane	52	4.2:1
11	$Pd(PivO)_2$	X-Phos	Pyrrolidine	CsF	1,4-Dioxane	53	4.2:1
12	$Pd(PivO)_2$	X-Phos	Dibenzylamine	CsF	1,4-Dioxane	60	5.6:1
13	$Pd(PivO)_2$	X-Phos	Piperidine	CsF	1,4-Dioxane	47	3.7:1
14[c]	$Pd(PivO)_2$	X-Phos	Dibenzylamine	CsF	1,4-Dioxane	70	5.6:1
15[d]	$Pd(PivO)_2$	X-Phos	Dibenzylamine	CsF	1,4-Dioxane	65	5.6:1

[a] Reaction condition: the reactions were performed with **33** (1.5 equiv), **34** (0.2 mmol), [Pd] (10 mol%), ligand (20

mol%), amine (20 mol%), base (2.0 equiv) in solvent (2 mL) at 110 °C (oil temperature). Yield and dr was determined by NMR. [b] The oil temperature was 80 °C. [c] **33** (2.0 equiv) was used and the isolated yield of the reaction was 65 %. [d] 1.0 g **34** and 33 (2.0 equiv) was used. Isolated yield.

10. Synthesis of eupomatilone-5 and eupomatilone-6

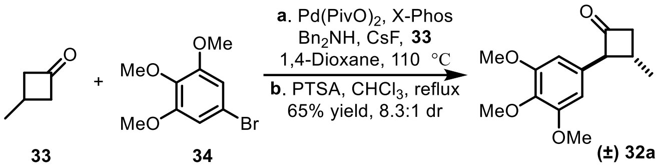

Compound (±) 32a: A 1,4-dioxane (10 mL) solution of 3-methylcyclobutan-1-one (681 mg, 8.1 mmol, 2.0 equiv), 5-bromo-1,2,3-trimethoxybenzene (1.0 g, 4.0 mmol, 1.0 equiv), Pd(PivO)$_2$ (125 mg, 0.4 mmol, 0.1 equiv), X-phos (386 mg, 0.8 mmol, 0.2 equiv), dibenzylamine (156 µL, 0.8 mmol, 0.2 equiv), CsF (1.23 g, 8.0 mmol, 2.0 equiv) was degassed with argon gas for 5 min and heated to 110 °C for 16 h before it was cooled down to room temperature. Water (10 mL) and EA was added and the mixture was extracted with EA three times. The combined organic layers were washed with brine three times, dried (NaSO$_4$), concentrated and the crude product was dissolved in CHCl$_3$ (20 mL). *p*-toluenesulfonic acid monohydrate (228 mg, 1.2 mmol, 0.3 equiv) was added, and the mixture was refluxing for 3 h before it was cooled down to room temperature. Water (10 mL) and EA was added and the mixture was extracted with EA three times. The combined organic layers were washed with water, brine, dried (NaSO$_4$), concentrated and the crude product was purified by flash column chromatography on silica gel to afford **(±) 32a** as colorless liquid (655 mg, 65% yield, 8.3:1 dr).

^{1}H NMR (400 MHz, CDCl$_3$) δ 6.44 (s, 2H), 3.98-3.93 (m, 1H), 3.86 (s, 6H), 3.82 (s, 3H), 3.19-3.08 (m, 1H), 2.87-2.76 (m, 1H), 2.69-2.57 (m, 1H), 1.51 (d, J = 6.4 Hz, 3H).

^{13}C NMR (100 MHz, CDCl$_3$) δ 206.3, 153.3, 137.0, 131.7, 103.9, 71.0, 60.7, 56.0, 51.4, 27.9, 21.3.

HRMS (ESI) calculated for $C_{14}H_{19}O_4$ [M+H$^+$]: 251.1278, found: 251.1279.

FT-IR (cm^{-1}): 3534, 2959, 1776, 1586, 1455, 1420, 1242, 1127, 1010.

MS (EI) *m/z* (%): 208 (100), 193 (60), 250 (29), 209 (15),165 (10).

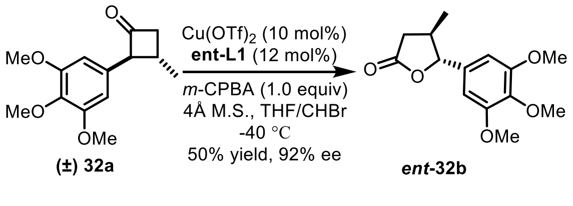

Compound *ent*-32b: The mixture of anhydrous Cu(OTf)$_2$ (7.2 mg, 0.02 mmol),***ent*-L1** (6.4 mg, 0.024 mmol) was stirred in THF (1 mL) for 1h at room temperature. The 4Å molecular sieves (60 mg), Al(O^{i}Pr)$_3$ (20.4 mg, 50% mol) and **(±)-32a** was added to an oven-dried reaction tube , then the chiral catalyst solution prepared beforehand , THF (1 mL), CHBr$_3$ was added, the reaction mixture was cooled to -40 °C for 20 min. Then, the *m*-CPBA (0.2 mmol) was added to the reaction mixture a stirred at -40 °C. The Na$_2$SO$_3$ (126 mg, 1.0 mmol) was added after the reaction mixture was stirred for 32 h at -40 °C, and stirred for more 2 h at -40 °C. The DCM (10 mL) was added, the solution was extracted with DCM. The combined organic layers were washed with brine, dried (NaSO$_4$), concentrated and the crude product was purified by flash column chromatography on silica gel at **-20** °C to afford ***ent*-32b** (25.6 mg, 48% yield) as a yellow liquid, 92.4% ee.

[α]D27 = -5.0 (c = 1.0, $CHCl_3$)

^{1}H NMR (400 MHz, $CDCl_3$) δ 6.54 (s, 2H), 4.87 (d, *J* = 8.5 Hz, 1H), 3.88 (s, 6H), 3.85 (s, 3H), 2.80 (dd, *J* = 16.9, 7.6 Hz, 1H), 2.56-2.42 (m, 1H), 2.35 (dd, *J* = 16.9, 10.7 Hz, 1H), 1.21 (d, *J* = 6.5 Hz, 3H).

^{13}C NMR (100 MHz, $CDCl_3$) δ 176.0, 153.4, 138.1, 133.4, 102.9, 88.2, 60.8, 56.2, 39.8, 37.2, 16.5.

HRMS (ESI) calculated for $C_{14}H_{18}NaO_5$ [M+Na$^+$]: 289.1046, found:289.1046.

FT-IR (cm^{-1}): 3522, 2964, 1776, 1593, 1463, 1240, 1126, 1005, 987.

MS (EI) *m/z* (%): 266 (100), 196 (45), 181 (40), 169 (25), 125 (25), 167 (15).

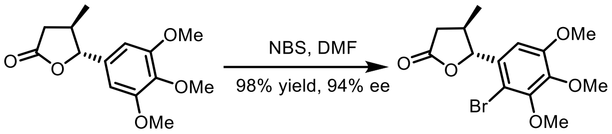

***ent*-32b** **36a**

Compound 36a: To a solution of ***ent*-32b** (300 mg, 1.1 mmol) in DMF (2 mL) was added N-Bromosuccinimide (220 mg, 1.2 mmol) at 0 °C, stirred for 12 h at room temperature. Water (10 mL) and EA was added and the solution was extracted with EA three times. The combined organic layers were washed with brine ten times, dried ($NaSO_4$), concentrated and the crude product was purified by column chromatography on silica gel to afford **36a** (380 mg, 98% yield) as colorless liquid, 94.1% ee.

[α]D27 = +24.0 (c = 1.0, $CHCl_3$)

^{1}H NMR (600 MHz, $CDCl_3$) δ 6.67 (s, 1H), 5.44 (d, *J* = 5.4 Hz, 1H), 3.91 (s, 3H), 3.89 (s, 3H), 3.86 (s, 3H), 2.75 (dd, *J* = 17.4, 7.8 Hz, 1H), 2.61-2.49 (m, 1H), 2.29 (dd, *J* = 17.4, 6.6 Hz, 1H), 1.33 (d, *J* = 7.2 Hz, 3H).

^{13}C NMR (150 MHz, $CDCl_3$) δ 176.6, 153.2, 150.9, 143.0, 133.4, 108.2, 105.1, 86.0, 61.0, 56.2, 38.9, 35.8, 18.2.

HRMS (ESI) calculated for $C_{14}H_{17}BrNaO_5$ [M+Na$^+$]: 367.0152, 369.0131, found: 367.0152, 369.0132.

FT-IR (cm^{-1}): 3542, 2938, 1781, 1485, 1396, 1327, 1202, 1106, 1006, 941.

MS (EI) *m/z* (%): 344 (100), 146 (95), 276 (56), 275 (55), 274 (53), 277 (46), 169 (28), 259 (20), 261 (18).

The ee value was determined by the chiral HPLC analysis (CHIRALPAK® OD-H, Hexane/ Isopropanol = 90/10, v = 1.0 mL/min, λ = 210.0 nm), t (major) = 11.436, t (minor) = 12.699.

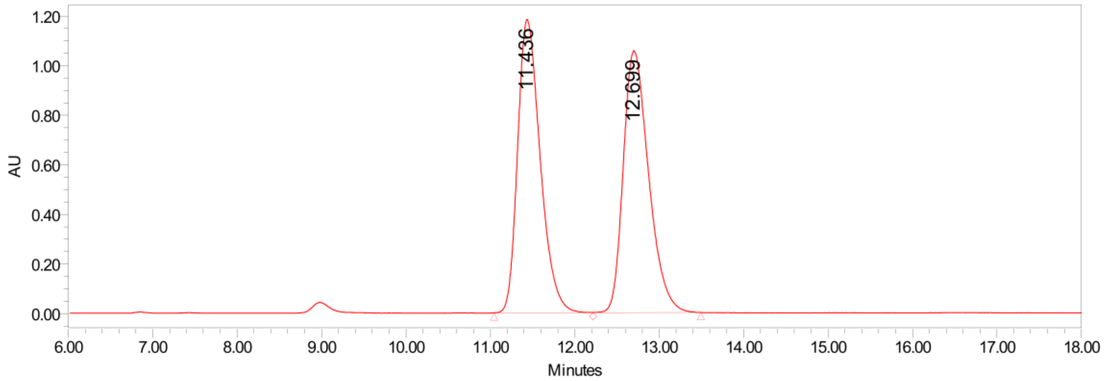

peak information

	RetTime (min)	Area (μV*s)	Area (%)	Height (μV)
1	11.436	22012537	49.93	1183177
2	12.699	22078440	50.07	1056870

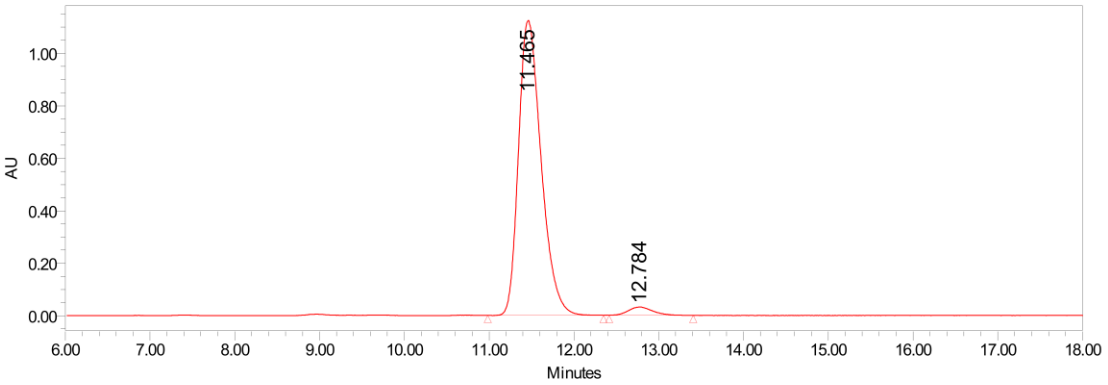

peak information

	RetTime (min)	Area (μV*s)	Area (%)	Height (μV)
1	11.465	20847498	97.05	1123564
2	12.784	633405	2.95	31811

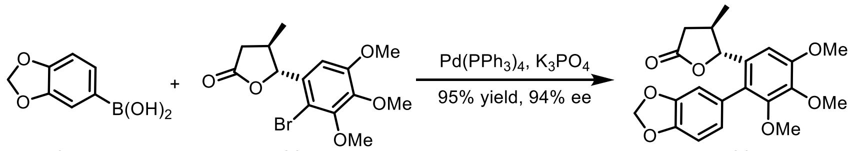

35 36a 36

Compound 36: To a solution of **36a** (300 mg, 0.87 mmol) in dioxane (0.1 M, 8.7 mL), K_3PO_4 (922 mg, 4.3 mmol, 5 equiv), 3,4-(methylenedioxy)phenylboronic acid (**35**, 431 mg, 2.6 mmol, 3 equiv) and $Pd(PPh_3)_4$ (50 mg, 0.043 mmol) was added . The reaction mixture was degassed with argon gas for 10 min and stirred for 2 h at 100 °C. Then the resulting mixture was filtered through a pad of celite and concentrated and the crude product was purified by column chromatography on silica gel to afford **36** (319 mg, 95% yield as a mixture of atropdiastereomers) as a colorless liquid, 94.4% ee.

$[\alpha]_D^{27}$ = -58.0 (c = 1.0, $CHCl_3$).

^{1}H NMR (400 MHz, $CDCl_3$) δ 6.91-6.88 (m, 1H), 6.88-6.84 (m, 1H), 6.74 (d, *J* = 1.6 Hz, 1H), 6.73-6.68 (m, 4H), 6.64 (dd, *J* = 8.0, 1.6 Hz, 1H), 6.07-5.97 (m, 4H), 4.98 (d, *J* = 7.6 Hz, 1H), 4.93 (d, *J* = 7.4 Hz, 1H), 3.91 (s, 6H), 3.90 (s, 6H), 3.64 (s, 3H), 3.64 (s, 3H), 2.74 (dd, J = 8.0, 4.8 Hz, 1H), 2.70 (dd, J = 8.0, 4.8 Hz, 1H), 2.56-2.40 (m, 2H), 2.18 (dd, J = 9.4, 2.6 Hz, 1H), 2.14 (dd, J = 9.4, 2.6 Hz, 1H), 0.88 (d, J = 5.6 Hz, 3H), 0.86 (d, J = 5.6 Hz, 3H).

^{13}C NMR (100 MHz, $CDCl_3$) δ 176.4, 176.4, 153.2, 153.2, 151.4, 151.3, 147.5, 147.4, 146.9, 146.8, 142.3, 142.3, 131.7, 131.6, 128.8, 128.8, 128.8, 124.1, 123.1, 111.2, 110.3, 108.2, 107.9, 104.4, 101.1, 101.1, 84.7, 84.7, 61.1, 61.0, 60.8, 56.1, 39.2, 39.2, 36.9, 36.8, 16.78.

HRMS (ESI) calculated for $C_{21}H_{22}NaO_7$ [M+Na$^+$]: 409.1258, found: 409.1257.

FT-IR (cm^{-1}): 3487, 2965, 1777, 1483, 1324, 1224, 1127, 1002, 754.

MS (EI) *m/z* (%): 386 (100), 299 (60), 327 (15), 249 (13), 316 (12).

The ee value was determined by the chiral HPLC analysis (CHIRALPAK® OD-H, Hexane/ Isopropanol = 80/20, v = 1.0 mL/min, λ = 207.0 nm), t (major) = 8.971, t (minor) = 12.384

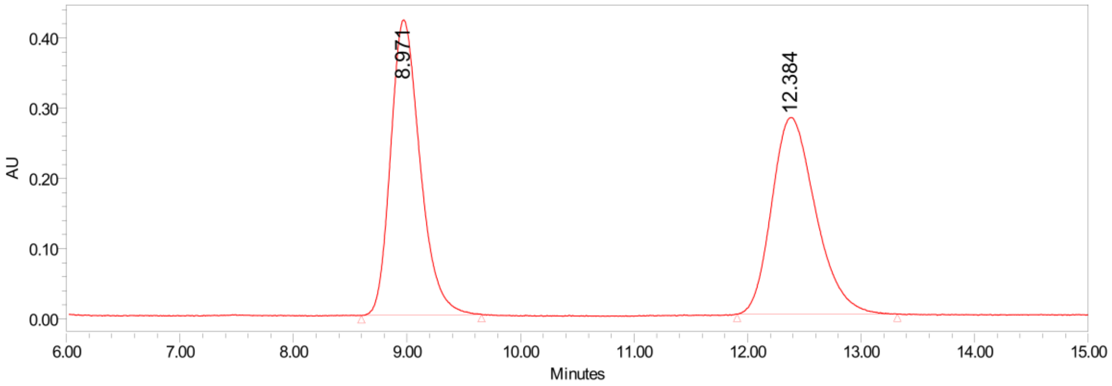

peak information

	RetTime (min)	Area (μV*s)	Area (%)	Height (μV)
1	8.971	7584074	50.25	420598
2	12.384	7509456	49.75	279845

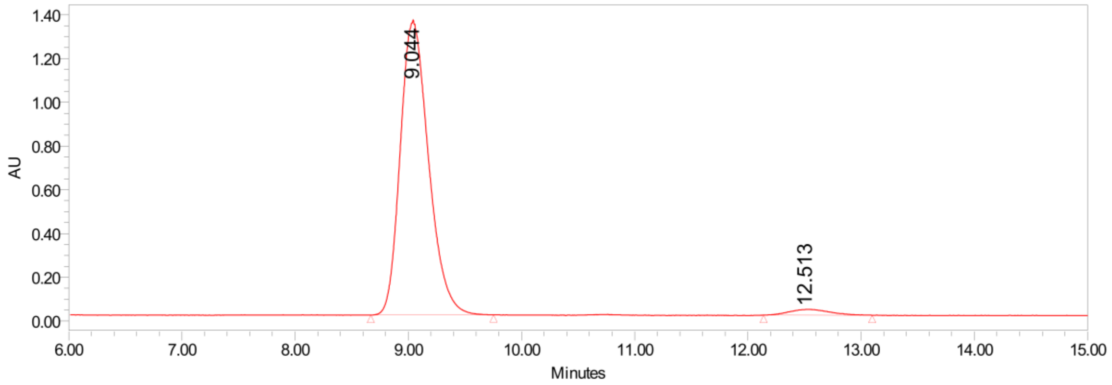

peak information

	RetTime (min)	Area (μV*s)	Area (%)	Height (μV)
1	9.044	23421479	97.21	1350041
2	12.513	672050	2.79	26776

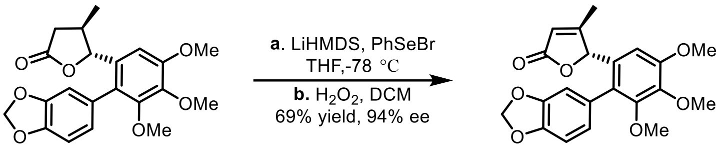

36 37a

Compound 37a: Compound **36** (300 mg, 0.78 mmol) was dissolved in dry THF under argon atmosphere at room temperature and then cooled down to -78 °C. Lithium bis(trimethylsilyl)amide (1.0 M in THF, 1.55 mL 2 equiv) was added dropwise via a syringe. The resulted solution was stirred at -78 °C for one hour. Then PhSeBr (275 mg, 1.16 mmol, 1.5 equiv) was added, and the mixture was stirred at -78 °C. The reaction mixture was quenched with saturated NH_4Cl aqueous solution after full conversion monitored by TLC, and the extracted with DCM. The combined organic layers were washed again with brine, dried ($NaSO_4$) and concentrated. The residue was dissolved in DCM and cooled to 0 ℃. 30% H_2O_2 solution (775 μL, 10 equiv) was added dropwise and stirred for 1 h at 0 °C. After full conversion, water (10 mL) was added, the solution was extracted with DCM. The combined organic layers were washed with brine, dried ($NaSO_4$), concentrated and the crude product was purified by flash column chromatography on silica gel to afford **37a**[7] (207 mg, 69% yield, as a mixture of

atropdiastereomers) as yellow liquid, 94.5% ee.

[α]$_D^{27}$ = -66.0 (c = 1.0, $CHCl_3$).

^{1}H NMR (400 MHz, $CDCl_3$) δ 6.88 (t, *J* = 7.6 Hz, 2H), 6.85-6.79 (m, 2H), 6.75 (d, *J* = 1.2 Hz, 1H), 6.69 (dd, *J* = 8.0, 1.6 Hz, 1H), 6.32 (s, 2H), 6.06-5.99 (m, 4H), 5.91 (s, 2H), 5.70 (s, 2H), 3.92 (d, *J* = 0.8 Hz, 6H), 3.83 (s, 6H), 3.65 (s, 3H), 3.64 (s, 3H), 1.87 (s, 3H), 1.86 (s, 3H).

^{13}C NMR (100 MHz, $CDCl_3$) δ 173.4, 173.3, 168.5, 153.5, 153.5, 151.7, 151.7, 147.7, 147.5, 147.1, 143.1, 130.4, 130.4, 128.5, 128.5, 127.6, 127.6, 123.8, 116.9, 116.8, 110.9, 110.9, 108.3, 108.0, 104.5, 104.4, 101.2, 101.2, 83.2, 83.1, 61.1, 61.1, 60.9, 56.1, 14.3.

HRMS (ESI) calculated for $C_{21}H_{21}O_7$ [M+H$^+$]: 385.1282, found: 385.1280.

FT-IR (cm^{-1}): 2937, 1759, 1483, 1237, 1127, 1087, 756.

MS (EI) *m/z* (%): 384 (100), 385 (25), 135 (27), 115 (25), 335 (23), 307 (20), 152 (20), 285 (10).

The ee value was determined by the chiral HPLC analysis (CHIRALPAK® OD-H, Hexane/ Isopropanol = 80/20, v = 1.0 mL/min, λ = 210.0 nm), t (major) = 9.398, t (minor) = 13.691.

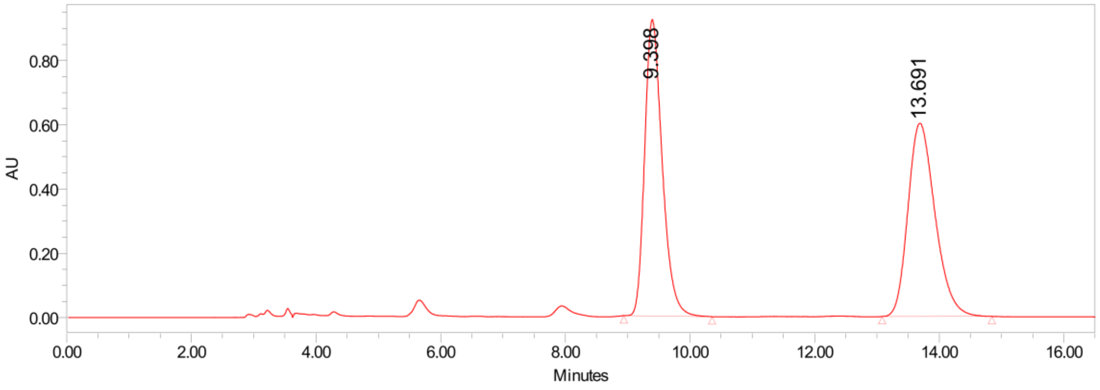

peak information

	RetTime (min)	Area (μV*s)	Area (%)	Height (μV)
1	9.398	18057296	49.81	923002
2	13.691	18195800	50.19	601653

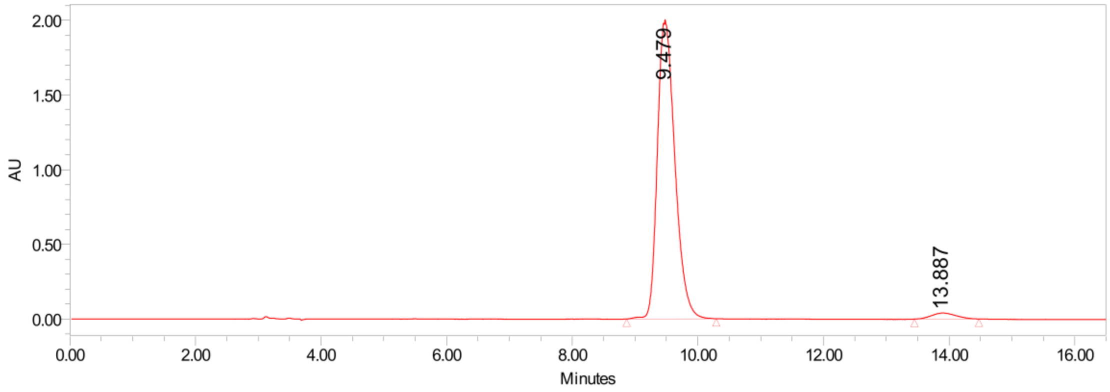

peak information

	RetTime (min)	Area (μV*s)	Area (%)	Height (μV)
1	9.479	38776237	97.27	2001450
2	13.887	1088323	2.73	39860

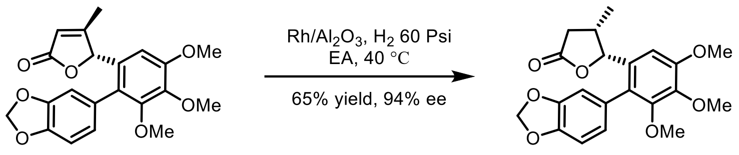

Compound 37: Compound **37** was prepared according to the literature.[8] Compound **37a** (82 mg, 0.21 mmol) was dissolved in dry EA at room temperature and Rh/Al_2O_3 (5%, 10 mg) was added. Then the reaction mixture stirred under H_2 (5 atm) at 40 °C for 24 h before the catalyst was filtered, and the filtrate concentrated to afford a residue, which on purification by column chromatography on silica gel to afford **37** (53.5 mg, 65% yield, as a mixture of atropdiastereomers) as white amorphous solid, 94.4% ee, m. p.: 166.0-167.8 °C

$[\alpha]_D^{27}$ = +21.0 (c = 1.0, $CHCl_3$)

^{1}H NMR (600 MHz, $CDCl_3$) δ 6.87 (dd, *J* = 7.8, 6.0 Hz, 2H), 6.81 (d, *J* = 4.8 Hz, 2H), 6.72 (d, *J* = 1.8 Hz, 1H), 6.69 (dd, *J* = 7.8, 1.8 Hz, 1H), 6.62 (d, *J* = 1.8 Hz, 1H), 6.57 (dd, *J* = 7.8, 1.8 Hz, 1H), 6.04 (d, *J* = 1.8 Hz, 2H), 6.02 (dd, *J* = 2.4, 1.2 Hz, 2H), 5.50 (d, *J* = 6.0 Hz, 1H), 5.41 (d, *J* = 5.4 Hz, 1H), 3.91 (s, 12H), 3.65 (s, 3H), 3.65 (s, 3H), 2.70 (dt, *J* = 17.4, 7.8 Hz, 2H), 2.30-2.19 (m, 4H), 0.70 (d, *J* = 7.2 Hz, 3H), 0.67 (d, *J* = 7.2 Hz, 3H).

^{13}C NMR (150 MHz, $CDCl_3$) δ 176.4, 152.8, 151.5, 147.7, 147.6, 147.0, 146.9, 130.1, 129.1, 126.4, 126.3, 123.4, 122.4, 110.6, 109.6, 108.5, 108.2, 104.7, 104.6, 101.2, 101.2, 82.1, 82.1, 61.2, 61.1, 60.9, 56.2, 37.9, 34.0, 33.7, 15.5, 15.4.

HRMS (ESI) calculated for $C_{21}H_{22}NaO_7$ [M+Na$^+$]: 409.1258, found: 409.1258.

FT-IR (cm^{-1}): 2968, 2936, 1780, 1482, 1457, 1236, 1162, 1127, 1038, 752.

MS (EI) *m/z* (%): 386 (100), 299 (60), 327 (15), 249 (13), 316 (12).

The ee value was determined by the chiral HPLC analysis (CHIRALPAK® OD-H, Hexane/ Isopropanol = 80/20, v = 1.0 mL/min, λ = 207.0 nm), t (minor) = 10.422, t (major) = 12.463.

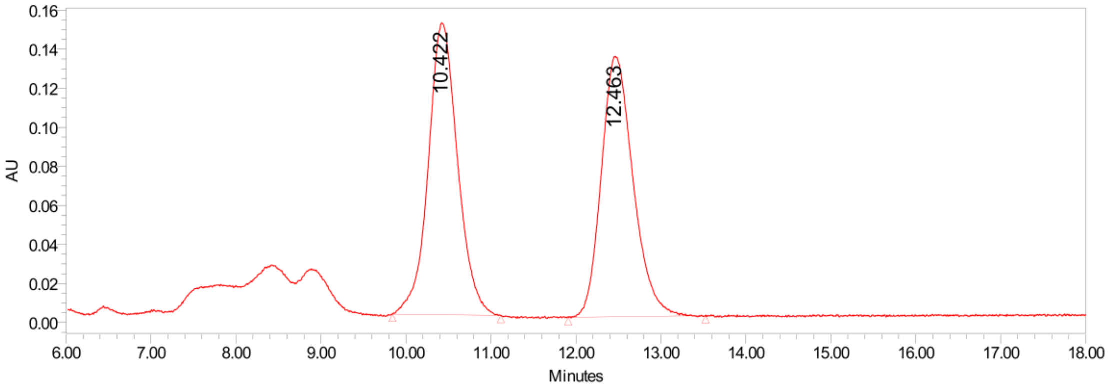

peak information

	RetTime (min)	Area (μV*s)	Area (%)	Height (μV)
1	10.422	3584151	50.77	149478
2	12.463	3475139	49.23	133436

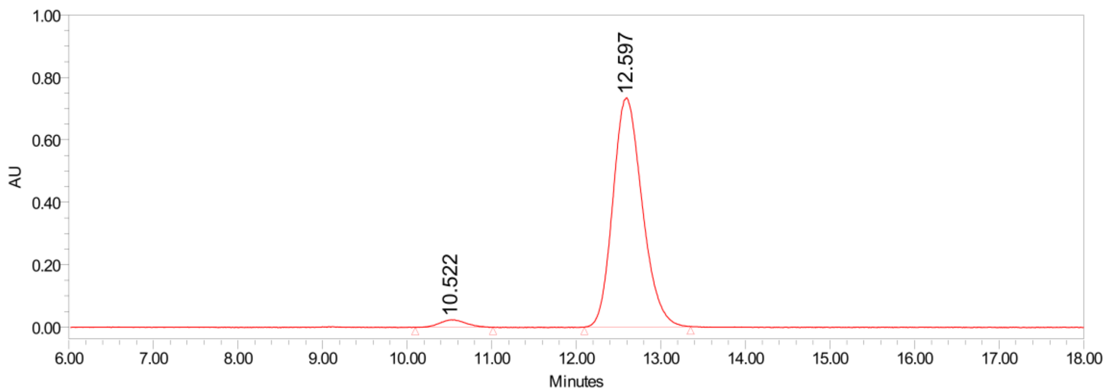

peak information

	RetTime (min)	Area (μV*s)	Area (%)	Height (μV)
1	10.522	513261	2.82	24119
2	12.597	17685972	97.18	734509

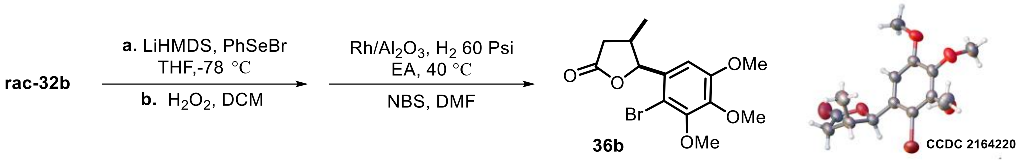

This compound was synthesized according to the procedure above and the relative configuration of **36b** was confirmed by X-ray analysis. The asymmetric product **32b** would be partially racemized after these reactions.

^{1}H NMR (600 MHz, $CDCl_3$) δ 6.81 (s, 1H), 5.77 (d, *J* = 6.0 Hz, 1H), 3.91 (s, 3H), 3.90 (s, 3H), 3.88 (s, 3H), 3.28-3.20 (m, 1H), 2.96 (dd, *J* = 17.4, 7.8 Hz, 1H), 2.36 (dd, *J* = 17.4, 1.8 Hz, 1H), 0.68 (d, *J* = 7.2 Hz, 3H).

^{13}C NMR (150 MHz, $CDCl_3$) δ 176.2, 152.9, 150.7, 142.5, 131.0, 106.7, 106.0, 83.4, 61.0, 61.0, 56.2, 37.5, 32.5, 15.3.

HRMS (ESI) calculated for $C_{14}H_{17}BrNaO_5$ [M+Na$^+$]: 367.0152, 369.0131, found: 367.0154, 369.0131.

FT-IR (cm^{-1}): 3543, 2937, 1779, 1487, 1398, 1327, 1202, 1106, 1006, 940.

MS (EI) *m/z* (%): 344 (100), 146 (95), 276 (56), 275 (55), 274 (53), 277 (46), 169 (28), 259 (20), 261 (18).

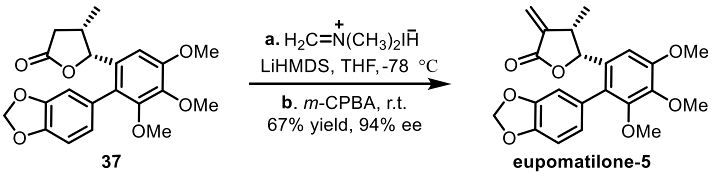

eupomatilone-5: Compound **38** was prepared according to the literature.[9] To a solution of **37** (20 mg, 0.052 mmol) in dry THF (0.02 M, 2.6 mL) under argon atmosphere at -78 °C, Lithium bis(trimethylsilyl)amide (1.0 M in THF, 105 μL, 2.0 equiv) was added. Eschenmoser's Salt (28.7 mg, 0.155 mmol, 3.0 equiv) was added after the reaction mixture was stirred for 1 h at -78 °C. The resulting mixture was stirred for 1 h at –78 °C before it was quenched with the addition of saturated aqueous $NaHCO_3$ and diluted with DCM. The solution was extracted with DCM. The combined organic layers were washed with brine, dried ($NaSO_4$), concentrated in vacuo. The residue was dissolved in DCM (0.01 M, 5.2 mL), saturated aqueous $NaHCO_3$ (0.02 M, 2.6 mL) and 85% *m*-CPBA (21.0 mg, 0.10

mmol, 2.0 equiv) was added at room temperature. The reaction mixture was stirred for 10 min before it was diluted with saturated aqueous $NaHCO_3$. The organic layers were separated, and the aqueous layer was extracted with DCM. The combined organic layers were washed again with saturated aqueous $NaHCO_3$ and brine, dried ($NaSO_4$) and concentrated to afford a residue, which on purification by column chromatography on silica gel to afford **eupomatilone-5** (13.8 mg, 67% yield, as a mixture of atropdiastereomers) as a colorless liquid, 94.1% ee.

[α]$_D^{21}$ = +31.0 (c = 1.0, $CHCl_3$).

^{1}H NMR (600 MHz, $CDCl_3$) δ 6.87 (dd, *J* = 7.8, 1.2 Hz, 2H), 6.73 (d, *J* = 1.2 Hz, 1H), 6.70 (dd, *J* = 7.8, 1.8 Hz, 1H), 6.69 (s, 2H), 6.64 (d, *J* = 1.8 Hz, 1H), 6.59 (dd, *J* = 7.8, 1.8 Hz, 1H), 6.24 (d, *J* = 1.8 Hz, 2H), 6.07-5.99 (m, 4H), 5.58-5.49 (m, 3H), 5.44 (d, *J* = 7.2 Hz, 1H), 3.91 (s, 6H), 3.88 (s, 6H), 3.65 (s, 3H), 3.64 (s, 3H), 2.91-2.82 (m, 2H), 0.82 (d, *J* = 7.3 Hz, 3H), 0.80 (d, *J* = 7.8 Hz, 3H).

^{13}C NMR (150 MHz, $CDCl_3$) δ 170.2, 170.1, 152.9, 151.5, 147.7, 147.7, 147.1, 147.0, 141.8, 141.2, 141.1, 130.1, 130.1, 129.0, 127.1, 123.3, 122.7, 122.0, 110.6, 109.9, 108.5, 108.2, 104.8, 104.8, 101.2, 101.2, 79.4, 79.3, 61.2, 61.1, 60.9, 56.1, 38.4, 38.2, 17.3, 17.1.

HRMS (ESI) calculated for $C_{22}H_{22}NaO_7$ [M+Na$^+$]: 421.1258, found: 421.1259

FT-IR (cm^{-1}): 2935, 1767, 1483, 1458, 1324, 1236, 1128, 1037, 970.

MS (EI) *m/z* (%): 299 (100), 398 (85), 284 (25), 300 (22), 399 (22).

The ee value was determined by the chiral HPLC analysis (CHIRALPAK® IC-3, Hexane/ Isopropanol = 80/20, v = 1.0 mL/min, λ = 208.0 nm), t (major) = 14.616, t (minor) = 17.428.

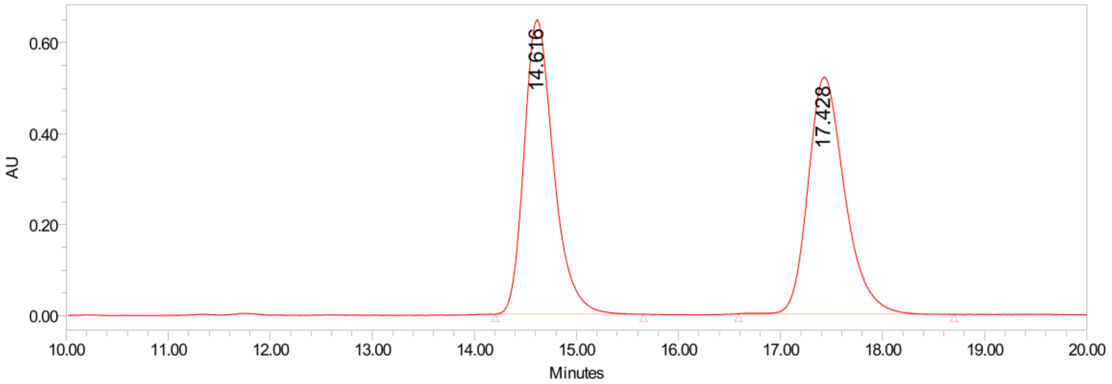

peak information

	RetTime (min)	Area (μV*s)	Area (%)	Height (μV)
1	14.616	13027170	49.95	648154
2	17.428	13053013	50.05	521837

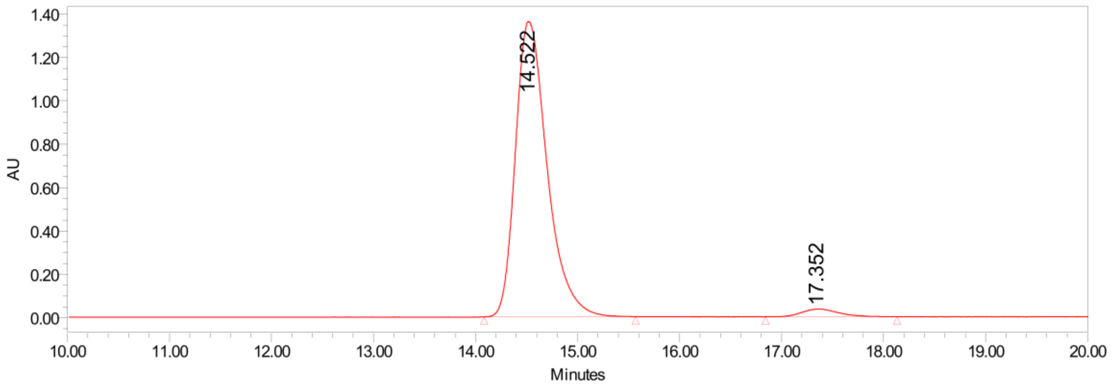

peak information

	RetTime (min)	Area (μV*s)	Area (%)	Height (μV)
1	14.522	29444388	97.05	1362231
2	17.352	896372	2.95	35906

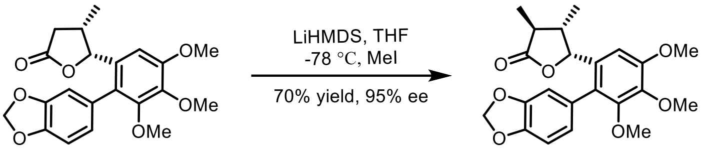

37 **eupomatilone-6**

eupomatilone-6: Compound **39** was prepared according to the literature.[9c] To a solution of **37** (20 mg, 0.052 mmol) in dry THF (0.05 M, 1.0 mL) under argon atmosphere at -78 °C, Lithium bis(trimethylsilyl)amide (1.0 M in THF, 105 μL, 2.0 equiv) was added. MeI (4.8 μL, 0.078 mmol, 1.5 equiv) was added after the reaction mixture was stirred for 1 h at -78 °C. The resulting mixture was stirred for 1 h at –78 °C before it was quenched with the addition of saturated aqueous NH_4Cl and diluted with EA. The solution was extracted with DCM. The combined organic layers were washed with brine, dried ($NaSO_4$), concentrated in vacuo. The crude product was purified by flash column chromatography on silica gel to afford **eupomatilone-6** (14.5 mg, 70% yield, as a mixture of atropdiastereomers) as a colorless liquid, 94.8% ee.

$[\alpha]_D^{21}$ = -23.0 (c = 1.0, $CHCl_3$).

^{1}H NMR (600 MHz, $CDCl_3$) δ 6.88 (d, *J* = 5.4 Hz, 1H), 6.86 (d, *J* = 4.8 Hz, 1H), 6.73 (d, *J* = 1.8 Hz, 1H), 6.70 (dd, *J* = 8.1, 1.5 Hz, 1H), 6.67 (d, *J* = 4.2 Hz, 2H), 6.65 (d, *J* = 1.8 Hz, 1H), 6.59 (dd, *J* = 7.8, 1.8 Hz, 1H), 6.03 (dd, *J* = 3.6, 1.2 Hz, 2H), 6.02 (dd, *J* = 4.2, 1.8 Hz, 2H), 5.64 (d, *J* = 7.2 Hz, 1H), 5.53 (d, *J* = 7.2 Hz, 1H), 3.90 (s, 6H), 3.89 (s, 6H), 3.64 (s, 3H), 3.63 (s, 3H), 2.40-2.32 (m, 2H), 2.06-1.93 (m, 2H), 1.20 (d, *J* = 6.6 Hz, 3H), 1.19 (d, *J* = 6.6 Hz, 3H), 0.72 (d, *J* = 7.2 Hz, 3H), 0.69 (d, *J* = 7.2 Hz, 3H).

^{13}C NMR (150 MHz, $CDCl_3$) δ 179.7, 152.9, 151.6, 147.7, 147.6, 146.9, 146.9, 141.8, 141.8, 130.3, 130.3, 129.1, 129.0, 127.2, 127.1, 123.8, 122.7, 111.0, 109.9, 108.4, 108.1, 104.7, 104.7, 101.2, 101.1, 79.9, 79.8, 61.2, 61.1, 60.9, 56.2, 42.7, 42.5, 41.5, 41.2, 15.5, 15.3, 15.0, 14.8.

HRMS (ESI) calculated for $C_{22}H_{24}NaO_7$ $[M+Na^+]$: 423.1414, found: 423.1415.

FT-IR (cm^{-1}): 2968, 2935, 1774, 1483, 1457, 1325, 1236, 1197, 1128, 1038, 1005, 937.

MS (EI) *m/z* (%): 400 (100), 316 (30), 299 (25), 401 (25), 341 (20), 243 (10).

The ee value was determined by the chiral HPLC analysis (CHIRALPAK® IC-3, Hexane/ Isopropanol = 80/20, v = 1.0 mL/min, λ = 208.0 nm), t (major) = 12.787, t (minor) = 16.696.

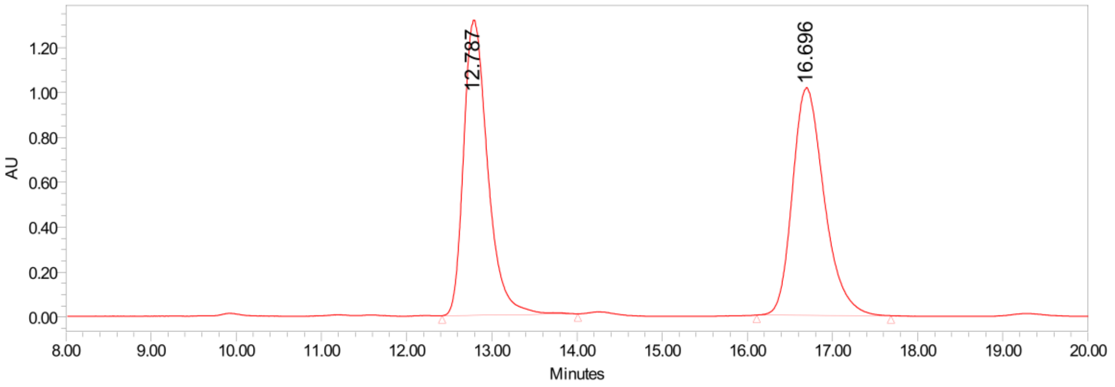

peak information

	RetTime (min)	Area (µV*s)	Area (%)	Height (µV)
1	12.787	25892953	49.65	1314846
2	16.696	26260845	50.35	1014760

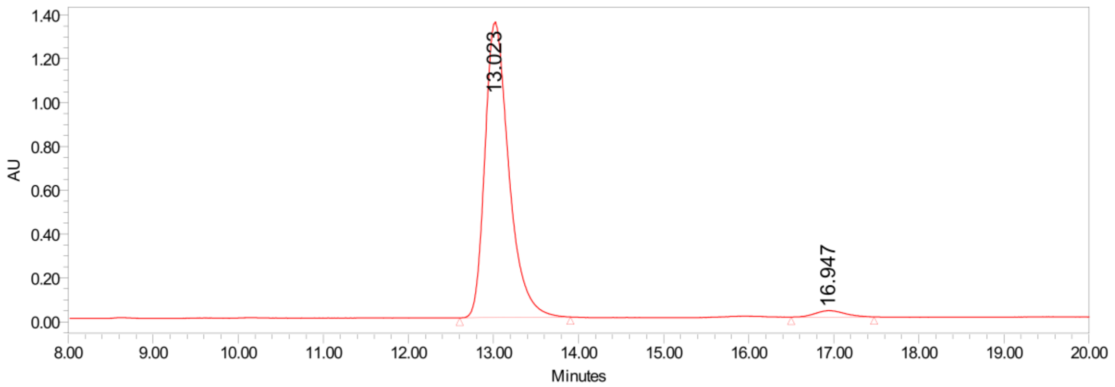

peak information

	RetTime (min)	Area (µV*s)	Area (%)	Height (µV)
1	13.023	26381381	97.43	1349896
2	16.947	695256	2.57	29148

11. A possible mechanism for Cu/SPDO catalyzed Baeyer–Villiger oxidation

Based on the reported B–V oxidation reaction in literature and the absolute configuration of four products (**6b**, **16b**, **21b**, and **28b**; CCDC Nos. 2169290, 2169293, 2164219, and 2164218, respectively), a preliminary model of classical kinetic resolution of 2-aryl-substituted cyclobutanones through B–V oxidation catalysed using a Cu(II)/SPDO system is proposed (Scheme R1). Initially, 2-aryl cyclobutanone could be activated by chiral Cu-SPDO complex and then reacted with 3-chloroperoxybenzoic acid, and two possible Criegee intermediates **A** and **A′** were proposed. For the intermediate **A** derived from enantiomer **(*S*)-1a**, due to the π-π stacking interactions between the substrate and the SPDO ligand, the leaving group of peroxide acid in this transition state is oriented antiperiplanar to the adjacent migrating group, which accelerated the subsequent alkyl migration (Scheme R1a). Hence this favoured intermediate **A′** easily generated lactone **b** and by-product 3-chlorobenzoic acid through a rearrangement procedure and simultaneously released the chiral Cu-catalyst for the next catalytic cycle. However, for the latter, because of the lack of possible π-π stacking interactions between the chiral catalyst and enantiomer **(*R*)-1a** and suitable orientation between the leaving group and migrating group in the expected rearrangement reaction, this unfavoured intermediate **A′** remained unreacted under the optimal reaction conditions, and then released the ketone **(*R*)-1a** in a high enantioselective fashion after quenching with Na_2SO_3 solution (Scheme S1).

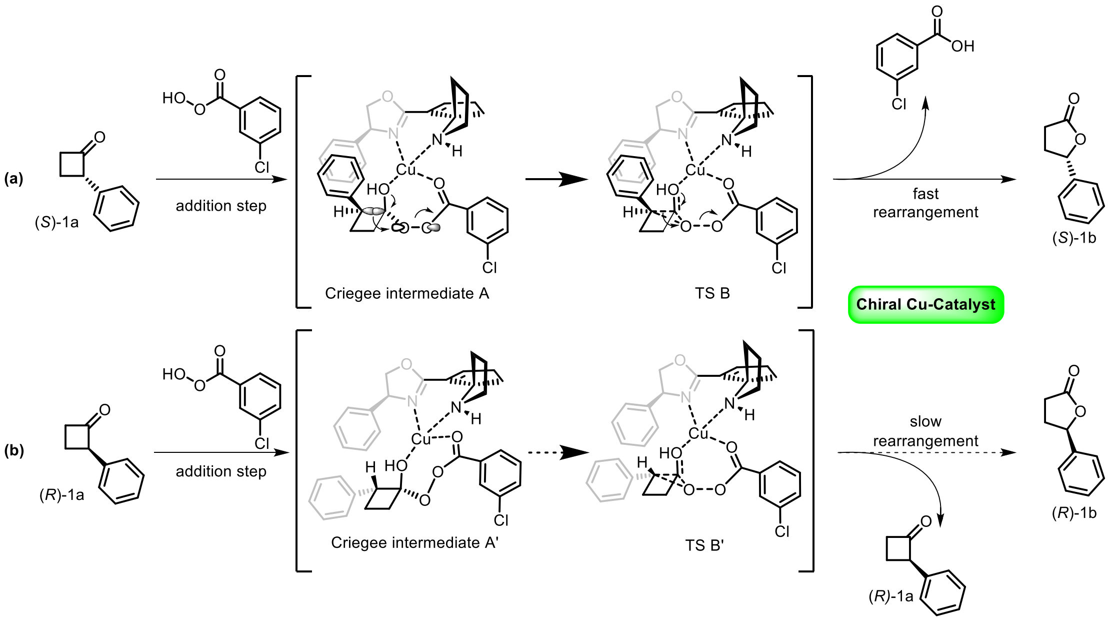

Scheme S1. A possible mechanism for Cu/SPDO catalyzed Baeyer–Villiger oxidation

12. References

[1] (a) J.-M. Tian, A.-F. Wang, J.-S. Yang, X.-J. Zhao, Y.-Q. Tu, S.-Y. Zhang and Z.-M. Chen, *Angew. Chem., Int. Ed.*, 2019, **58**, 11023; (b) X.-J. Zhao, Z.-H. Li, T.-M. Ding, J.-M. Tian, Y-Q Tu, A.-F. Wang and Y.-Y. Xie, *Angew. Chem., Int. Ed*., 2021, **60**, 7061.

[2] Y. Wang, M. E. Muratore, Z. Rong and A. M. Echavarren, *Angew. Chem., Int. Ed.*, 2014, **53**, 14022.

[3] X. Zhao, M. Tian, L. Ji, J. Liu and K. Lu, *Org. Lett*., 2020, **22**, 863.

[4] C. Battilocchio, G. Iannucci, S. Wang, E. Godineau, A. Kolleth, A. D. Mesmaeker and S. V. Ley, *React. Chem. Eng*., 2017, **2**, 295.

[5] C. Aïssa, D. Crépin, D. J. Tetlow and K. Y. T. Ho, *Org. Lett.*, 2013, **15**, 1322.

[6] M. M. Hussain, H. Li, N. Hussain, M. Ureña, P. J. Carroll and P. J. Walsh, *J. Am. Chem. Soc.*, 2009, **131**, 6516.

[7] L. Guillaume, F. Josep, P. Teodor, A. Ramon and F. Marta, *J. Org. Chem*., 2015, **80**, 9437.

[8] M. K. Gurjar, J. Cherian and C. V. Ramana, *Org. Lett*., 2004, **6**, 317.

[9] (a) T. S. Kaoud, H. Park, S. Mitra, C. Yan, C.-C. Tseng, Y. Shi, J. Jose, J. M. Taliaferro, K. Lee, P. Ren, J. Hong and K. N. Dalby, *ACS Chem. Biol.*, 2012, **7**, 1873. (b) J. Schreiber, H. Maag, N. Hashimoto and A. Eschenmoser, *Angew. Chem., Int. Ed.*, 1971, **10**, 330. (c) M. Mandal, H. Yun, G. B. Dudley, S. Lin, D. S. Tan and S. J. Danishefsky, *J. Org. Chem.*, 2005, **70**, 10619.

13. X-ray Crystallographic Data

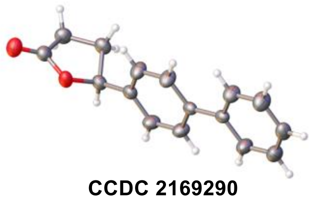

CCDC 2169290

**Table S7. Crystal data and structure refinement for 6b (CCDC 2169290)**	
Identification code	**6b**
Empirical formula	$C_{16}H_{14}O_2$
Formula weight	238.27
Temperature/K	149.99(10)
Crystal system	orthorhombic
Space group	P212121
a/Å	5.2470(2)
b/Å	8.2089(2)
c/Å	28.1307(9)
α/°	90
β/°	90
γ/°	90
Volume/Å^3	1211.65(7)
Z	4
ρcalcg/cm^3	1.306
μ/mm^{-1}	0.679
F(000)	504.0
Crystal size/mm^3	0.18 × 0.15 × 0.12
Radiation	Cu Kα (λ = 1.54184)
2Θ range for data collection/°	6.284 to 154.806
Index ranges	-6 ≤ h ≤ 6, -10 ≤ k ≤ 10, -30 ≤ l ≤ 34
Reflections collected	2834
Independent reflections	2834 [R_{int} = ?, R_{sigma} = 0.0166]
Data/restraints/parameters	2834/0/164
Goodness-of-fit on F^2	1.112
Final R indexes [I>=2σ (I)]	R_1 = 0.0687, wR_2 = 0.1927
Final R indexes [all data]	R_1 = 0.0727, wR_2 = 0.1994
Largest diff. peak/hole / e Å^{-3}	0.32/-0.37
Flack parameter	-0.3(3)

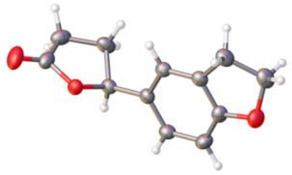

CCDC 2169293

**Table S7. Crystal data and structure refinement for 16b (CCDC 2169293)**	
Identification code	**16b**
Empirical formula	$C_{12}H_{12}O_3$
Formula weight	204.22
Temperature/K	150.00(10)
Crystal system	orthorhombic
Space group	P212121
a/Å	8.7960(2)
b/Å	10.4133(2)
c/Å	10.7857(2)
α/°	90
β/°	90
γ/°	90
Volume/Å^3	987.92(3)
Z	4
ρcalcg/cm^3	1.373
μ/mm^{-1}	0.809
F(000)	432.0
Crystal size/mm^3	0.17 × 0.14 × 0.12
Radiation	Cu Kα (λ = 1.54184)
2Θ range for data collection/°	11.814 to 154.708
Index ranges	$-5 \leq h \leq 10$, $-13 \leq k \leq 12$, $-12 \leq l \leq 13$
Reflections collected	3439
Independent reflections	1765 [R_{int} = 0.0201, R_{sigma} = 0.0231]
Data/restraints/parameters	1765/0/136
Goodness-of-fit on F^2	0.877
Final R indexes [I>=2σ (I)]	R_1 = 0.0298, wR_2 = 0.0924
Final R indexes [all data]	R_1 = 0.0307, wR_2 = 0.0941
Largest diff. peak/hole / e Å^{-3}	0.18/-0.13
Flack parameter	-0.06(9)

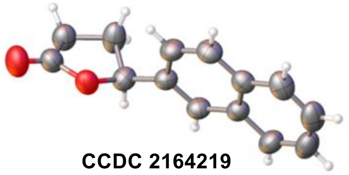

CCDC 2164219

**Table S5. Crystal data and structure refinement for 21b (CCDC 2164219)**	
Identification code	**21b**
Empirical formula	$C_{14}H_{12}O_2$
Formula weight	212.24
Temperature/K	298.23(10)
Crystal system	orthorhombic
Space group	$P2_12_12_1$
a/Å	8.67930(10)
b/Å	22.7043(3)
c/Å	11.04130(10)
α/°	90
β/°	90
γ/°	90
Volume/Å^3	2175.77(4)
Z	4
ρ_{calc}g/cm^3	1.296
μ/mm^{-1}	0.689
F (000)	896.0
Crystal size/mm^3	0.18 × 0.15 × 0.12
Radiation	Cu Kα (λ = 1.54184)
2Θ range for data collection/°	8.906 to 151.824
Index ranges	-10 ≤ h ≤ 5, -28 ≤ k ≤ 28, -13 ≤ l ≤ 13
Reflections collected	11192
Independent reflections	4292 [R_{int} = 0.0235, R_{sigma} = 0.0235]
Data/restraints/parameters	4292/0/289
Goodness-of-fit on F^2	1.067
Final R indexes [I>=2σ (I)]	R1 = 0.0368, wR2 = 0.1012
Final R indexes [all data]	R1 = 0.0393, wR2 = 0.1027
Largest diff. peak/hole / e Å^{-3}	0.13/-0.14
Flack parameter	0.10(9)

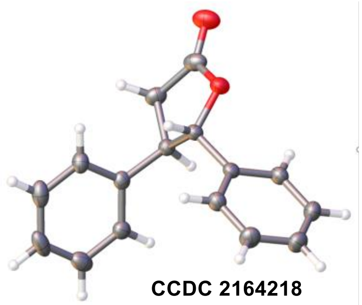

CCDC 2164218

Table S6. Crystal data and structure refinement for 28b (CCDC 2164218)	
Identification code	**28b**
Empirical formula	$C_{16}H_{14}O_2$
Formula weight	238.27
Temperature/K	150.01(10)
Crystal system	orthorhombic
Space group	$P2_12_12_1$
a/Å	5.91070(10)
b/Å	13.8035(3)
c/Å	15.3174(3)
α/°	90
β/°	90
γ/°	90
Volume/Å^3	1249.72(4)
Z	4
ρcalcg/cm^3	1.266
μ/mm^{-1}	0.658
F(000)	504.0
Crystal size/mm^3	0.15 × 0.12 × 0.04
Radiation	Cu Kα (λ = 1.54184)
2Θ range for data collection/°	8.624 to 154.91
Index ranges	$-7 \leq h \leq 7$, $-16 \leq k \leq 17$, $-18 \leq l \leq 14$
Reflections collected	6468
Independent reflections	2485 [R_{int} = 0.0289, R_{sigma} = 0.0260]
Data/restraints/parameters	2485/0/163
Goodness-of-fit on F^2	1.065
Final R indexes [I>=2σ (I)]	R_1 = 0.0468, wR_2 = 0.1244
Final R indexes [all data]	R_1 = 0.0481, wR_2 = 0.1256
Largest diff. peak/hole / e Å^{-3}	0.66/-0.26
Flack parameter	-0.19(15)

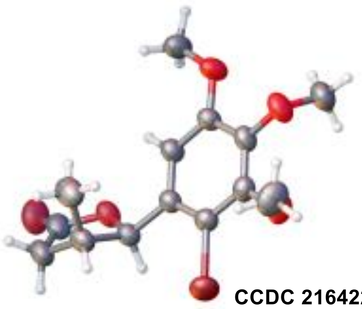

Table S7. Crystal data and structure refinement for 36b (CCDC 2164220)	
Identification code	**36b**
Empirical formula	$C_{14}H_{17}BrO_5$
Formula weight	345.18
Temperature/K	303.15(10)
Crystal system	triclinic
Space group	P-1
a/Å	7.8803(2)
b/Å	9.7157(2)
c/Å	10.6212(2)
α/°	94.2410(10)
β/°	94.434(2)
γ/°	111.794(2)
Volume/Å^3	748.16(3)
Z	2
ρcalcg/cm^3	1.532
μ/mm^{-1}	3.900
F(000)	352.0
Crystal size/mm^3	0.13 × 0.11 × 0.09
Radiation	Cu Kα (λ = 1.54184)
2Θ range for data collection/°	8.402 to 152.902
Index ranges	$-9 \le h \le 9$, $-12 \le k \le 12$, $-12 \le l \le 13$
Reflections collected	8578
Independent reflections	2995 [R_{int} = 0.0248, R_{sigma} = 0.0229]
Data/restraints/parameters	2995/0/185
Goodness-of-fit on F^2	1.068
Final R indexes [I>=2σ (I)]	R_1 = 0.0306, wR_2 = 0.0819
Final R indexes [all data]	R_1 = 0.0327, wR_2 = 0.0832
Largest diff. peak/hole / e Å^{-3}	0.46/-0.49

14. ^{1}H NMR, ^{13}C NMR and ^{19}F NMR Spectra of the Compounds

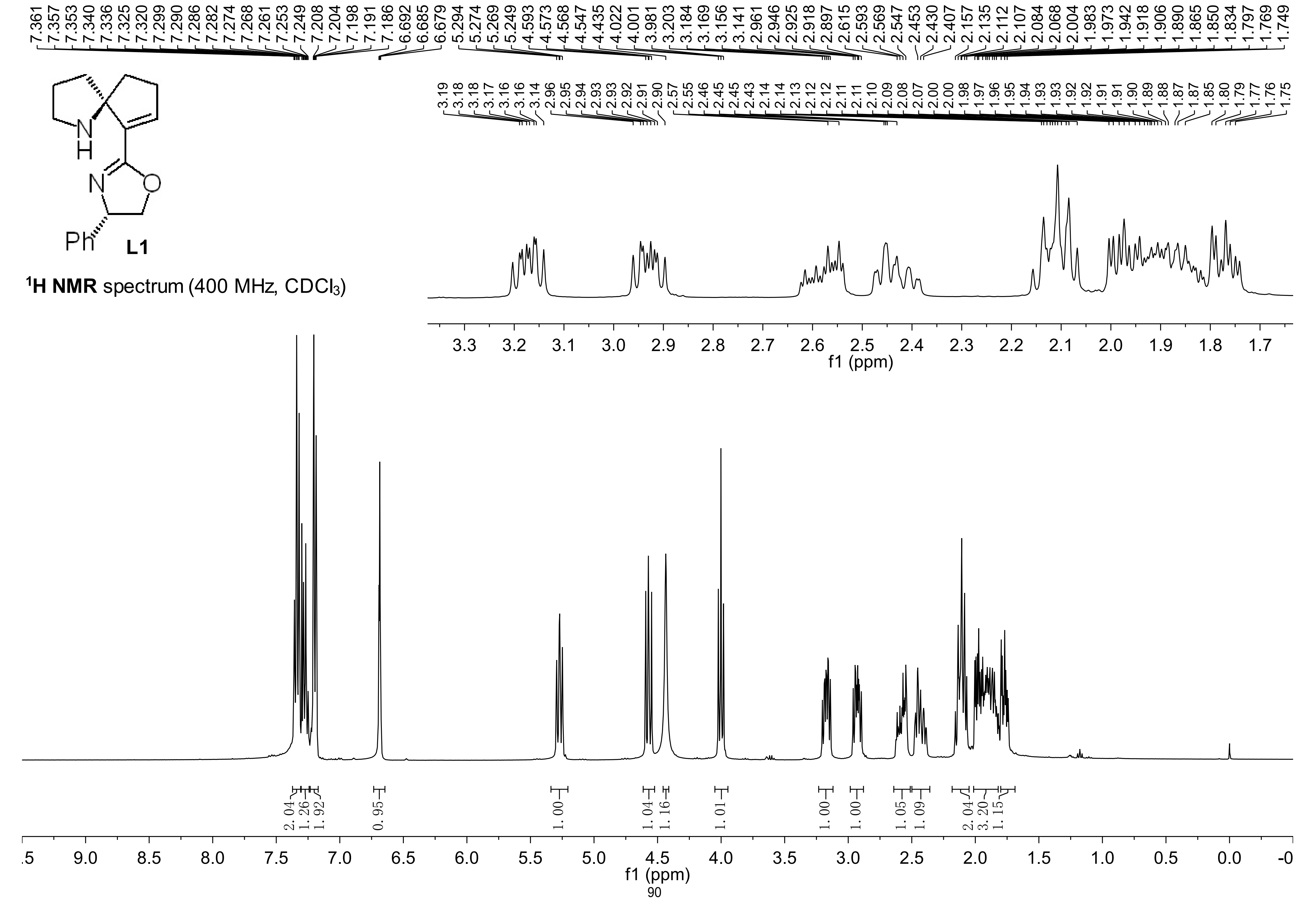

¹H NMR spectrum (400 MHz, $CDCl_3$)

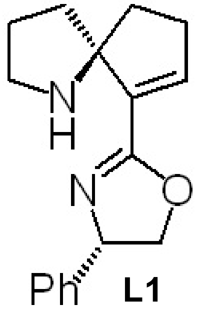

L1

^{13}C NMR spectrum (100 MHz, $CDCl_3$)

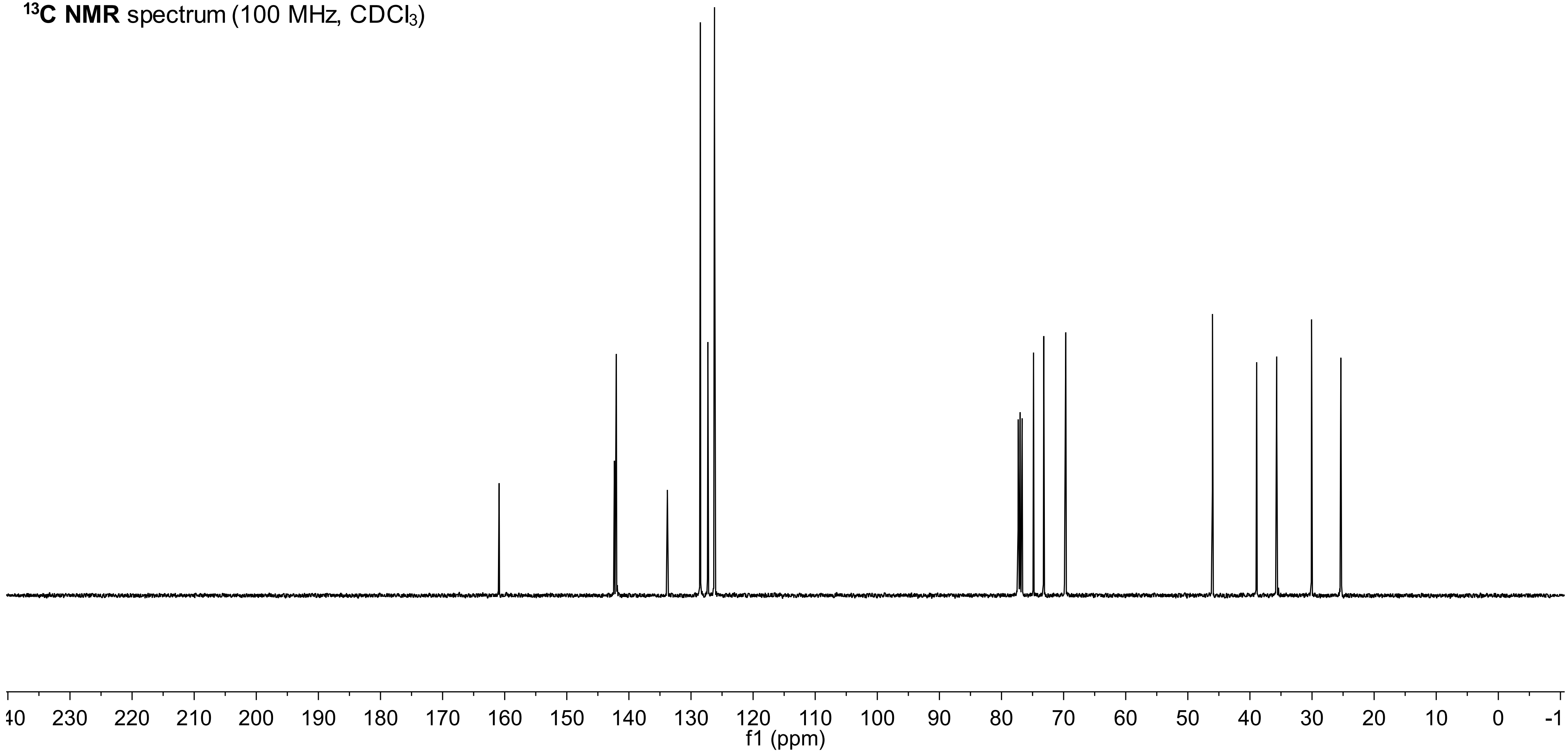

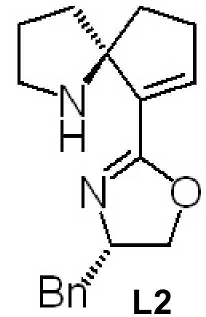

L2

^{1}H NMR spectrum (600 MHz, $CDCl_3$)

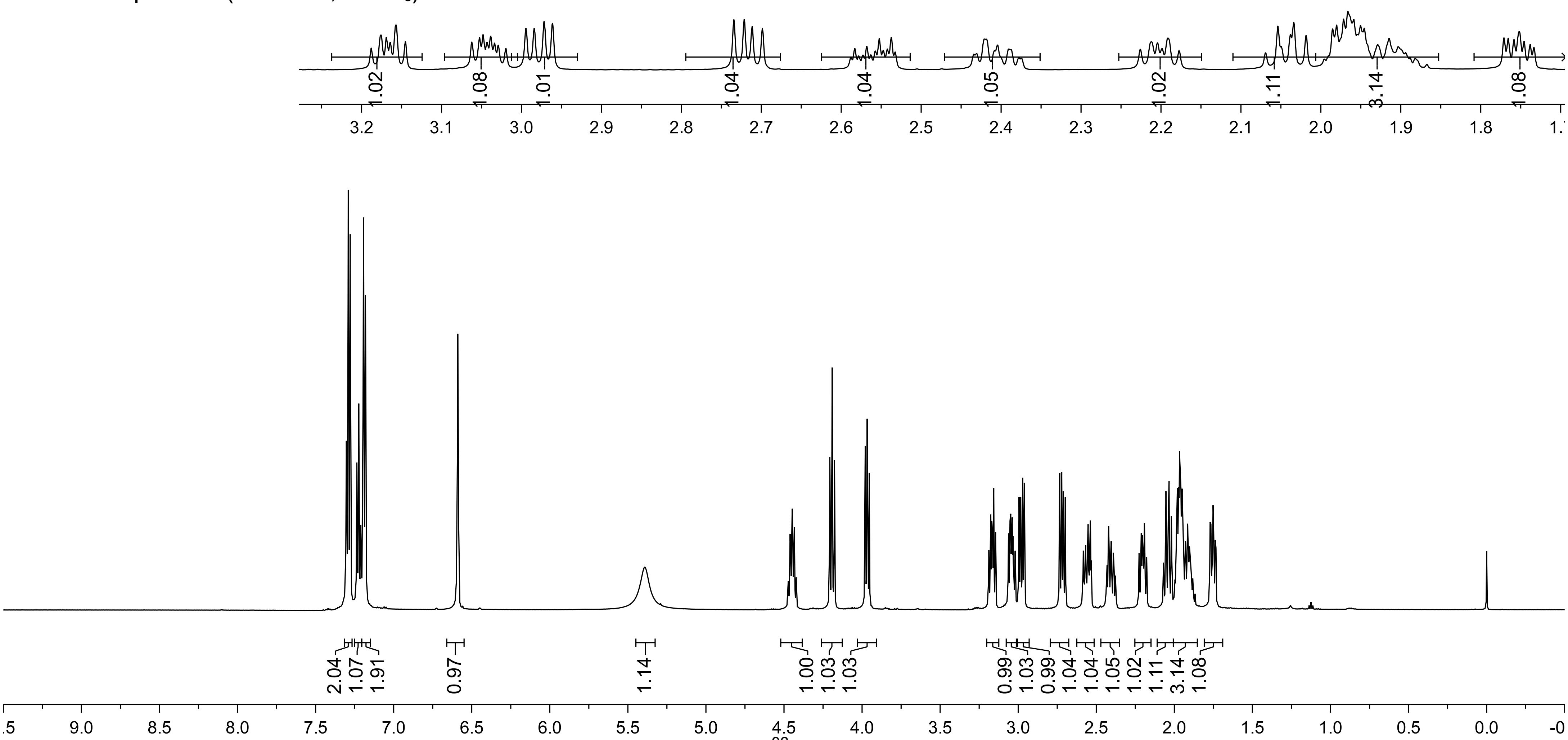

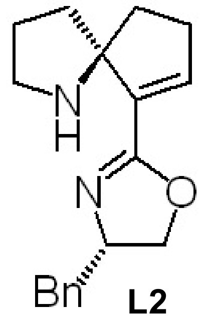

L2

^{13}C NMR spectrum (150 MHz, $CDCl_3$)

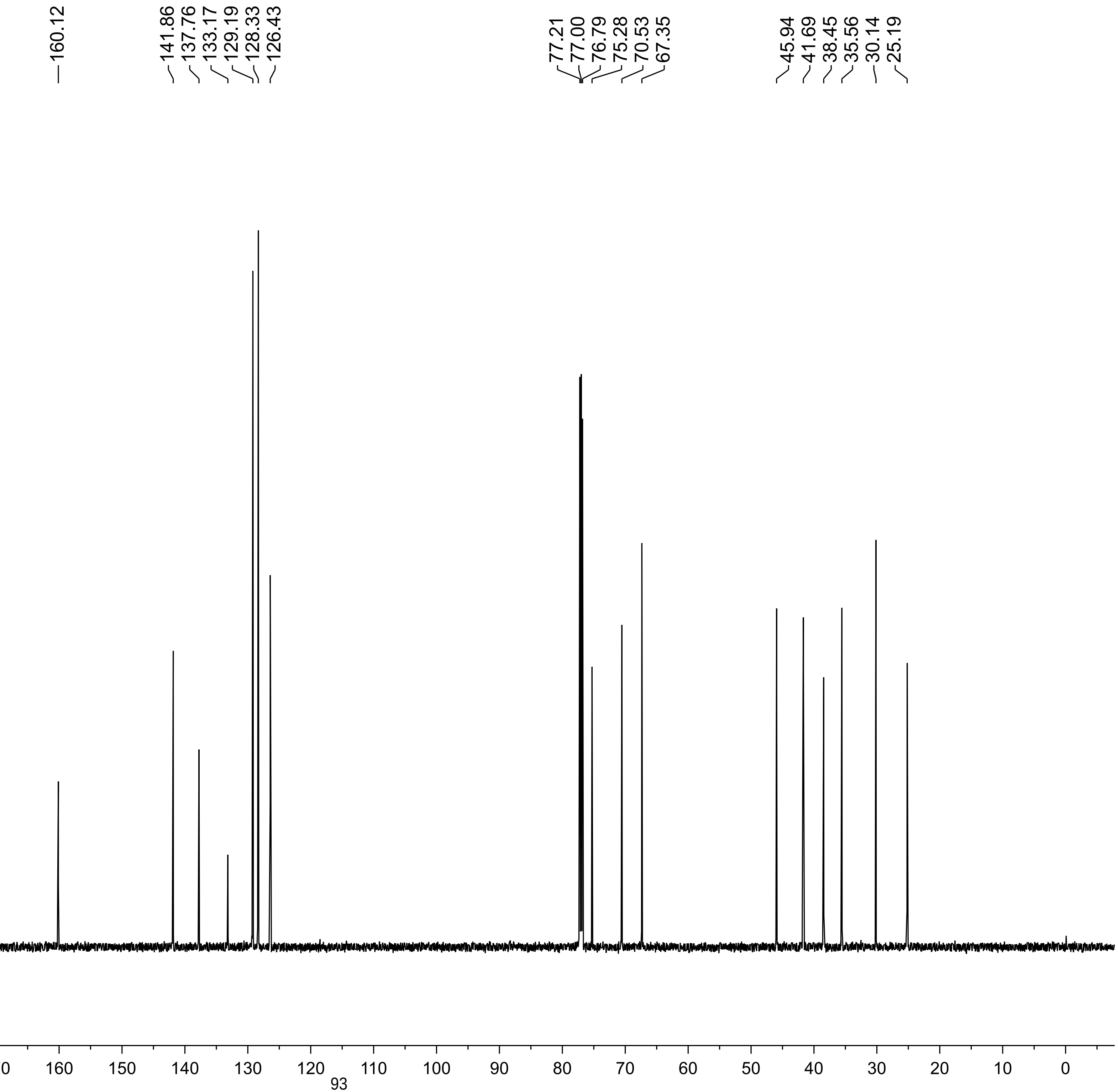

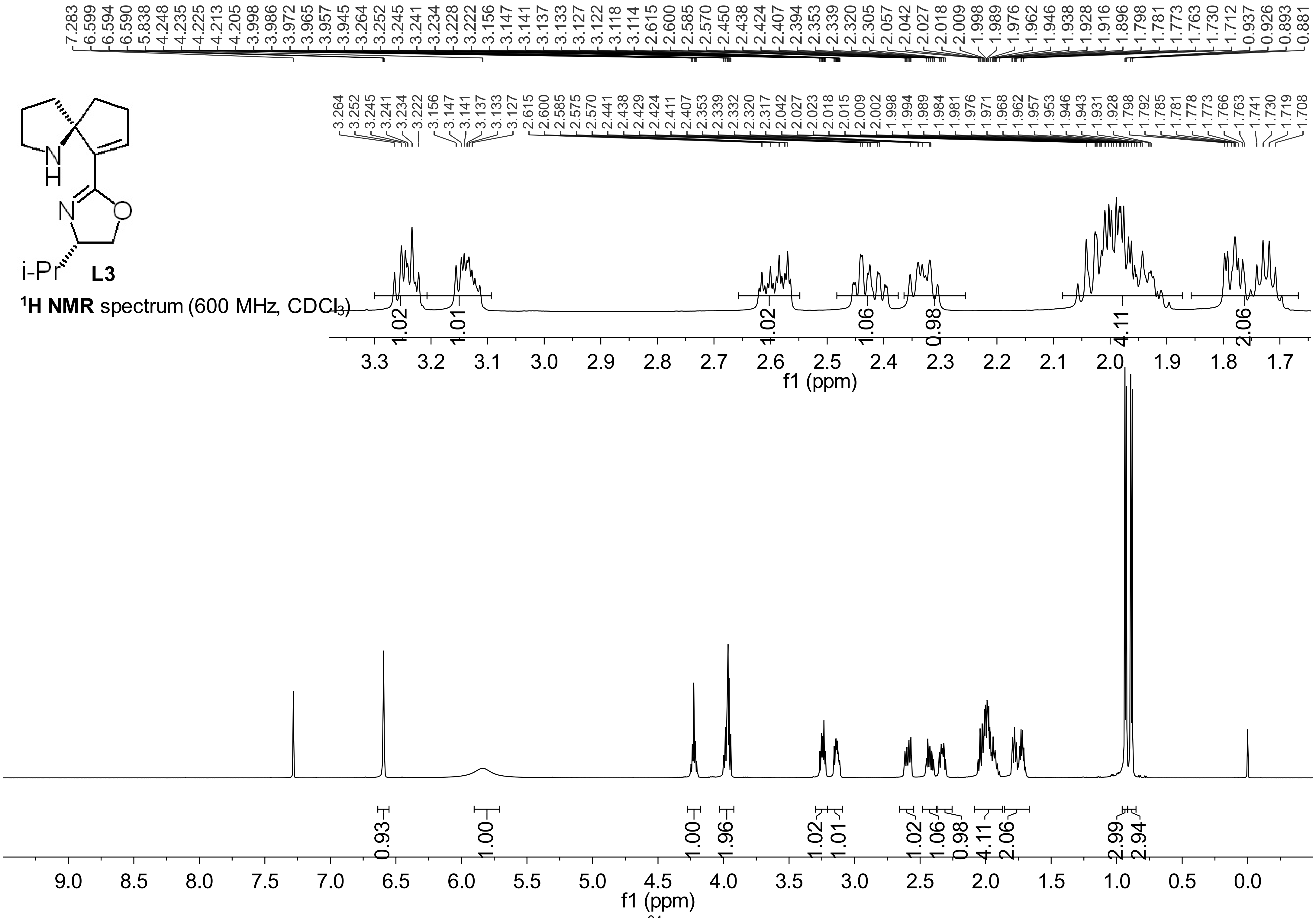
7.283 6.599 6.594 6.590 5.838 4.248 4.235 4.225 4.213 4.205 3.998 3.986 3.972 3.965 3.957 3.945 3.264 3.252 3.245 3.241 3.234 3.228 3.222 3.156 3.147 3.141 3.137 3.133 3.127 3.122 3.118 3.114 2.615 2.600 2.585 2.570 2.450 2.438 2.424 2.407 2.394 2.353 2.339 2.320 2.305 2.057 2.042 2.027 2.018 2.009 1.998 1.989 1.976 1.962 1.946 1.938 1.928 1.916 1.896 1.798 1.781 1.773 1.763 1.730 1.712 0.937 0.926 0.893 0.881
3.264 3.252 3.245 3.241 3.234 3.222 3.156 3.147 3.141 3.137 3.133 3.127 2.615 2.600 2.585 2.575 2.570 2.441 2.438 2.429 2.424 2.411 2.407 2.353 2.339 2.332 2.320 2.317 2.042 2.027 2.023 2.018 2.015 2.009 2.002 1.998 1.994 1.989 1.984 1.981 1.976 1.971 1.968 1.962 1.957 1.953 1.946 1.943 1.931 1.928 1.798 1.792 1.785 1.781 1.778 1.773 1.766 1.763 1.741 1.730 1.719 1.708
N
H
N
O
i-Pr
L3
^{1}H NMR spectrum (600 MHz, $CDCl_3$)
1.02 1.01 1.02 1.06 0.98 4.11 2.06
3.3 3.2 3.1 3.0 2.9 2.8 2.7 2.6 2.5 2.4 2.3 2.2 2.1 2.0 1.9 1.8 1.7
f1 (ppm)
0.93 1.00 1.00 1.96 1.02 1.01 1.02 1.06 0.98 4.11 2.06 2.99 2.94
9.0 8.5 8.0 7.5 7.0 6.5 6.0 5.5 5.0 4.5 4.0 3.5 3.0 2.5 2.0 1.5 1.0 0.5 0.0
f1 (ppm)

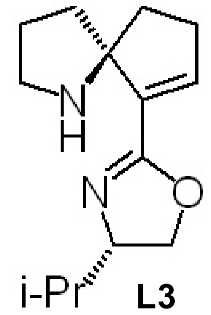

L3

^{13}C NMR spectrum (150 MHz, $CDCl_3$)

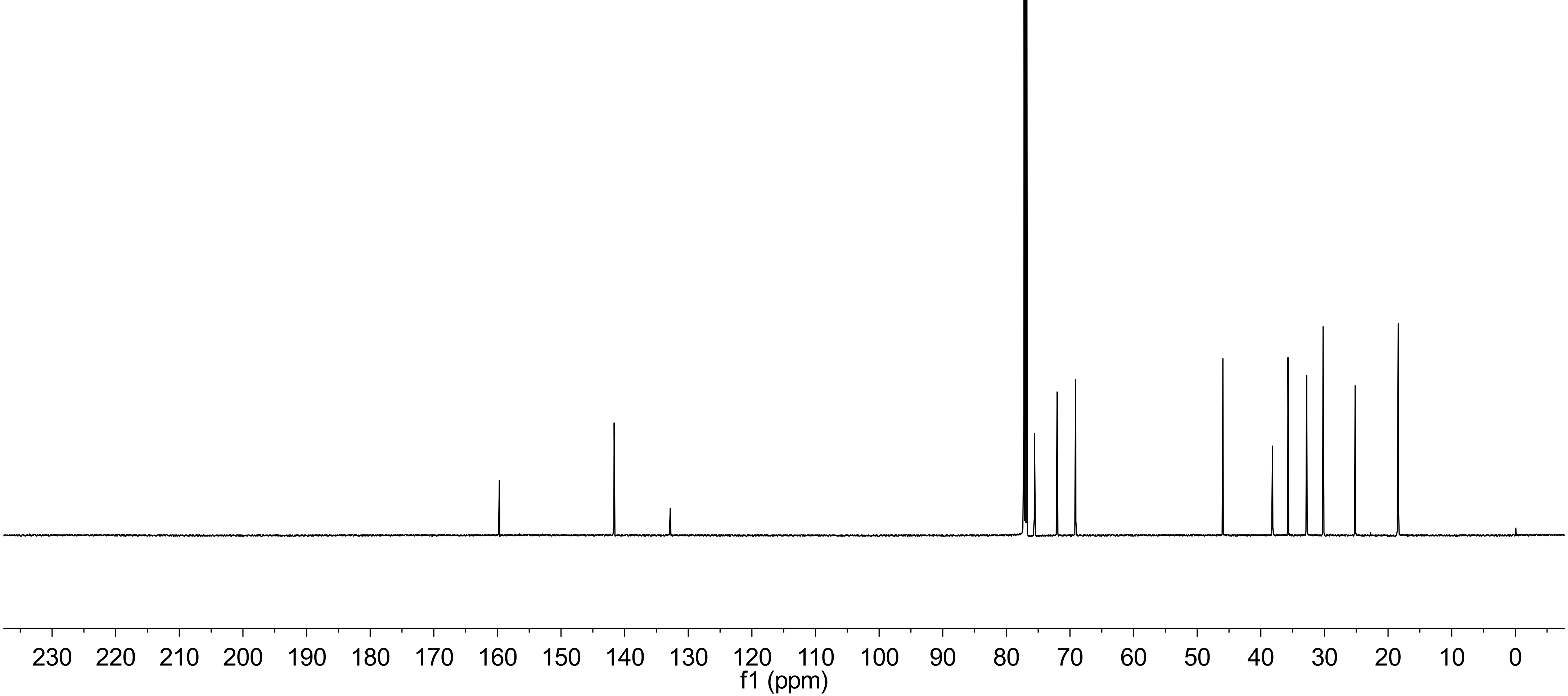

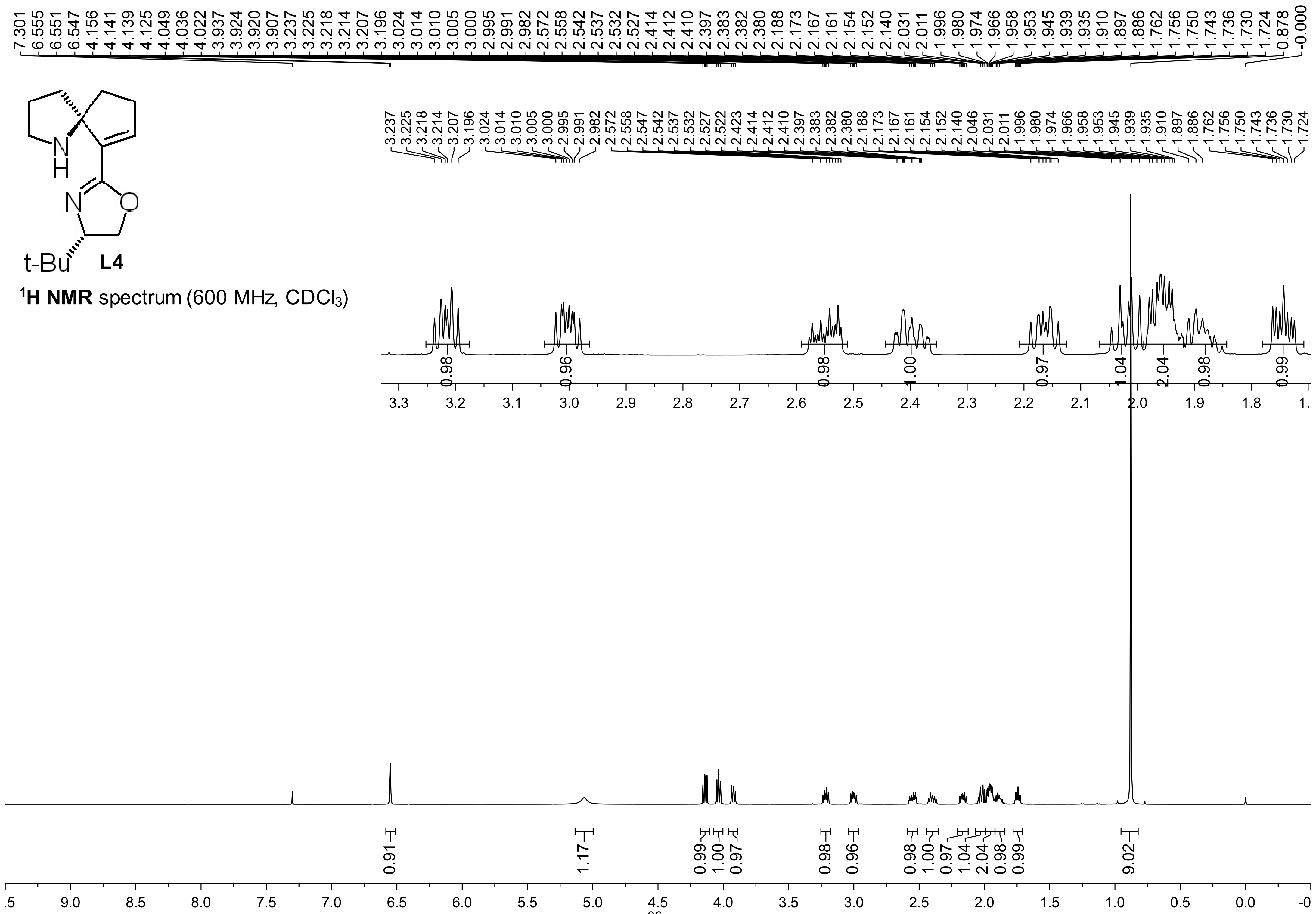

L4

^{1}**H NMR** spectrum (600 MHz, $CDCl_3$)

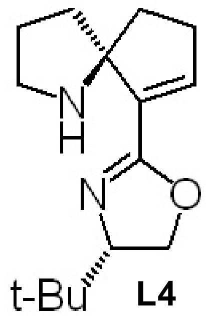

L4

^{13}C NMR spectrum (150 MHz, $CDCl_3$)

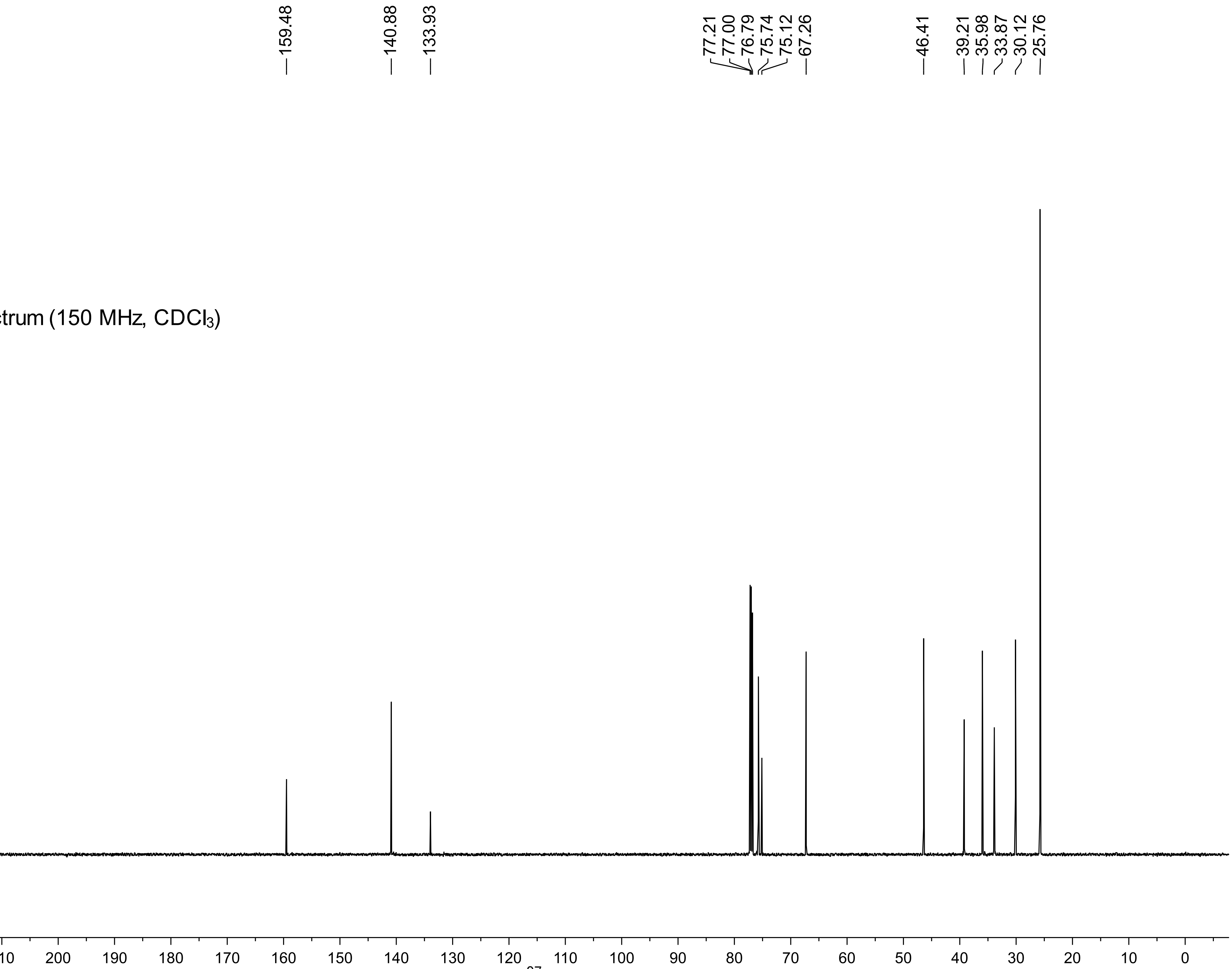

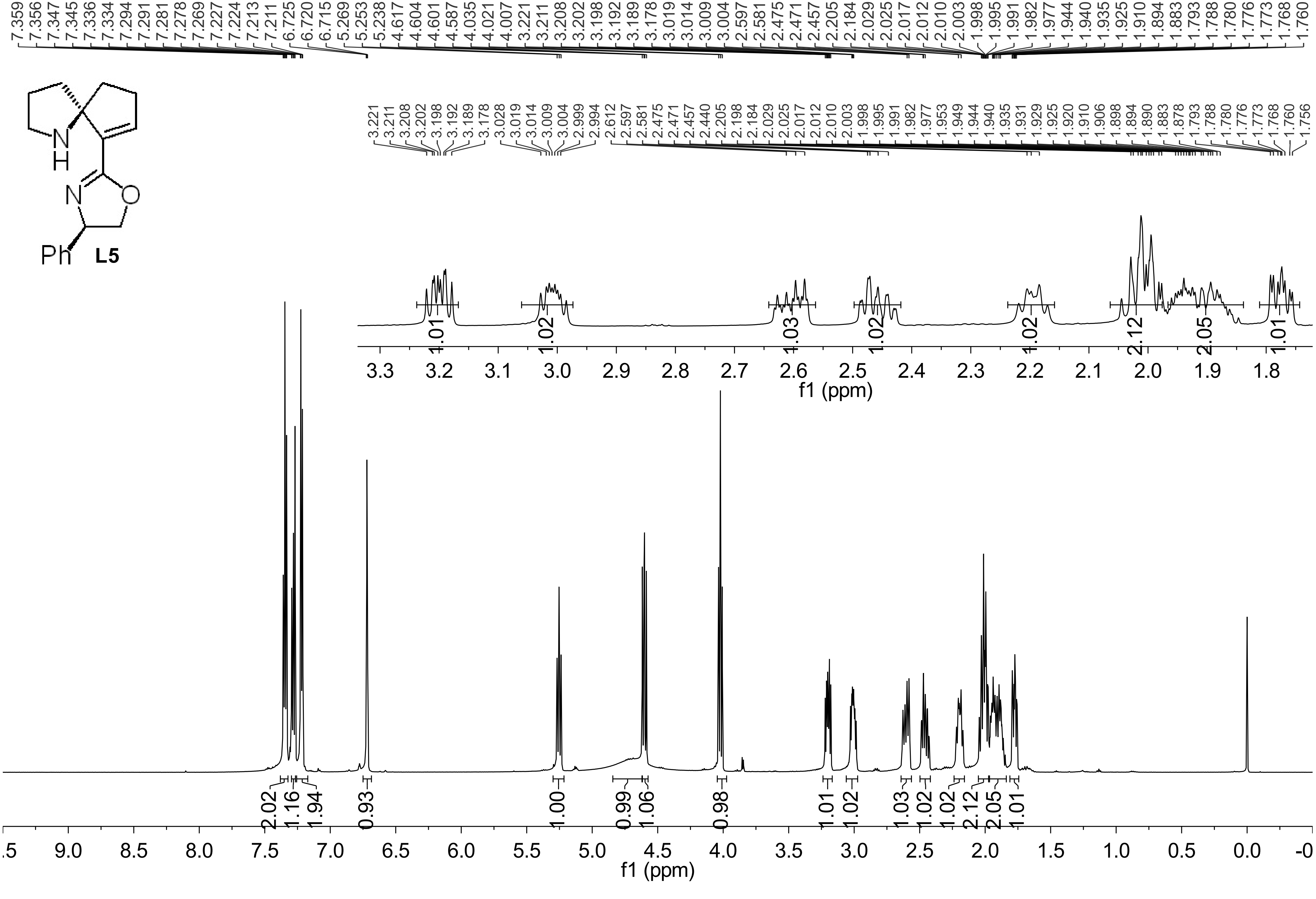
7.359 7.356 7.347 7.345 7.336 7.334 7.294 7.291 7.281 7.278 7.269 7.227 7.224 7.213 7.211 6.725 6.720 6.715 5.269 5.253 5.238 4.617 4.604 4.601 4.587 4.035 4.021 4.007 3.221 3.211 3.208 3.202 3.198 3.192 3.189 3.178 3.019 3.014 3.009 3.004 2.597 2.581 2.475 2.471 2.457 2.205 2.184 2.029 2.025 2.017 2.012 2.010 2.003 1.998 1.995 1.991 1.982 1.977 1.944 1.940 1.935 1.925 1.910 1.894 1.883 1.793 1.788 1.780 1.776 1.773 1.768 1.760
3.221 3.211 3.208 3.202 3.198 3.192 3.189 3.178 3.028 3.019 3.014 3.009 3.004 2.999 2.994 2.612 2.597 2.581 2.475 2.471 2.457 2.440 2.205 2.198 2.184 2.029 2.025 2.017 2.012 2.010 2.003 1.998 1.995 1.991 1.982 1.977 1.953 1.949 1.944 1.940 1.935 1.931 1.929 1.925 1.920 1.910 1.906 1.898 1.894 1.890 1.883 1.878 1.793 1.788 1.780 1.776 1.773 1.768 1.760 1.756
N
H
N
O
Ph
L5
1.01 1.02 1.03 1.02 1.02 2.12 2.05 1.01
3.3 3.2 3.1 3.0 2.9 2.8 2.7 2.6 2.5 2.4 2.3 2.2 2.1 2.0 1.9 1.8
f1 (ppm)
2.02 1.16 1.94 0.93 1.00 0.99 1.06 0.98 1.01 1.02 1.03 1.02 1.02 2.12 2.05 1.01
.5 9.0 8.5 8.0 7.5 7.0 6.5 6.0 5.5 5.0 4.5 4.0 3.5 3.0 2.5 2.0 1.5 1.0 0.5 0.0 -0
f1 (ppm)

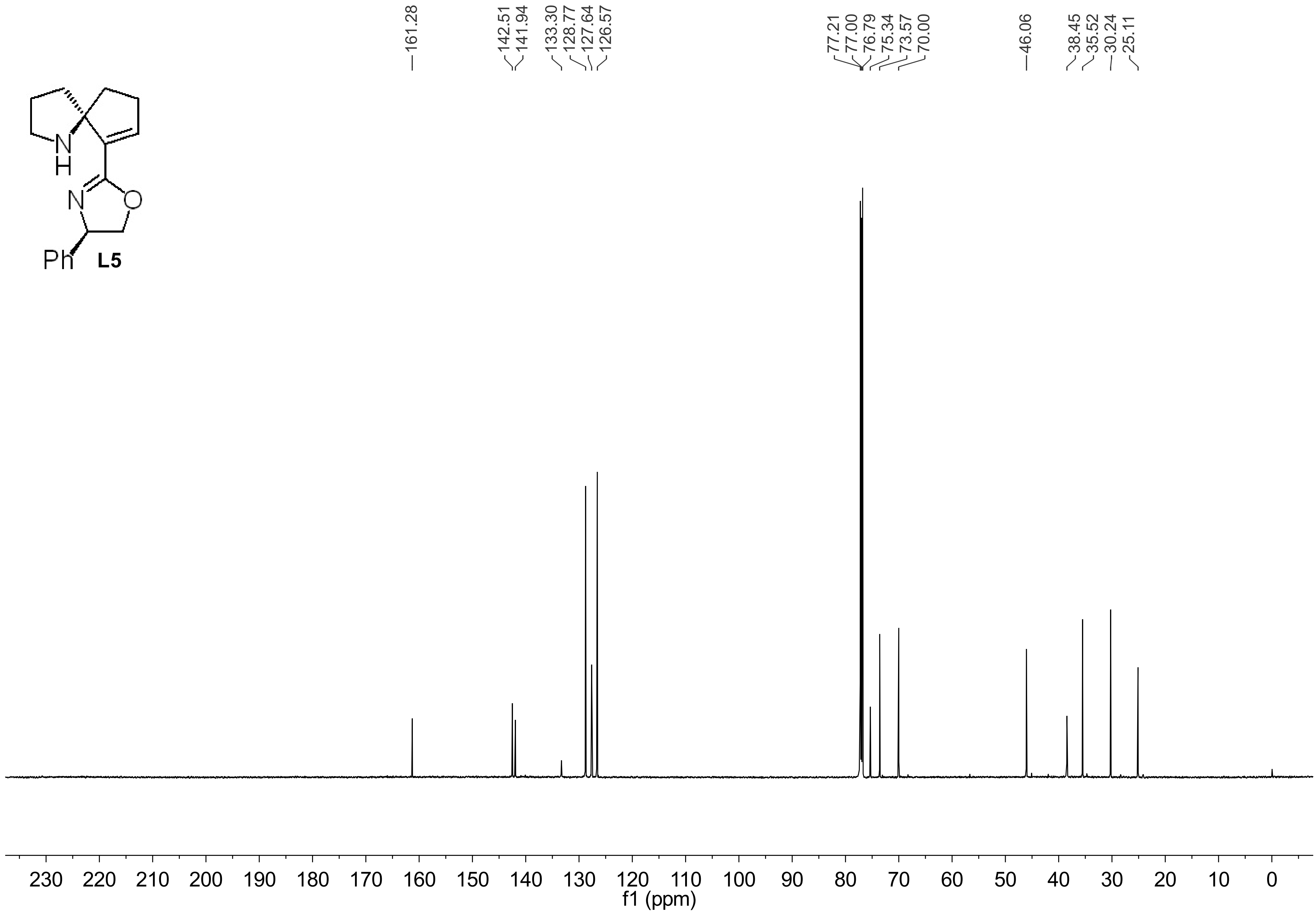

161.28
142.51
141.94
133.30
128.77
127.64
126.57
77.21
77.00
76.79
75.34
73.57
70.00
46.06
38.45
35.52
30.24
25.11
N
H
N
O
Ph
L5
230 220 210 200 190 180 170 160 150 140 130 120 110 100 90 80 70 60 50 40 30 20 10 0
f1 (ppm)

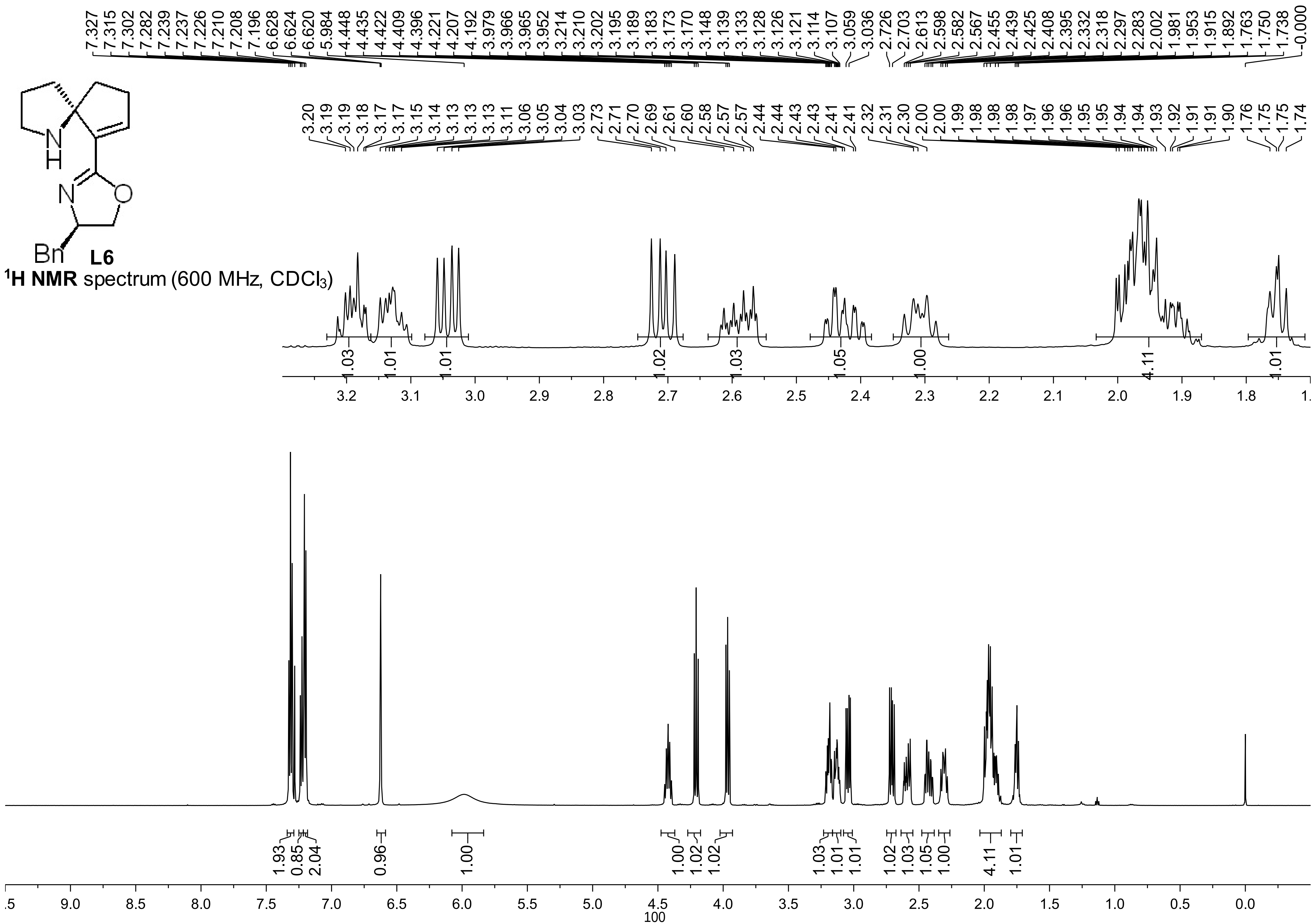

L6

^{1}H NMR spectrum (600 MHz, $CDCl_3$)

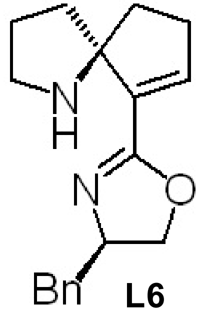

L6

[13]**C NMR** spectrum (150 MHz, $CDCl_3$)

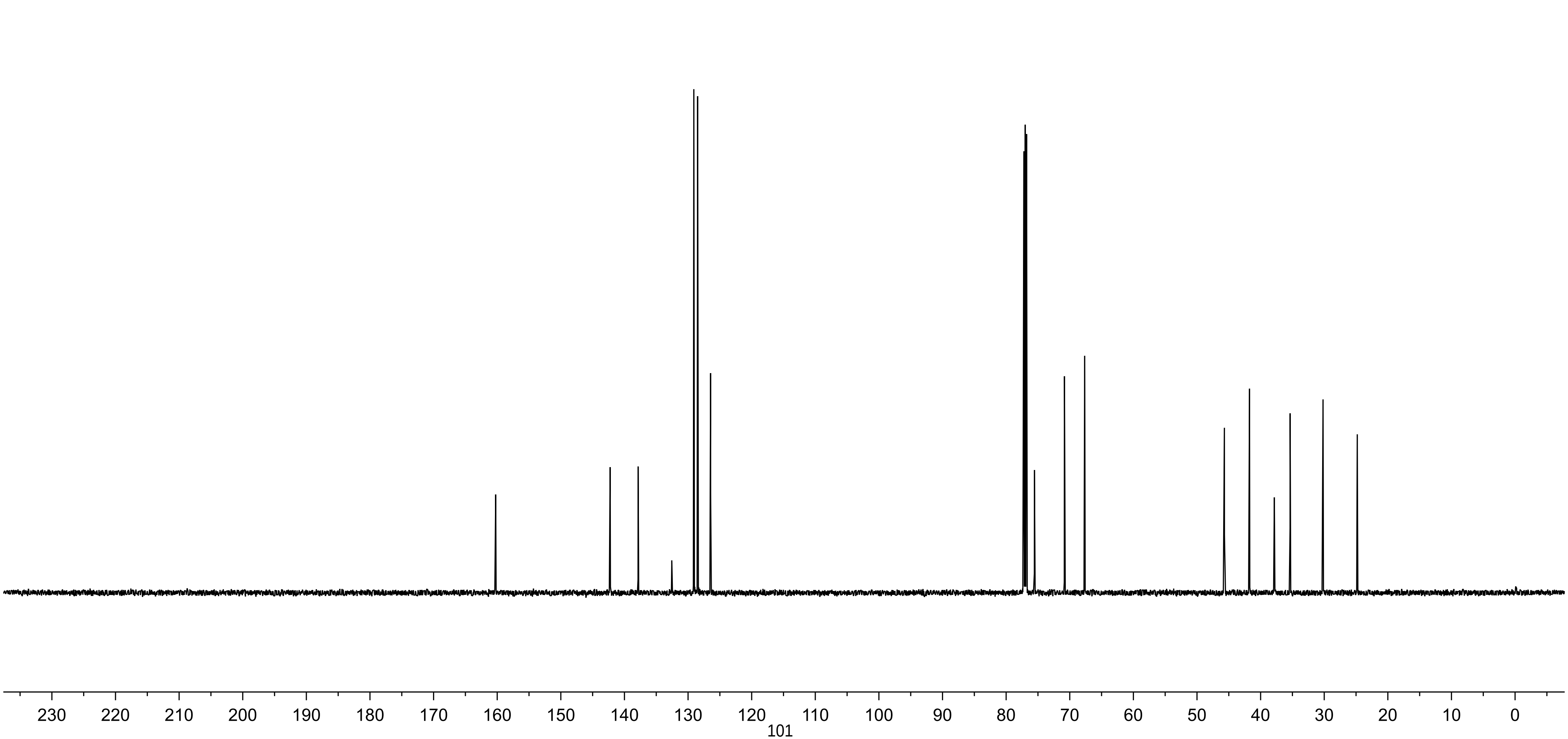

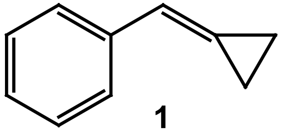

1

^{1}H NMR spectrum (400 MHz, $CDCl_3$)

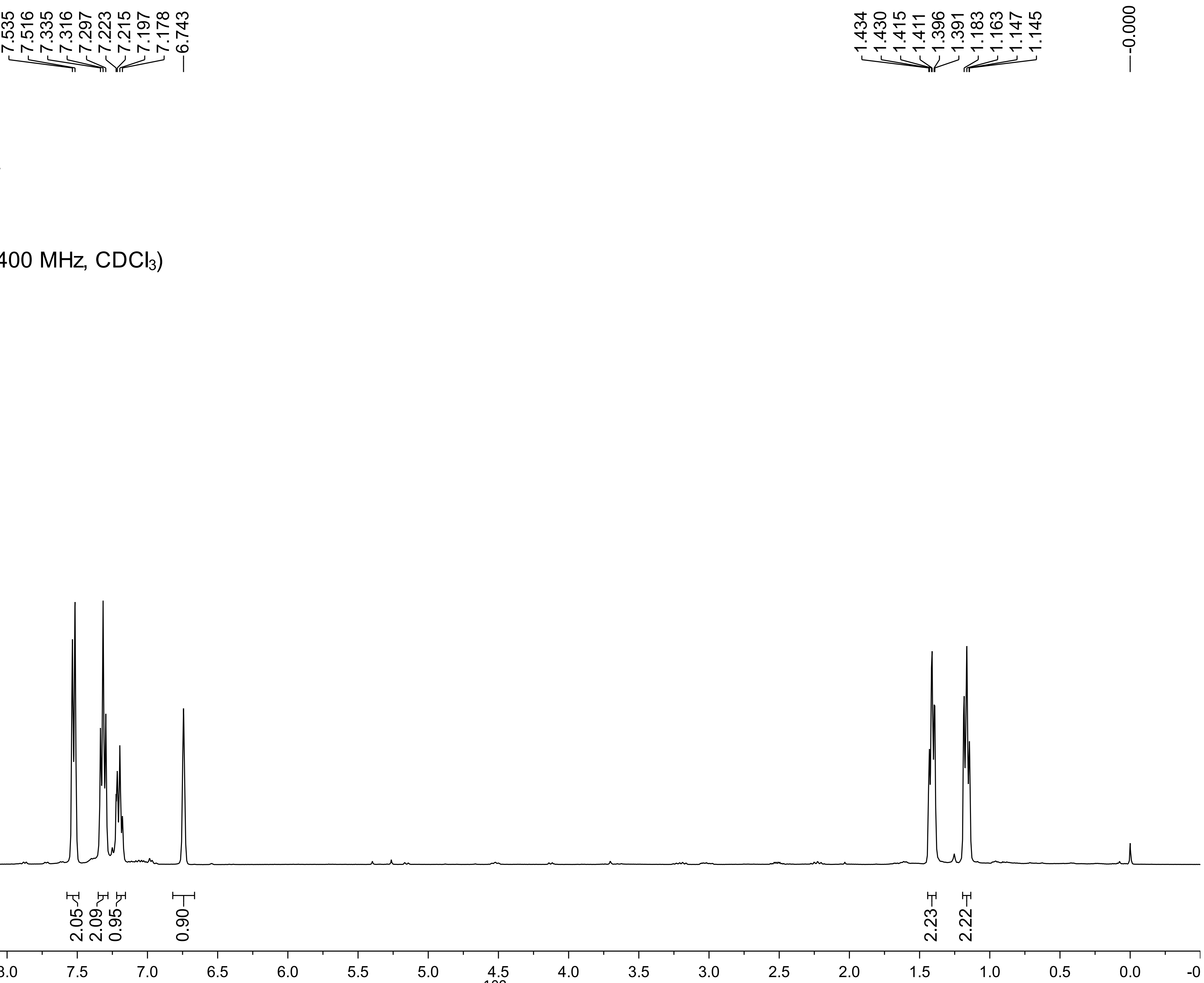

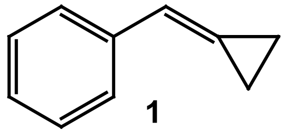

1

^{13}C NMR spectrum (100 MHz, $CDCl_3$)

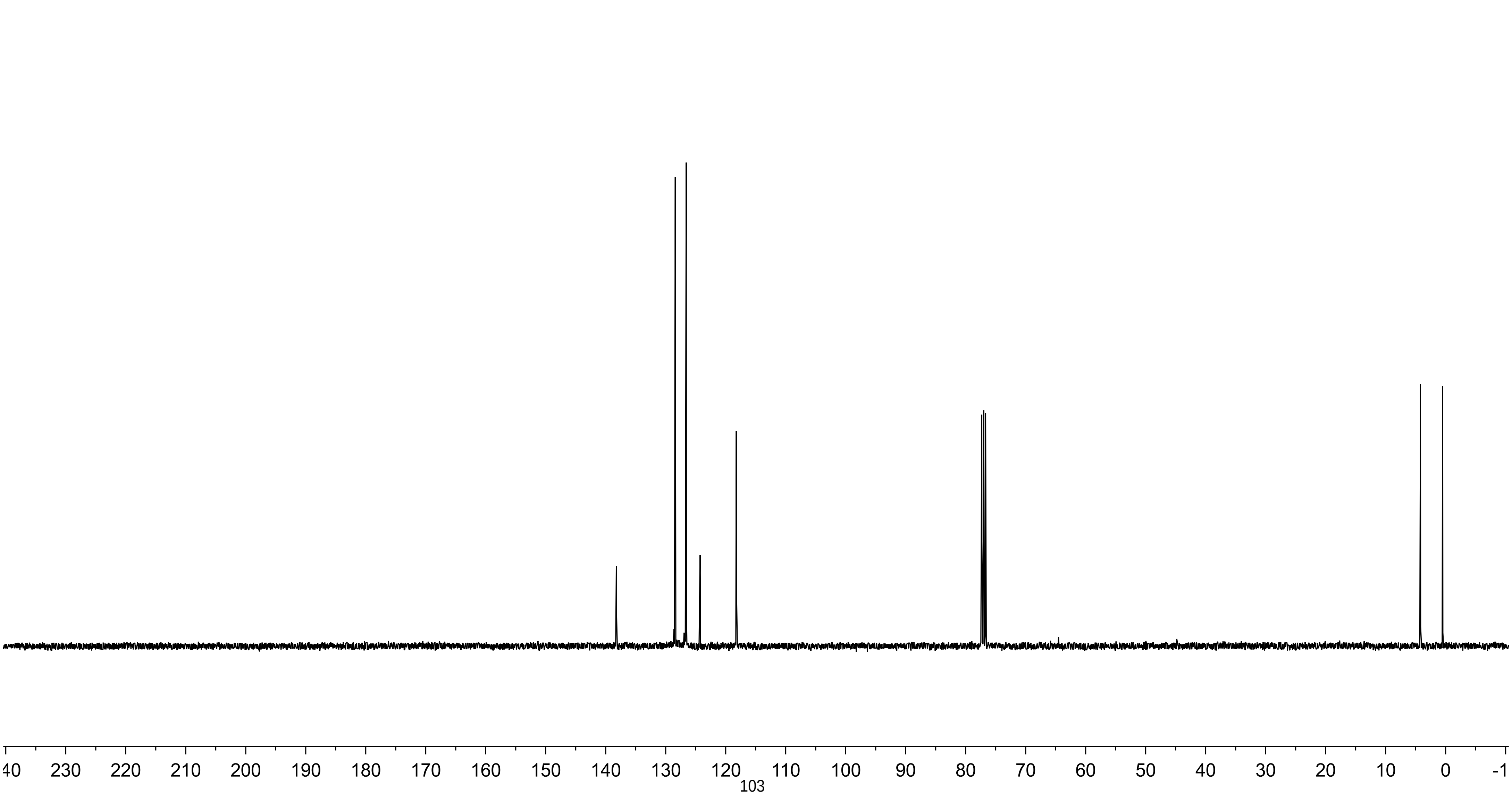

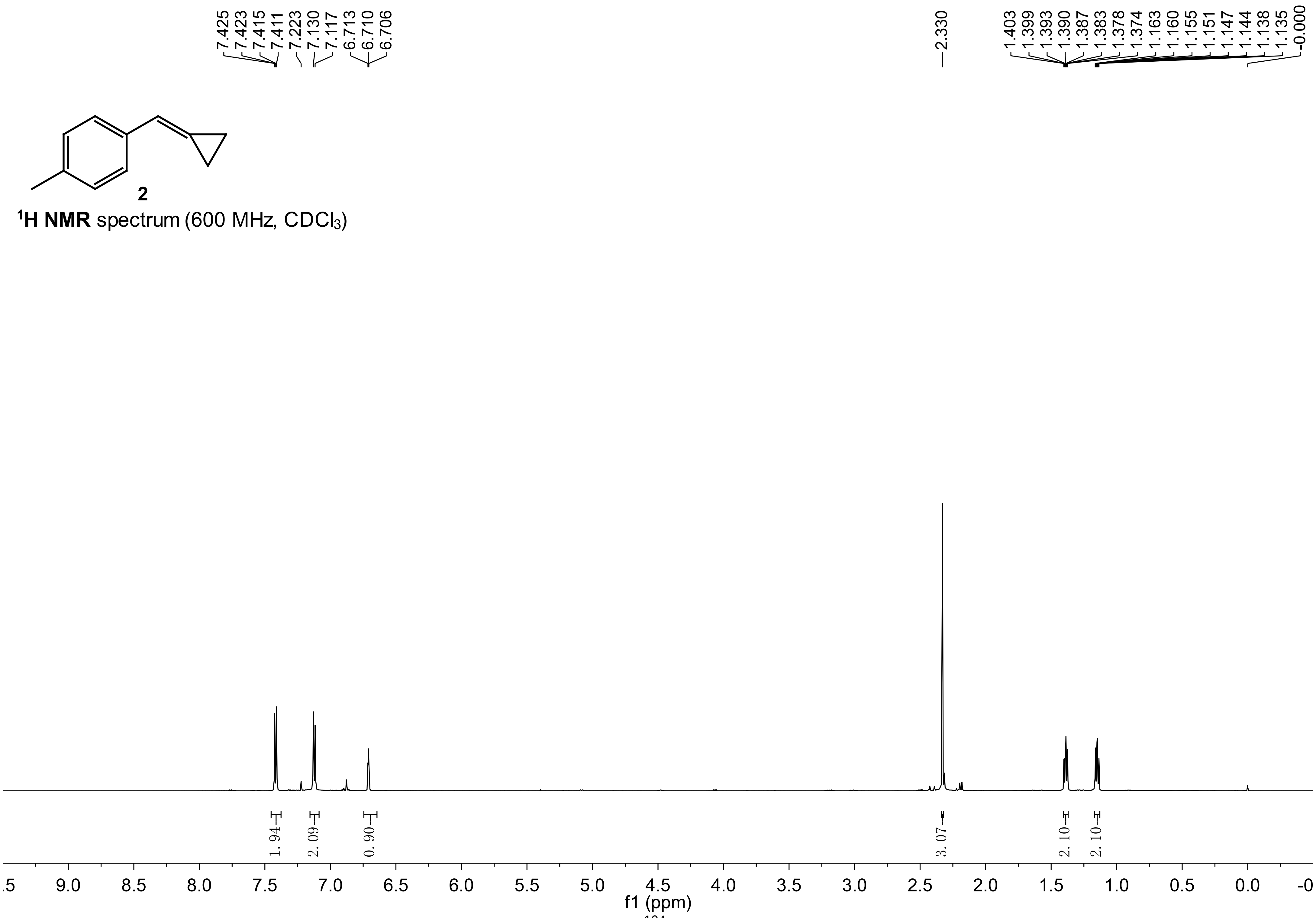

^{1}H NMR spectrum (600 MHz, $CDCl_3$)

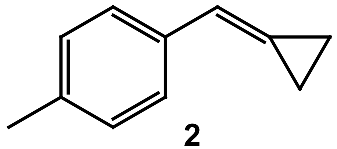

2

^{13}C NMR spectrum (150 MHz, $CDCl_3$)

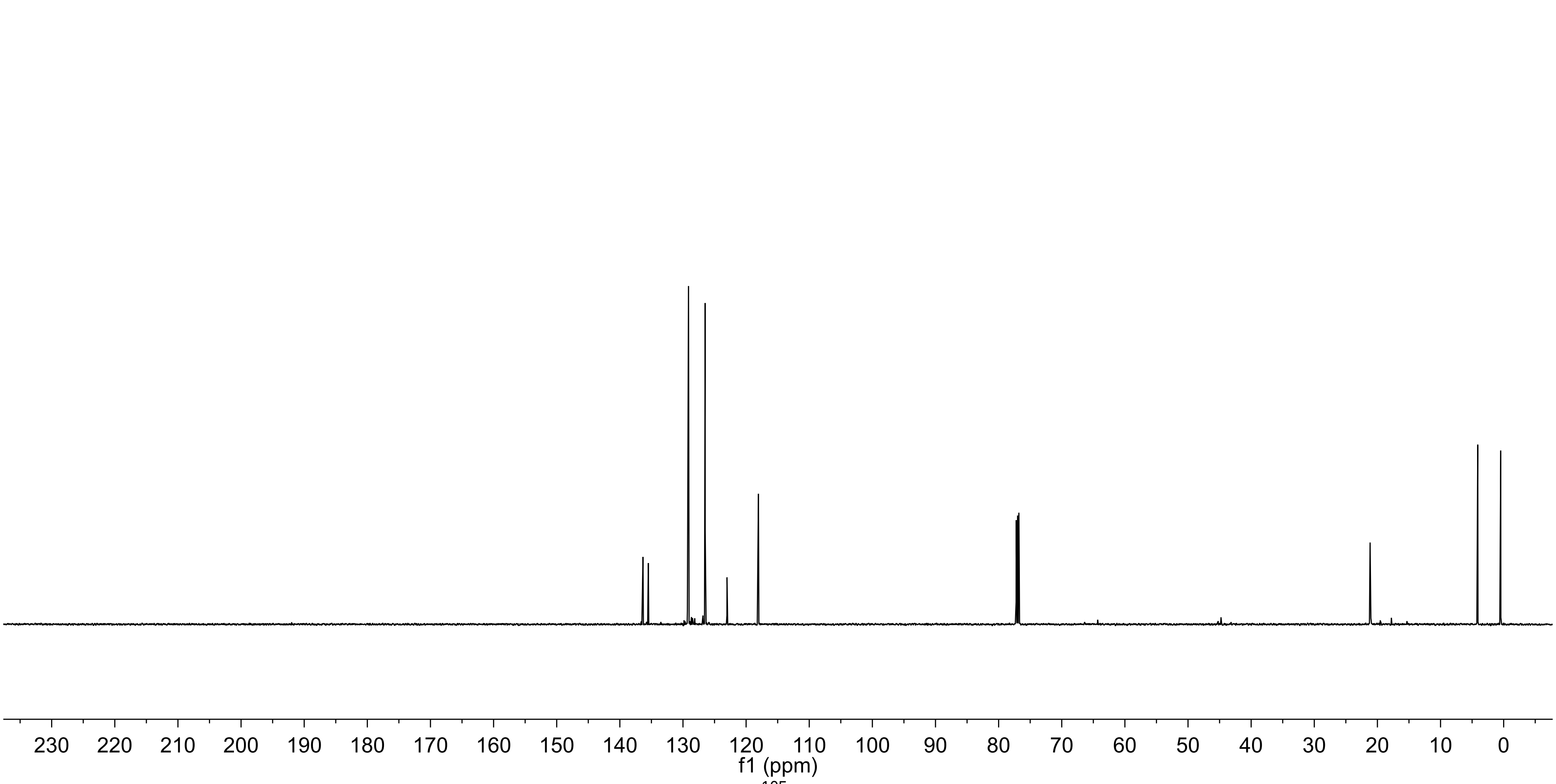

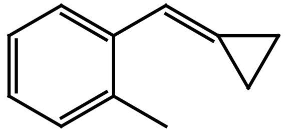

3

^{1}H NMR spectrum (400 MHz, $CDCl_3$)

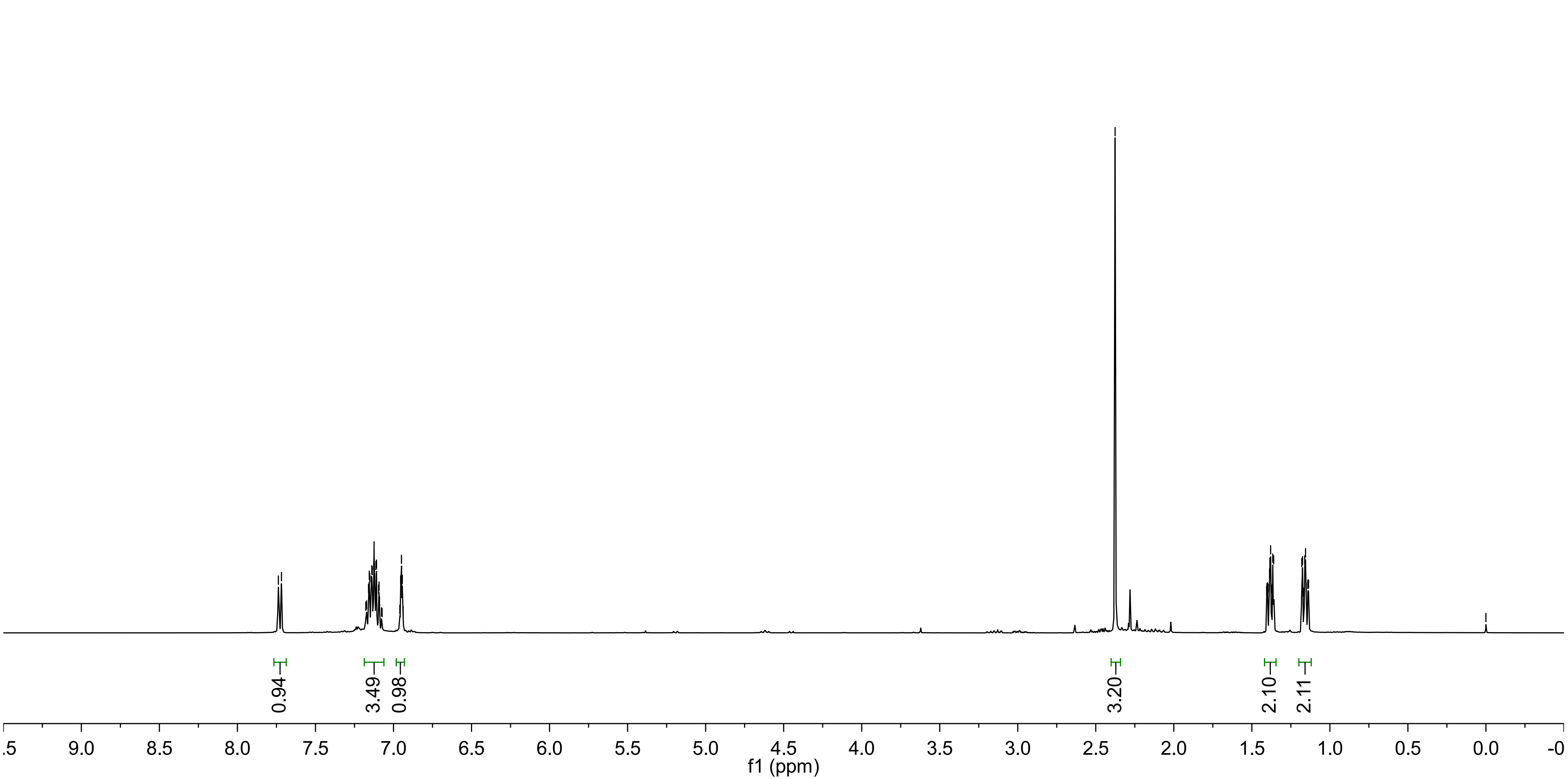

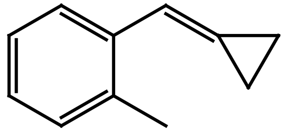

3

^{13}C NMR spectrum (100 MHz, $CDCl_3$)

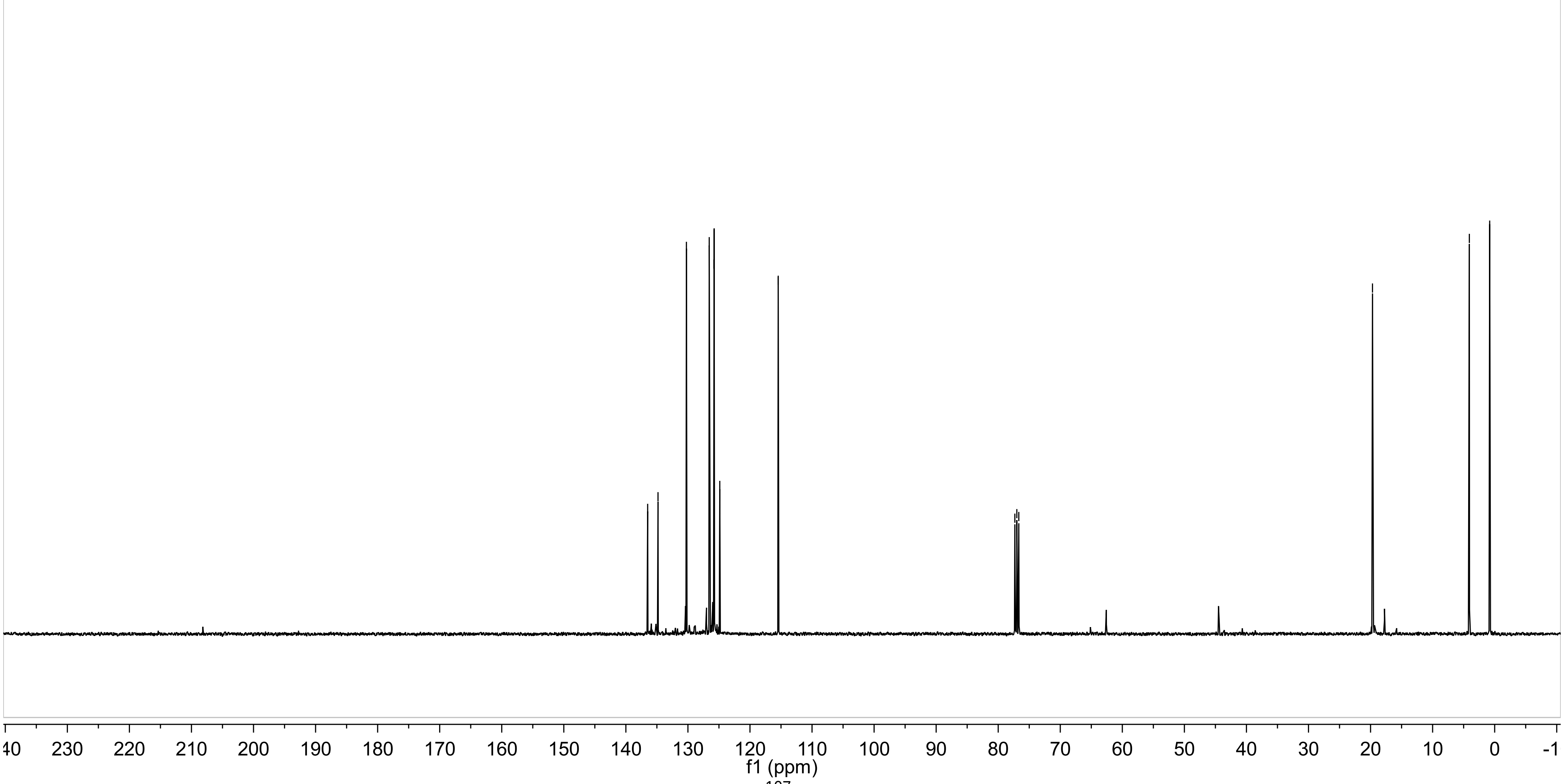

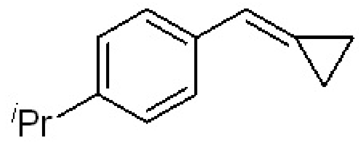

4

^{1}H NMR spectrum (600 MHz, $CDCl_3$)

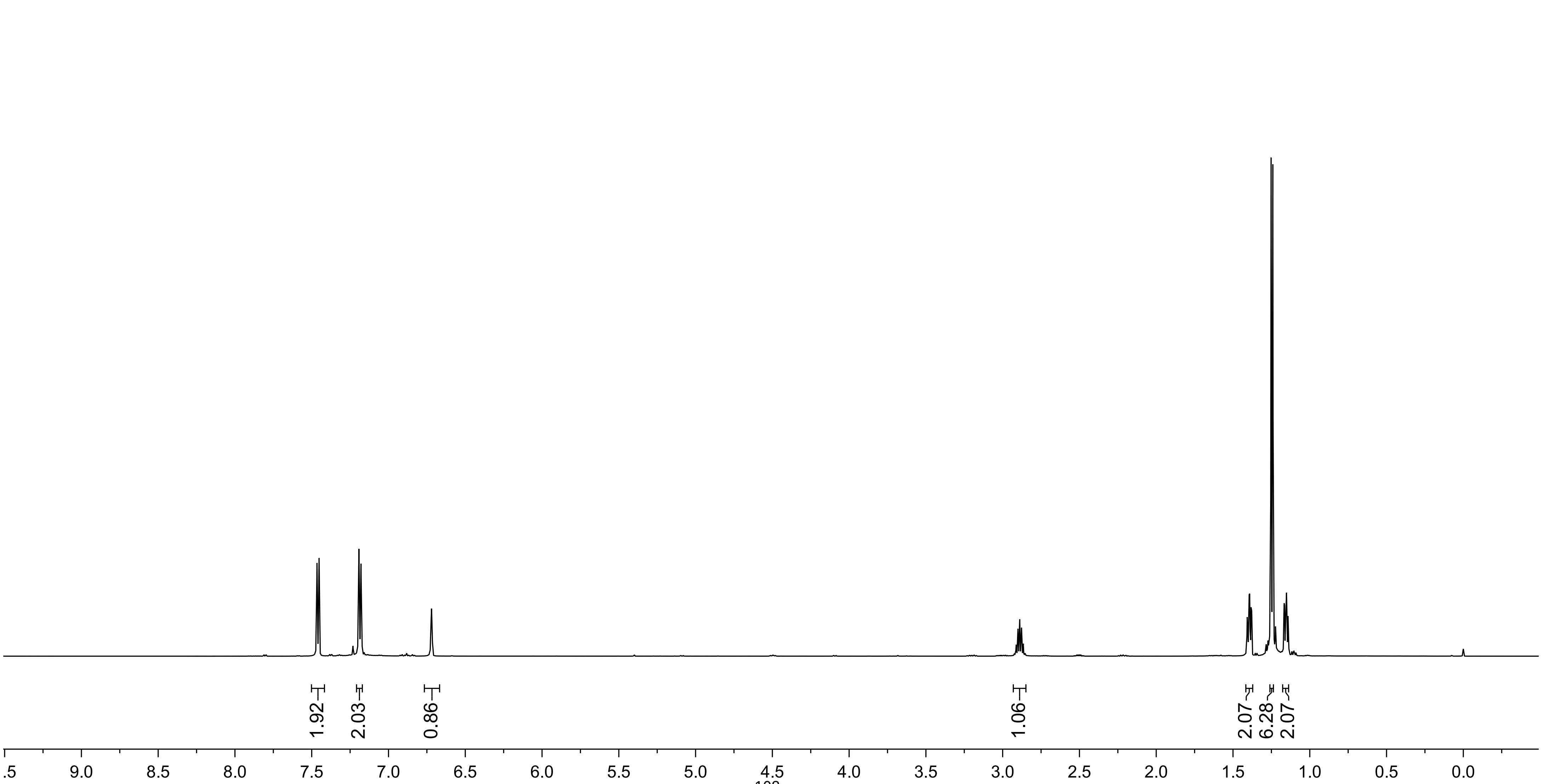

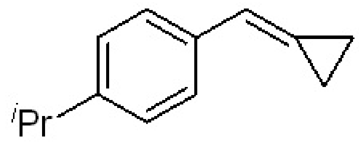

4

^{13}**C NMR** spectrum (150 MHz, $CDCl_3$)

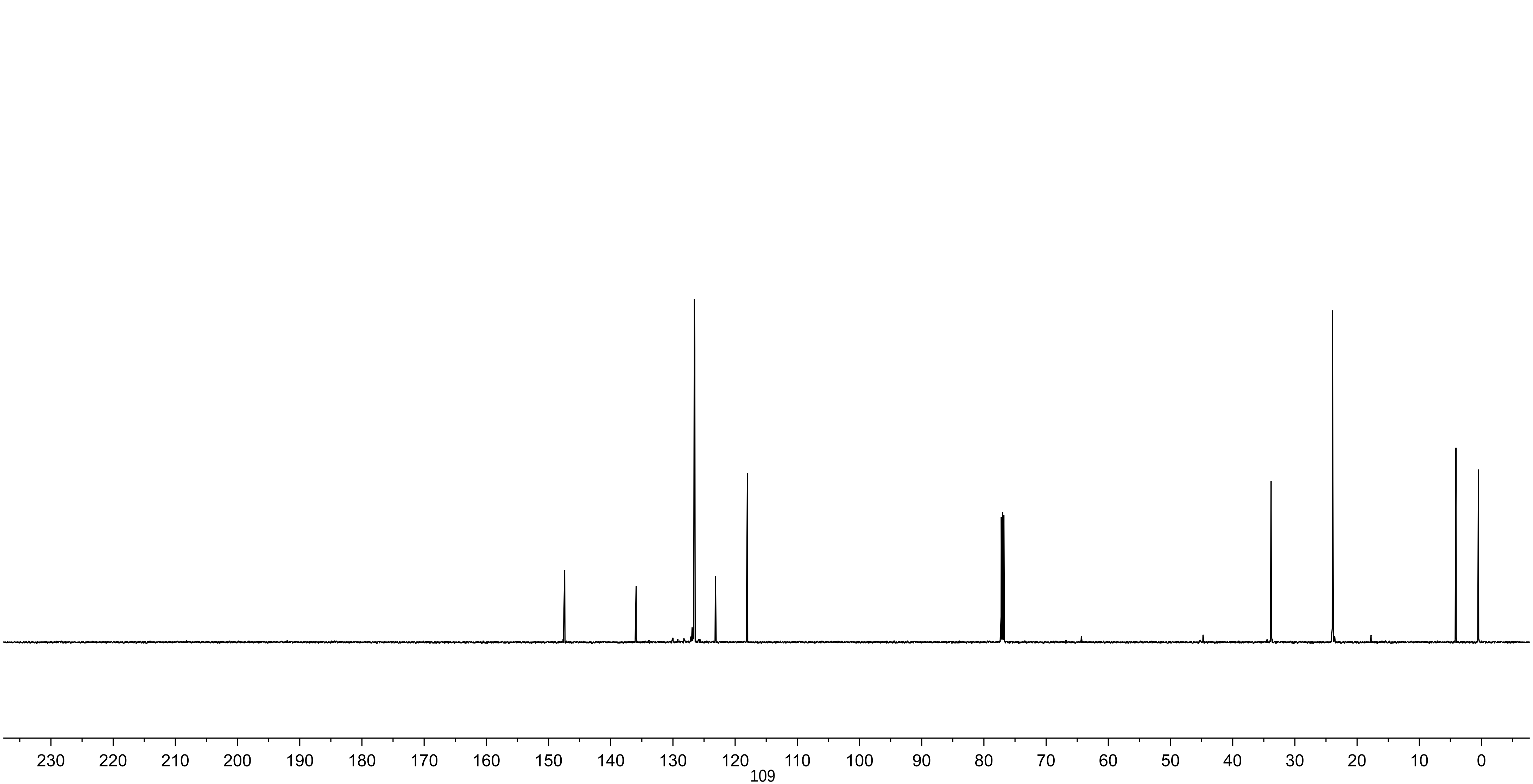

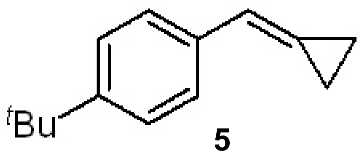

5

^{1}H NMR spectrum (600 MHz, $CDCl_3$)

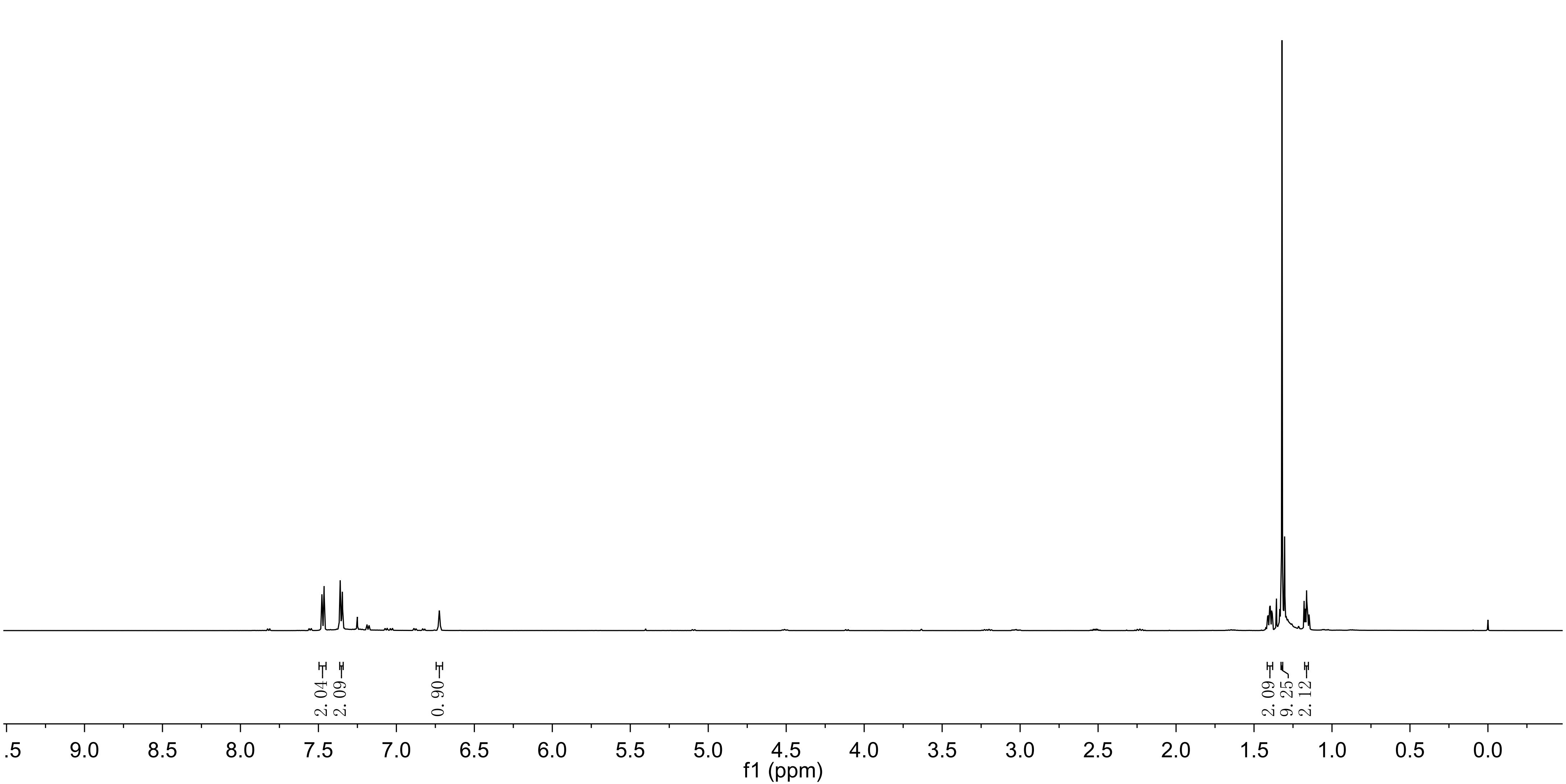

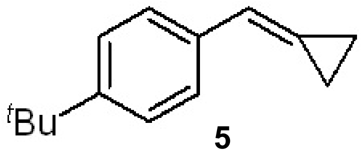

5

^{13}C NMR spectrum (150 MHz, $CDCl_3$)

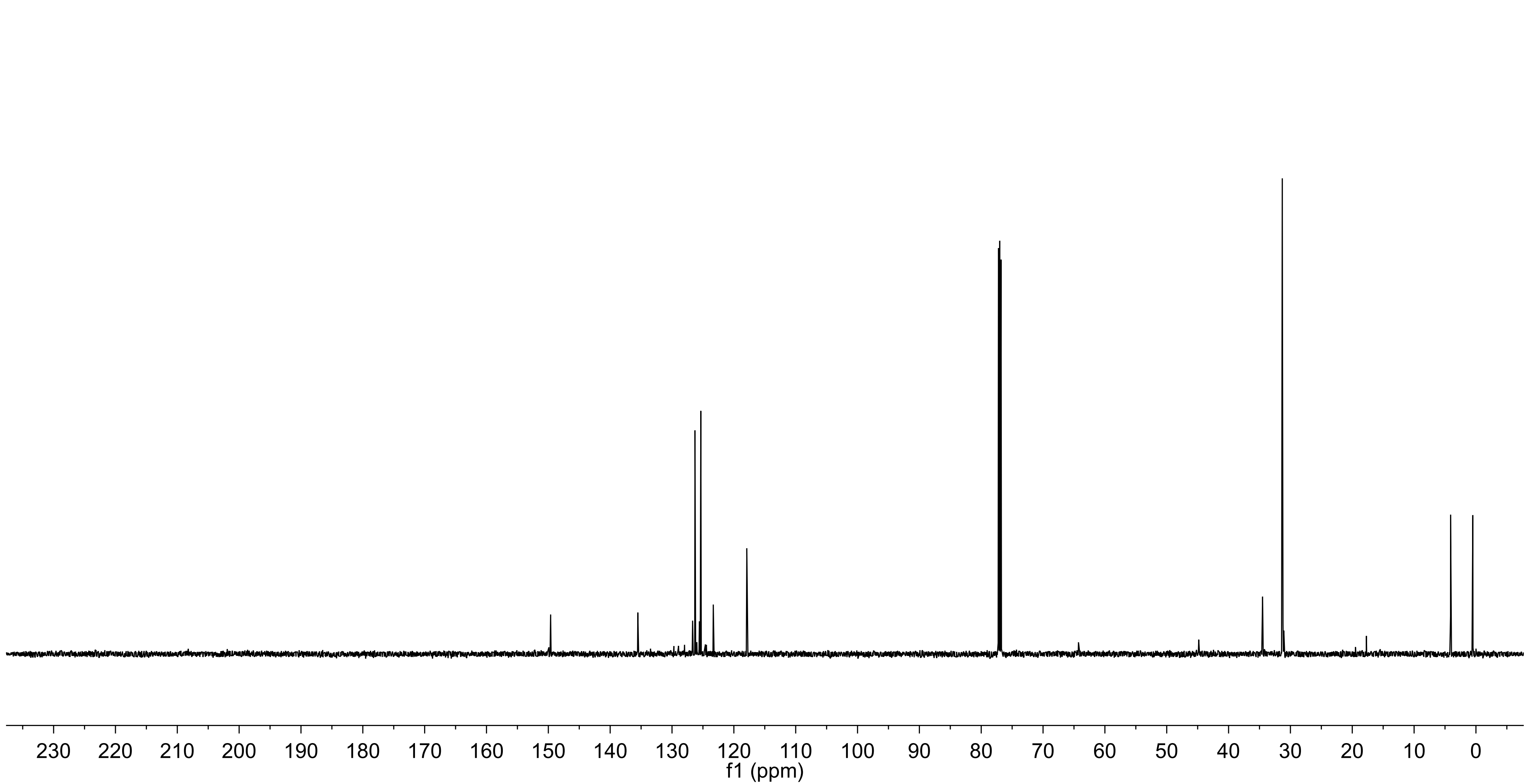

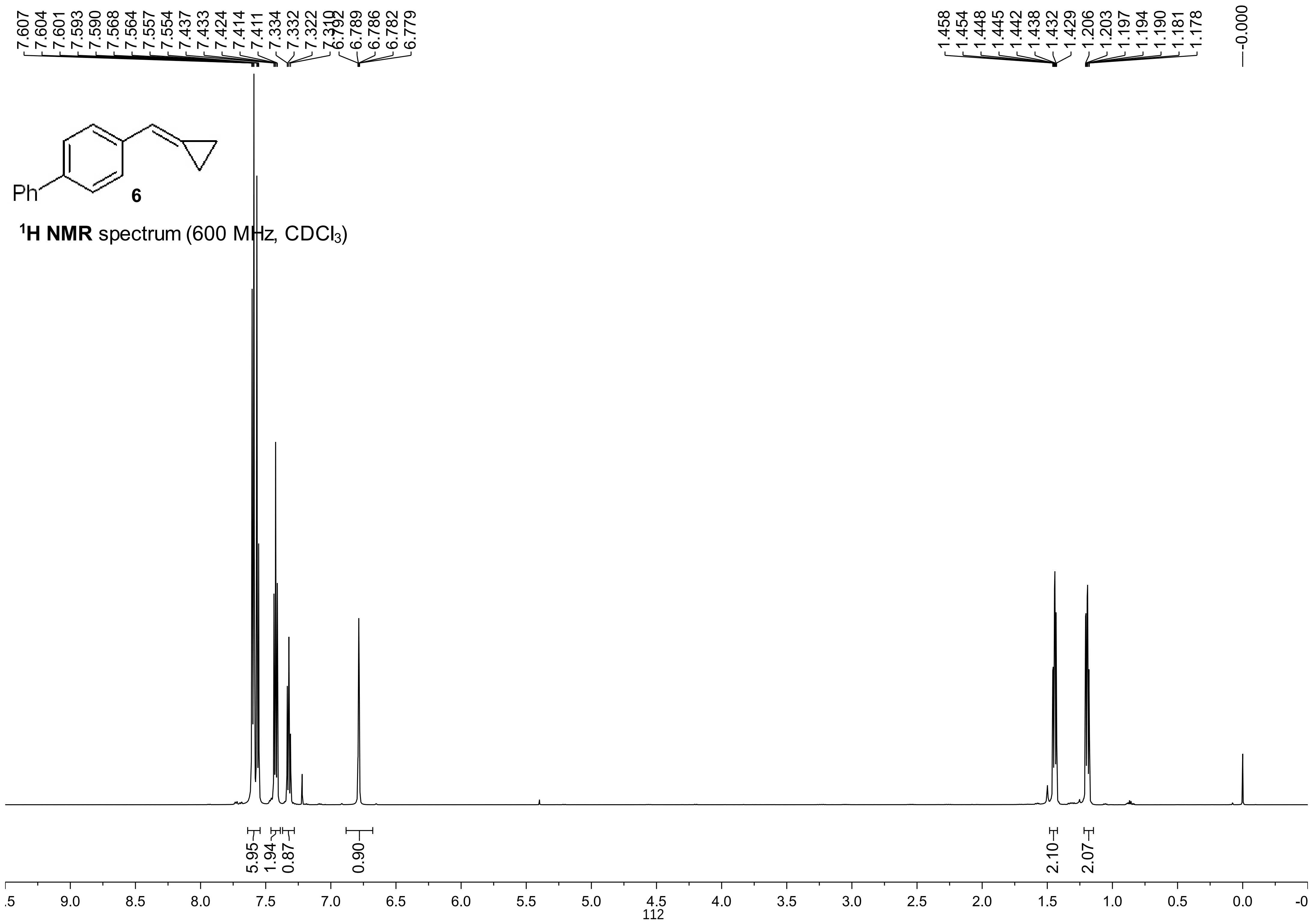
7.607
7.604
7.601
7.593
7.590
7.568
7.564
7.557
7.554
7.437
7.433
7.424
7.414
7.411
7.334
7.332
7.322
7.310
6.792
6.789
6.786
6.782
6.779
1.458
1.454
1.448
1.445
1.442
1.438
1.432
1.429
1.206
1.203
1.197
1.194
1.190
1.181
1.178
-0.000
Ph
6
1H NMR spectrum (600 MHz, CDCl3)
5.95
1.94
0.87
0.90
2.10
2.07
9.0
8.5
8.0
7.5
7.0
6.5
6.0
5.5
5.0
4.5
4.0
3.5
3.0
2.5
2.0
1.5
1.0
0.5
0.0

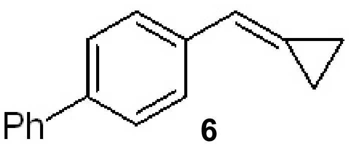

6

^{13}C NMR spectrum (150 MHz, $CDCl_3$)

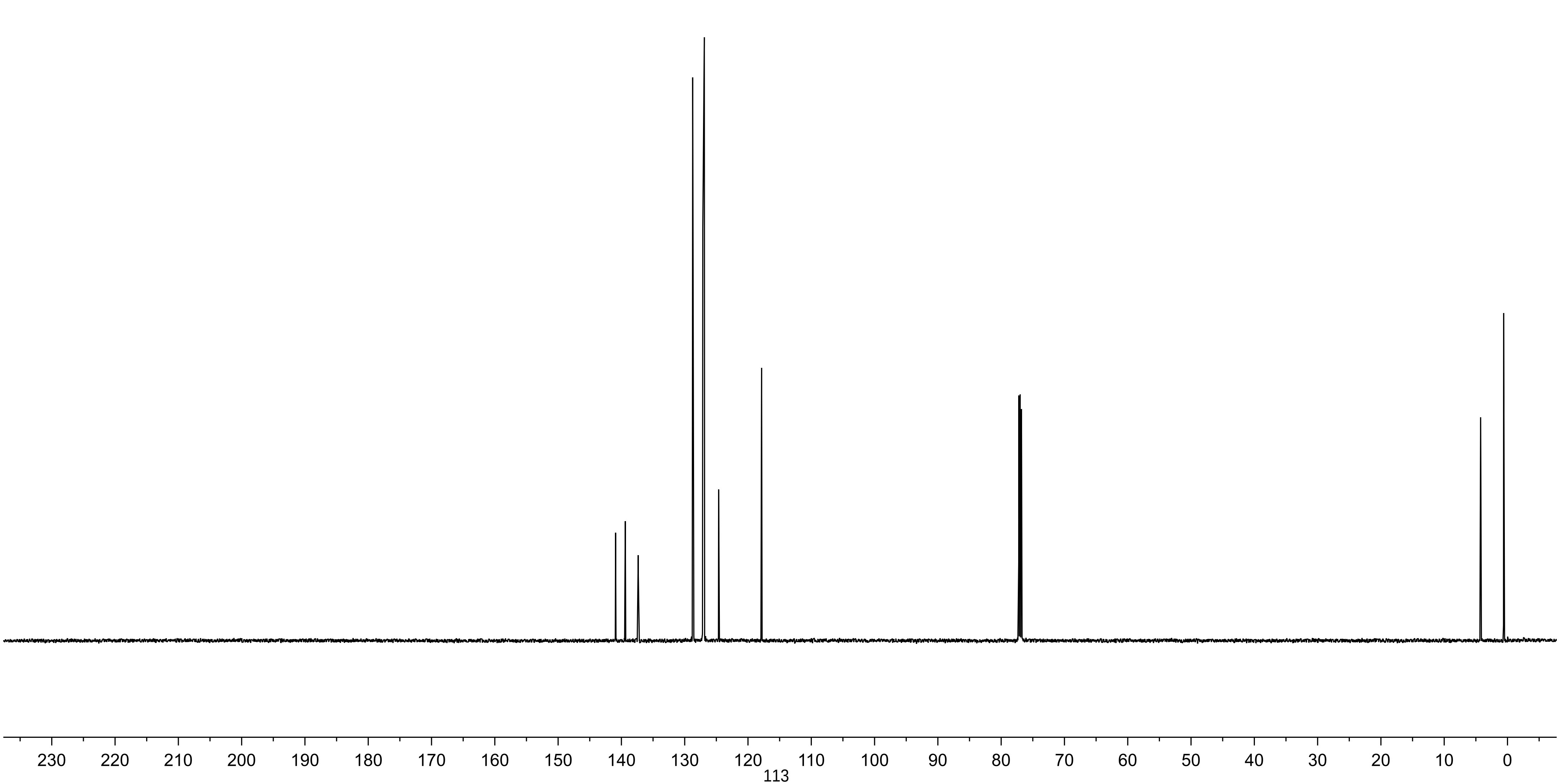

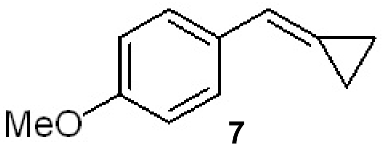

7

^{1}H NMR spectrum (400 MHz, $CDCl_3$)

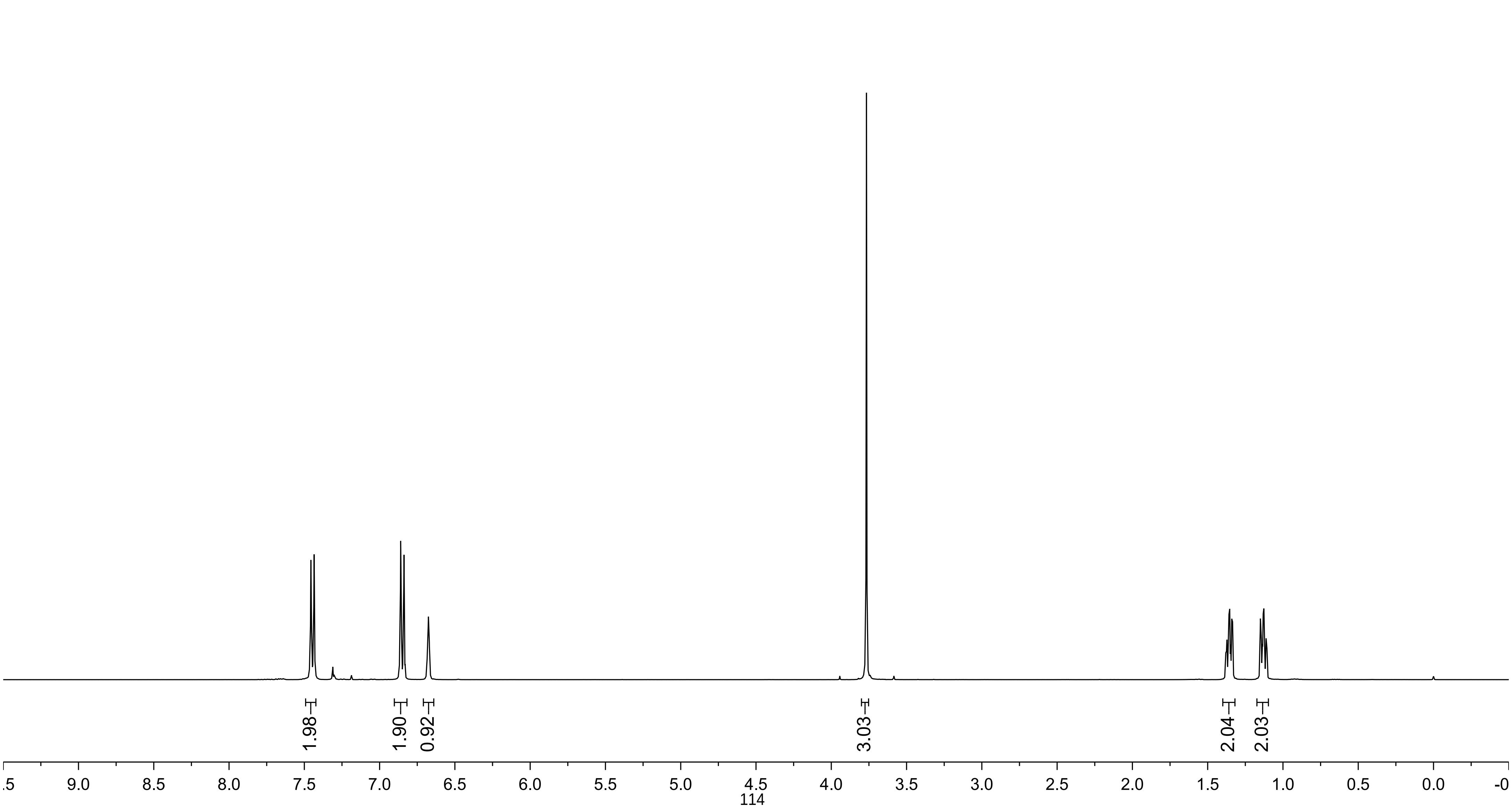

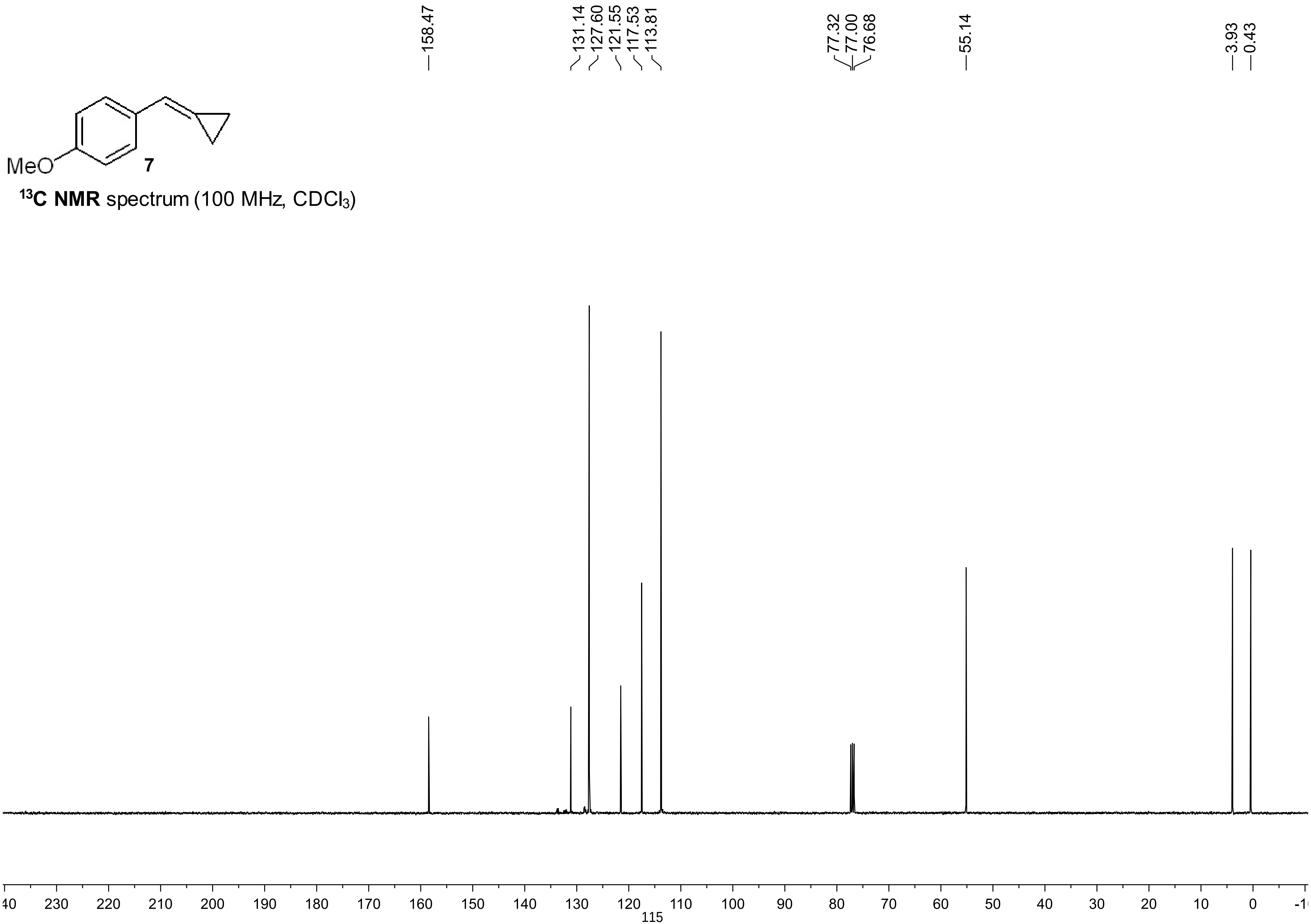

7

^{13}C NMR spectrum (100 MHz, $CDCl_3$)

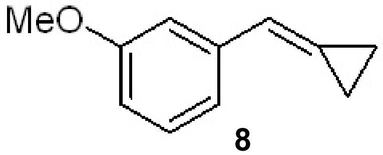

8

^{1}H NMR spectrum (400 MHz, $CDCl_3$)

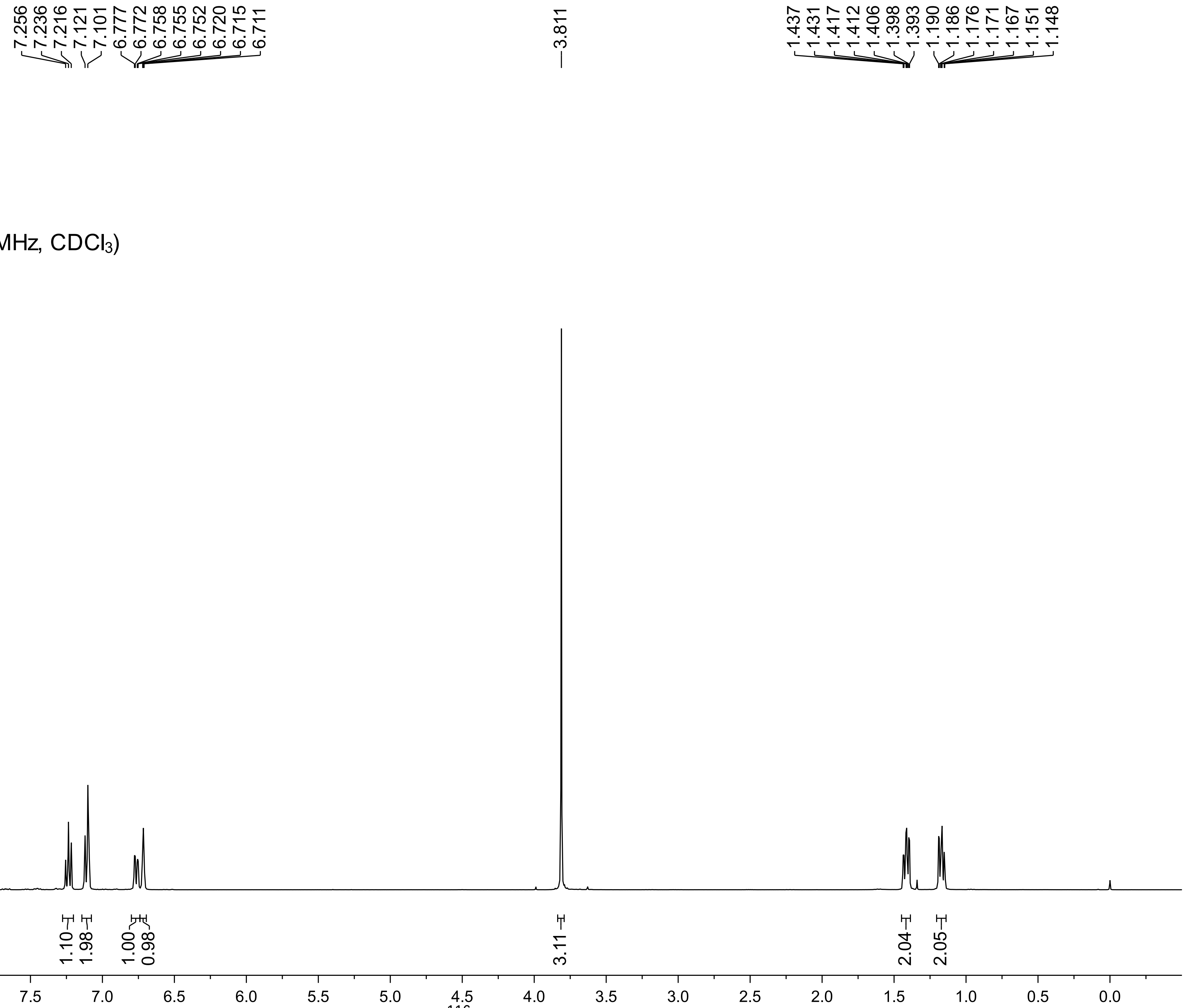

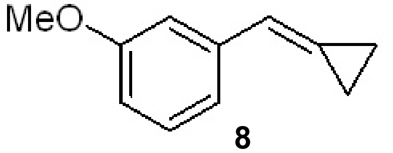

8

^{13}C NMR spectrum (100 MHz, $CDCl_3$)

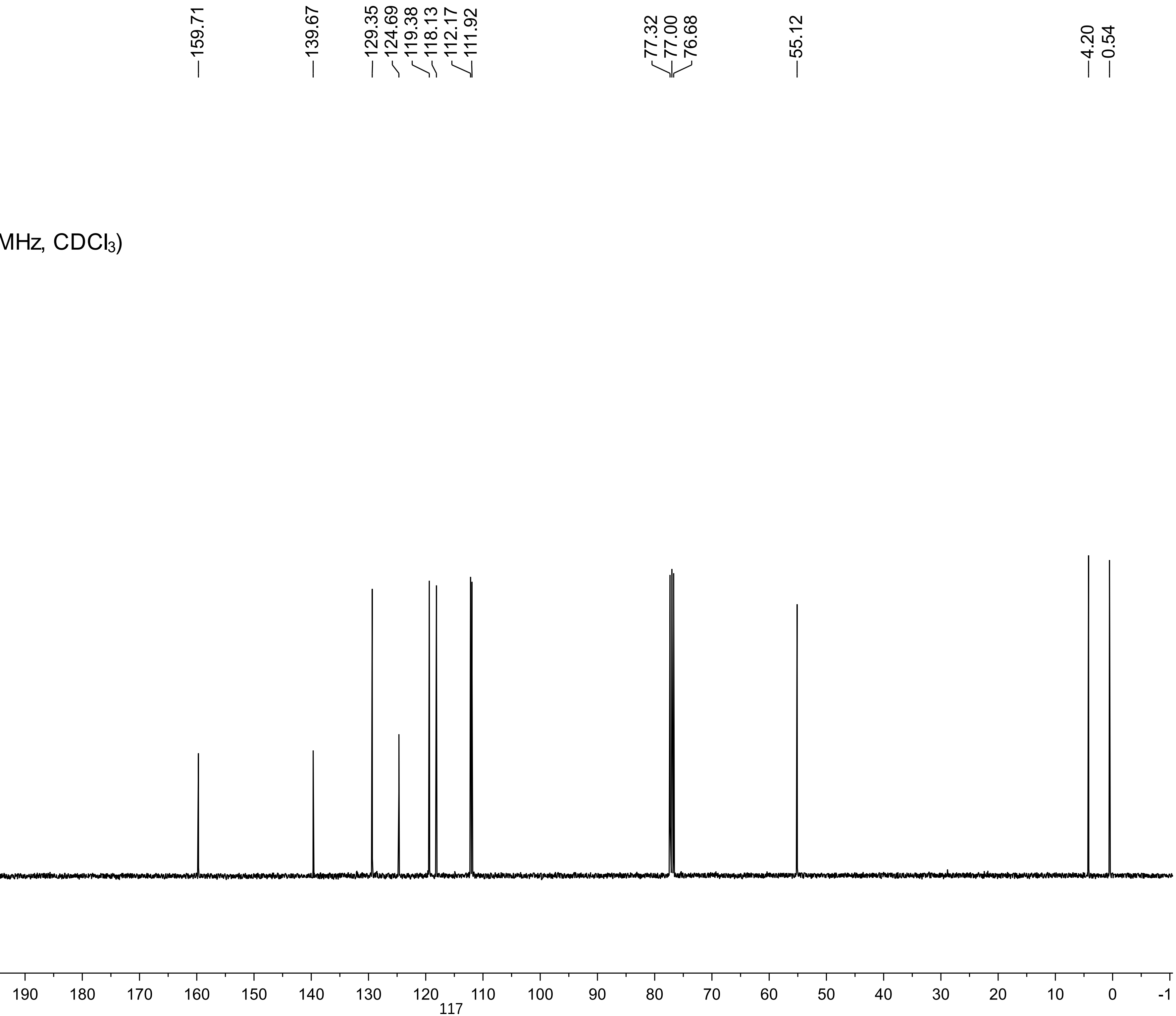

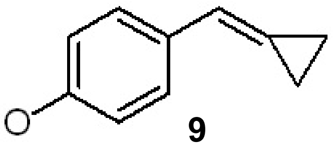

PhO

9

[1]**H NMR** spectrum (400 MHz, $CDCl_3$)

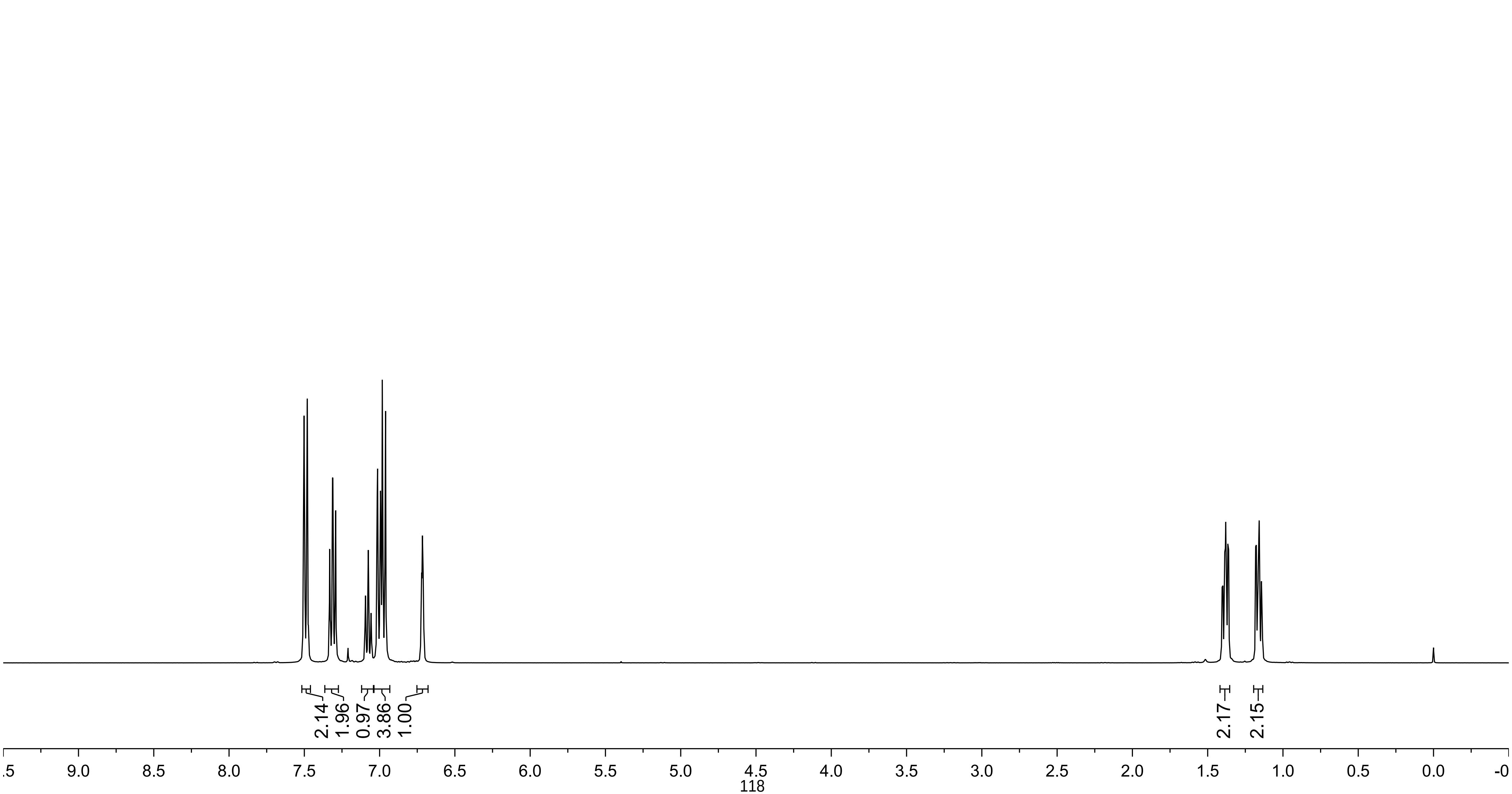

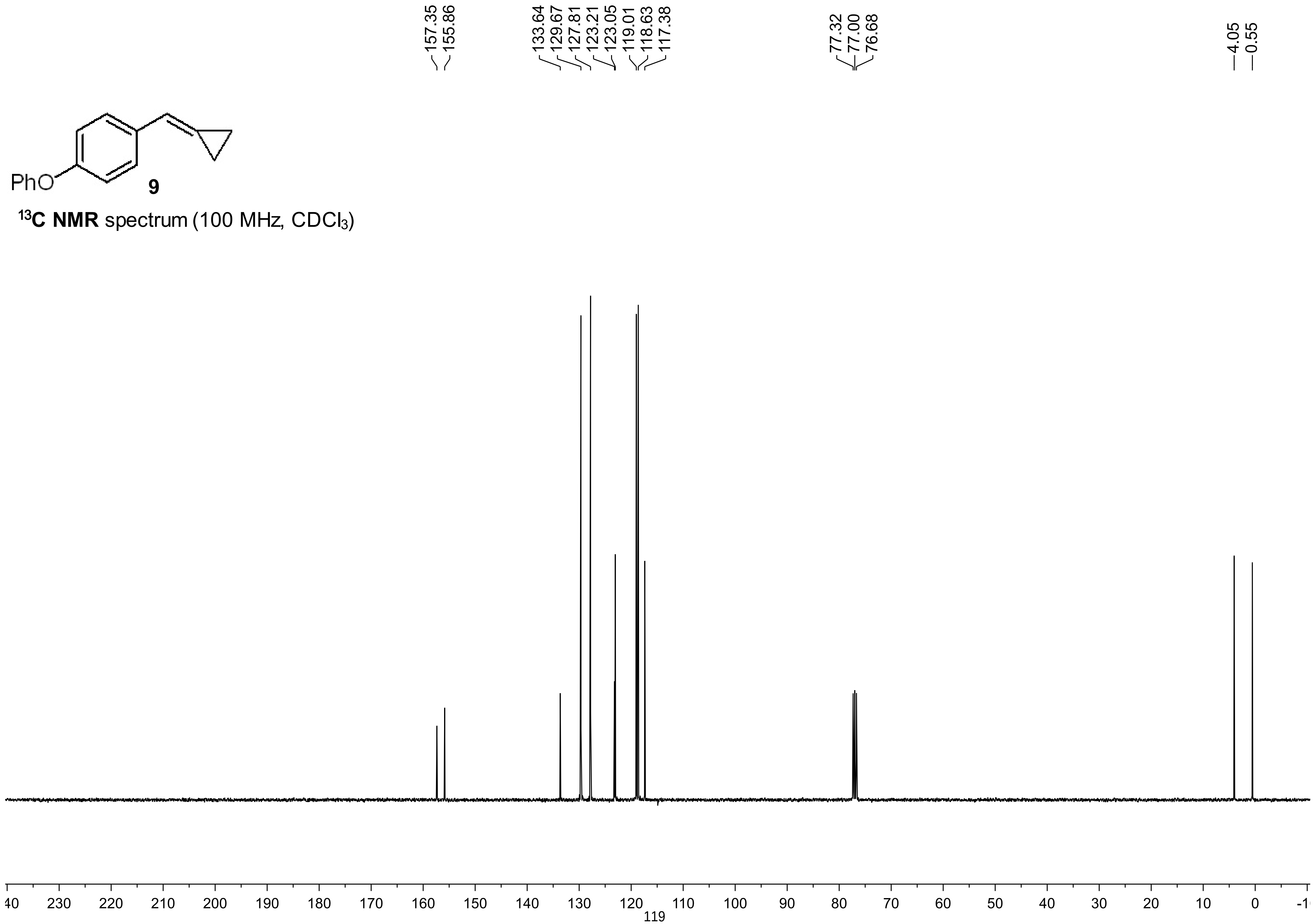

9

^{13}C NMR spectrum (100 MHz, $CDCl_3$)

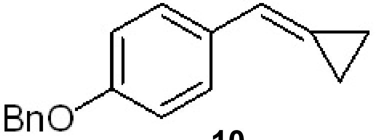

10

^{1}H NMR spectrum (600 MHz, $CDCl_3$)

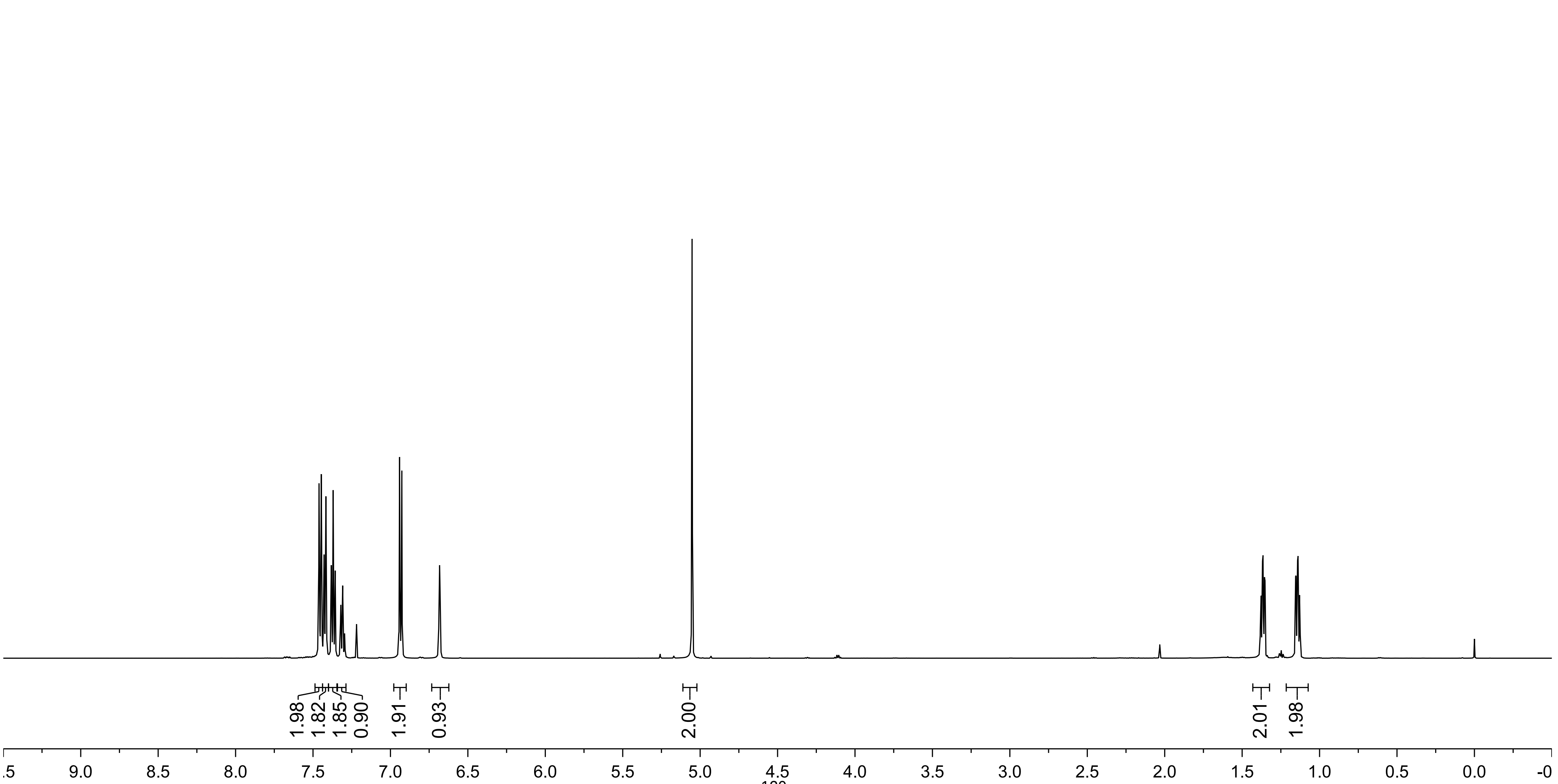

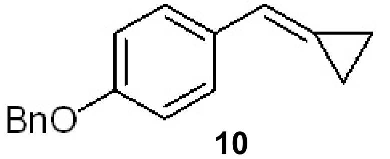

10

^{13}C NMR spectrum (150 MHz, $CDCl_3$)

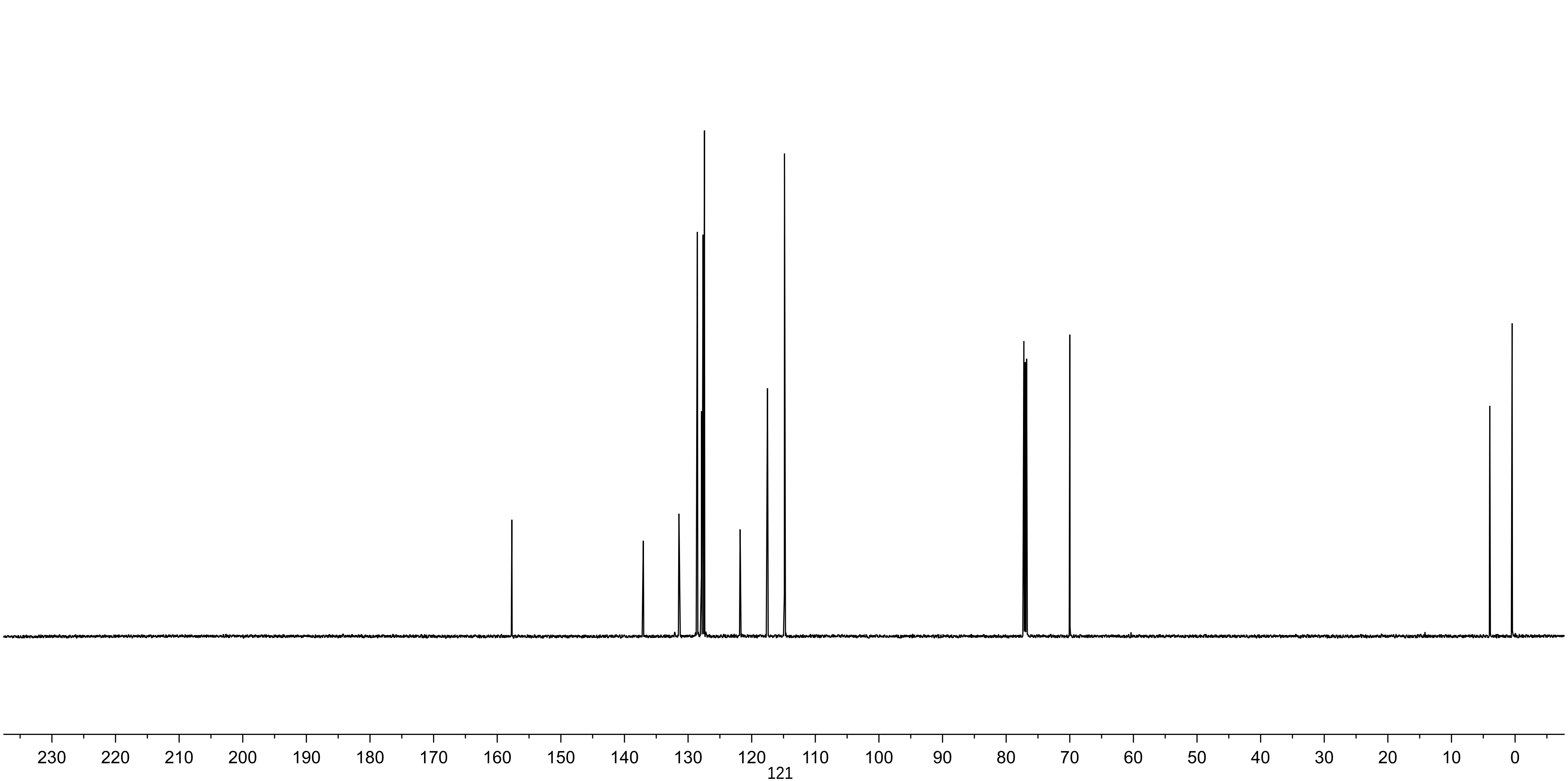

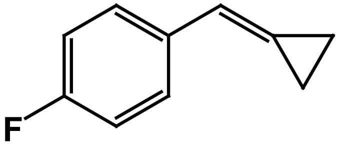

11

[1]**H NMR** spectrum (400 MHz, $CDCl_3$)

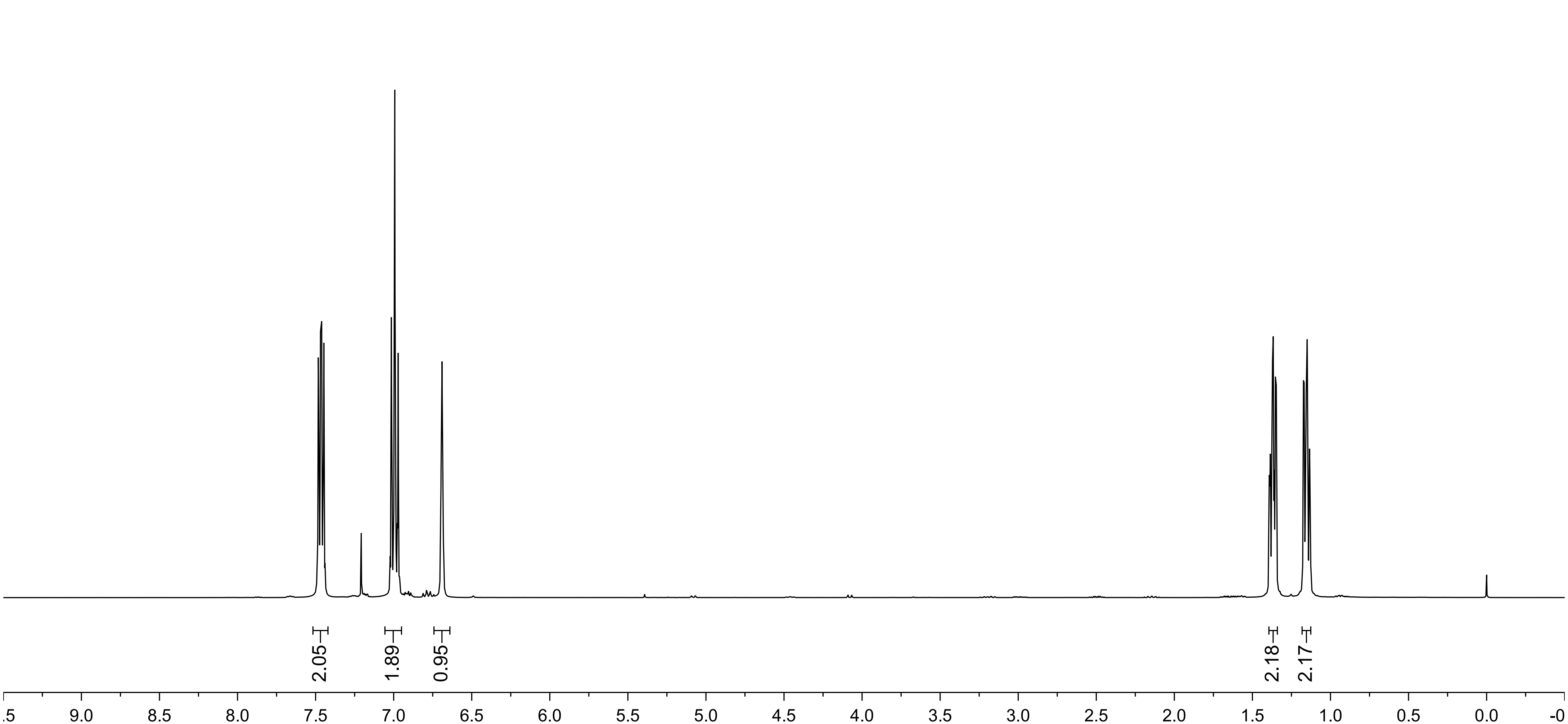

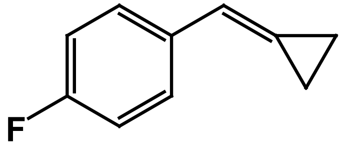

11

^{13}**C NMR** spectrum (100 MHz, $CDCl_3$)

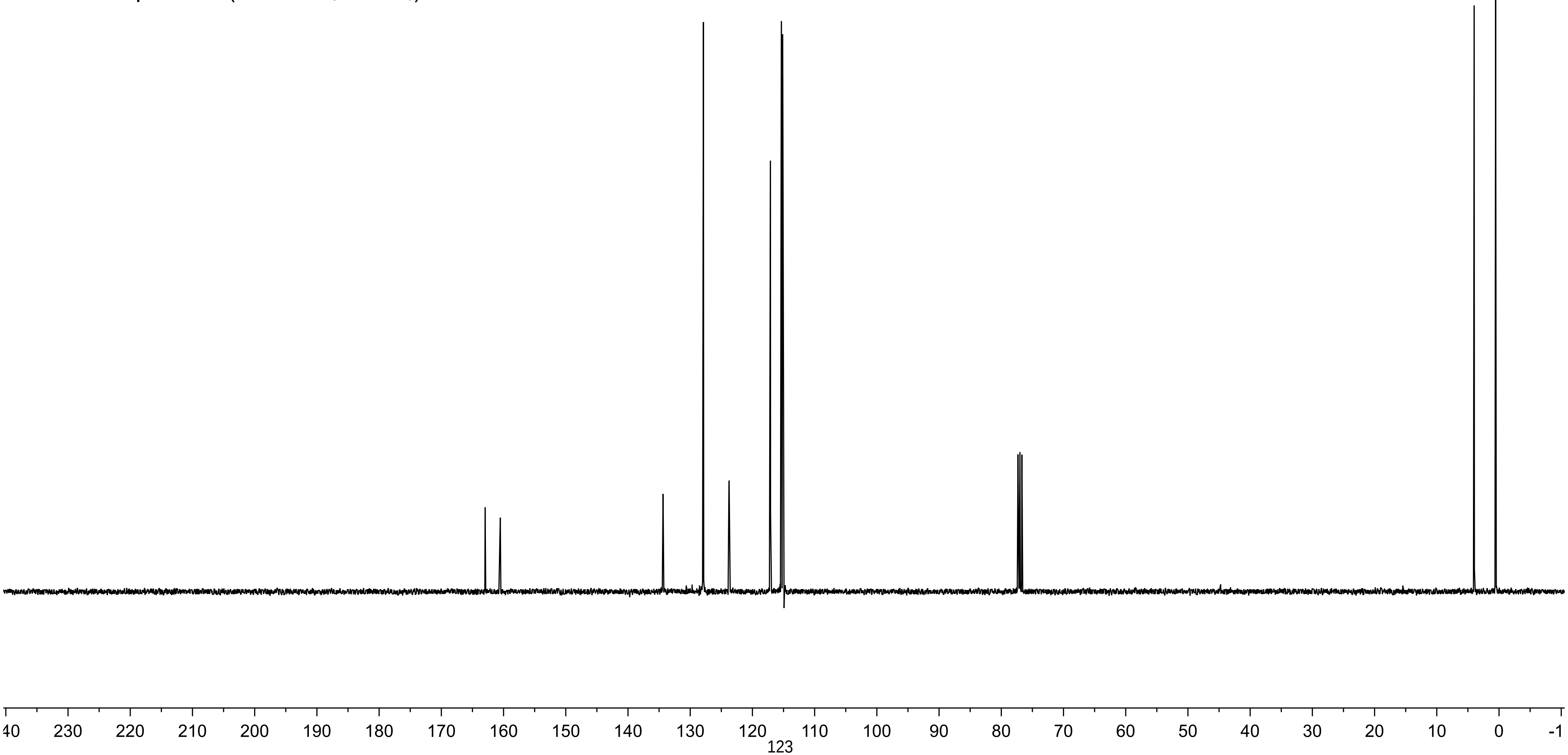

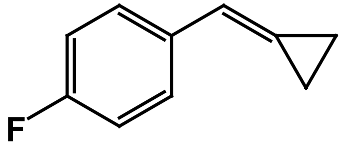

11

^{19}**F NMR** spectrum (376 MHz, $CDCl_3$)

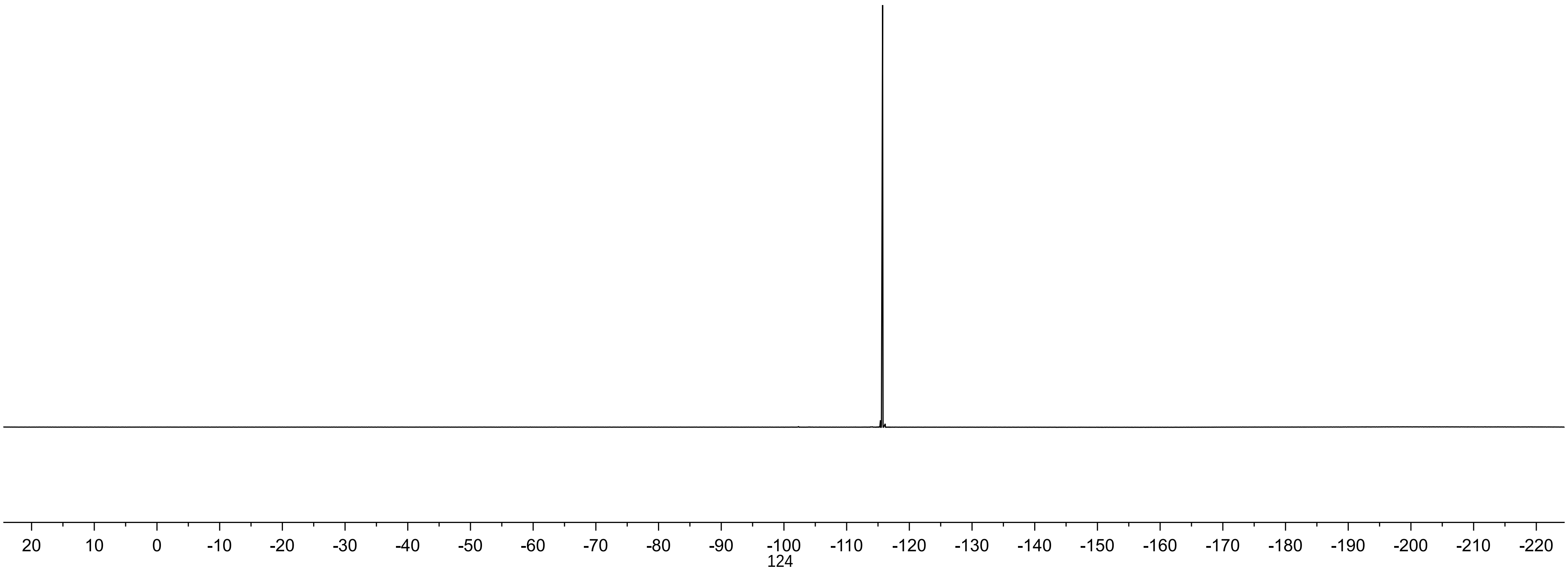

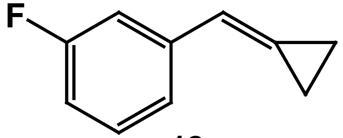

12

[1]**H NMR** spectrum (400 MHz, $CDCl_3$)

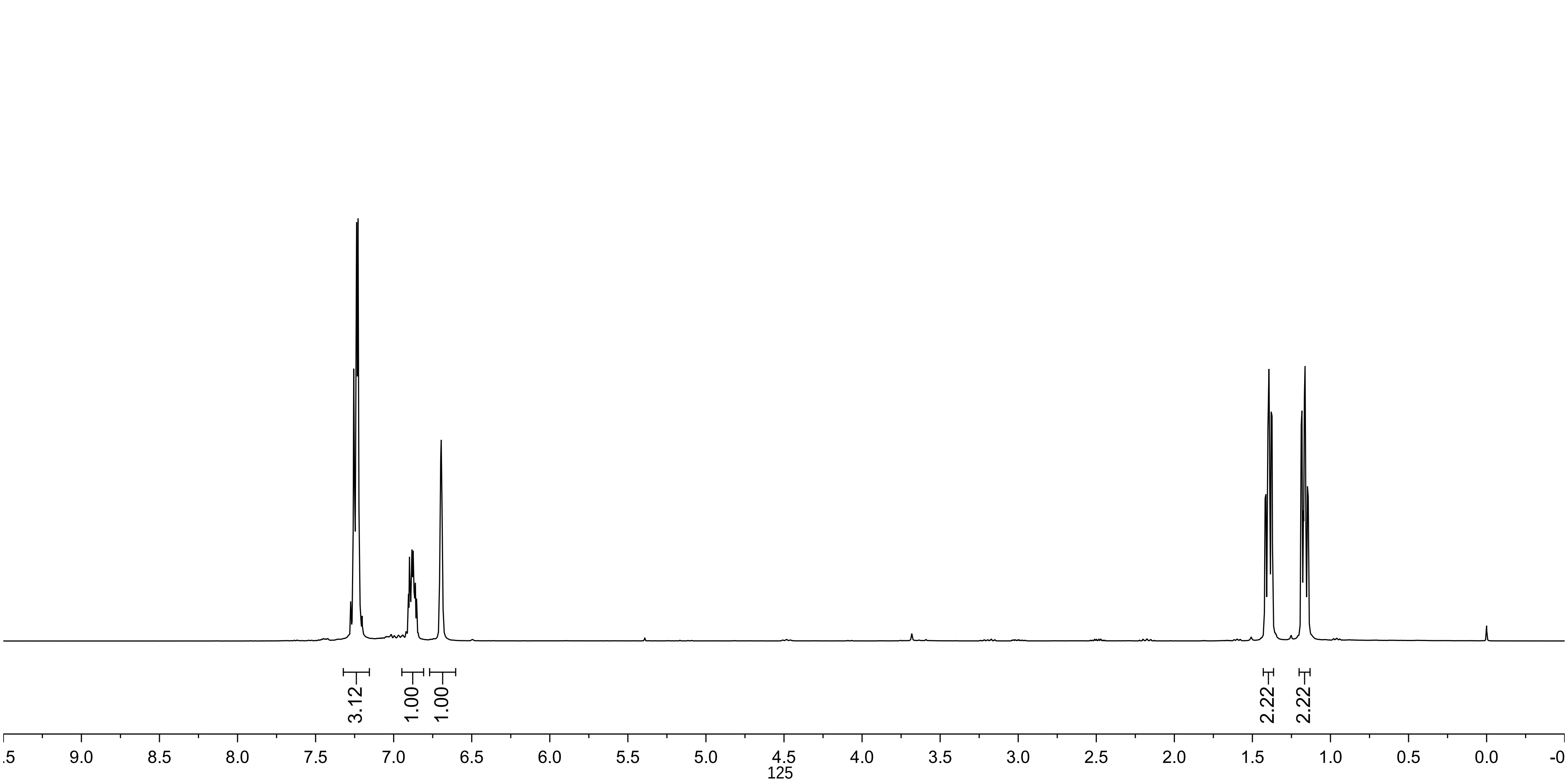

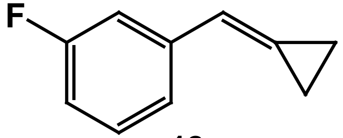

12

^{13}C NMR spectrum (100 MHz, $CDCl_3$)

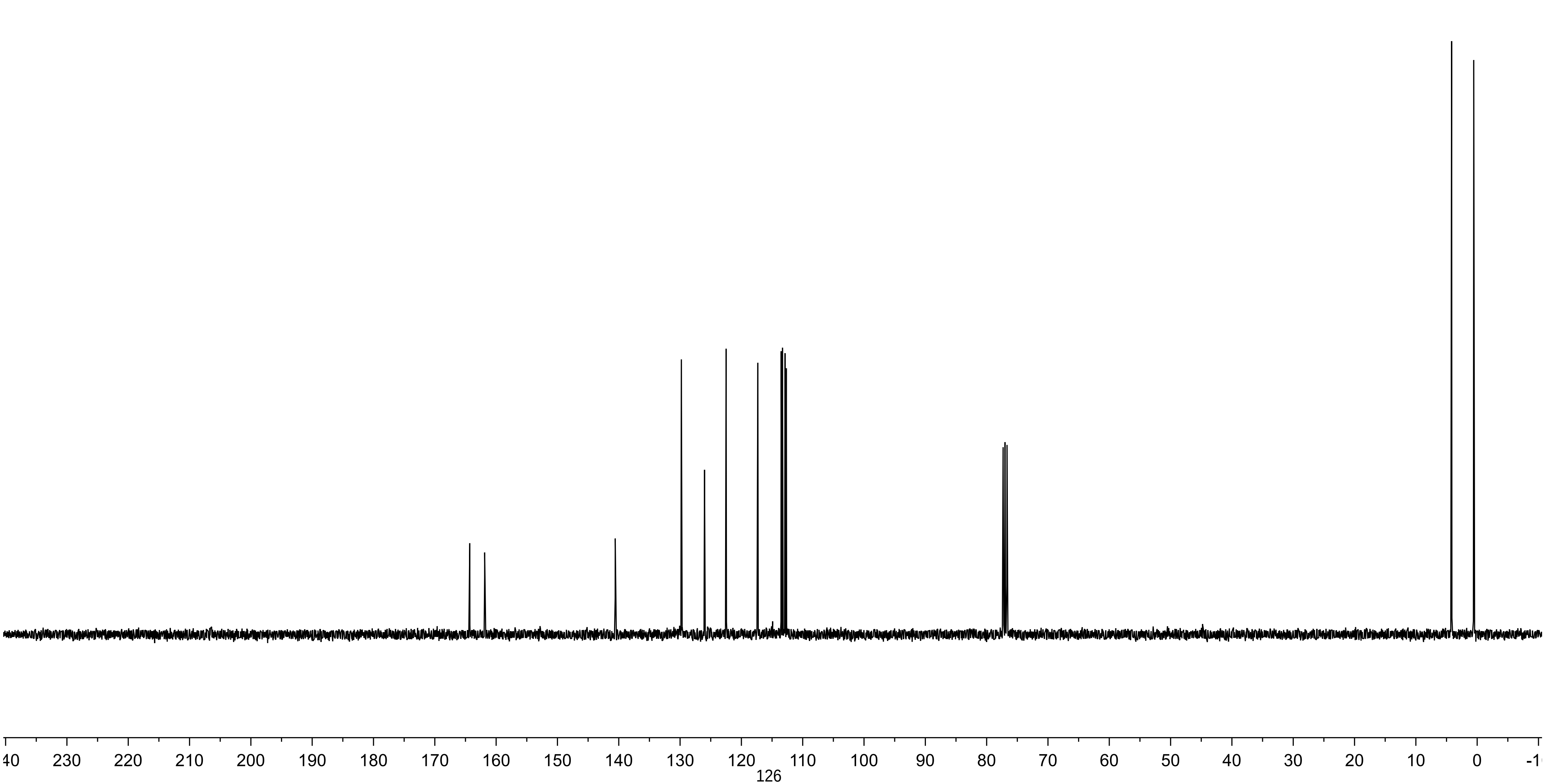

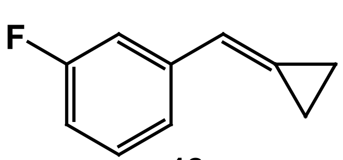

12

^{19}**F NMR** spectrum (376 MHz, $CDCl_3$)

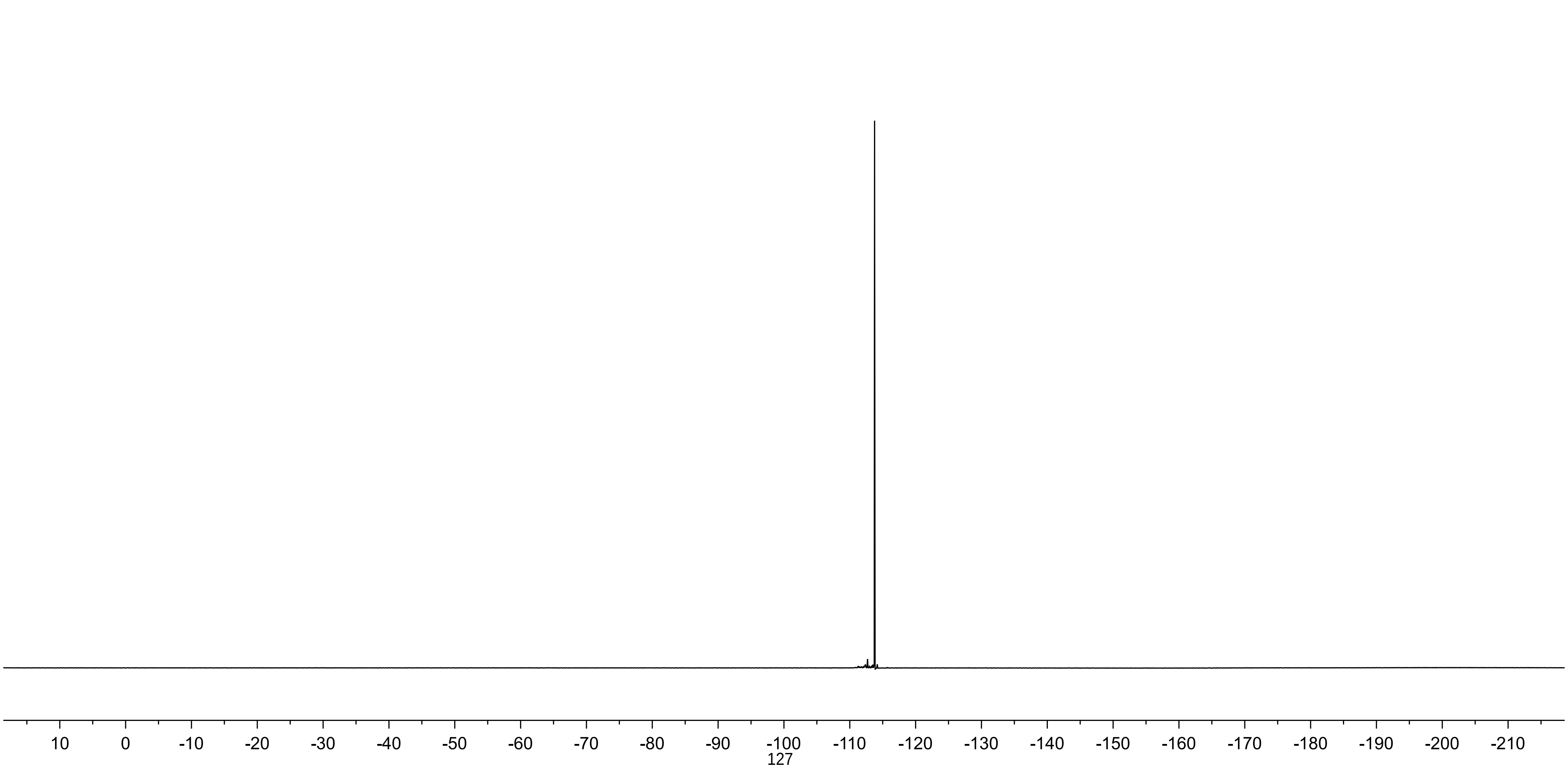

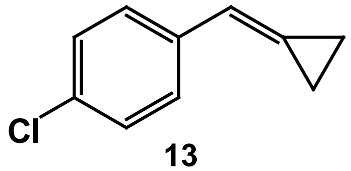

13

^{1}H NMR spectrum (600 MHz, $CDCl_3$)

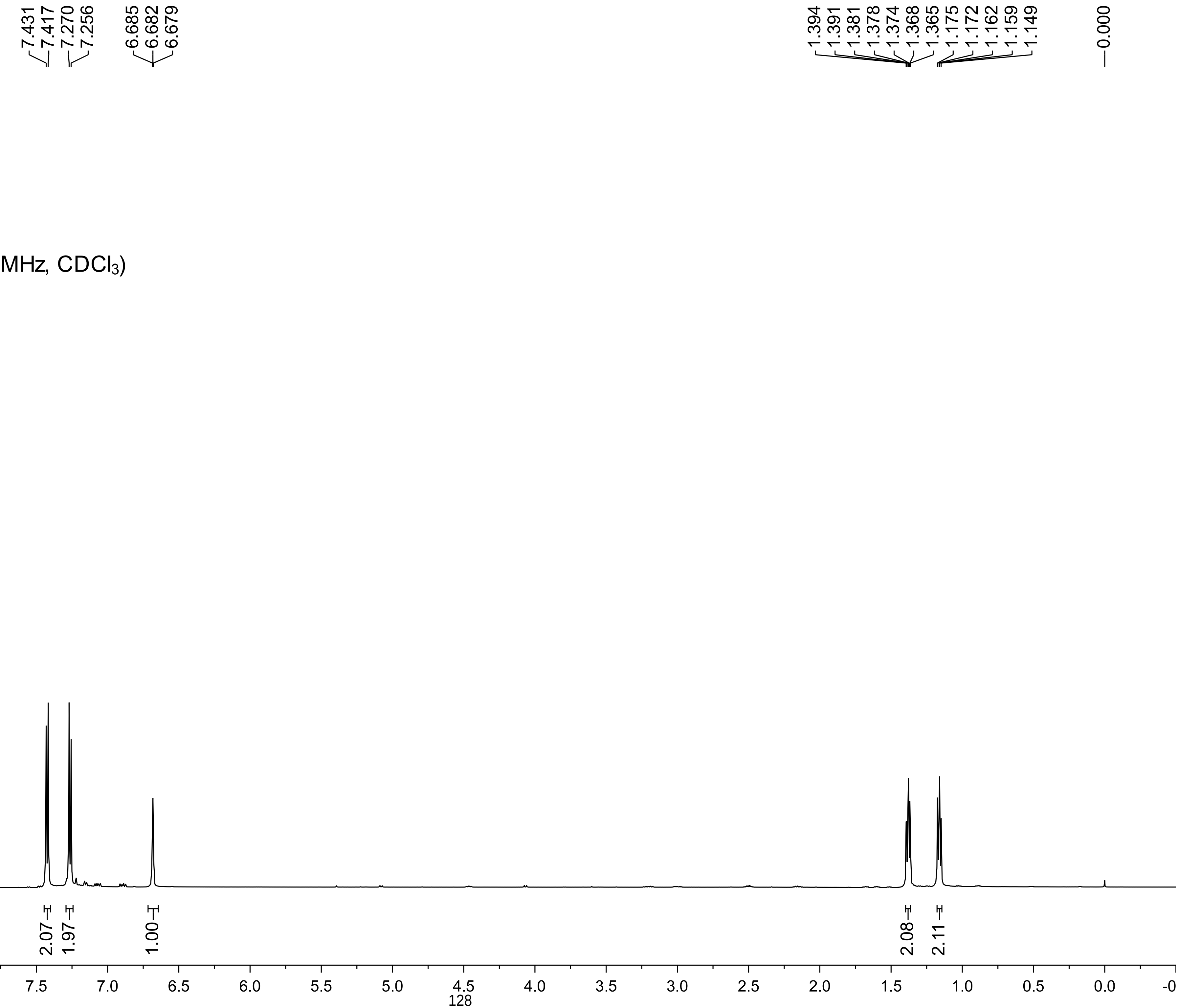

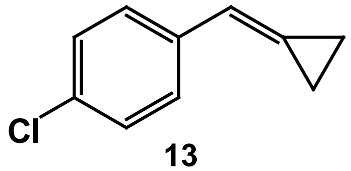

13

^{13}C NMR spectrum (150 MHz, $CDCl_3$)

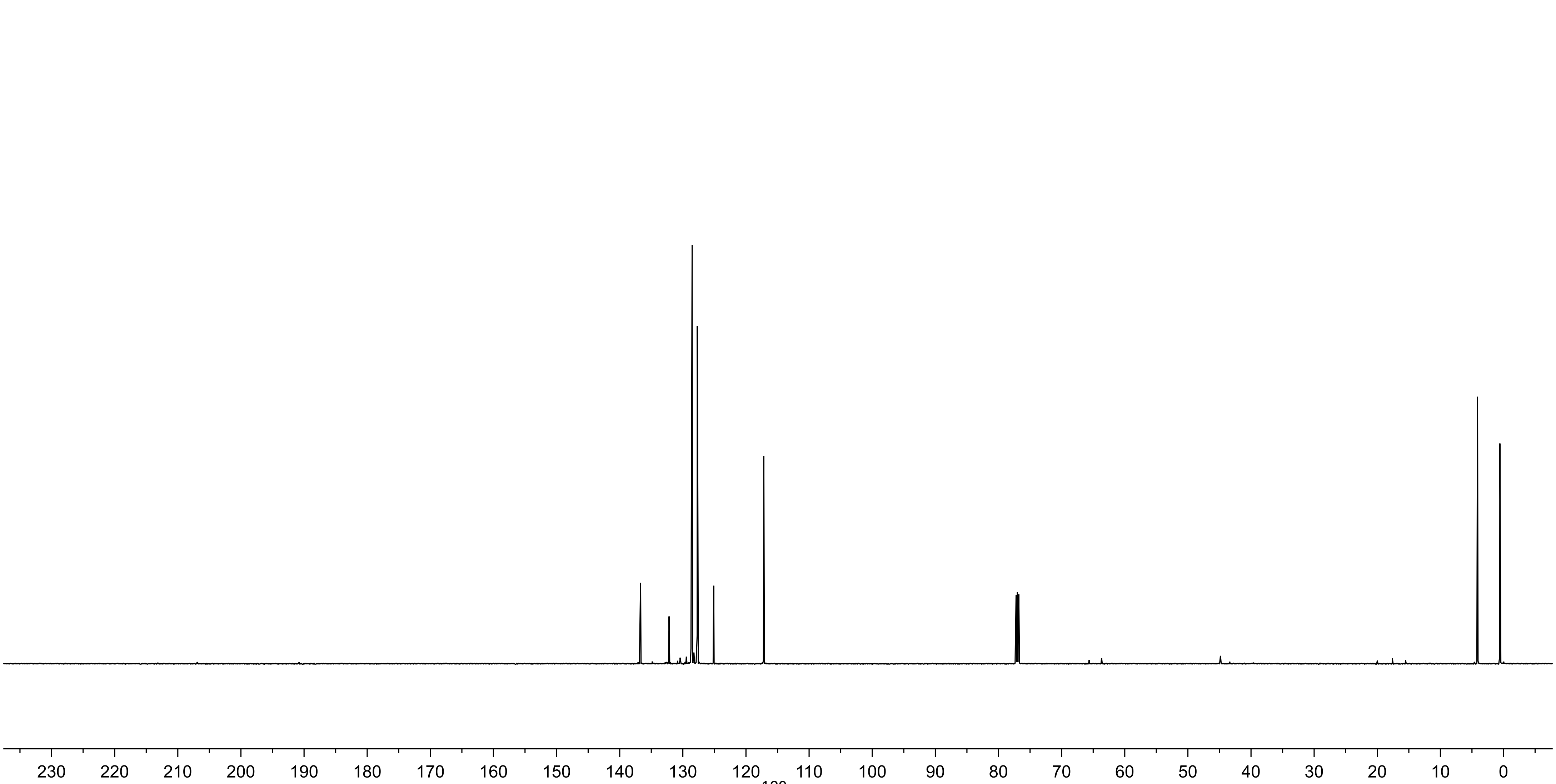

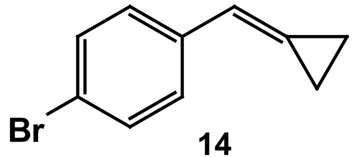

14

[1]**H NMR** spectrum (600 MHz, $CDCl_3$)

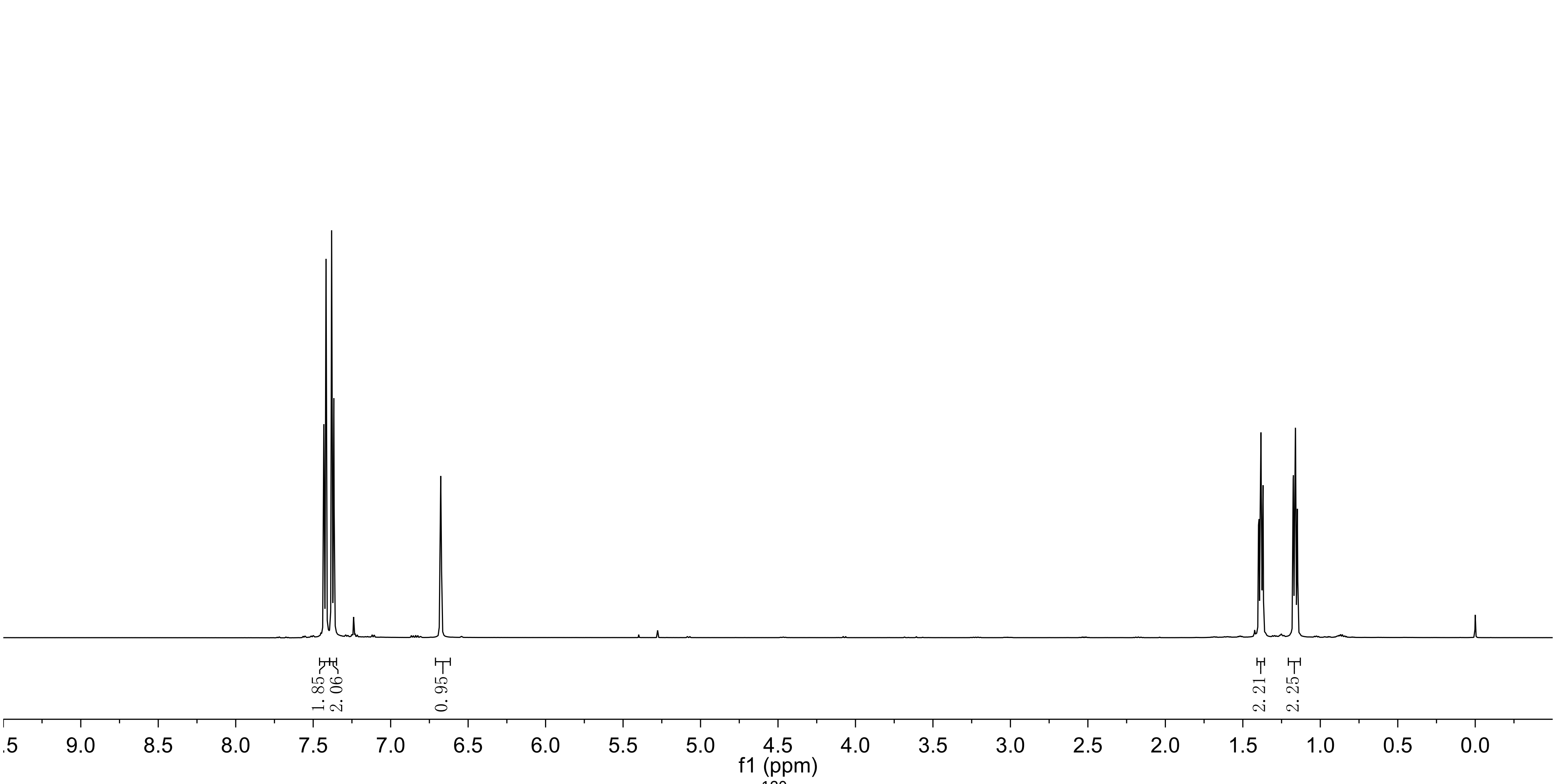

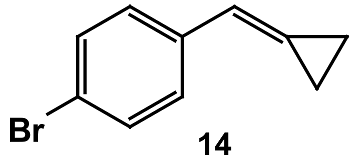

14

^{13}C NMR spectrum (150 MHz, $CDCl_3$)

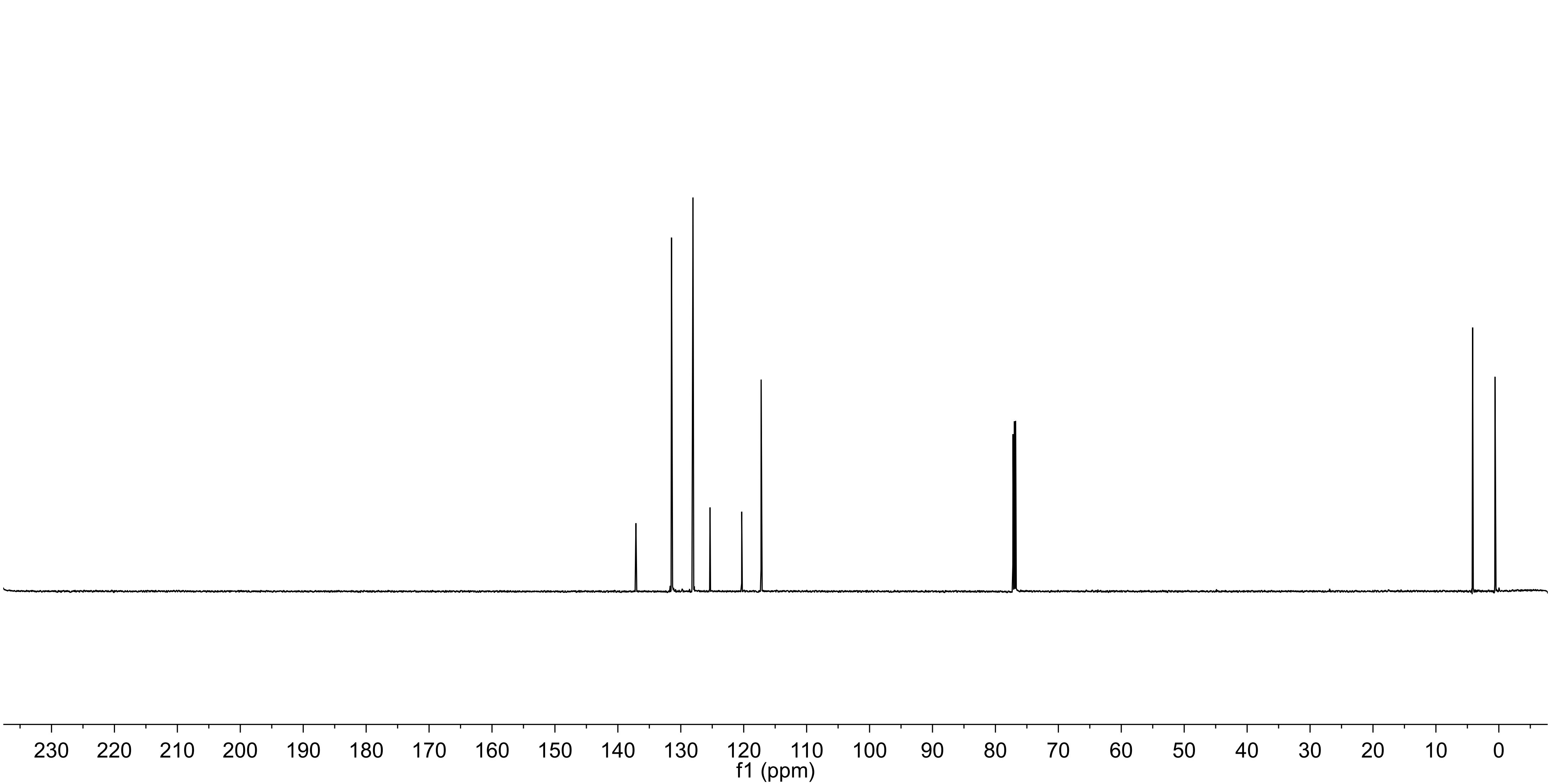

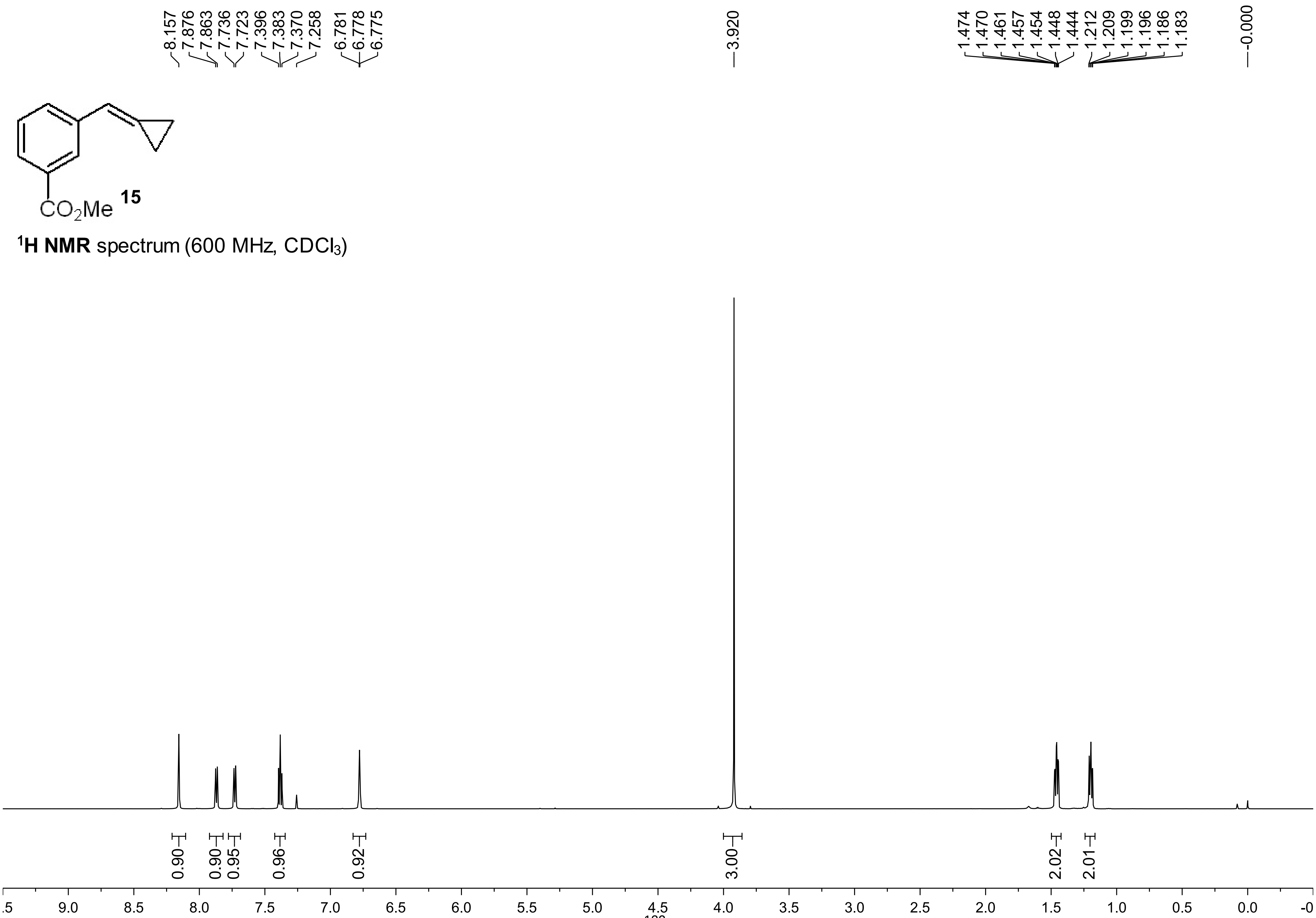

15

^{1}H NMR spectrum (600 MHz, $CDCl_3$)

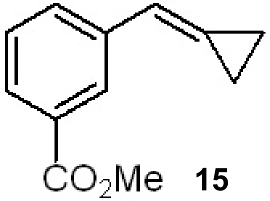

15

[13]**C NMR** spectrum (150 MHz, $CDCl_3$)

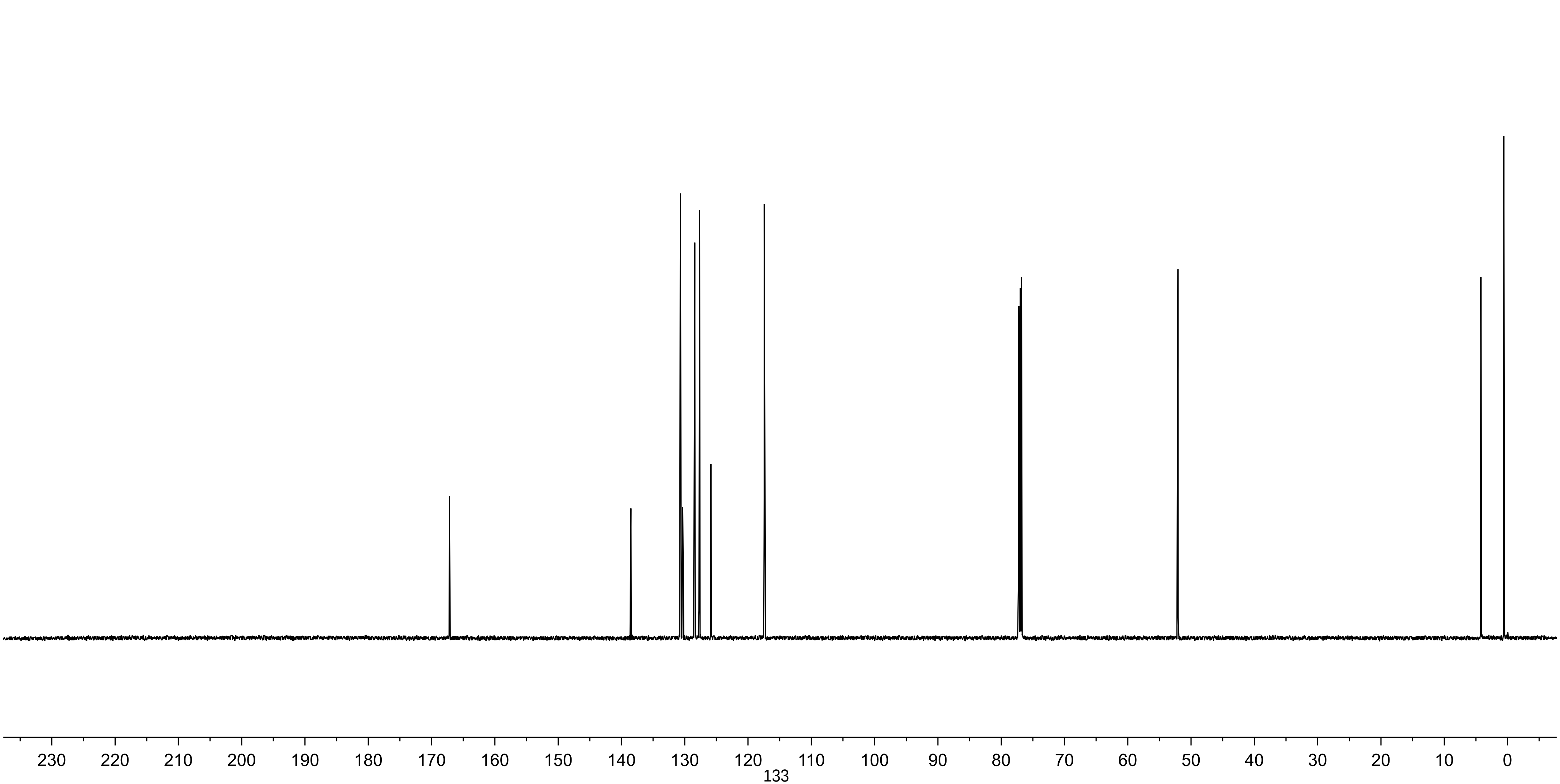

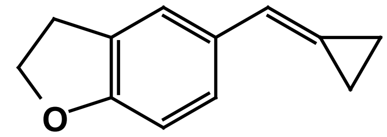

16

^{1}H NMR spectrum (400 MHz, $CDCl_3$)

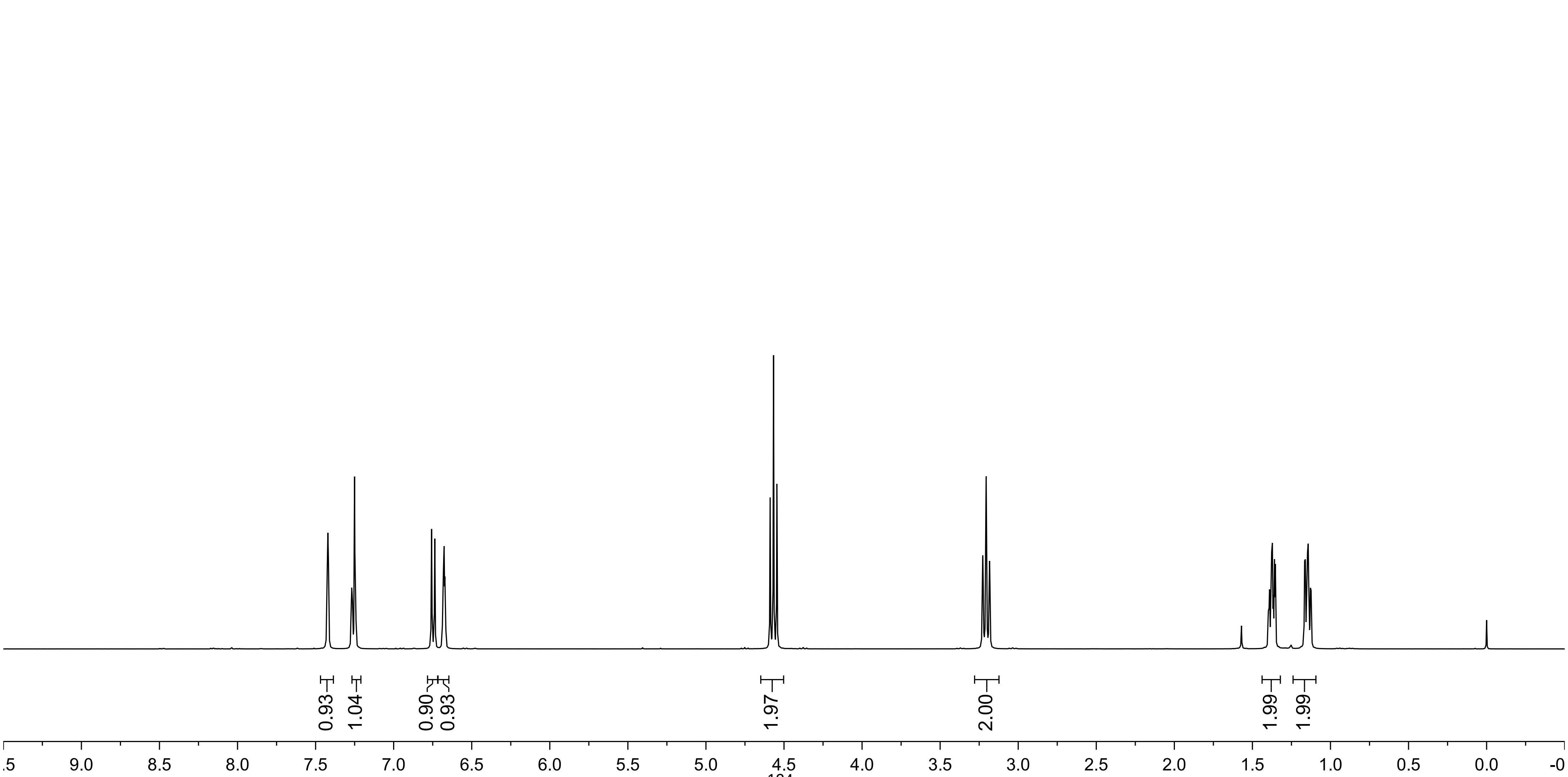

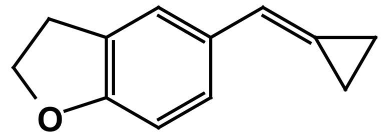

16

^{13}**C NMR** spectrum (100 MHz, $CDCl_3$)

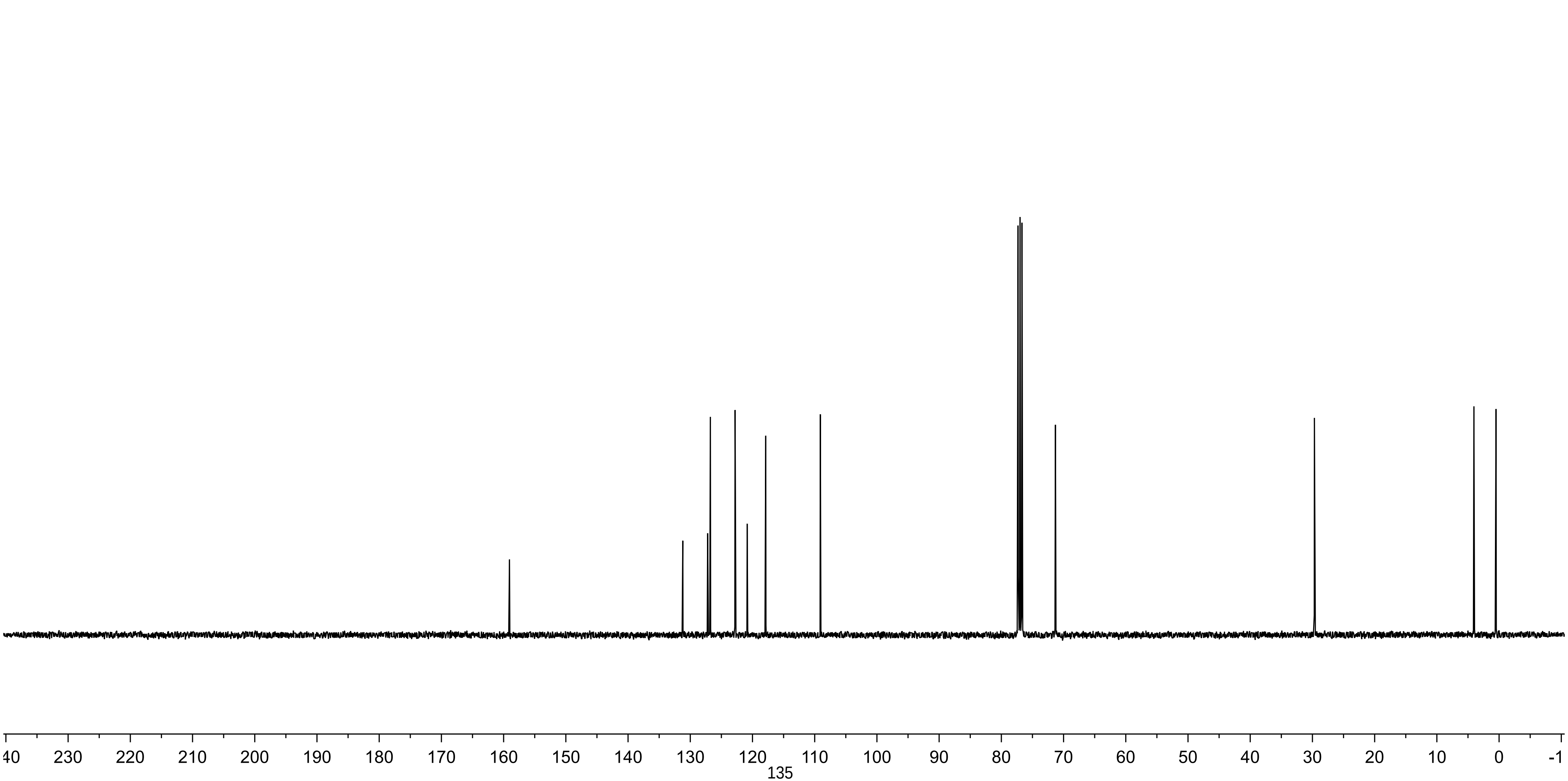

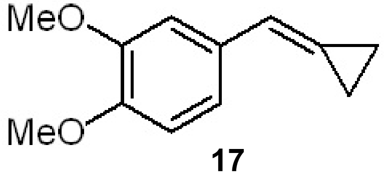

17

[1]**H NMR** spectrum (600 MHz, $CDCl_3$)

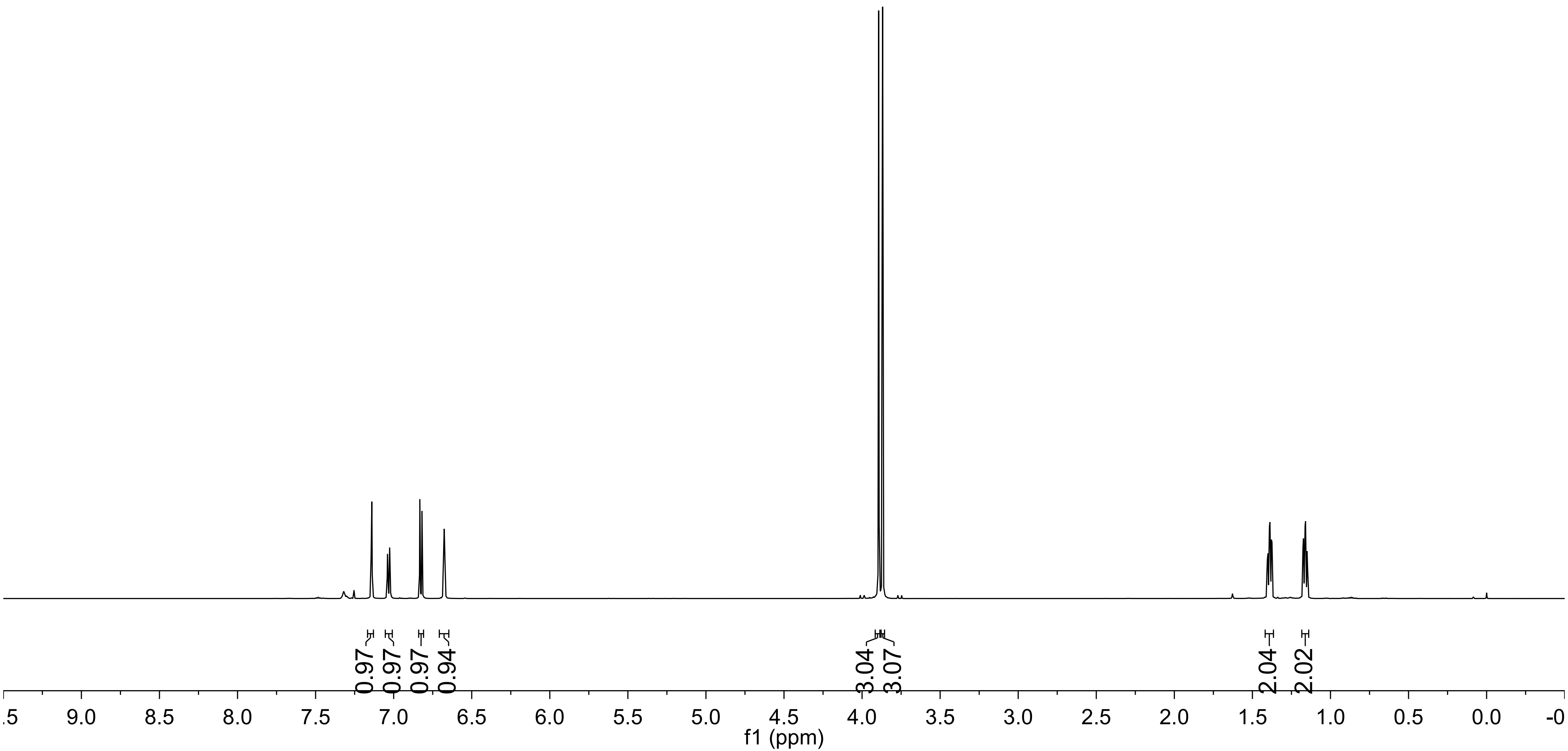

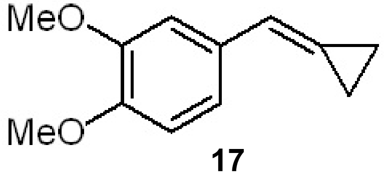

17

^{13}C NMR spectrum (150 MHz, $CDCl_3$)

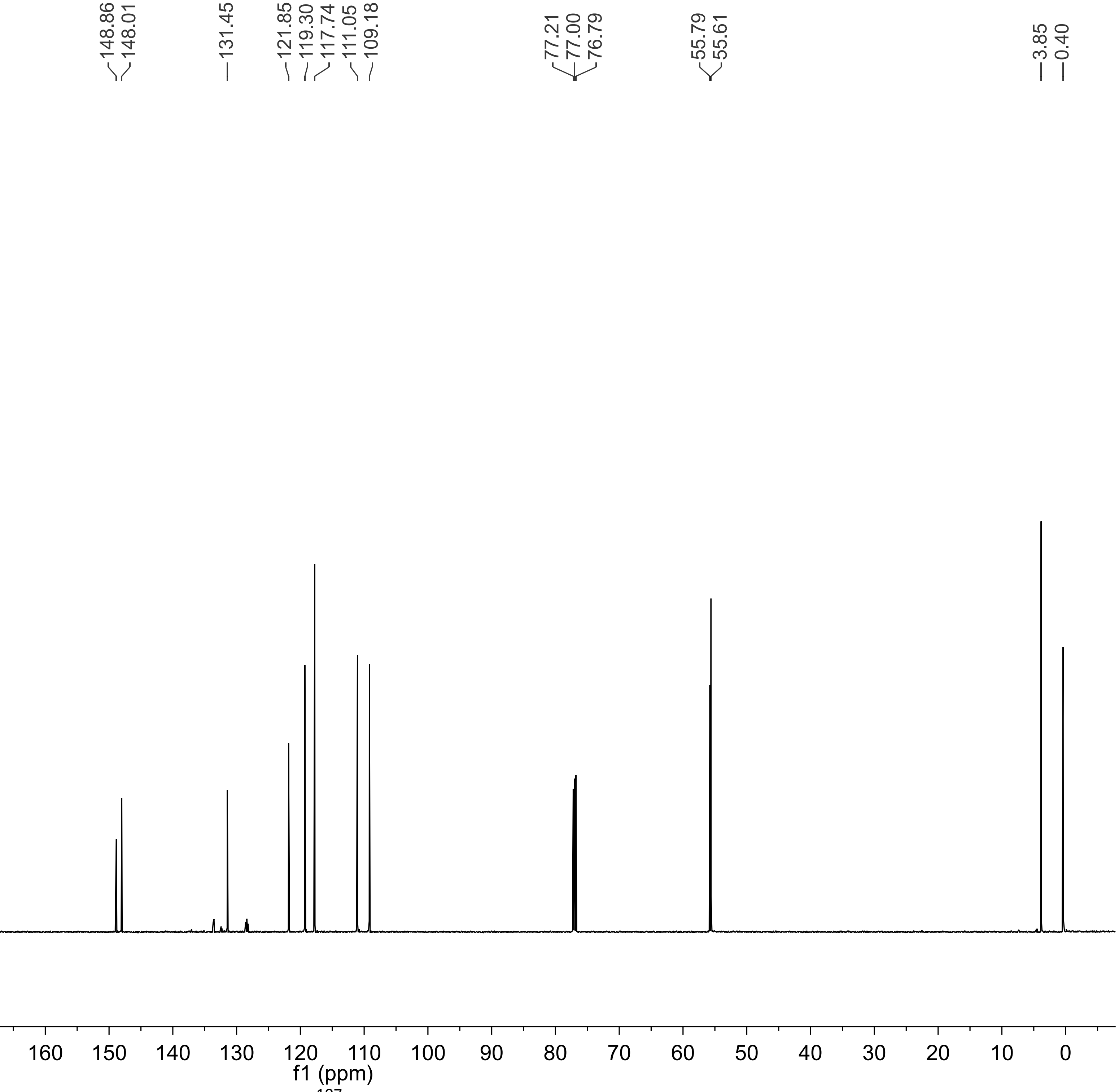

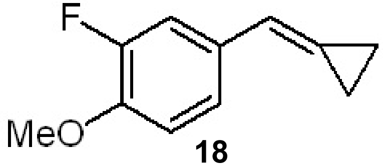

18

[1]**H NMR** spectrum (600 MHz, $CDCl_3$)

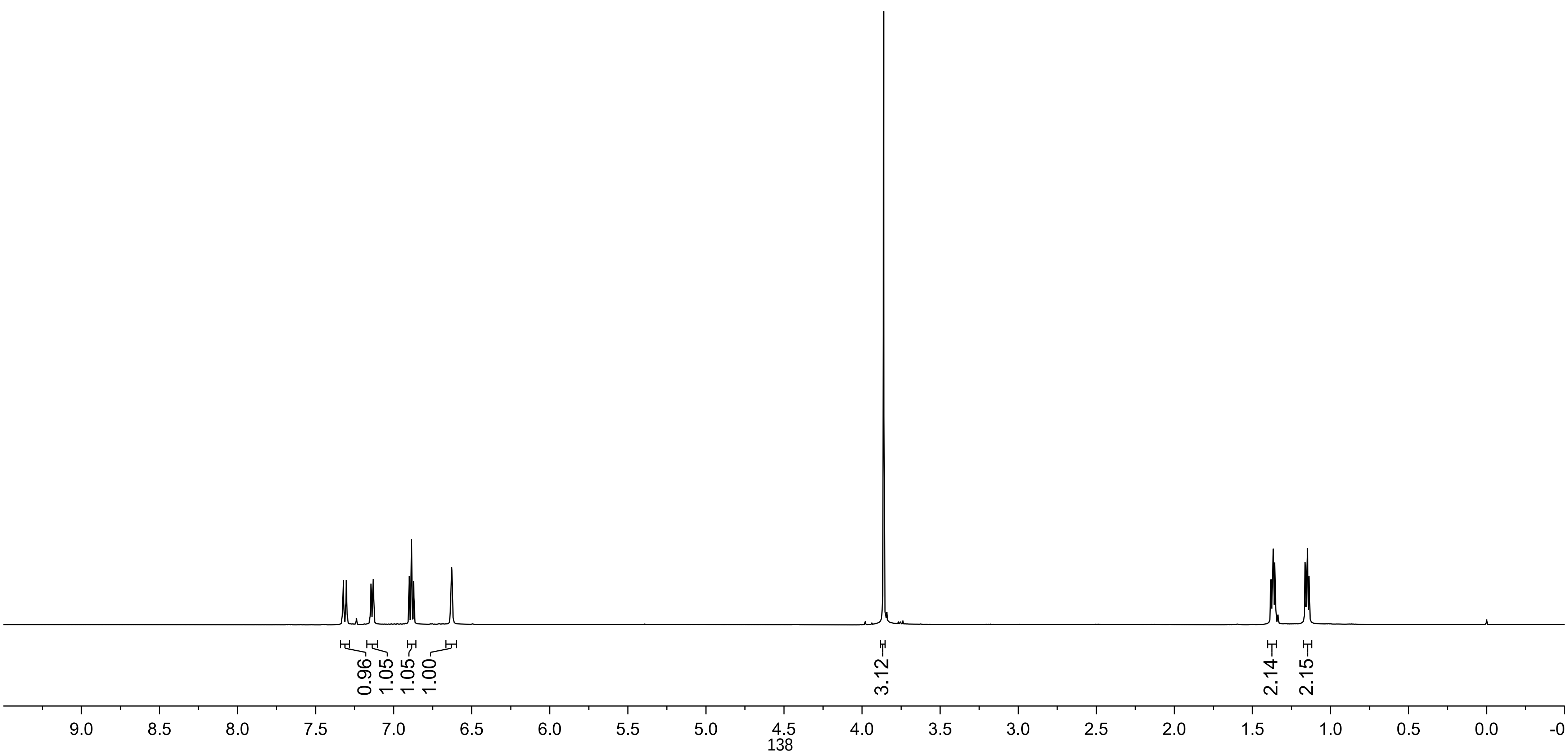

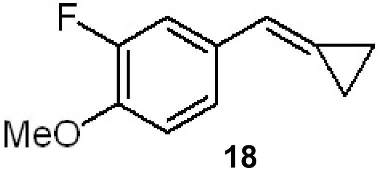

18

^{13}C NMR spectrum (150 MHz, $CDCl_3$)

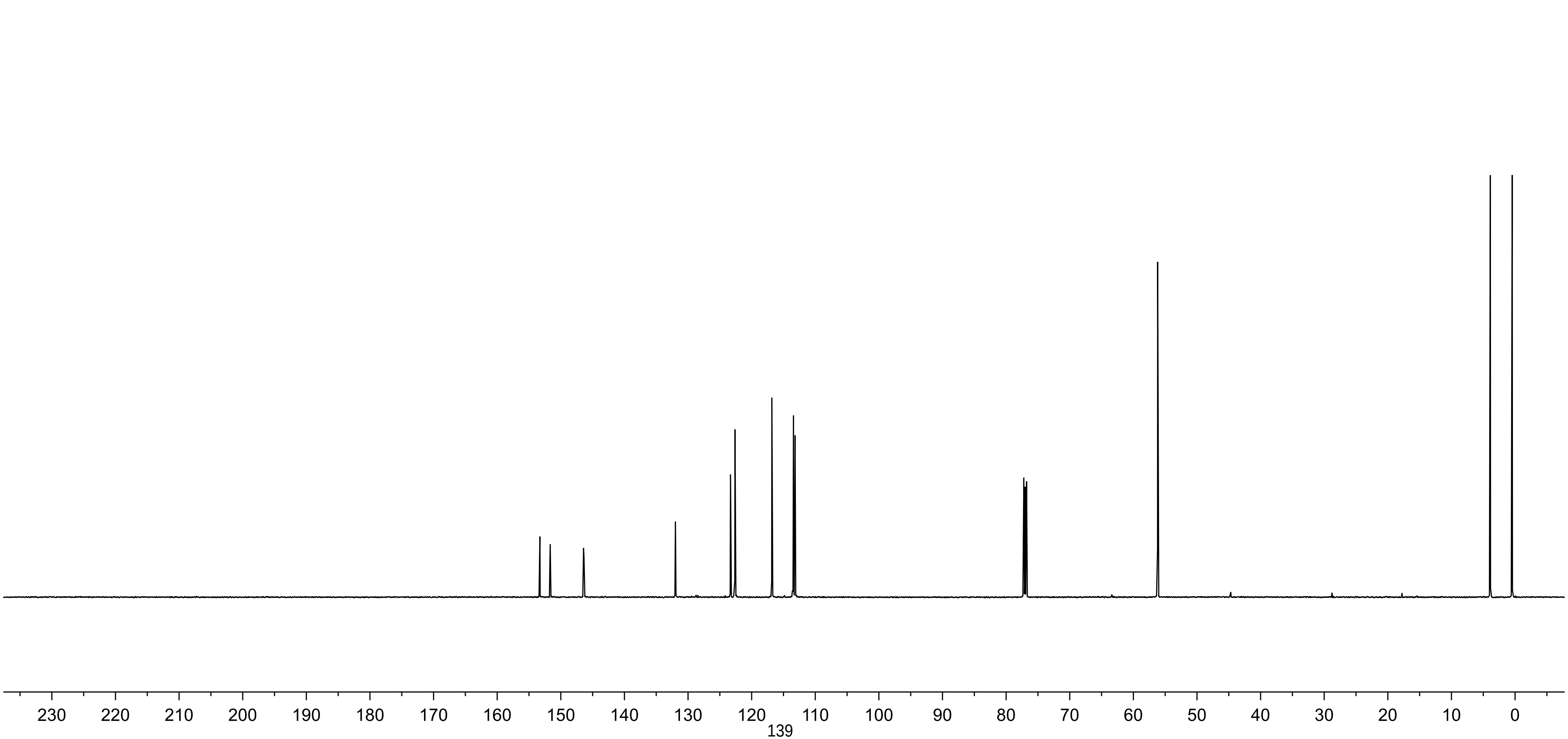

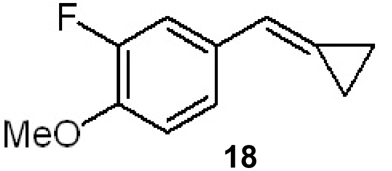

18

[19]**F NMR** spectrum (376 MHz, $CDCl_3$)

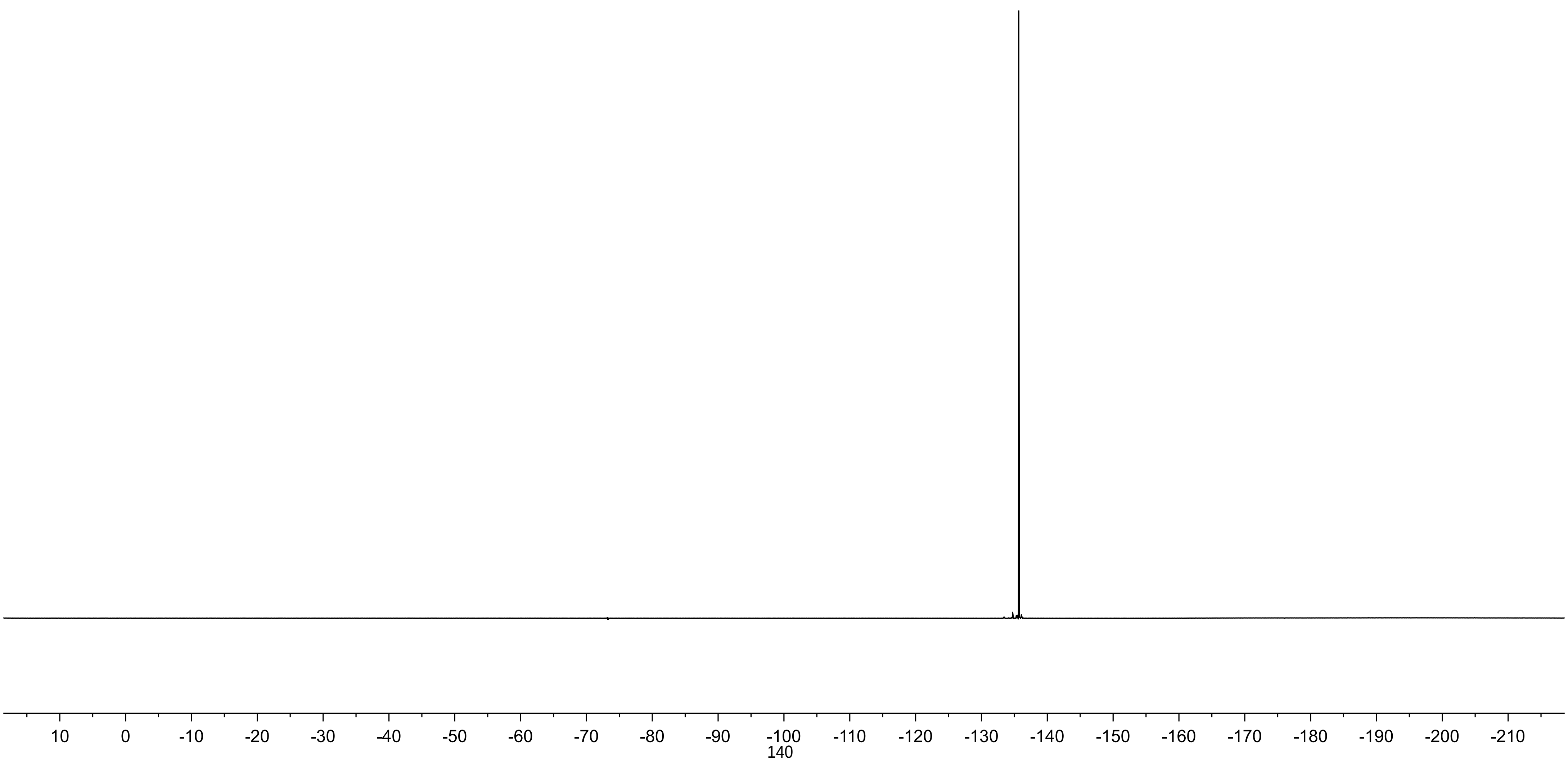

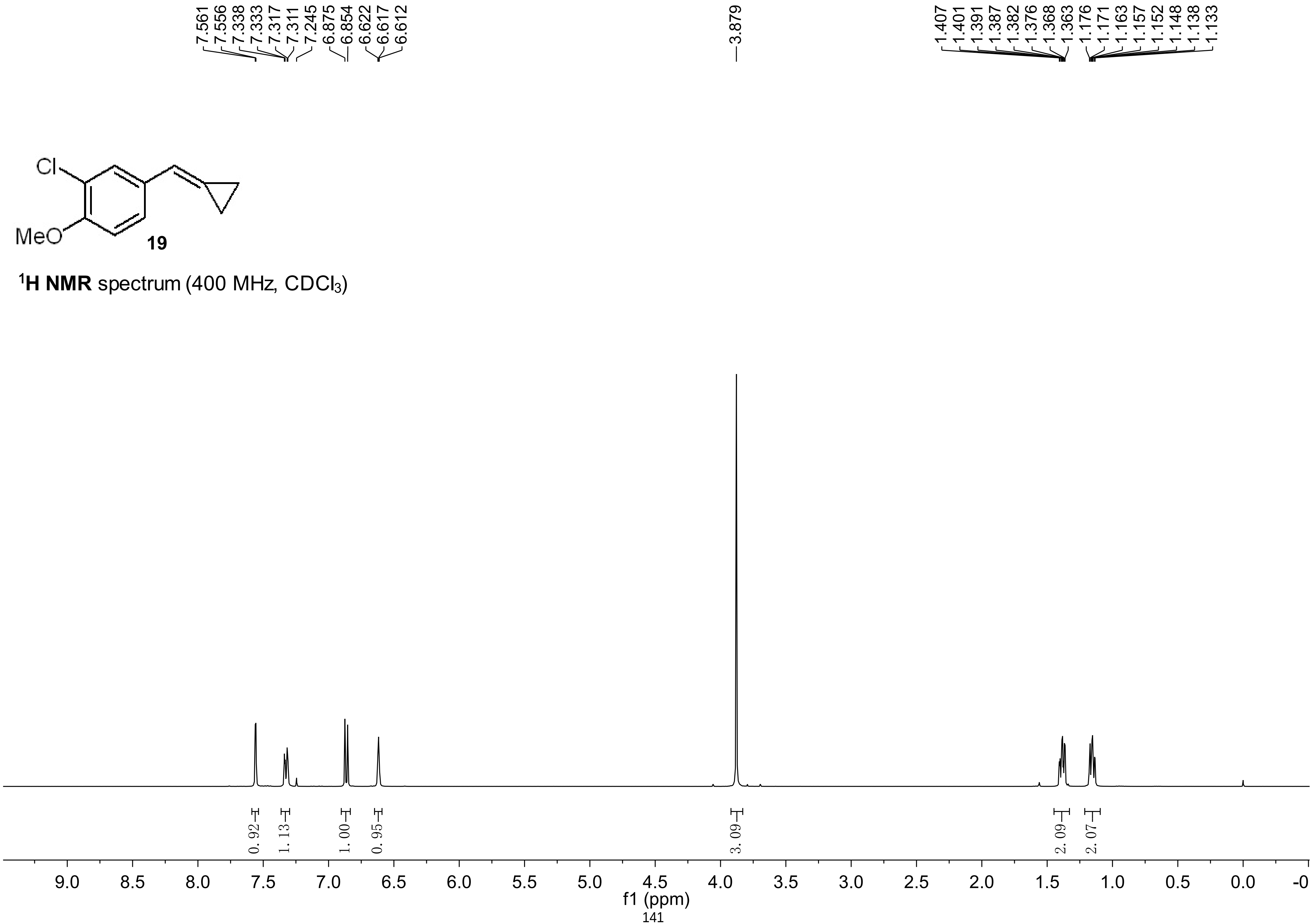

^{1}H NMR spectrum (400 MHz, $CDCl_3$)

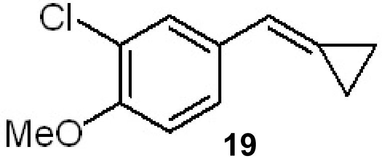

19

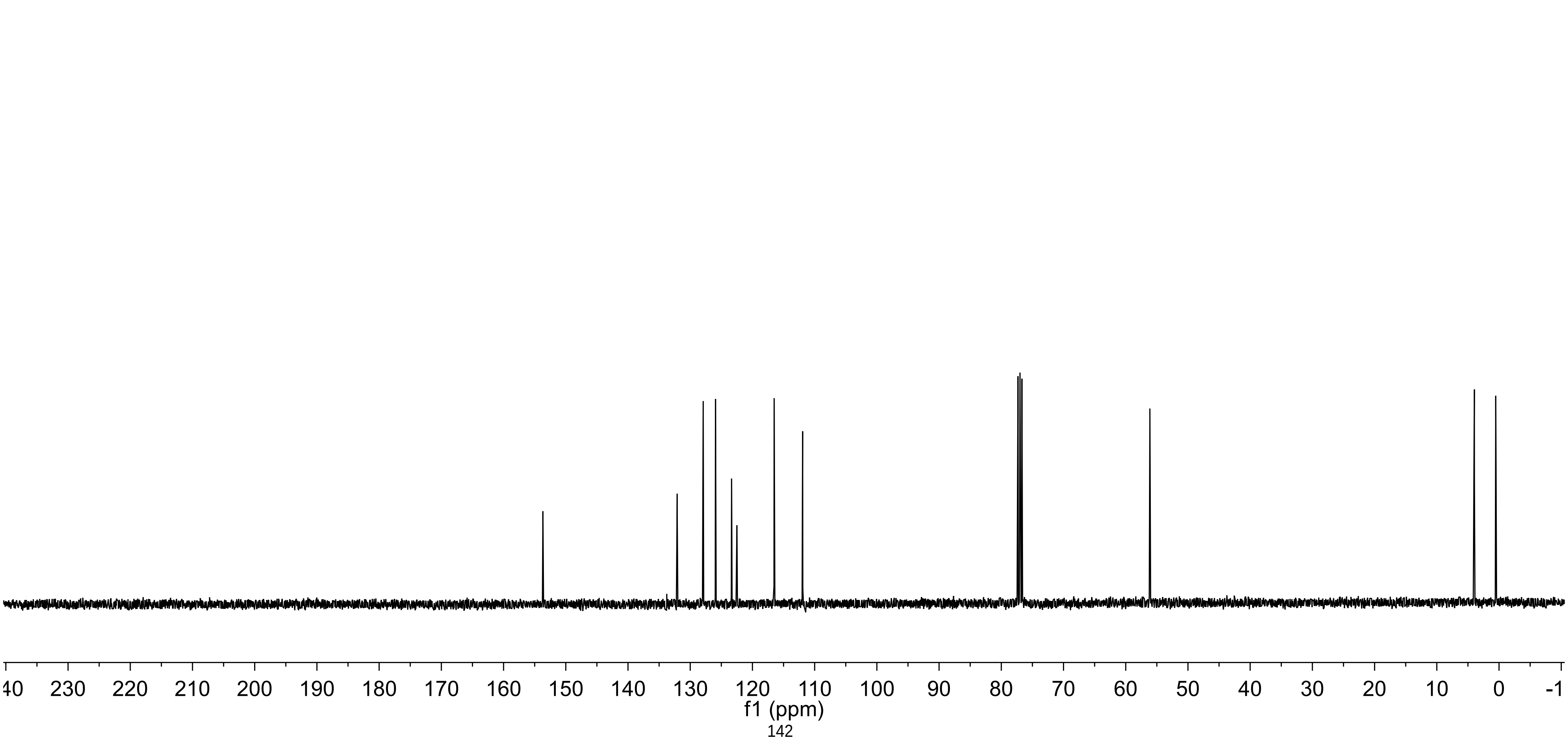

^{13}**C NMR** spectrum (100 MHz, $CDCl_3$)

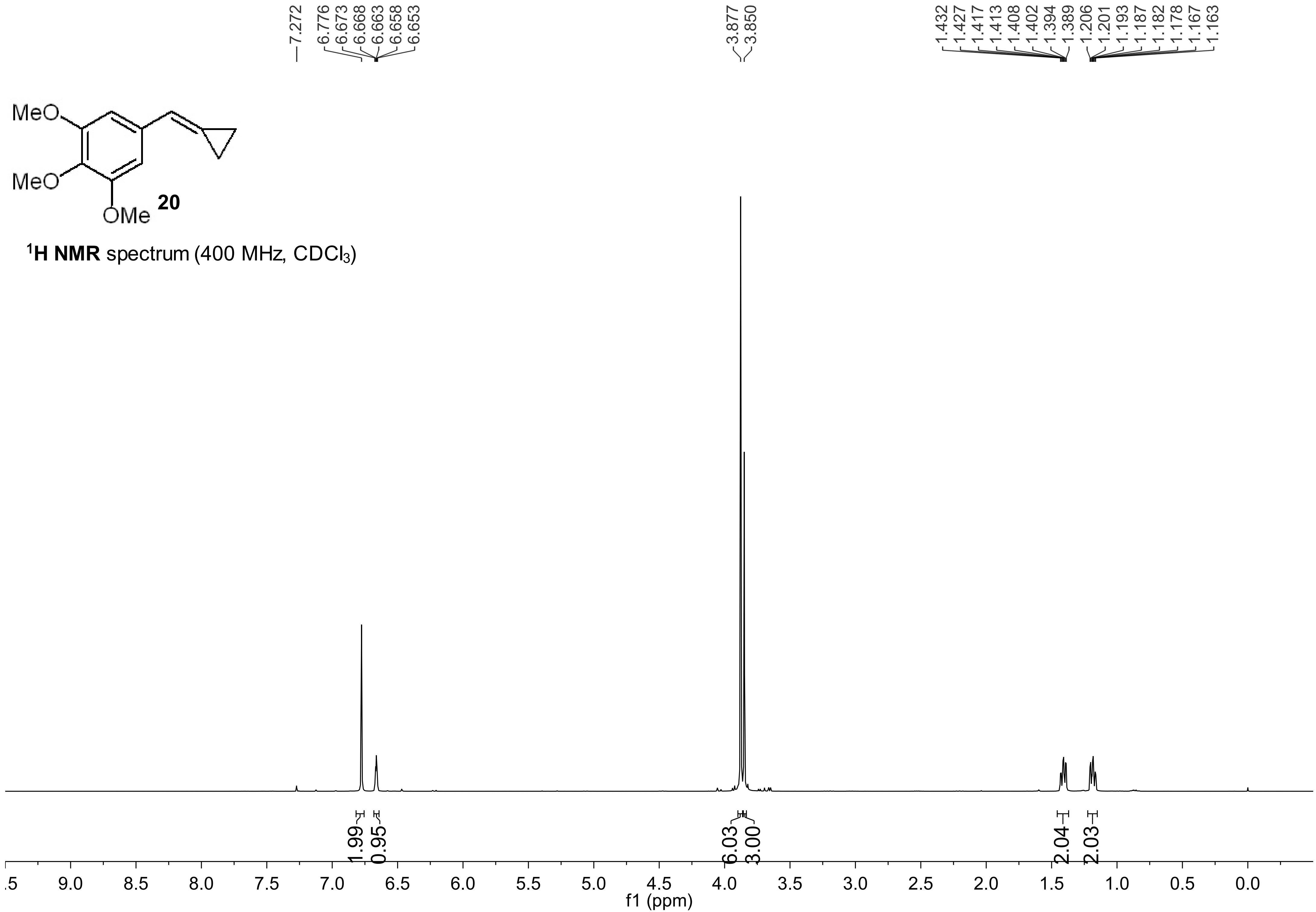

^{1}H NMR spectrum (400 MHz, $CDCl_3$)

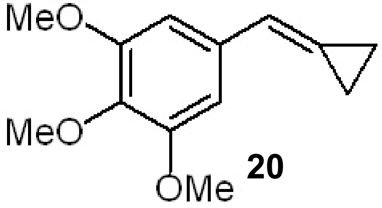

20

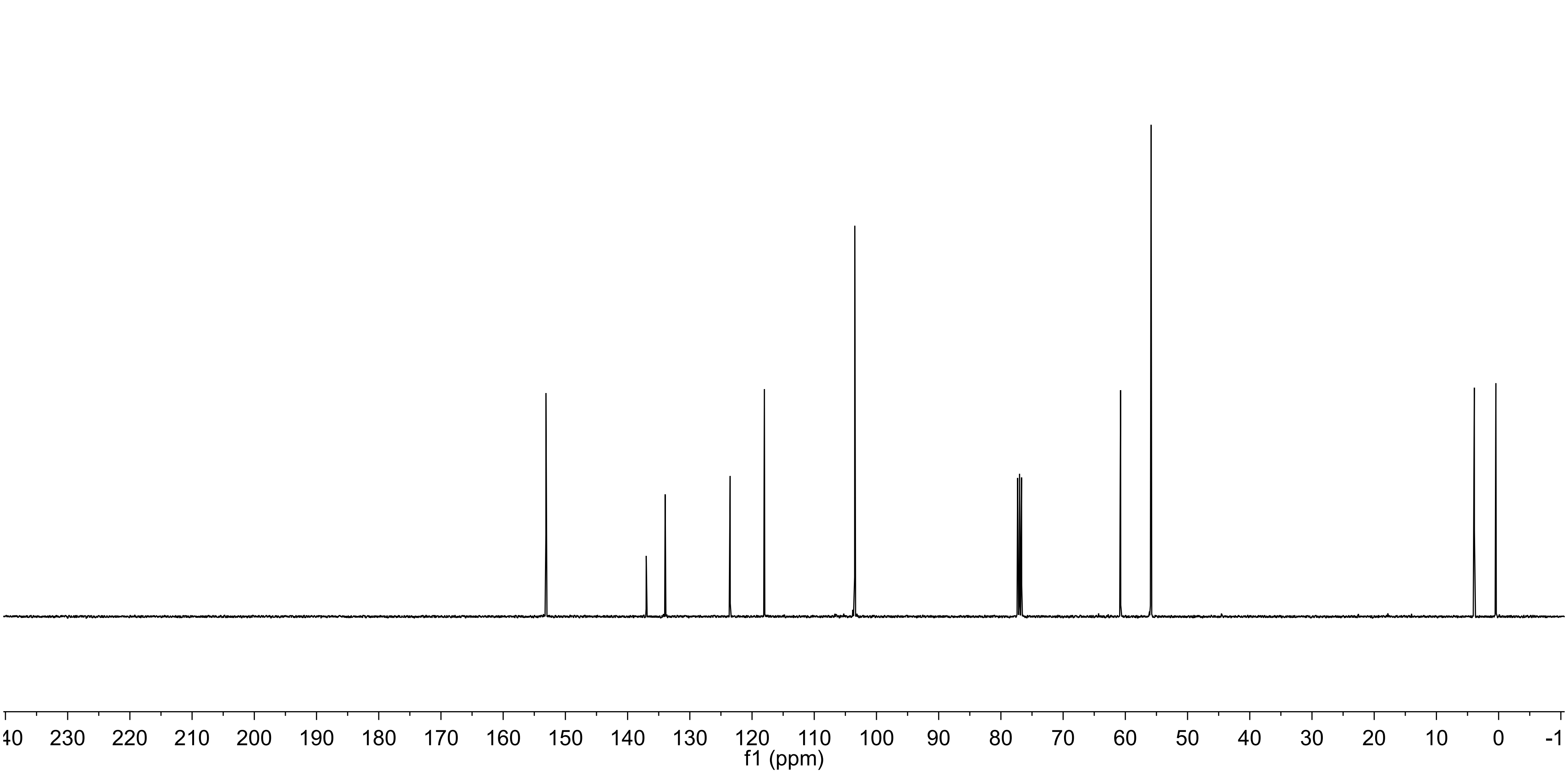

^{13}**C NMR** spectrum (100 MHz, $CDCl_3$)

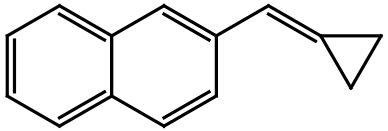

21

[1]**H NMR** spectrum (400 MHz, $CDCl_3$)

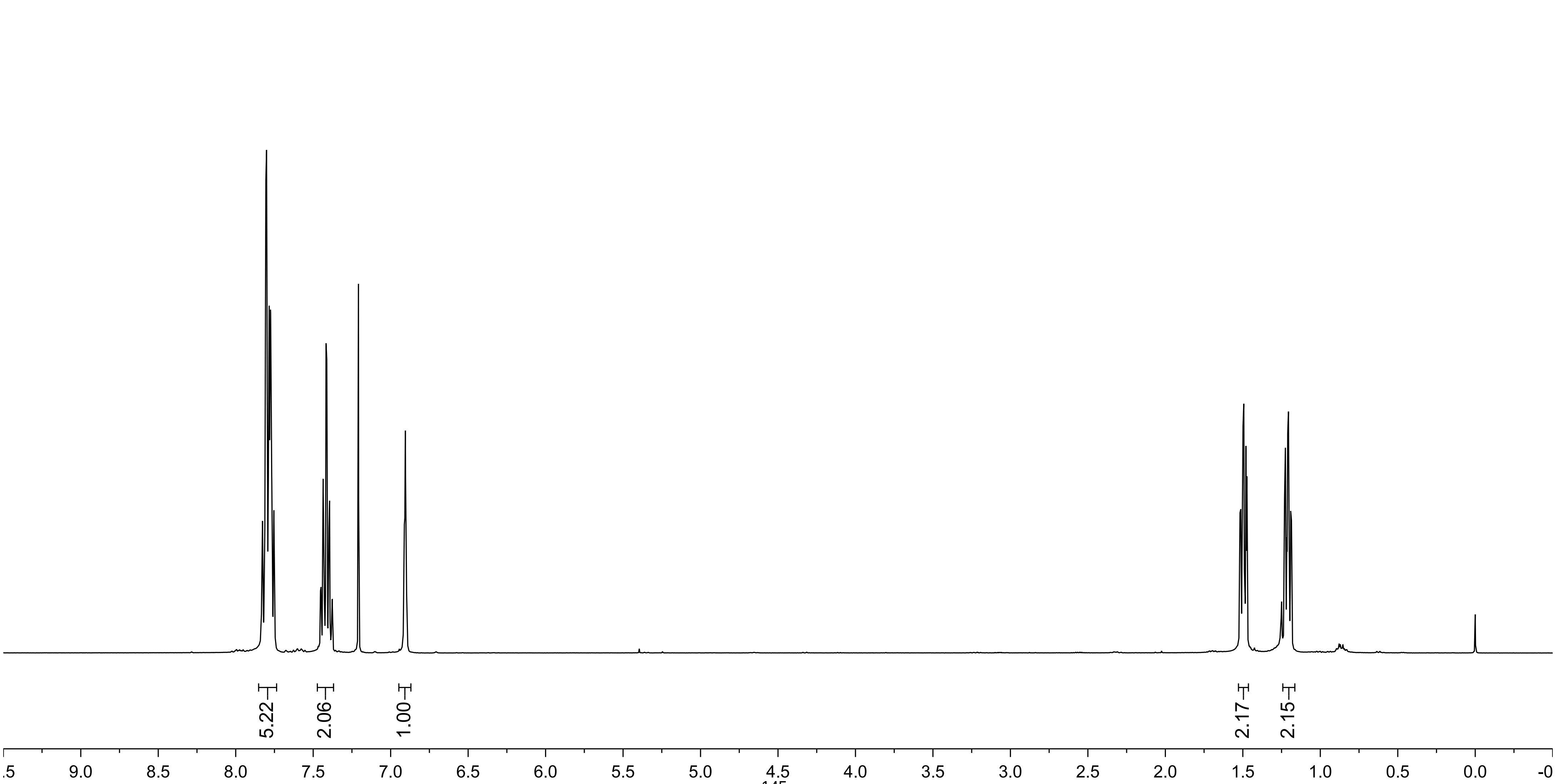

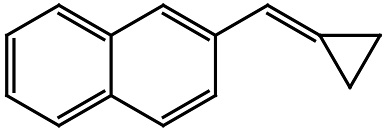

21

^{13}C NMR spectrum (100 MHz, $CDCl_3$)

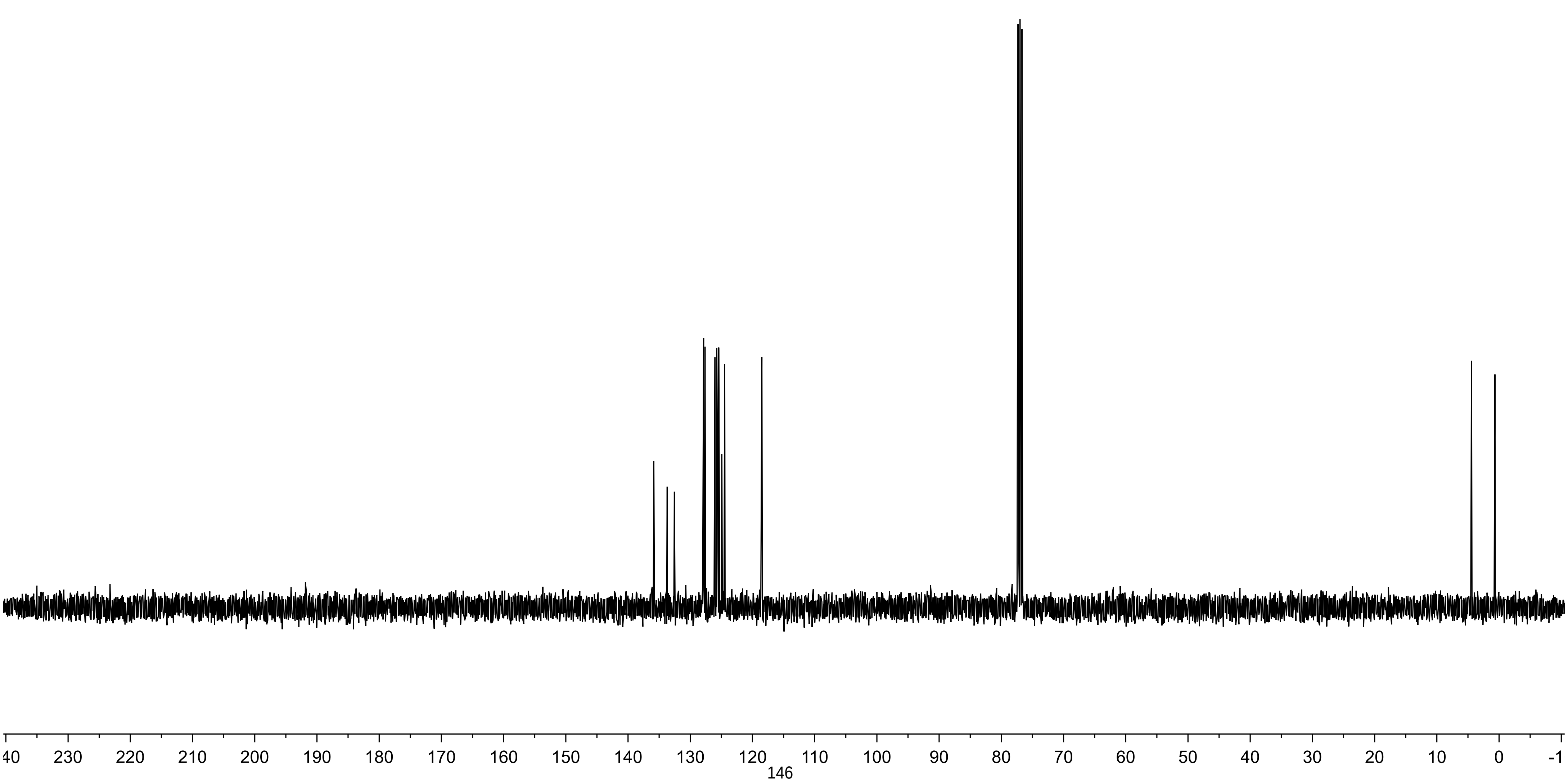

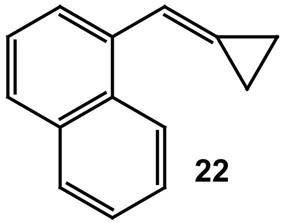

22

[1]**H NMR** spectrum (400 MHz, $CDCl_3$)

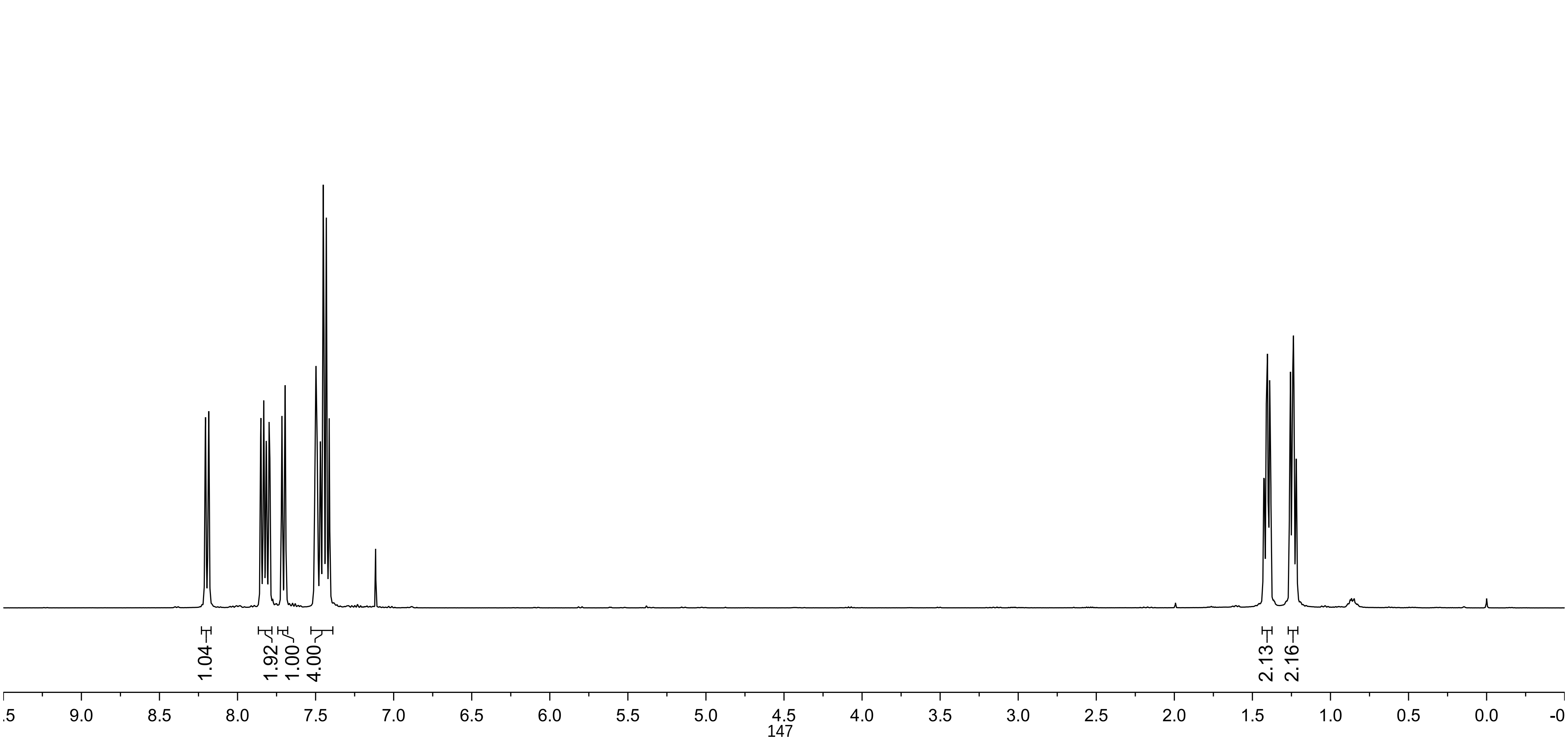

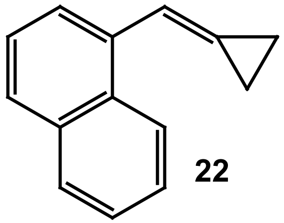

22

^{13}**C NMR** spectrum (100 MHz, $CDCl_3$)

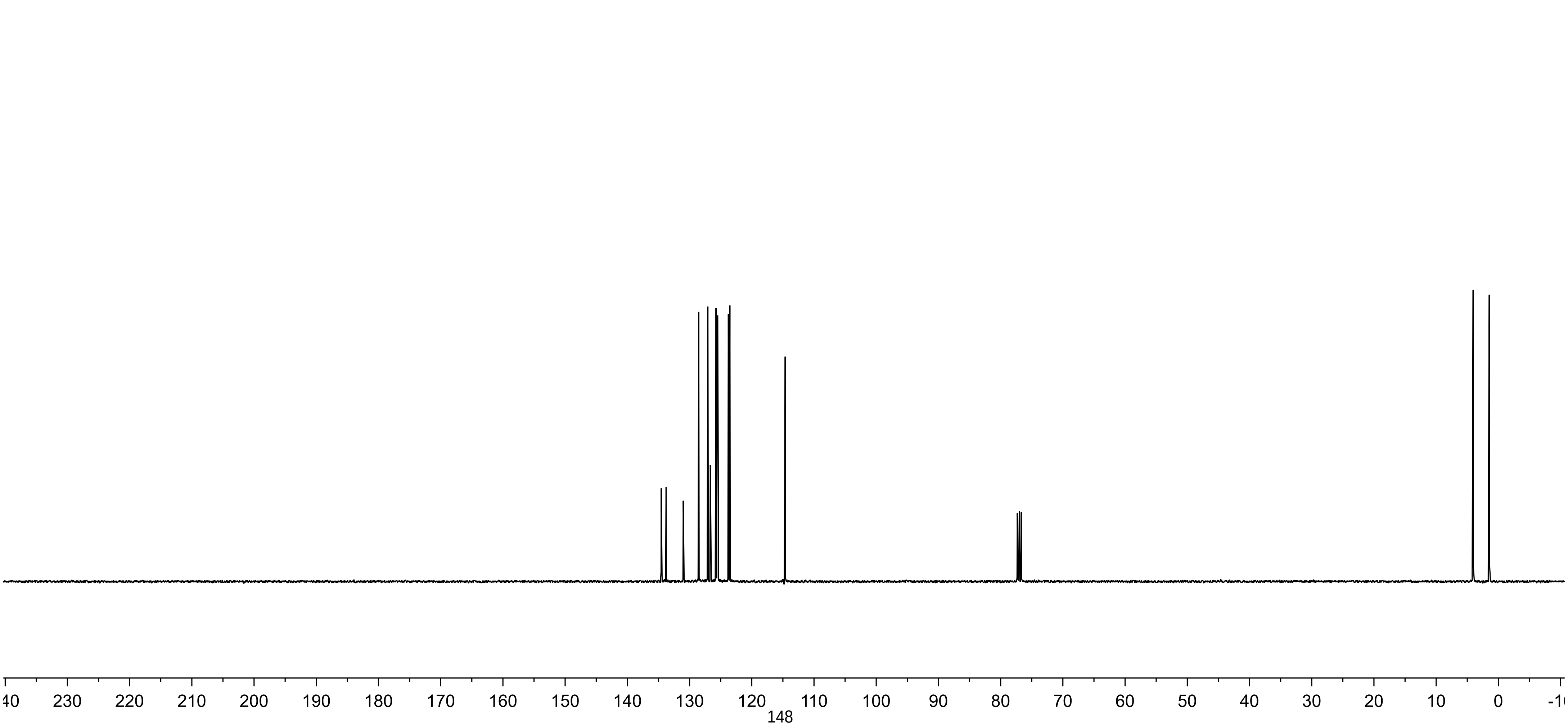

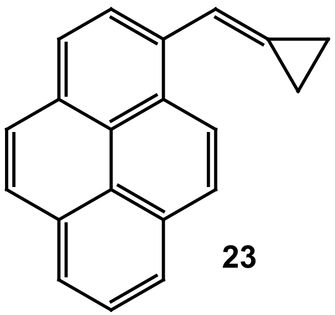

23

[1]**H NMR** spectrum (400 MHz, $CDCl_3$)

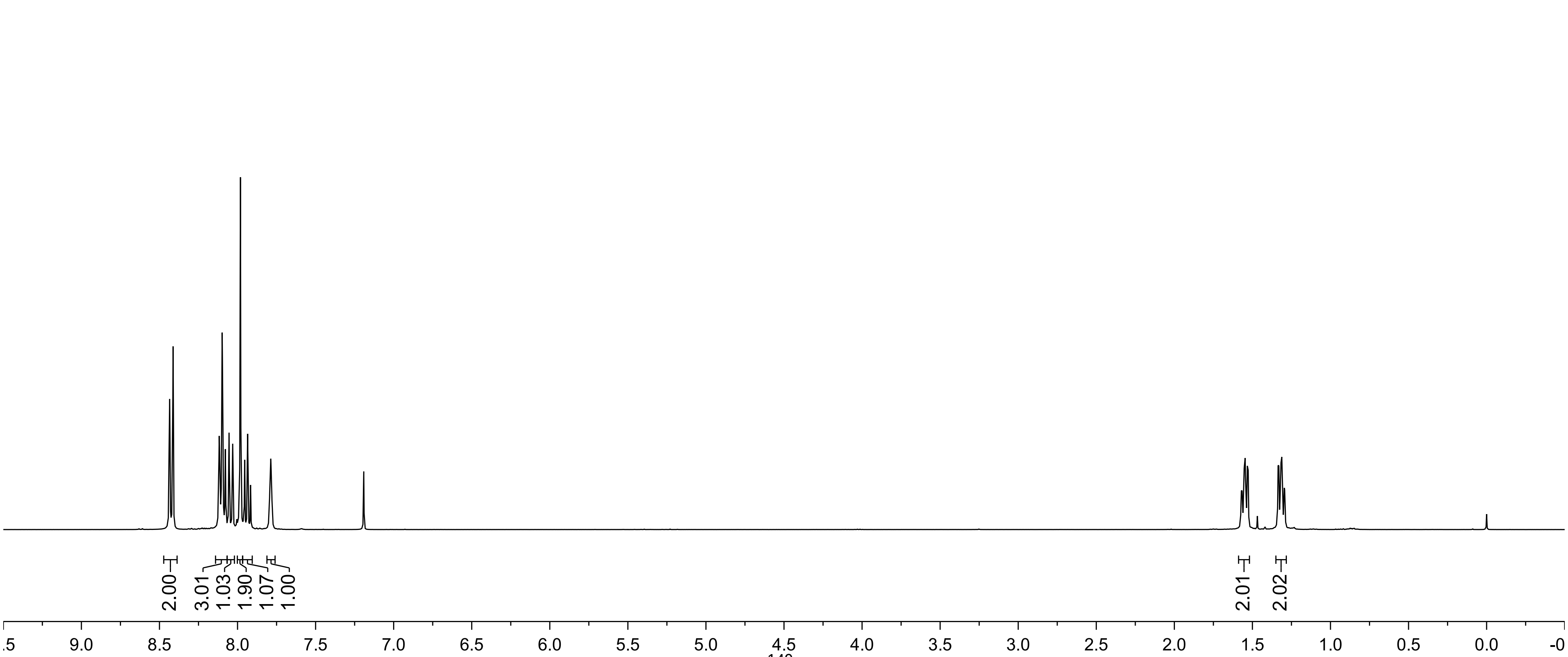

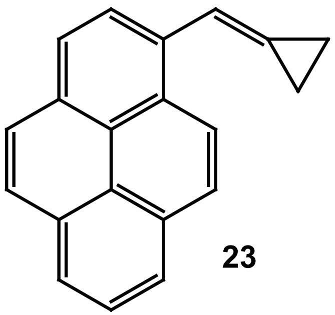

23

^{1}H NMR spectrum (400 MHz, $CDCl_3$)

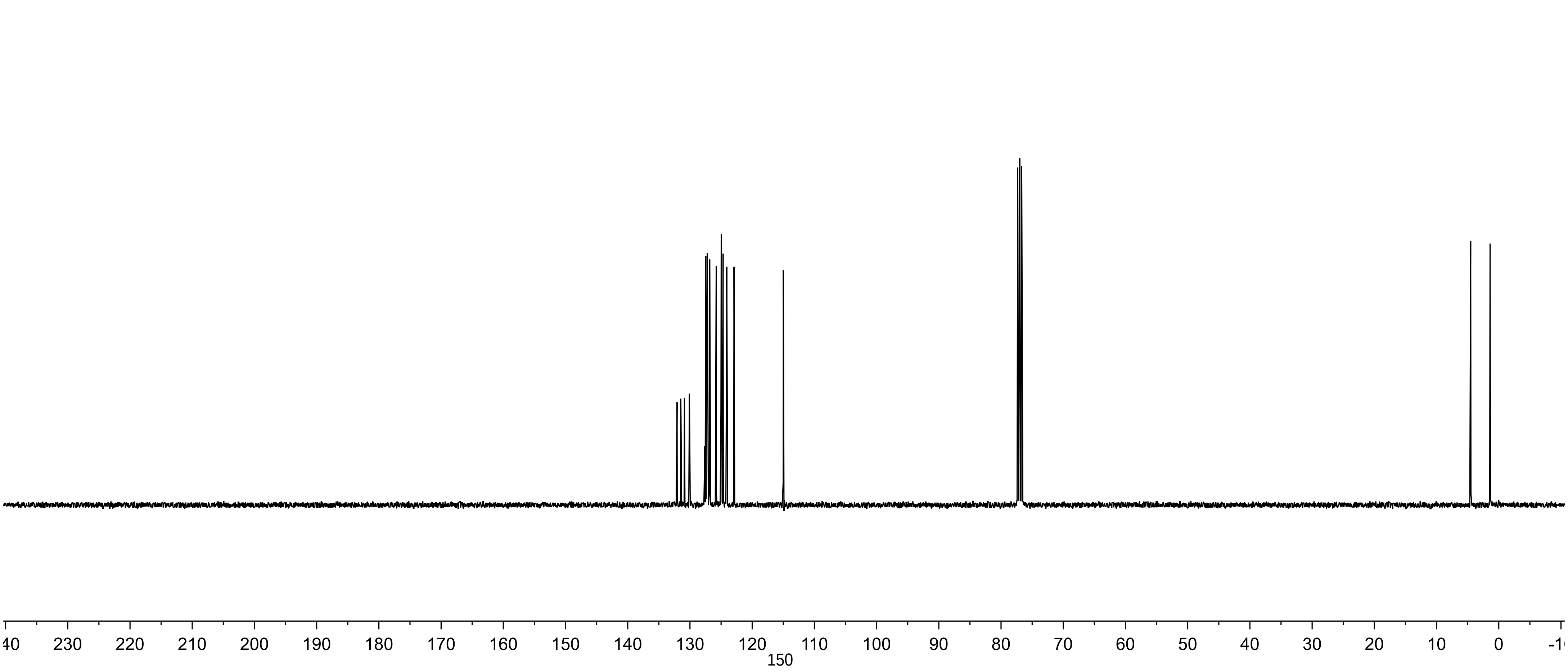

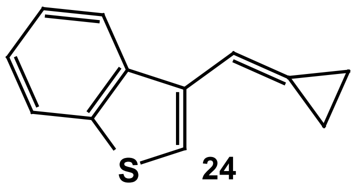

24

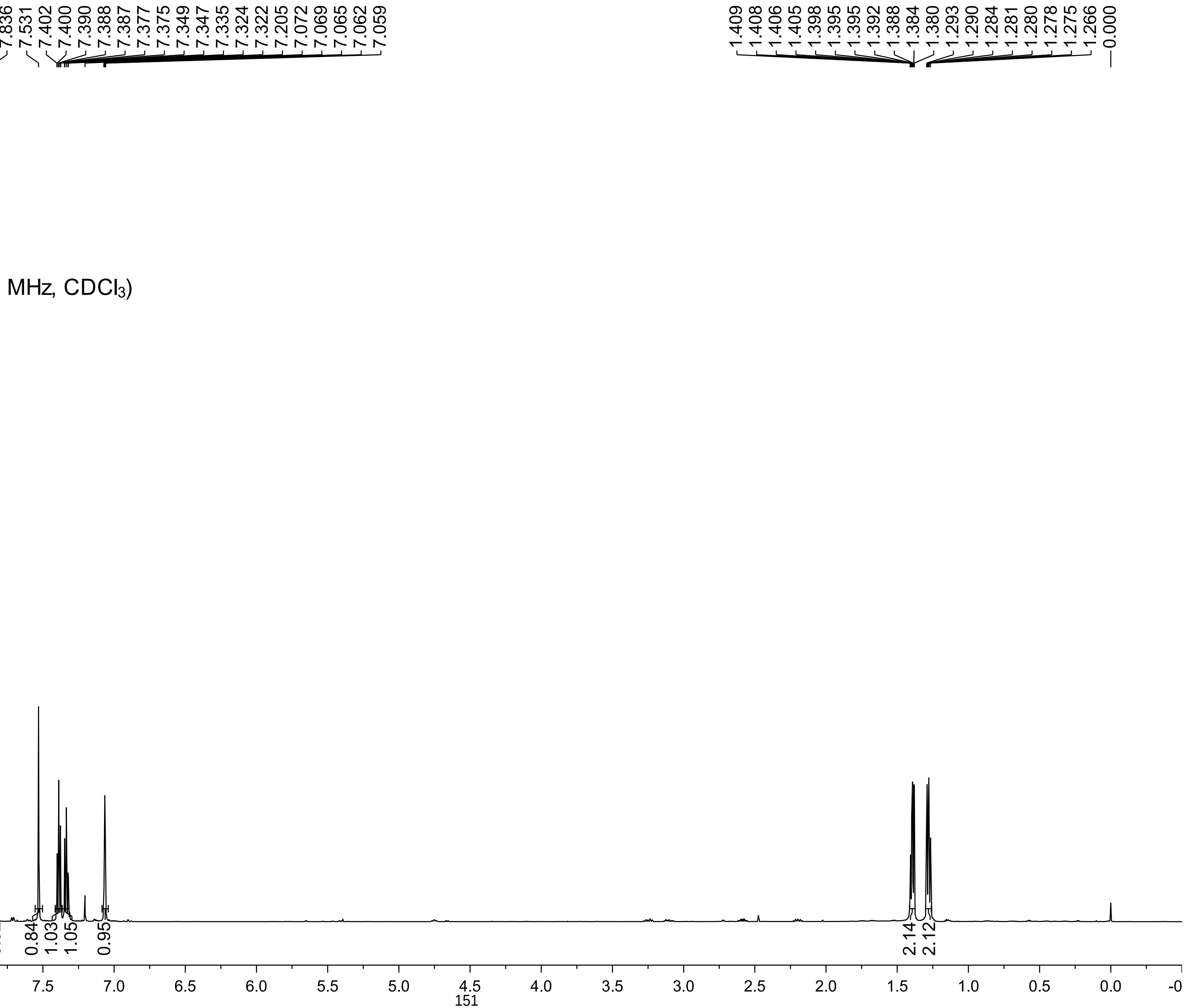

^{1}H NMR spectrum (600 MHz, $CDCl_3$)

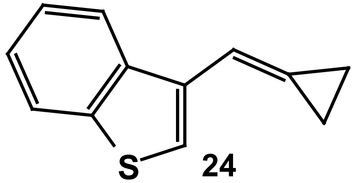

24

^{13}**C NMR** spectrum (150 MHz, $CDCl_3$)

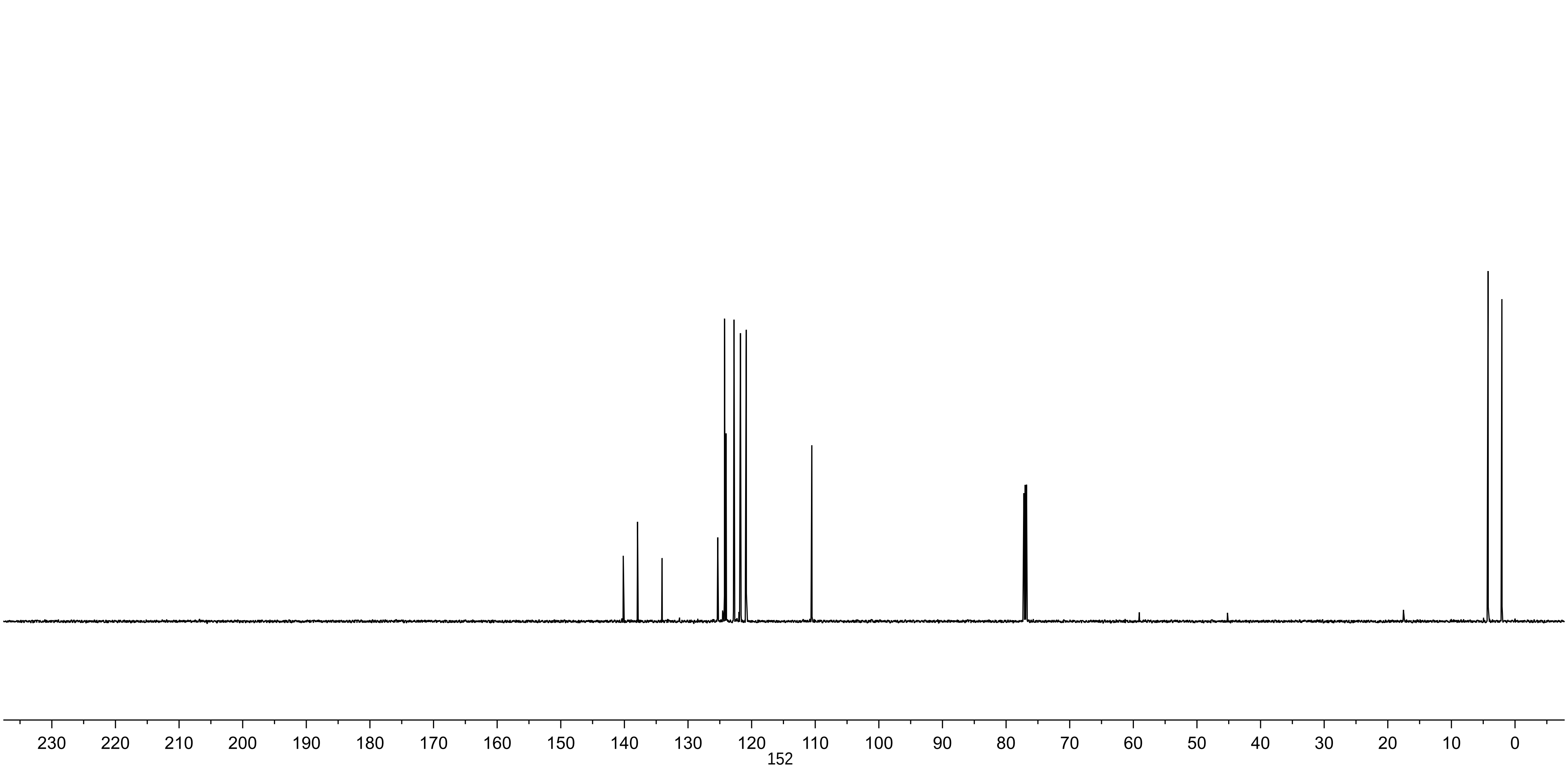

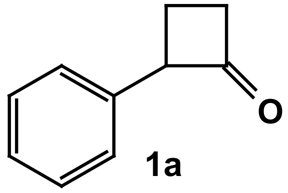

1a

[1]**H NMR** spectrum (400 MHz, $CDCl_3$)

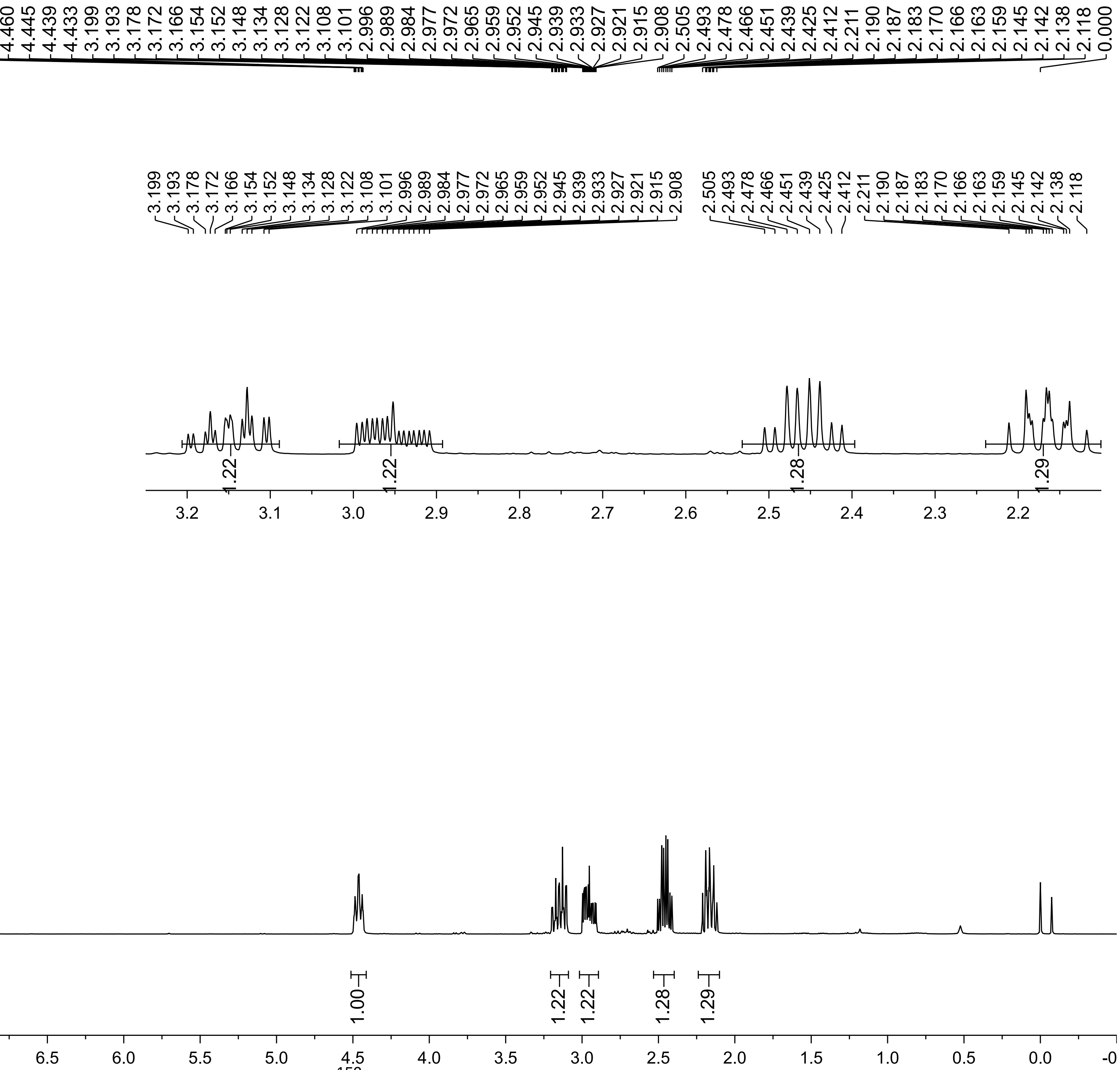

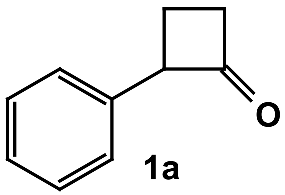

1a

^{13}**C NMR** spectrum (100 MHz, $CDCl_3$)

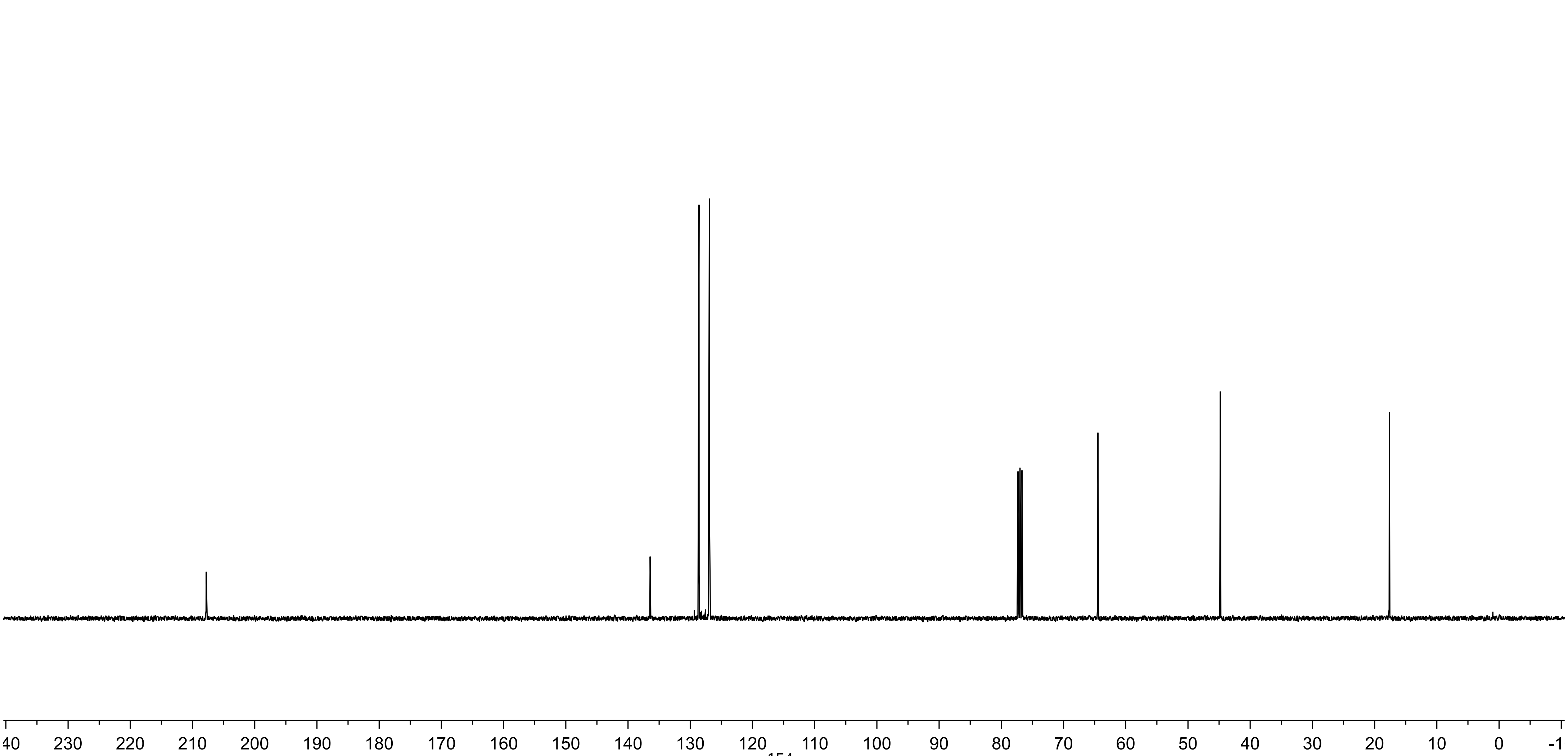

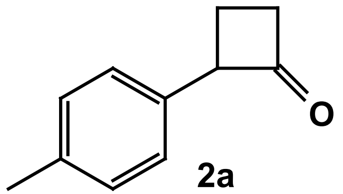

2a

[1]**H NMR** spectrum (400 MHz, $CDCl_3$)

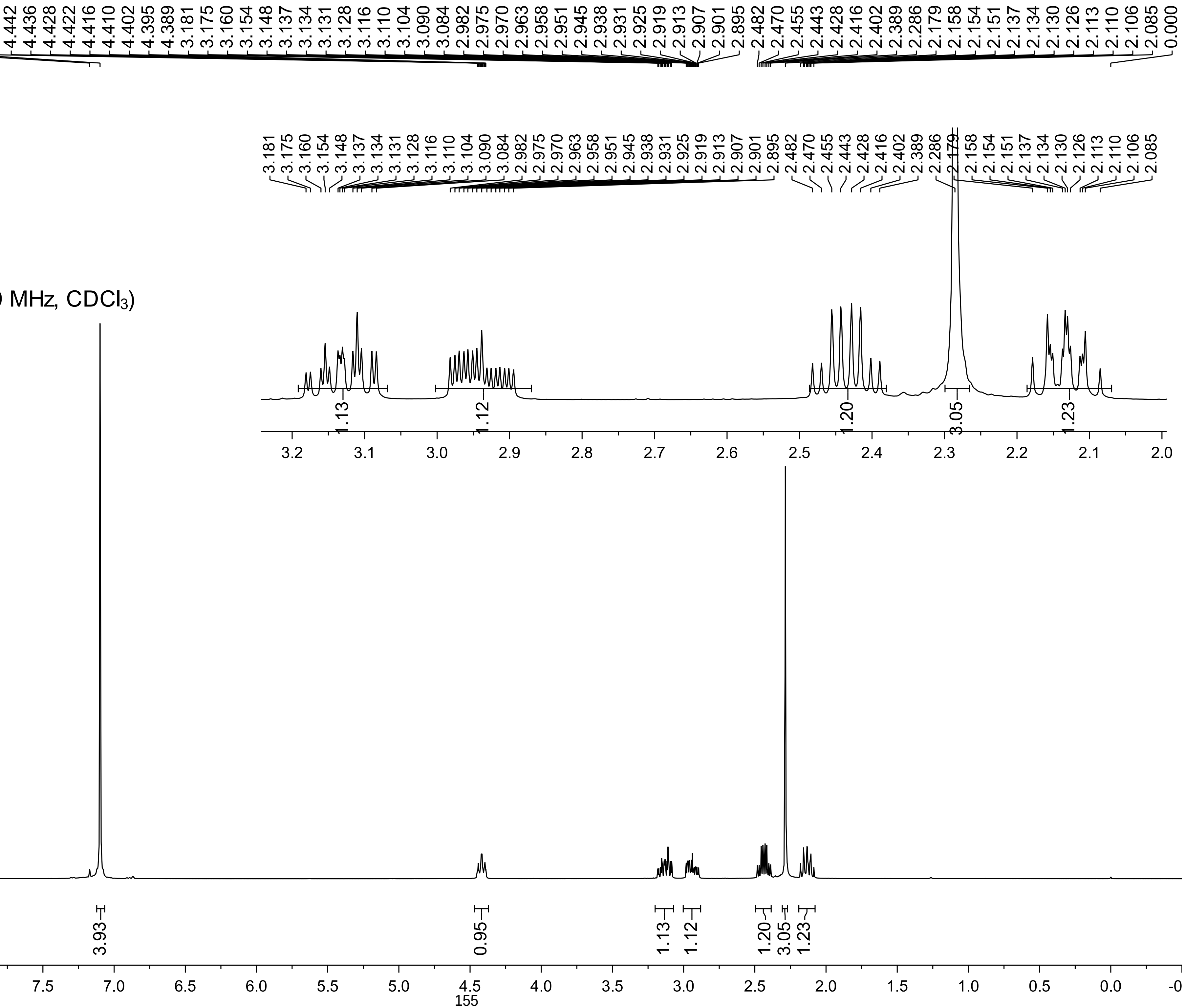

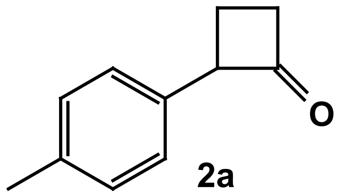

2a

^{13}C NMR spectrum (100 MHz, $CDCl_3$)

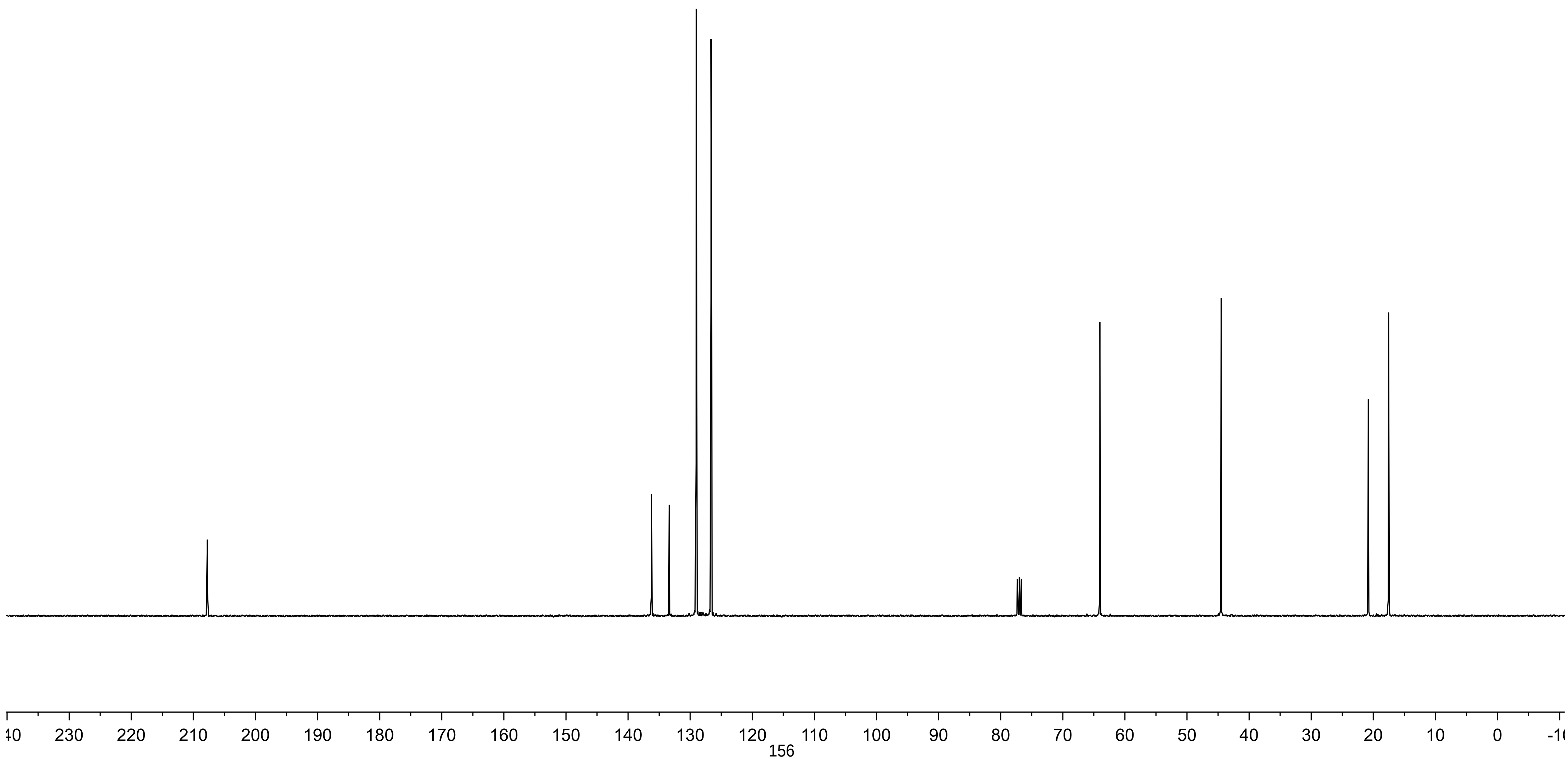

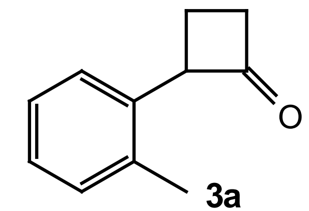

3a

[1]**H NMR** spectrum (400 MHz, $CDCl_3$)

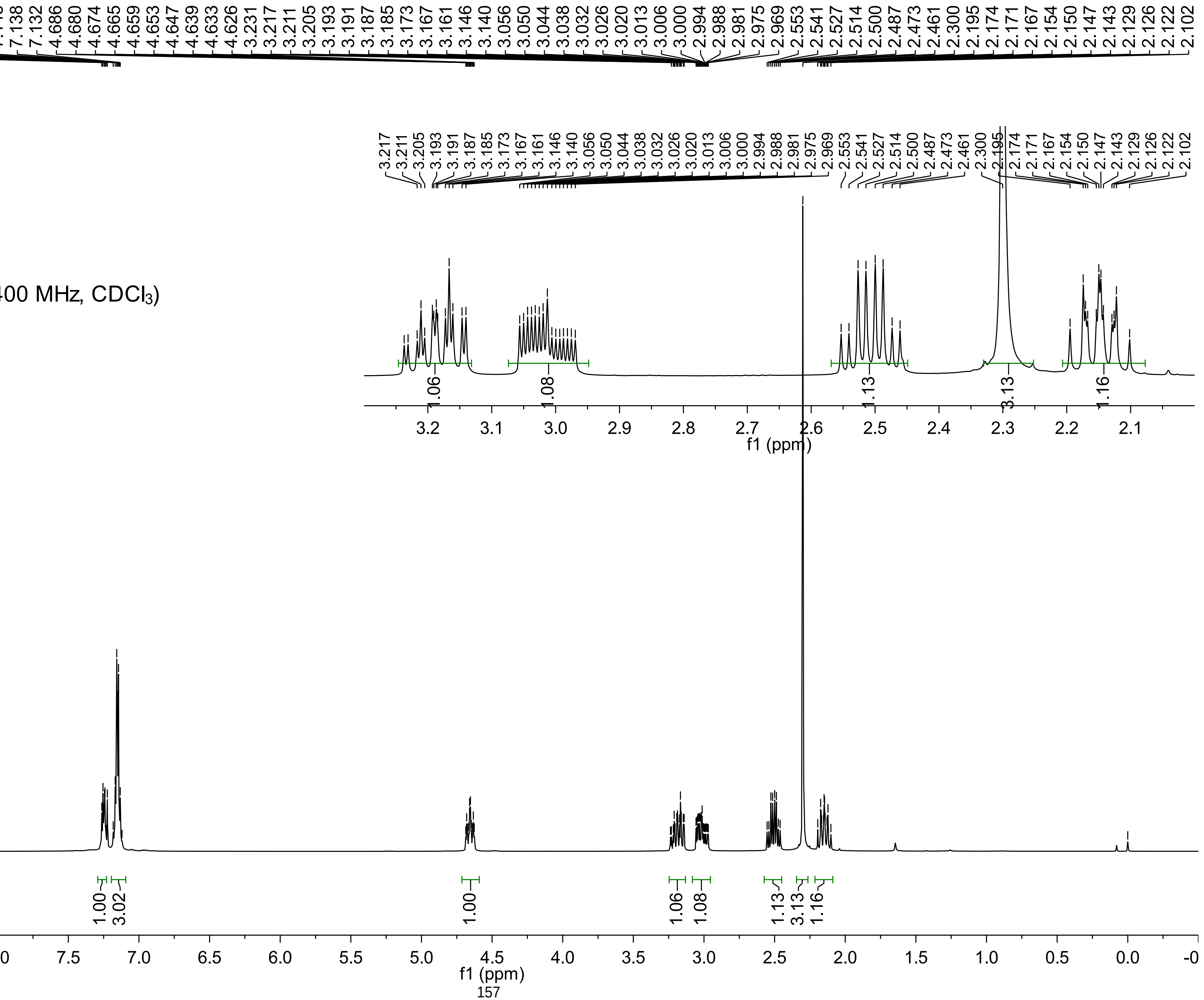

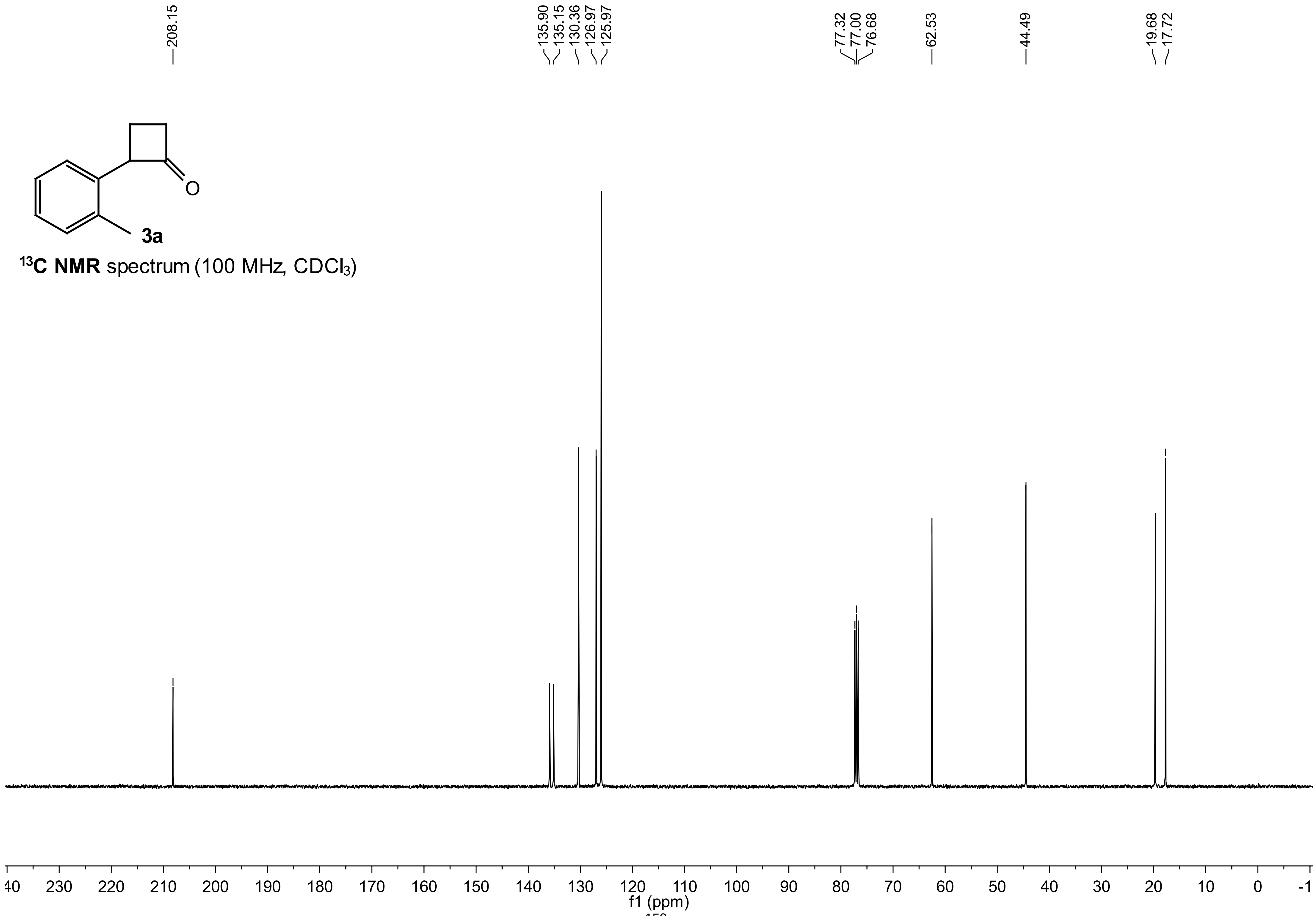
208.15
135.90
135.15
130.36
126.97
125.97
77.32
77.00
76.68
62.53
44.49
19.68
17.72
O
3a
13C NMR spectrum (100 MHz, CDCl3)
40
230
220
210
200
190
180
170
160
150
140
130
120
110
100
90
80
70
60
50
40
30
20
10
0
-1
f1 (ppm)

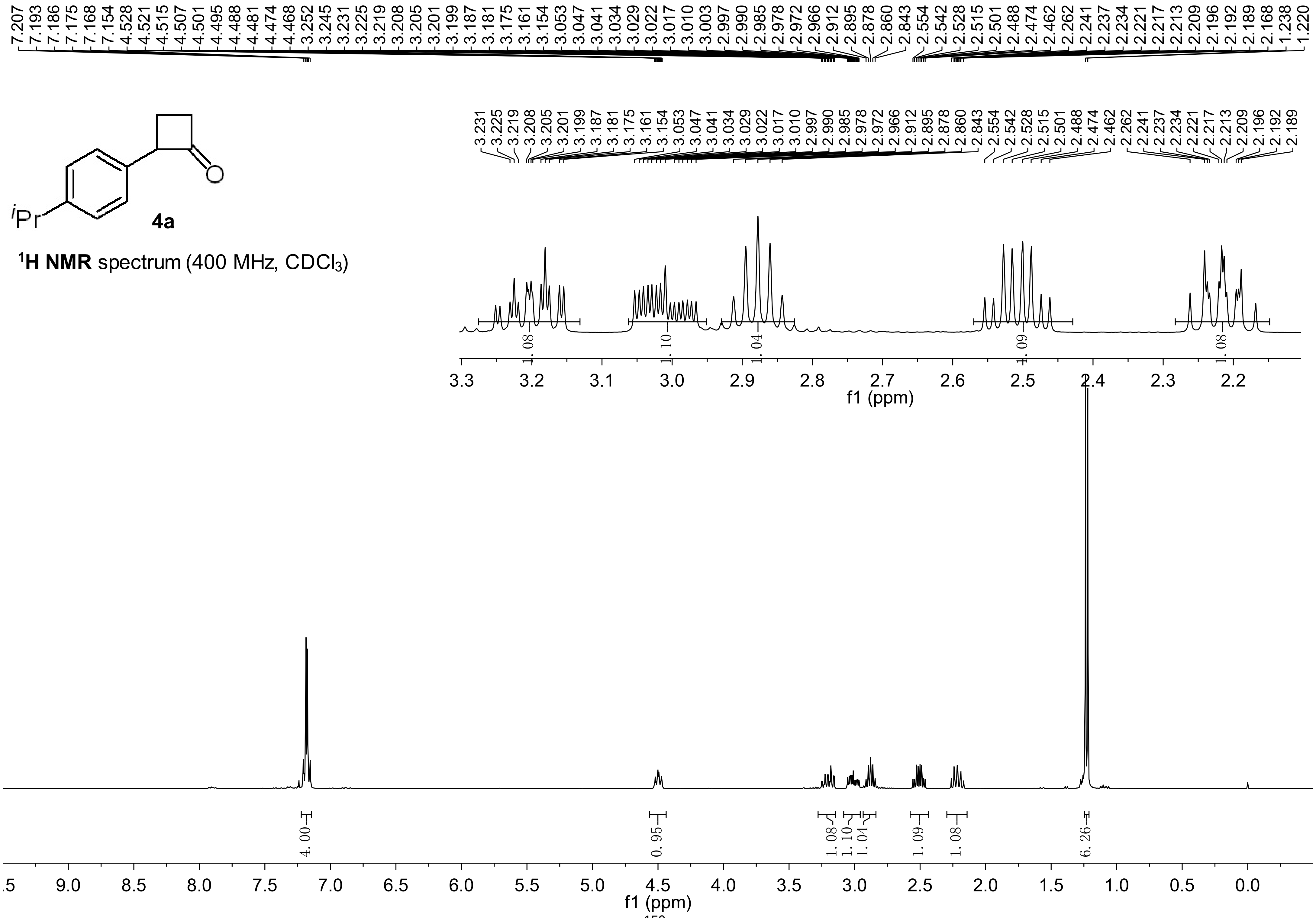
7.207 7.193 7.186 7.175 7.168 7.154 4.528 4.521 4.515 4.507 4.501 4.495 4.488 4.481 4.474 4.468 3.252 3.245 3.231 3.225 3.219 3.208 3.205 3.201 3.199 3.187 3.181 3.175 3.161 3.154 3.053 3.047 3.041 3.034 3.029 3.022 3.017 3.010 3.003 2.997 2.990 2.985 2.978 2.972 2.966 2.912 2.895 2.878 2.860 2.843 2.554 2.542 2.528 2.515 2.501 2.488 2.474 2.462 2.262 2.241 2.237 2.234 2.221 2.217 2.213 2.209 2.196 2.192 2.189 2.168 1.238 1.220
iPr
4a
1H NMR spectrum (400 MHz, CDCl3)
1.08
1.10
1.04
1.09
1.08
3.3 3.2 3.1 3.0 2.9 2.8 2.7 2.6 2.5 2.4 2.3 2.2
f1 (ppm)
4.00
0.95
1.08
1.10
1.04
1.09
1.08
6.26
.5 9.0 8.5 8.0 7.5 7.0 6.5 6.0 5.5 5.0 4.5 4.0 3.5 3.0 2.5 2.0 1.5 1.0 0.5 0.0
f1 (ppm)

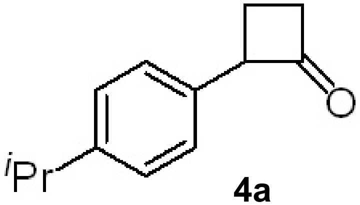

4a

^{13}C NMR spectrum (100 MHz, $CDCl_3$)

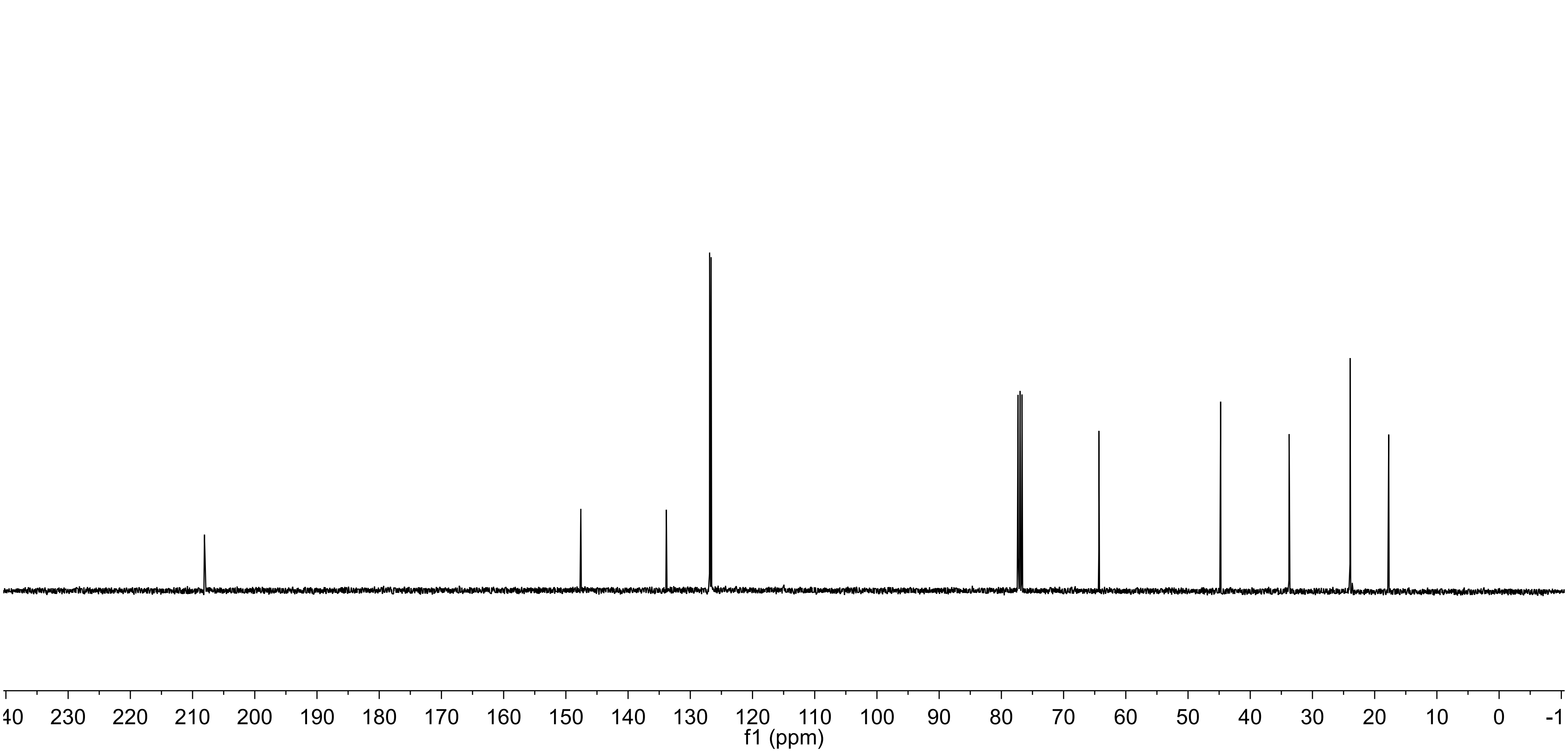

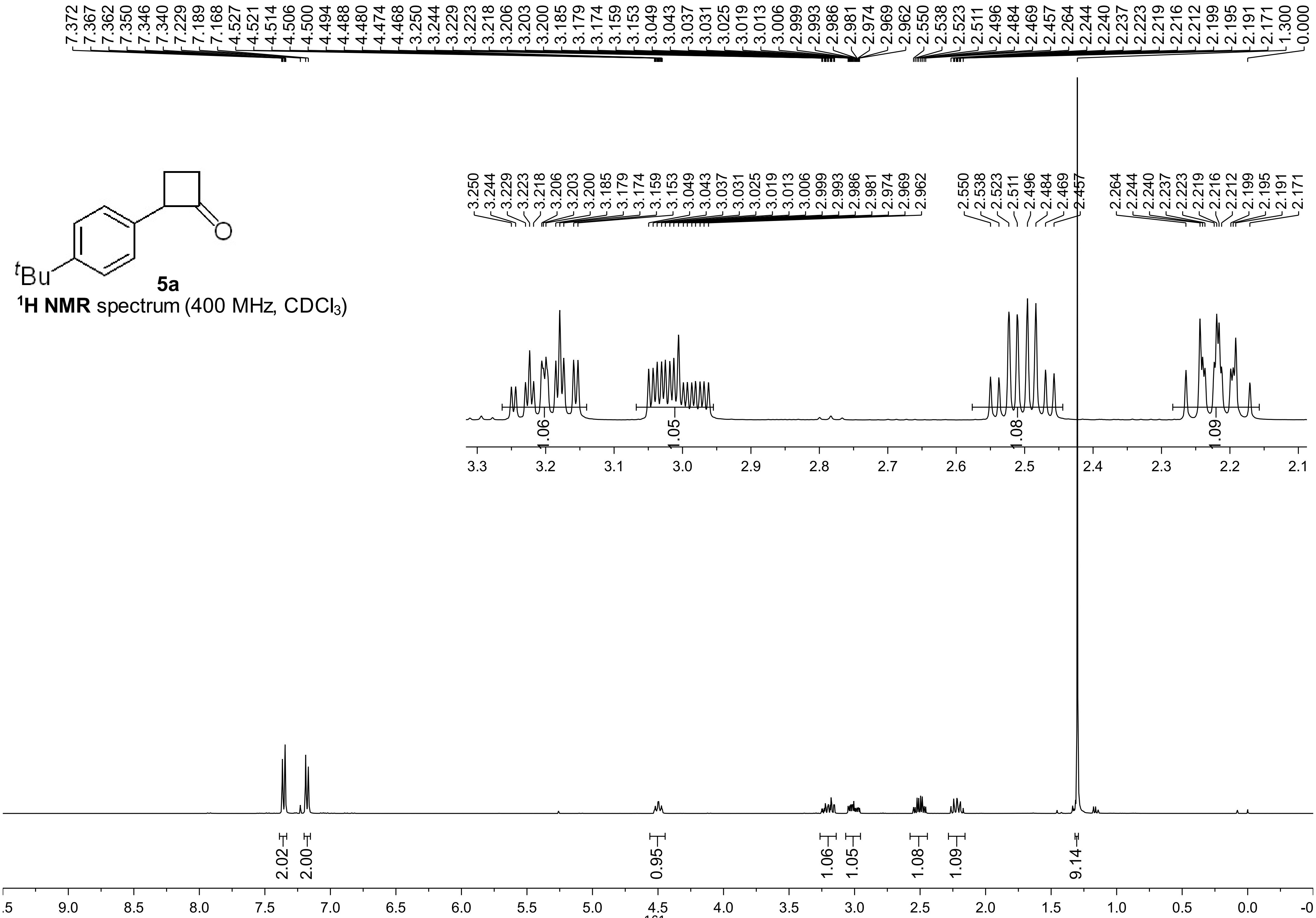

^{t}Bu
O
5a
^{1}H NMR spectrum (400 MHz, $CDCl_3$)
7.372 7.367 7.362 7.350 7.346 7.340 7.229 7.189 7.168 4.527 4.521 4.514 4.506 4.500 4.494 4.488 4.480 4.474 4.468 3.250 3.244 3.229 3.223 3.218 3.206 3.203 3.200 3.185 3.179 3.174 3.159 3.153 3.049 3.043 3.037 3.031 3.025 3.019 3.013 3.006 2.999 2.993 2.986 2.981 2.974 2.969 2.962 2.550 2.538 2.523 2.511 2.496 2.484 2.469 2.457 2.264 2.244 2.240 2.237 2.223 2.219 2.216 2.212 2.199 2.195 2.191 2.171 1.300 0.000
1.06
1.05
1.08
1.09
3.3 3.2 3.1 3.0 2.9 2.8 2.7 2.6 2.5 2.4 2.3 2.2 2.1
2.02
2.00
0.95
1.06
1.05
1.08
1.09
9.14
.5 9.0 8.5 8.0 7.5 7.0 6.5 6.0 5.5 5.0 4.5 4.0 3.5 3.0 2.5 2.0 1.5 1.0 0.5 0.0 -0

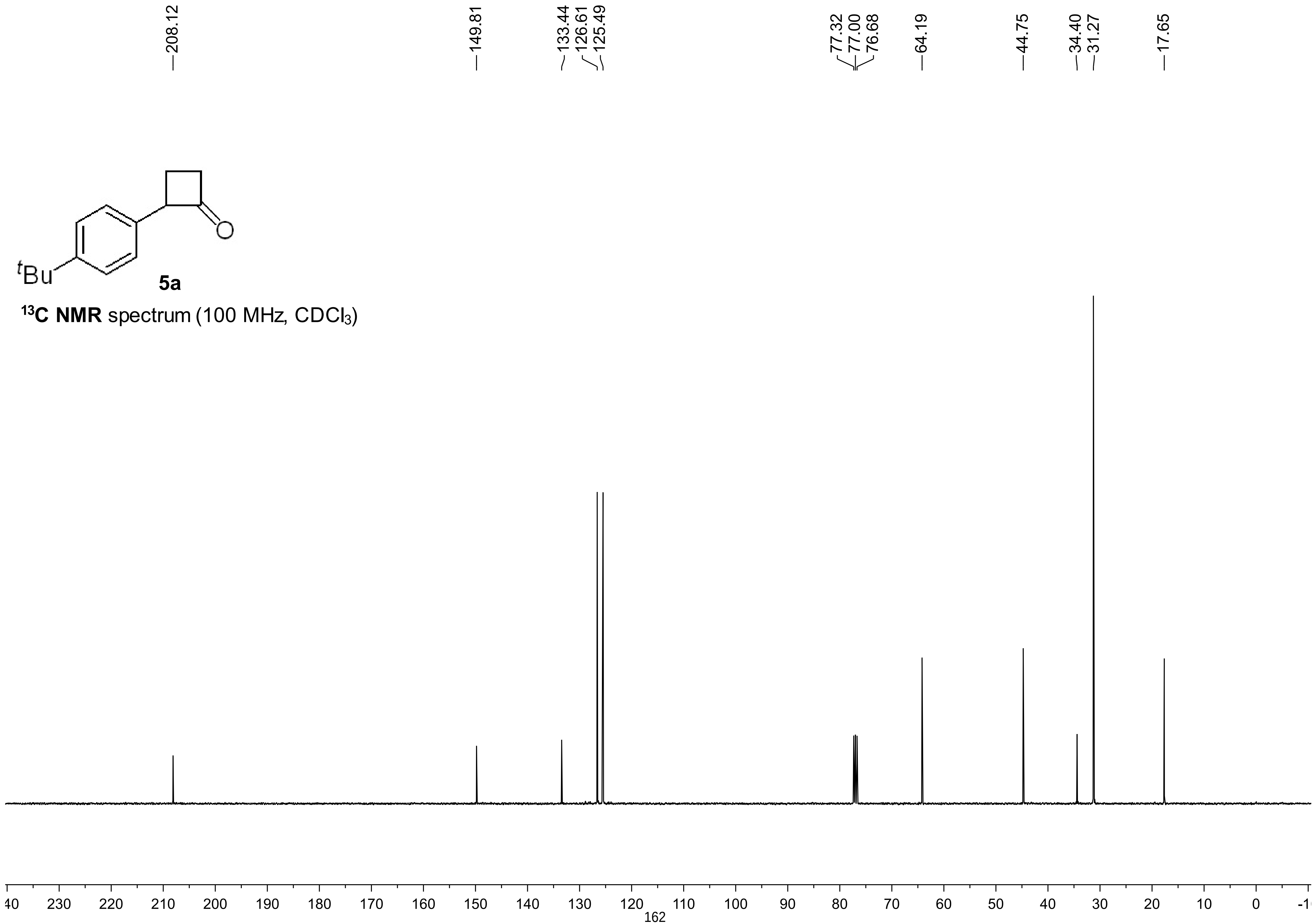
208.12
149.81
133.44
126.61
125.49
77.32
77.00
76.68
64.19
44.75
34.40
31.27
17.65
^{t}Bu
5a
^{13}C NMR spectrum (100 MHz, $CDCl_3$)
40 230 220 210 200 190 180 170 160 150 140 130 120 110 100 90 80 70 60 50 40 30 20 10 0

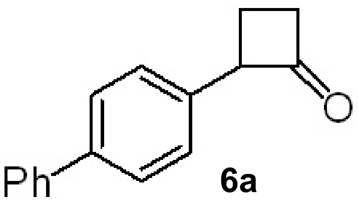

6a

^{1}H NMR spectrum (400 MHz, $CDCl_3$)

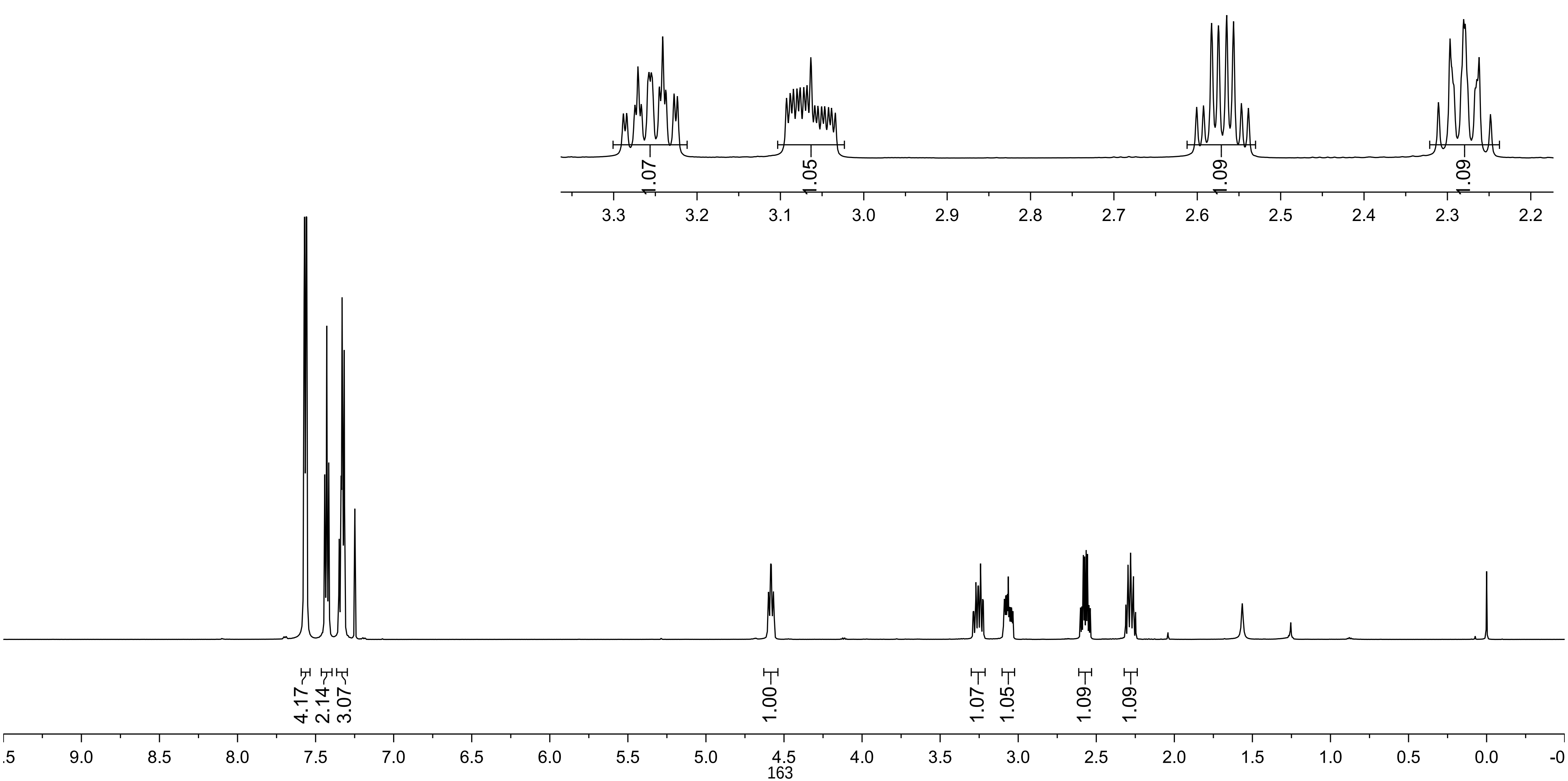

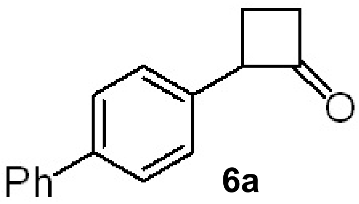

^{13}C NMR spectrum (100 MHz, $CDCl_3$)

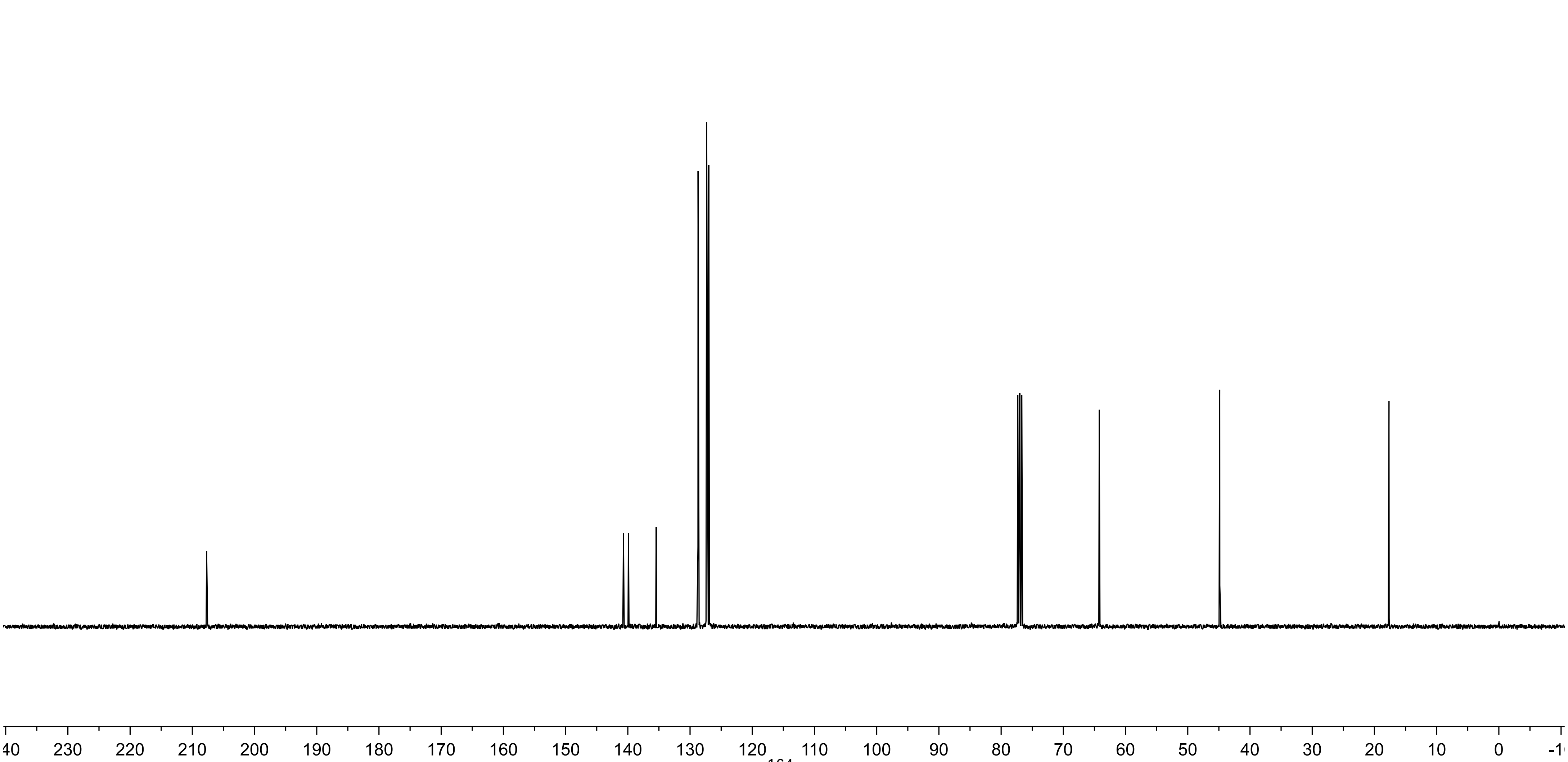

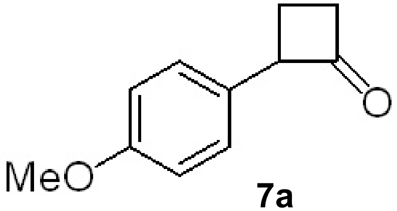

7a

^{1}H NMR spectrum (600 MHz, $CDCl_3$)

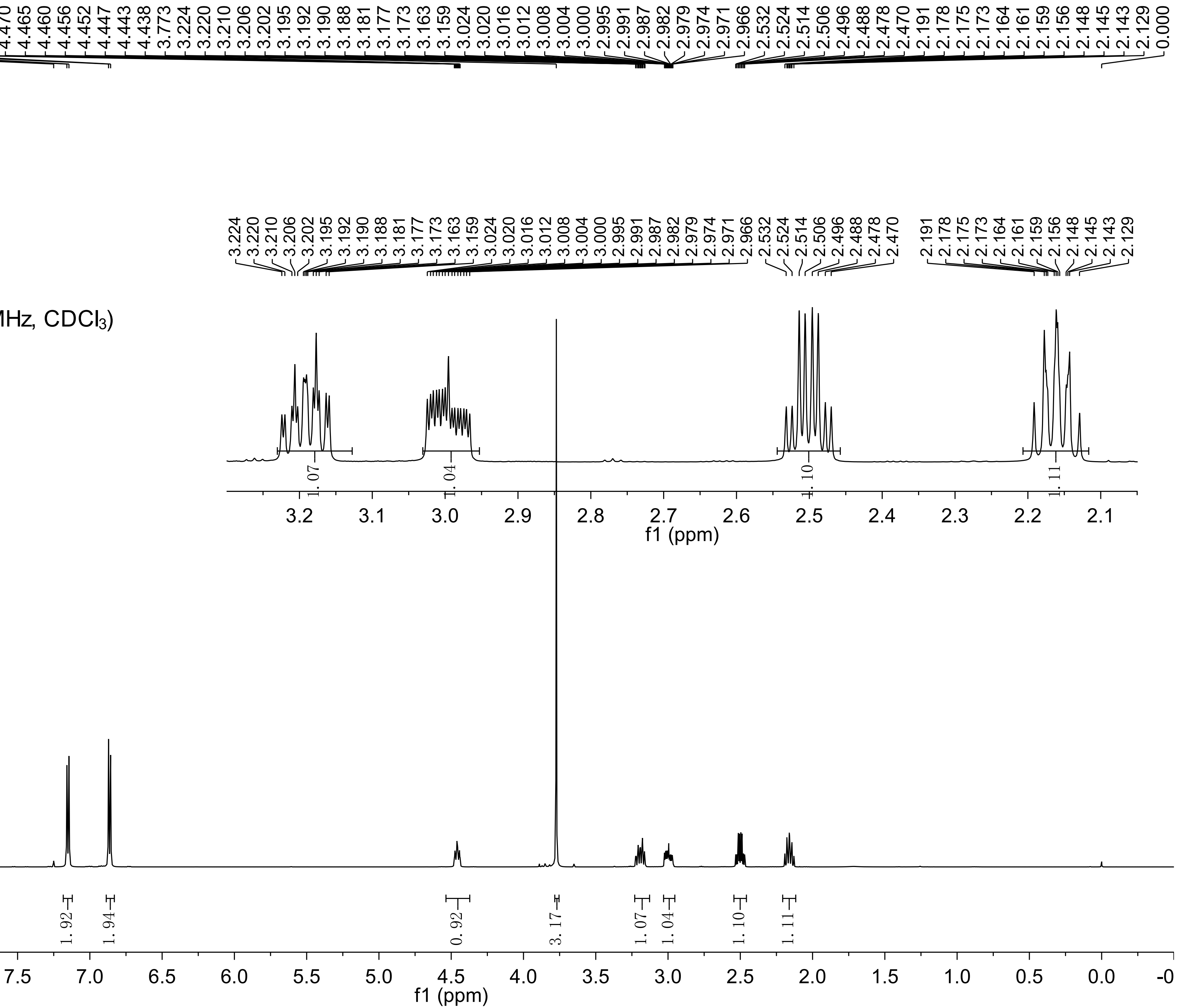

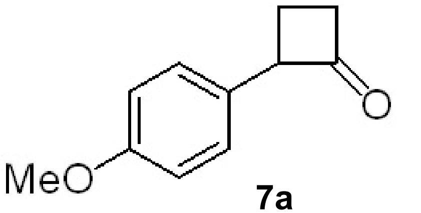

7a

^{13}C NMR spectrum (150 MHz, $CDCl_3$)

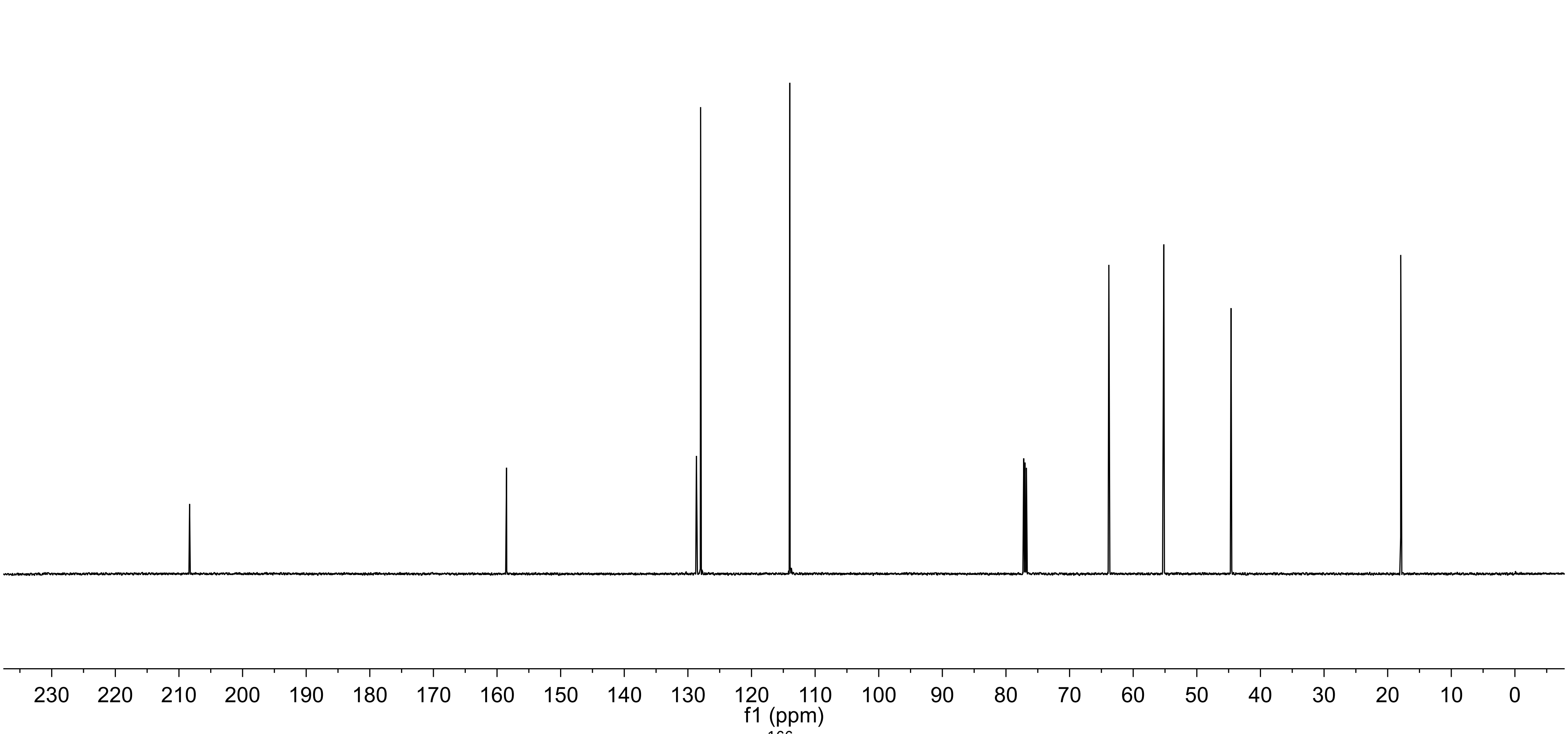

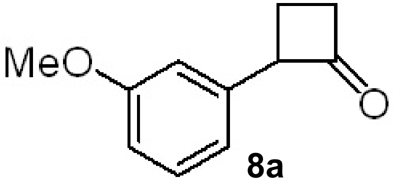

8a

^{1}H NMR spectrum (400 MHz, $CDCl_3$)

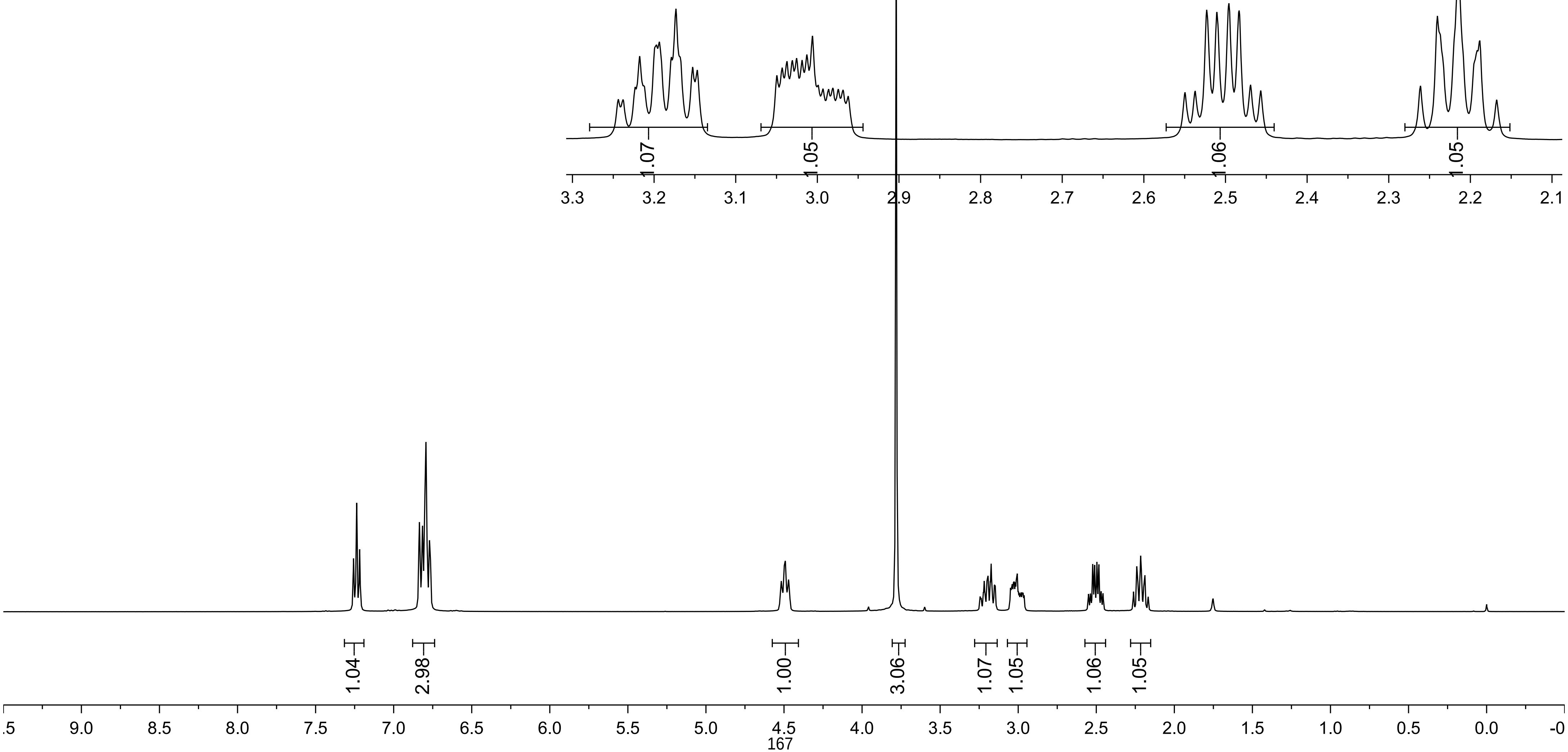

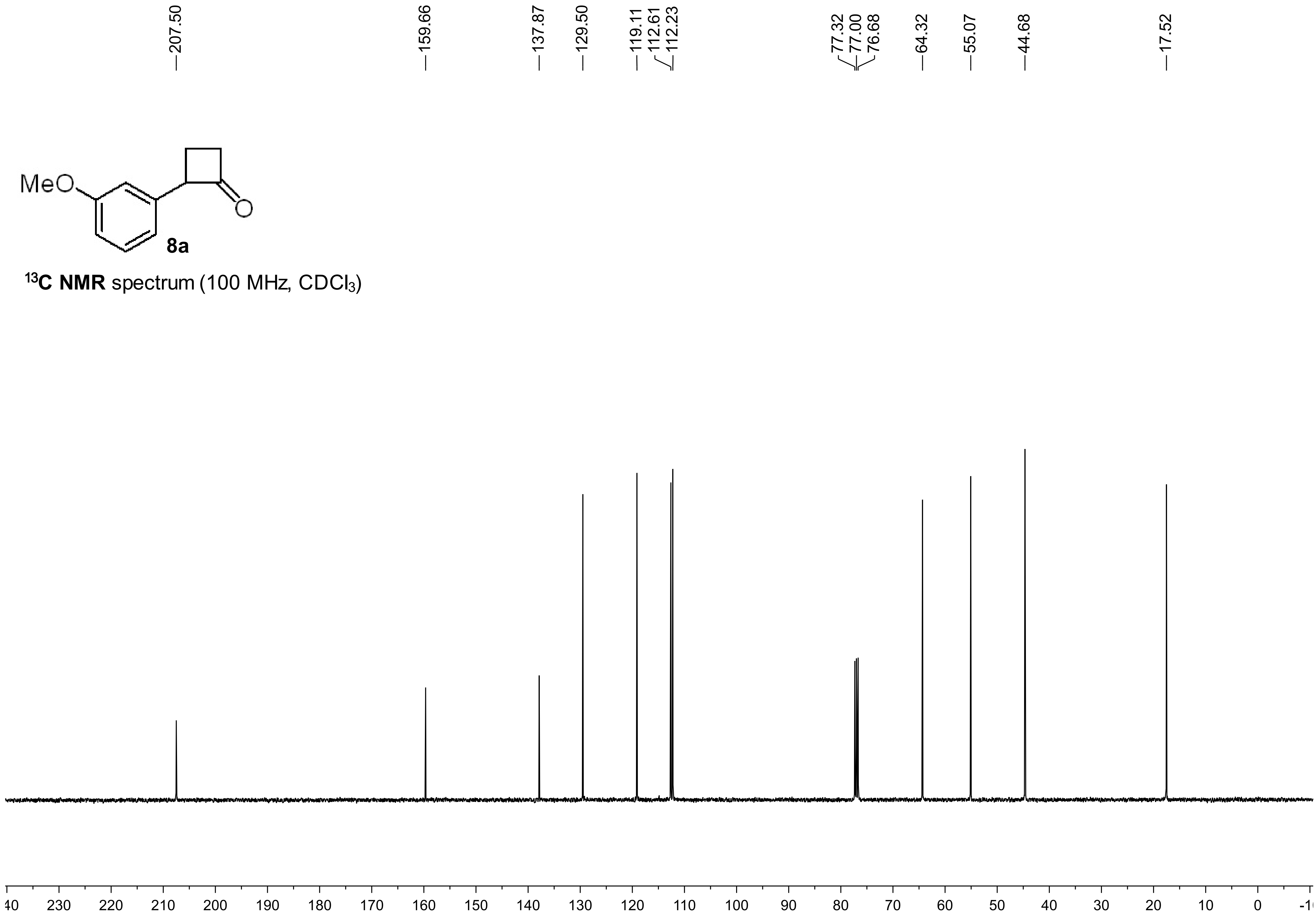
207.50
159.66
137.87
129.50
119.11
112.61
112.23
77.32
77.00
76.68
64.32
55.07
44.68
17.52
MeO
O
8a
^{13}C NMR spectrum (100 MHz, $CDCl_3$)
40
230
220
210
200
190
180
170
160
150
140
130
120
110
100
90
80
70
60
50
40
30
20
10
0
-1

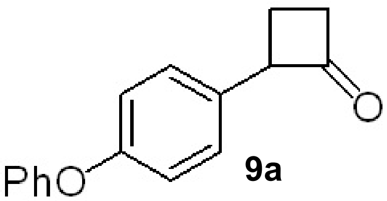

9a

^{1}H NMR spectrum (400 MHz, $CDCl_3$)

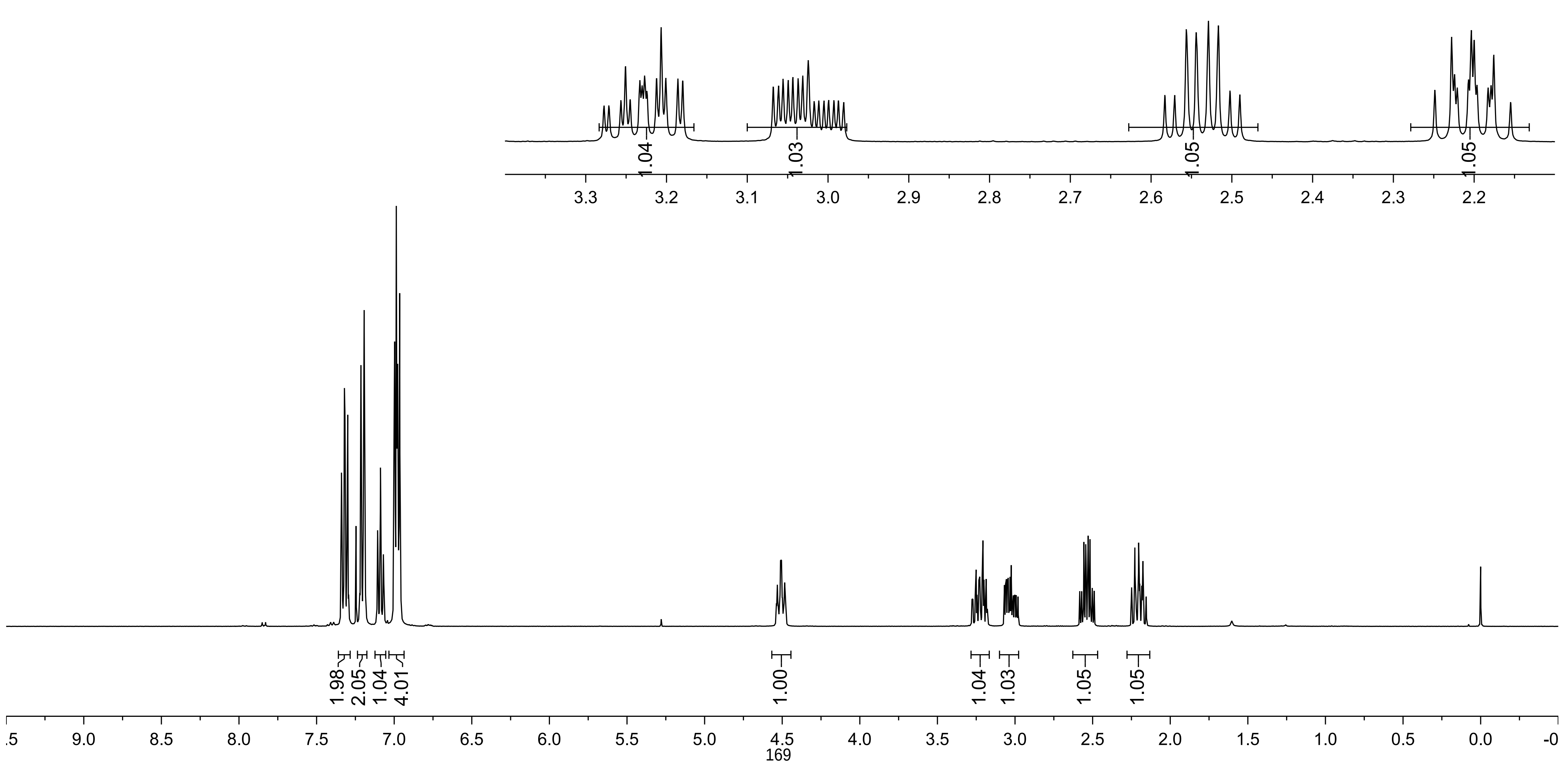

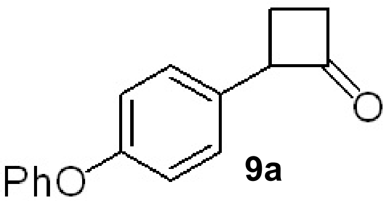

9a

^{13}C NMR spectrum (100 MHz, $CDCl_3$)

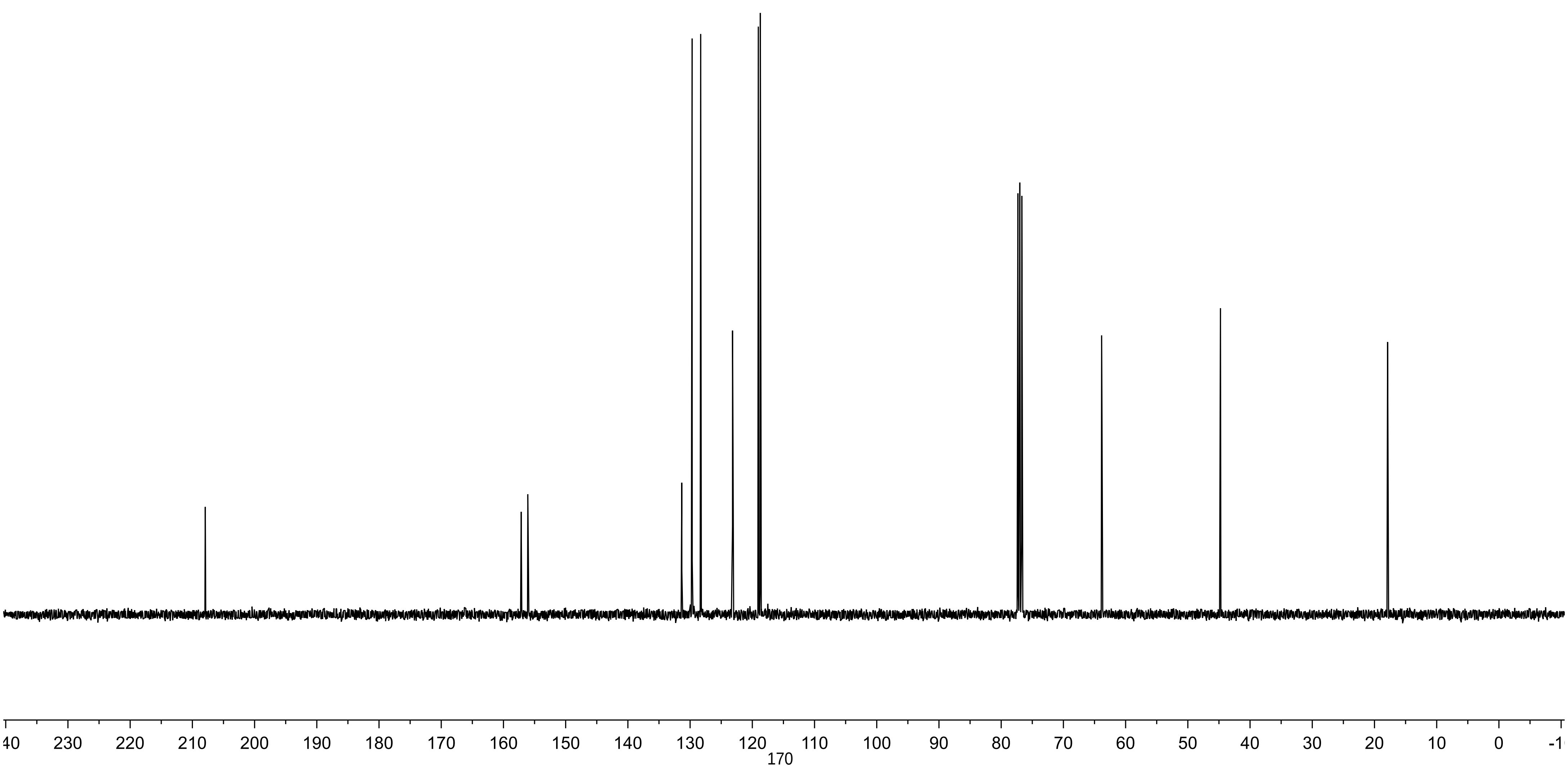

10a

[1]**H NMR** spectrum (400 MHz, $CDCl_3$)

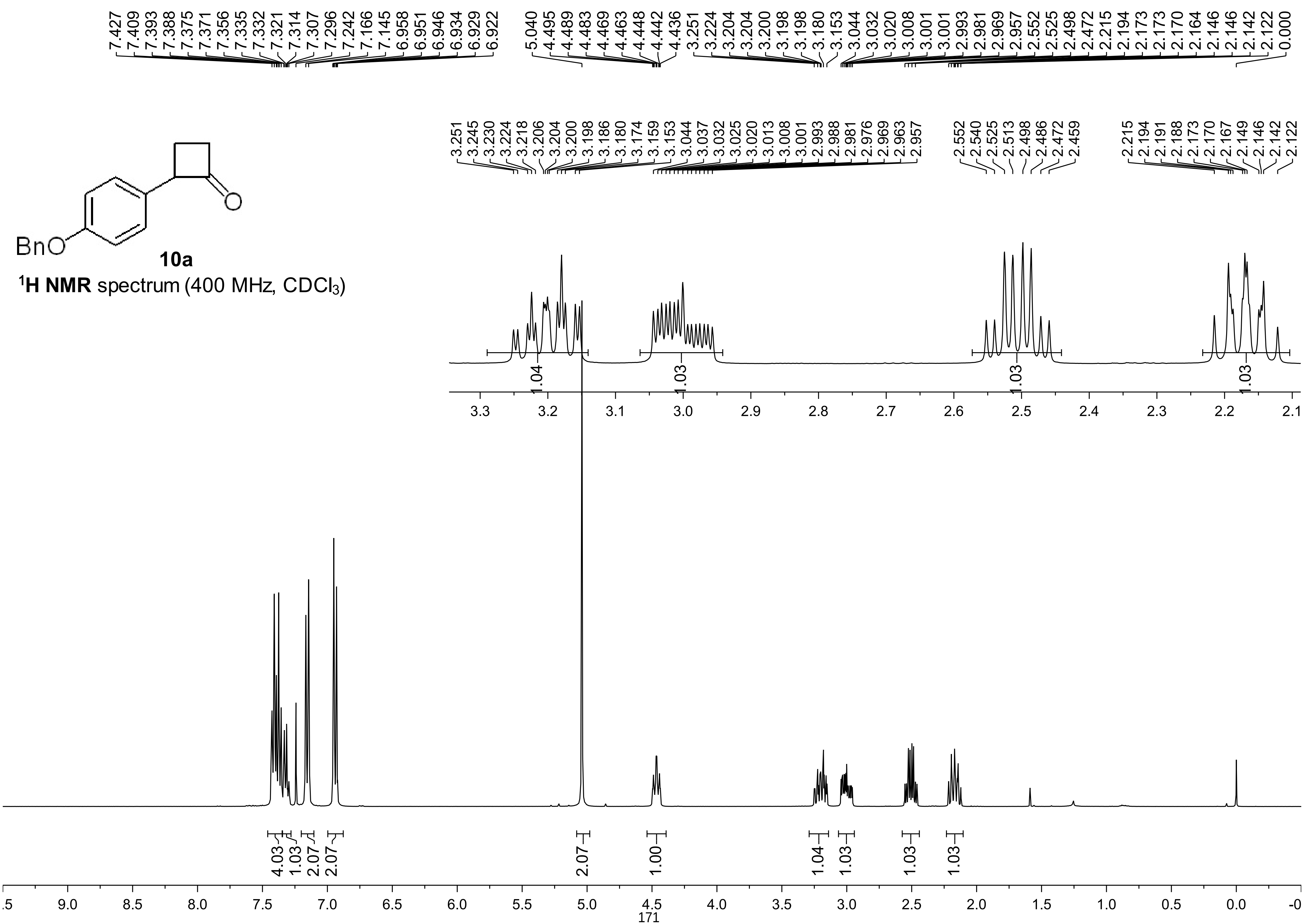

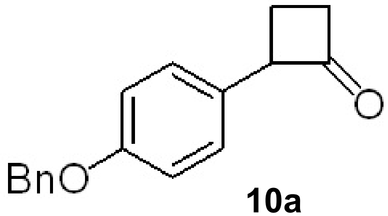

10a

[13]**C NMR** spectrum (100 MHz, $CDCl_3$)

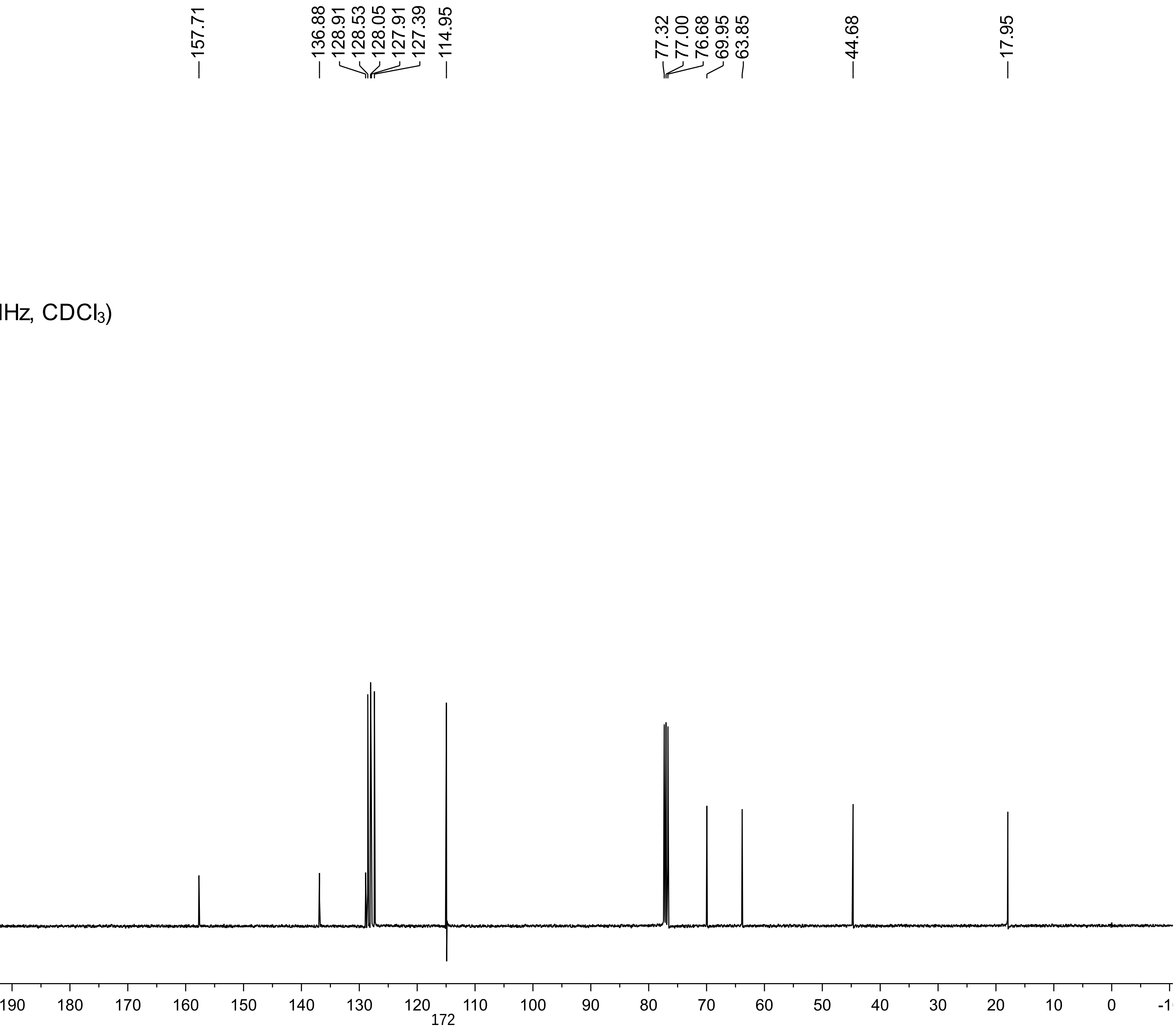

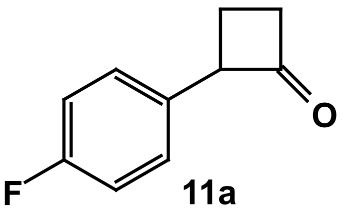

11a

[1]**H NMR** spectrum (400 MHz, $CDCl_3$)

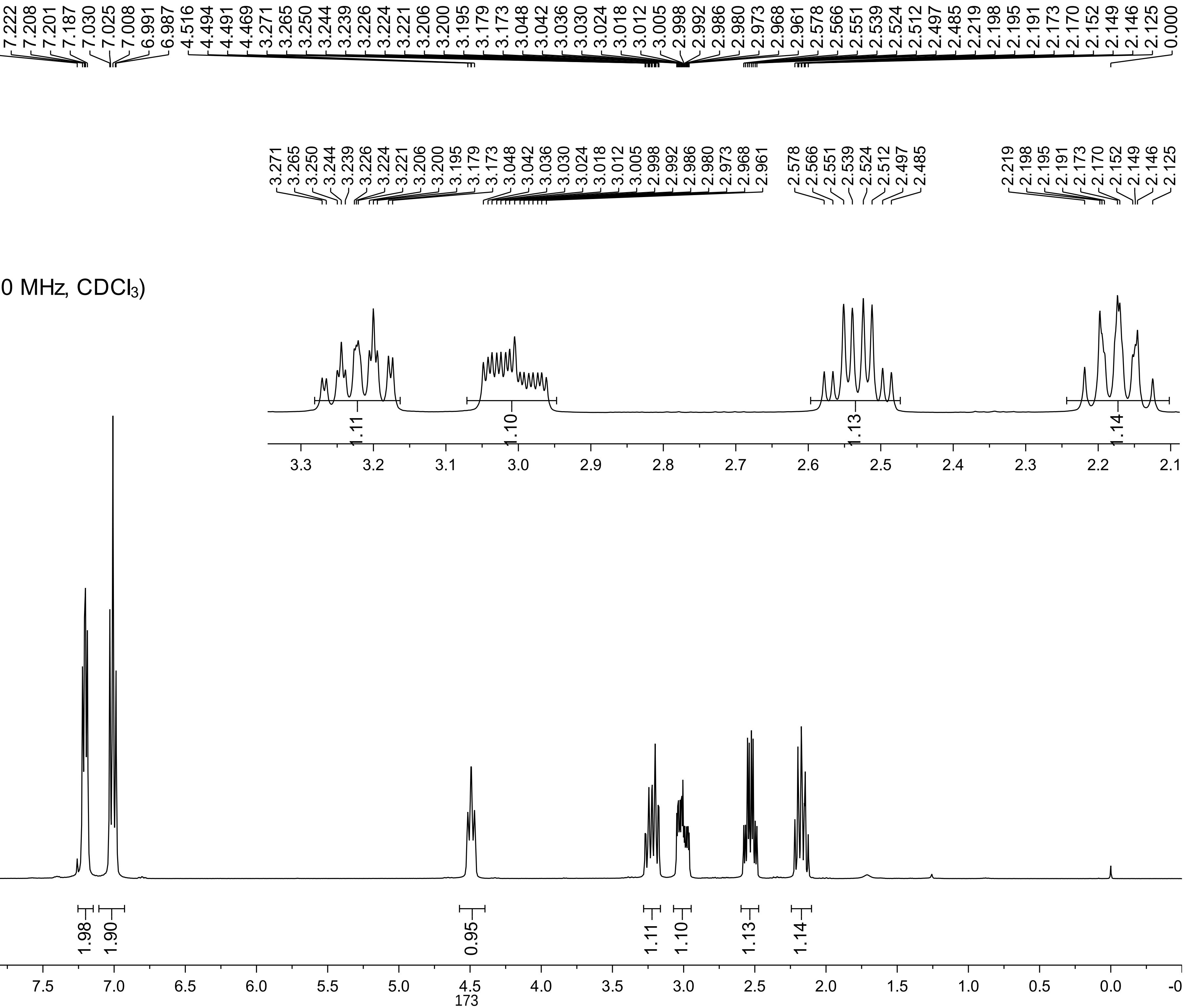

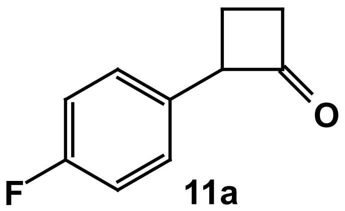

11a

^{13}C NMR spectrum (100 MHz, $CDCl_3$)

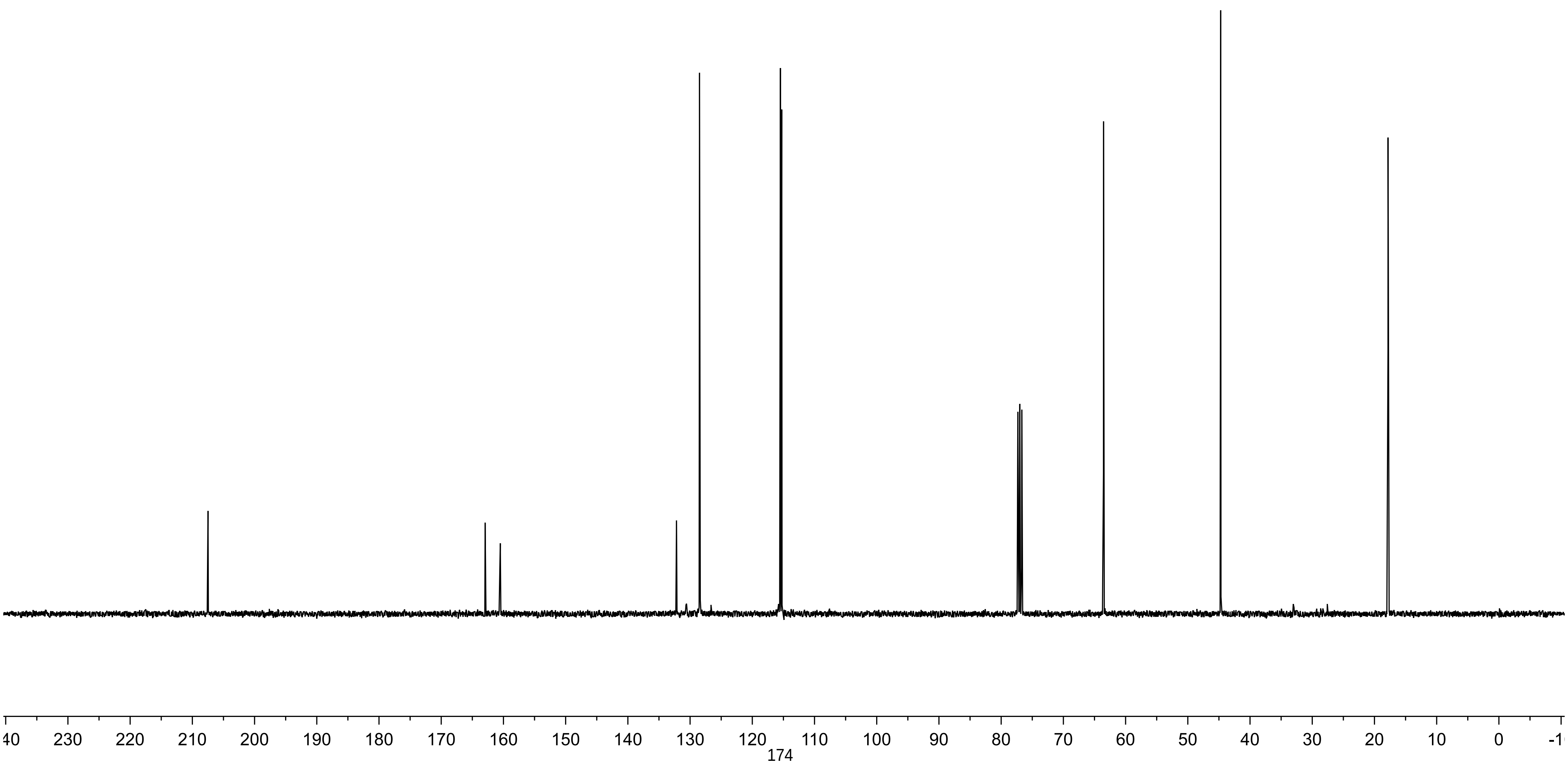

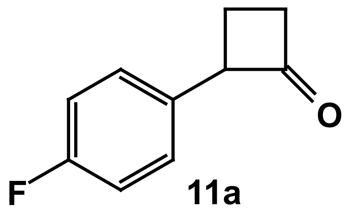

11a

^{19}**F NMR** spectrum (376 MHz, $CDCl_3$)

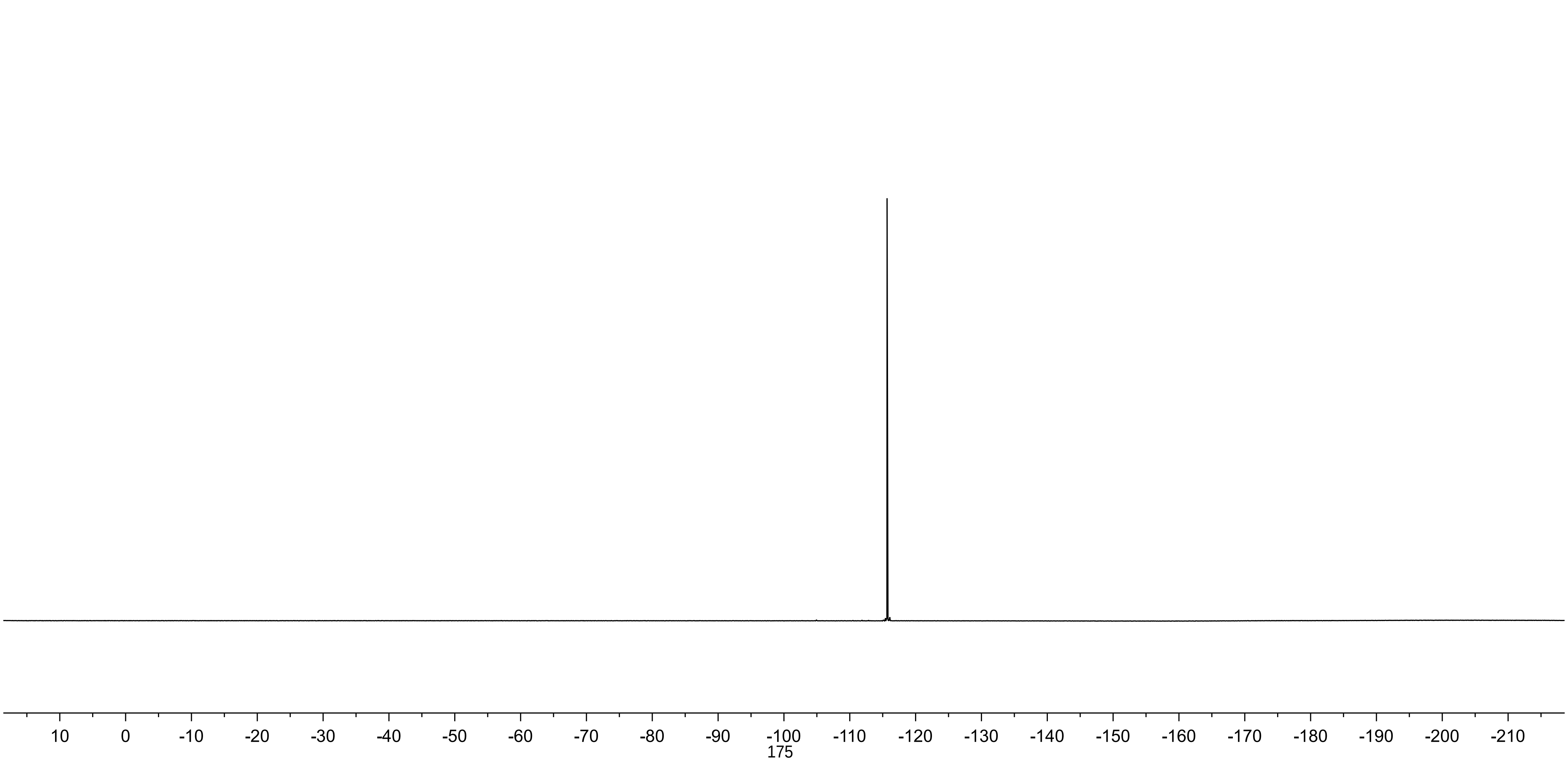

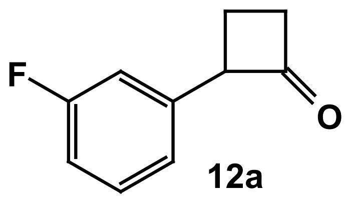

12a

^{1}H NMR spectrum (400 MHz, $CDCl_3$)

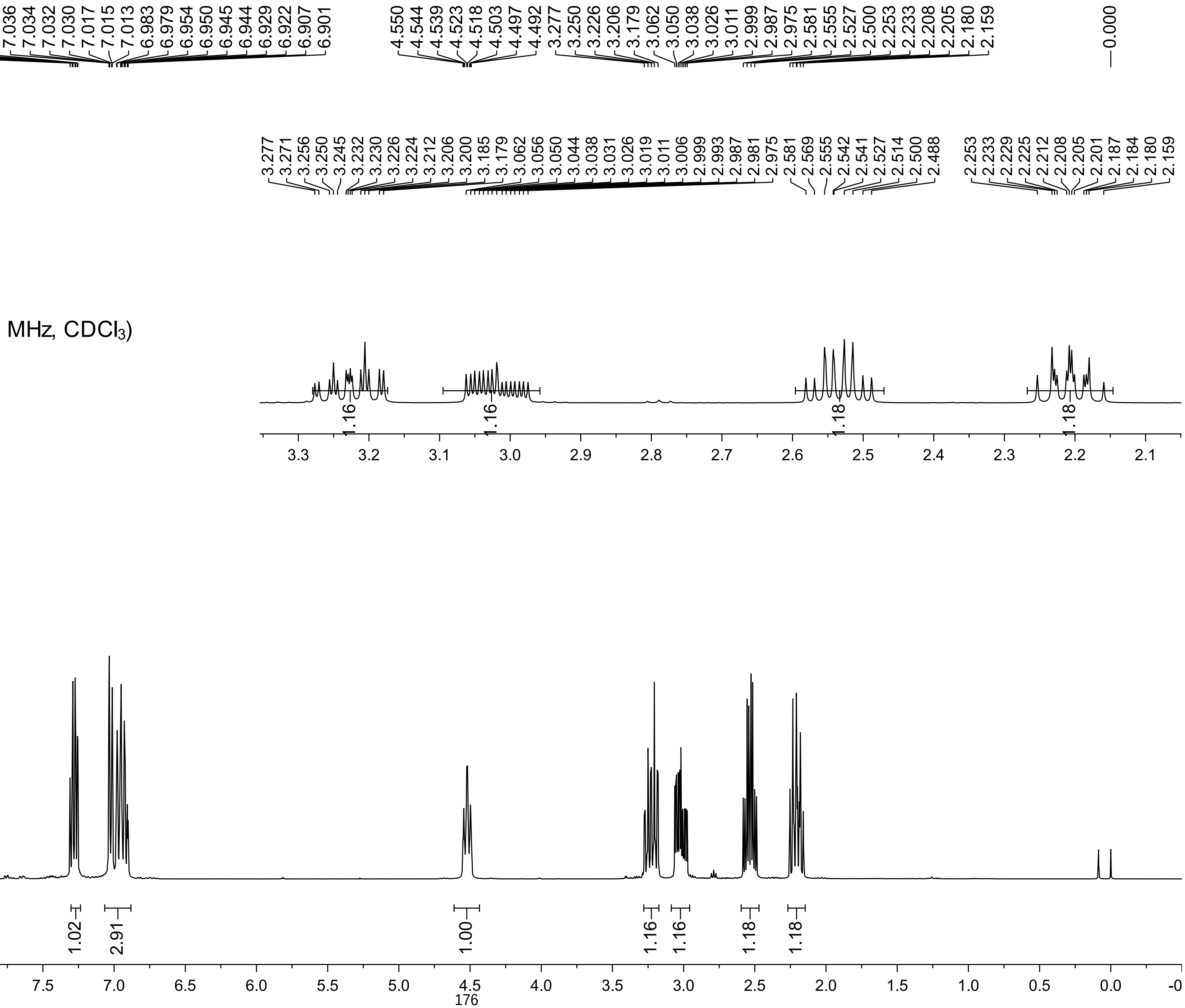

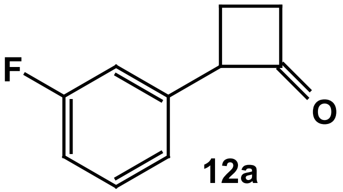

12a

^{13}C NMR spectrum (100 MHz, $CDCl_3$)

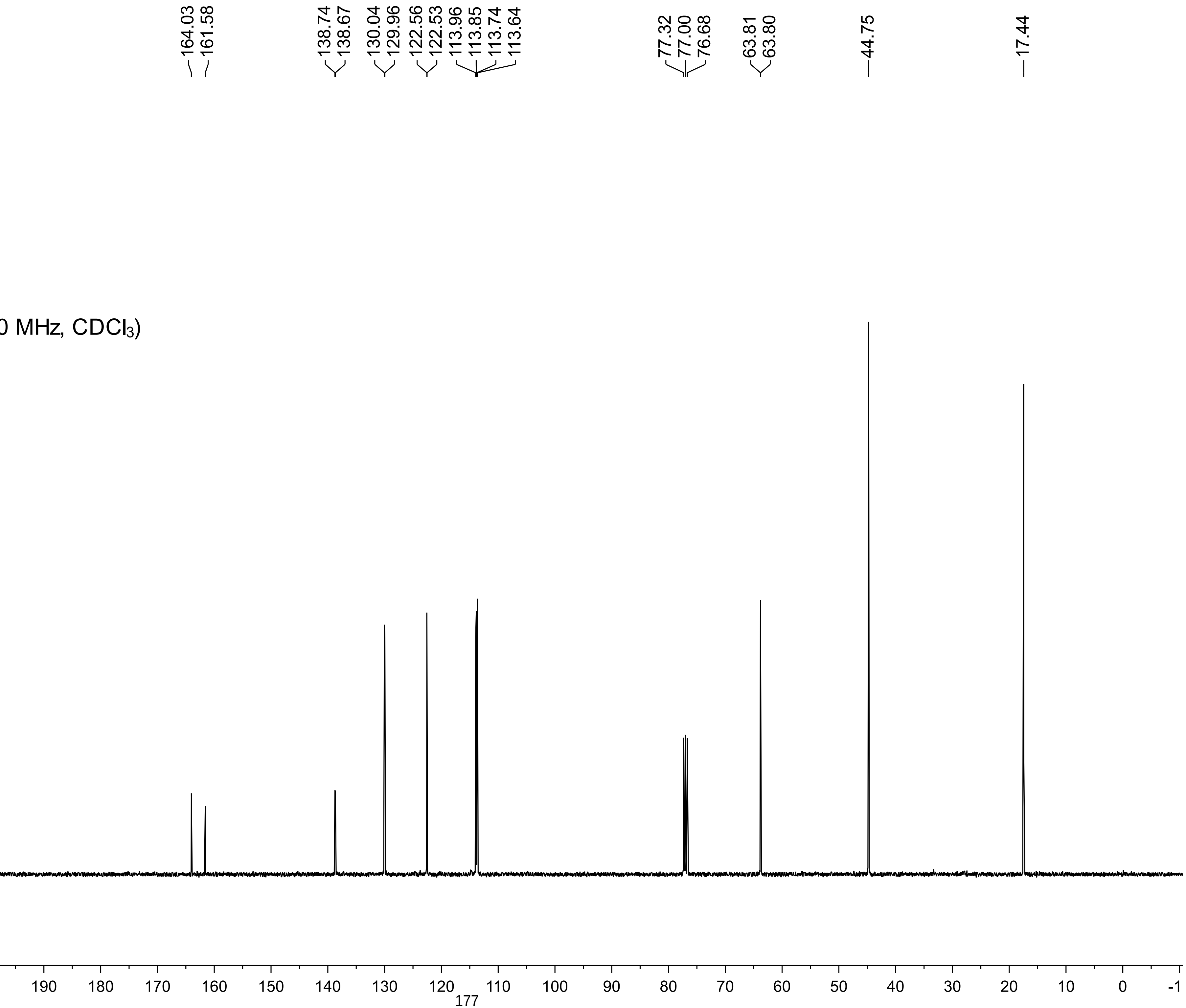

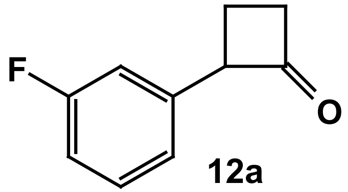

12a

[19]**F NMR** spectrum (376 MHz, $CDCl_3$)

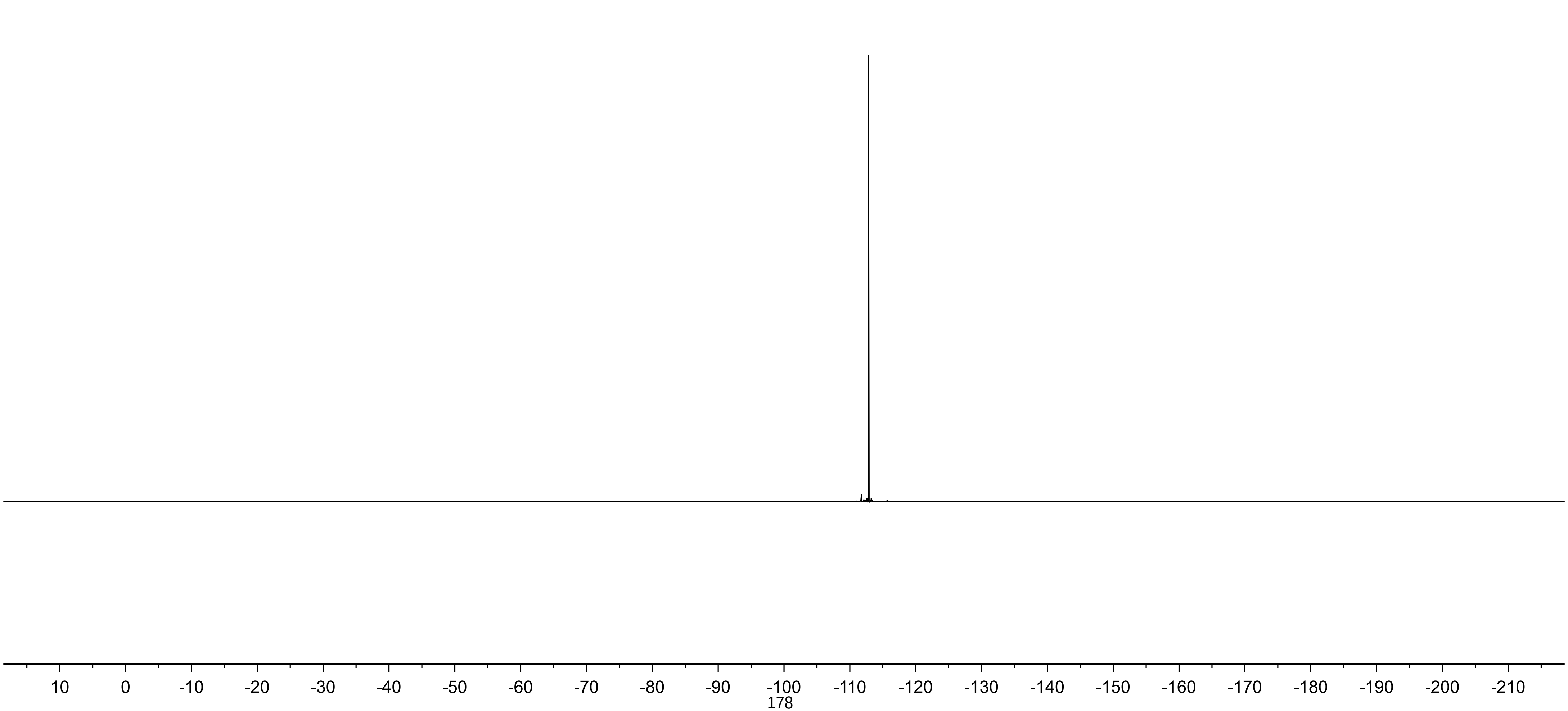

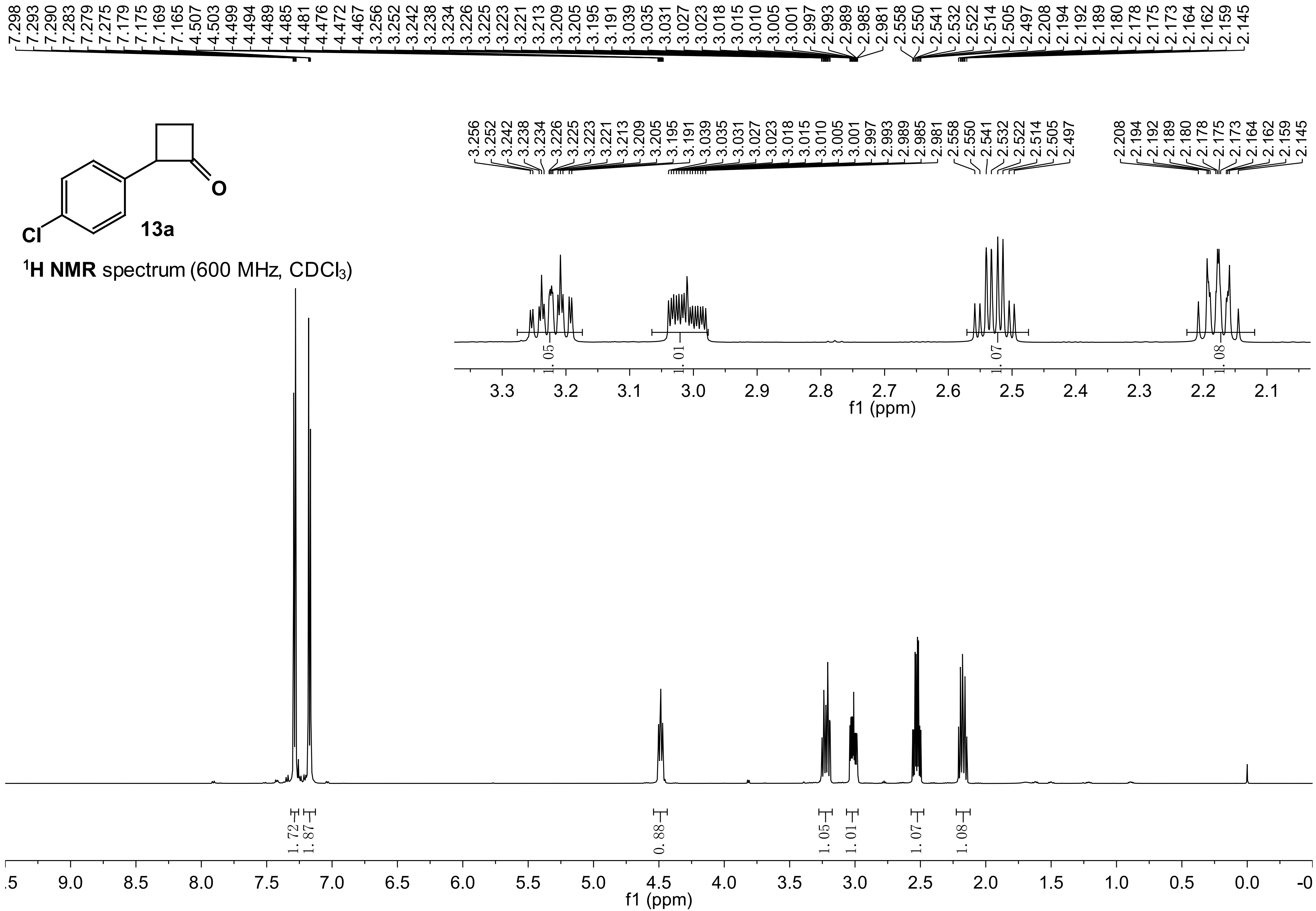

7.298 7.293 7.290 7.283 7.279 7.275 7.179 7.175 7.169 7.165 4.507 4.503 4.499 4.494 4.489 4.485 4.481 4.476 4.472 4.467 3.256 3.252 3.242 3.238 3.234 3.226 3.225 3.223 3.221 3.213 3.209 3.205 3.195 3.191 3.039 3.035 3.031 3.027 3.023 3.018 3.015 3.010 3.005 3.001 2.997 2.993 2.989 2.985 2.981 2.558 2.550 2.541 2.532 2.522 2.514 2.505 2.497 2.208 2.194 2.192 2.189 2.180 2.178 2.175 2.173 2.164 2.162 2.159 2.145
O
Cl
13a
1H NMR spectrum (600 MHz, CDCl3)
1.05
1.01
1.07
1.08
3.3 3.2 3.1 3.0 2.9 2.8 2.7 2.6 2.5 2.4 2.3 2.2 2.1
f1 (ppm)
1.72 1.87 0.88 1.05 1.01 1.07 1.08
.5 9.0 8.5 8.0 7.5 7.0 6.5 6.0 5.5 5.0 4.5 4.0 3.5 3.0 2.5 2.0 1.5 1.0 0.5 0.0 -0
f1 (ppm)

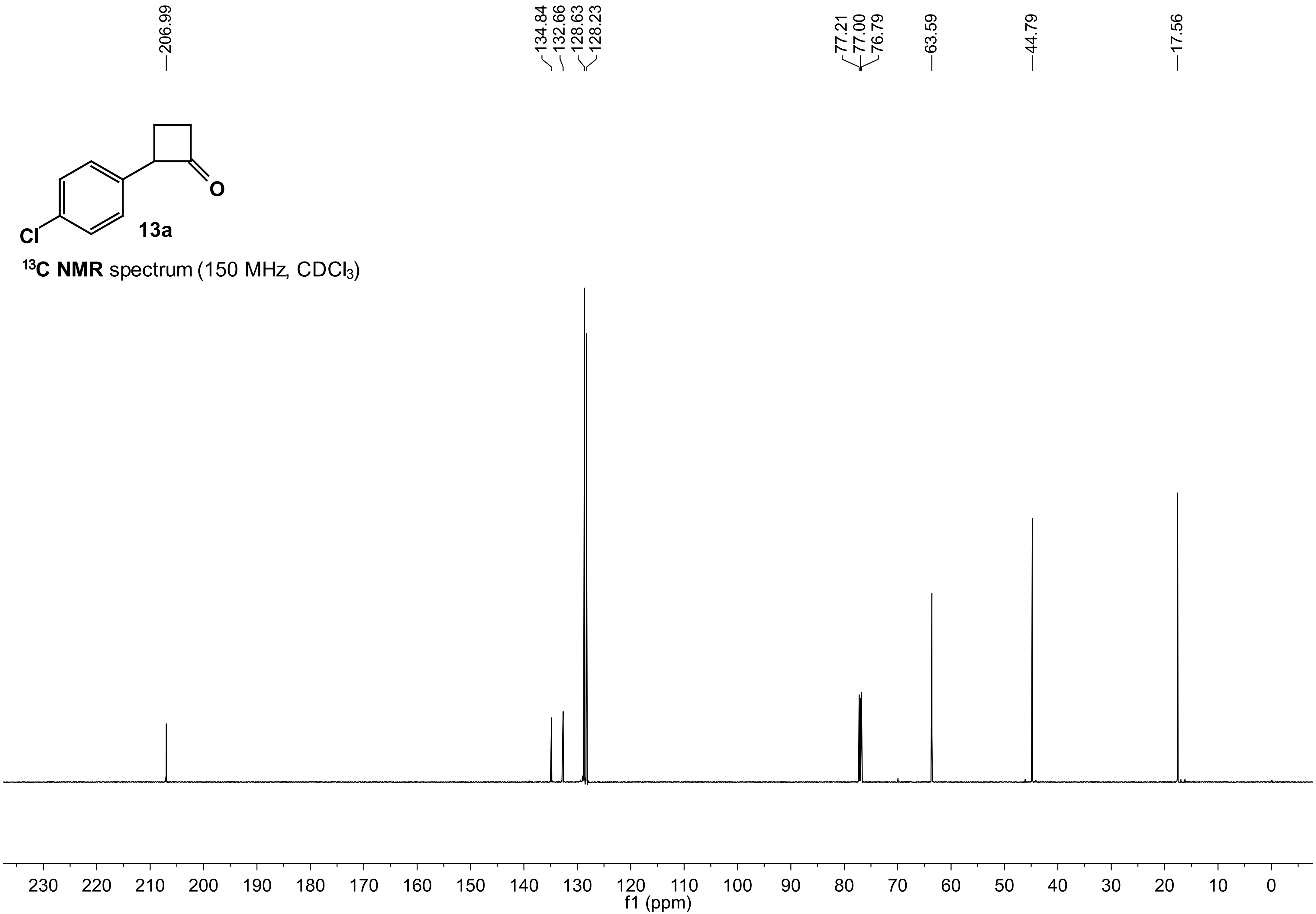

13a

^{13}**C NMR** spectrum (150 MHz, $CDCl_3$)

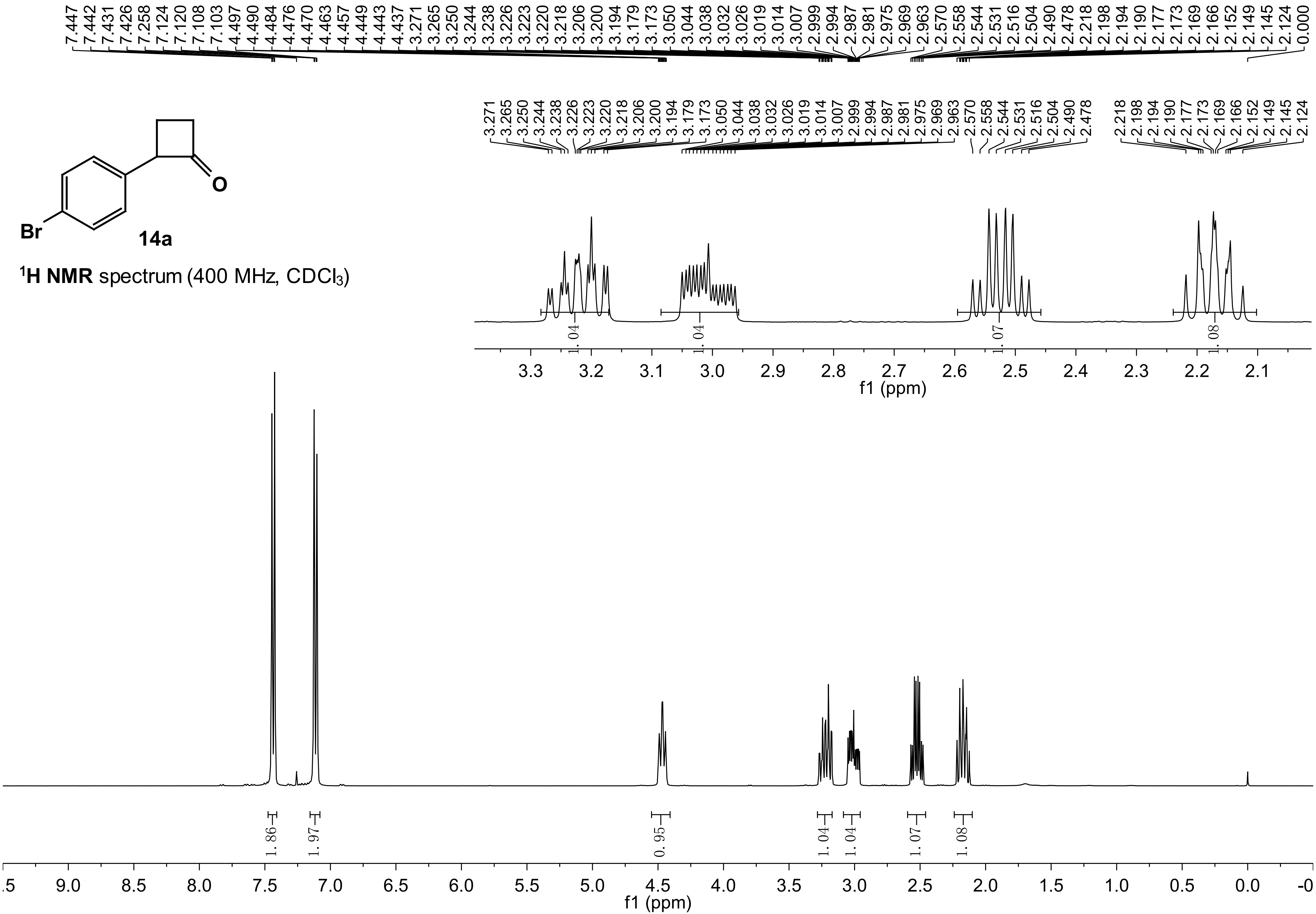

^{1}H NMR spectrum (400 MHz, $CDCl_3$)

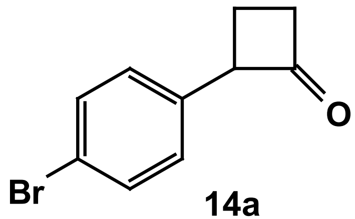

14a

^{13}C NMR spectrum (100 MHz, $CDCl_3$)

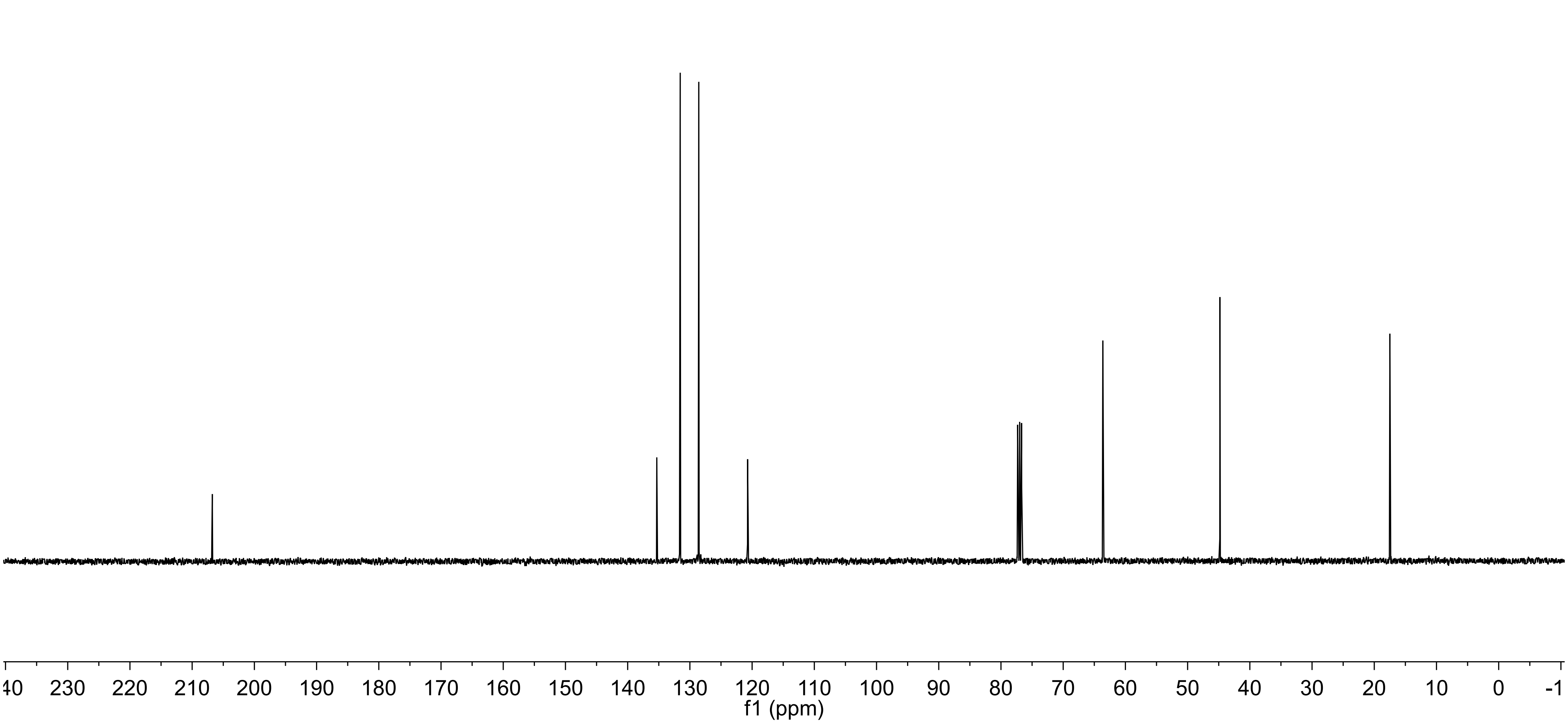

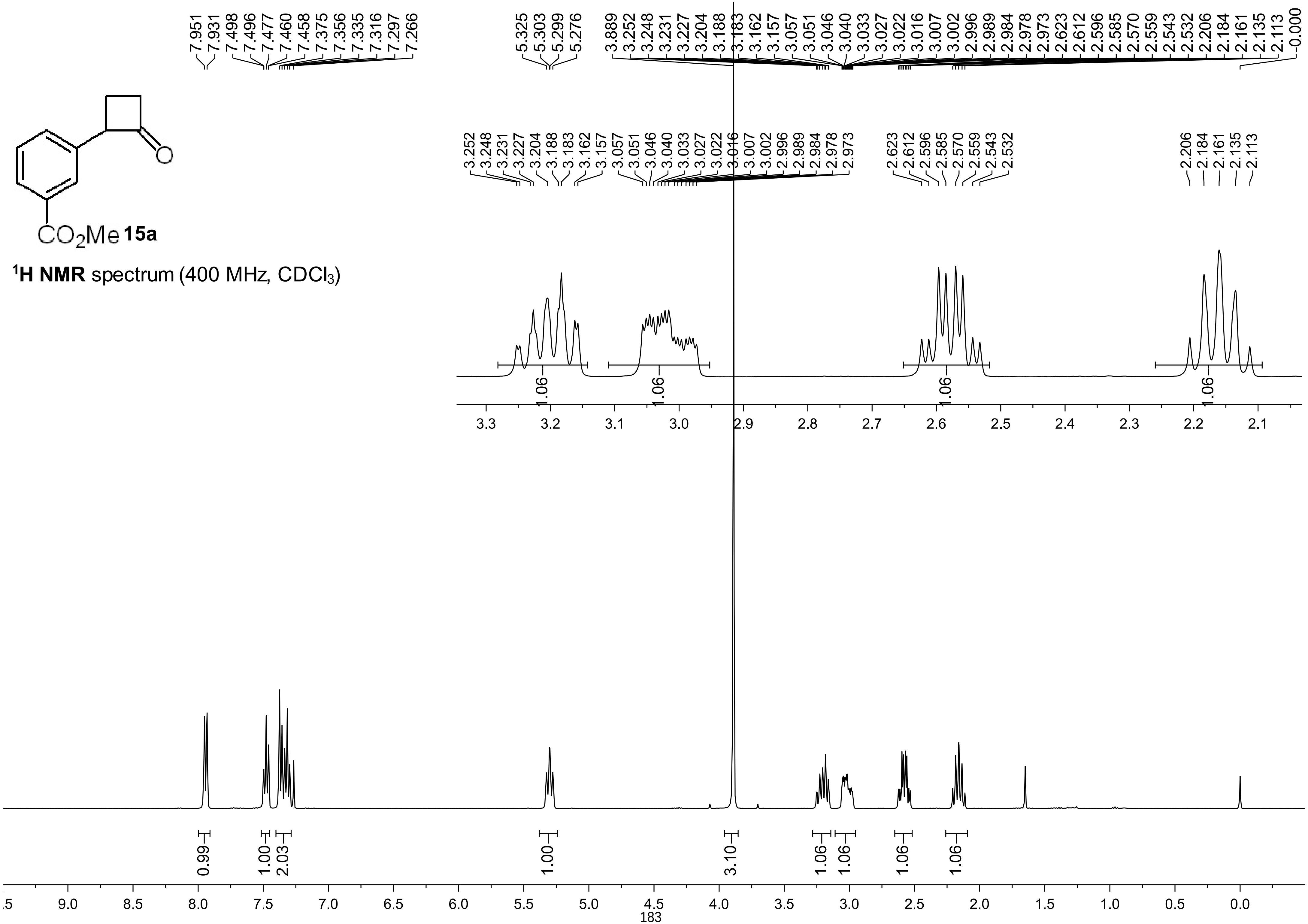

^{1}H NMR spectrum (400 MHz, $CDCl_3$)

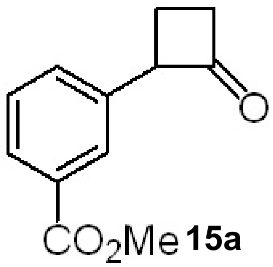

15a

[13]**C NMR** spectrum (100 MHz, $CDCl_3$)

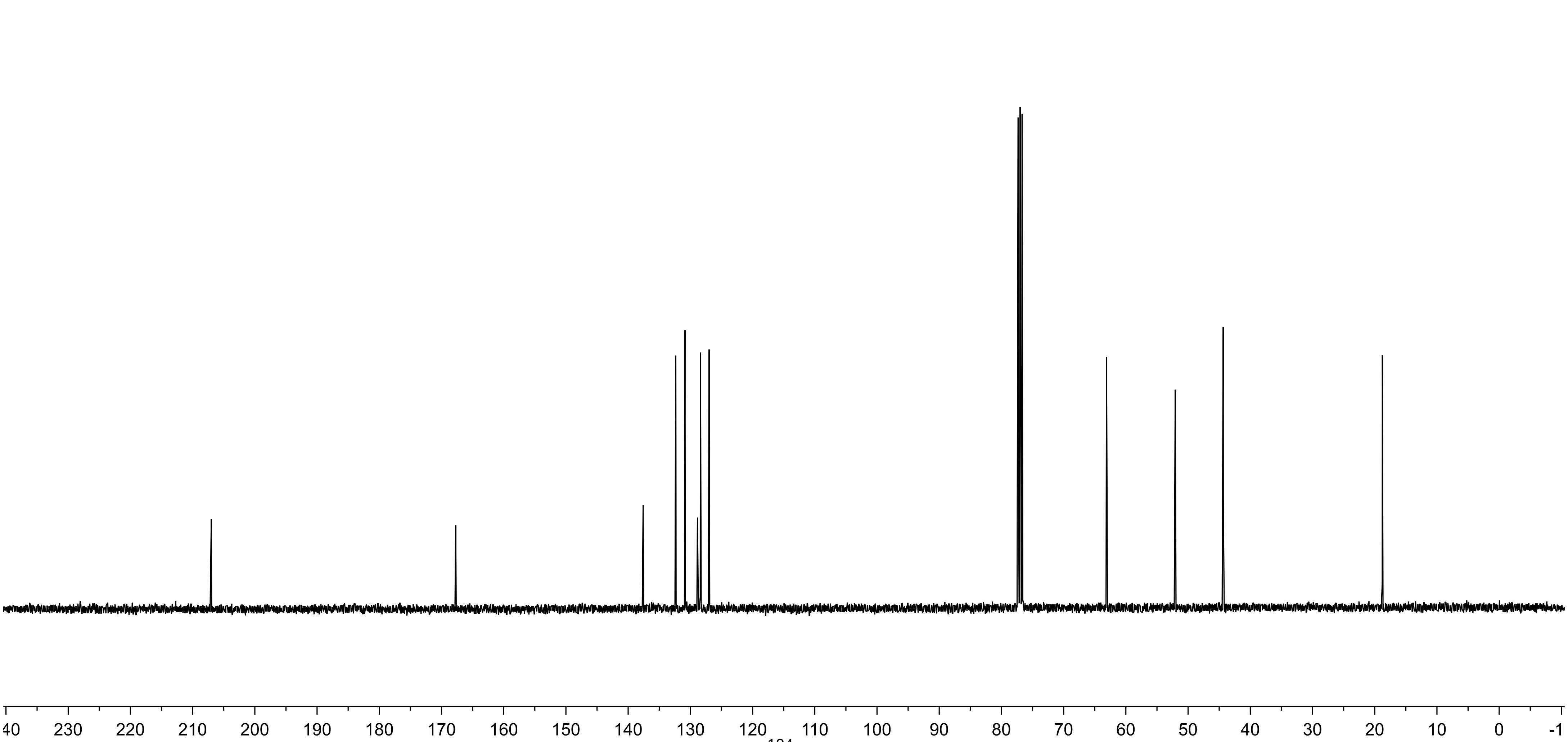

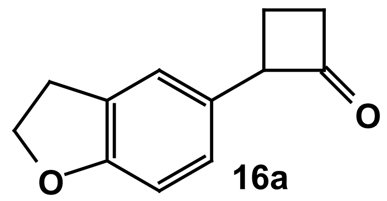

16a

[1]**H NMR** spectrum (400 MHz, $CDCl_3$)

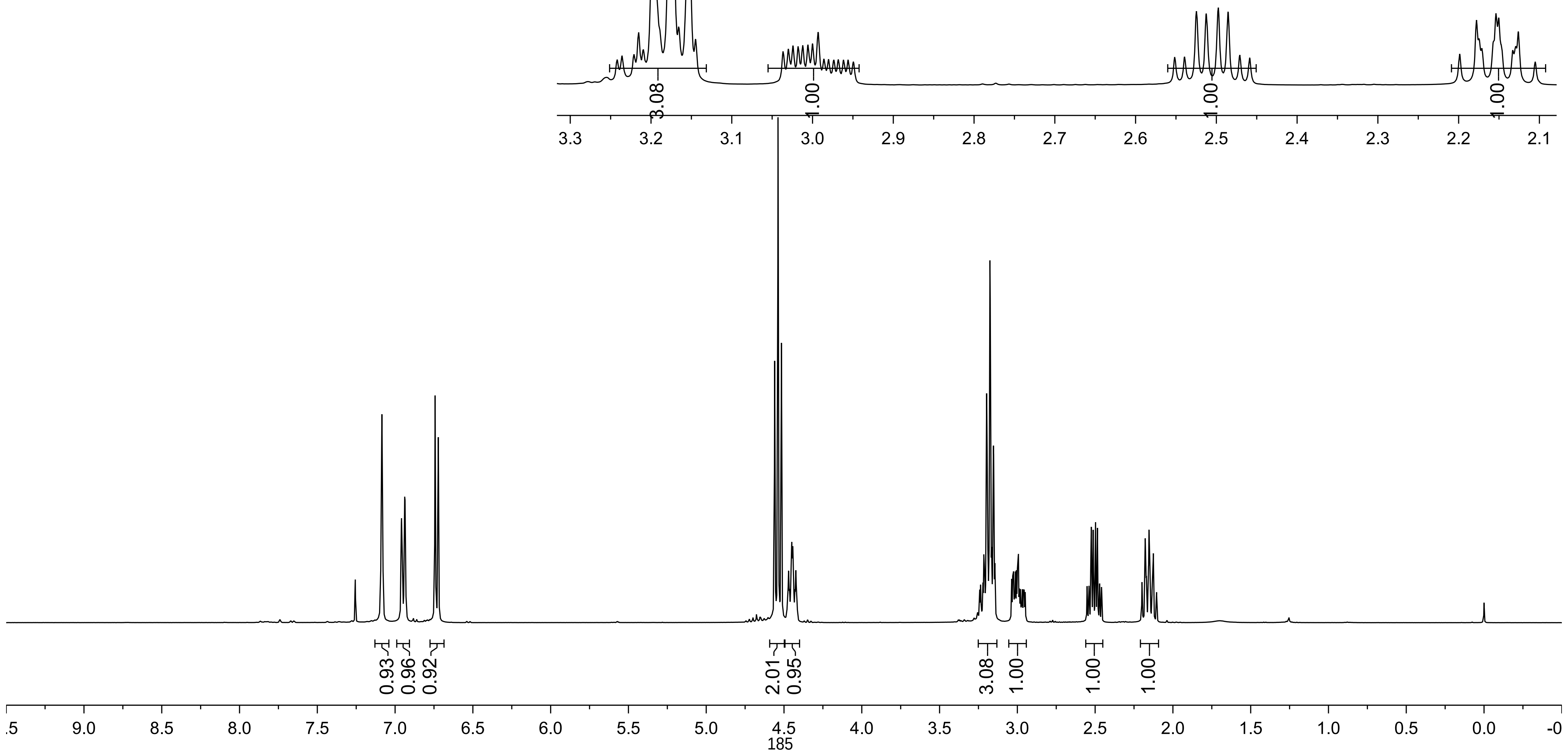

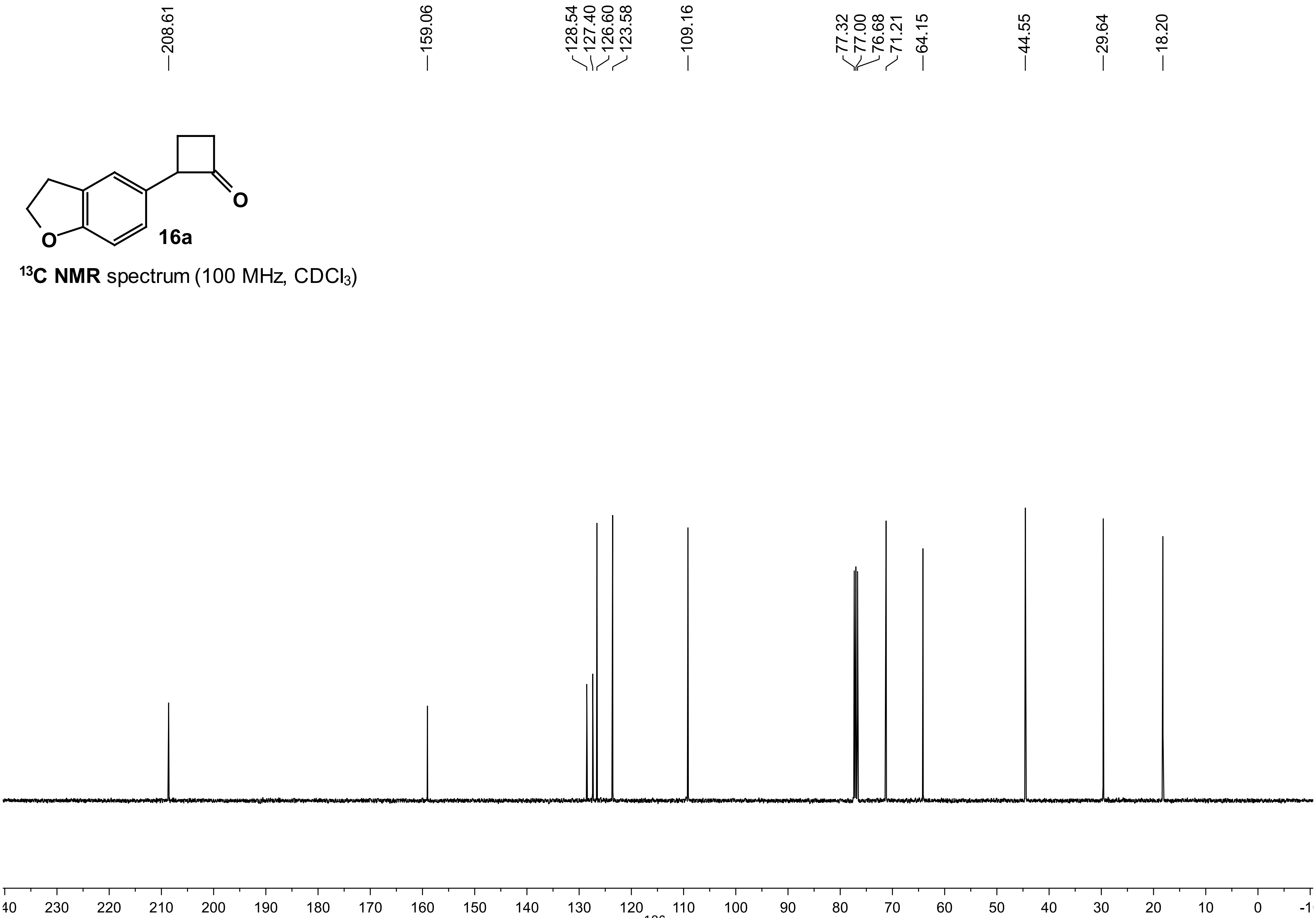

^{13}C NMR spectrum (100 MHz, $CDCl_3$)

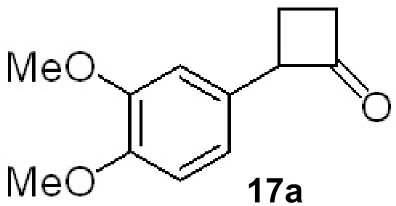

17a

^{1}H NMR spectrum (600 MHz, $CDCl_3$)

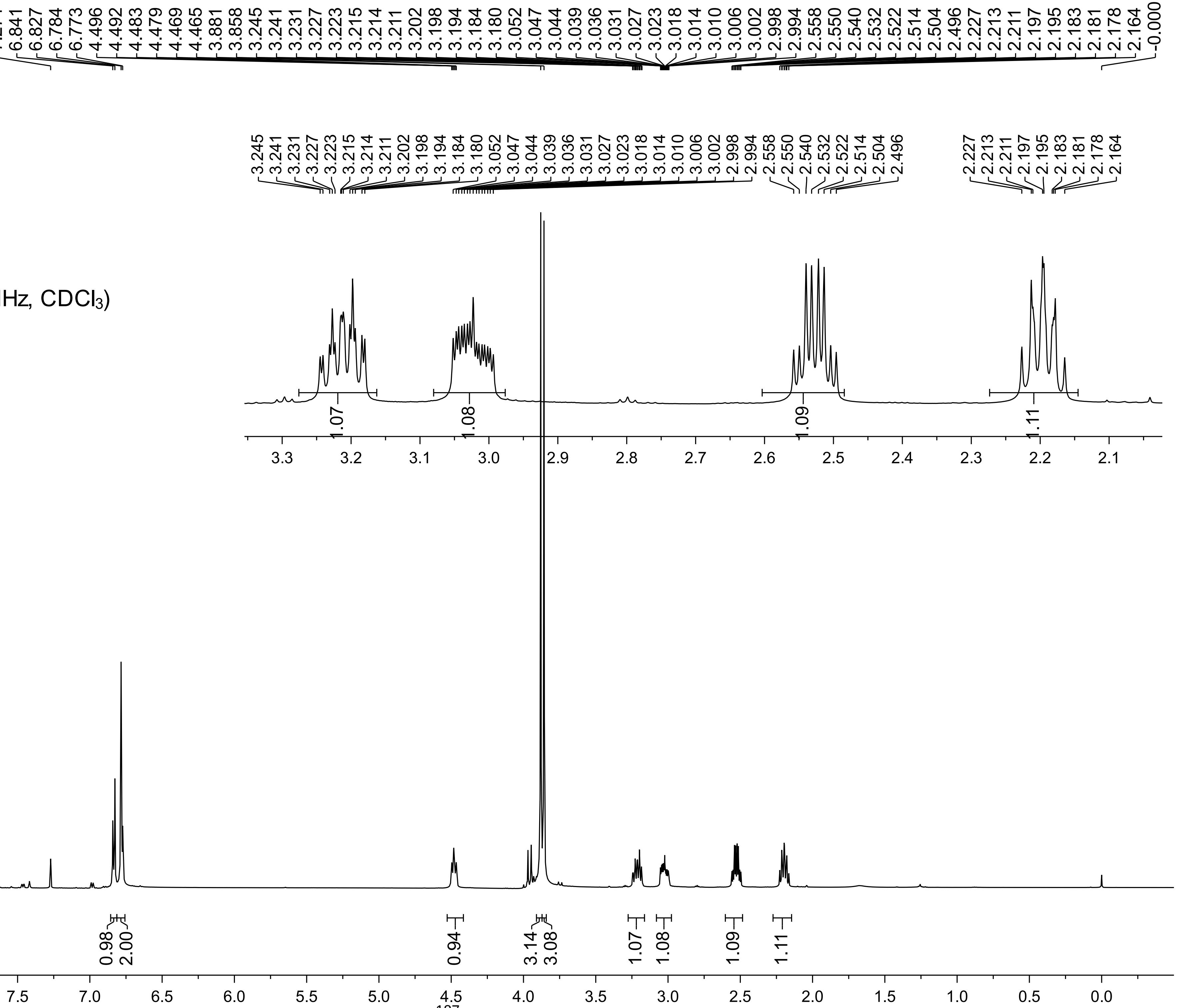

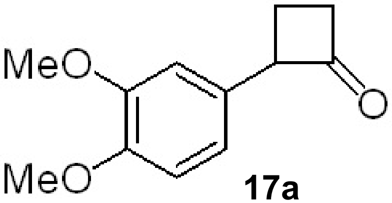

17a

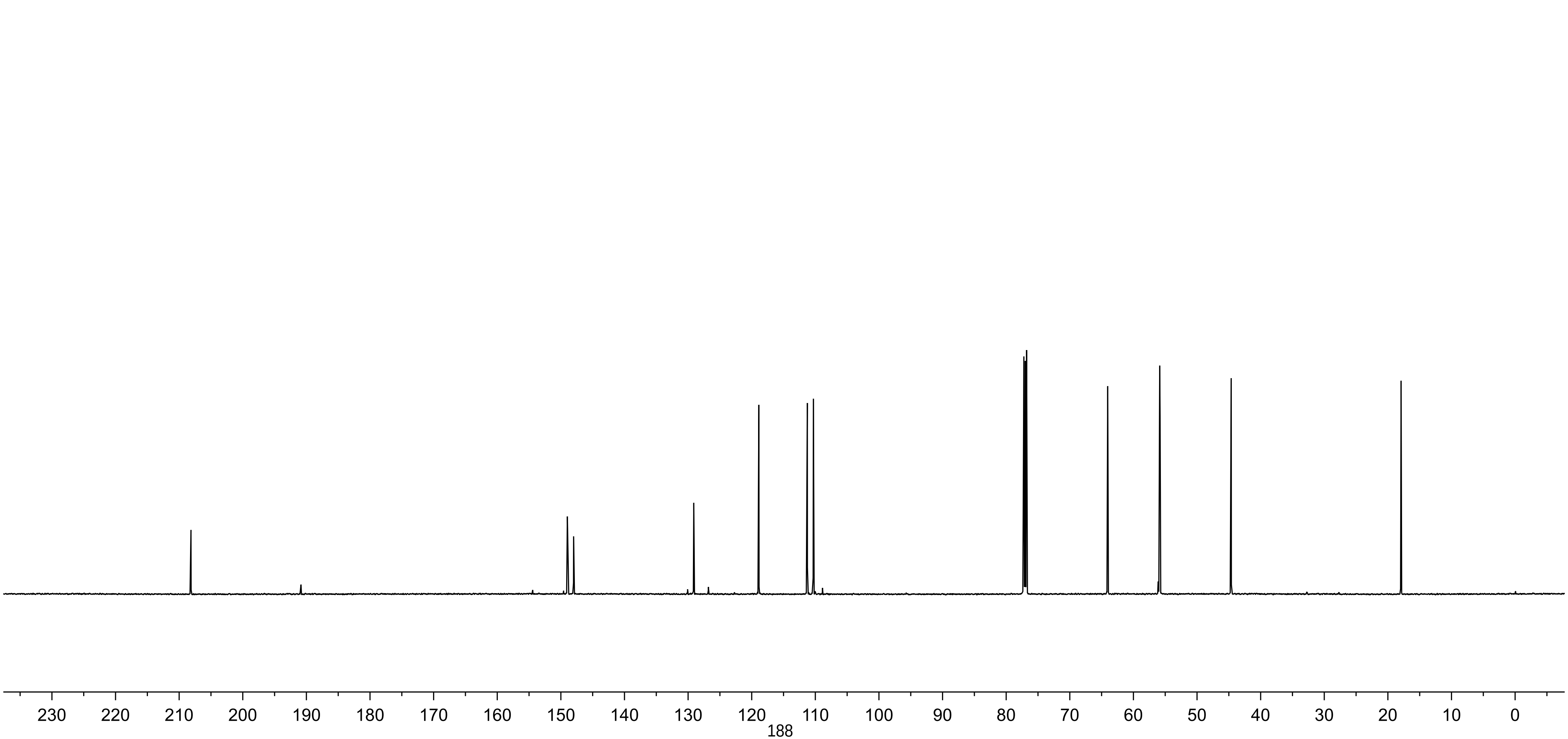

^{13}**C NMR** spectrum (150 MHz, $CDCl_3$)

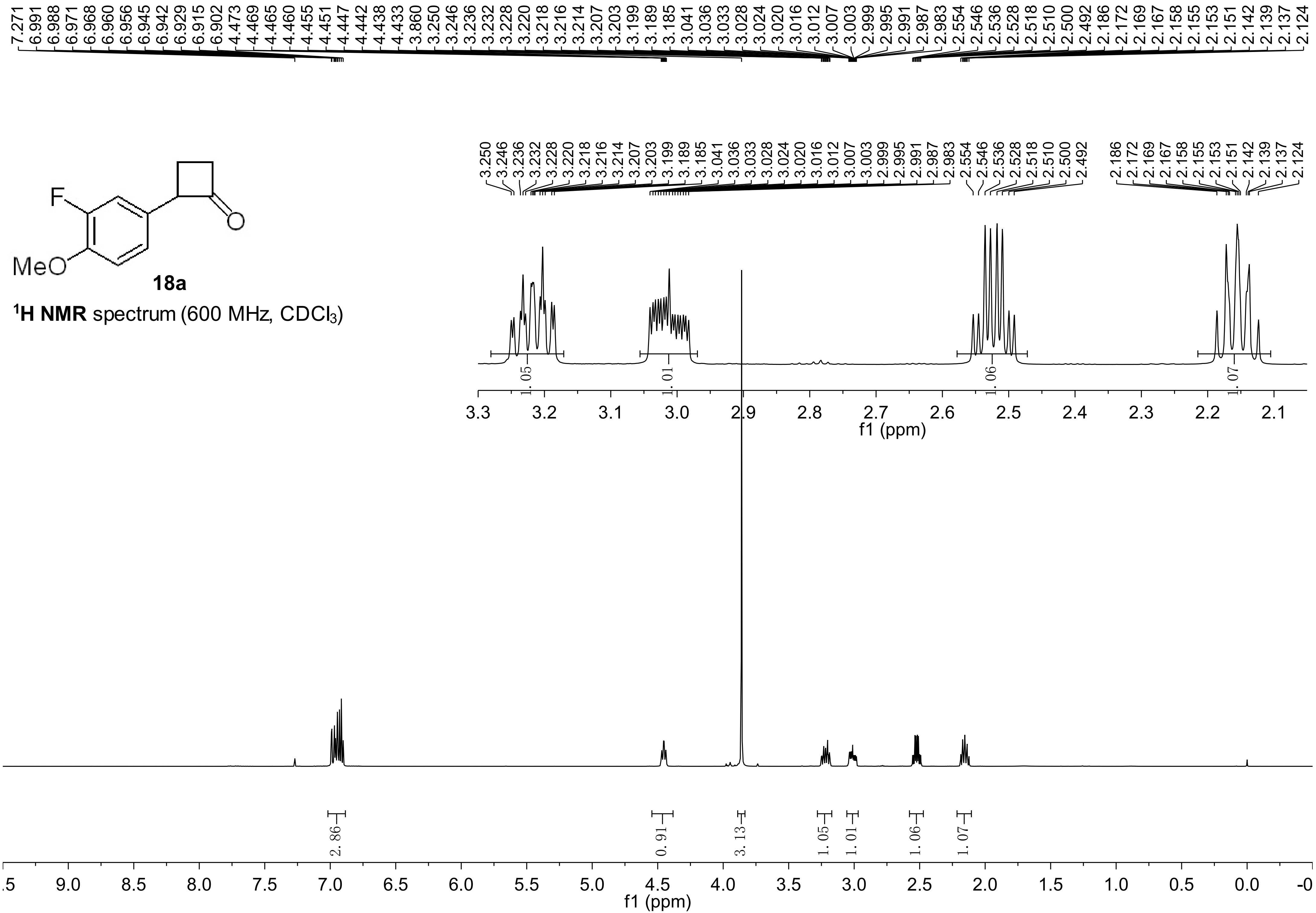
7.271 6.991 6.988 6.971 6.968 6.960 6.956 6.945 6.942 6.929 6.915 6.902 4.473 4.469 4.465 4.460 4.455 4.451 4.447 4.442 4.438 4.433 3.860 3.250 3.246 3.236 3.232 3.228 3.220 3.218 3.216 3.214 3.207 3.203 3.199 3.189 3.185 3.041 3.036 3.033 3.028 3.024 3.020 3.016 3.012 3.007 3.003 2.999 2.995 2.991 2.987 2.983 2.554 2.546 2.536 2.528 2.518 2.510 2.500 2.492 2.186 2.172 2.169 2.167 2.158 2.155 2.153 2.151 2.142 2.139 2.137 2.124
F
MeO
O
18a
1H NMR spectrum (600 MHz, CDCl3)
1.05 1.01 1.06 1.07
f1 (ppm)
2.86 0.91 3.13 1.05 1.01 1.06 1.07
f1 (ppm)

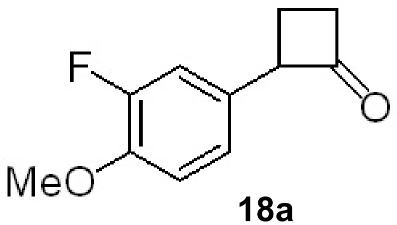

18a

^{13}C NMR spectrum (150 MHz, $CDCl_3$)

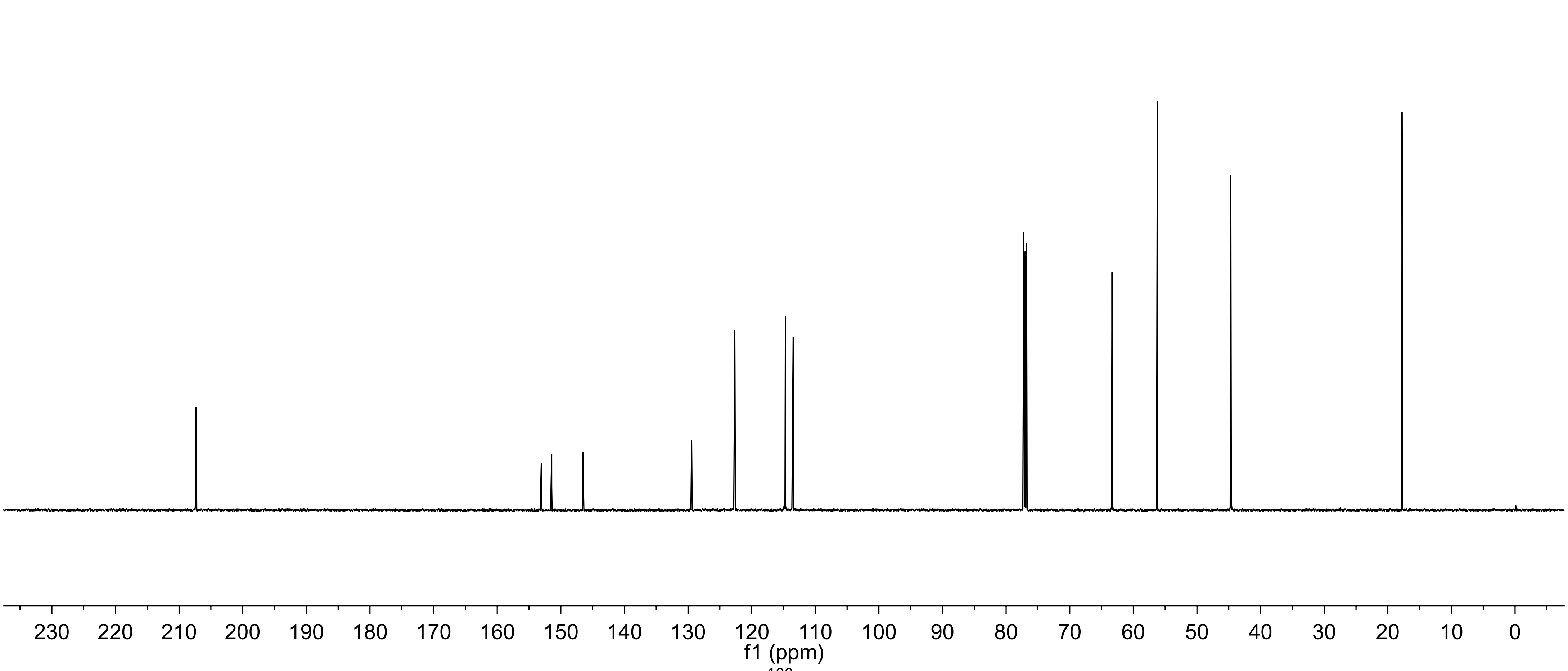

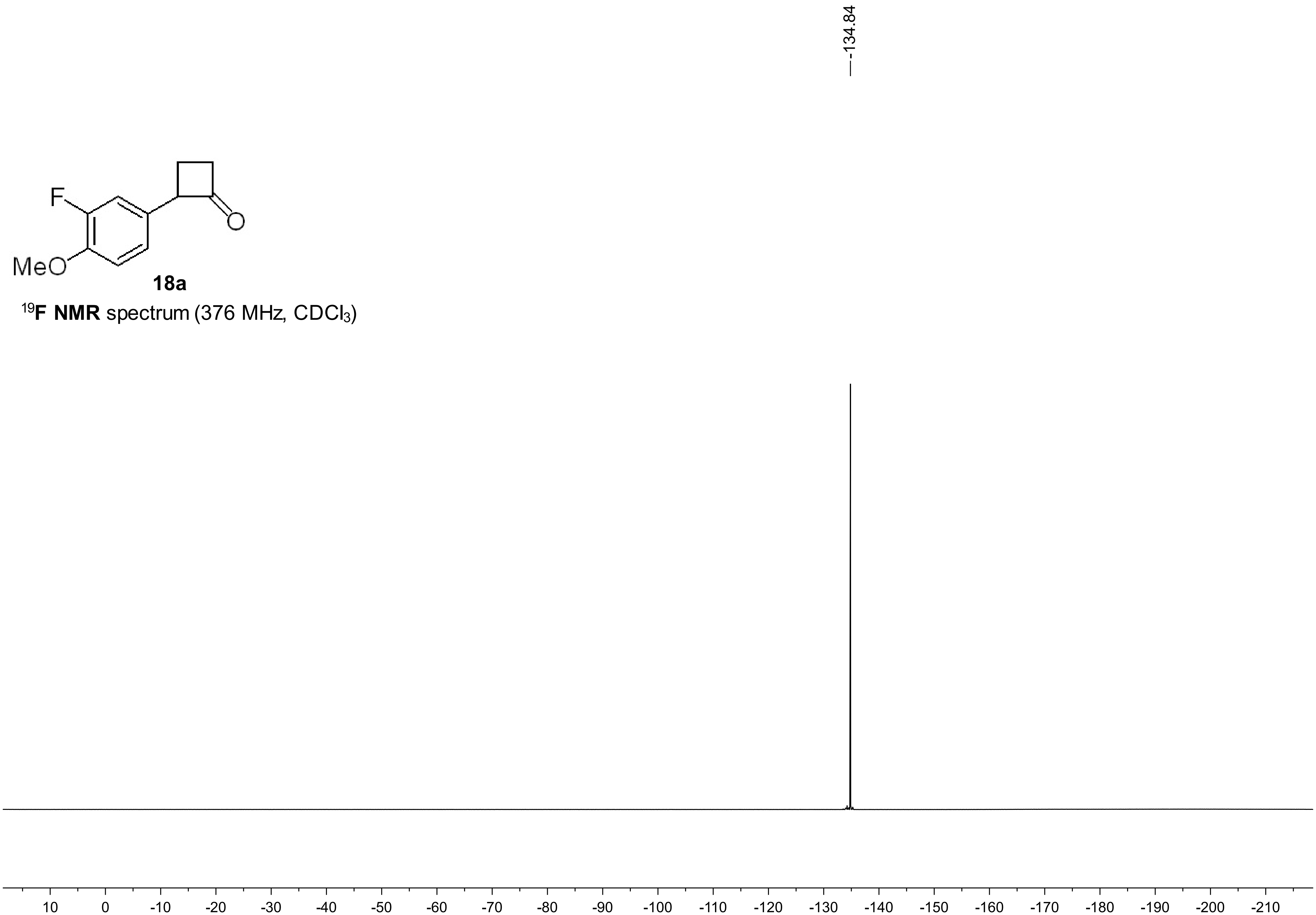

18a

^{19}**F NMR** spectrum (376 MHz, $CDCl_3$)

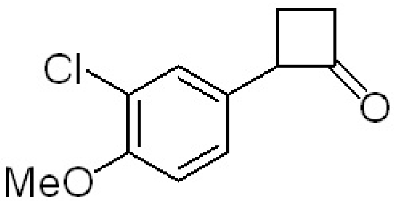

19a

^{1}H NMR spectrum (600 MHz, $CDCl_3$)

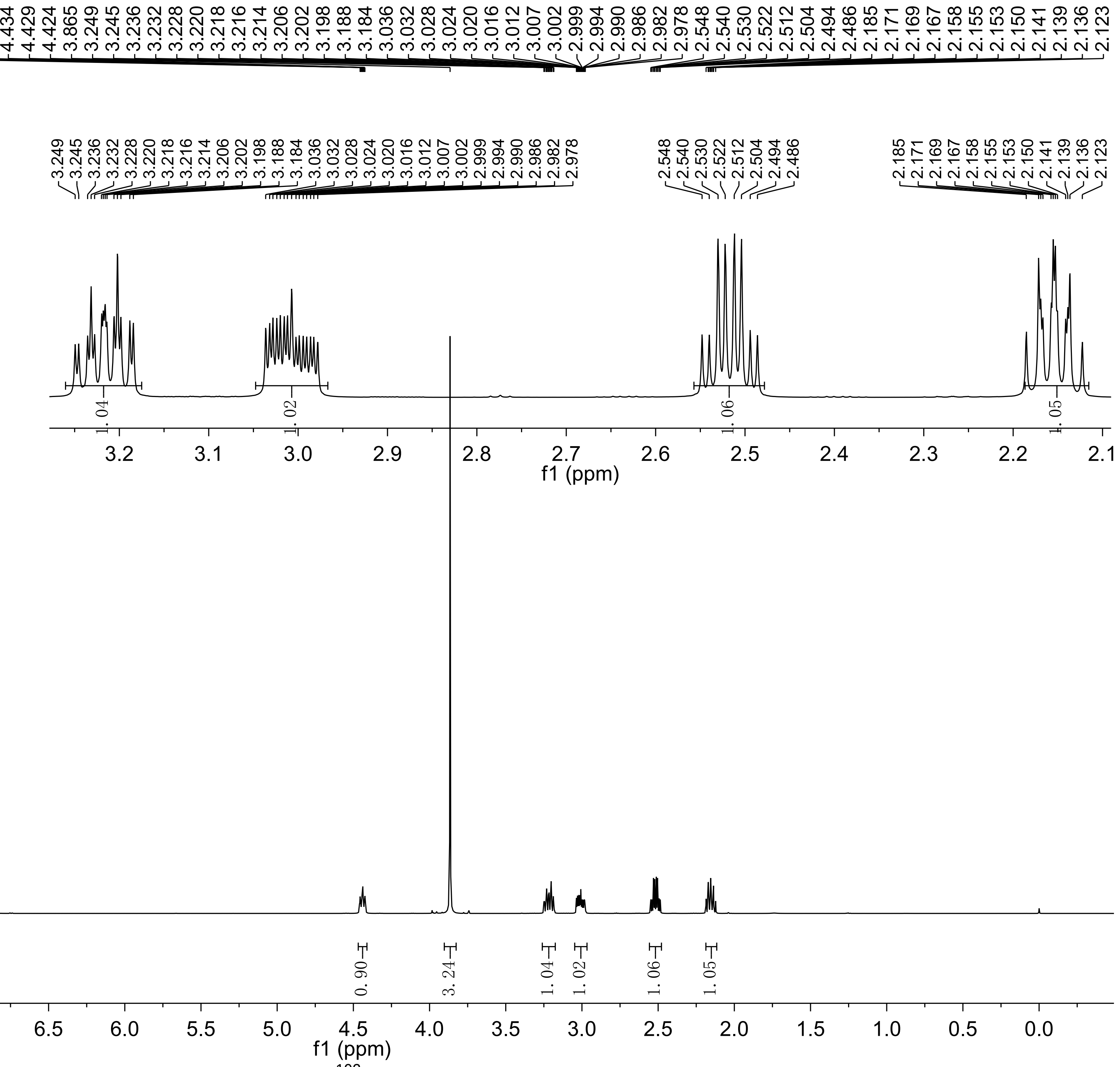

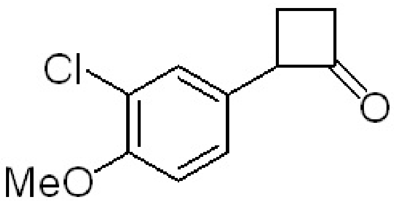

19a

^{13}**C NMR** spectrum (150 MHz, $CDCl_3$)

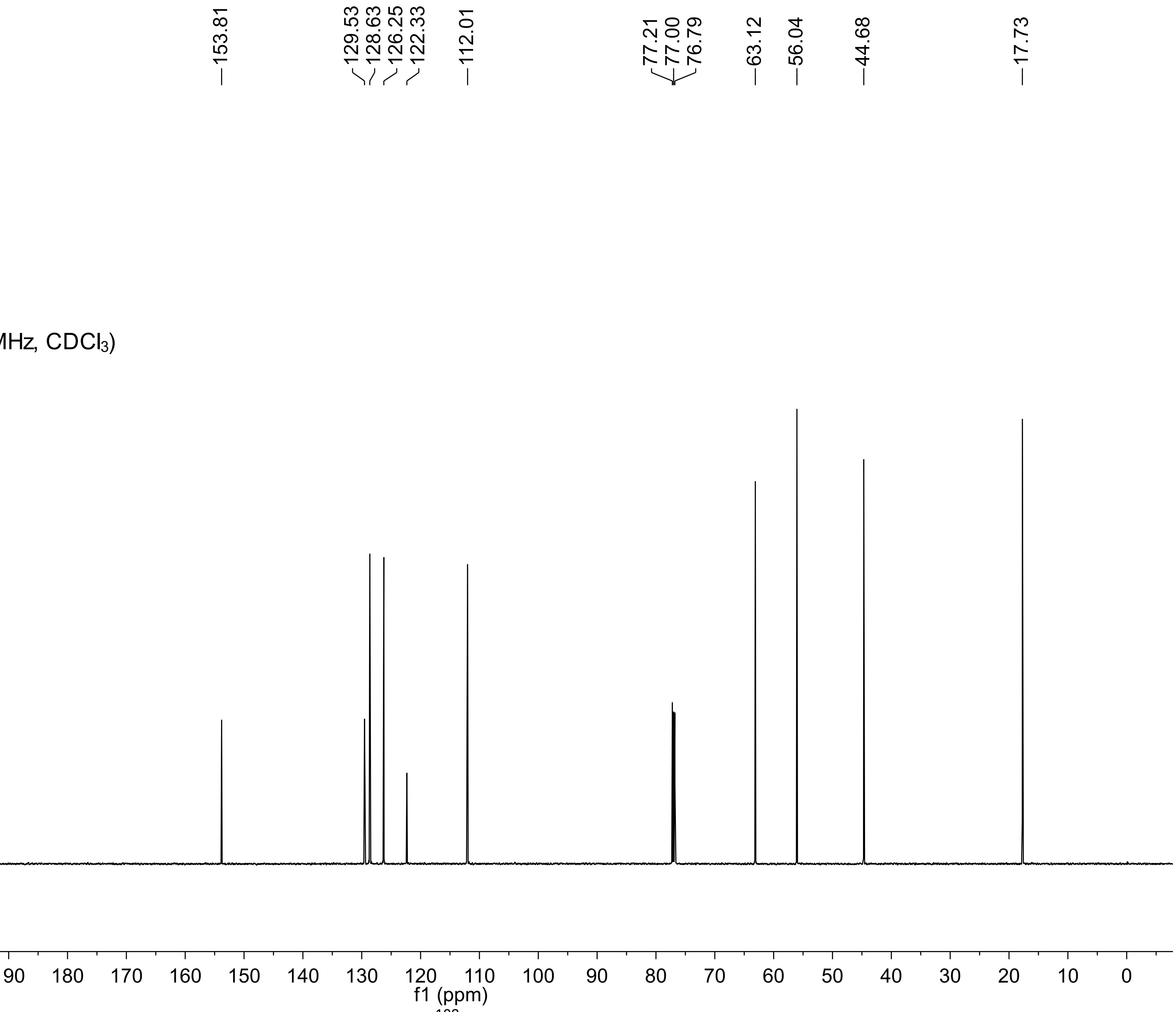

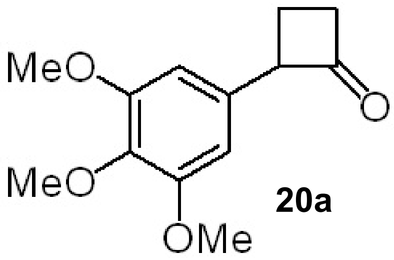

20a

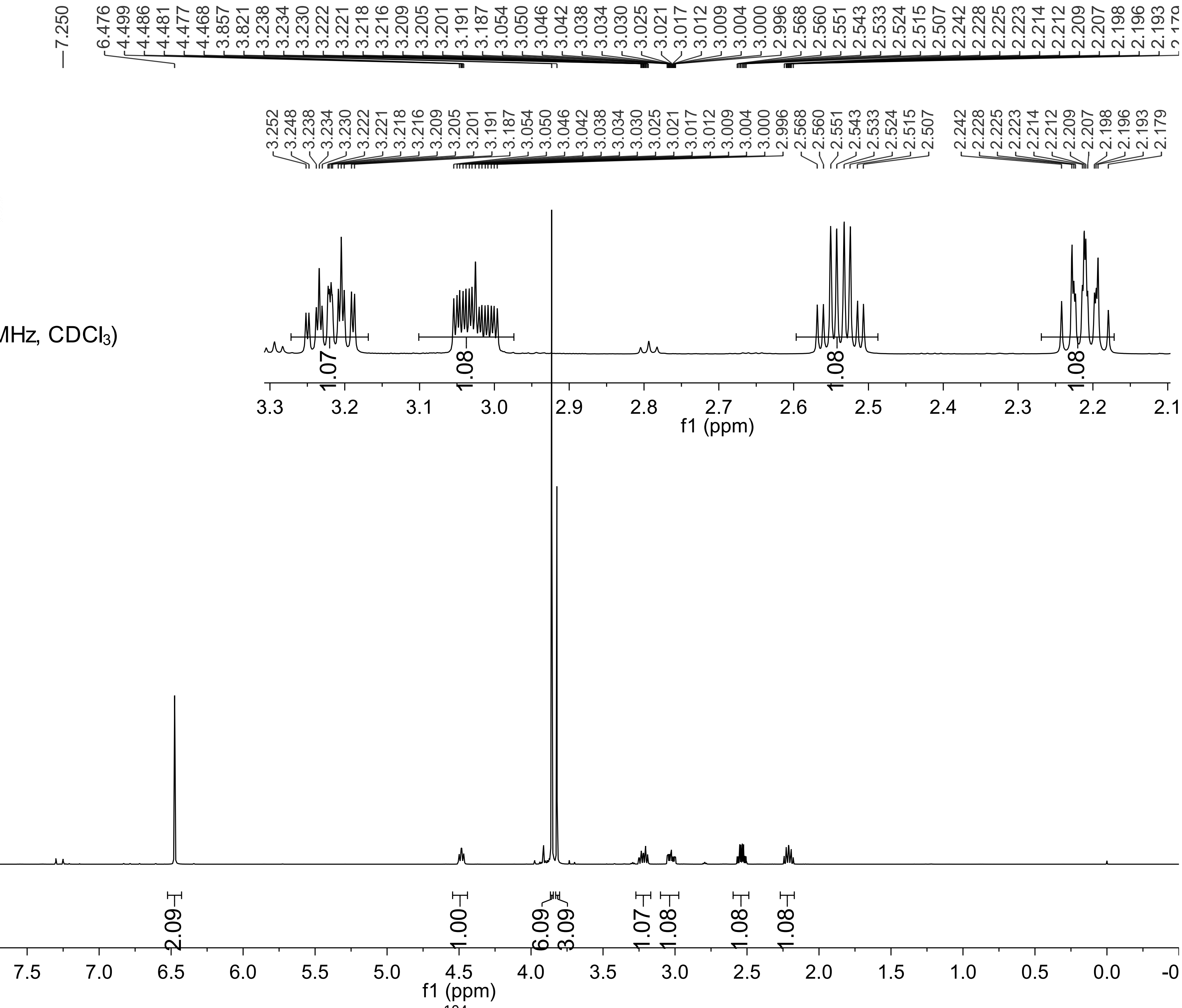

¹H NMR spectrum (600 MHz, $CDCl_3$)

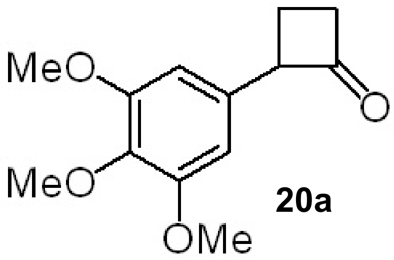

20a

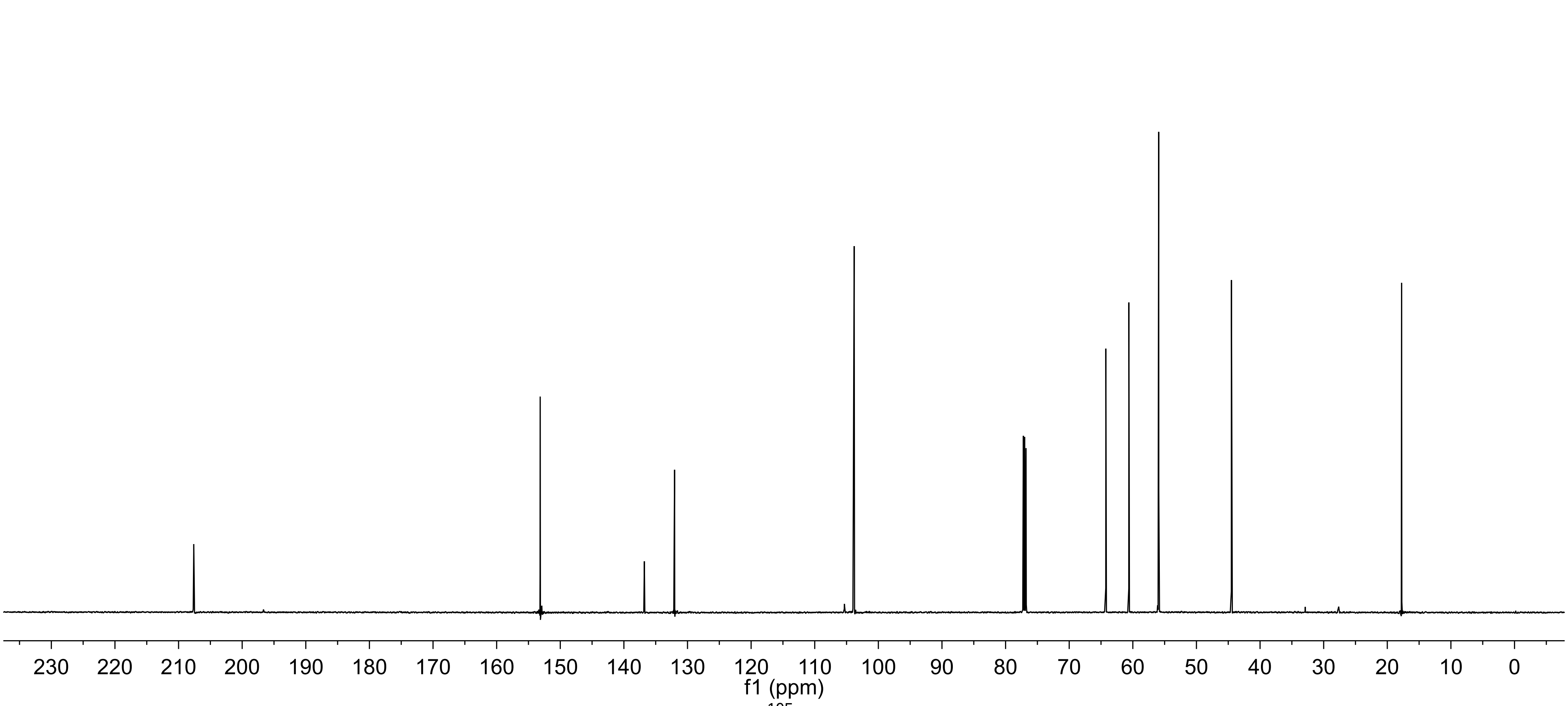

^{13}**C NMR** spectrum (150 MHz, $CDCl_3$)

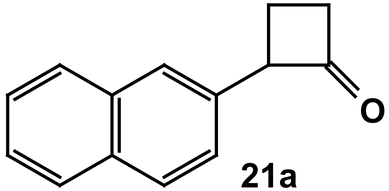

21a

^{1}H NMR spectrum (400 MHz, $CDCl_3$)

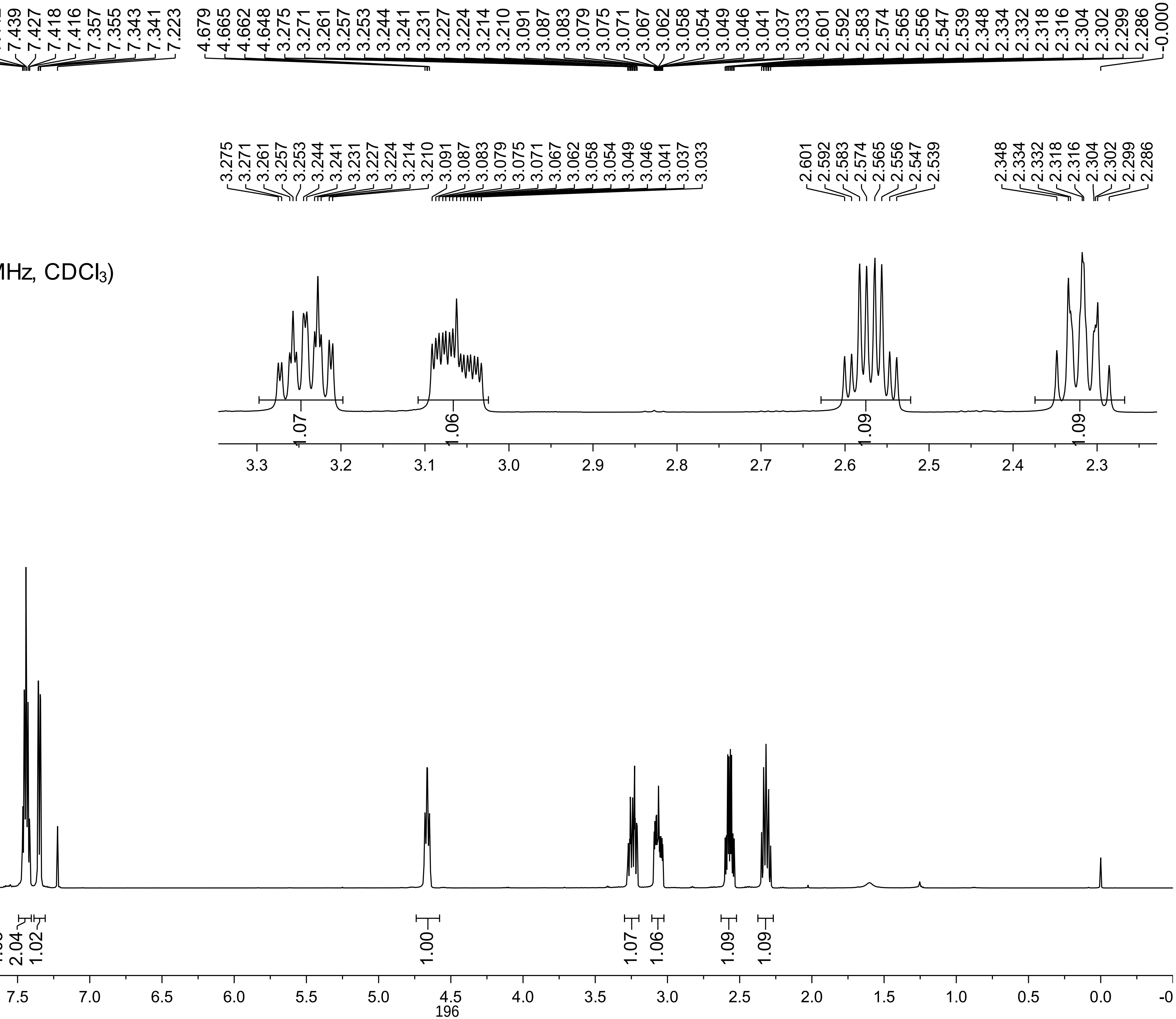

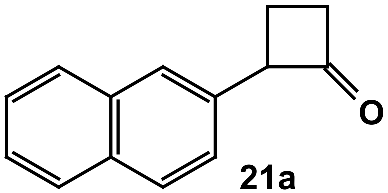

21a

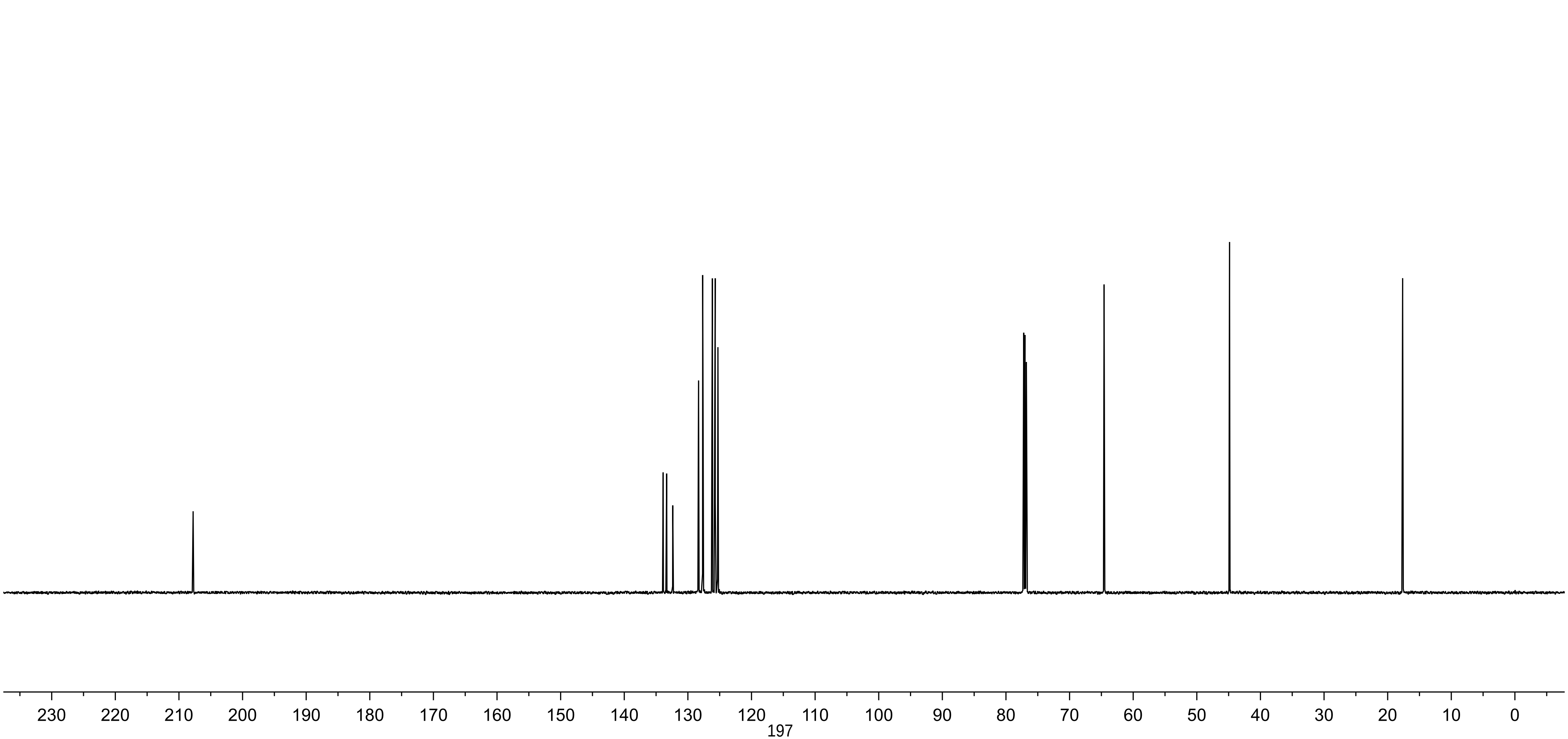

^{13}**C NMR** spectrum (100 MHz, $CDCl_3$)

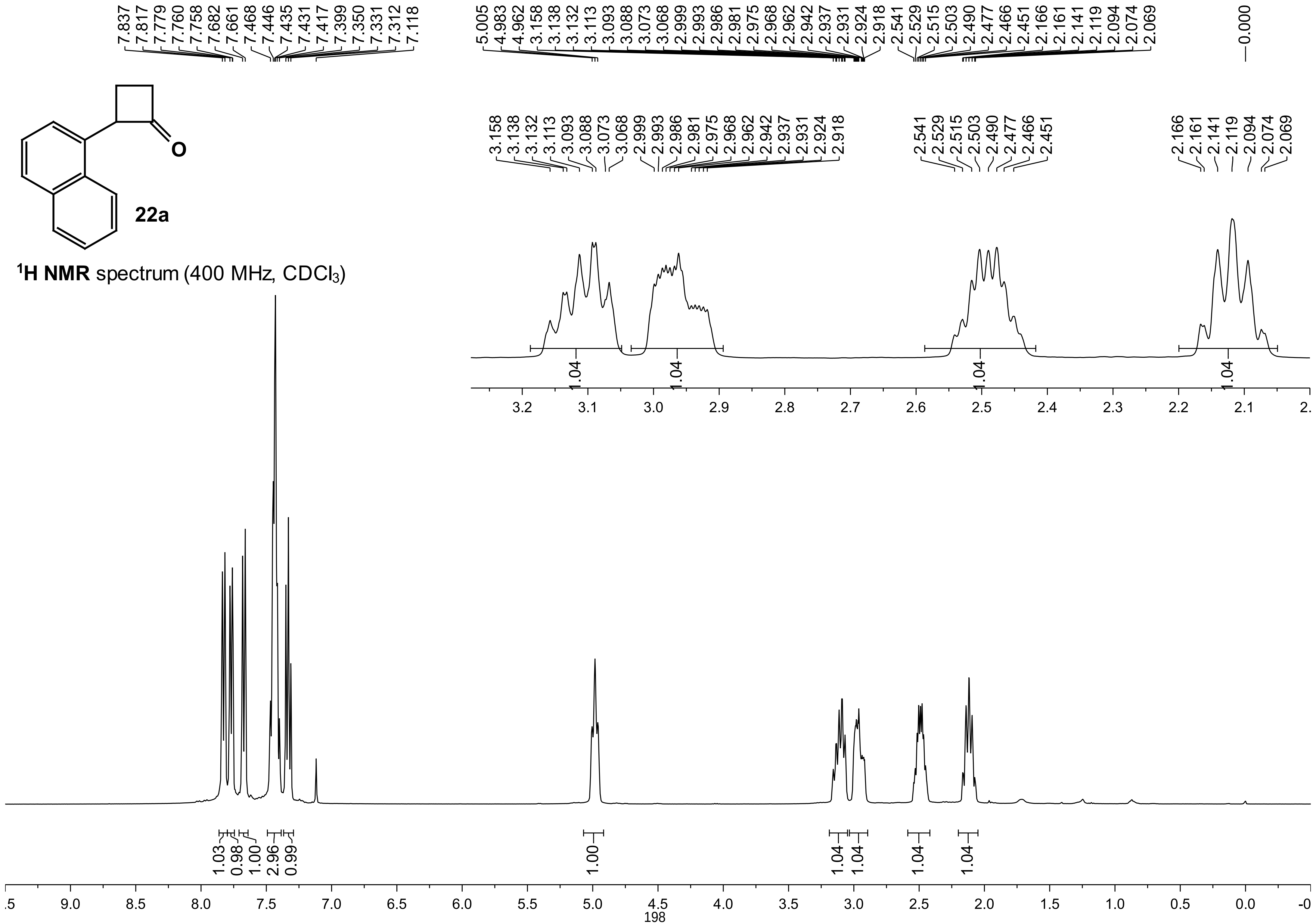

^{1}H NMR spectrum (400 MHz, $CDCl_3$)

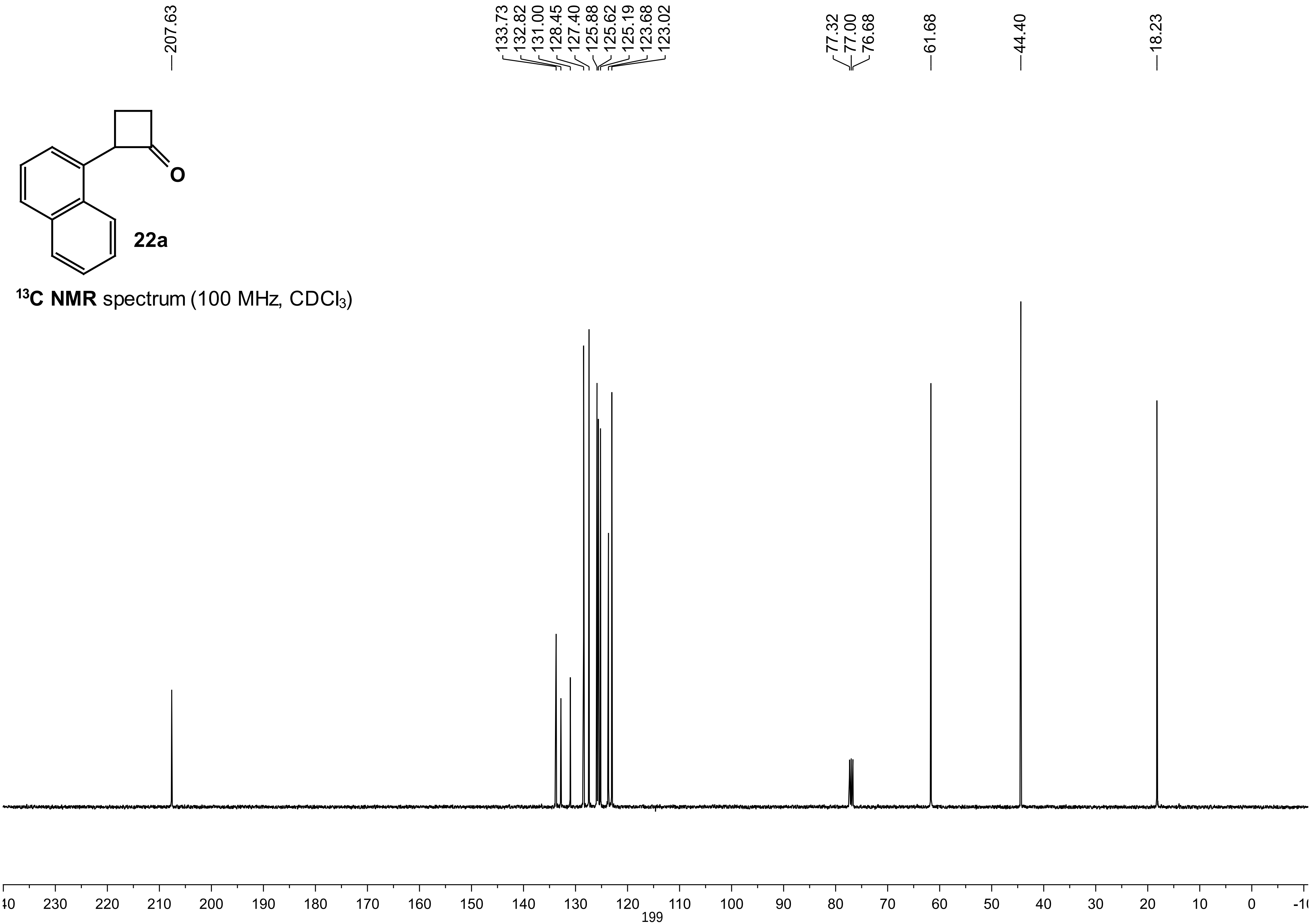
207.63
133.73
132.82
131.00
128.45
127.40
125.88
125.62
125.19
123.68
123.02
77.32
77.00
76.68
61.68
44.40
18.23
O
22a
13C NMR spectrum (100 MHz, CDCl3)
230
220
210
200
190
180
170
160
150
140
130
120
110
100
90
80
70
60
50
40
30
20
10
0

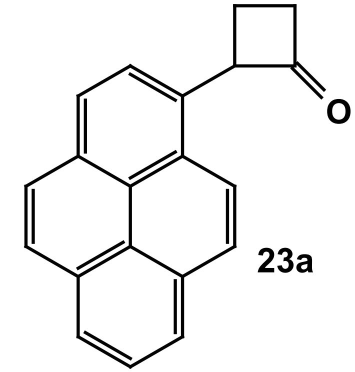

23a

[1]**H NMR** spectrum (400 MHz, $CDCl_3$)

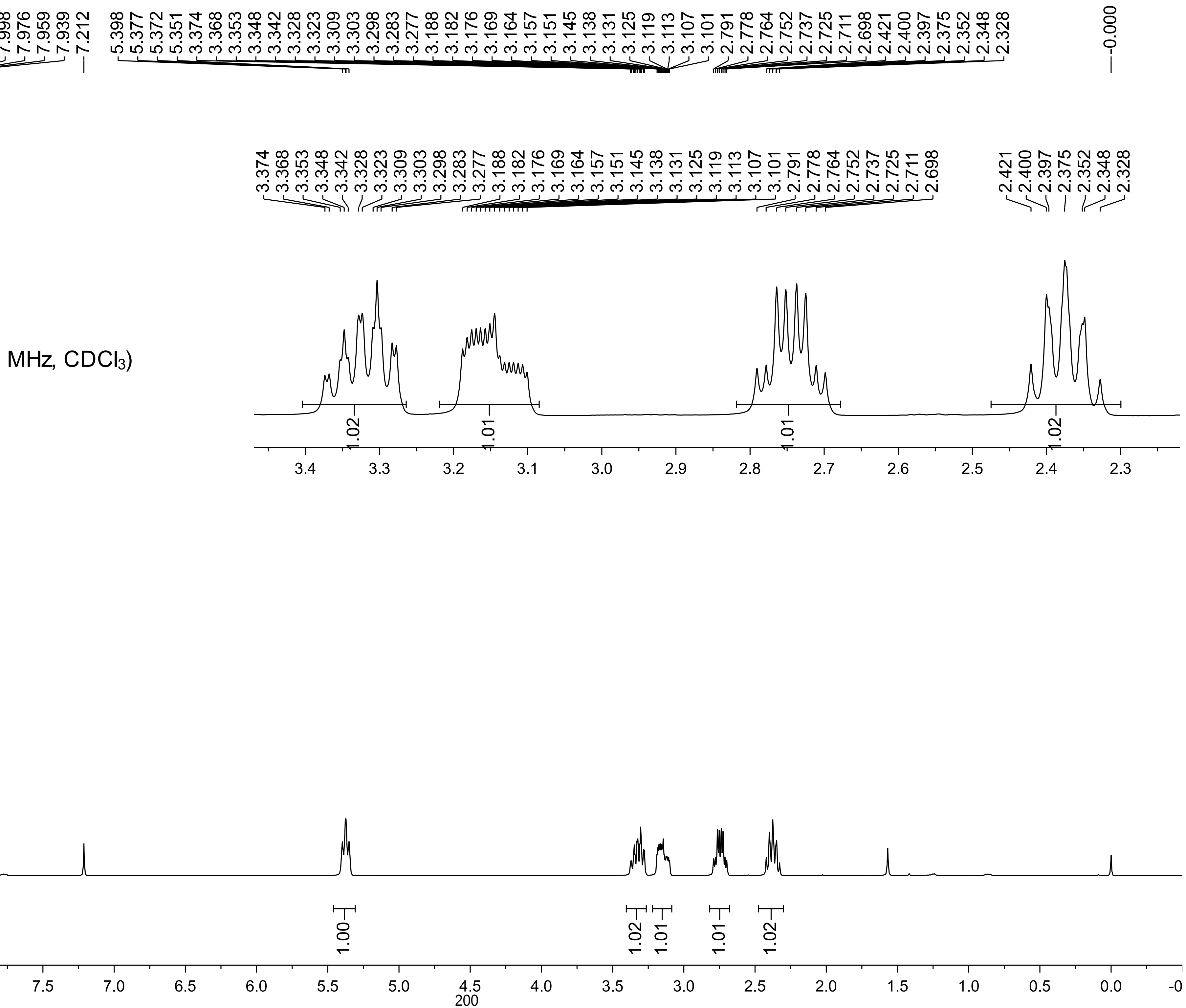

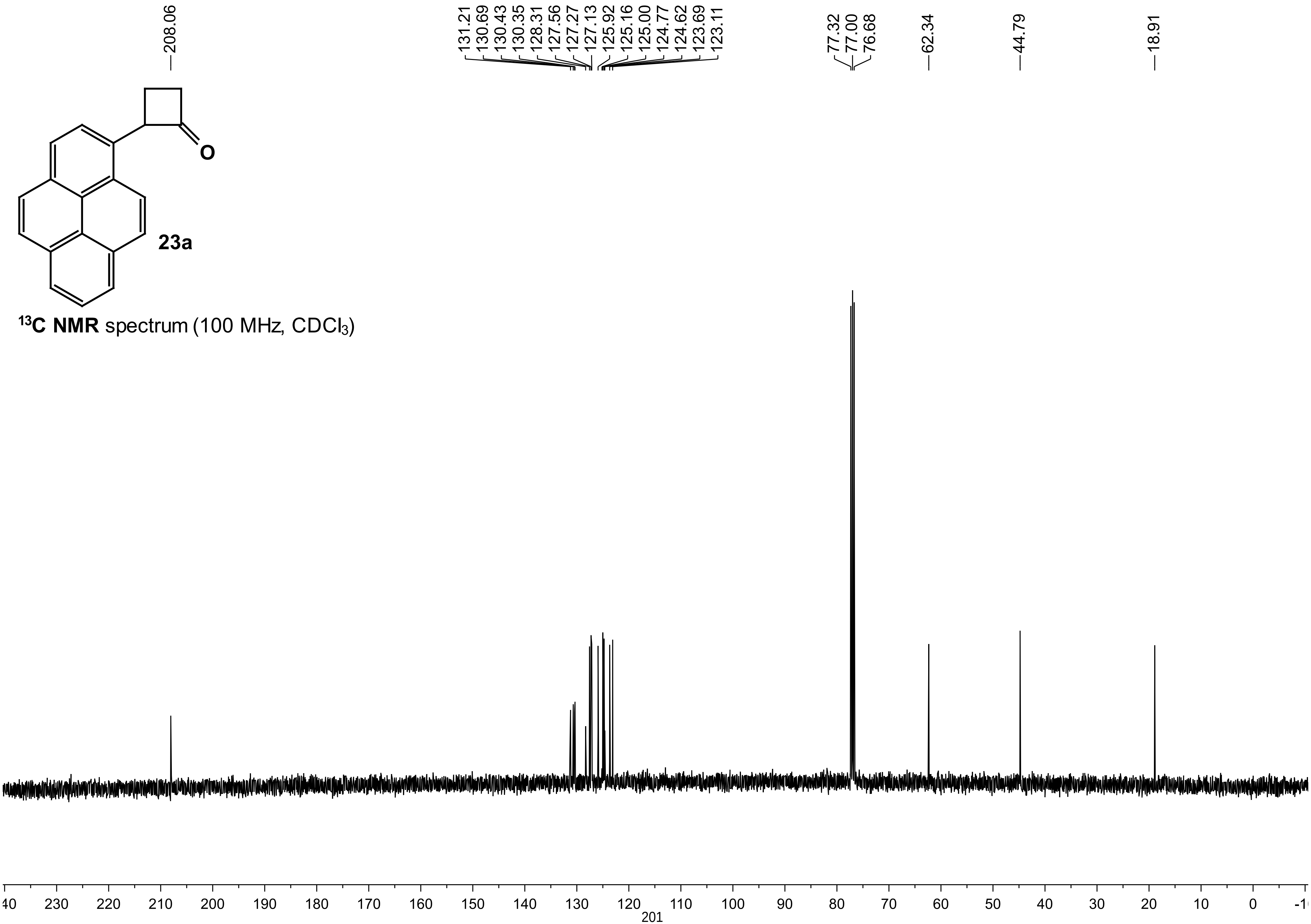
208.06
131.21
130.69
130.43
130.35
128.31
127.56
127.27
127.13
125.92
125.16
125.00
124.77
124.62
123.69
123.11
77.32
77.00
76.68
62.34
44.79
18.91
O
23a
^{13}C NMR spectrum (100 MHz, $CDCl_3$)
230
220
210
200
190
180
170
160
150
140
130
120
110
100
90
80
70
60
50
40
30
20
10
0

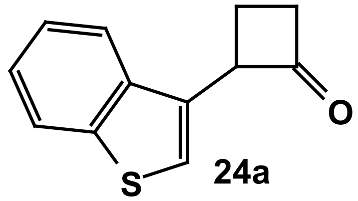

24a

[1]**H NMR** spectrum (600 MHz, $CDCl_3$)

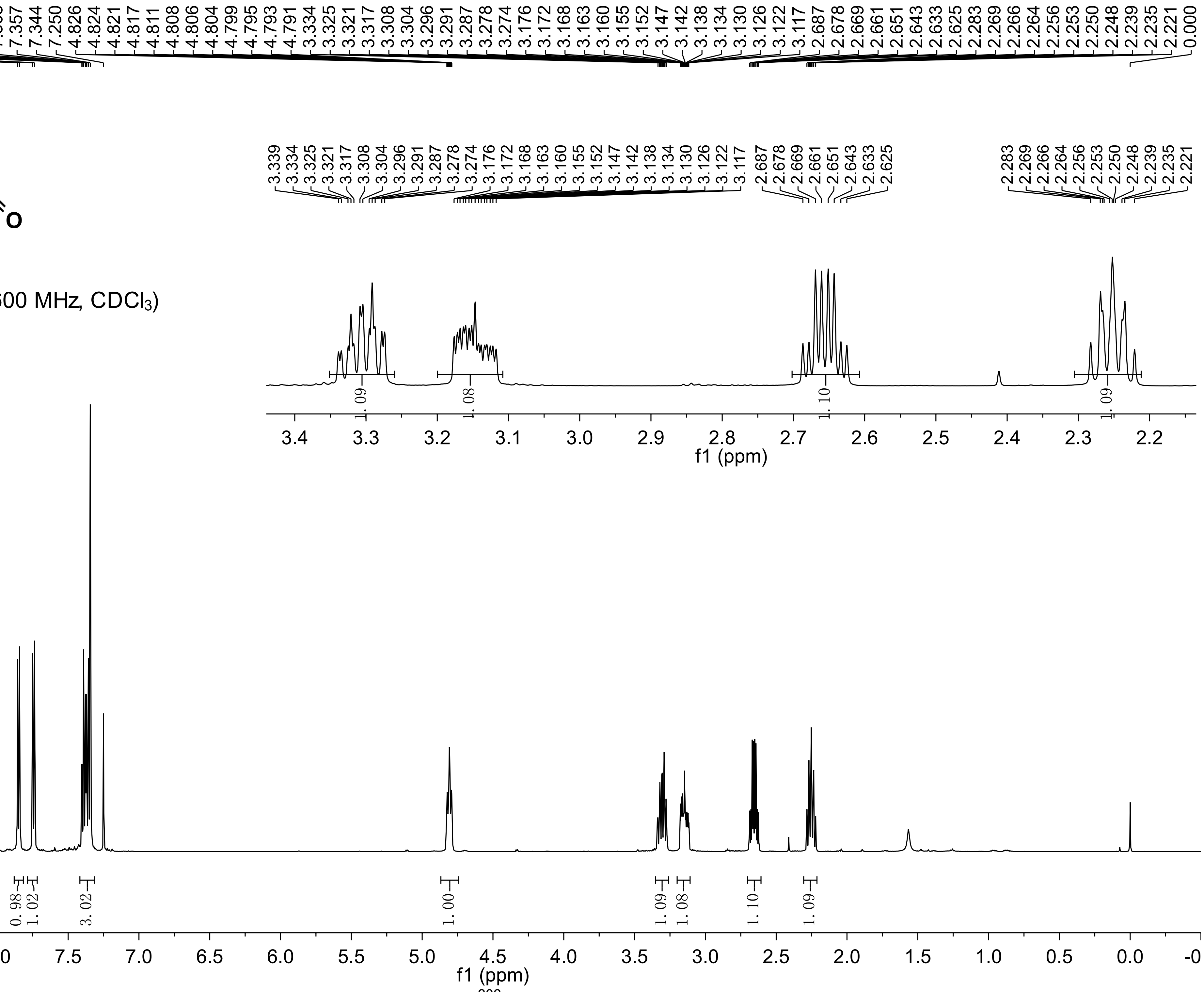

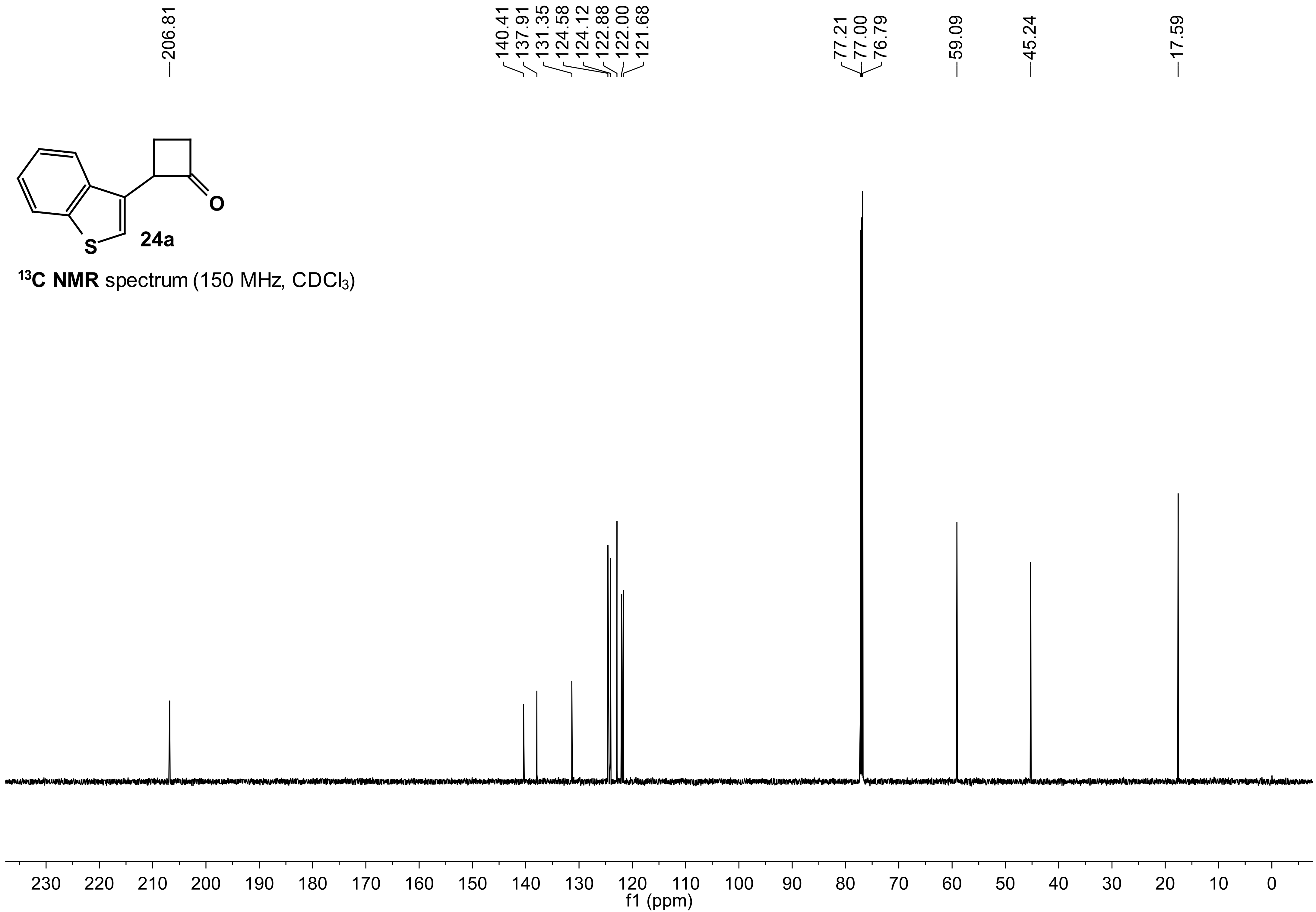

24a

^{13}C NMR spectrum (150 MHz, $CDCl_3$)

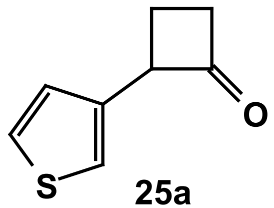

25a

^{1}H NMR spectrum (600 MHz, $CDCl_3$)

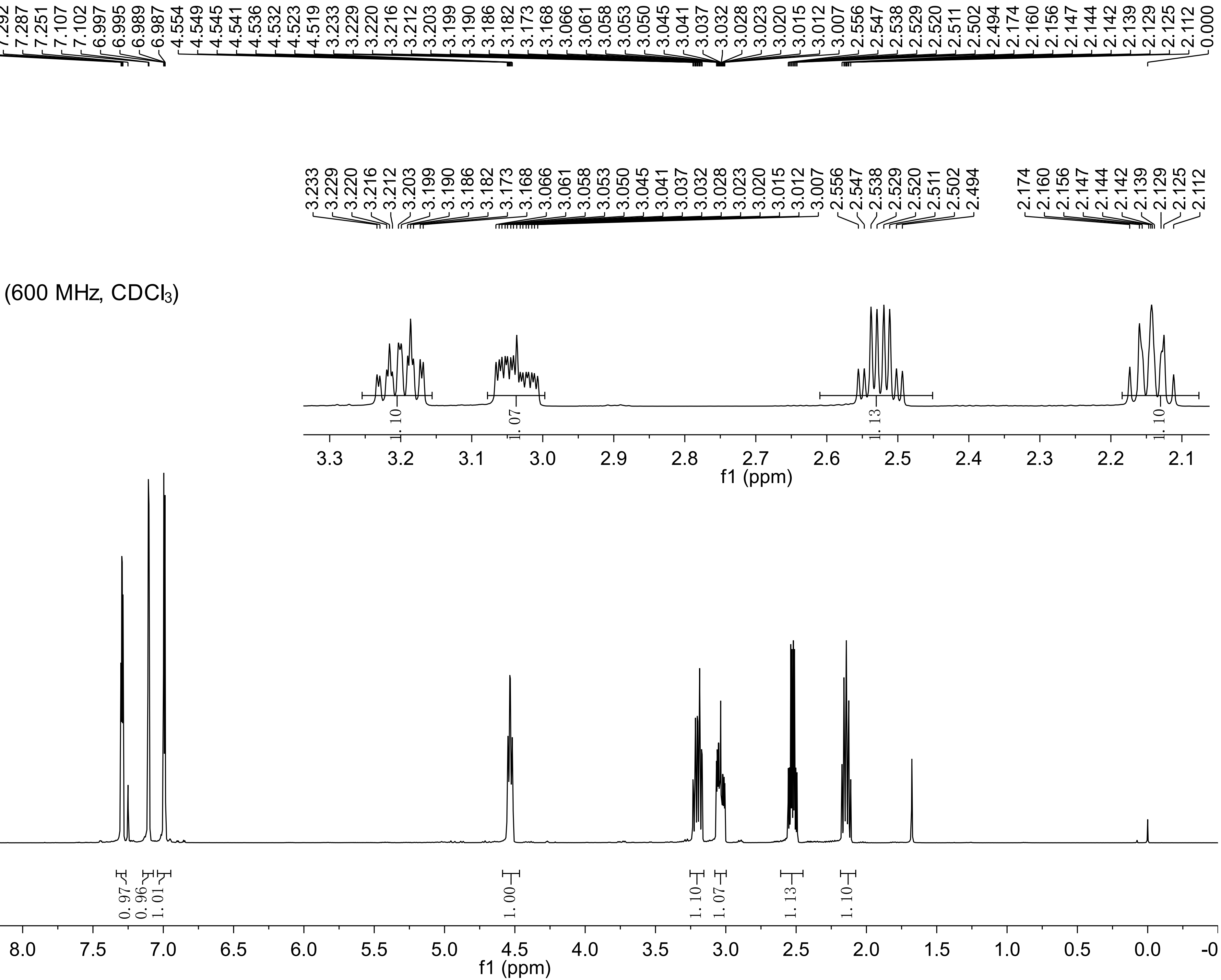

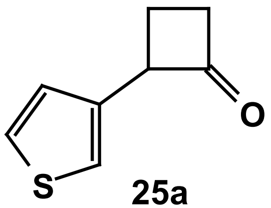

25a

^{13}C NMR spectrum (150 MHz, $CDCl_3$)

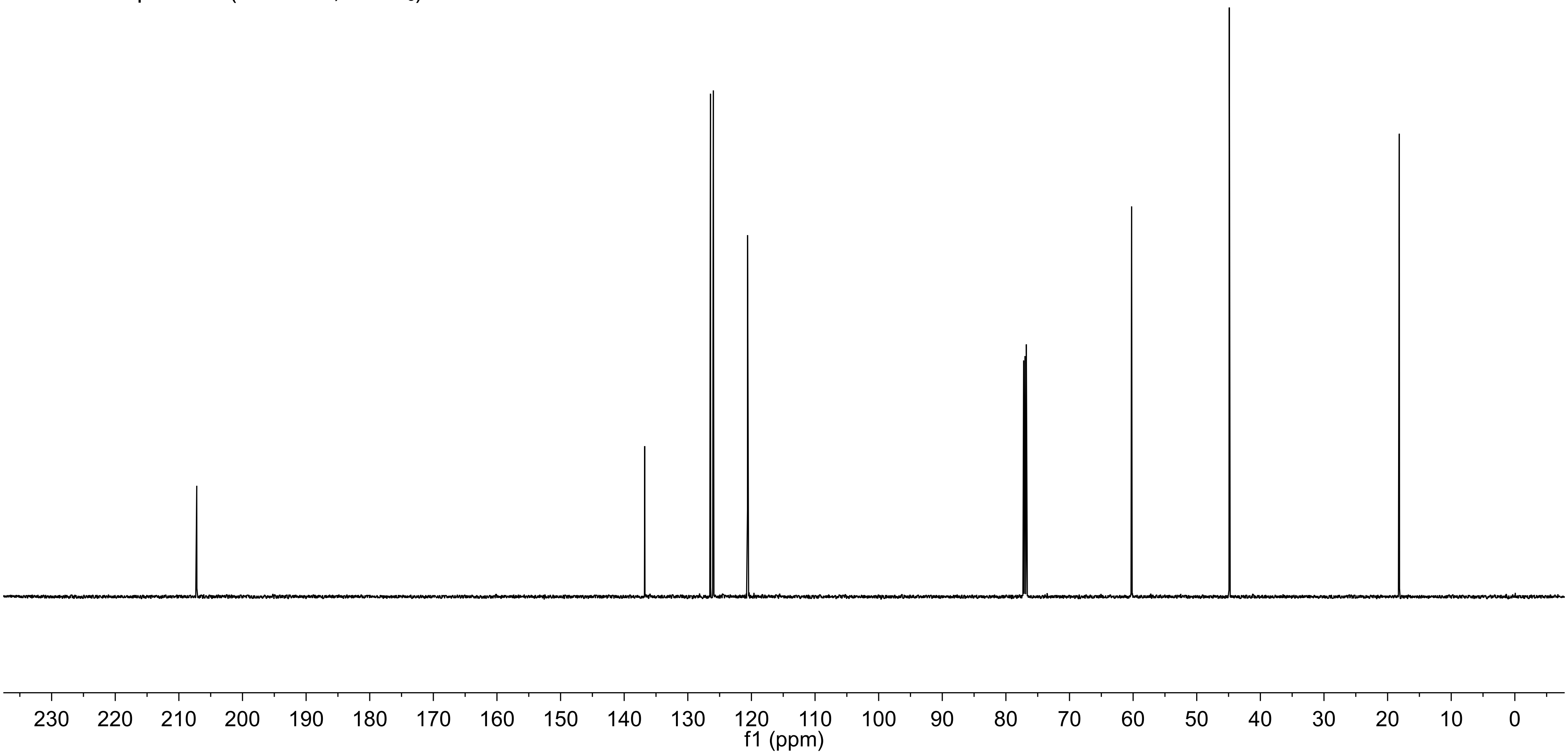

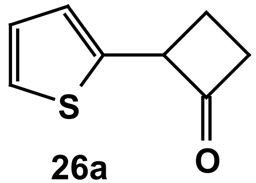

26a

^{1}H NMR spectrum (600 MHz, $CDCl_3$)

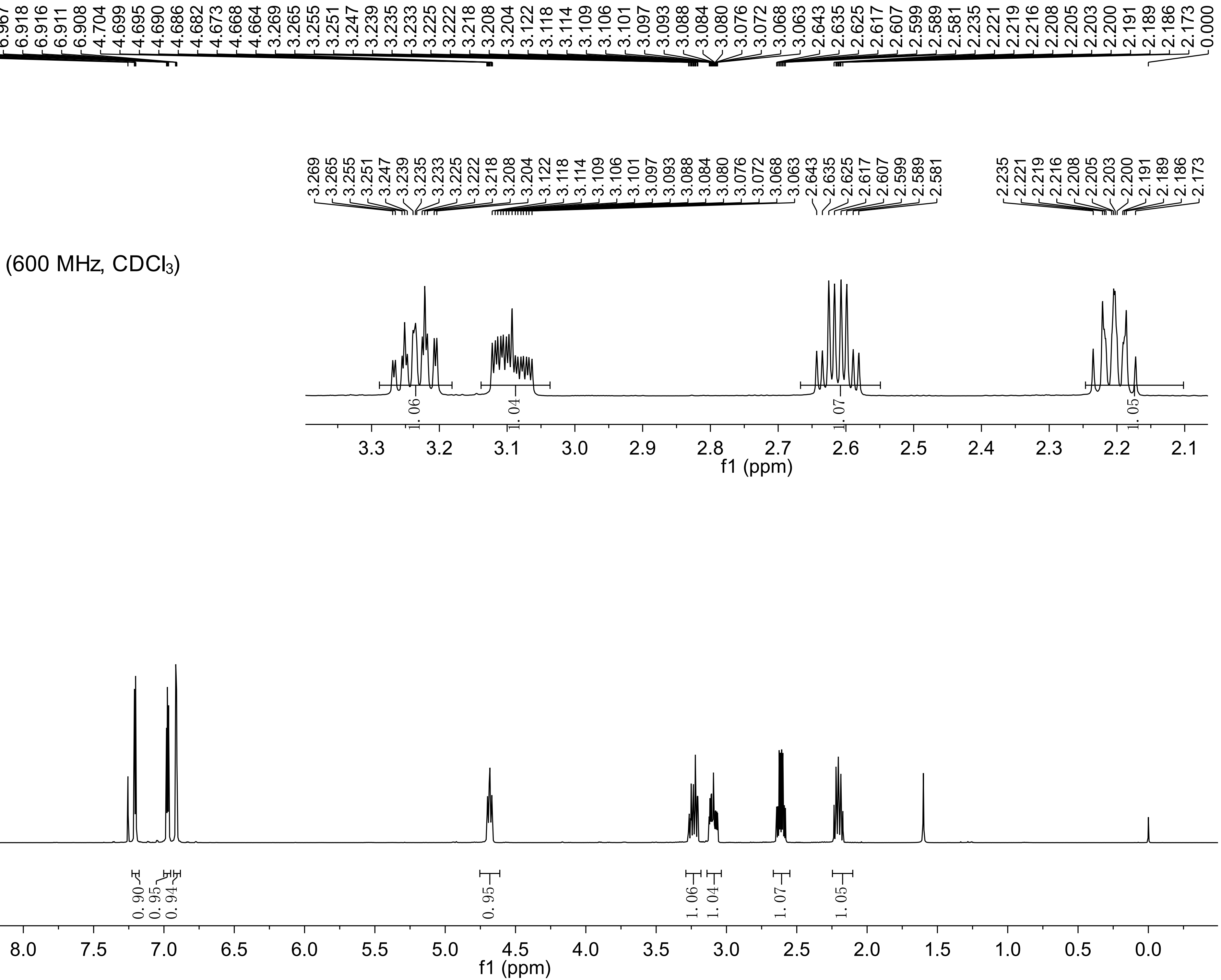

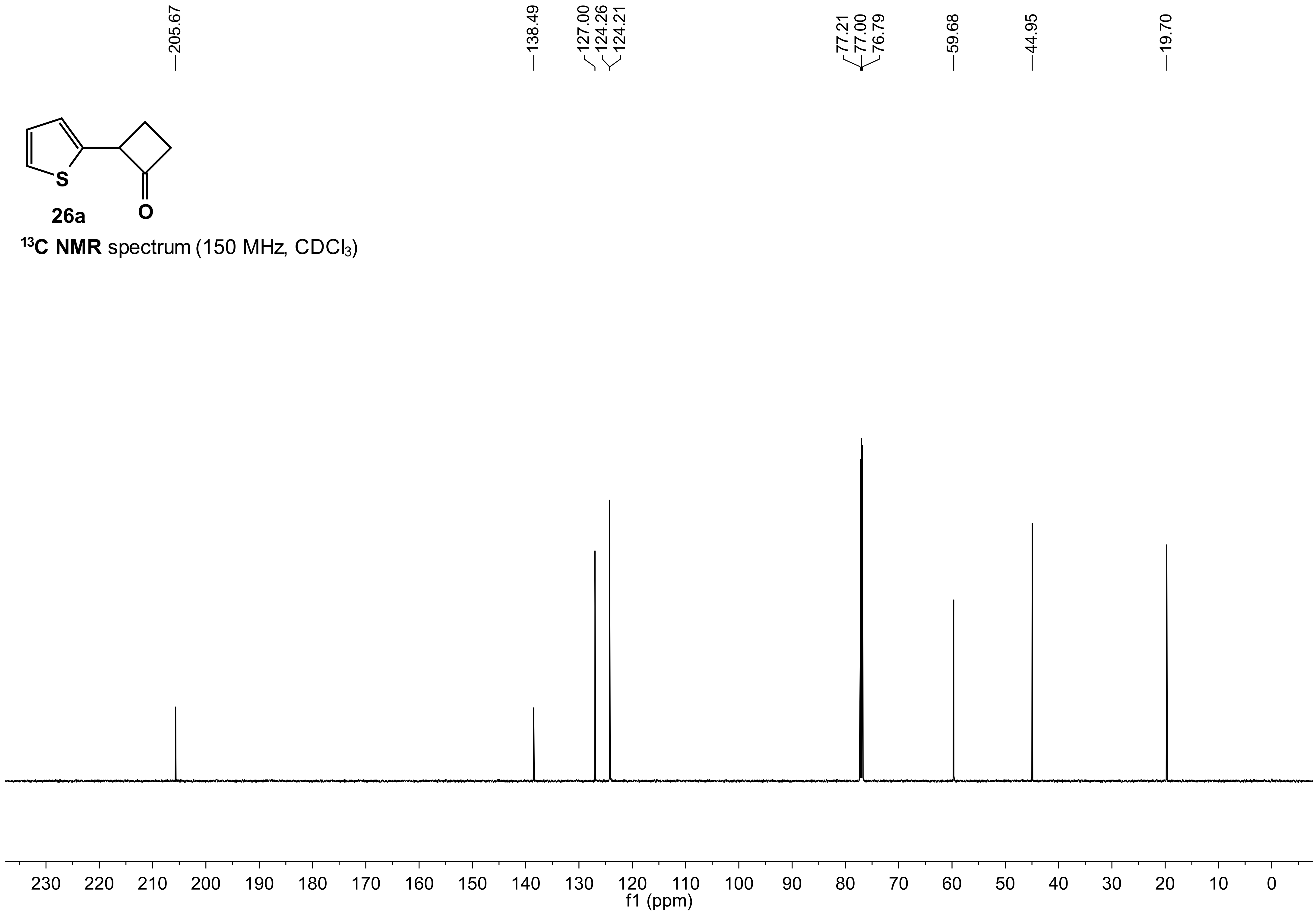
205.67
138.49
127.00
124.26
124.21
77.21
77.00
76.79
59.68
44.95
19.70
S
O
26a
^{13}C NMR spectrum (150 MHz, $CDCl_3$)
230 220 210 200 190 180 170 160 150 140 130 120 110 100 90 80 70 60 50 40 30 20 10 0
f1 (ppm)

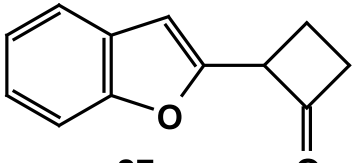

27a

^{1}H NMR spectrum (600 MHz, $CDCl_3$)

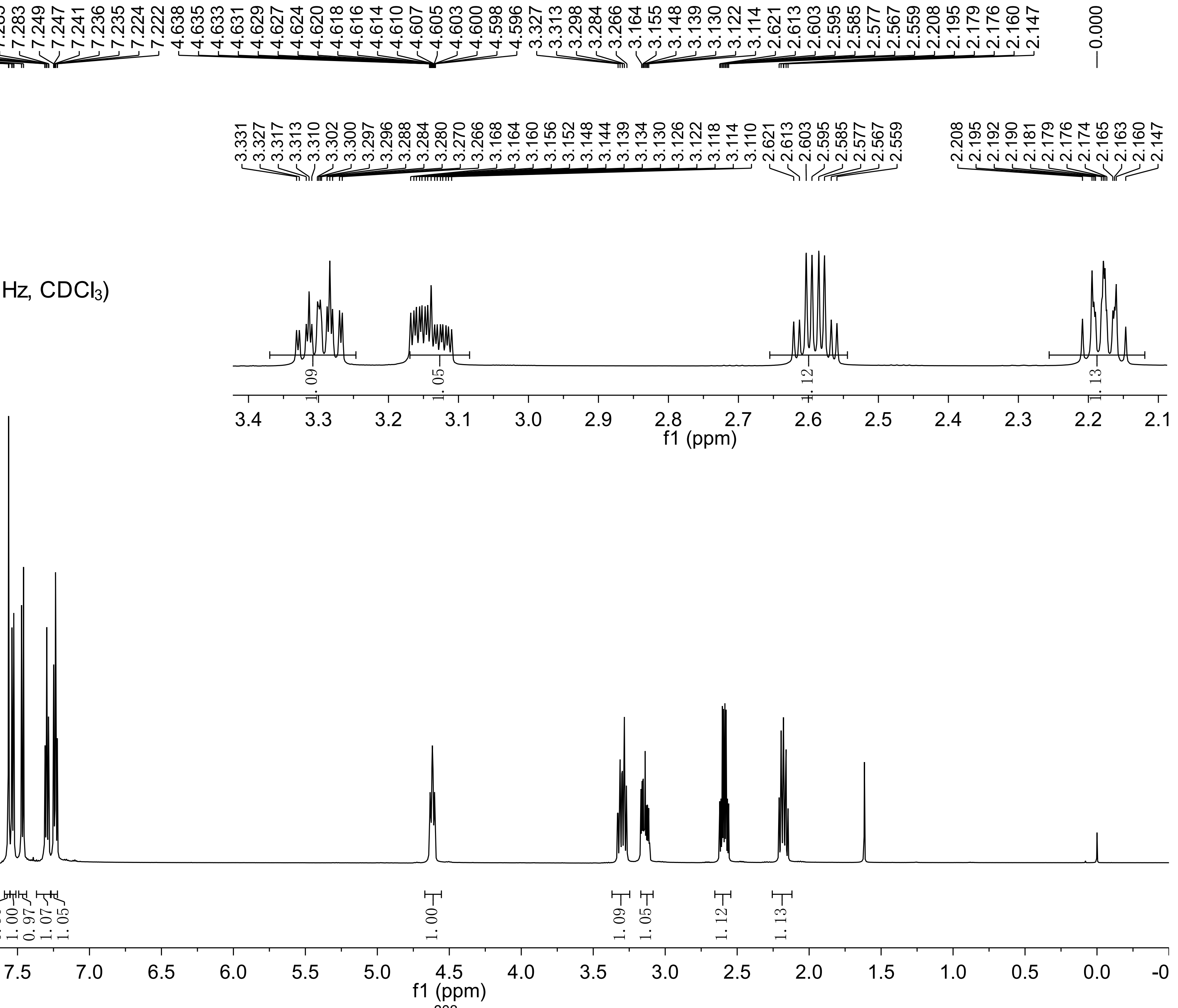

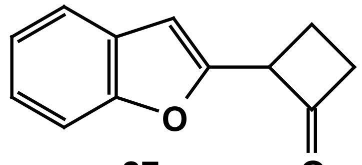

27a

^{13}C NMR spectrum (150 MHz, $CDCl_3$)

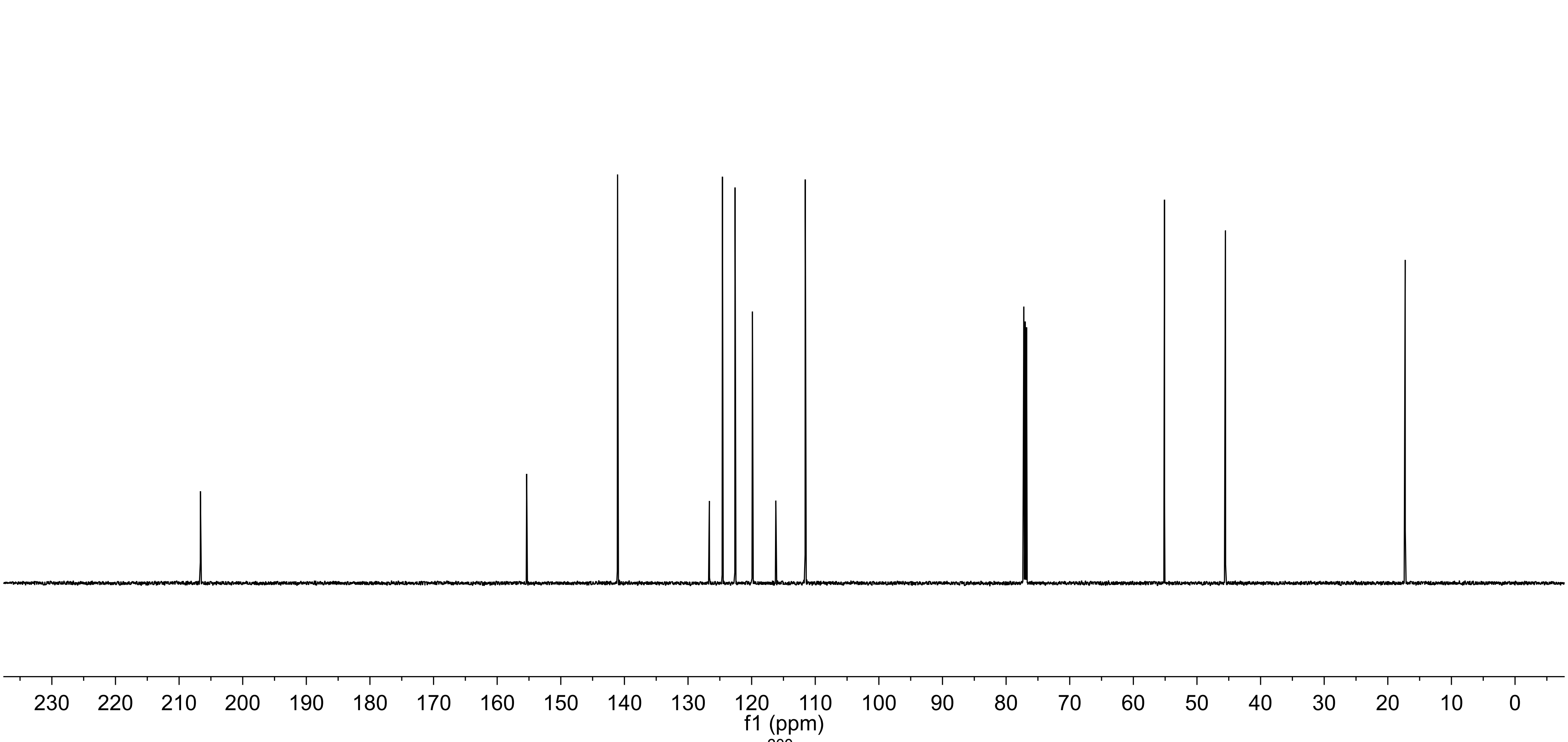

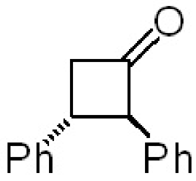

28a

^{1}H NMR spectrum (400 MHz, $CDCl_3$)

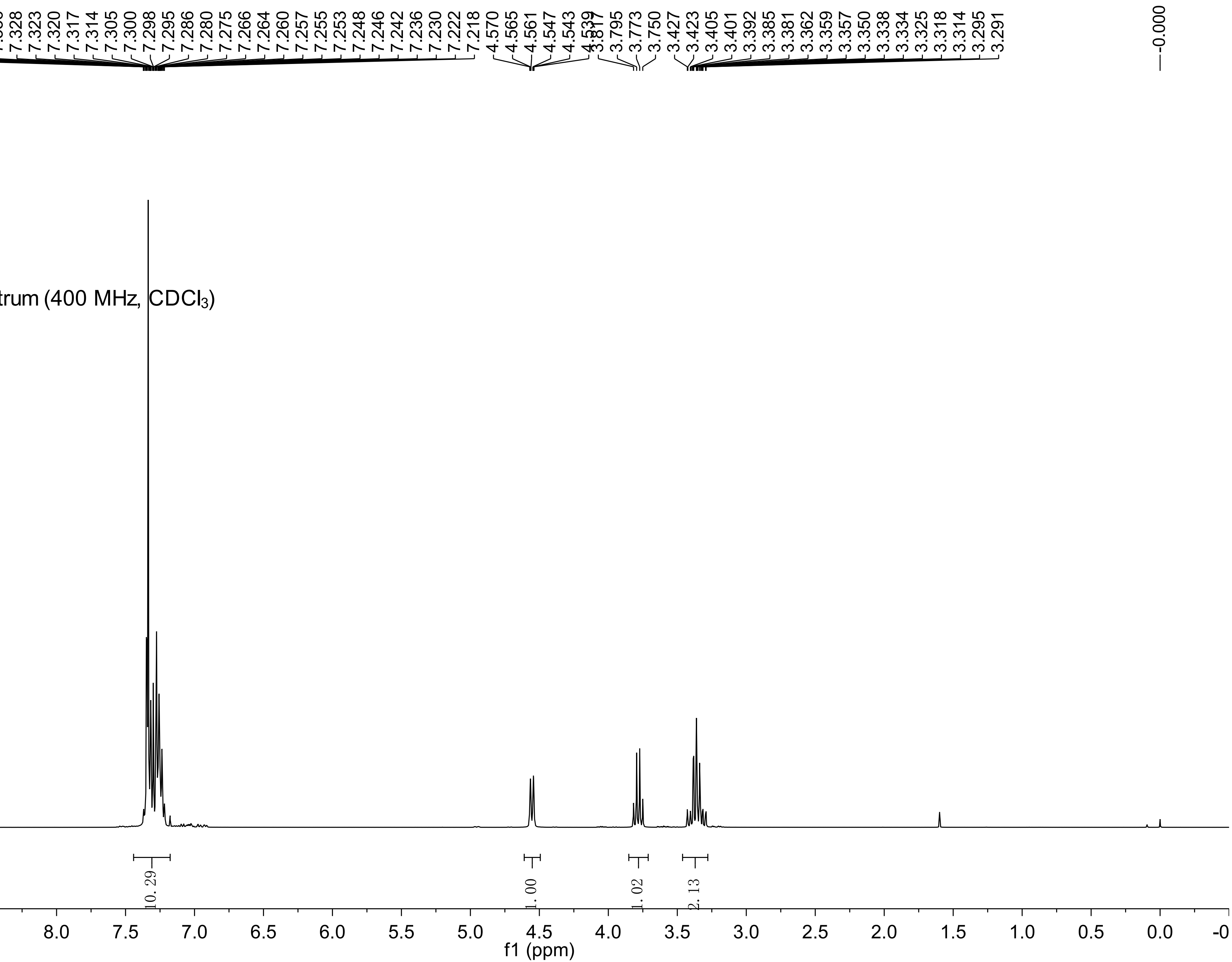

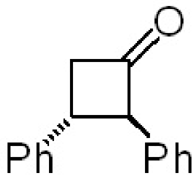

28a

^{13}**C NMR** spectrum (100 MHz, $CDCl_3$)

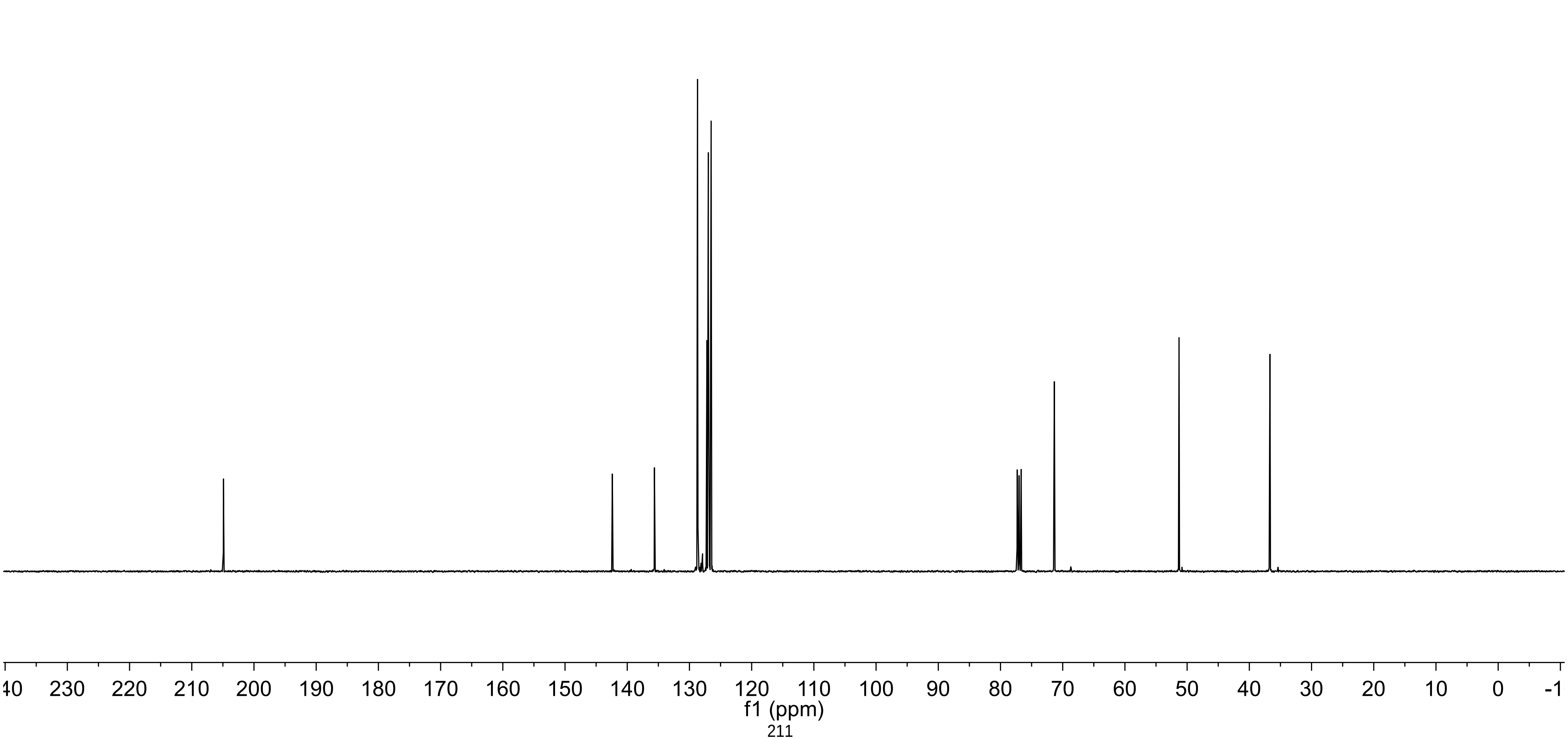

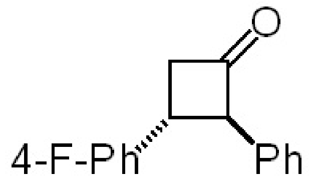

29a

^{1}H NMR spectrum (400 MHz, $CDCl_3$)

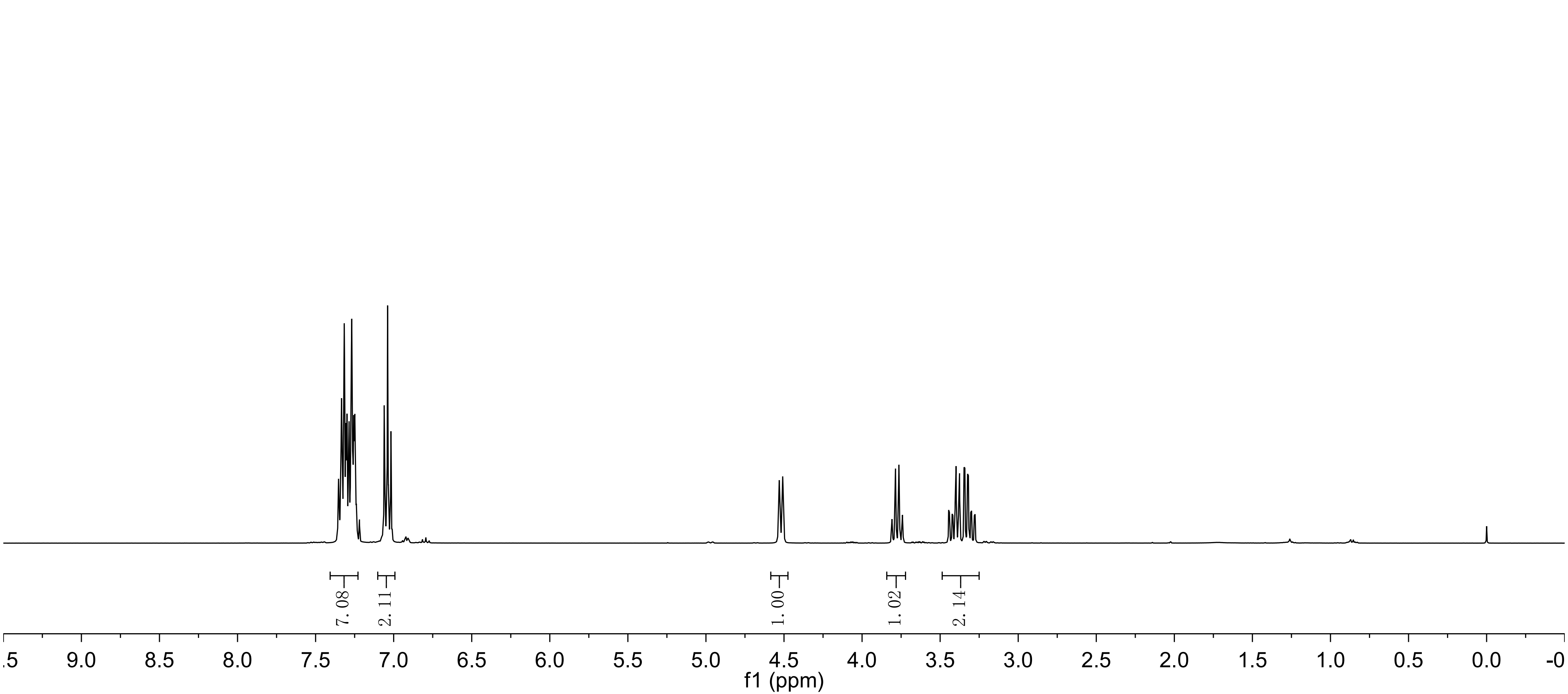

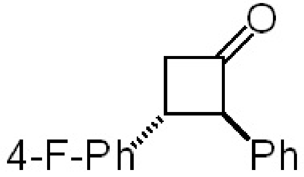

29a

^{13}C NMR spectrum (100 MHz, $CDCl_3$)

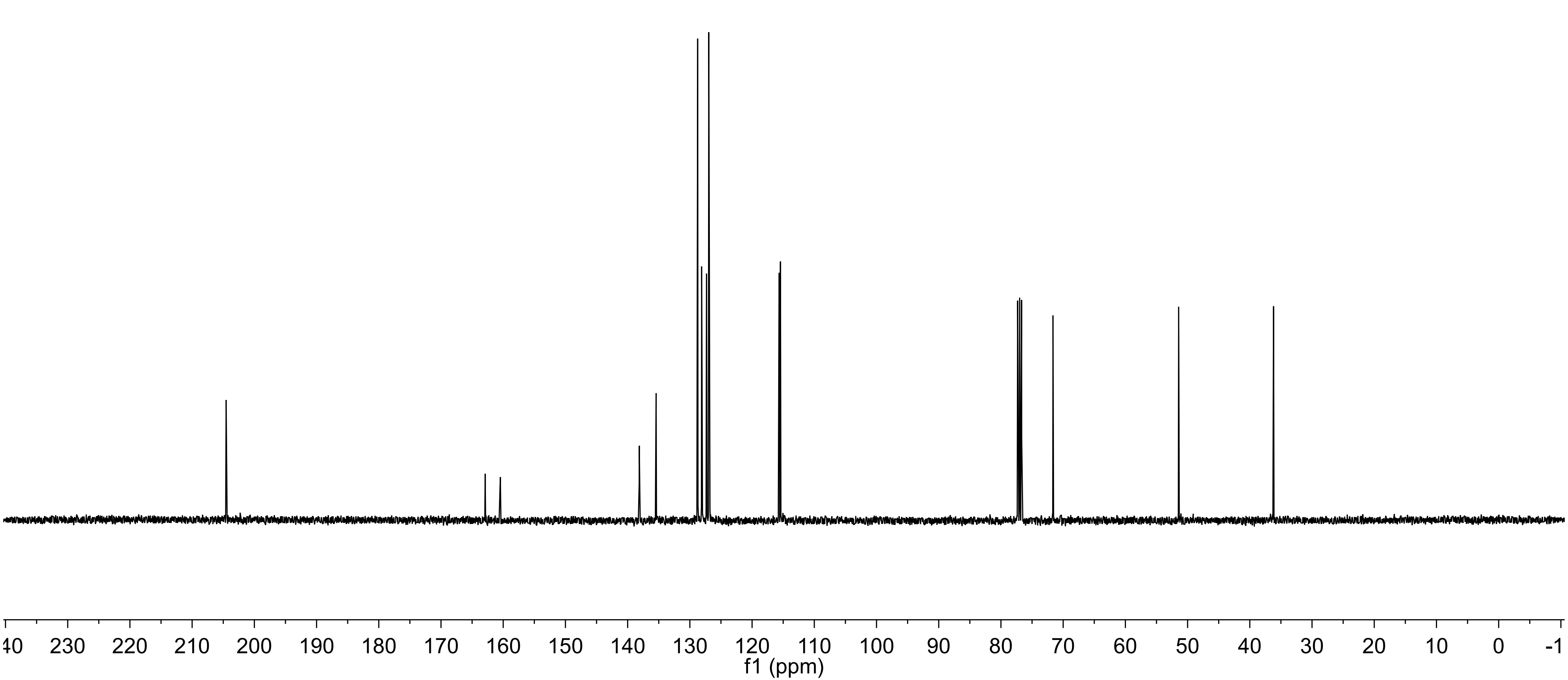

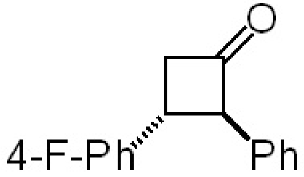

29a

^{19}F NMR spectrum (376 MHz, $CDCl_3$)

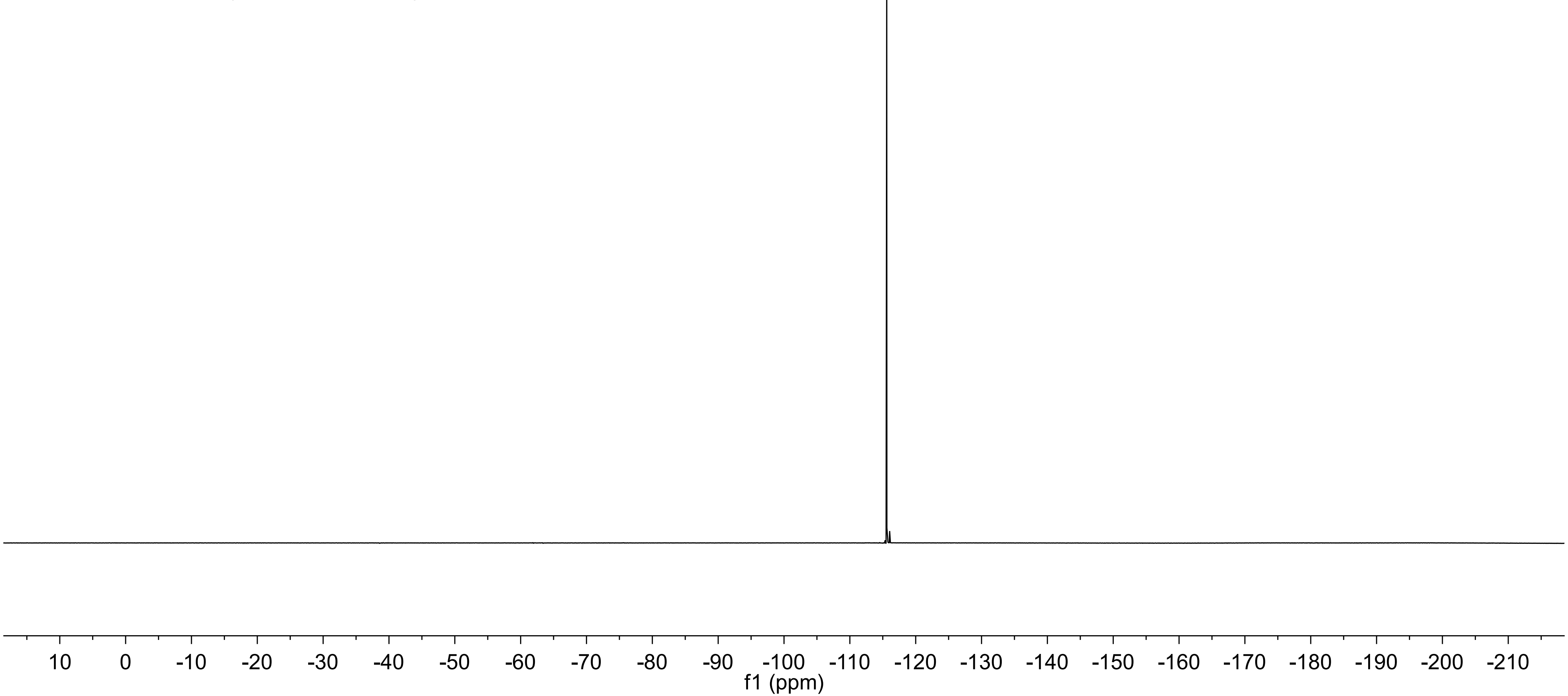

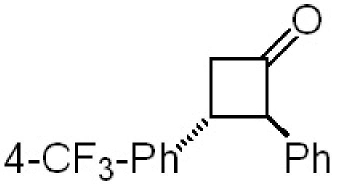

30a

^{1}H NMR spectrum (400 MHz, $CDCl_3$)

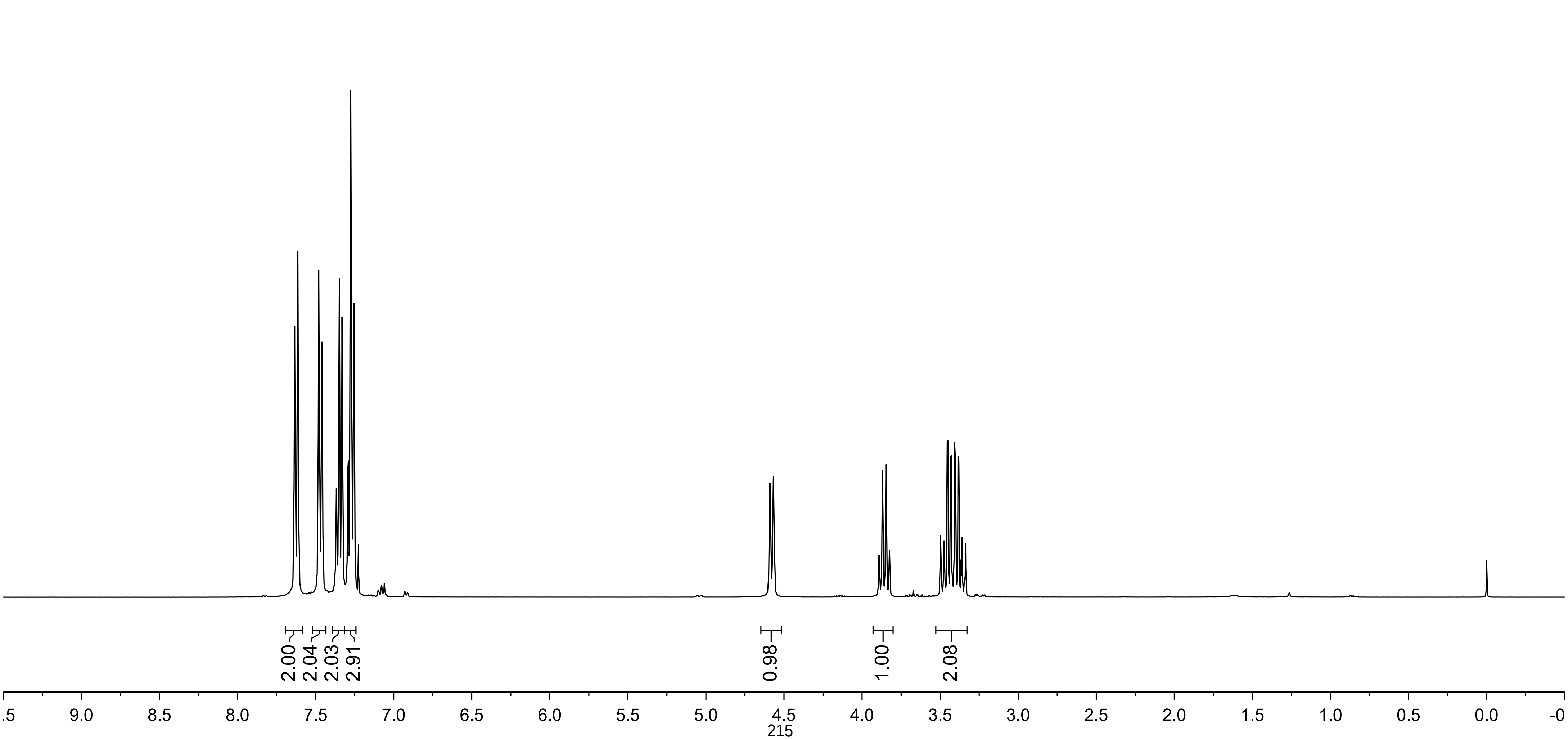

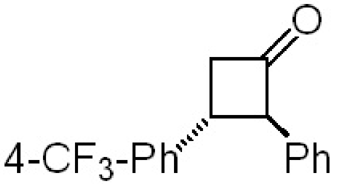

30a

^{13}**C NMR** spectrum (100 MHz, $CDCl_3$)

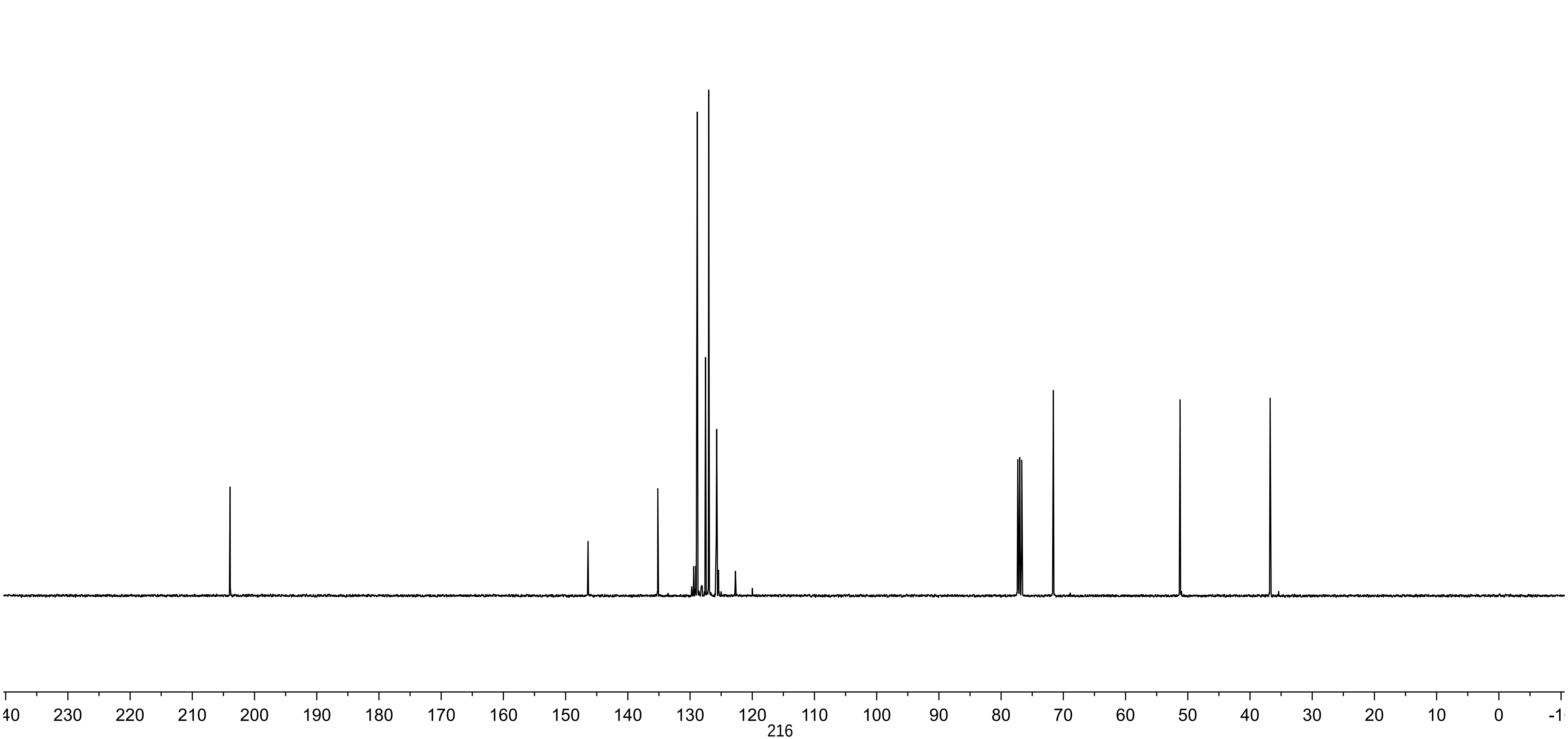

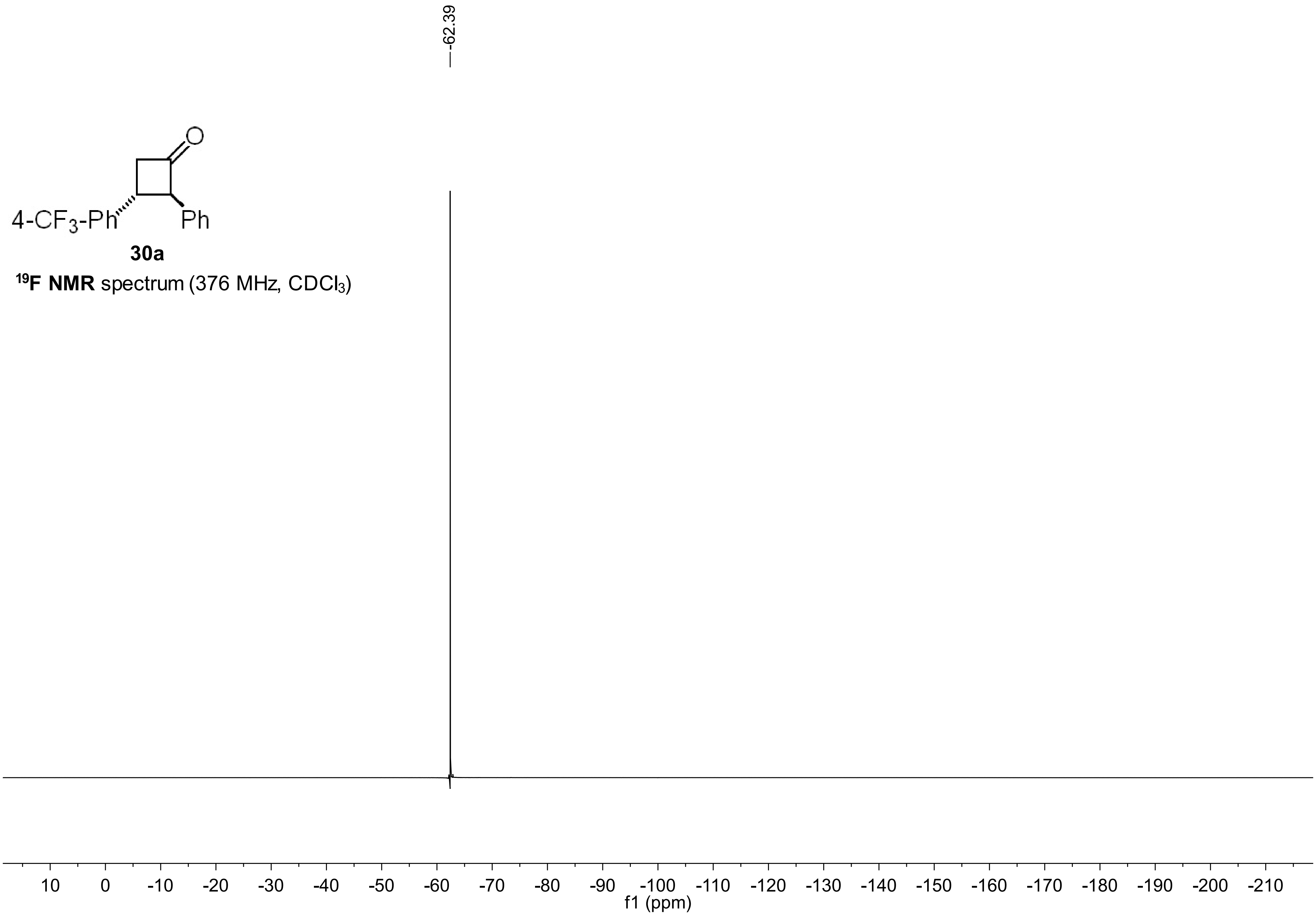

30a

[19]**F NMR** spectrum (376 MHz, $CDCl_3$)

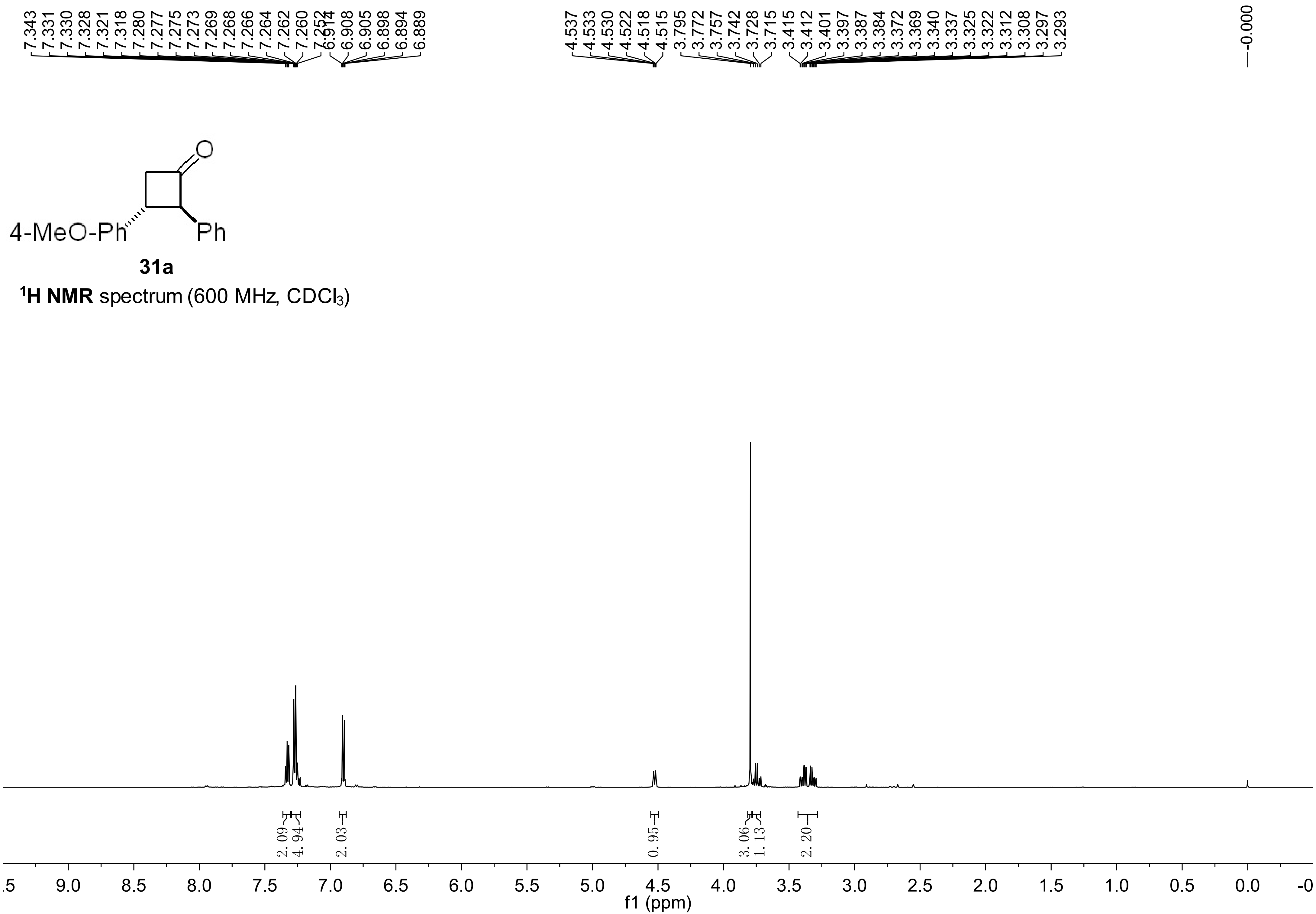

31a

^{1}H NMR spectrum (600 MHz, $CDCl_3$)

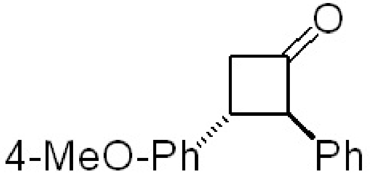

31a

^{13}C NMR spectrum (150 MHz, $CDCl_3$)

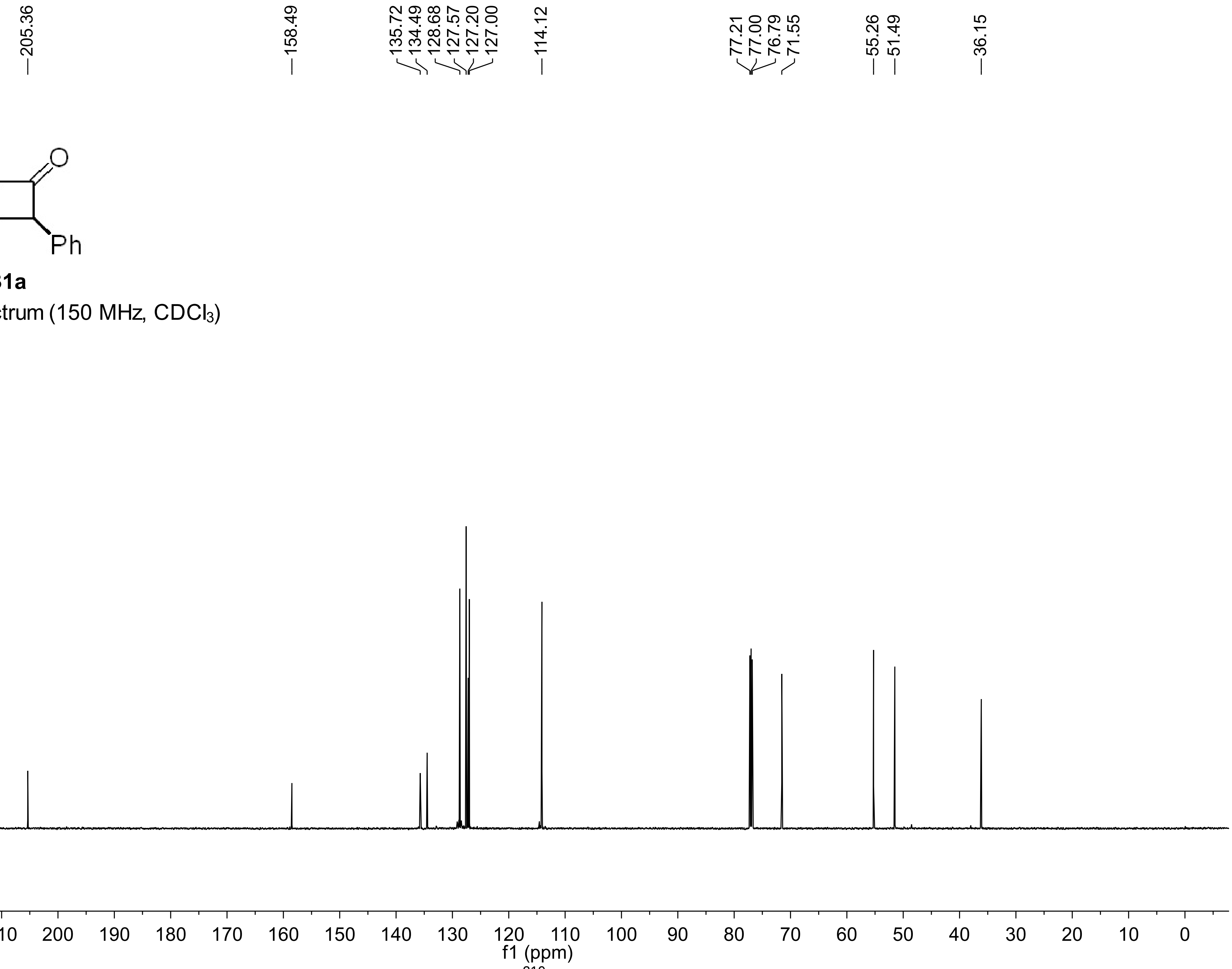

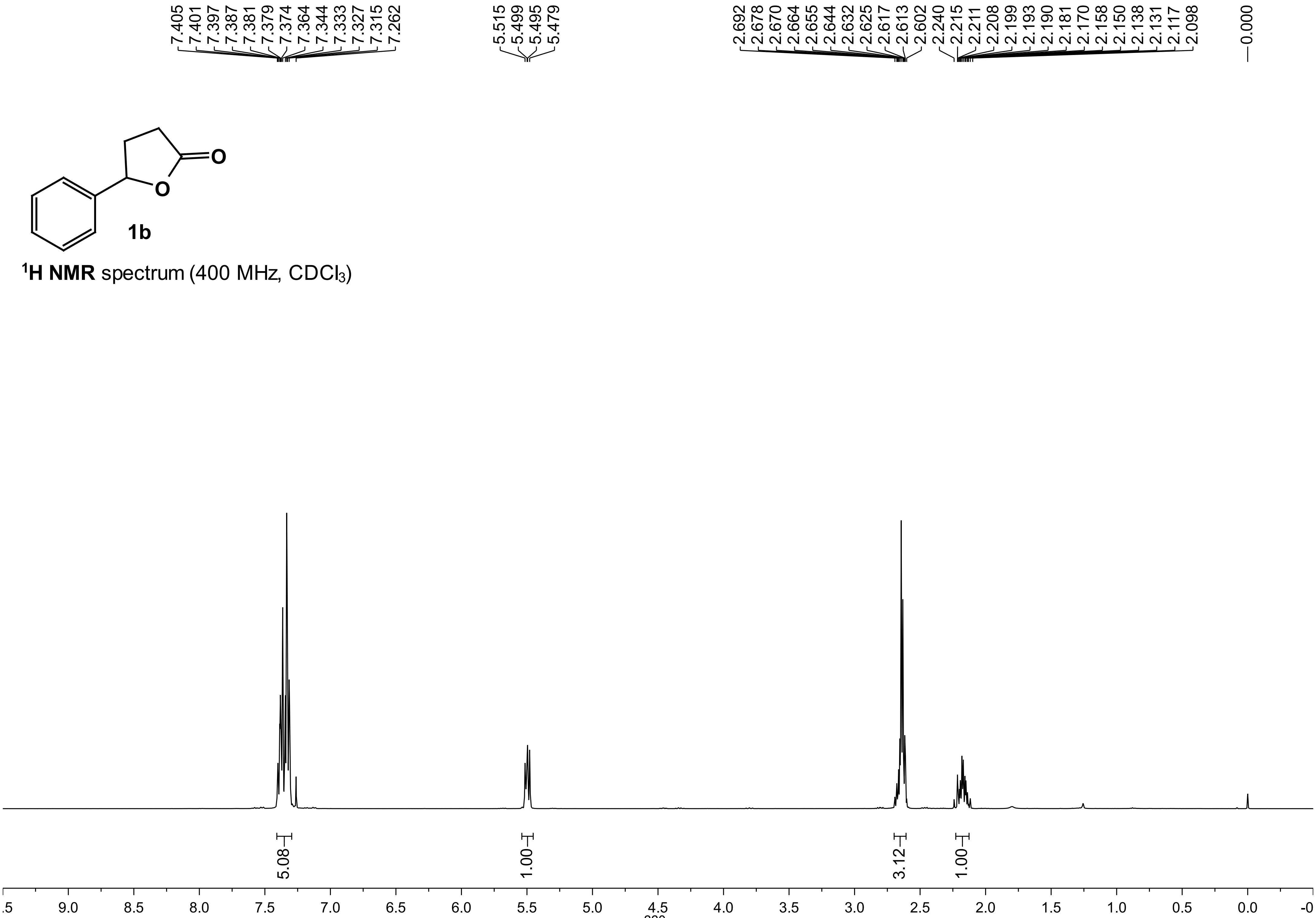

1b

^{1}H NMR spectrum (400 MHz, $CDCl_3$)

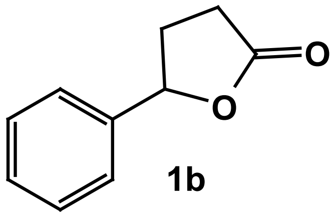

1b

^{13}C NMR spectrum (100 MHz, $CDCl_3$)

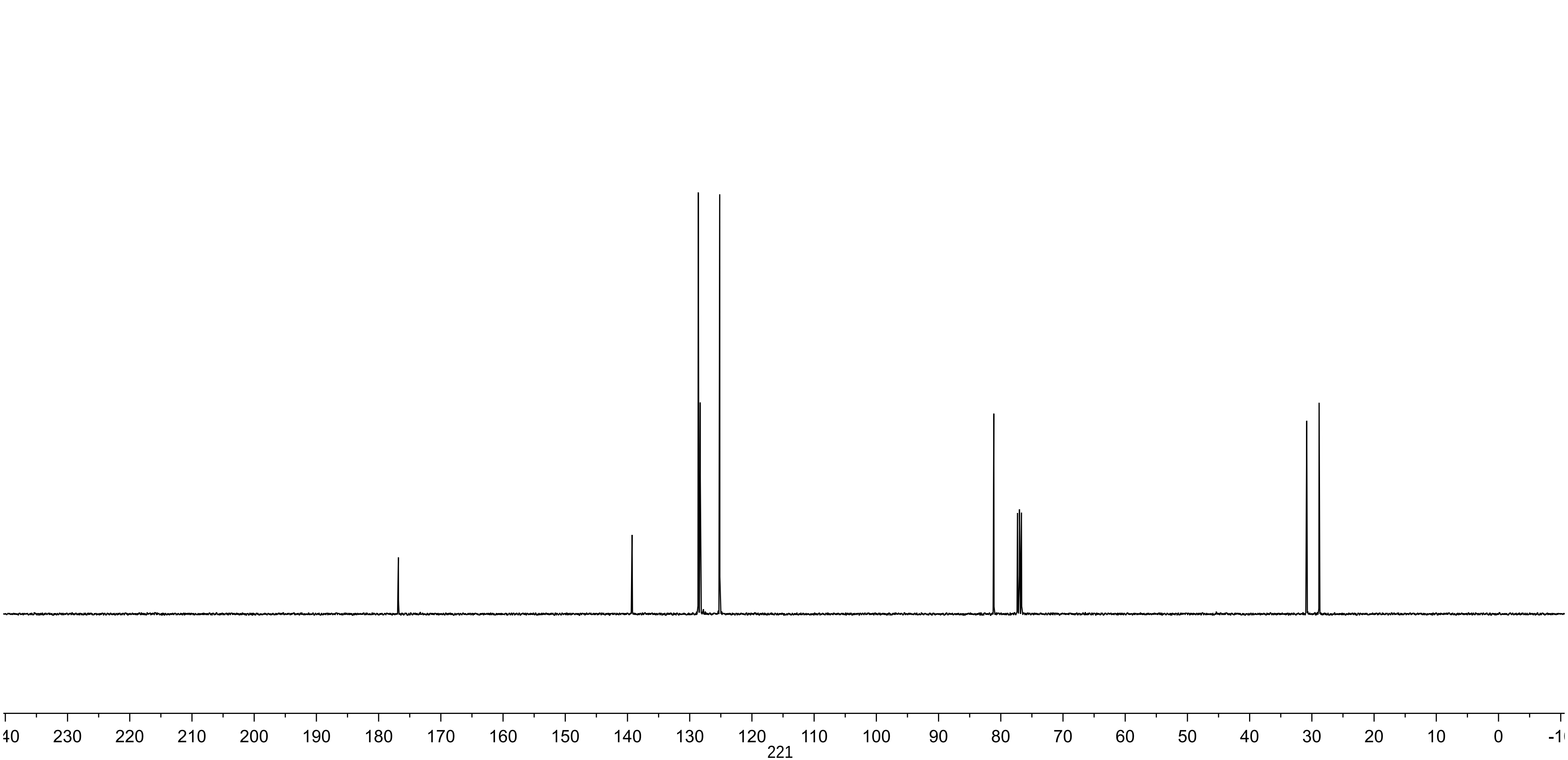

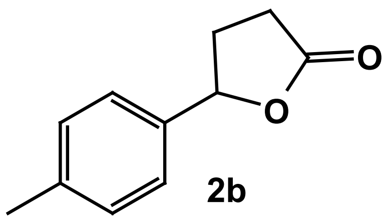

2b

^{1}H NMR spectrum (600 MHz, $CDCl_3$)

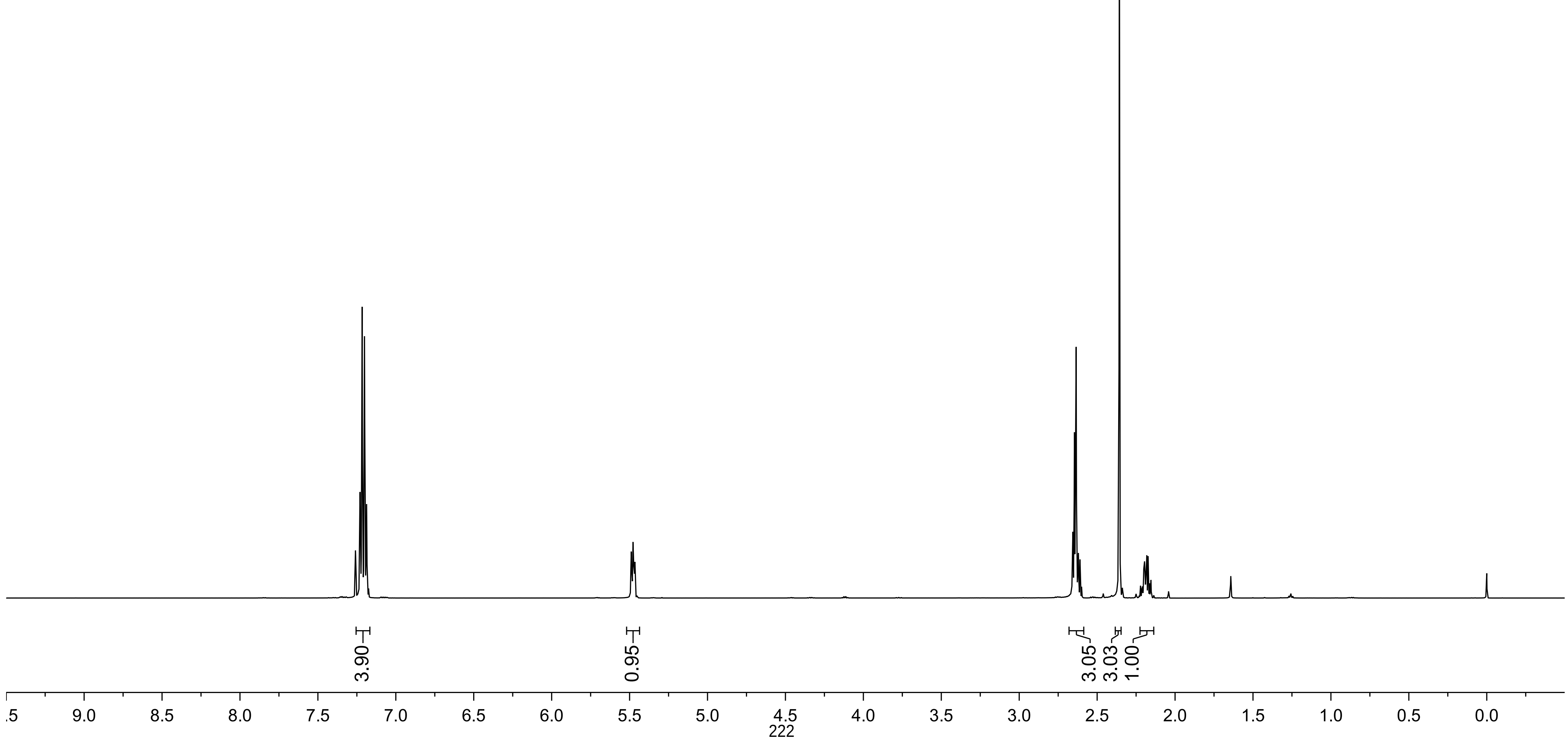

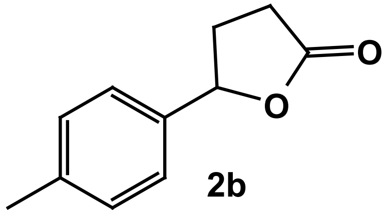

2b

^{13}C NMR spectrum (150 MHz, $CDCl_3$)

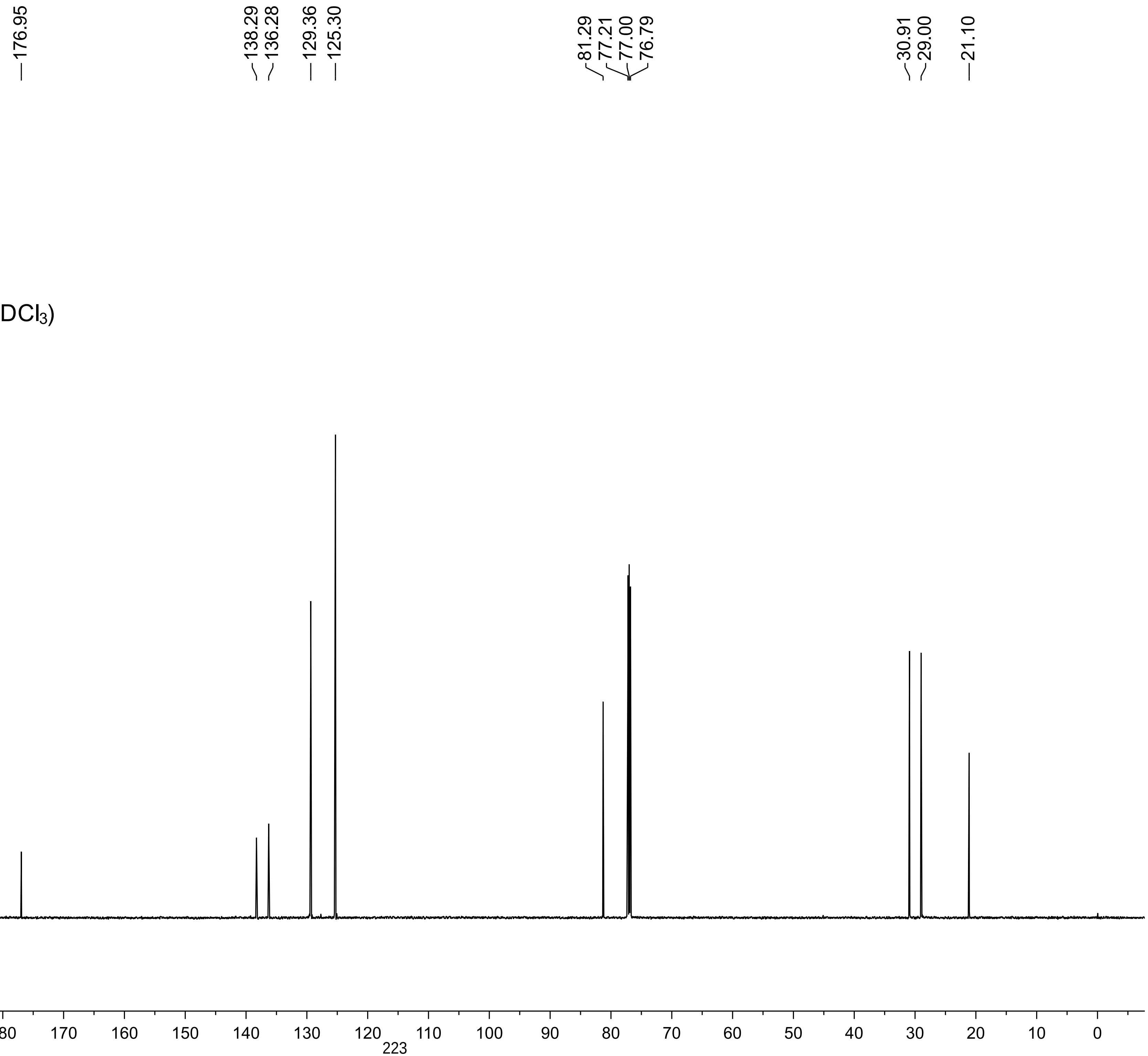

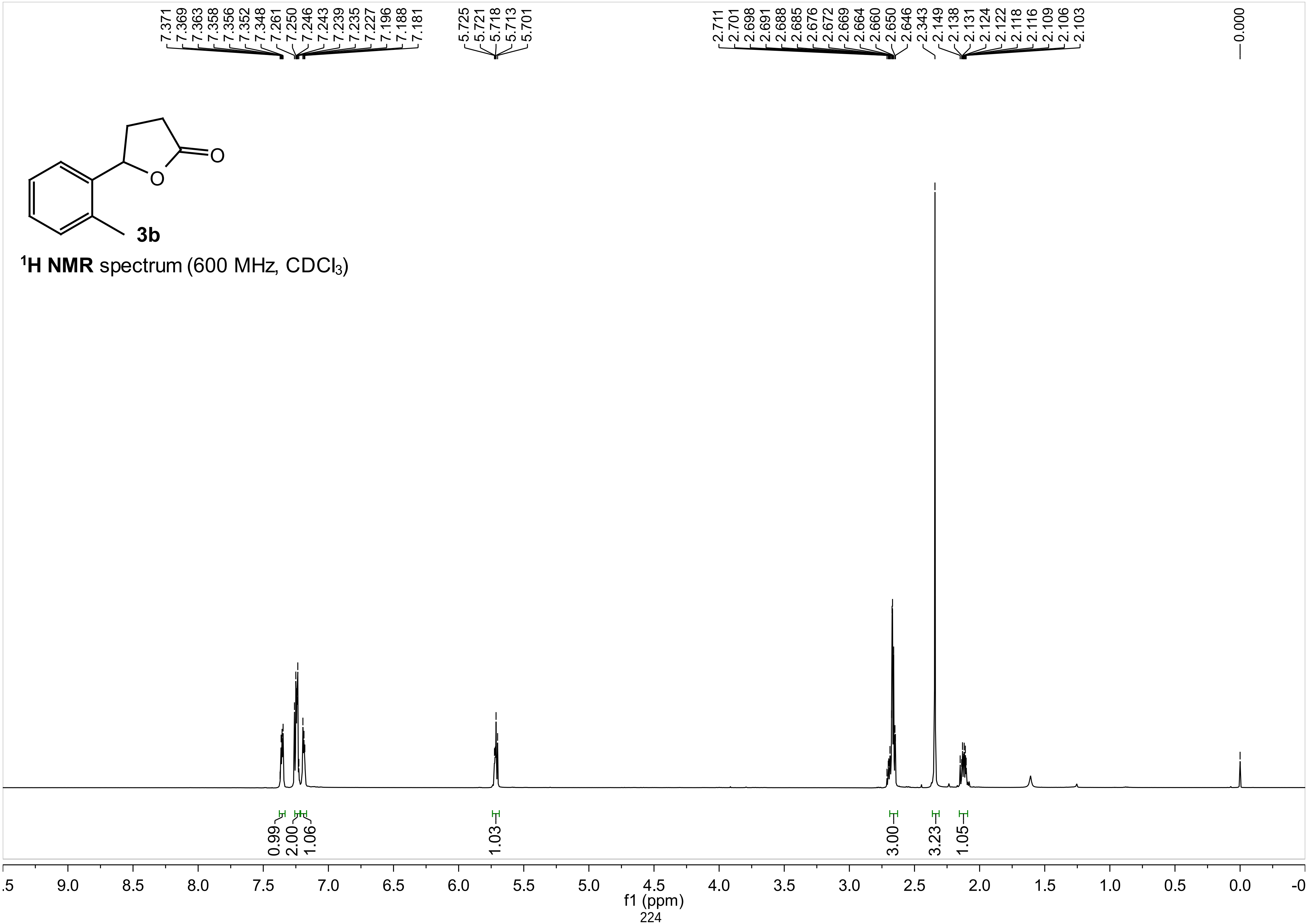
7.371 7.369 7.363 7.358 7.356 7.352 7.348 7.261 7.250 7.246 7.243 7.239 7.235 7.227 7.196 7.188 7.181
5.725 5.721 5.718 5.713 5.701
2.711 2.701 2.698 2.691 2.688 2.685 2.676 2.672 2.669 2.664 2.660 2.650 2.646 2.343 2.149 2.138 2.131 2.124 2.122 2.118 2.116 2.109 2.106 2.103
0.000
O
O
3b
^{1}H NMR spectrum (600 MHz, $CDCl_3$)
0.99
2.00
1.06
1.03
3.00
3.23
1.05
9.0
8.5
8.0
7.5
7.0
6.5
6.0
5.5
5.0
4.5
4.0
3.5
3.0
2.5
2.0
1.5
1.0
0.5
0.0
f1 (ppm)

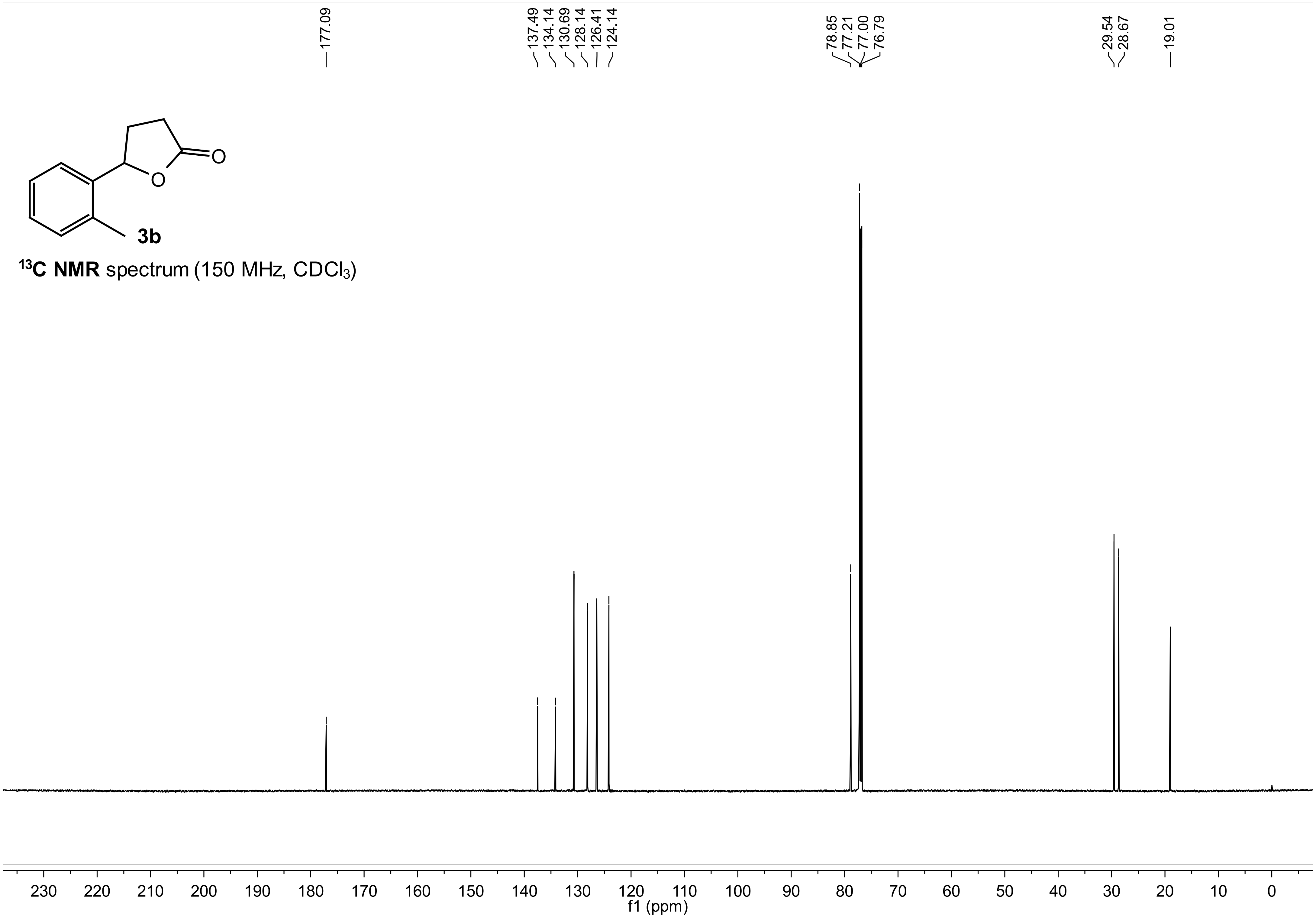
177.09
137.49
134.14
130.69
128.14
126.41
124.14
78.85
77.21
77.00
76.79
29.54
28.67
19.01
O
O
3b
^{13}C NMR spectrum (150 MHz, CDCl$_3$)
230 220 210 200 190 180 170 160 150 140 130 120 110 100 90 80 70 60 50 40 30 20 10 0
f1 (ppm)

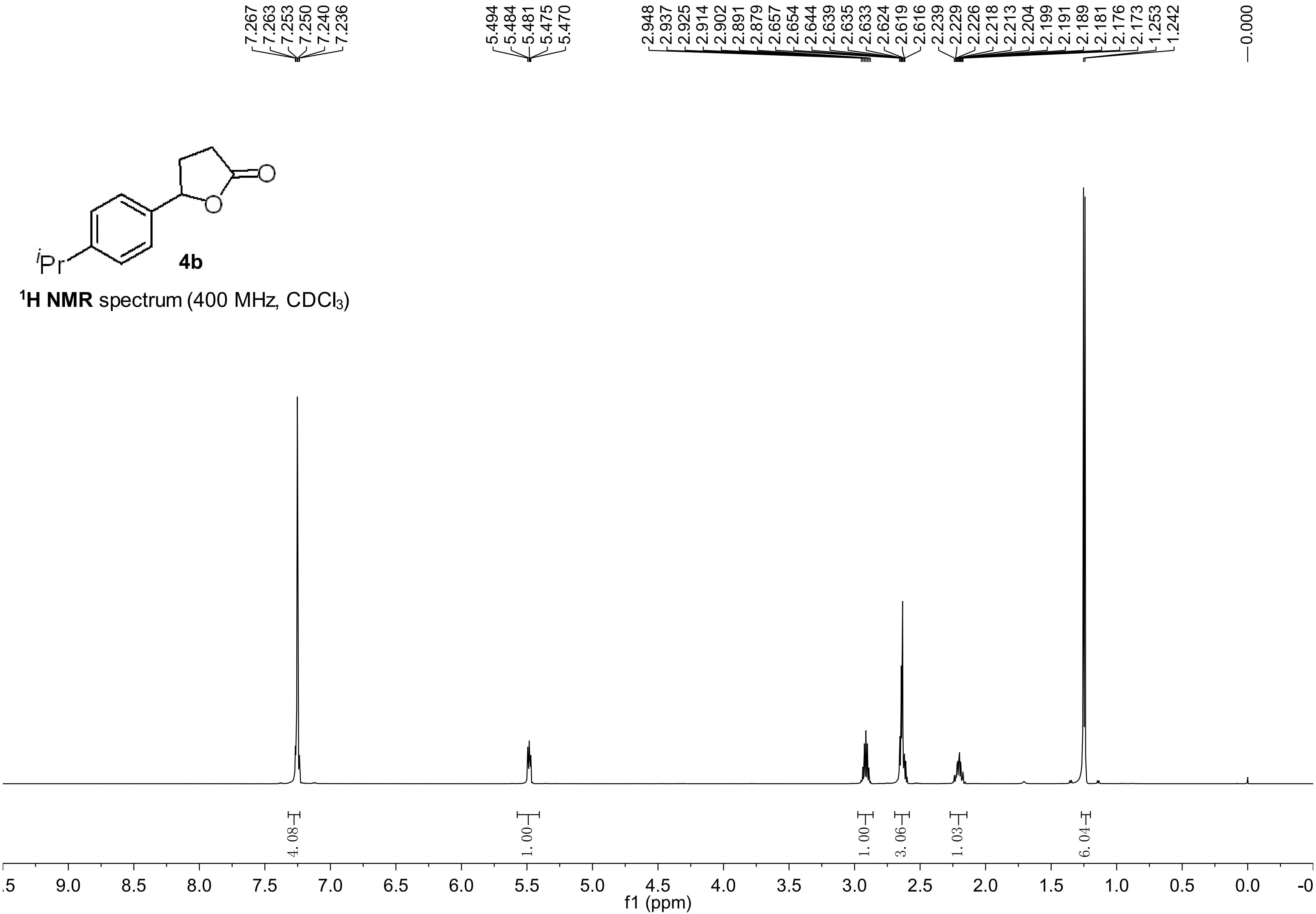

^{1}H NMR spectrum (400 MHz, $CDCl_3$)

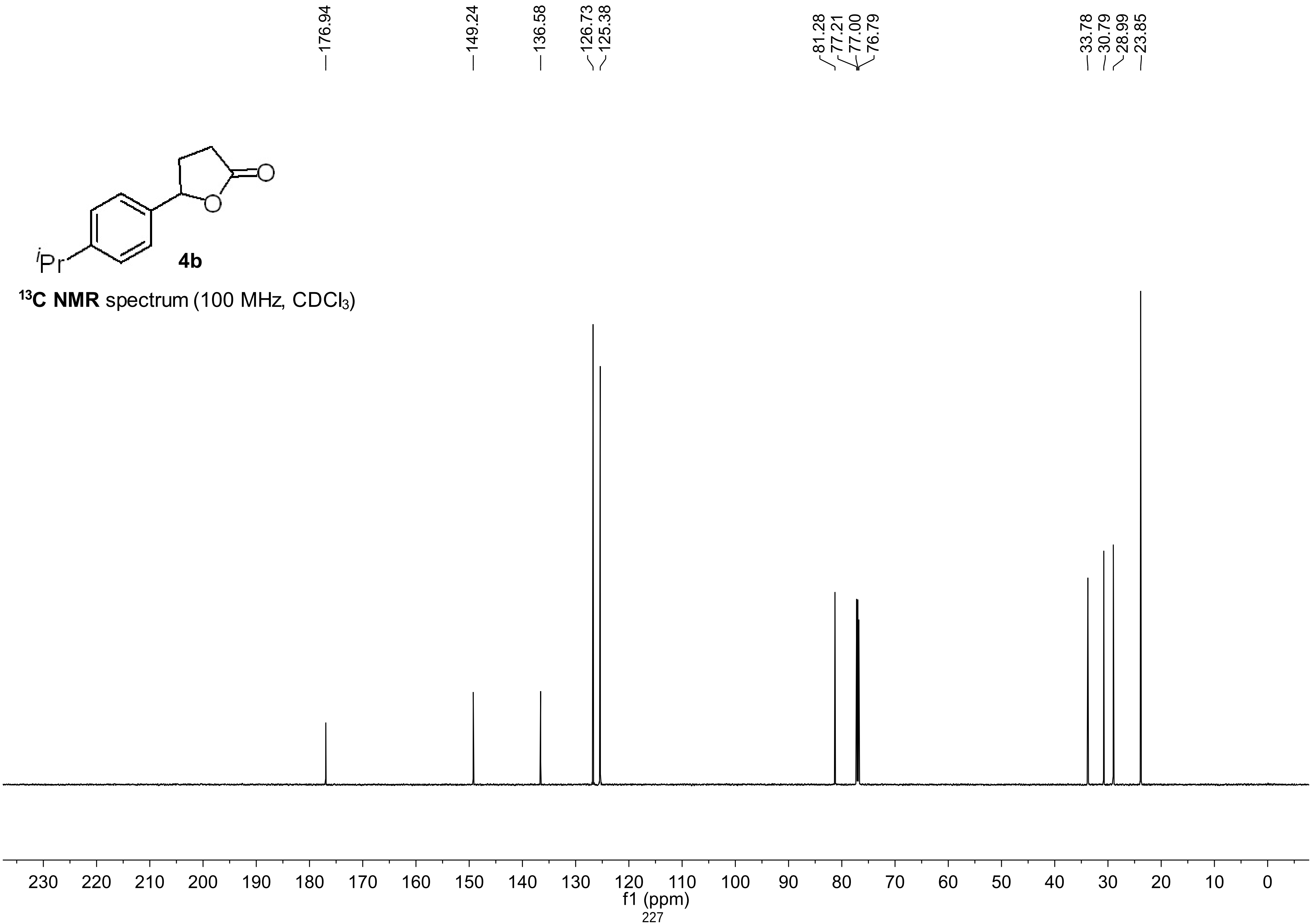

^{13}C NMR spectrum (100 MHz, $CDCl_3$)

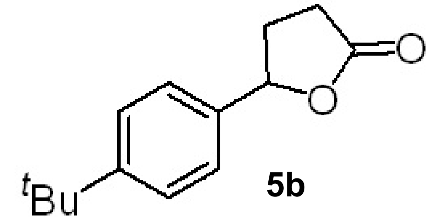

5b

¹H NMR spectrum (600 MHz, $CDCl_3$)

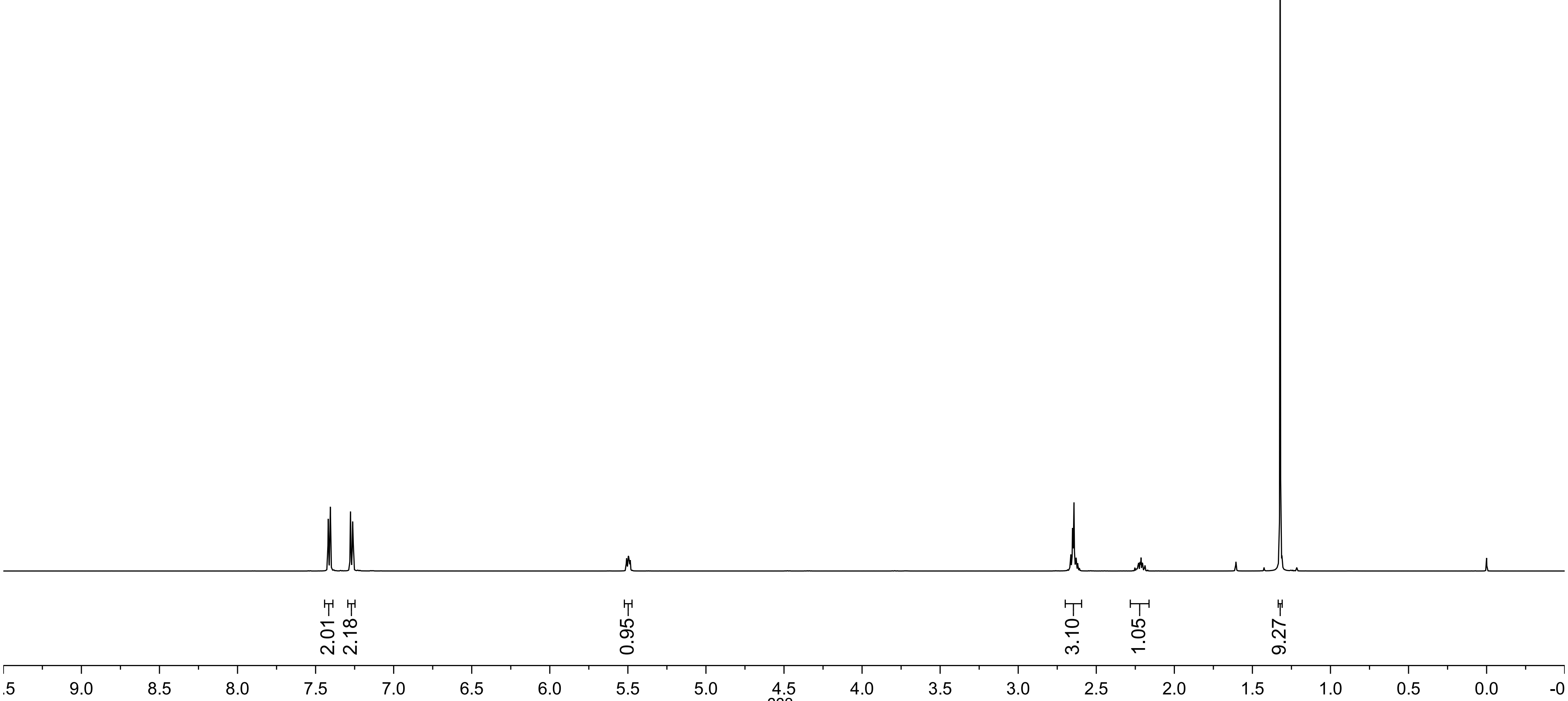

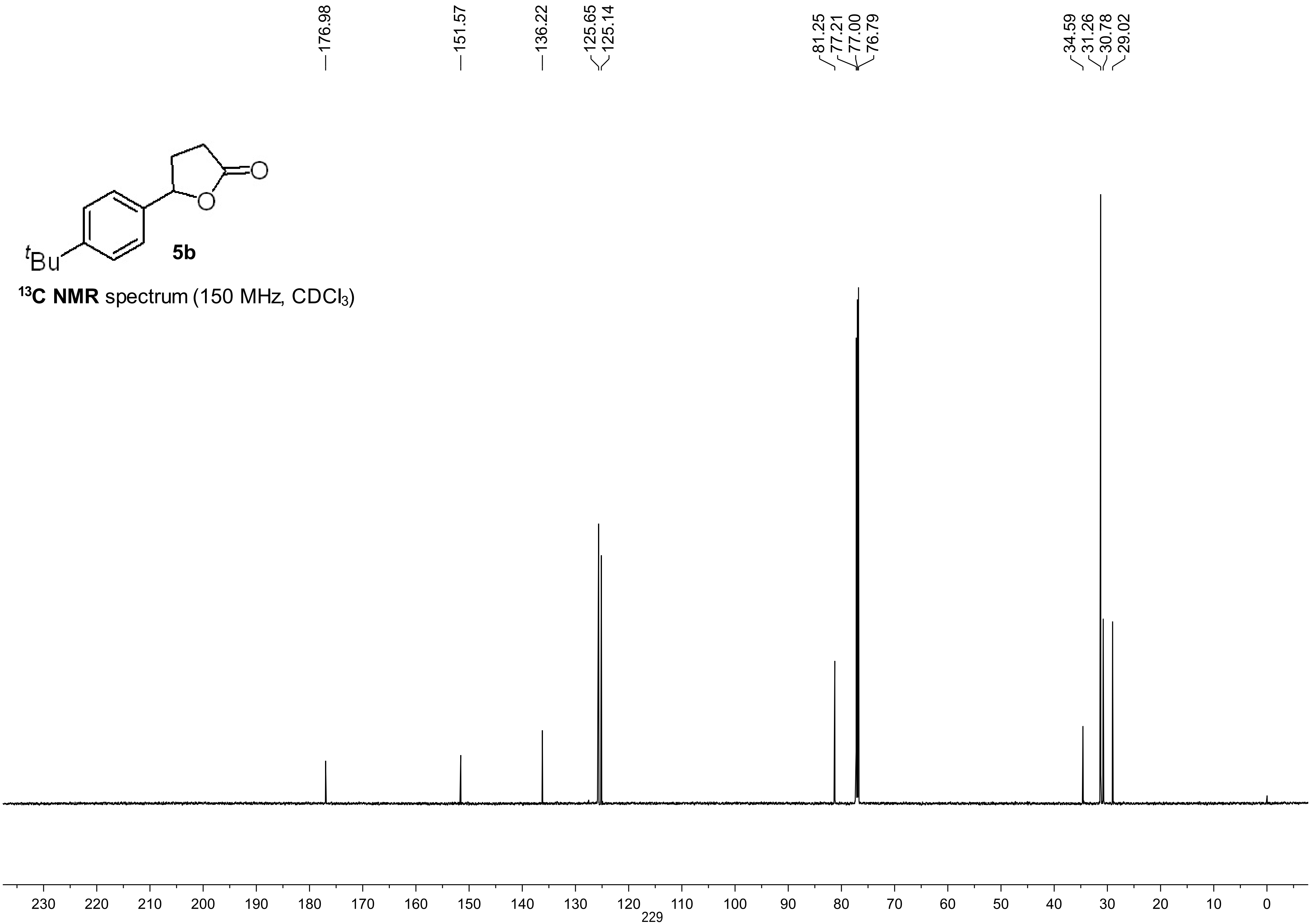

5b

^{13}C NMR spectrum (150 MHz, $CDCl_3$)

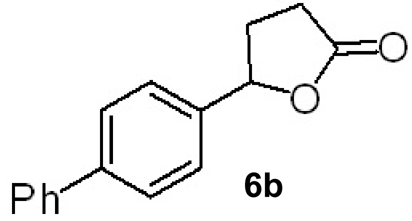

6b

^{1}H NMR spectrum (400 MHz, $CDCl_3$)

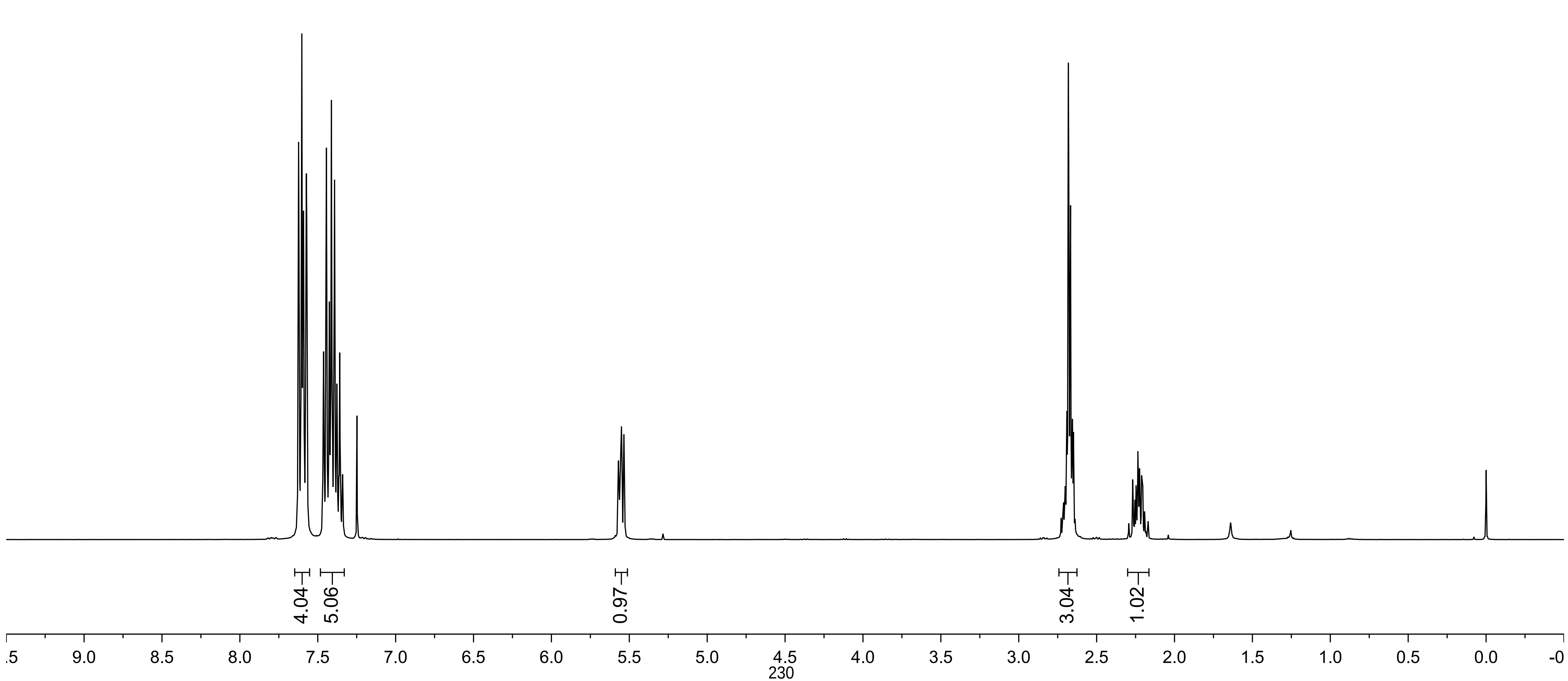

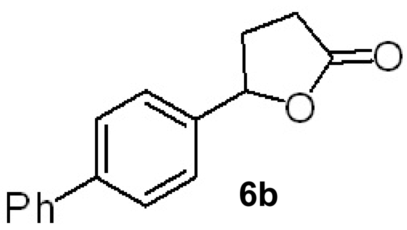

6b

^{13}C NMR spectrum (100 MHz, $CDCl_3$)

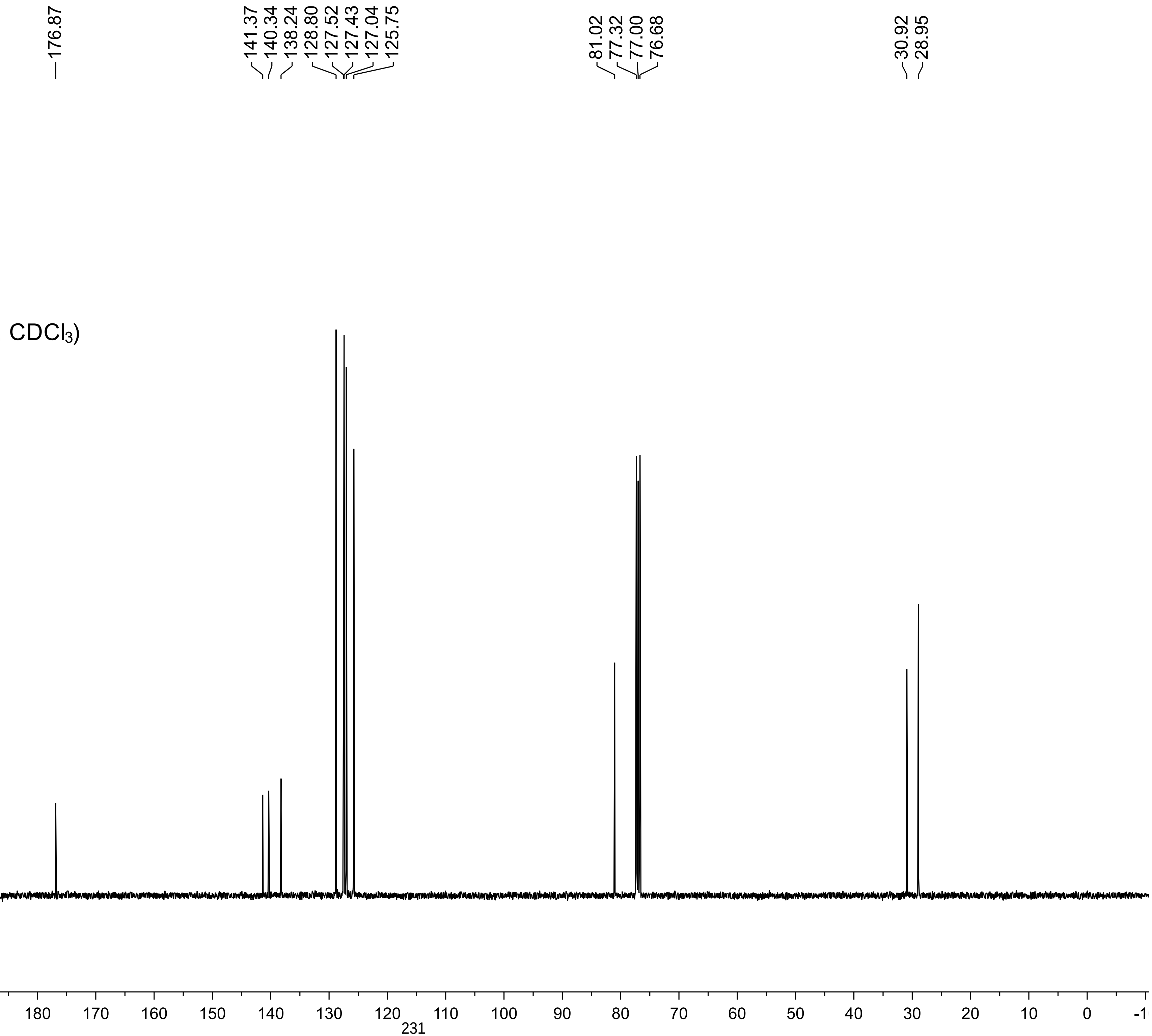

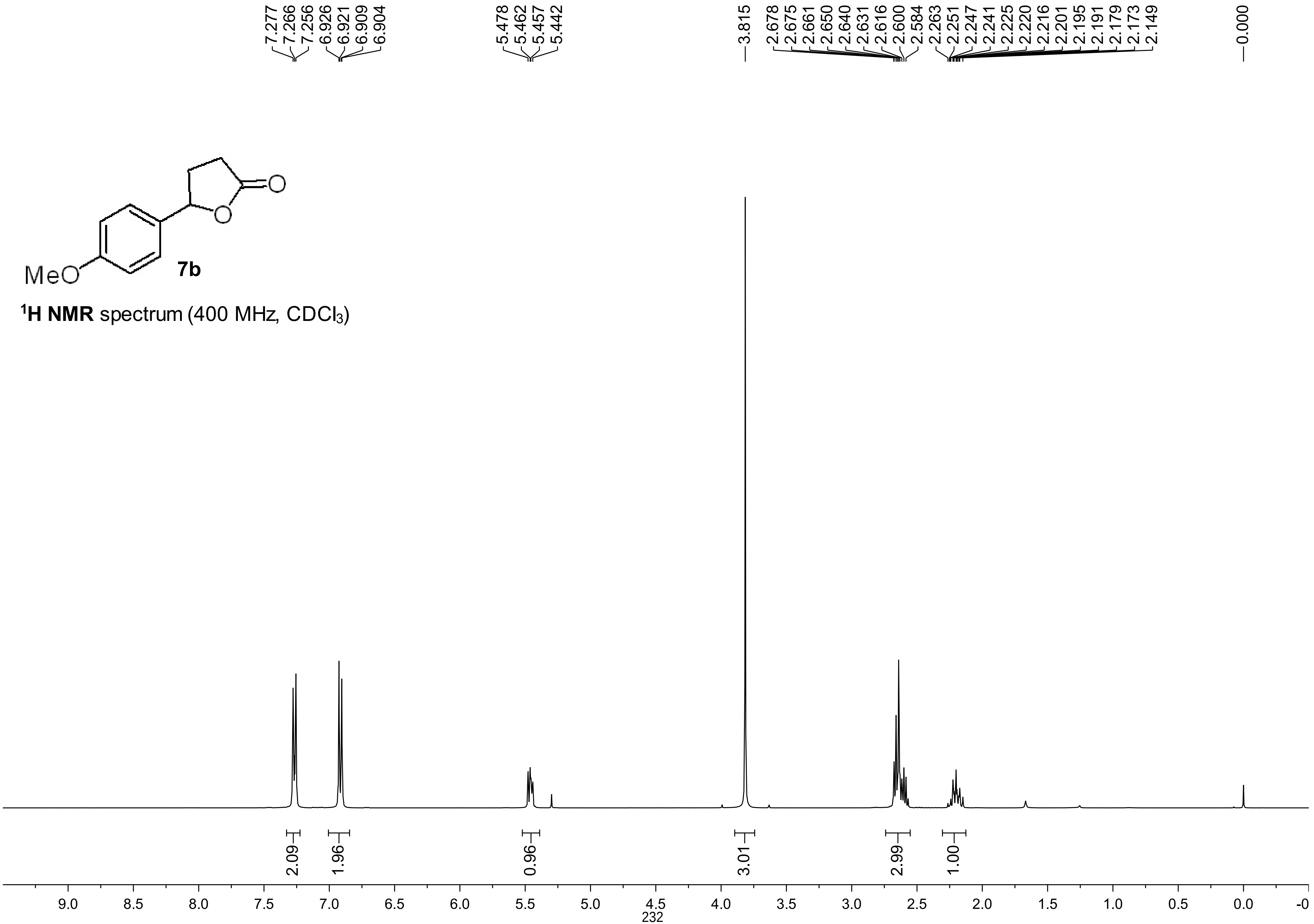

^{1}H NMR spectrum (400 MHz, $CDCl_3$)

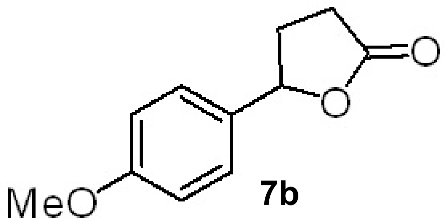

7b

^{13}C NMR spectrum (100 MHz, $CDCl_3$)

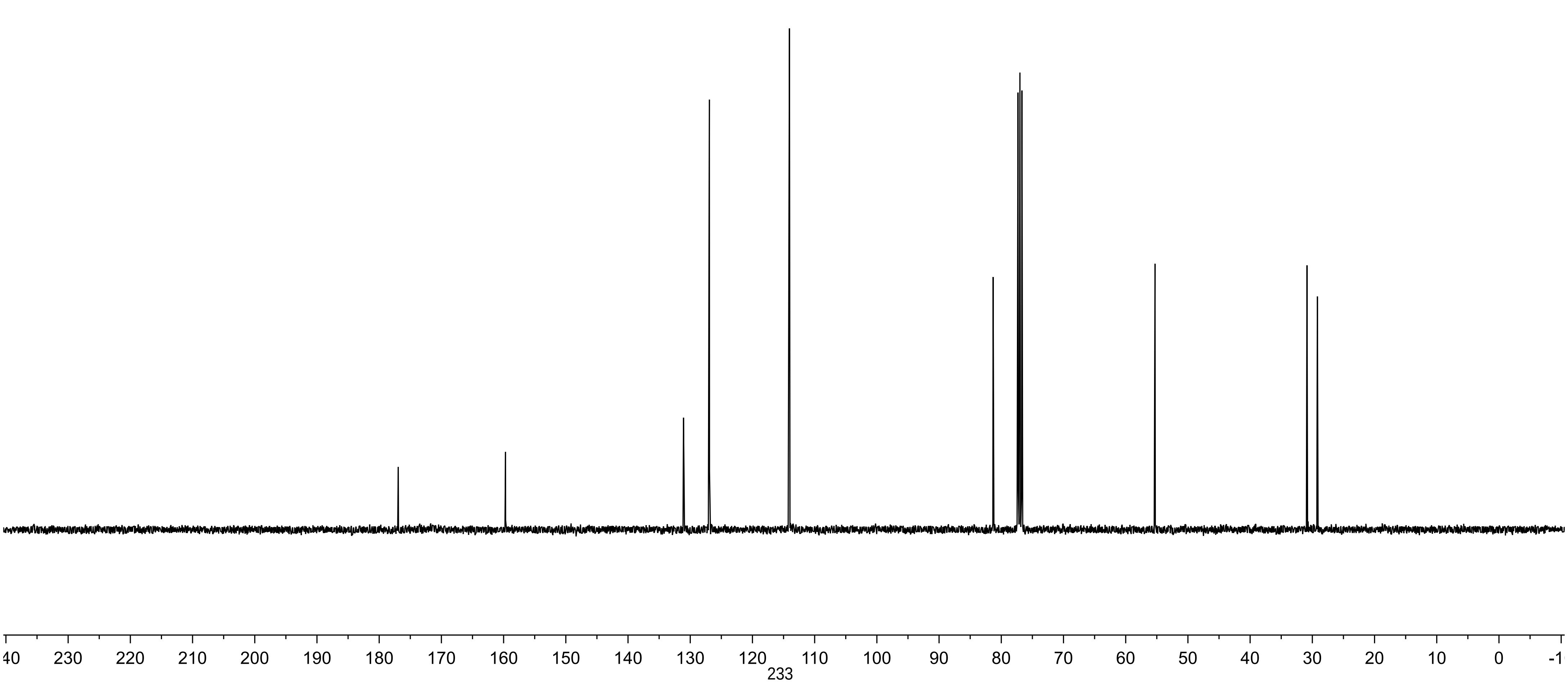

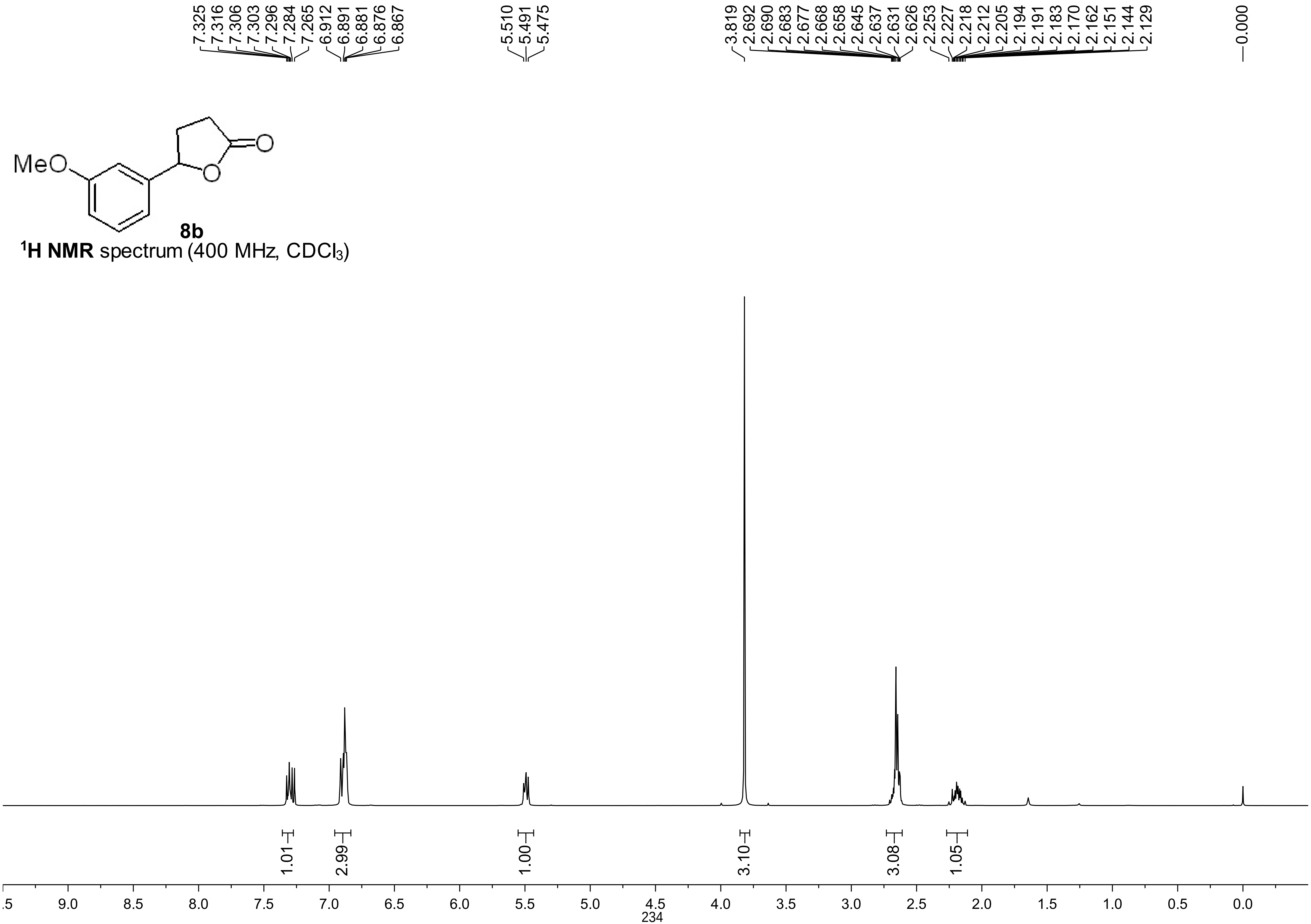

8b

^{1}H NMR spectrum (400 MHz, $CDCl_3$)

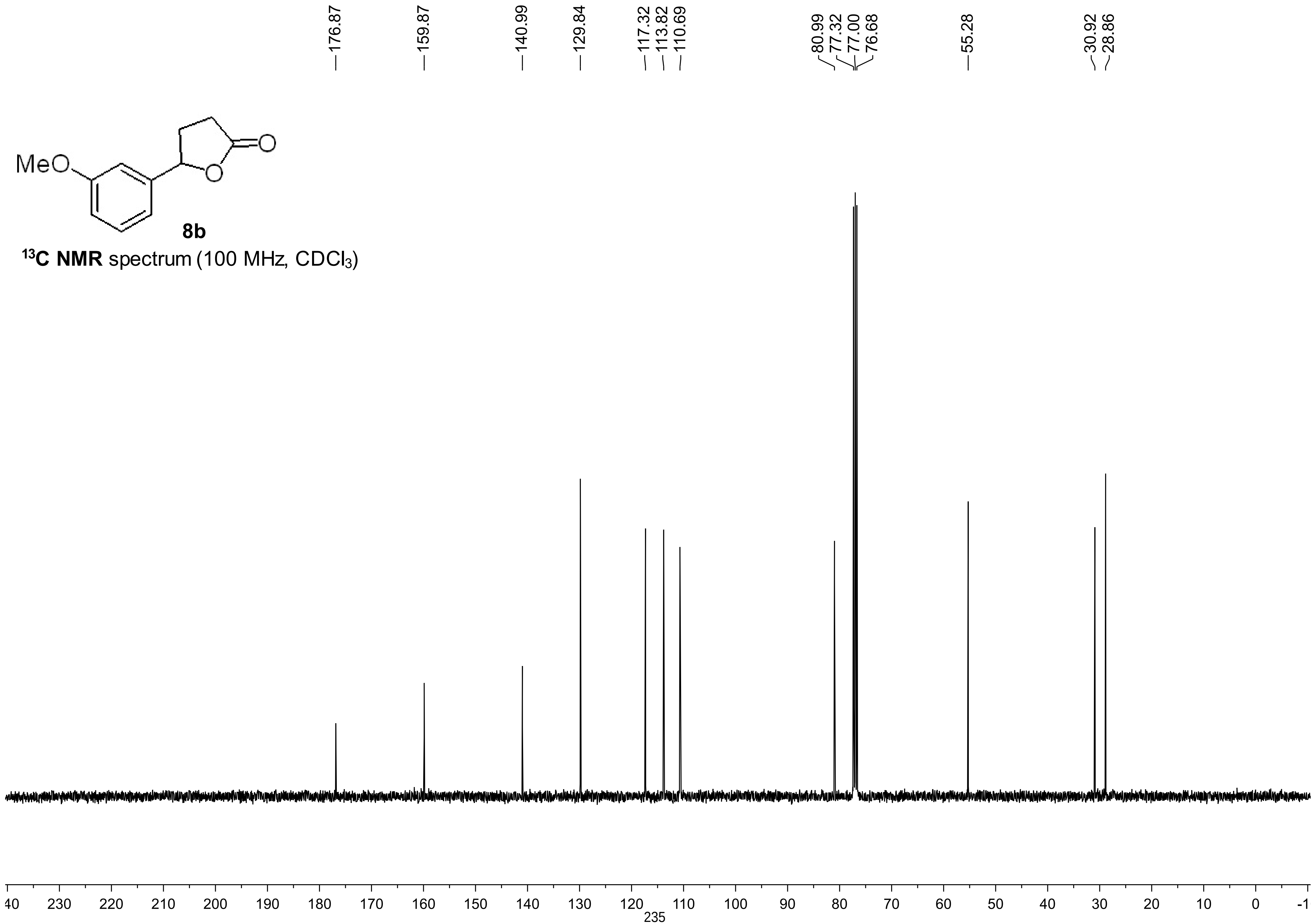

8b

^{13}C NMR spectrum (100 MHz, $CDCl_3$)

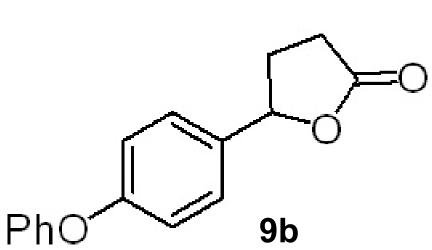

9b

^{1}H NMR spectrum (600 MHz, $CDCl_3$)

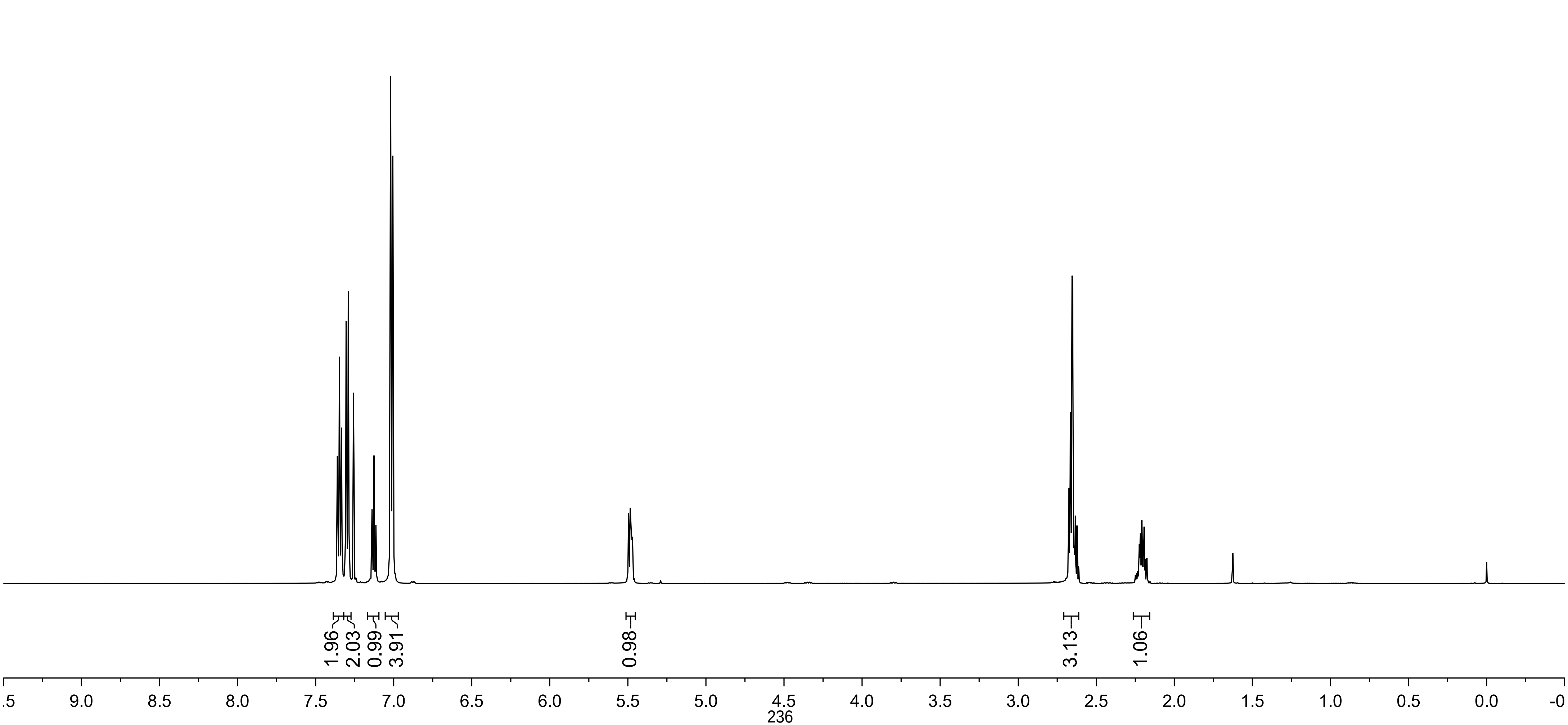

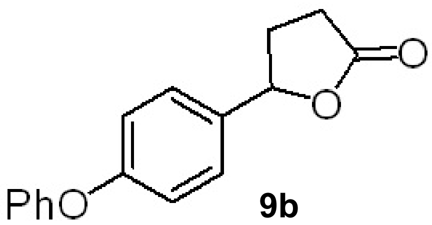

9b

^{13}C NMR spectrum (150 MHz, $CDCl_3$)

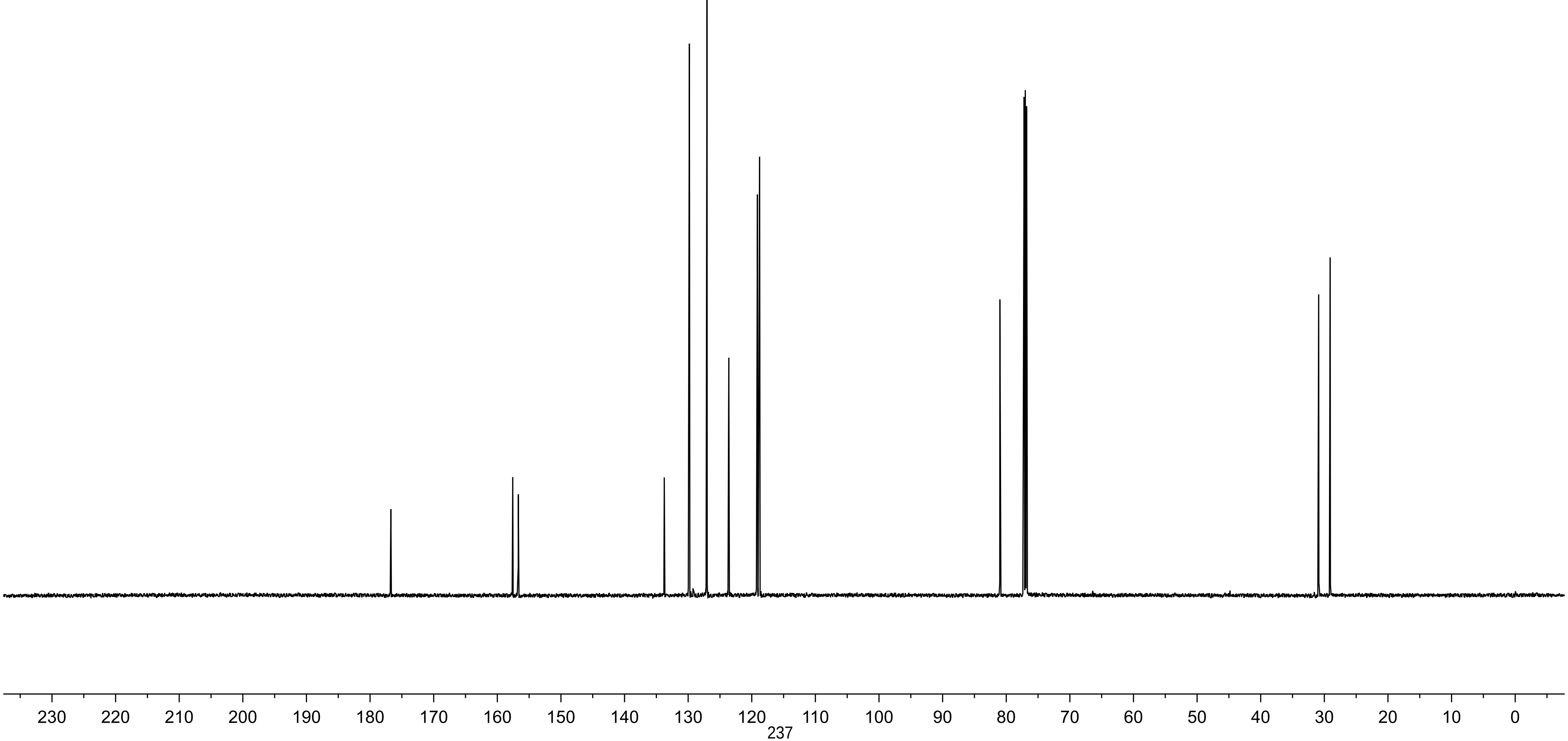

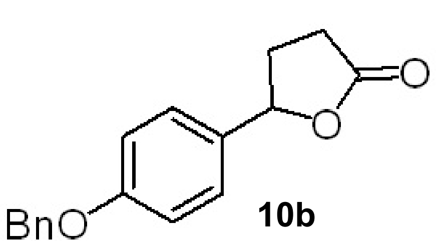

10b

^{1}H NMR spectrum (600 MHz, $CDCl_3$)

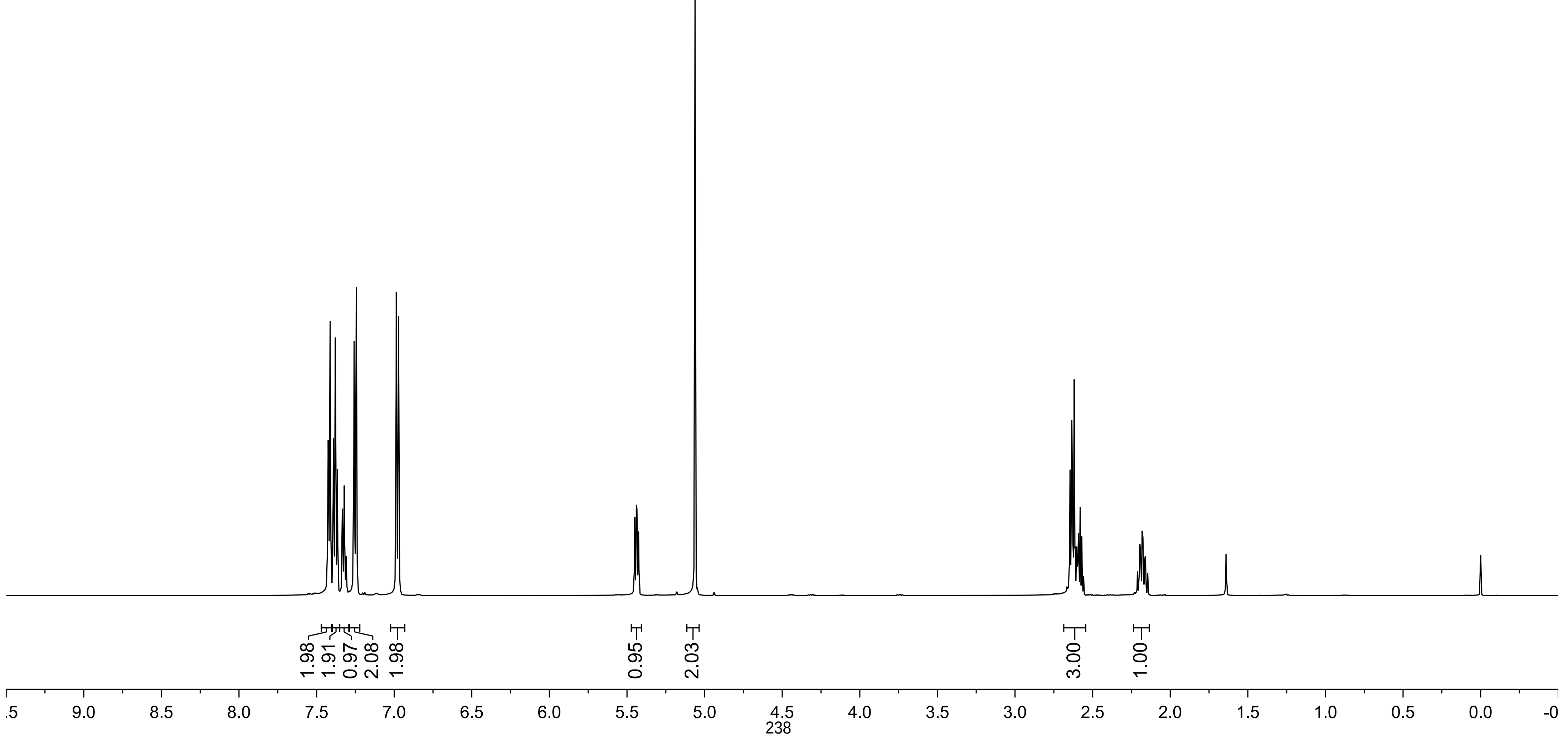

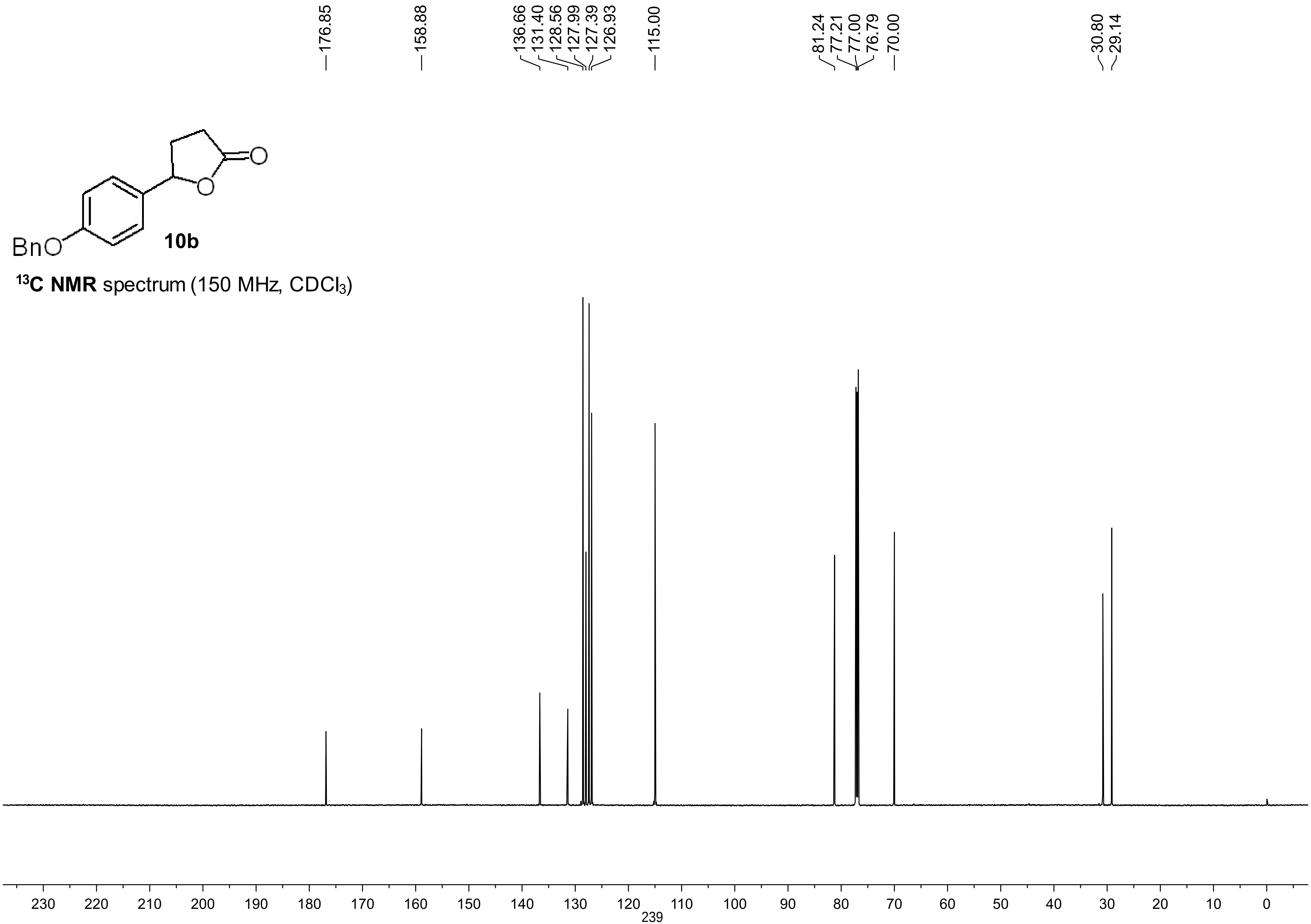

10b

^{13}C NMR spectrum (150 MHz, $CDCl_3$)

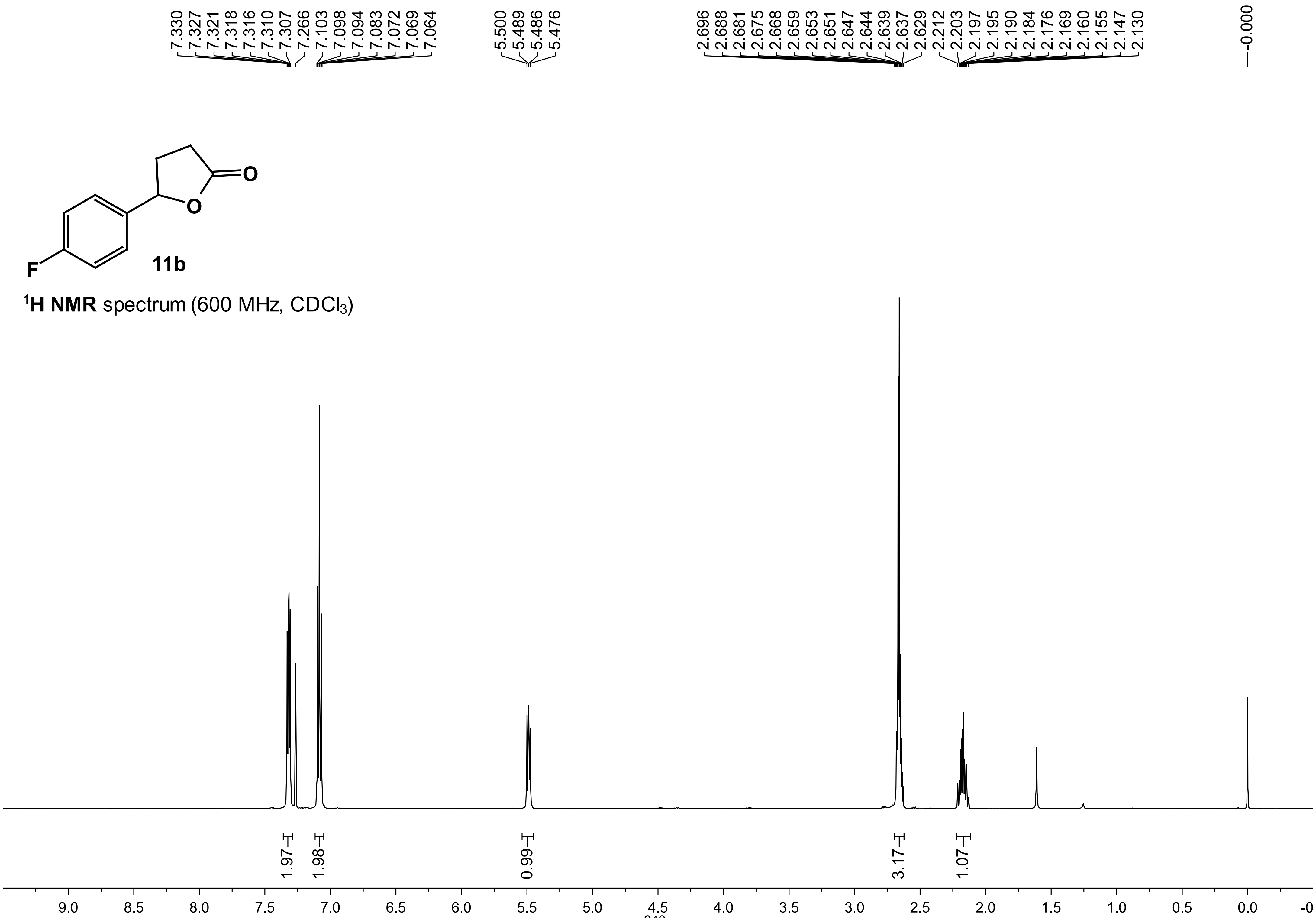
7.330 7.327 7.321 7.318 7.316 7.310 7.307 7.266 7.103 7.098 7.094 7.083 7.072 7.069 7.064
5.500 5.489 5.486 5.476
2.696 2.688 2.681 2.675 2.668 2.659 2.653 2.651 2.647 2.644 2.639 2.637 2.629 2.212 2.203 2.197 2.195 2.190 2.184 2.176 2.169 2.160 2.155 2.147 2.130
-0.000
O
O
F
11b
^{1}H NMR spectrum (600 MHz, $CDCl_3$)
1.97
1.98
0.99
3.17
1.07
9.0 8.5 8.0 7.5 7.0 6.5 6.0 5.5 5.0 4.5 4.0 3.5 3.0 2.5 2.0 1.5 1.0 0.5 0.0 -0

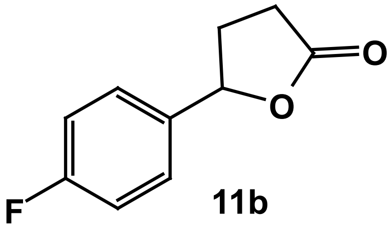

11b

^{13}C NMR spectrum (150 MHz, $CDCl_3$)

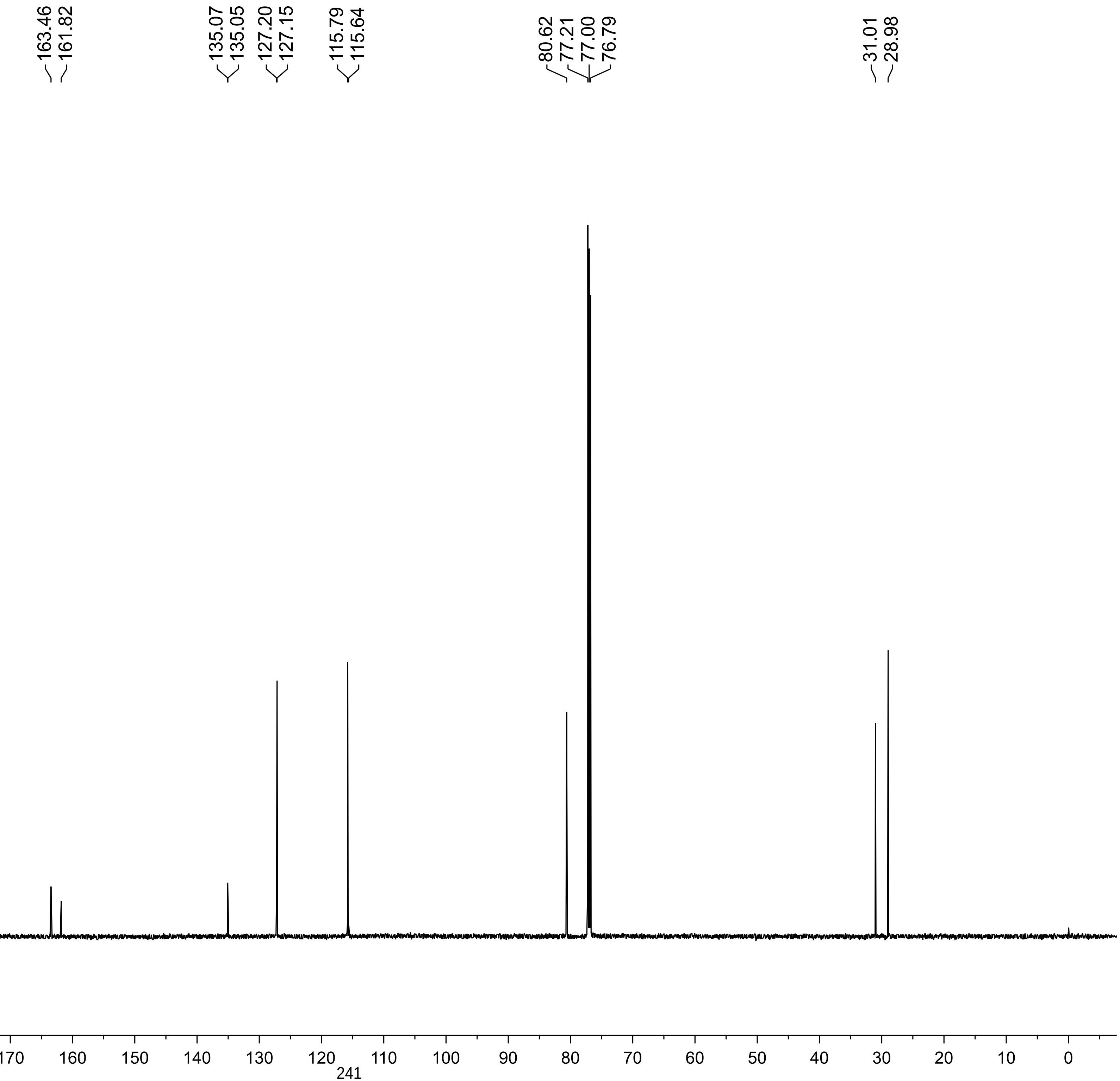

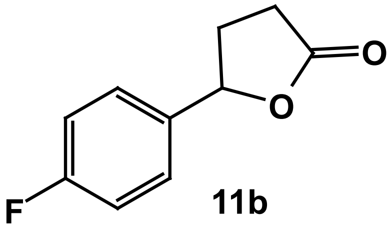

11b

^{19}**F NMR** spectrum (376 MHz, $CDCl_3$)

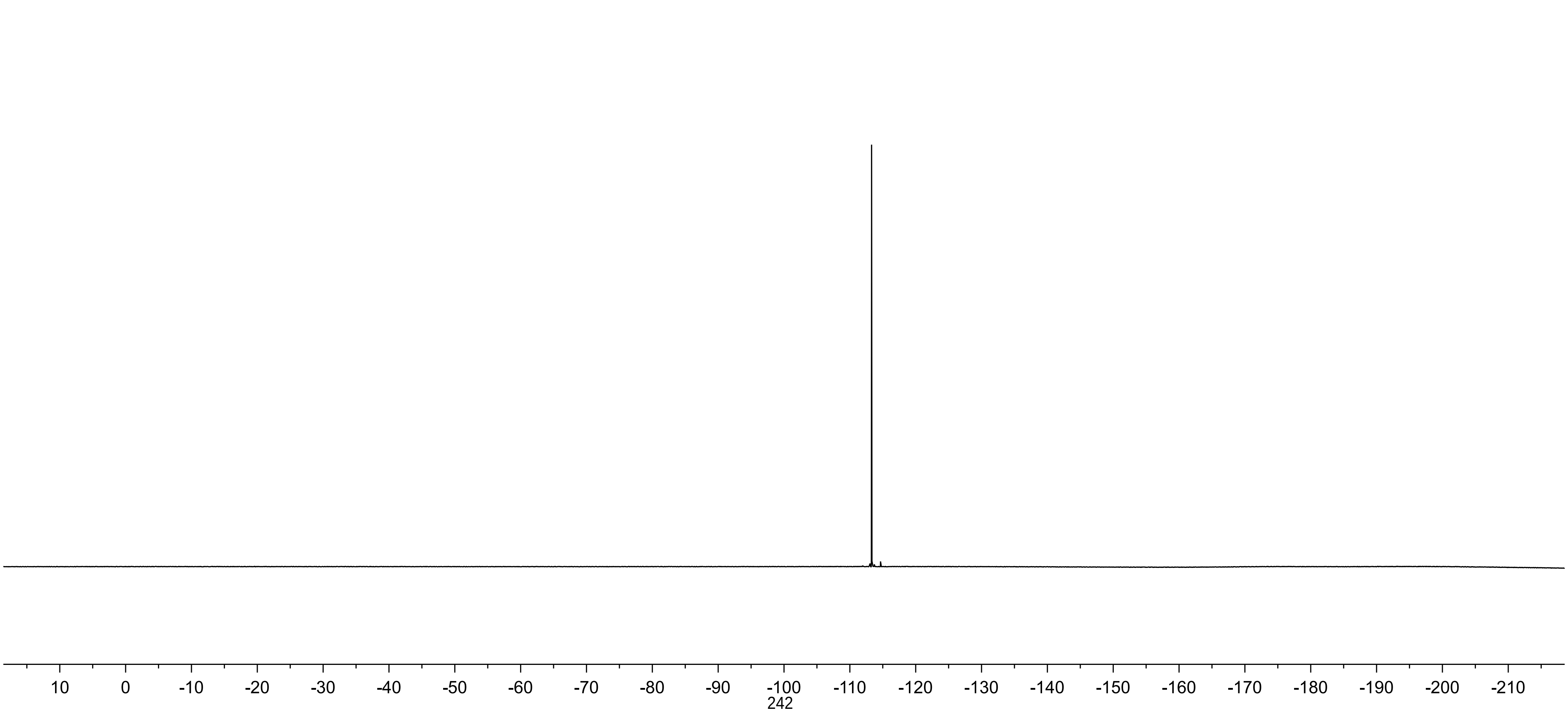

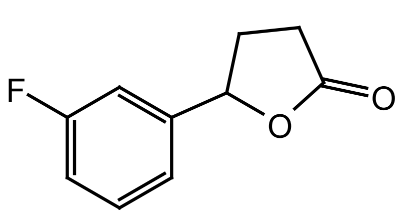

12b

^{1}H NMR spectrum (600 MHz, $CDCl_3$)

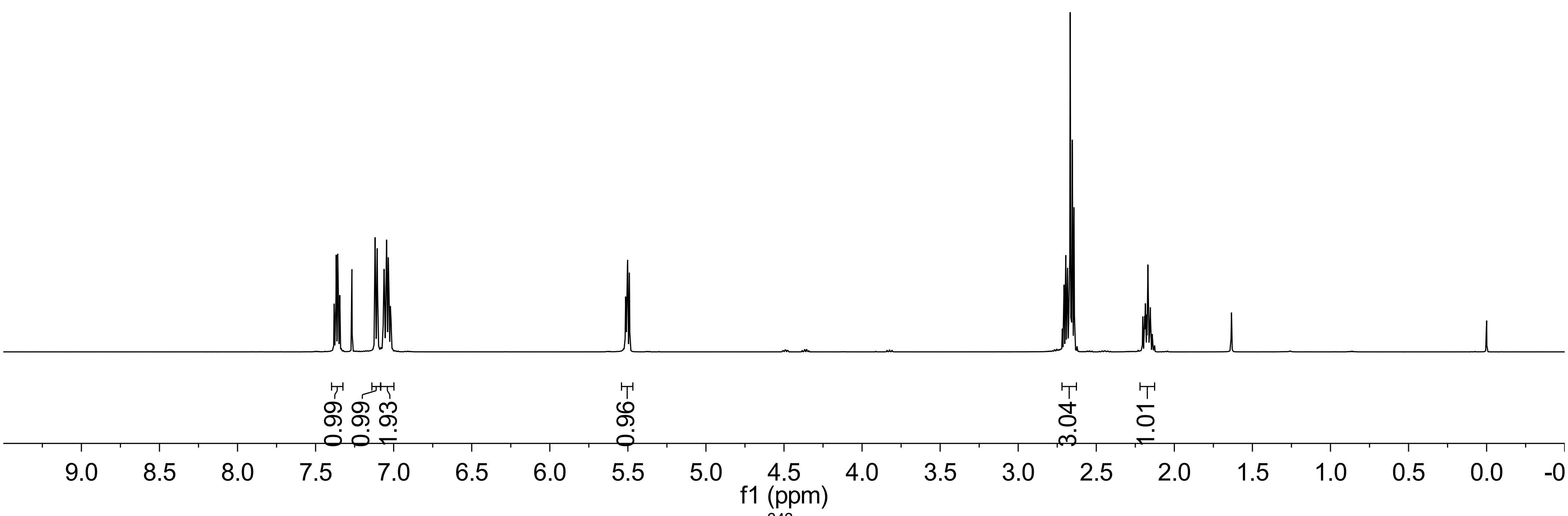

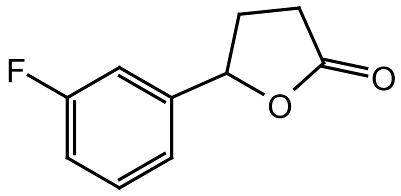

12b

^{13}C NMR spectrum (150 MHz, $CDCl_3$)

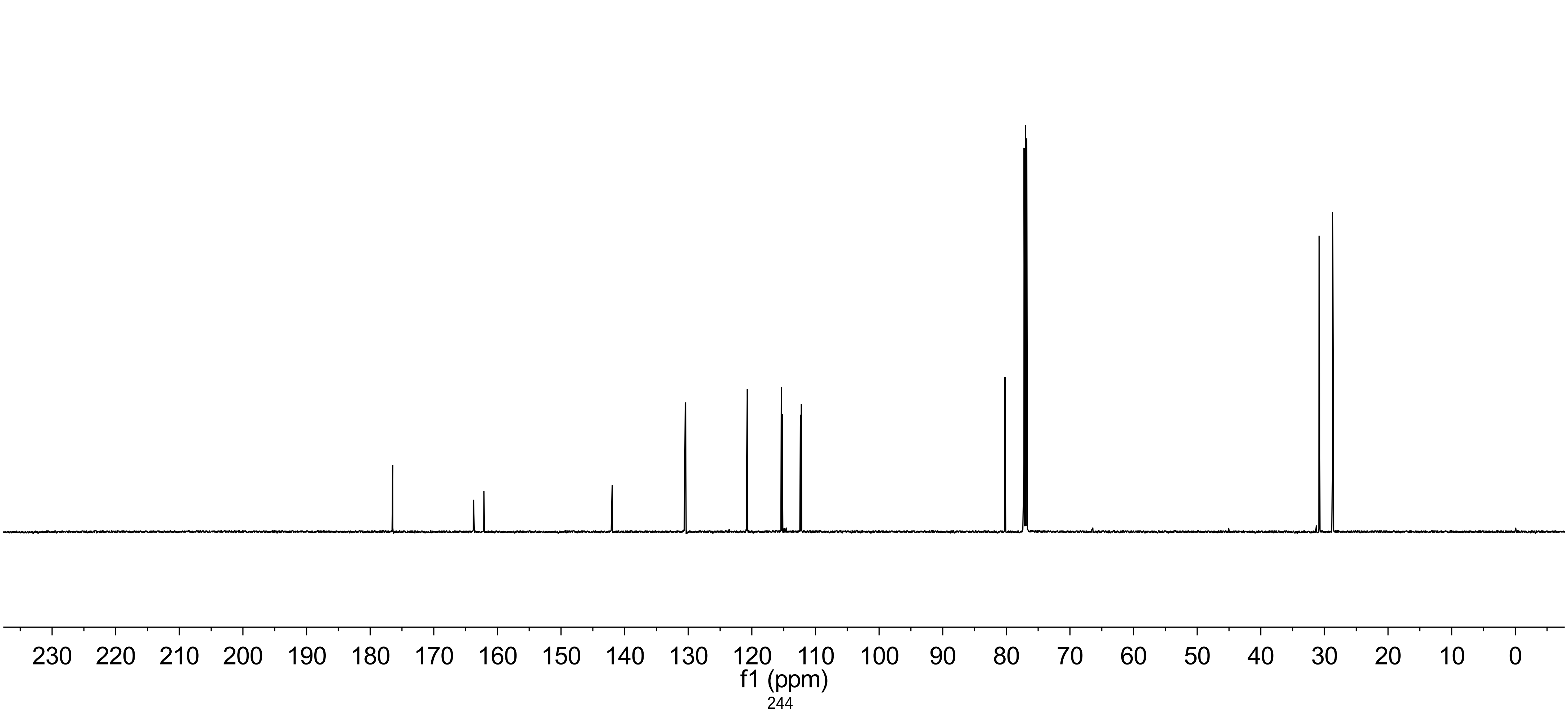

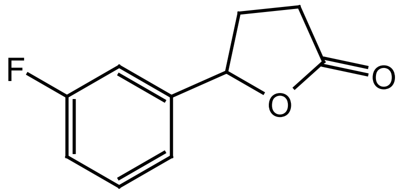

12b

[19]**F NMR** spectrum (376 MHz, $CDCl_3$)

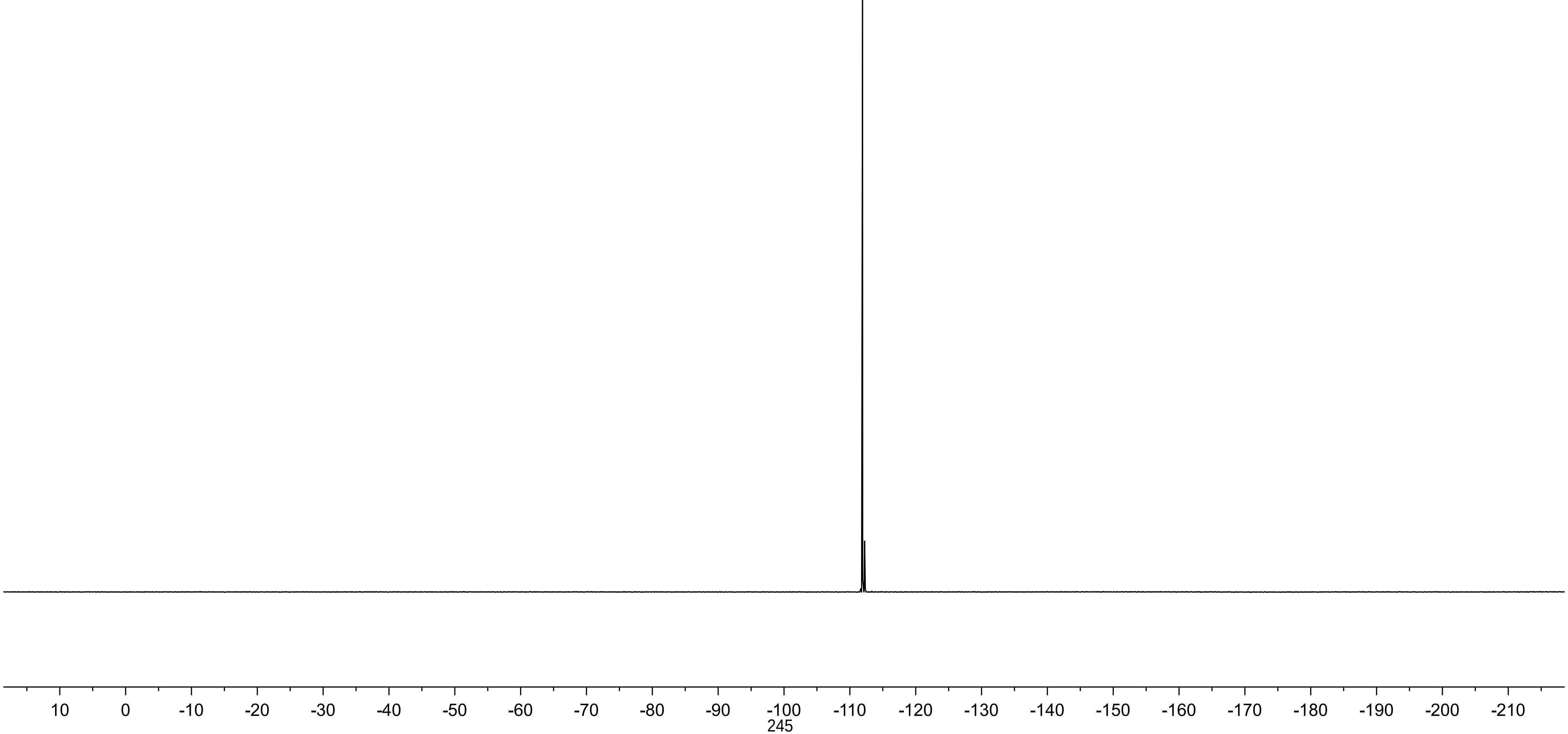

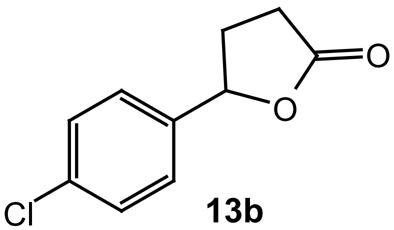

13b

^{1}H NMR spectrum (400 MHz, $CDCl_3$)

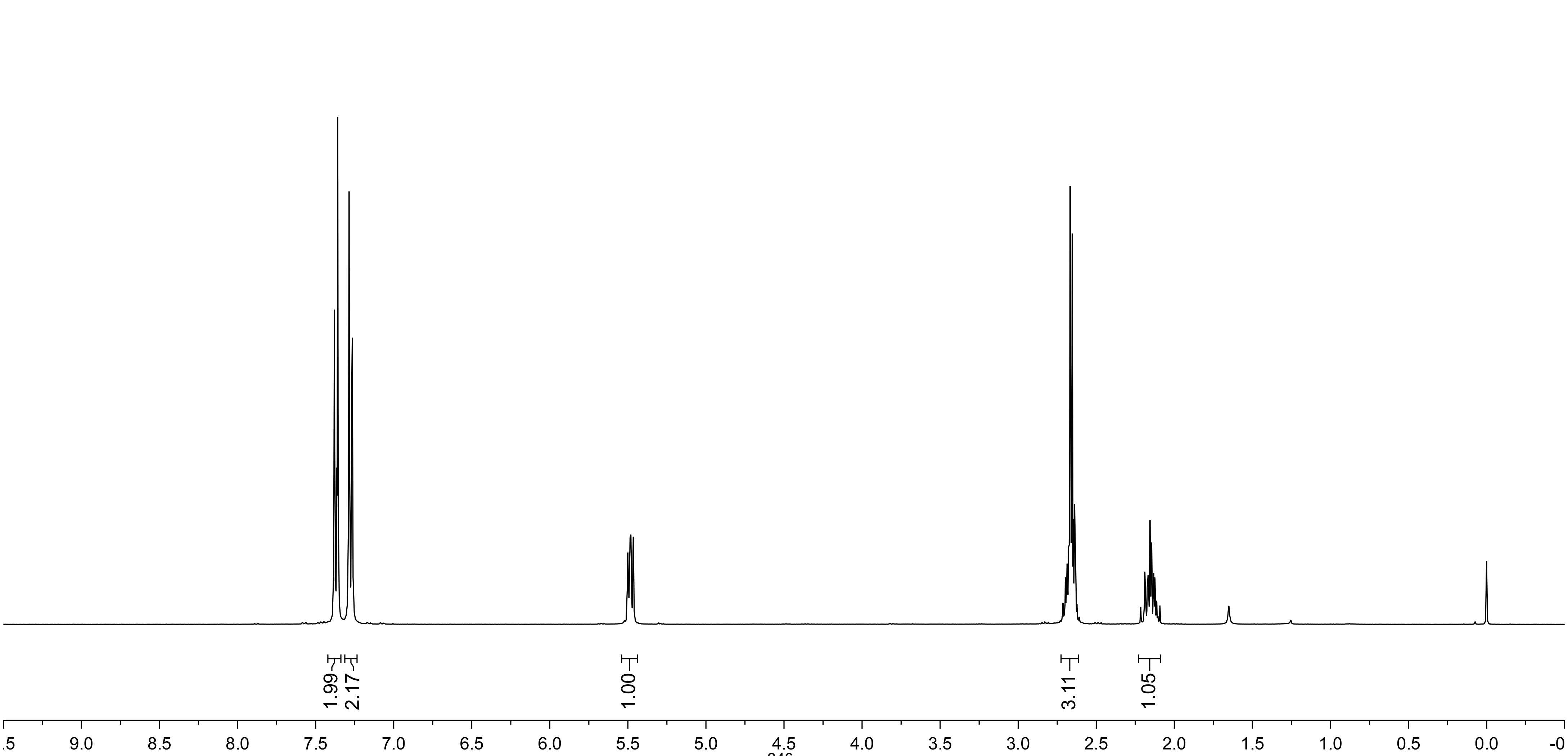

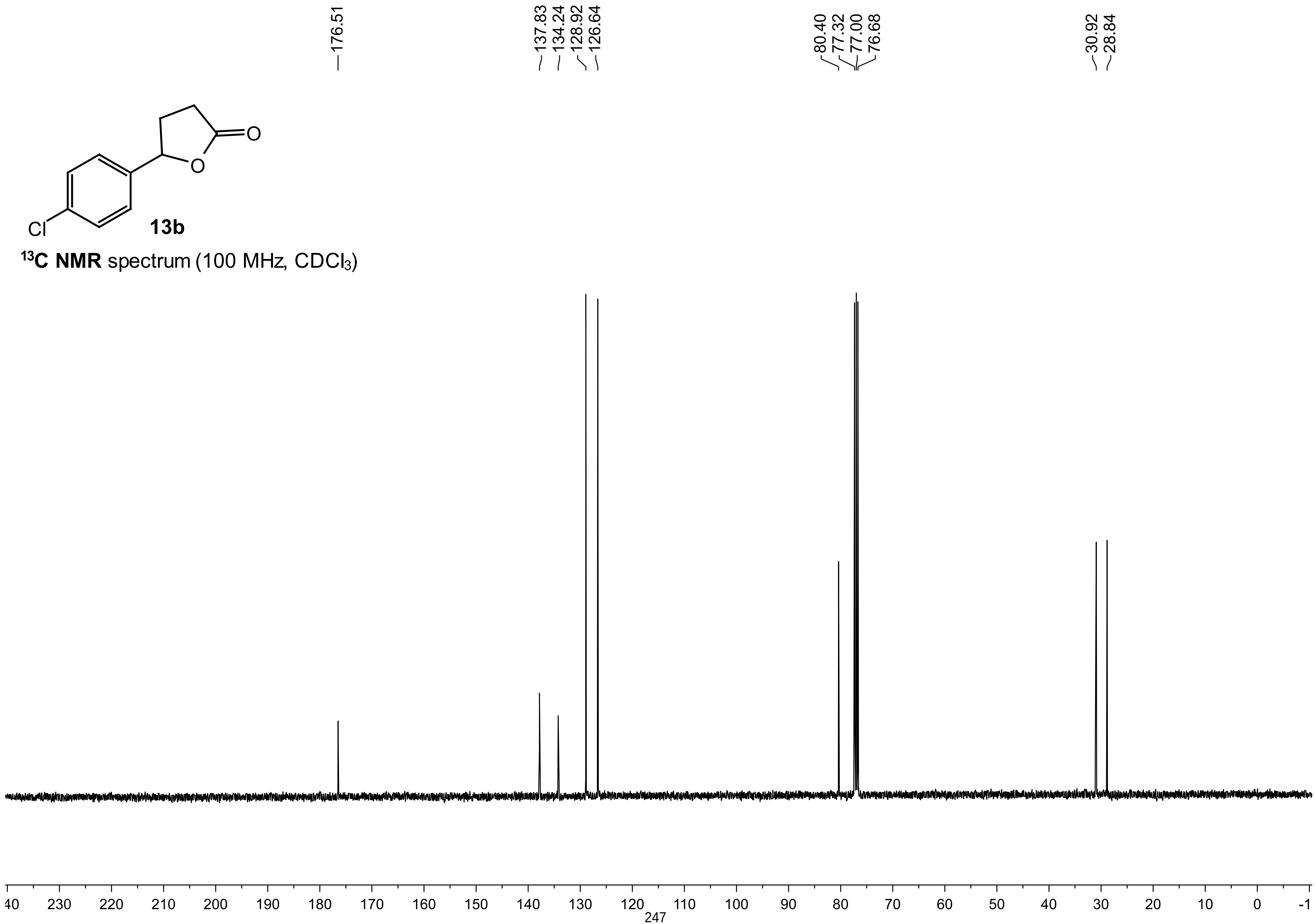

13b

^{13}C NMR spectrum (100 MHz, $CDCl_3$)

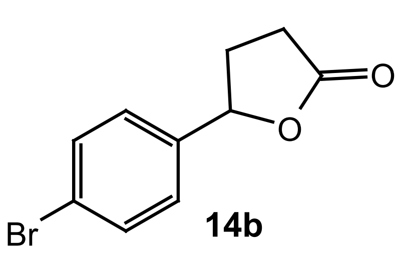

14b

^{1}H NMR spectrum (400 MHz, $CDCl_3$)

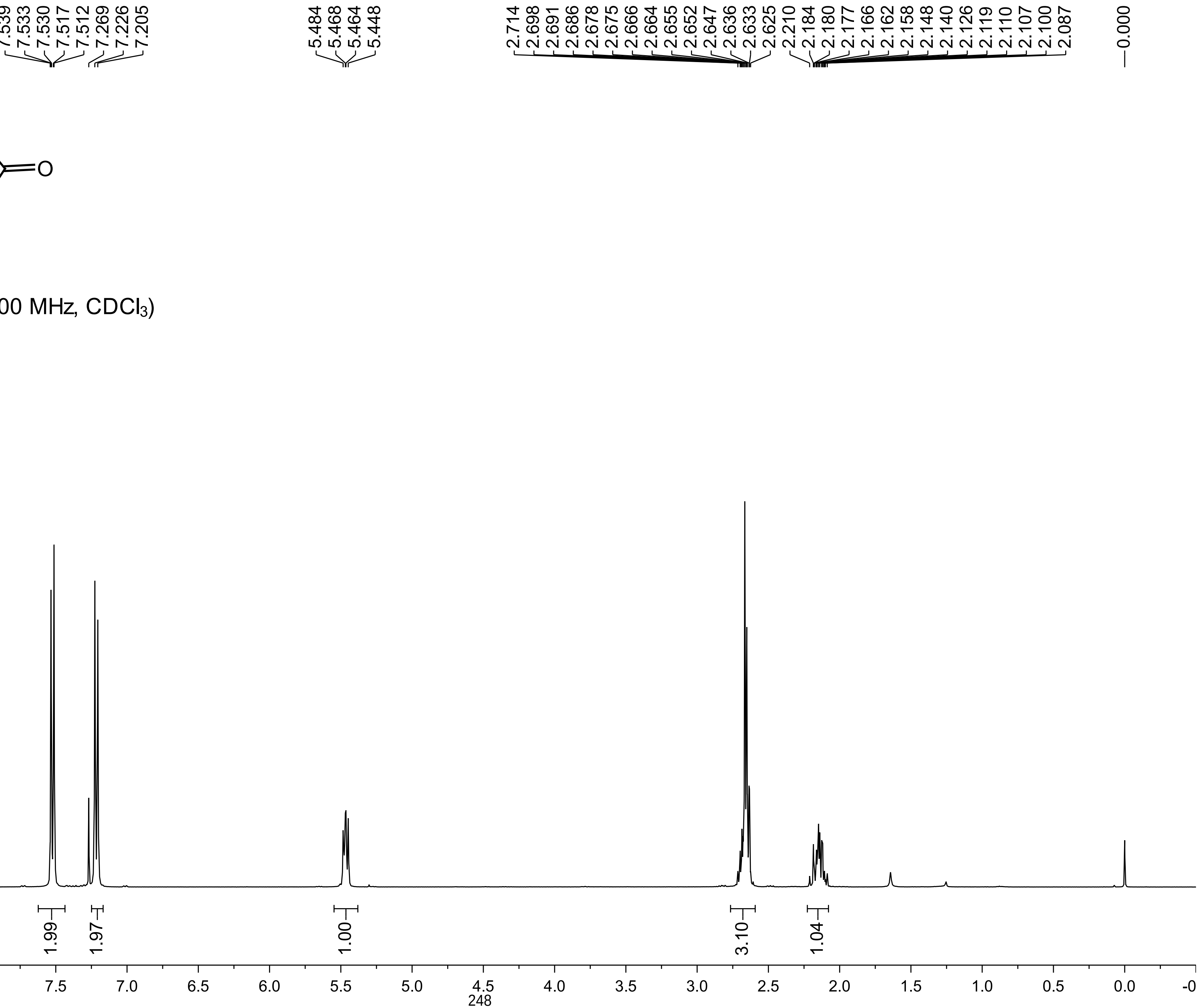

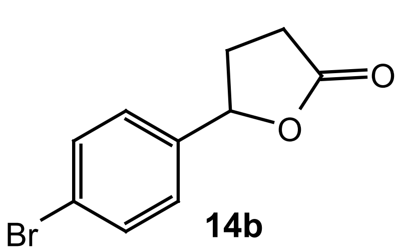

14b

^{13}**C NMR** spectrum (100 MHz, $CDCl_3$)

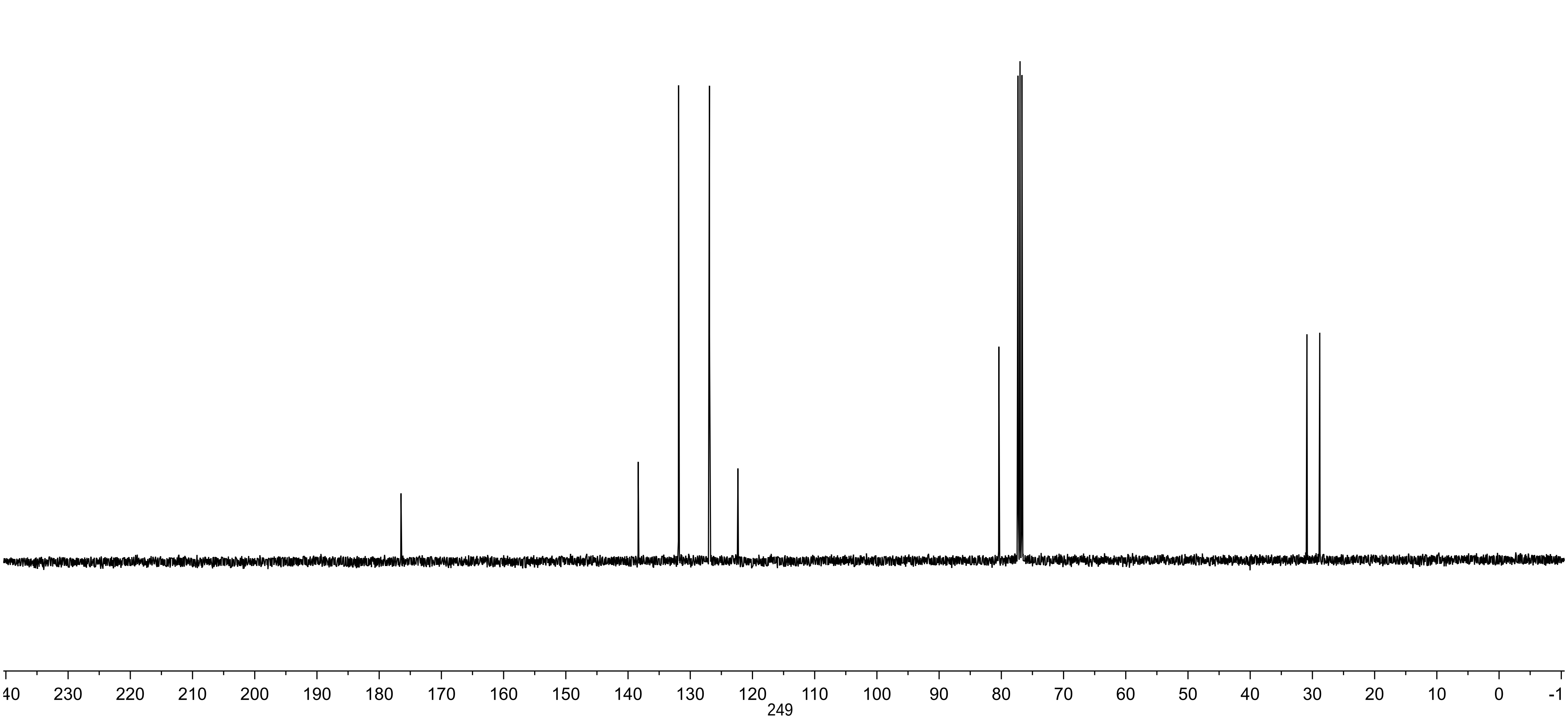

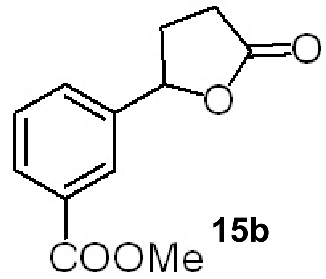

15b

^{1}H NMR spectrum (600 MHz, $CDCl_3$)

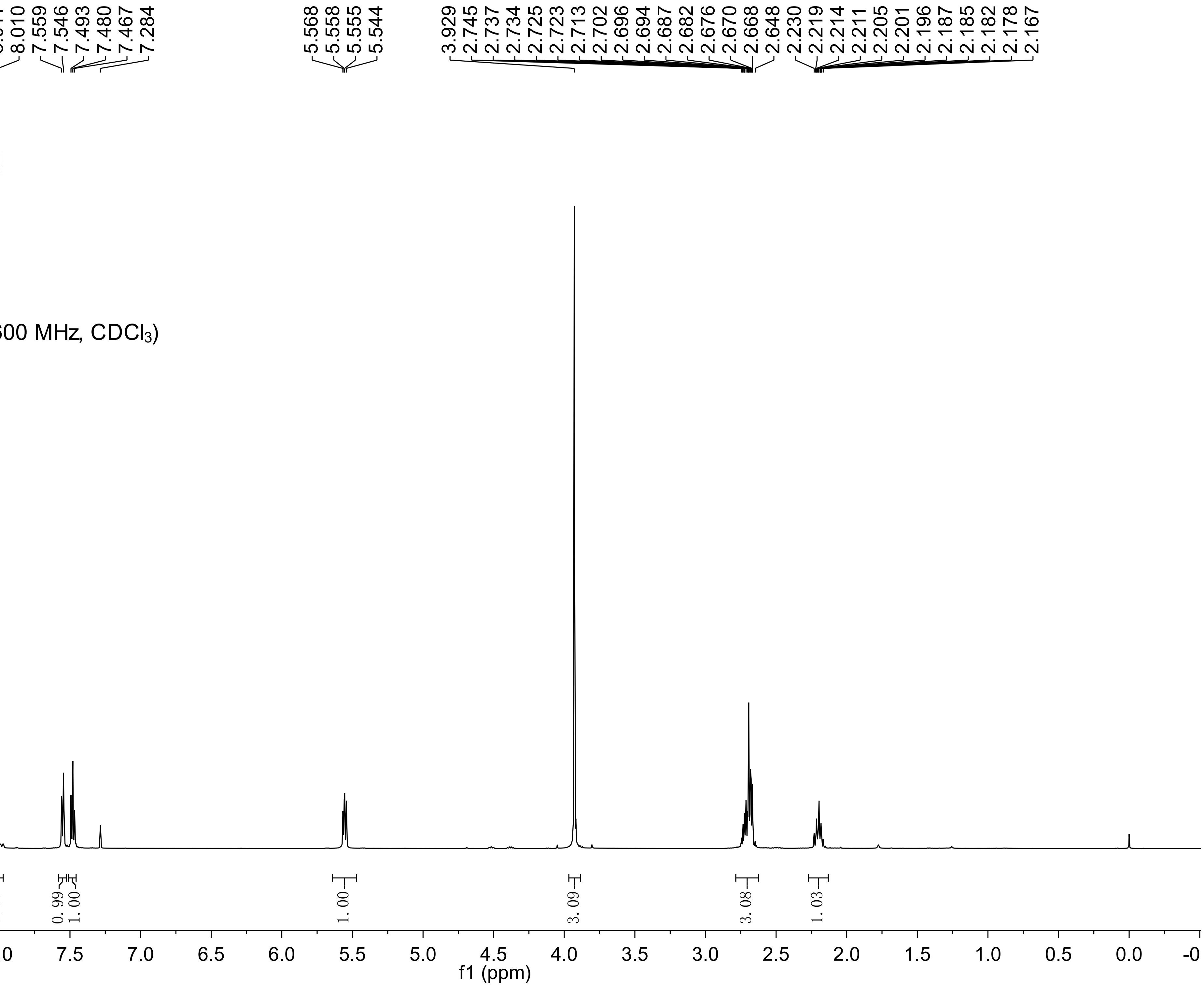

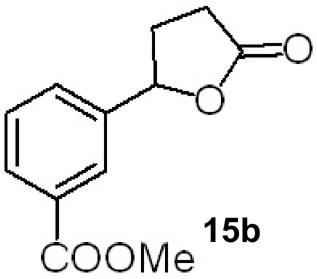

15b

^{13}**C NMR** spectrum (150 MHz, $CDCl_3$)

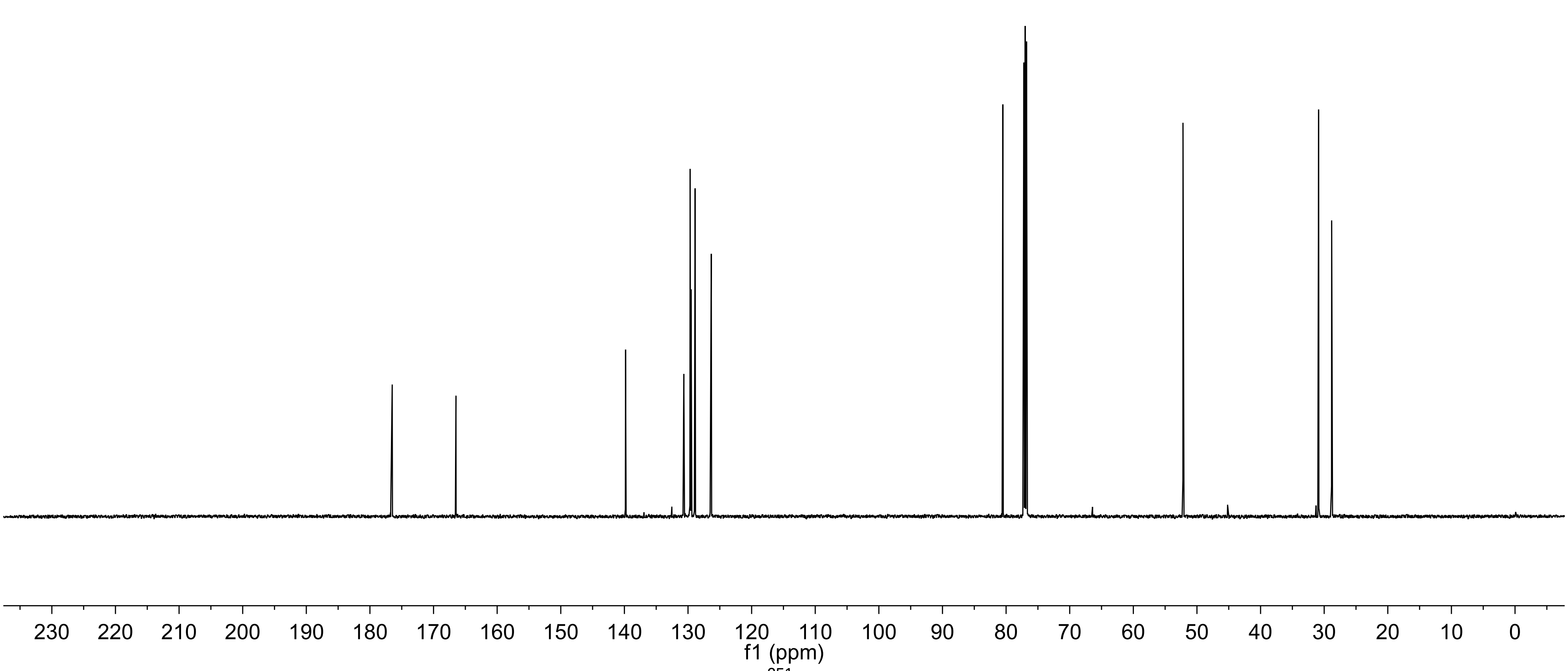

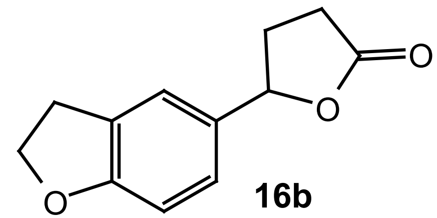

16b

^{1}H NMR spectrum (600 MHz, $CDCl_3$)

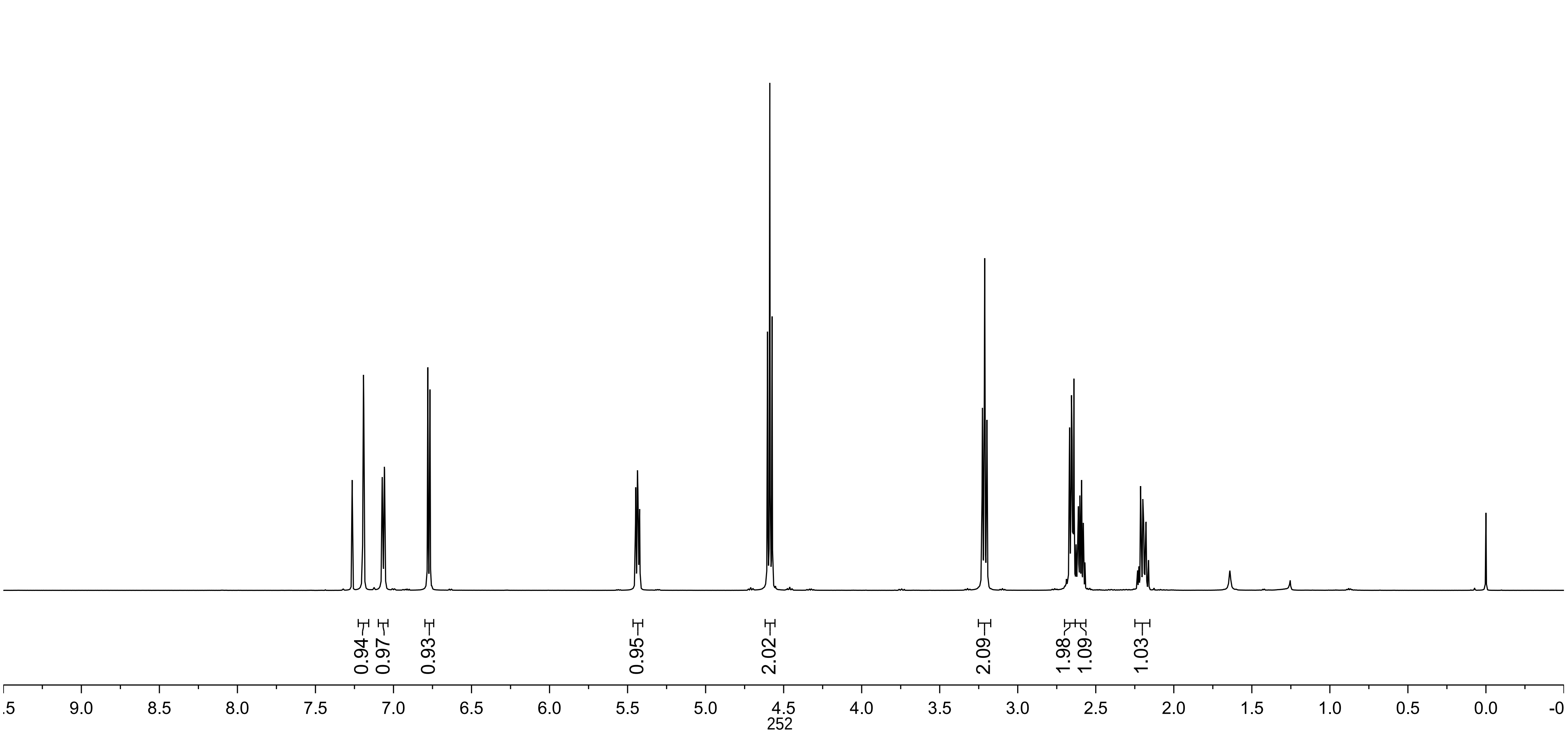

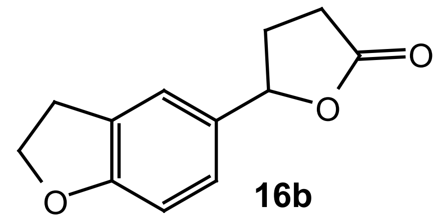

16b

^{13}C NMR spectrum (150 MHz, $CDCl_3$)

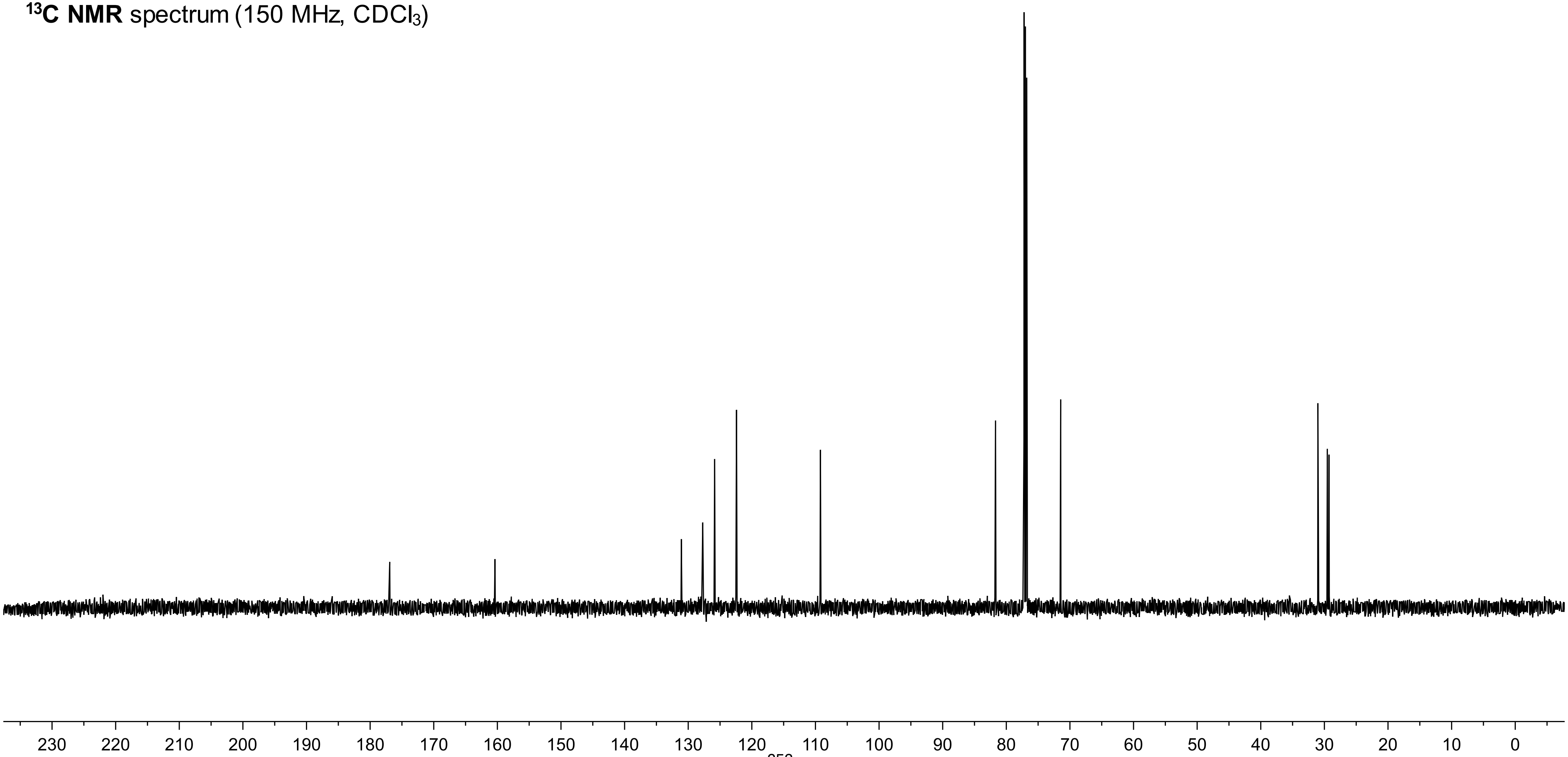

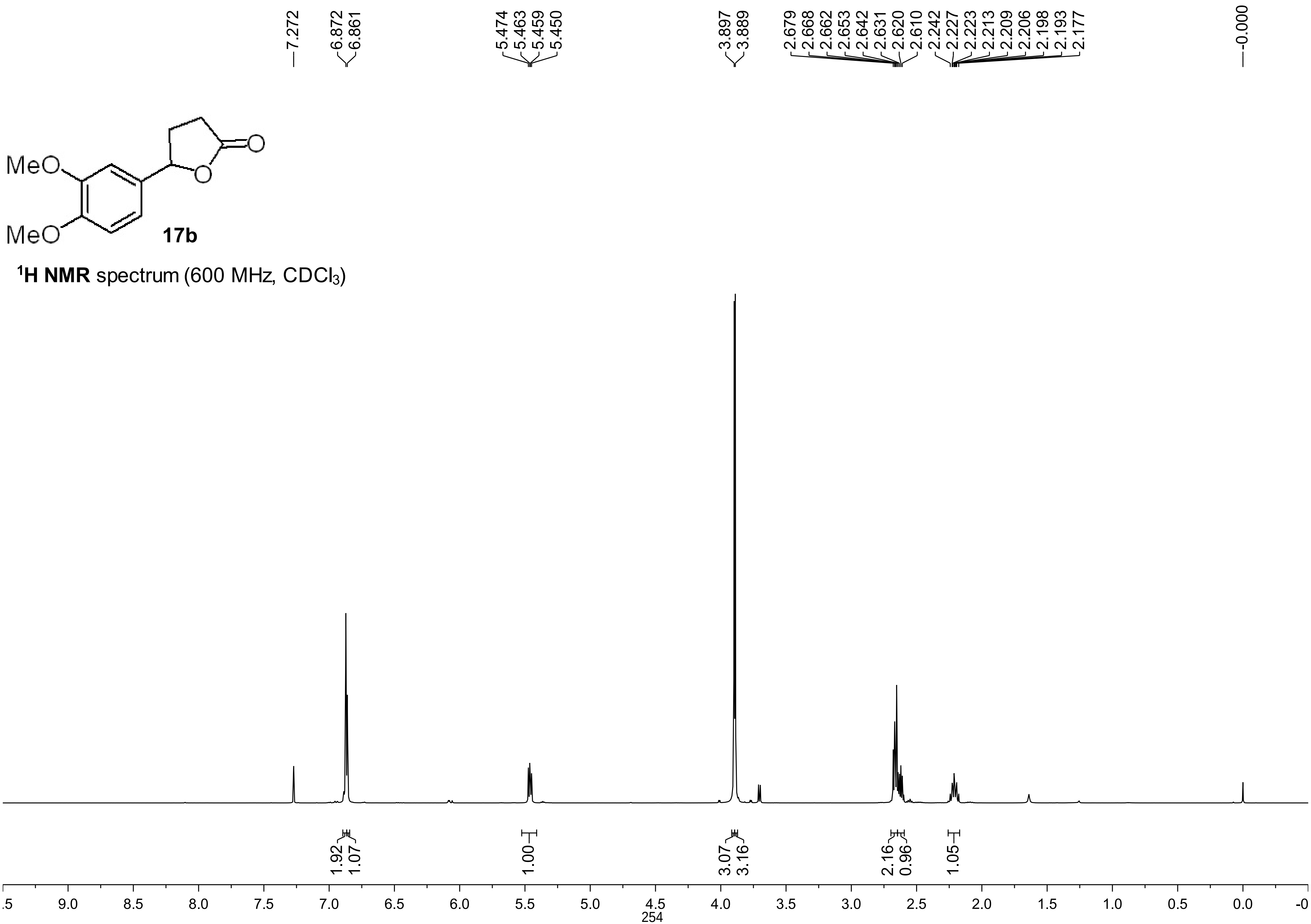

^{1}H NMR spectrum (600 MHz, $CDCl_3$)

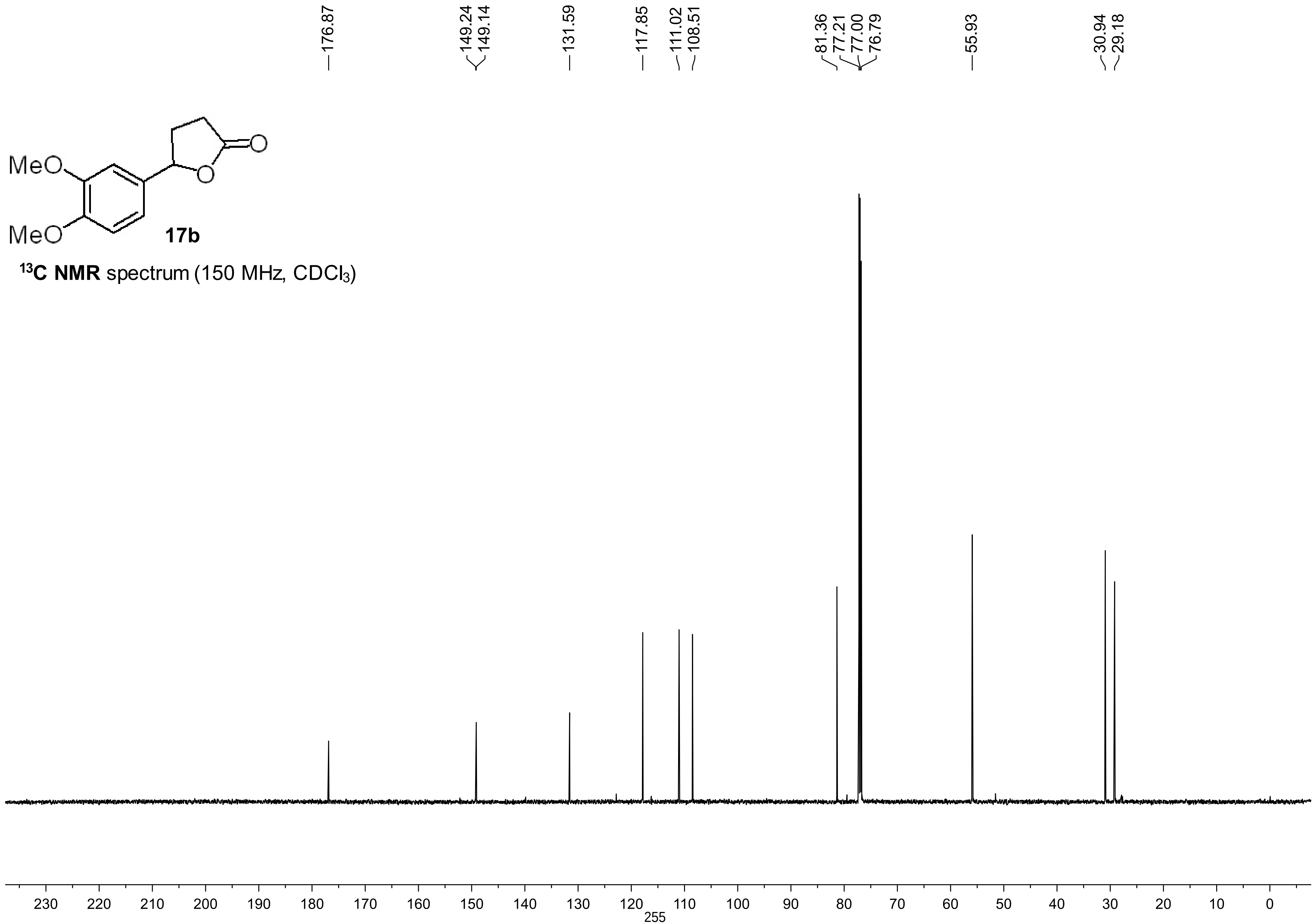

17b

^{13}C NMR spectrum (150 MHz, $CDCl_3$)

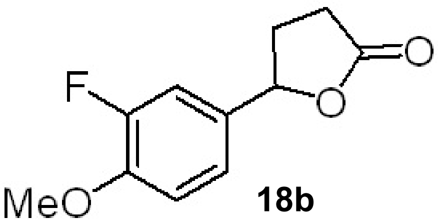

18b

^{1}H NMR spectrum (400 MHz, $CDCl_3$)

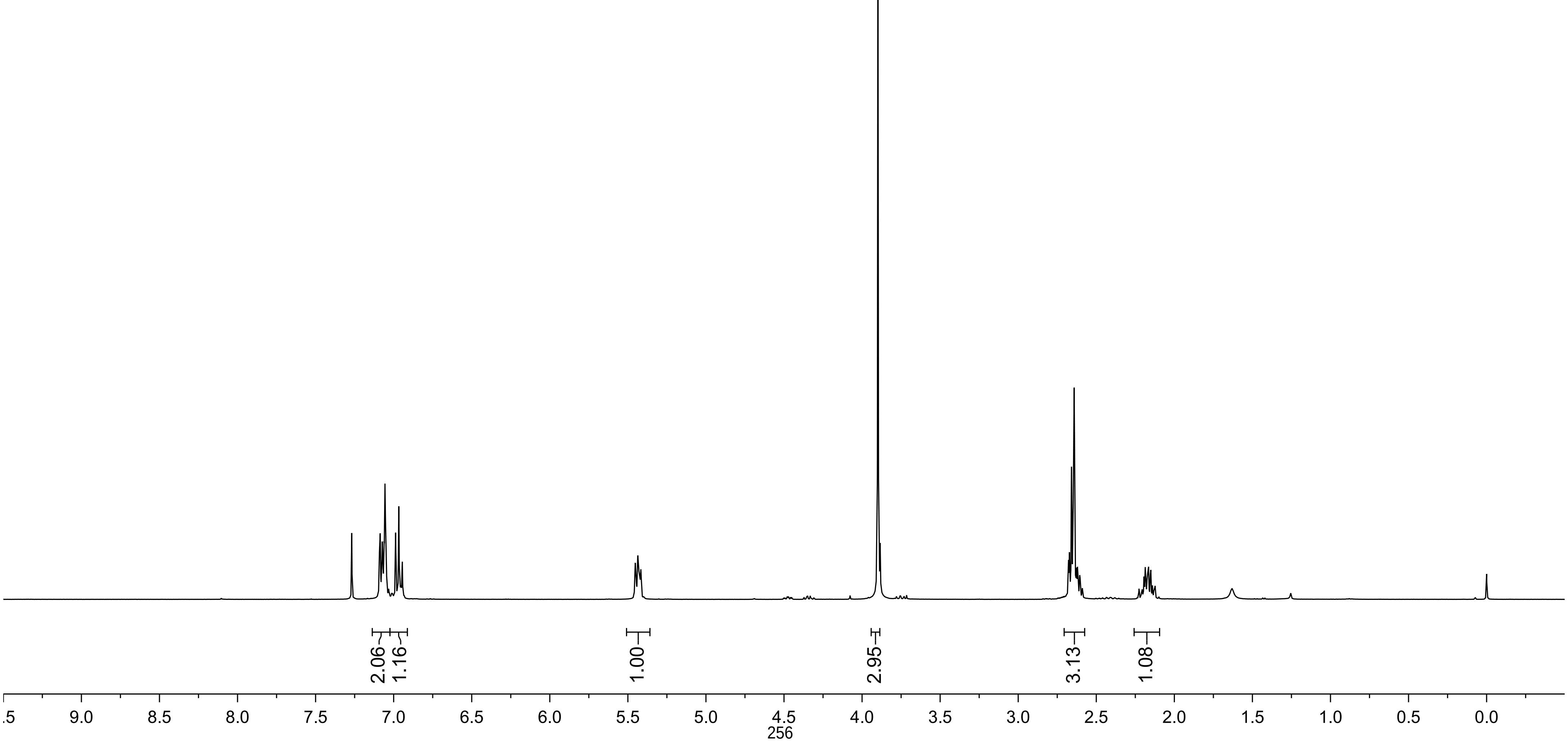

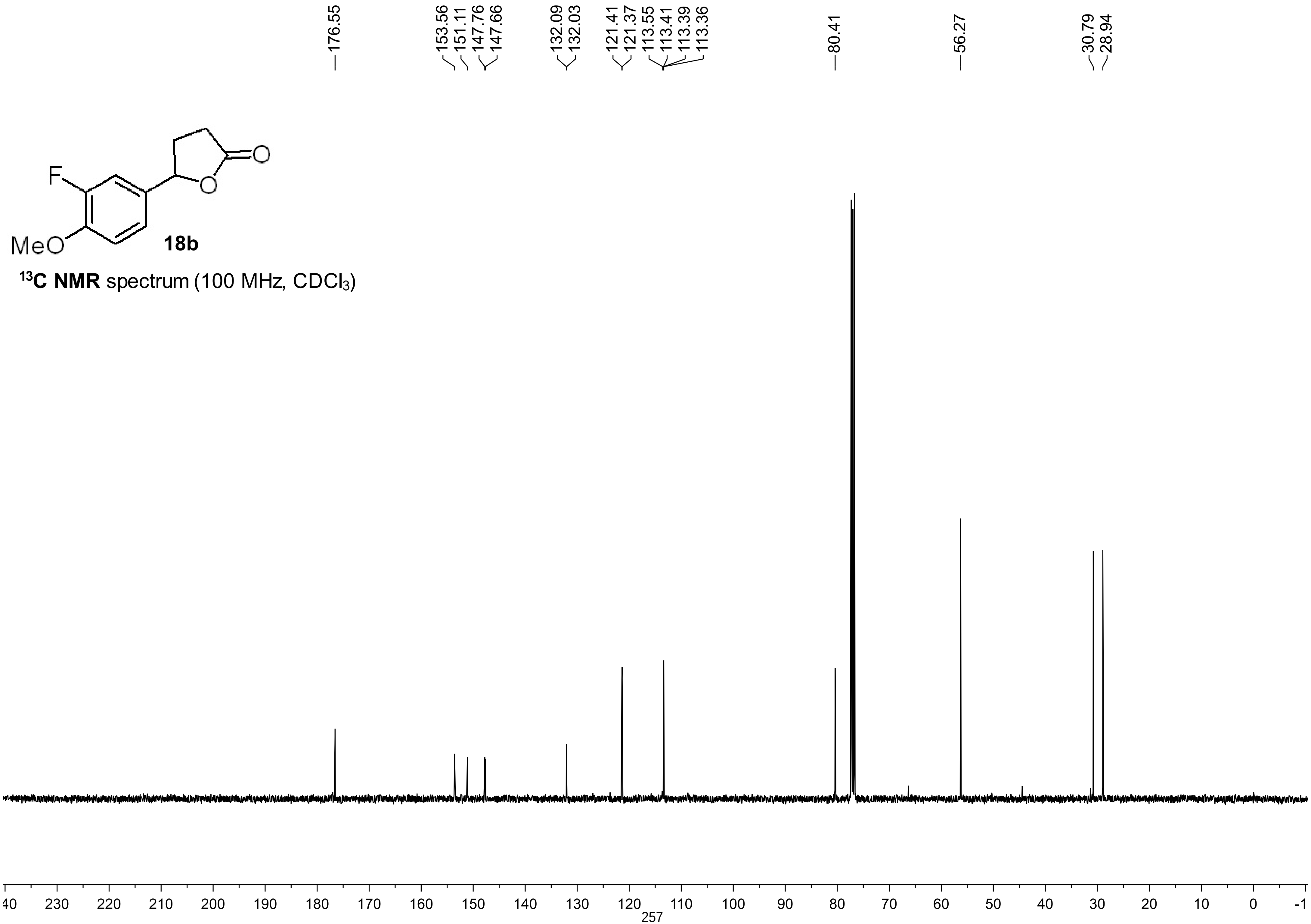

18b

^{13}C NMR spectrum (100 MHz, $CDCl_3$)

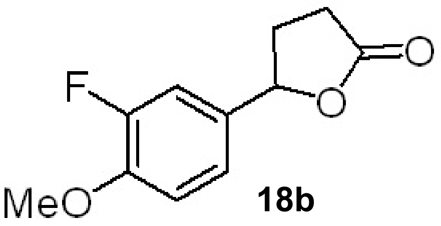

18b

^{19}F NMR spectrum (376 MHz, $CDCl_3$)

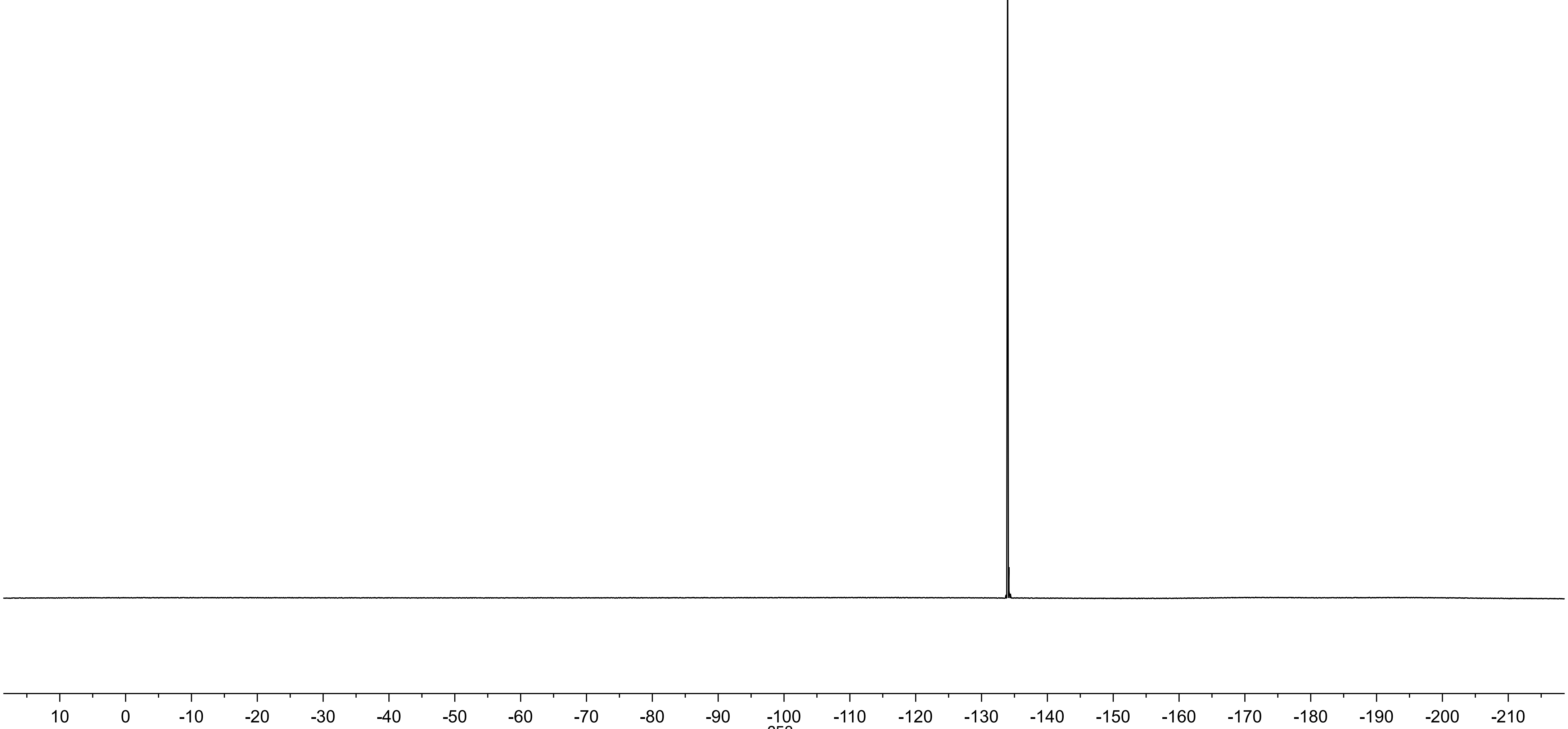

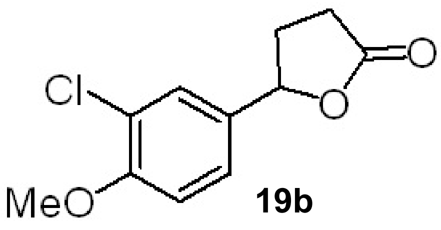

19b

^{1}H NMR spectrum (400 MHz, $CDCl_3$)

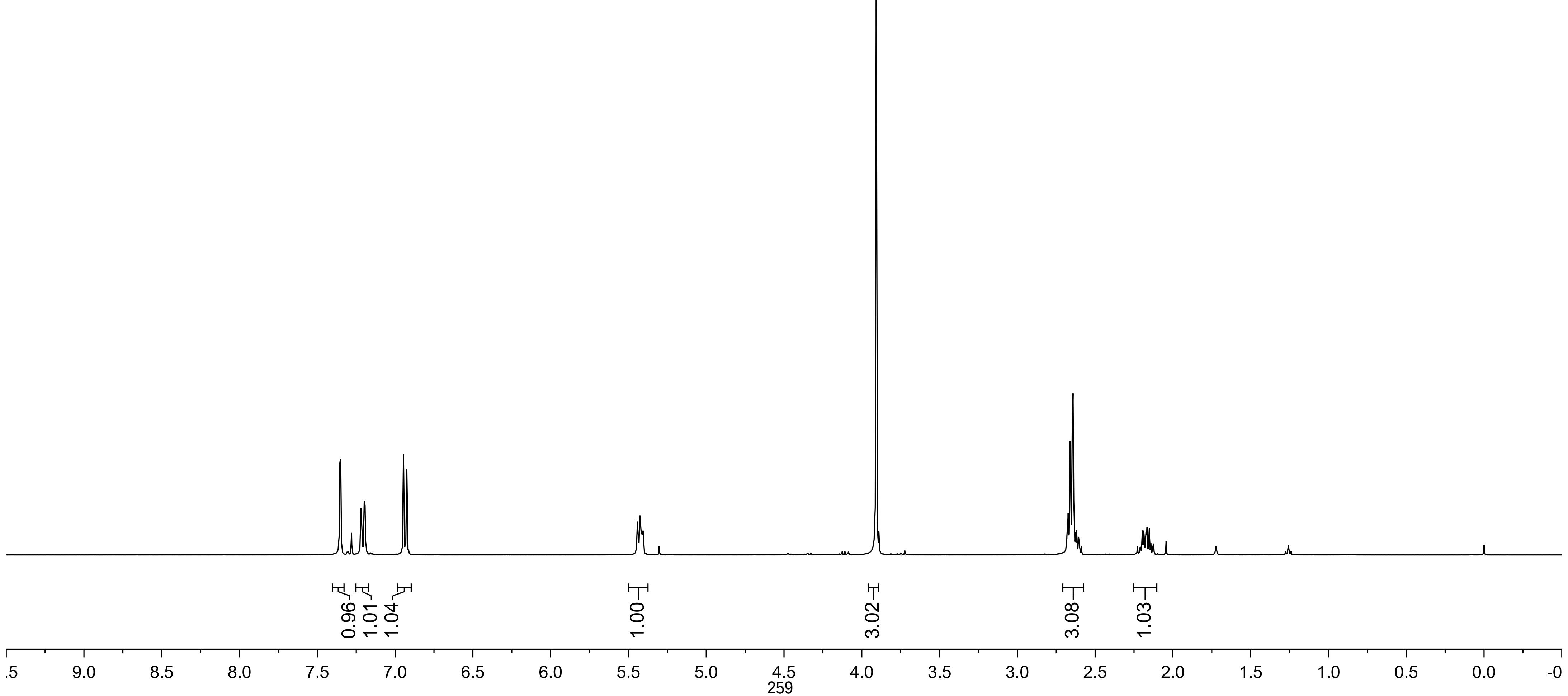

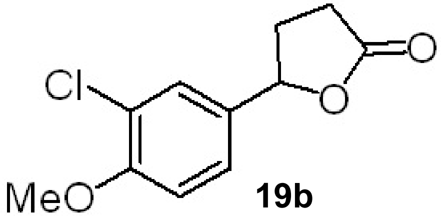

19b

^{13}C NMR spectrum (100 MHz, $CDCl_3$)

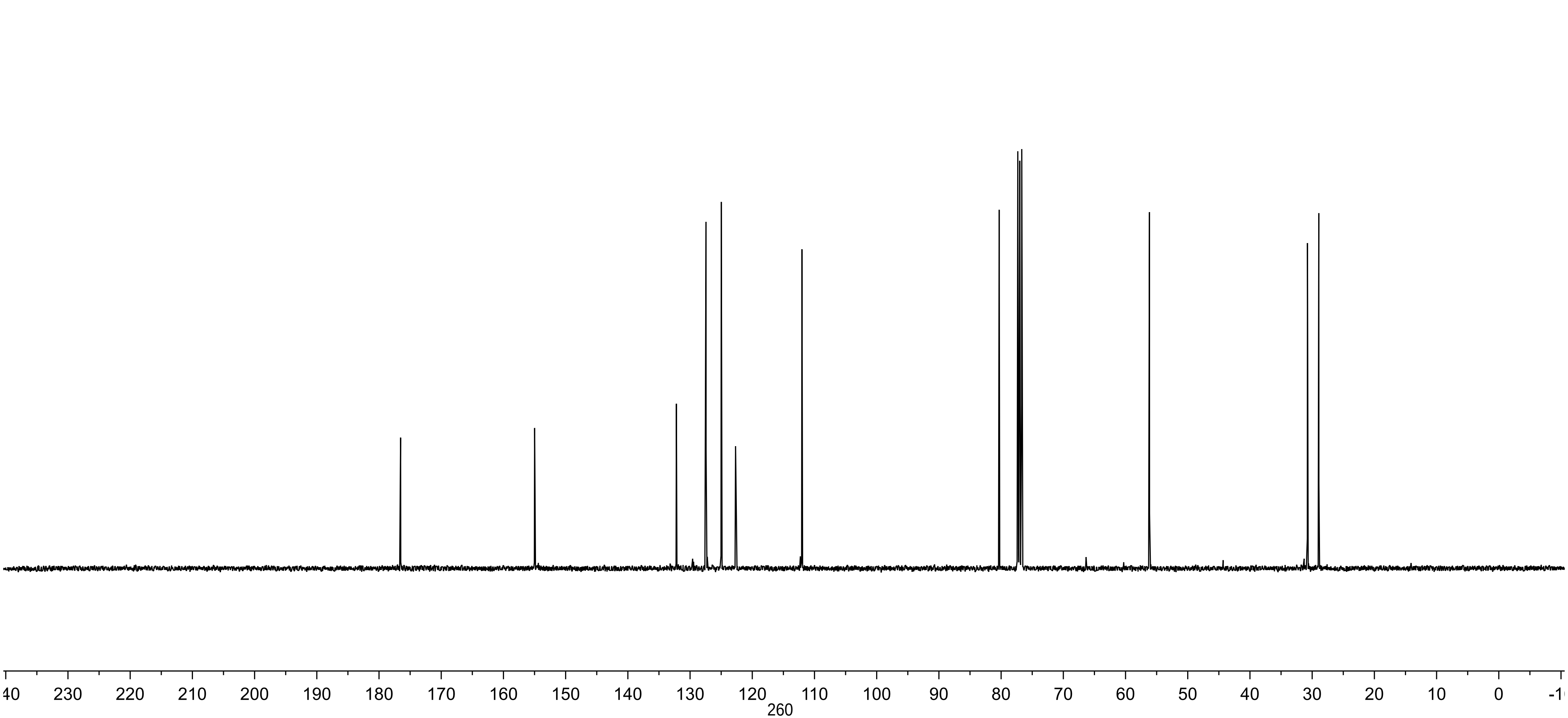

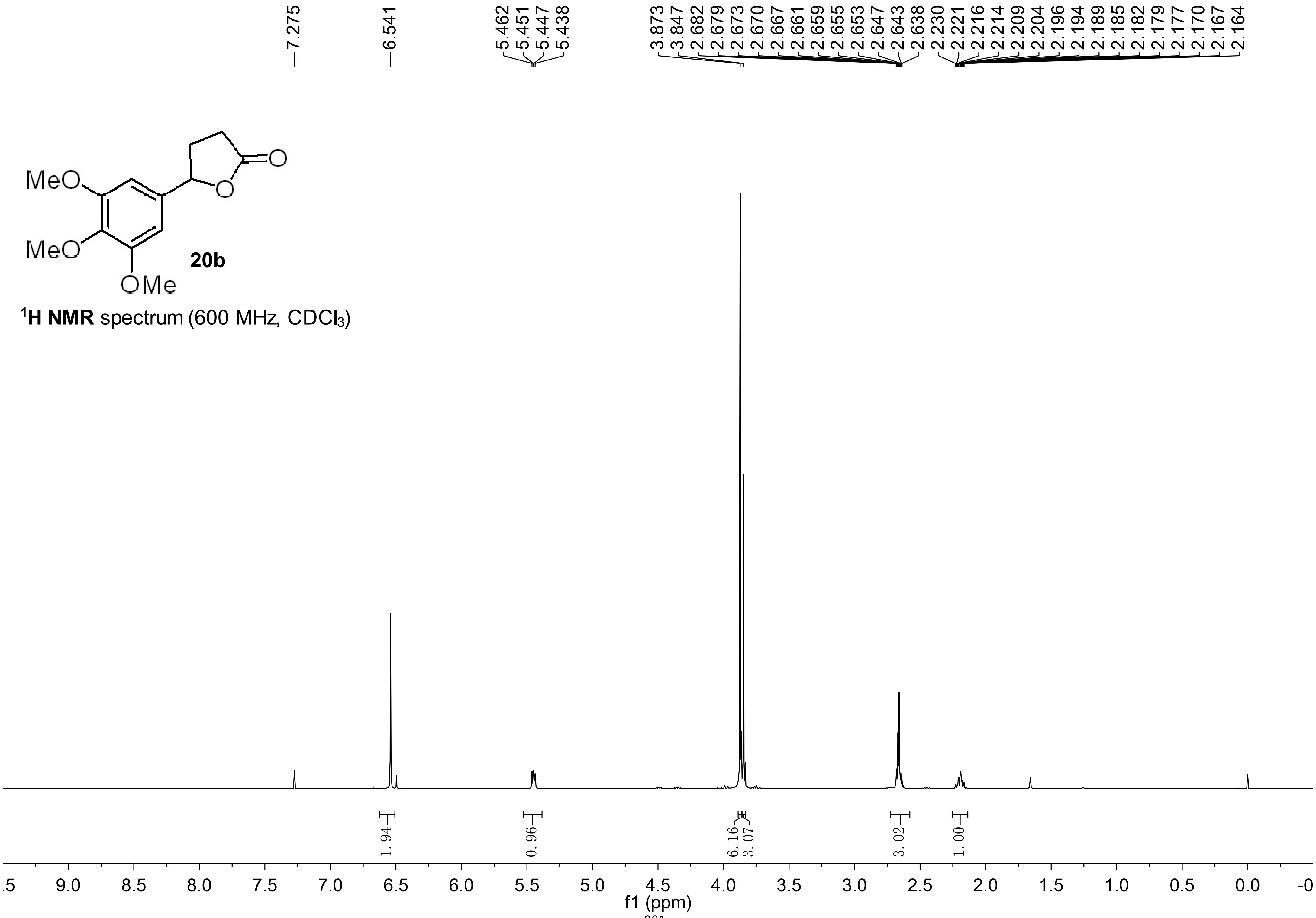
7.275
6.541
5.462
5.451
5.447
5.438
3.873
3.847
2.682
2.679
2.673
2.670
2.667
2.661
2.659
2.655
2.653
2.647
2.643
2.638
2.230
2.221
2.216
2.214
2.209
2.204
2.196
2.194
2.189
2.185
2.182
2.179
2.177
2.170
2.167
2.164
MeO
MeO
OMe
O
O
20b
1H NMR spectrum (600 MHz, CDCl3)
1.94
0.96
6.16
3.07
3.02
1.00
f1 (ppm)

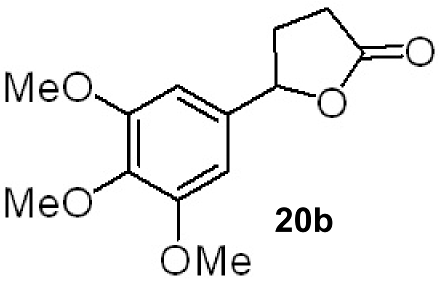

20b

^{13}C NMR spectrum (150 MHz, $CDCl_3$)

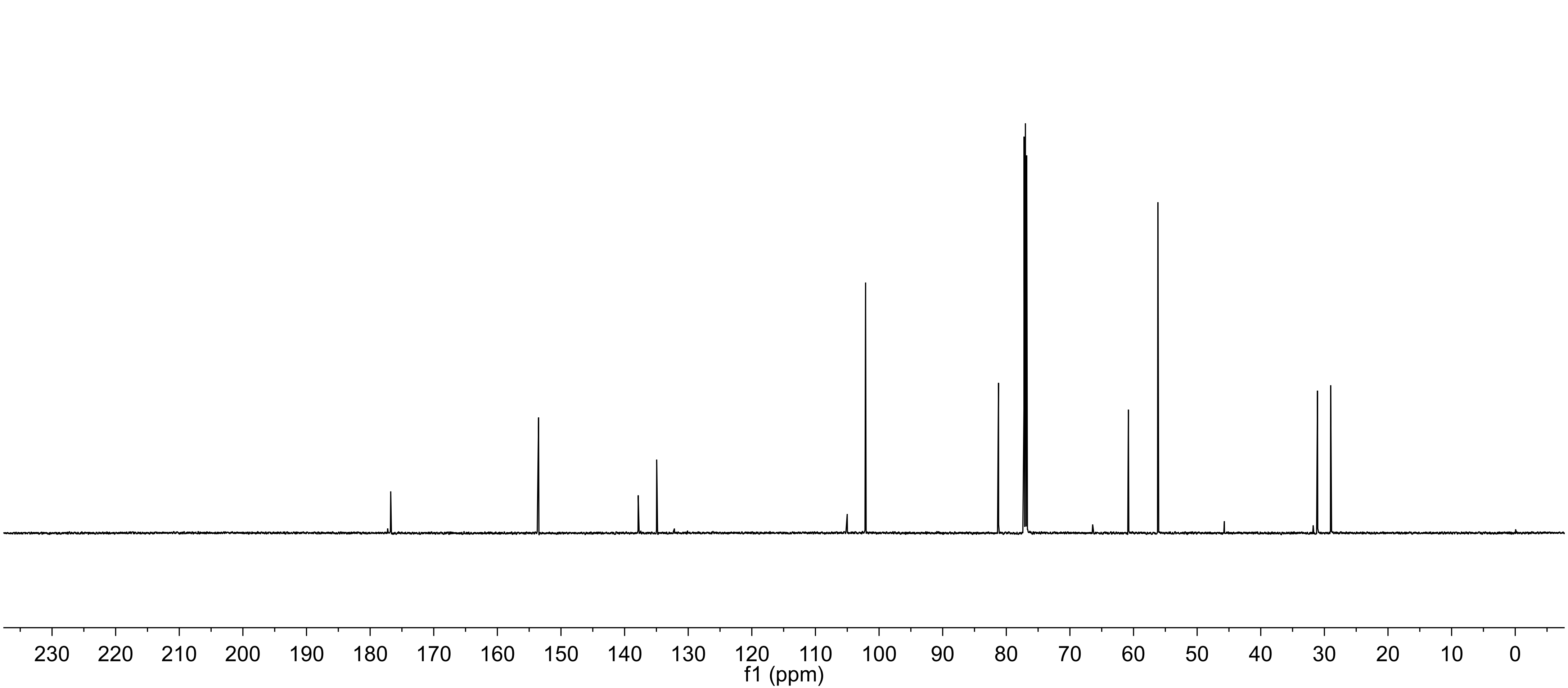

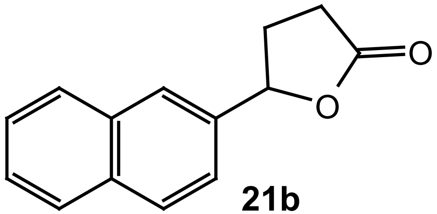

21b

^{1}H NMR spectrum (400 MHz, $CDCl_3$)

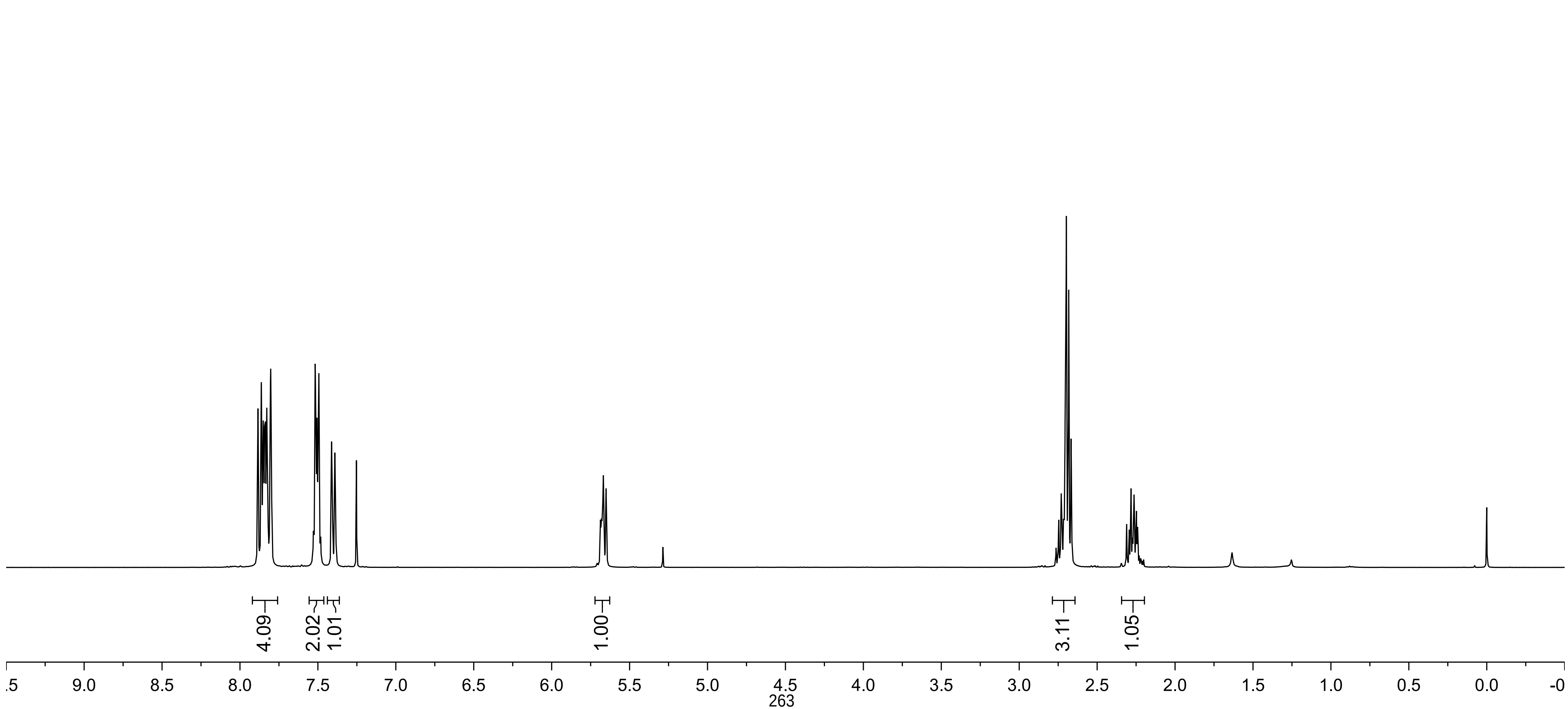

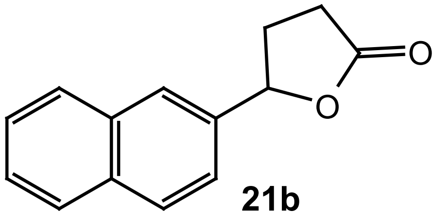

21b

^{13}C NMR spectrum (100 MHz, $CDCl_3$)

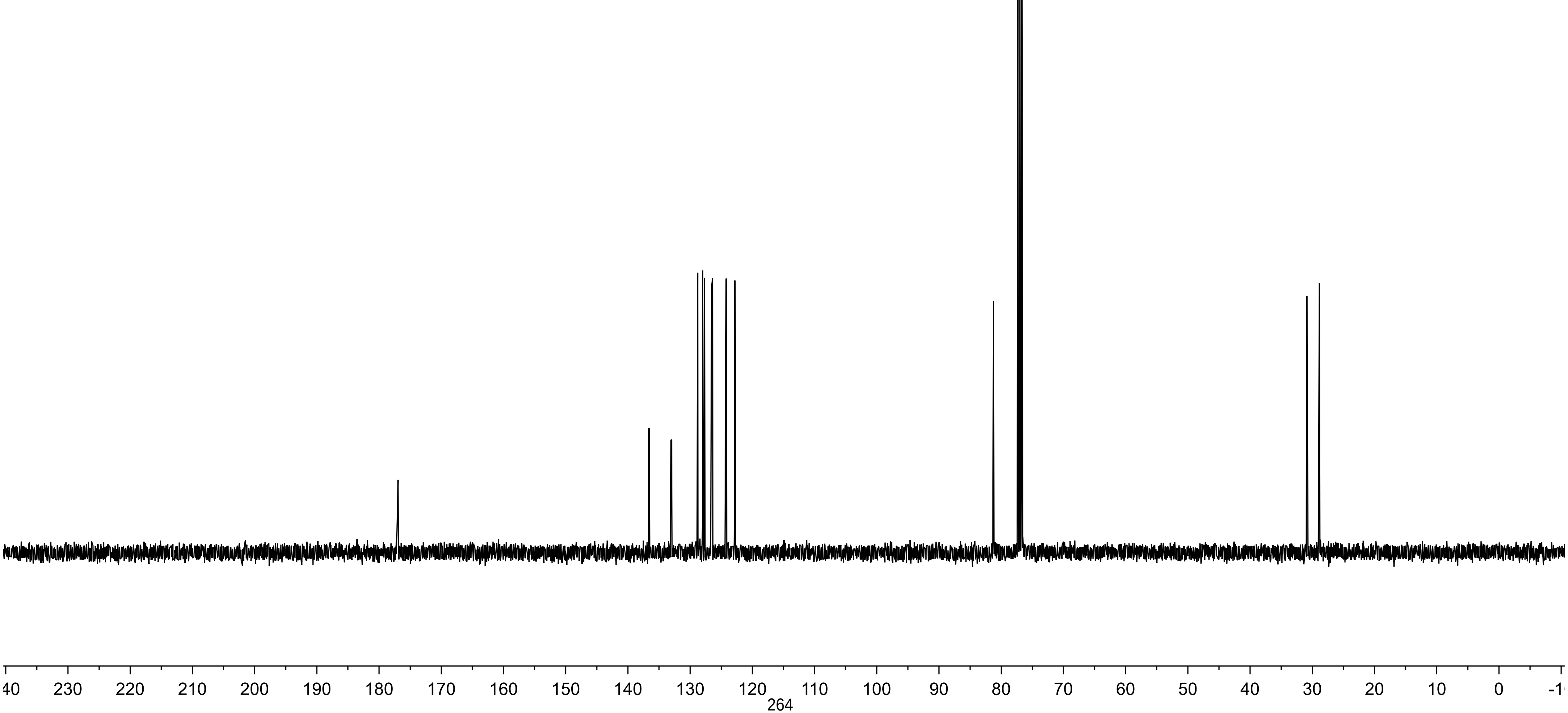

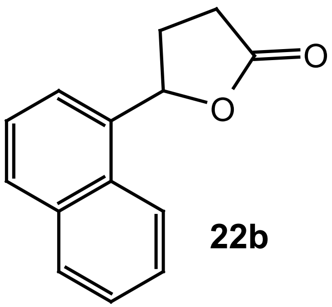

22b

^{1}H NMR spectrum (400 MHz, $CDCl_3$)

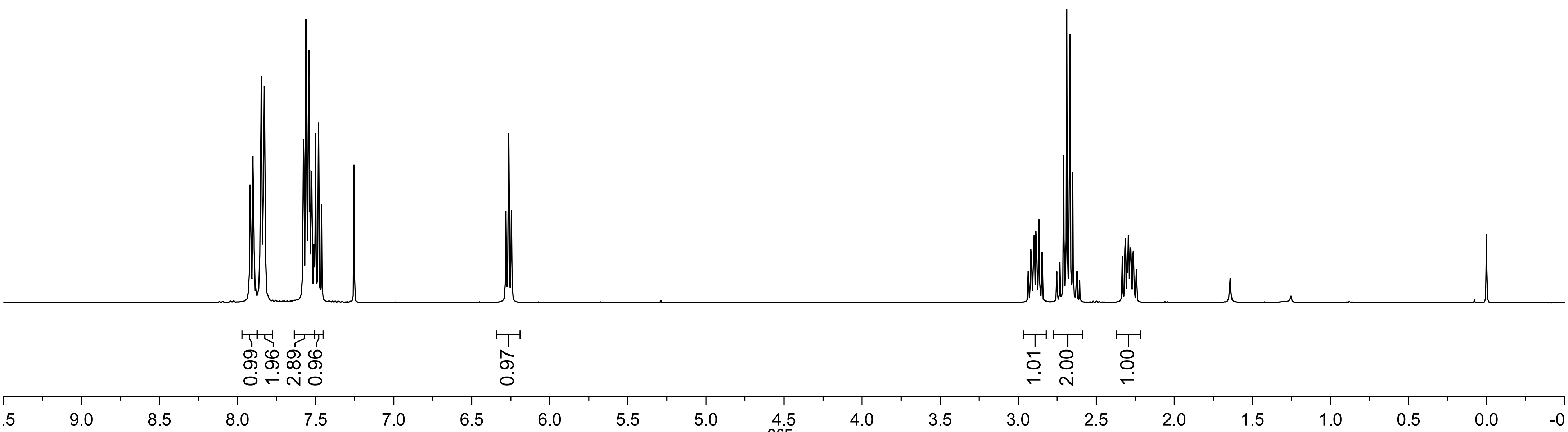

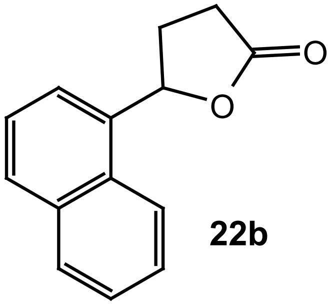

22b

^{13}C NMR spectrum (100 MHz, $CDCl_3$)

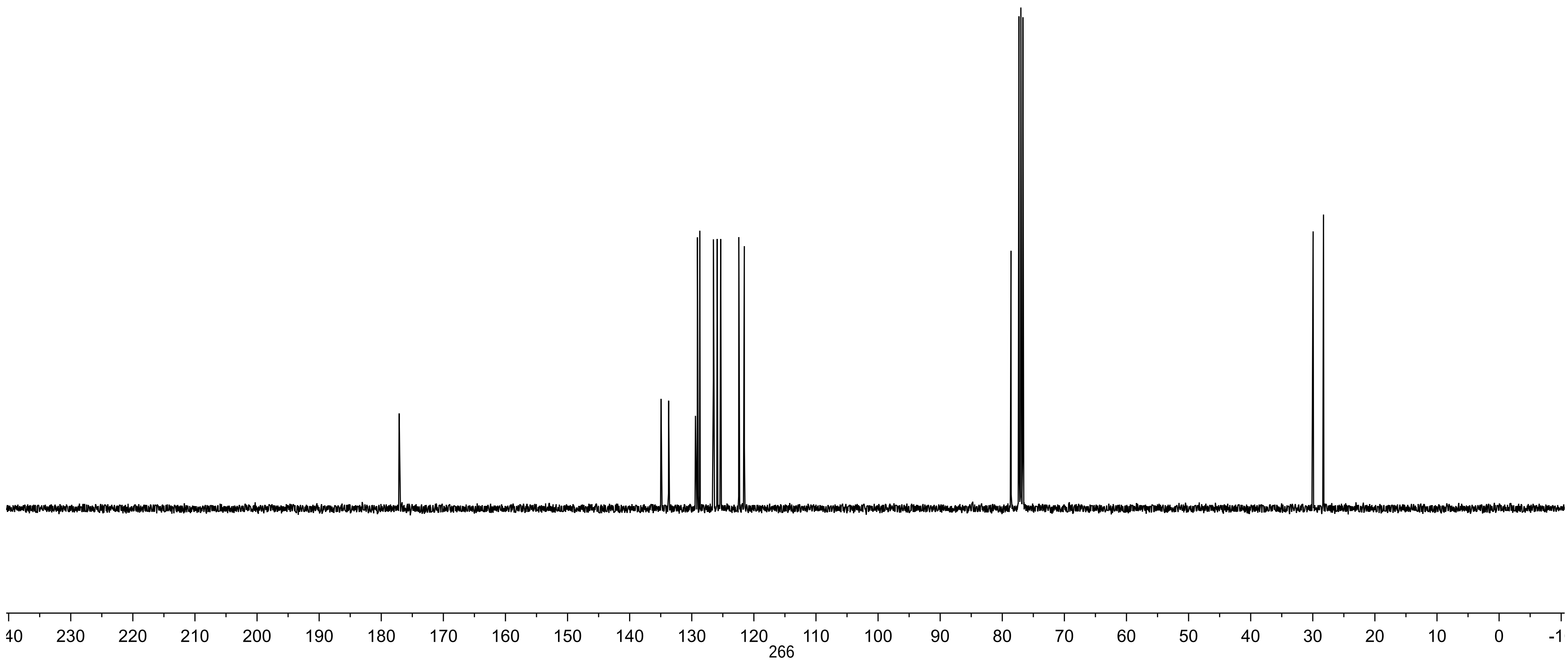

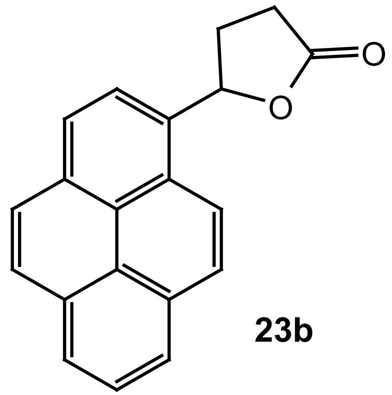

23b

^{1}H NMR spectrum (400 MHz, $CDCl_3$)

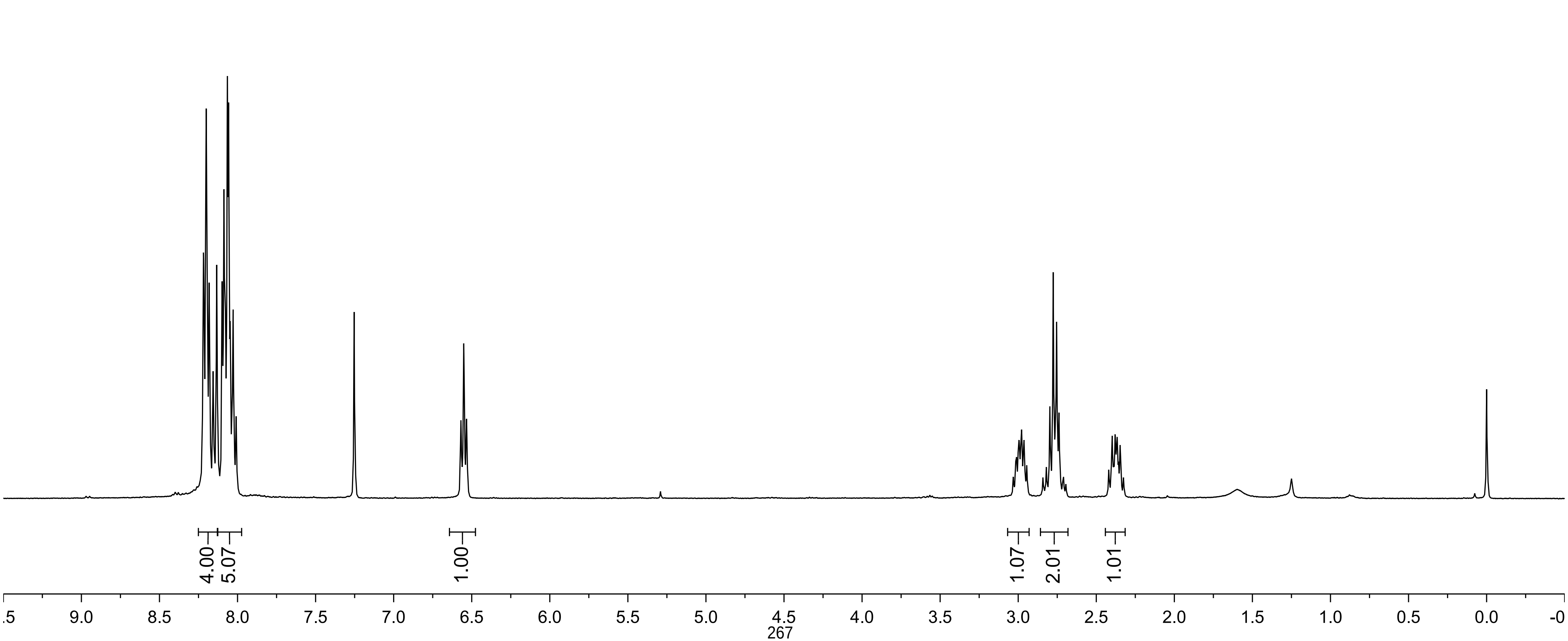

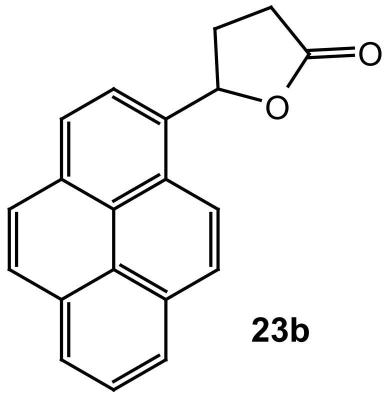

23b

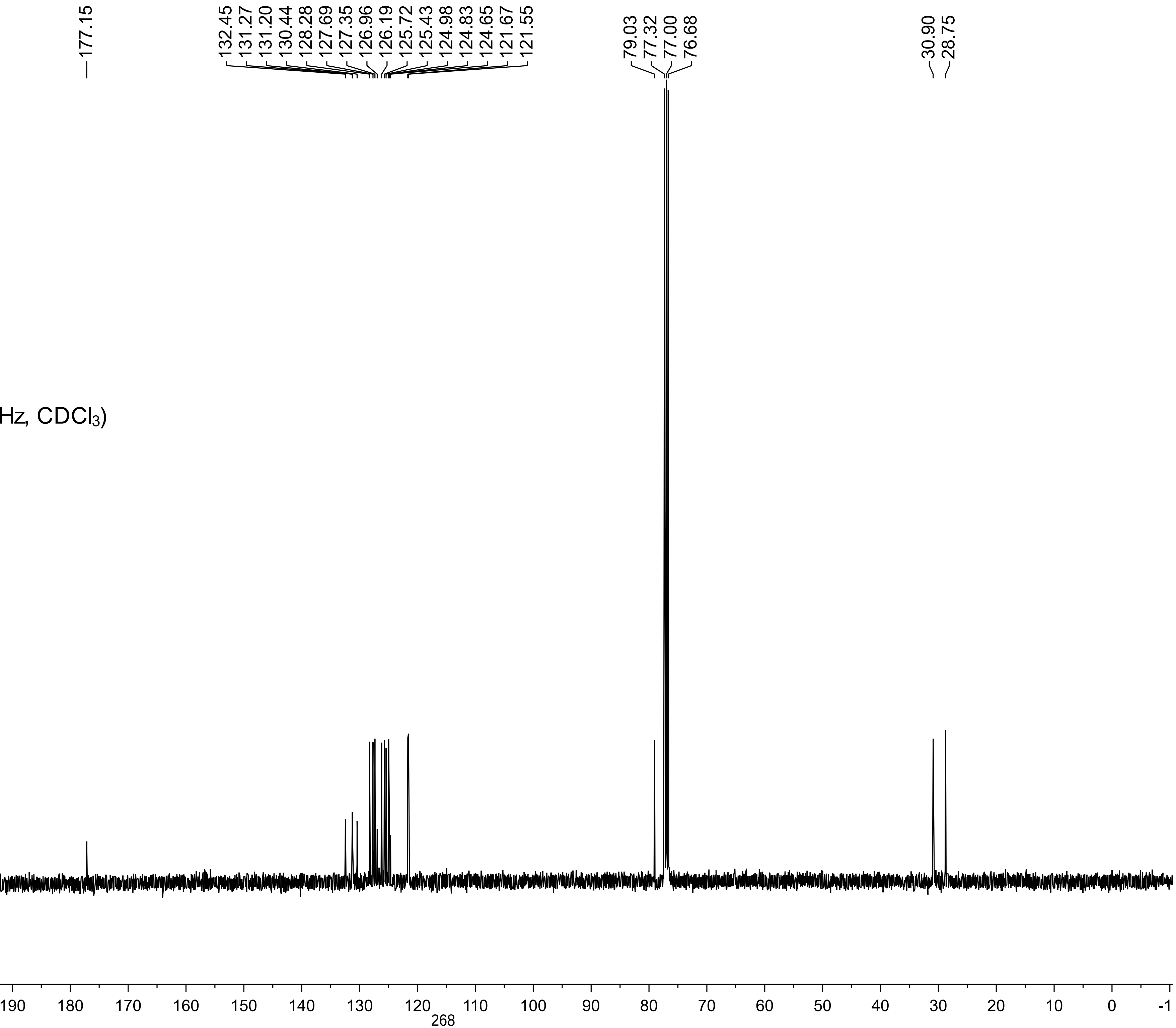

^{13}C NMR spectrum (100 MHz, $CDCl_3$)

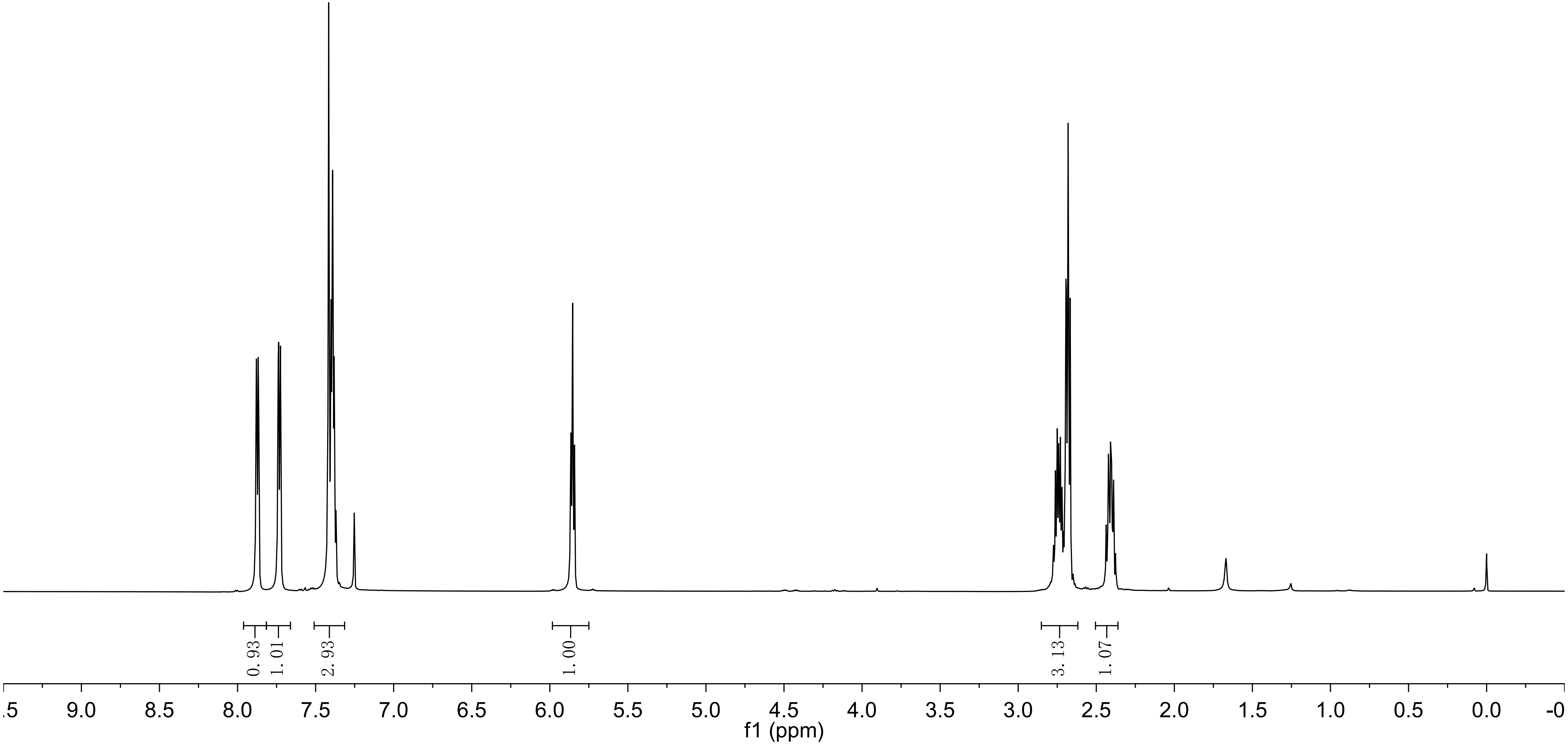

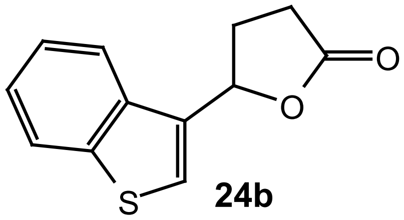

24b

^{1}H NMR spectrum (600 MHz, $CDCl_3$)

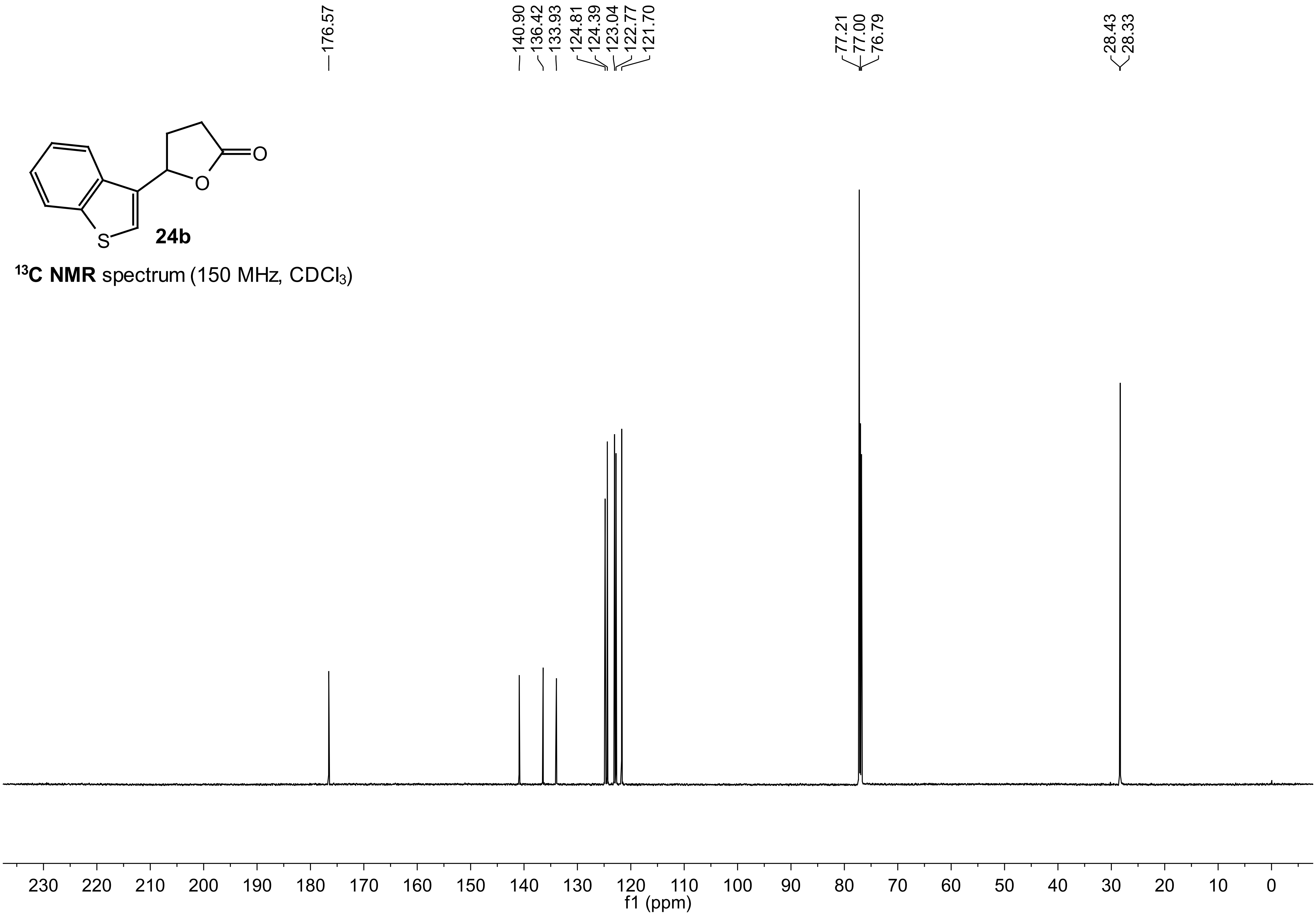
176.57
140.90
136.42
133.93
124.81
124.39
123.04
122.77
121.70
77.21
77.00
76.79
28.43
28.33
O
O
S
24b
^{13}C NMR spectrum (150 MHz, CDCl$_3$)
230 220 210 200 190 180 170 160 150 140 130 120 110 100 90 80 70 60 50 40 30 20 10 0
f1 (ppm)

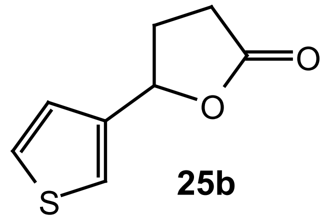

25b

^{1}H NMR spectrum (600 MHz, $CDCl_3$)

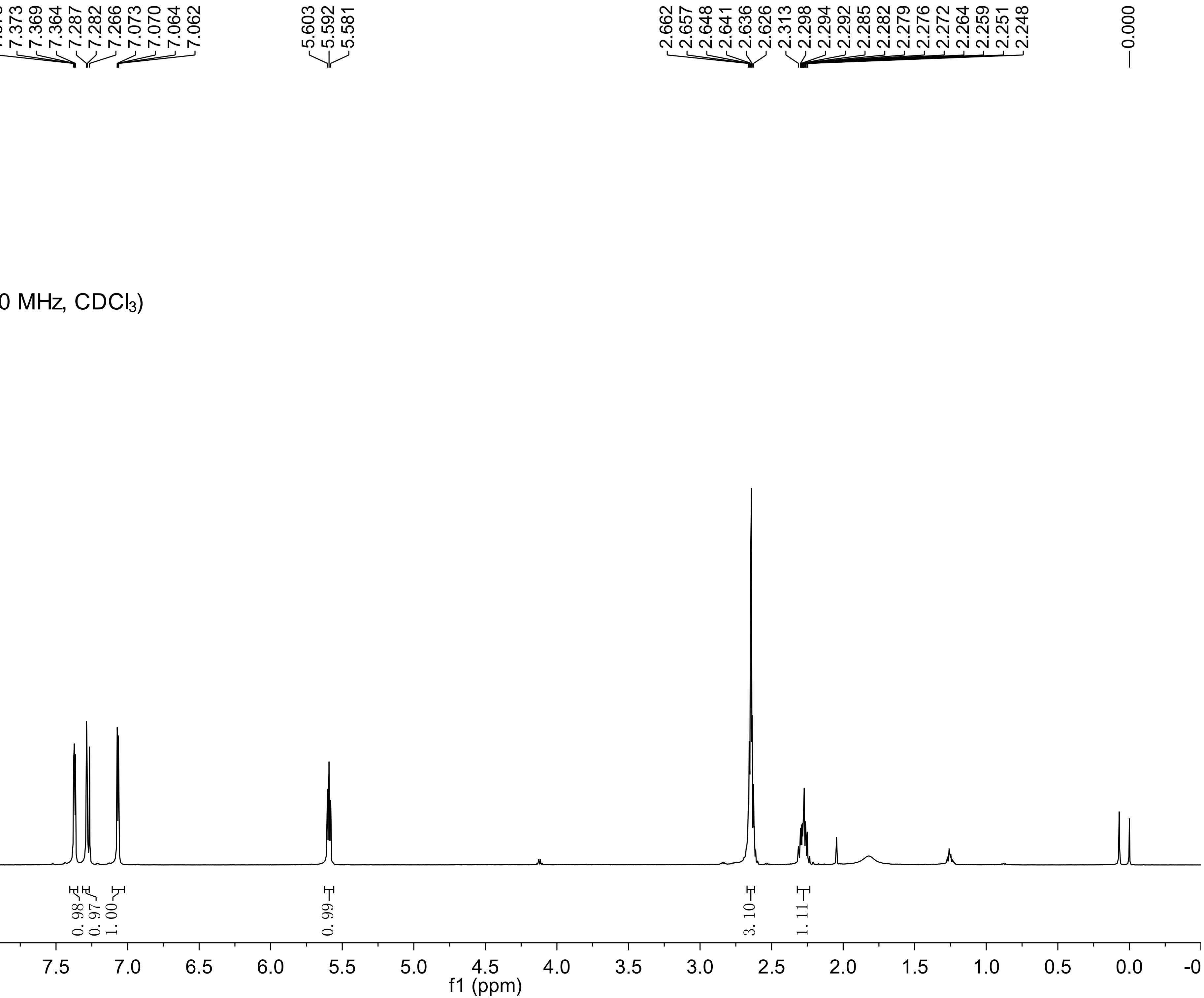

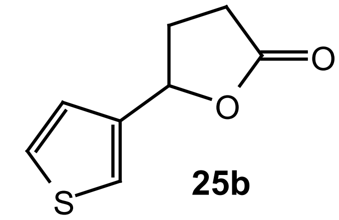

25b

^{13}C NMR spectrum (150 MHz, $CDCl_3$)

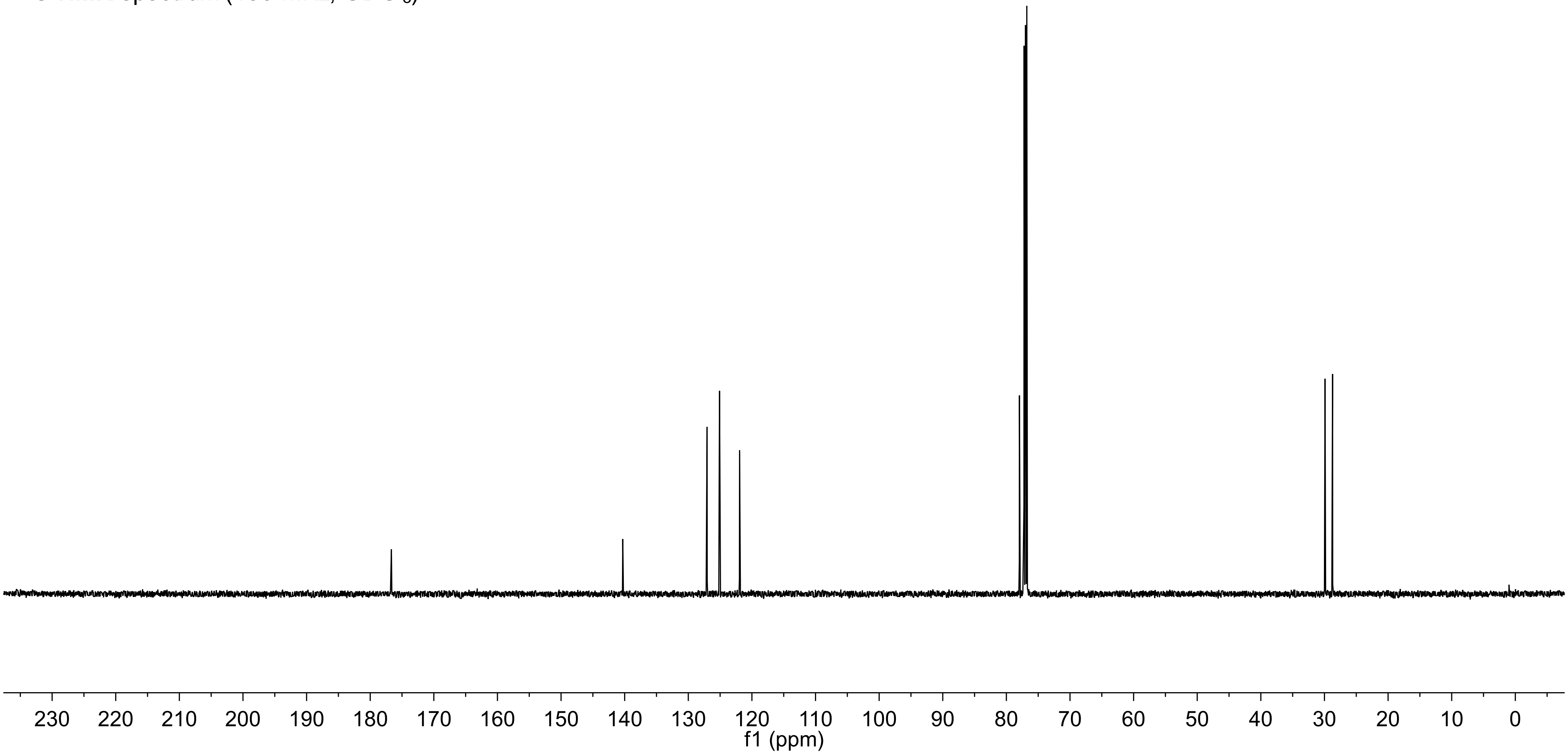

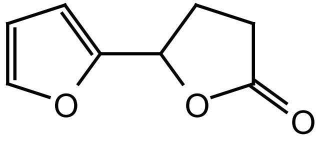

26b

^{1}H NMR spectrum (600 MHz, $CDCl_3$)

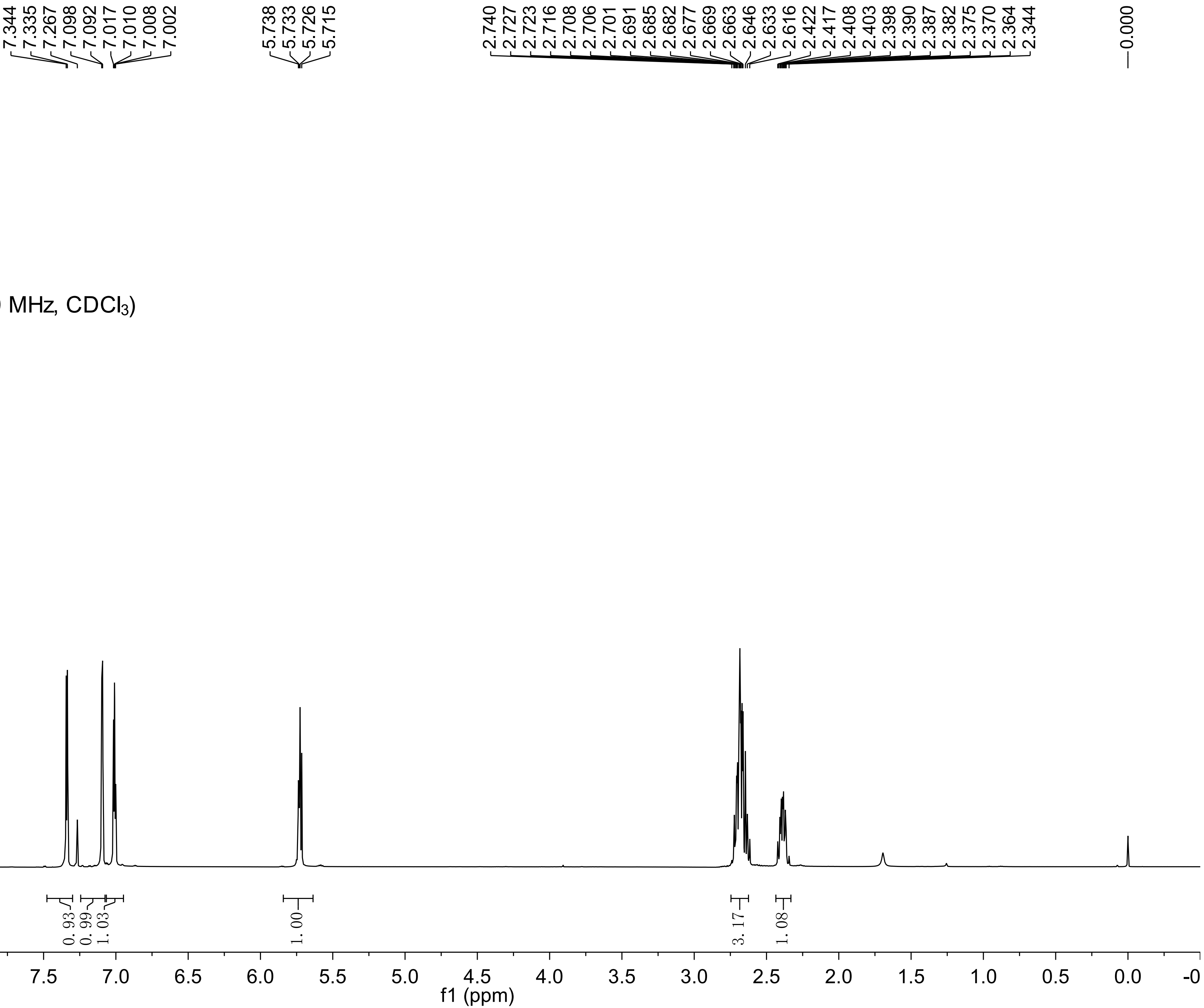

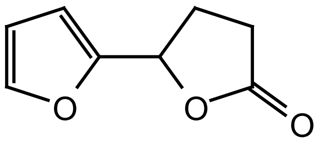

26b

^{13}C NMR spectrum (150 MHz, $CDCl_3$)

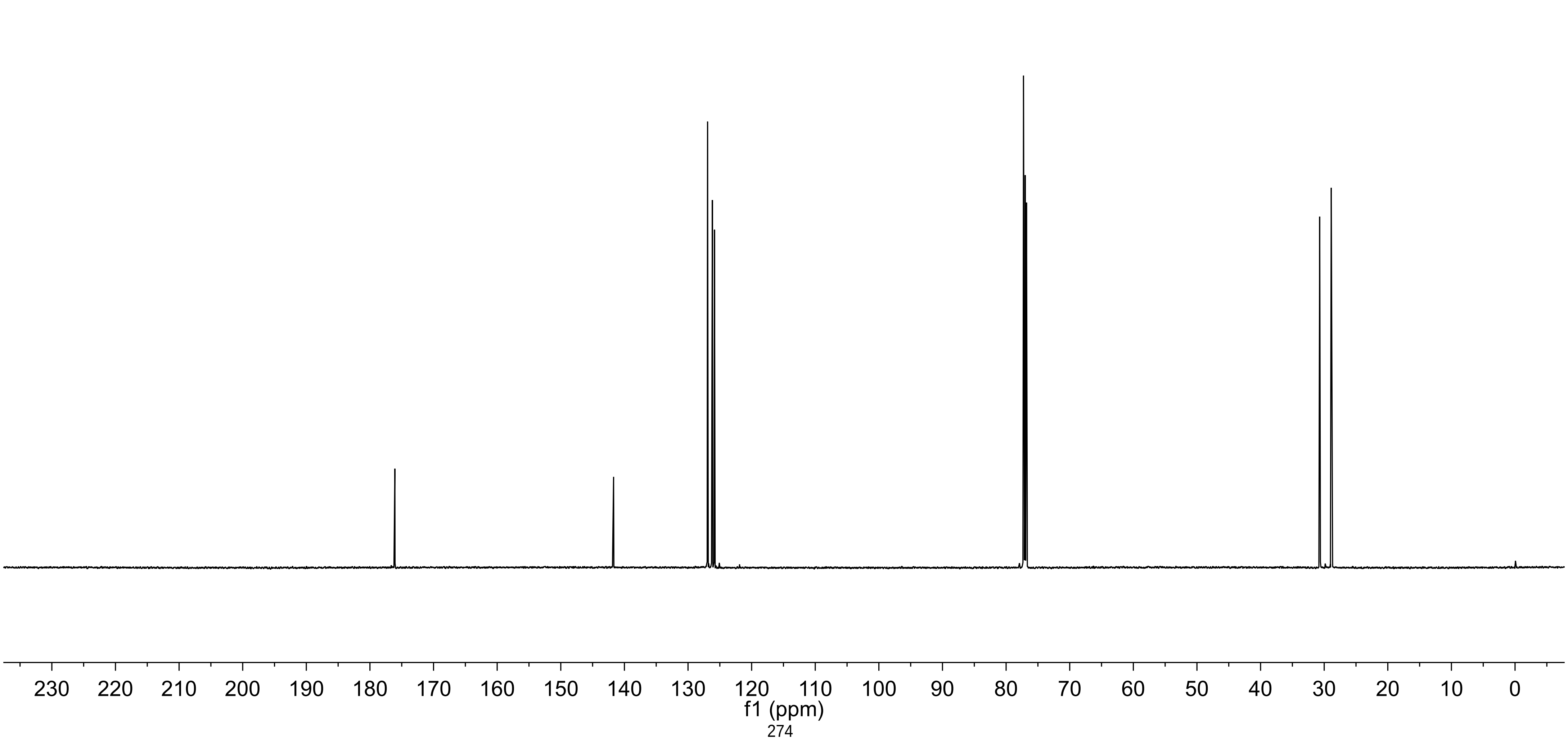

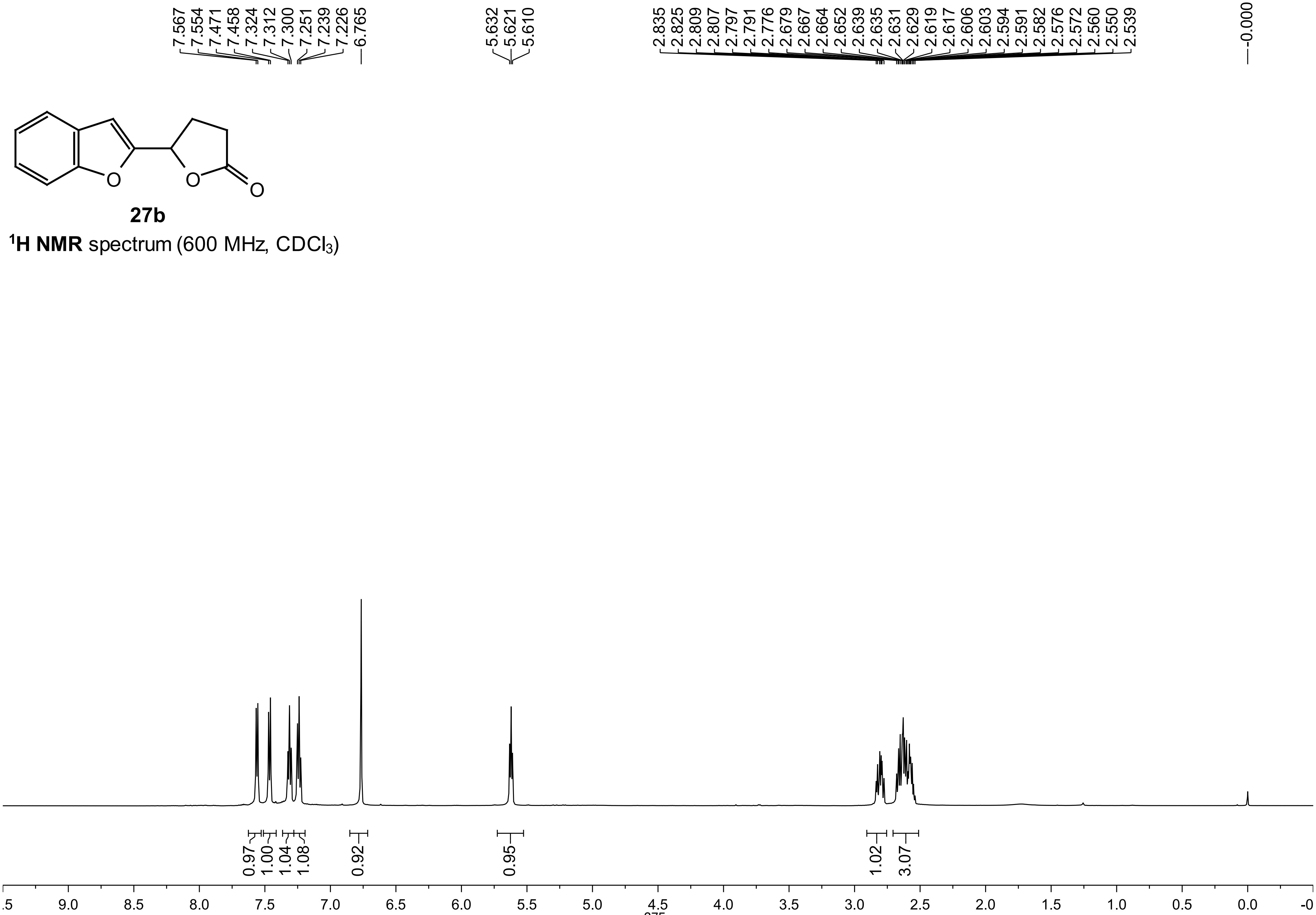

27b

^{1}H NMR spectrum (600 MHz, $CDCl_3$)

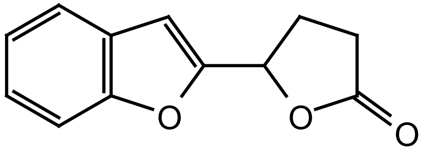

27b

^{13}C NMR spectrum (150 MHz, $CDCl_3$)

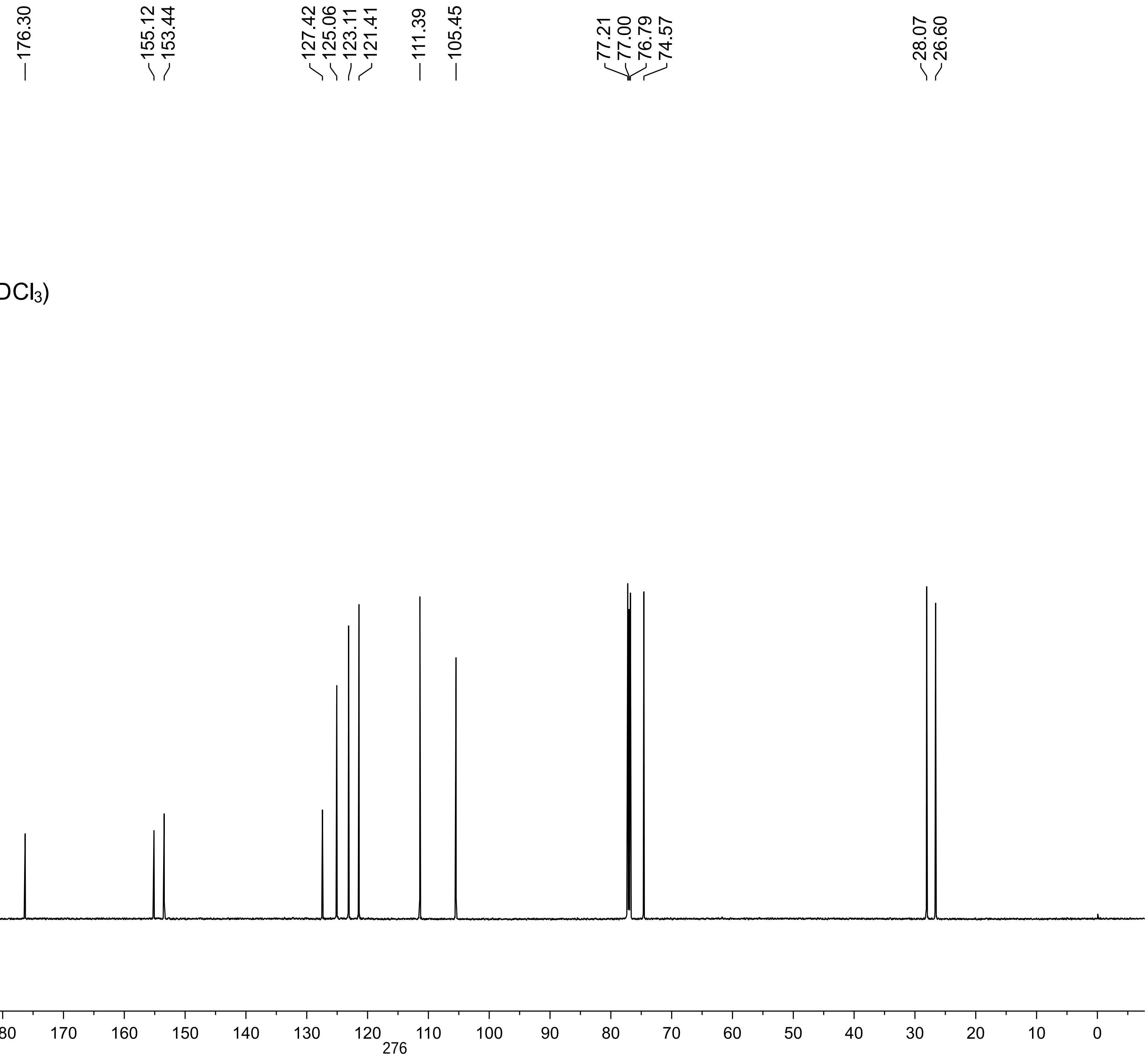

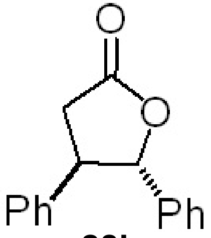

28b

^{1}H NMR spectrum (600 MHz, $CDCl_3$)

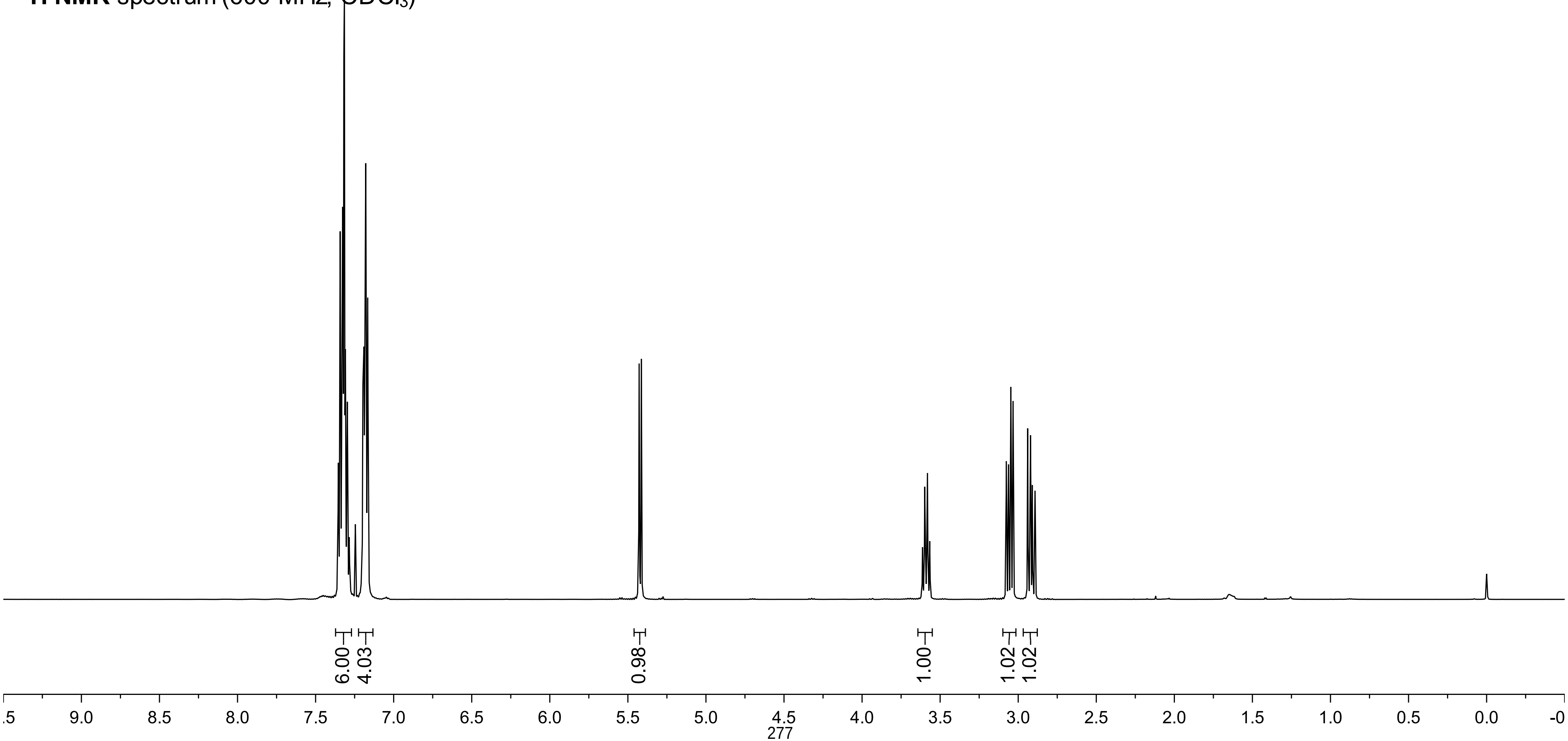

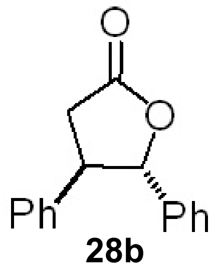

28b

^{13}C NMR spectrum (150 MHz, $CDCl_3$)

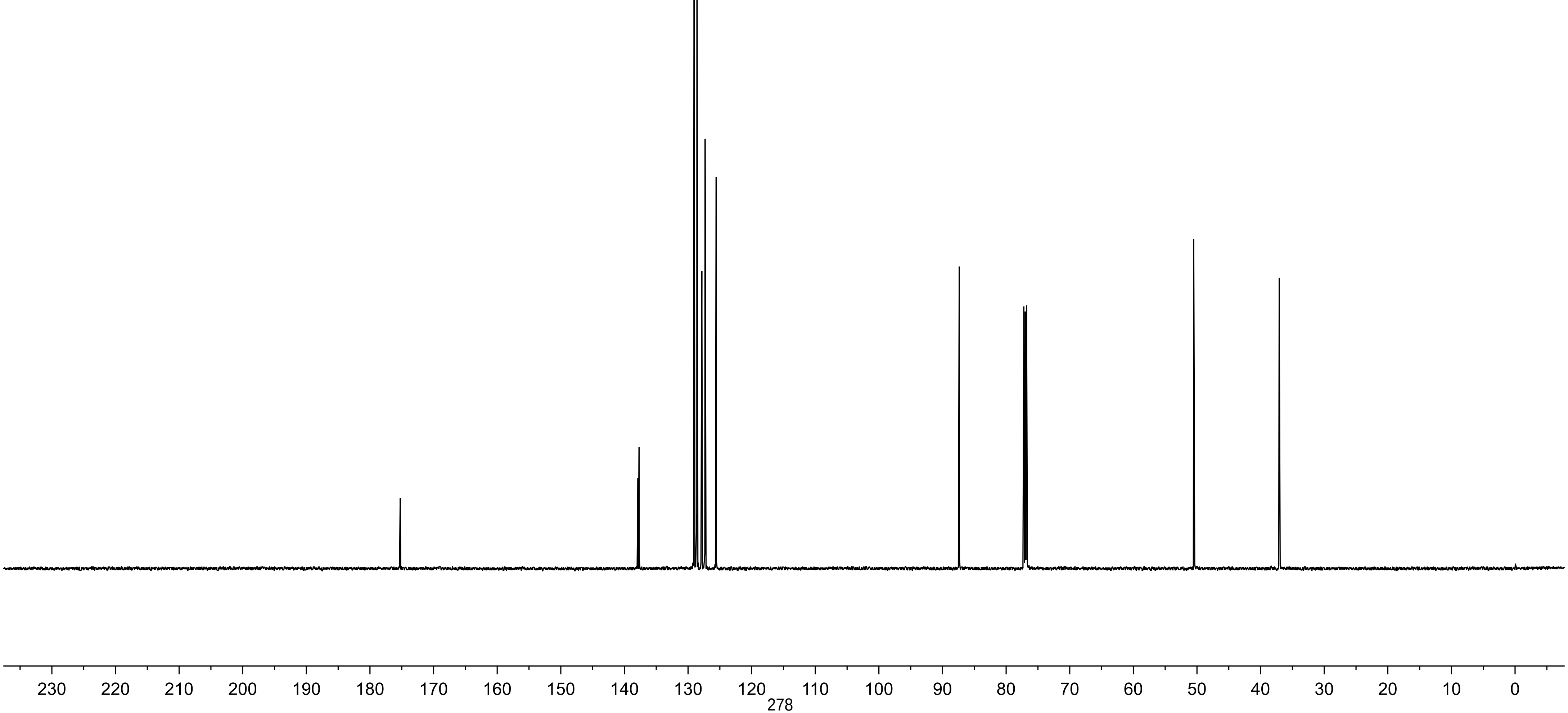

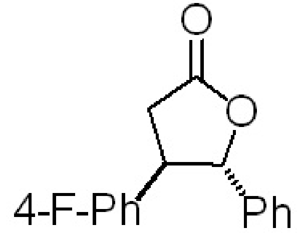

29b

^{1}H NMR spectrum (600 MHz, $CDCl_3$)

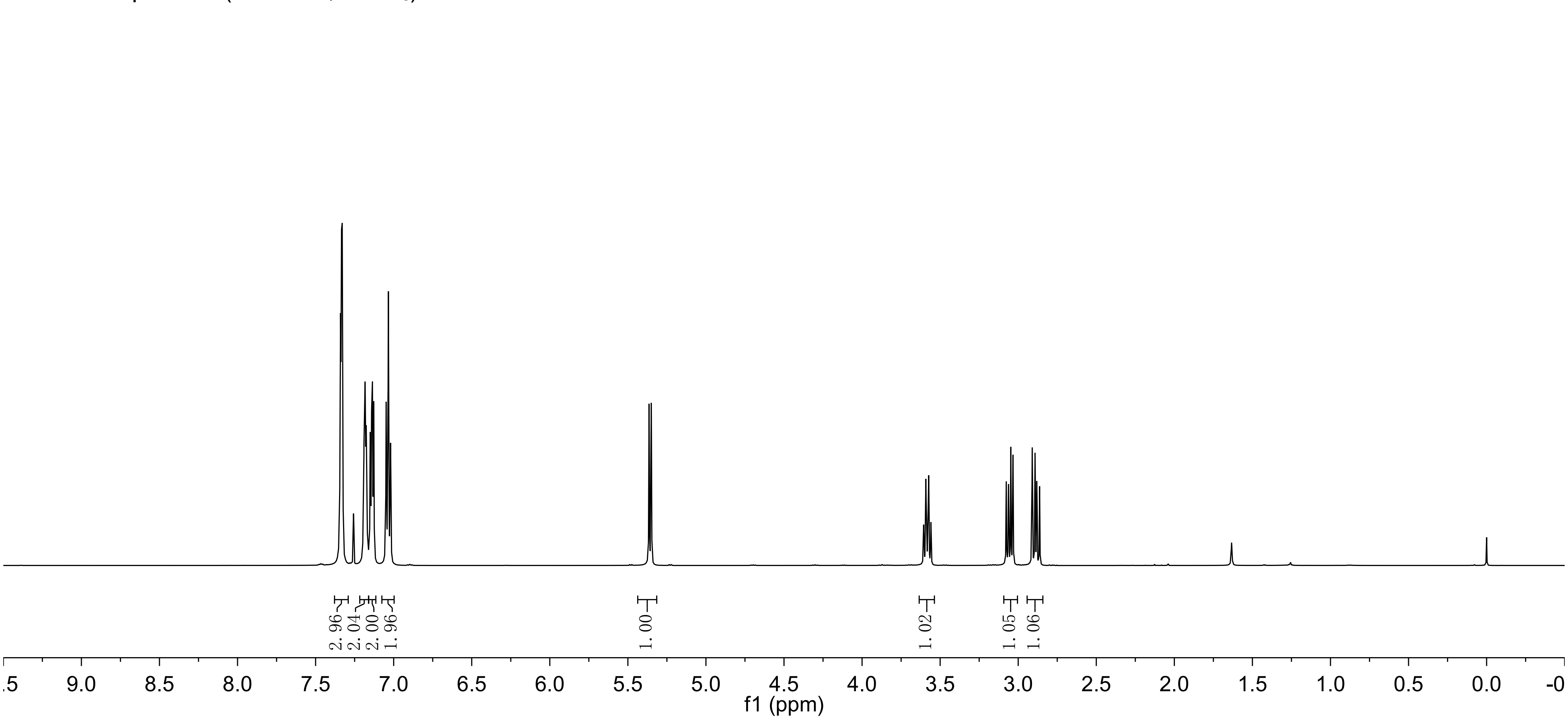

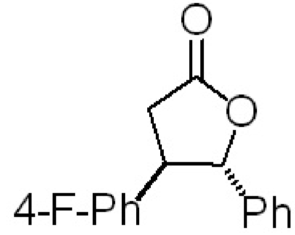

29b

^{13}C NMR spectrum (150 MHz, $CDCl_3$)

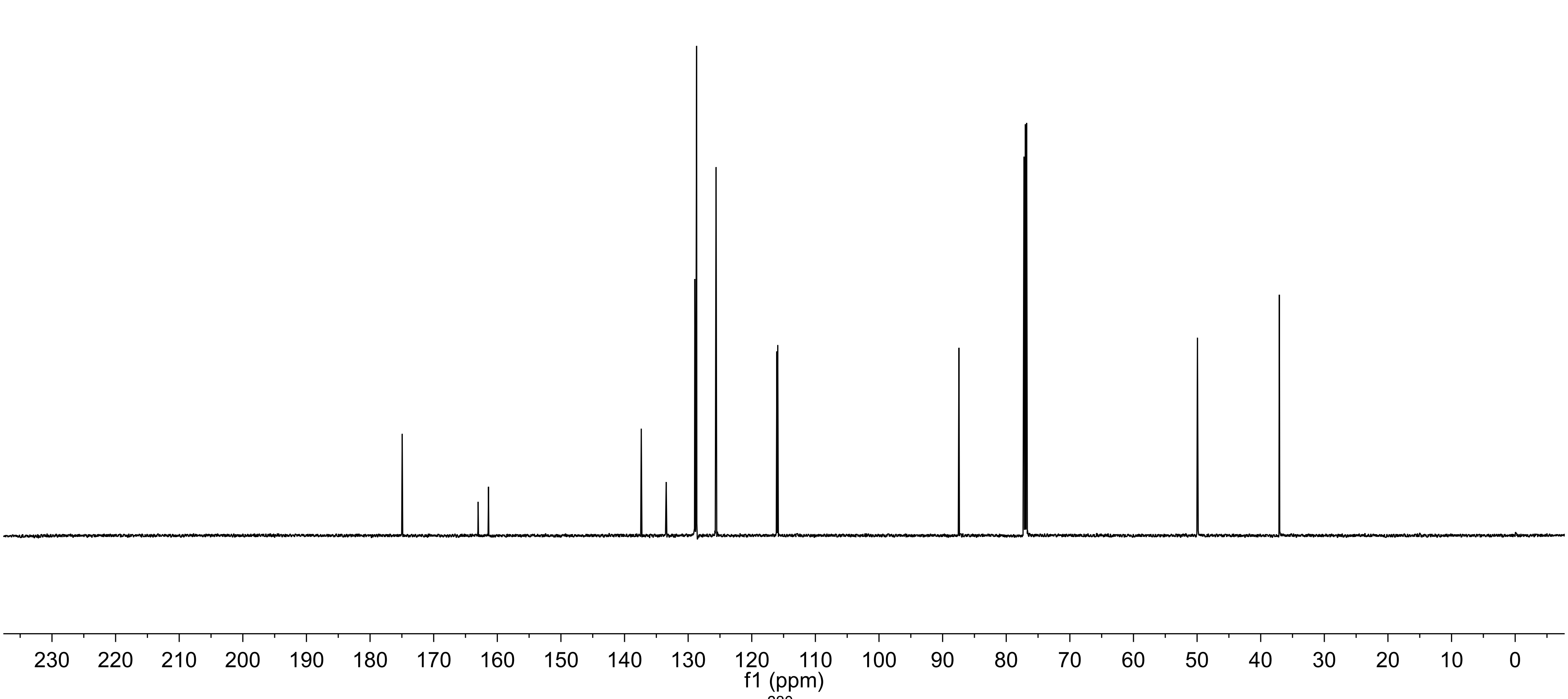

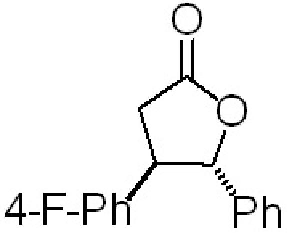

29b

^{19}F NMR spectrum (376 MHz, $CDCl_3$)

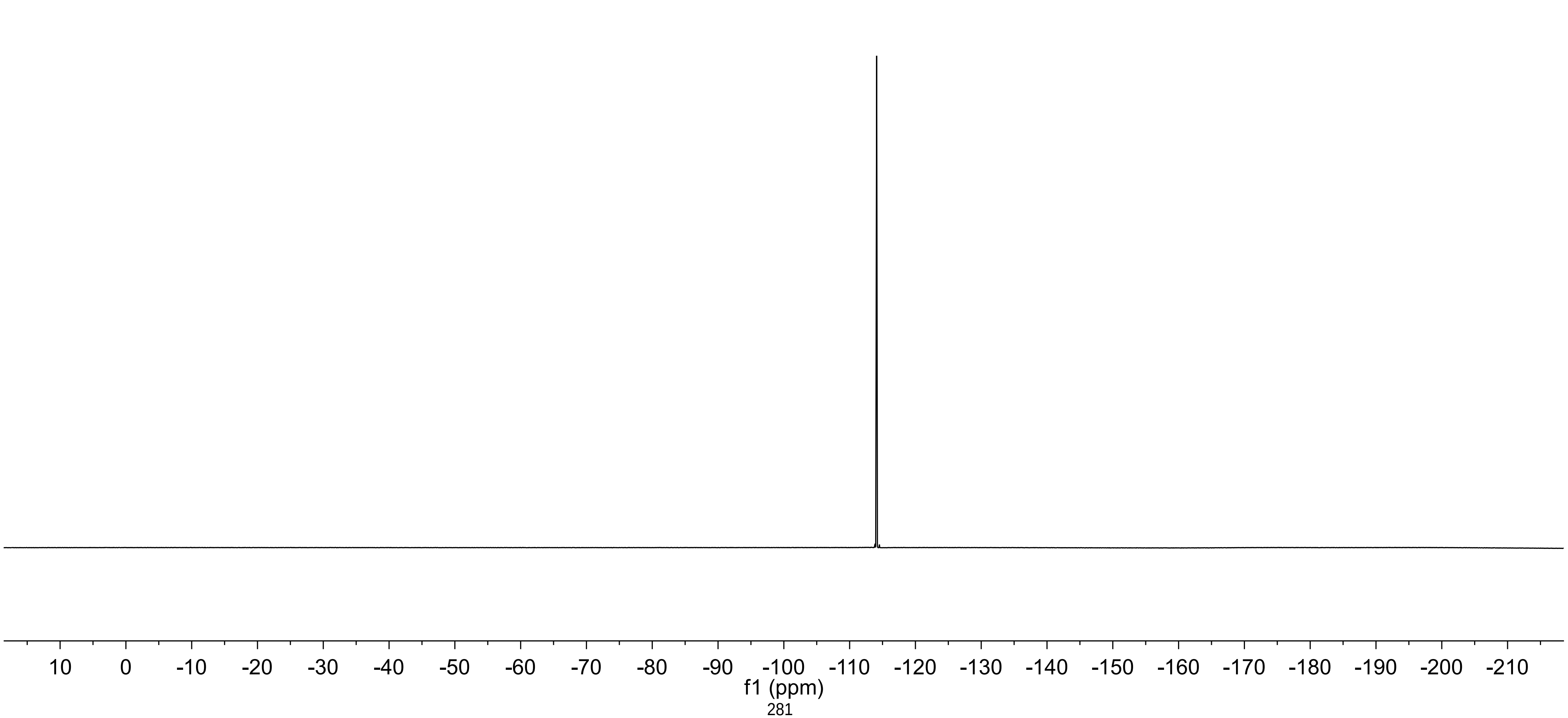

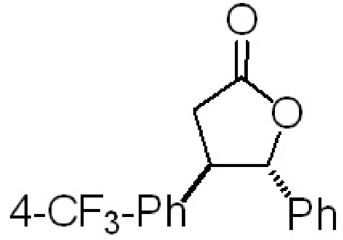

30b

^{1}H NMR spectrum (600 MHz, $CDCl_3$)

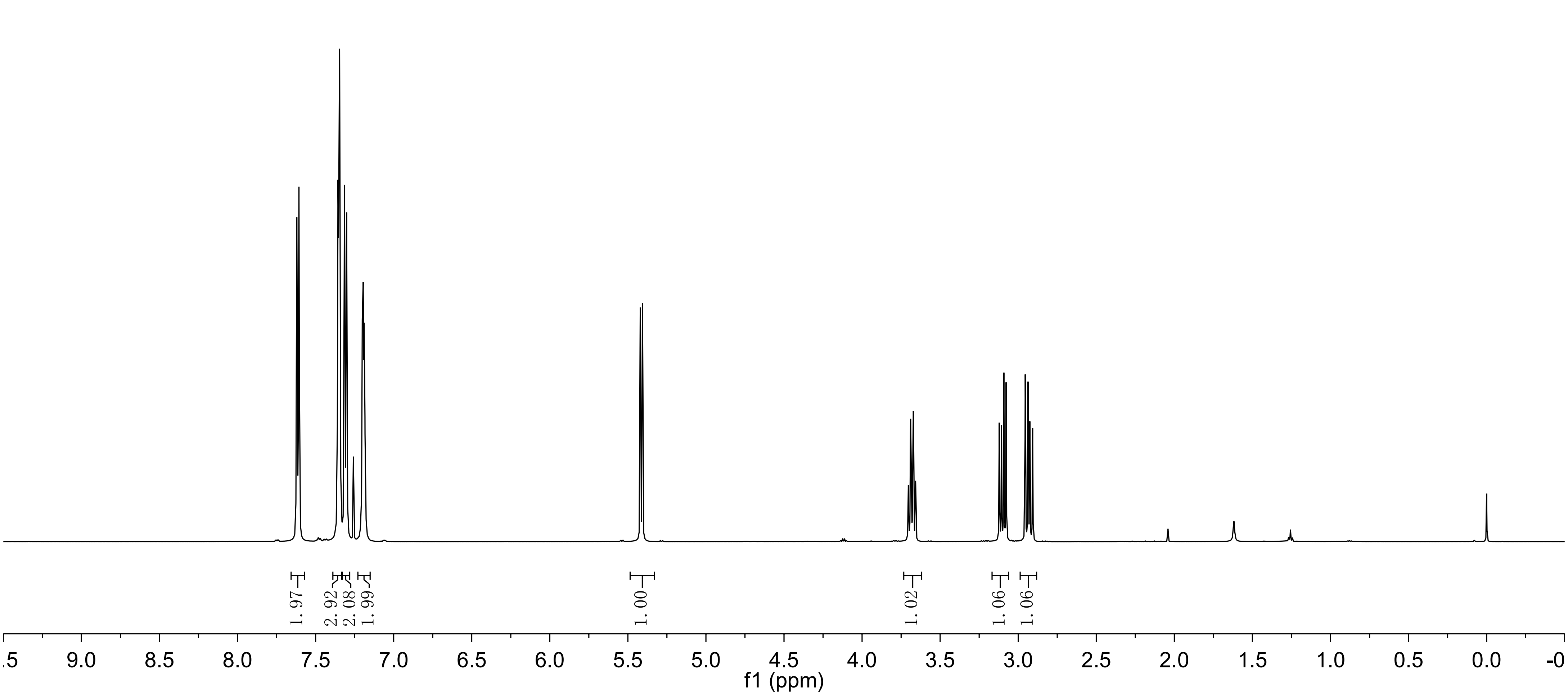

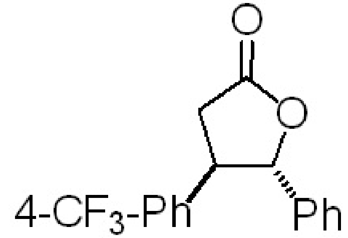

30b

13C NMR spectrum (150 MHz, $CDCl_3$)

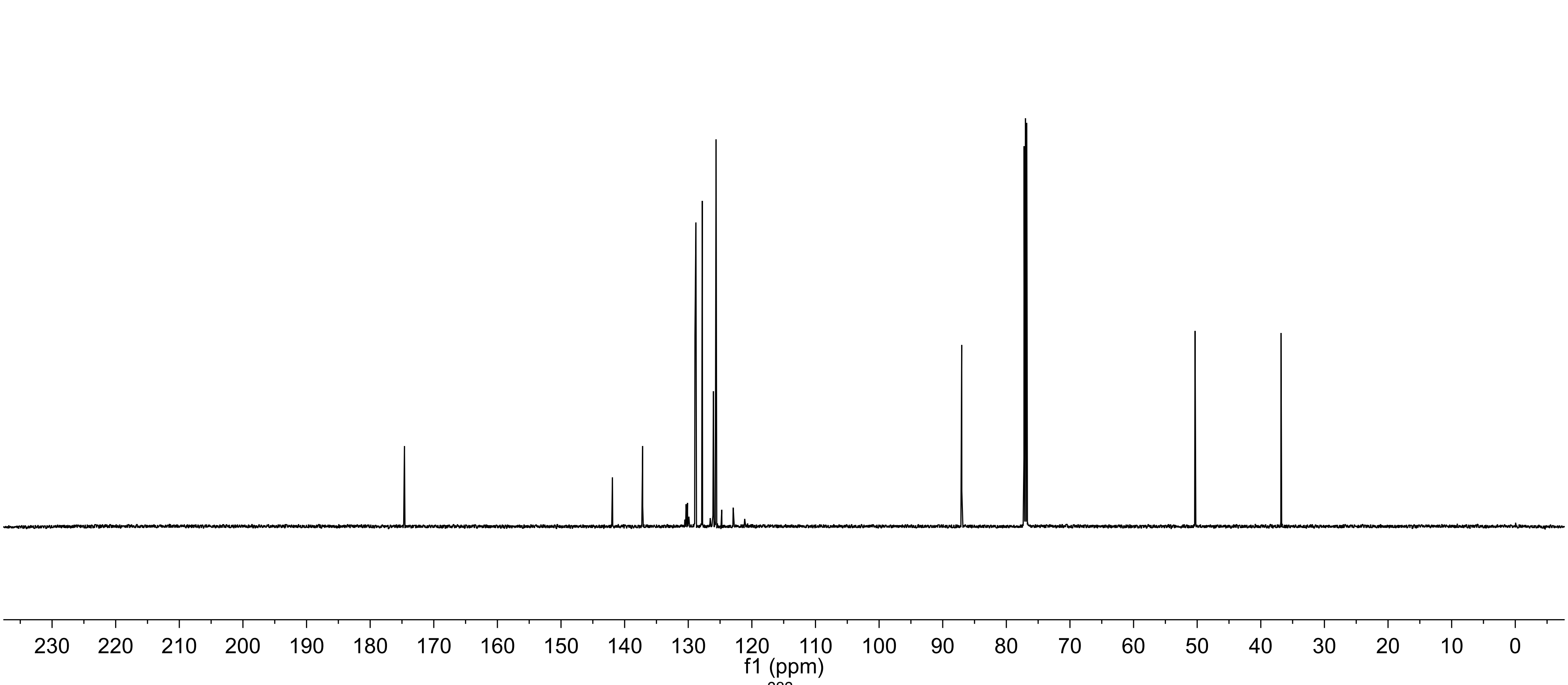

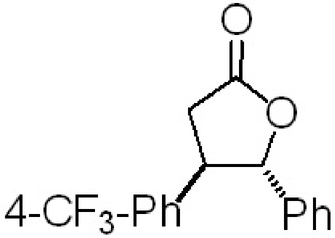

30b

^{19}F NMR spectrum (376 MHz, $CDCl_3$)

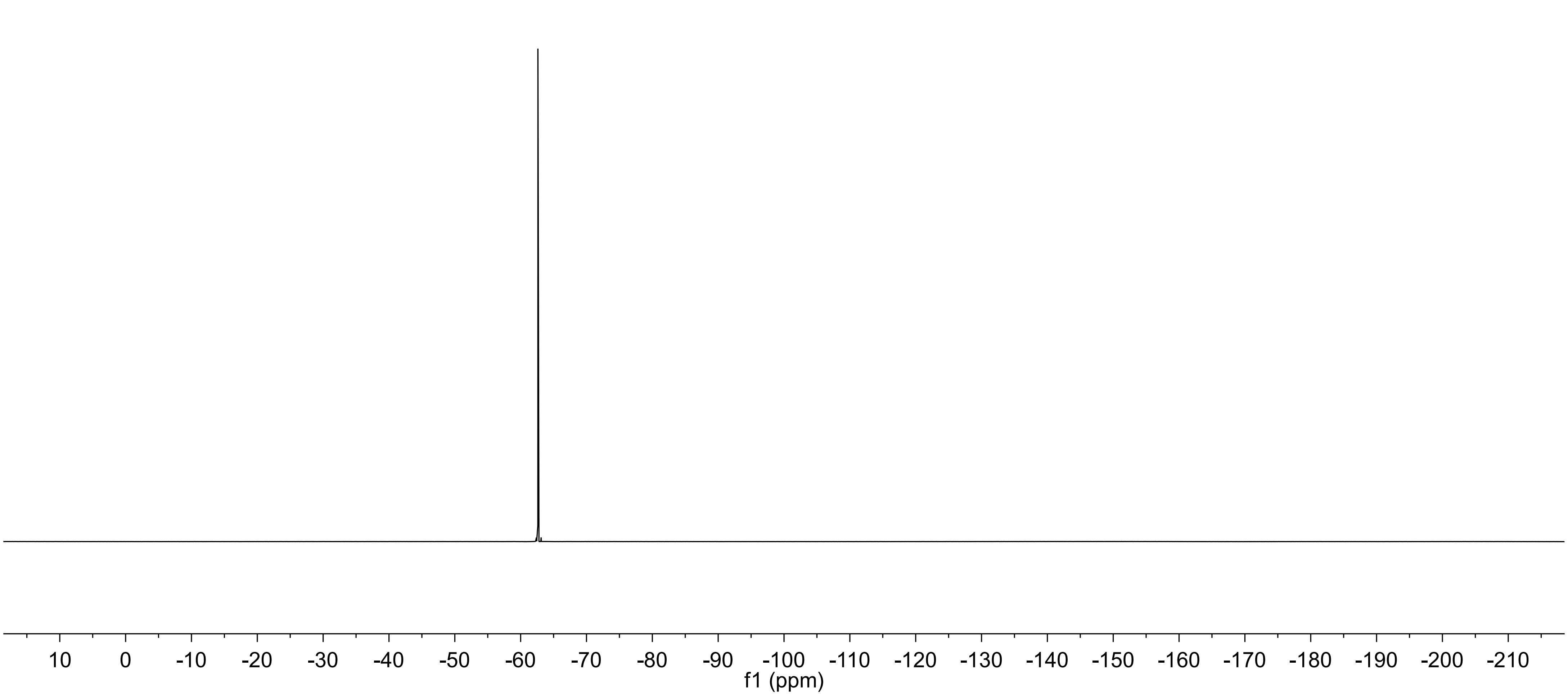

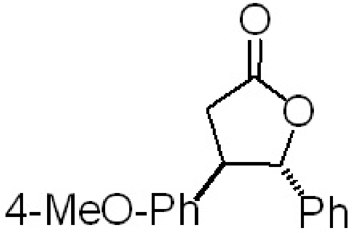

31b

^{1}H NMR spectrum (600 MHz, $CDCl_3$)

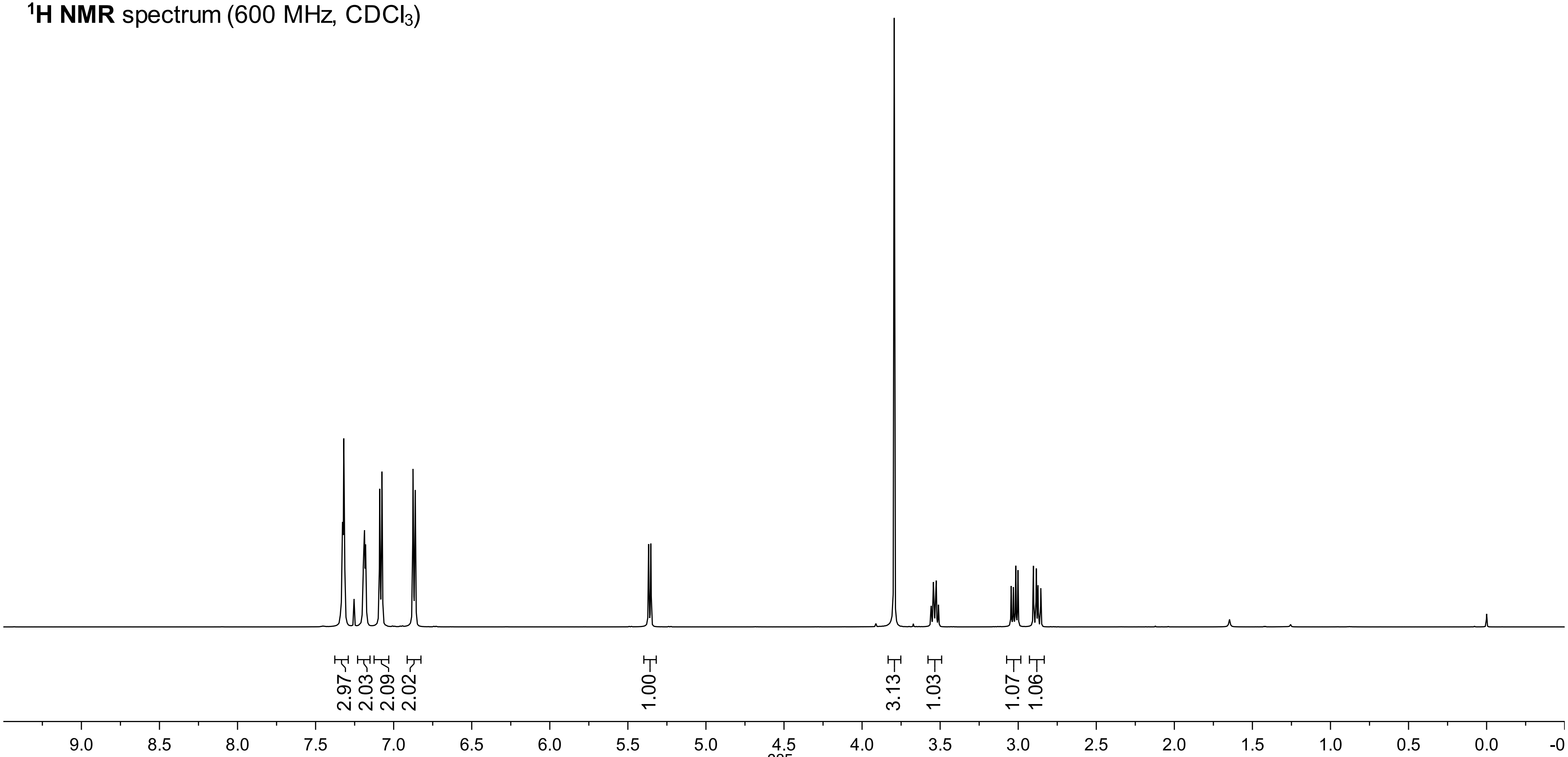

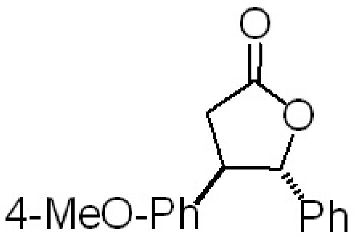

31b

^{13}C NMR spectrum (150 MHz, $CDCl_3$)

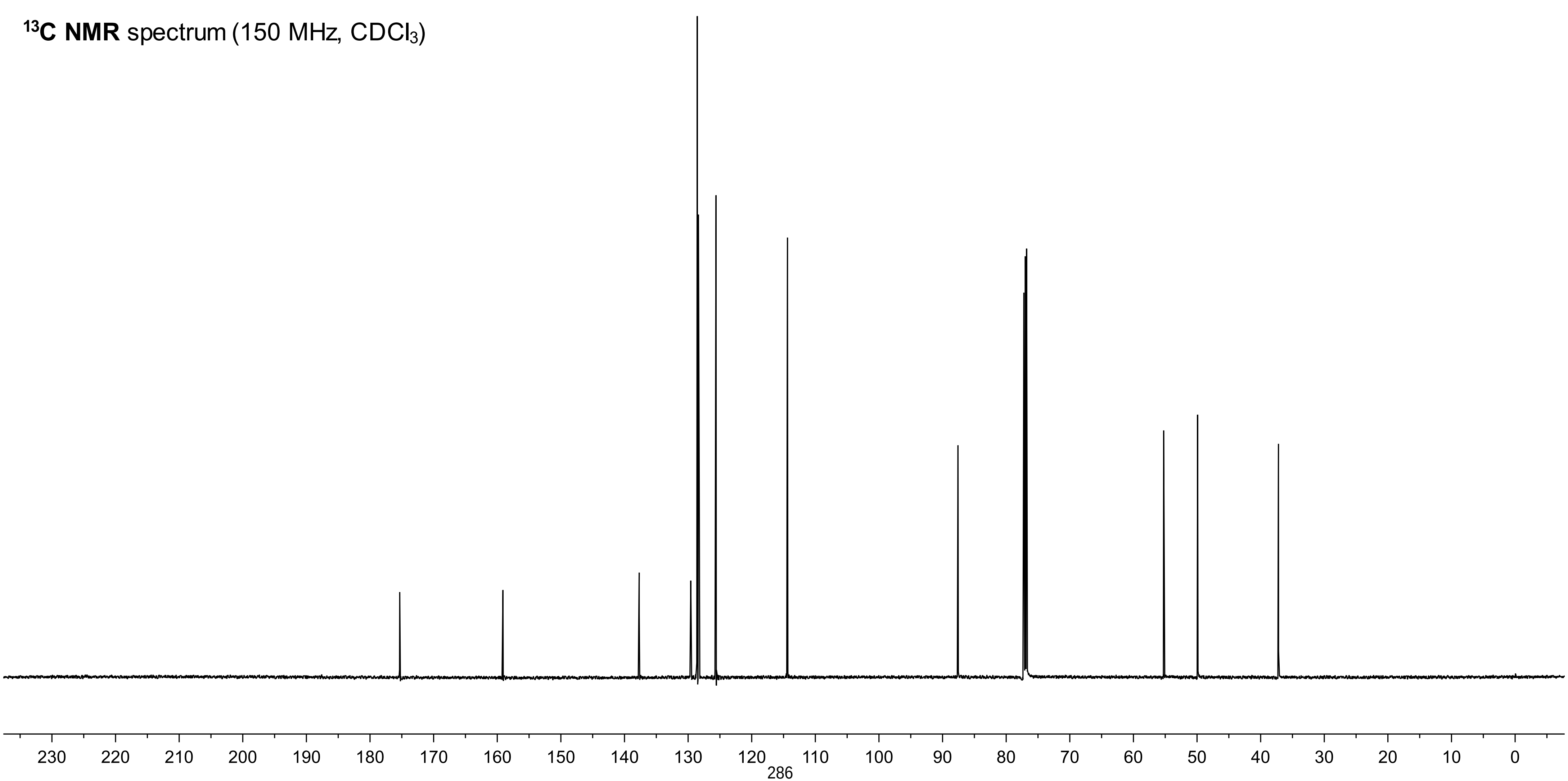

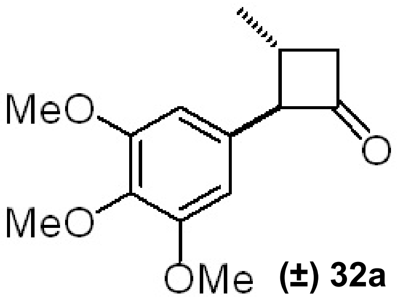

(±) 32a

^{1}H NMR spectrum (400 MHz, $CDCl_3$)

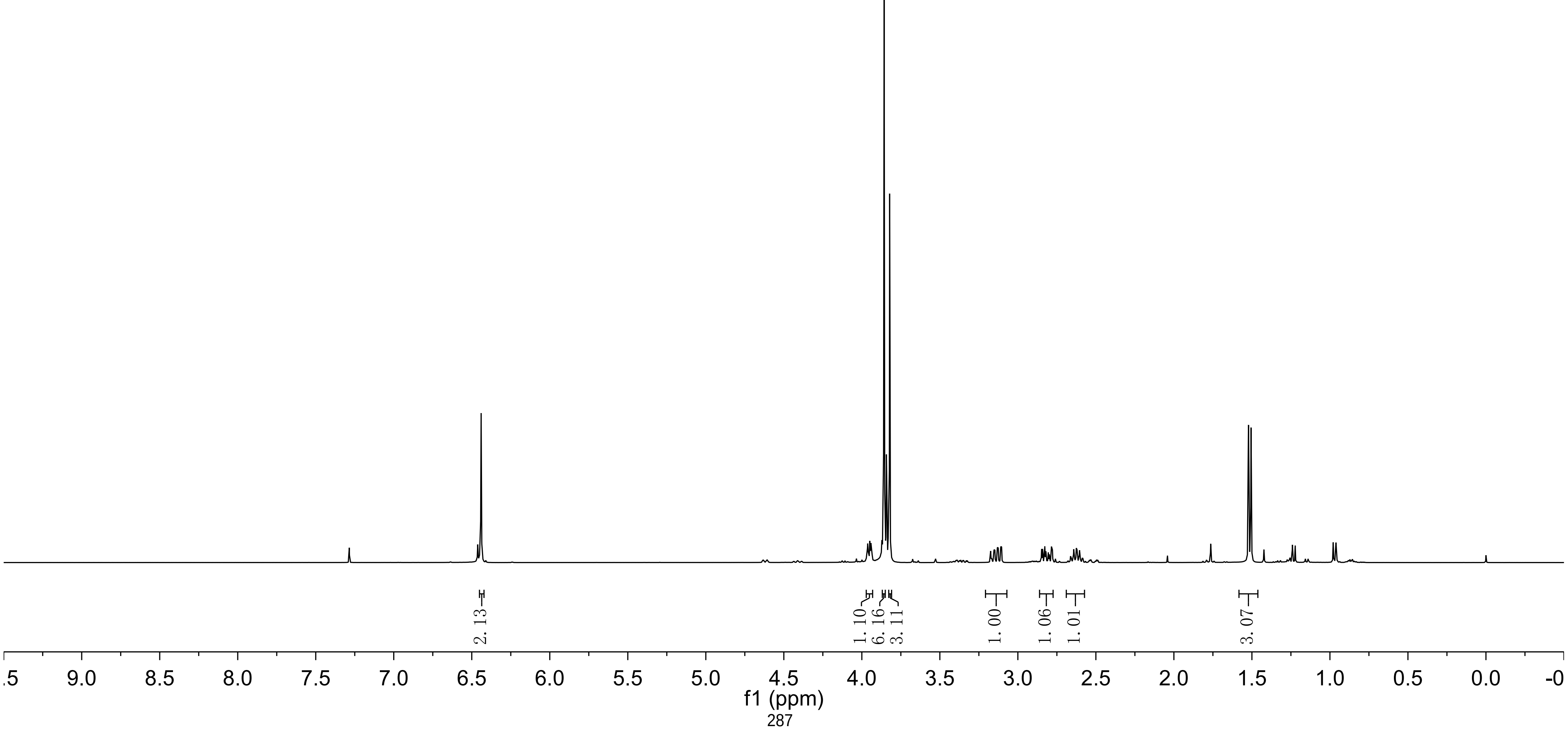

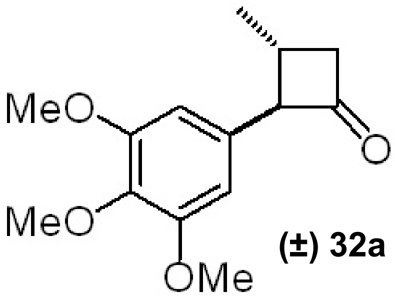

(±) 32a

^{13}C NMR spectrum (100 MHz, $CDCl_3$)

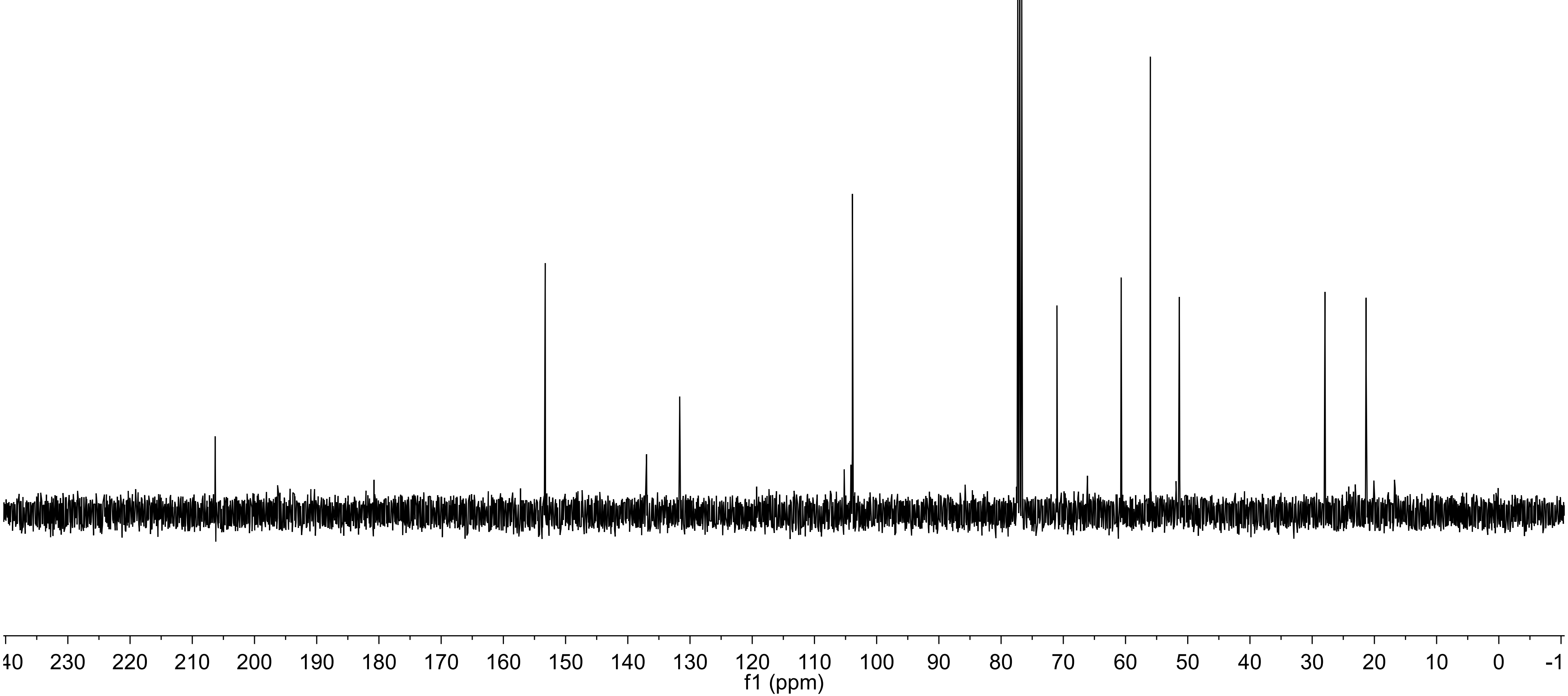

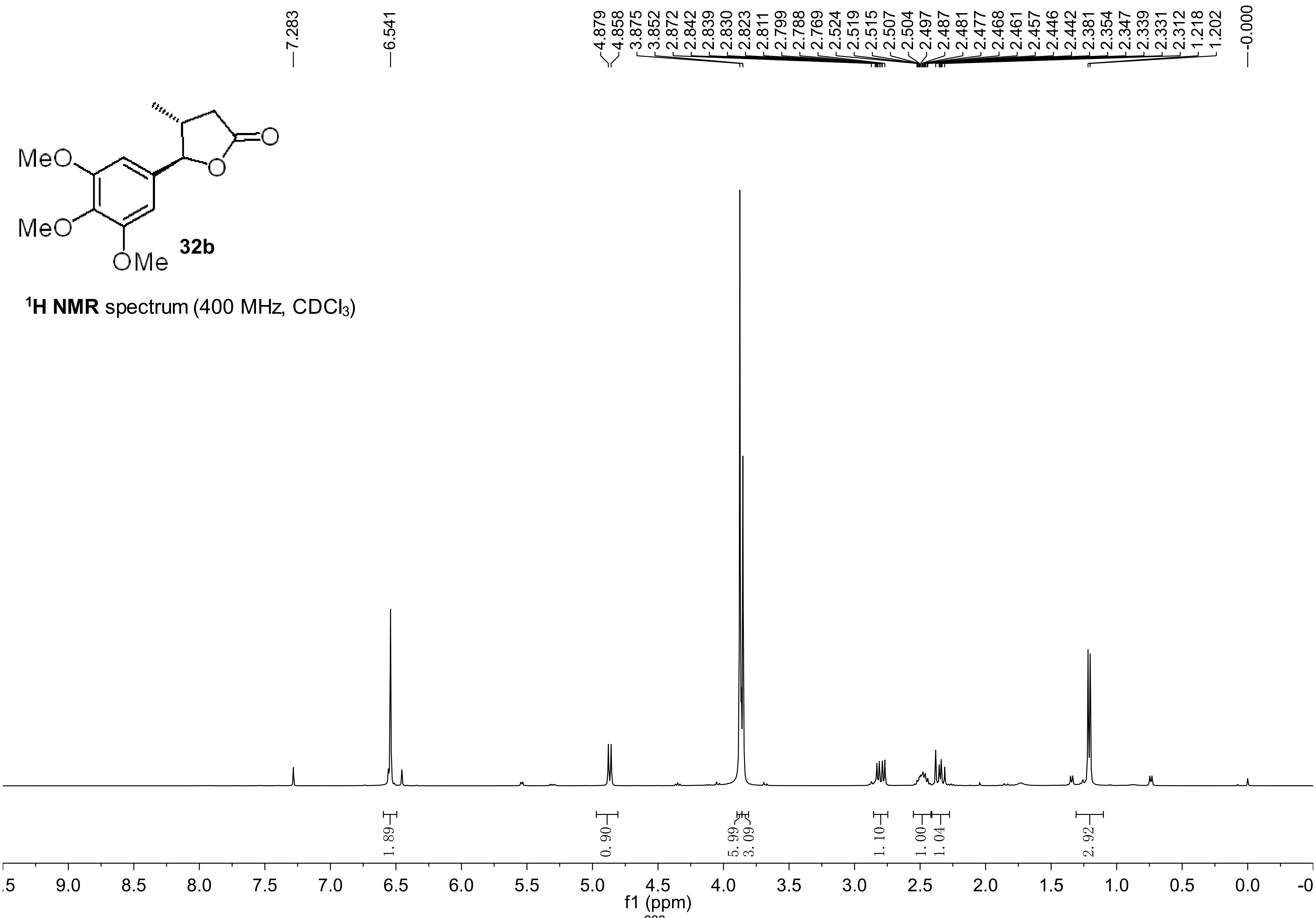
7.283
6.541
4.879
4.858
3.875
3.852
2.872
2.842
2.839
2.830
2.823
2.811
2.799
2.788
2.769
2.524
2.519
2.515
2.507
2.504
2.497
2.487
2.481
2.477
2.468
2.461
2.457
2.446
2.442
2.381
2.354
2.347
2.339
2.331
2.312
1.218
1.202
-0.000
MeO
MeO
OMe
O
O
32b
^{1}H NMR spectrum (400 MHz, $CDCl_3$)
1.89
0.90
5.99
3.09
1.10
1.00
1.04
2.92
.5
9.0
8.5
8.0
7.5
7.0
6.5
6.0
5.5
5.0
4.5
4.0
3.5
3.0
2.5
2.0
1.5
1.0
0.5
0.0
-0
f1 (ppm)

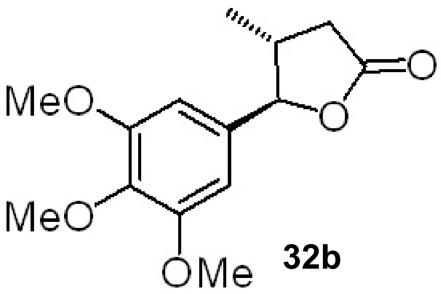

32b

^{13}C NMR spectrum (100 MHz, $CDCl_3$)

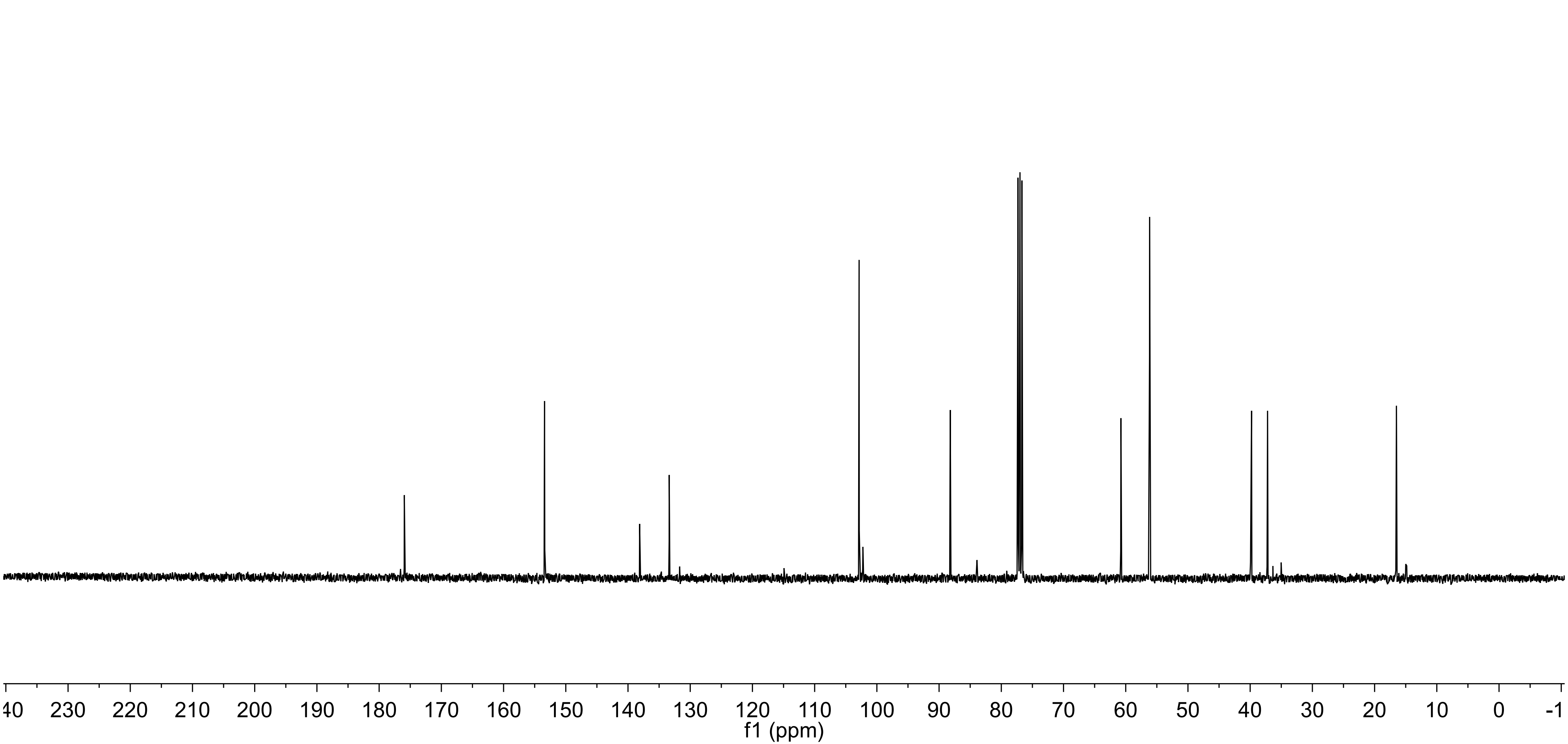

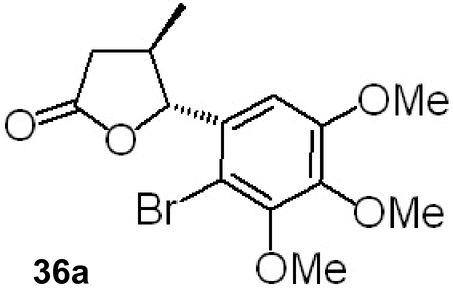

36a

^{1}H NMR spectrum (600 MHz, $CDCl_3$)

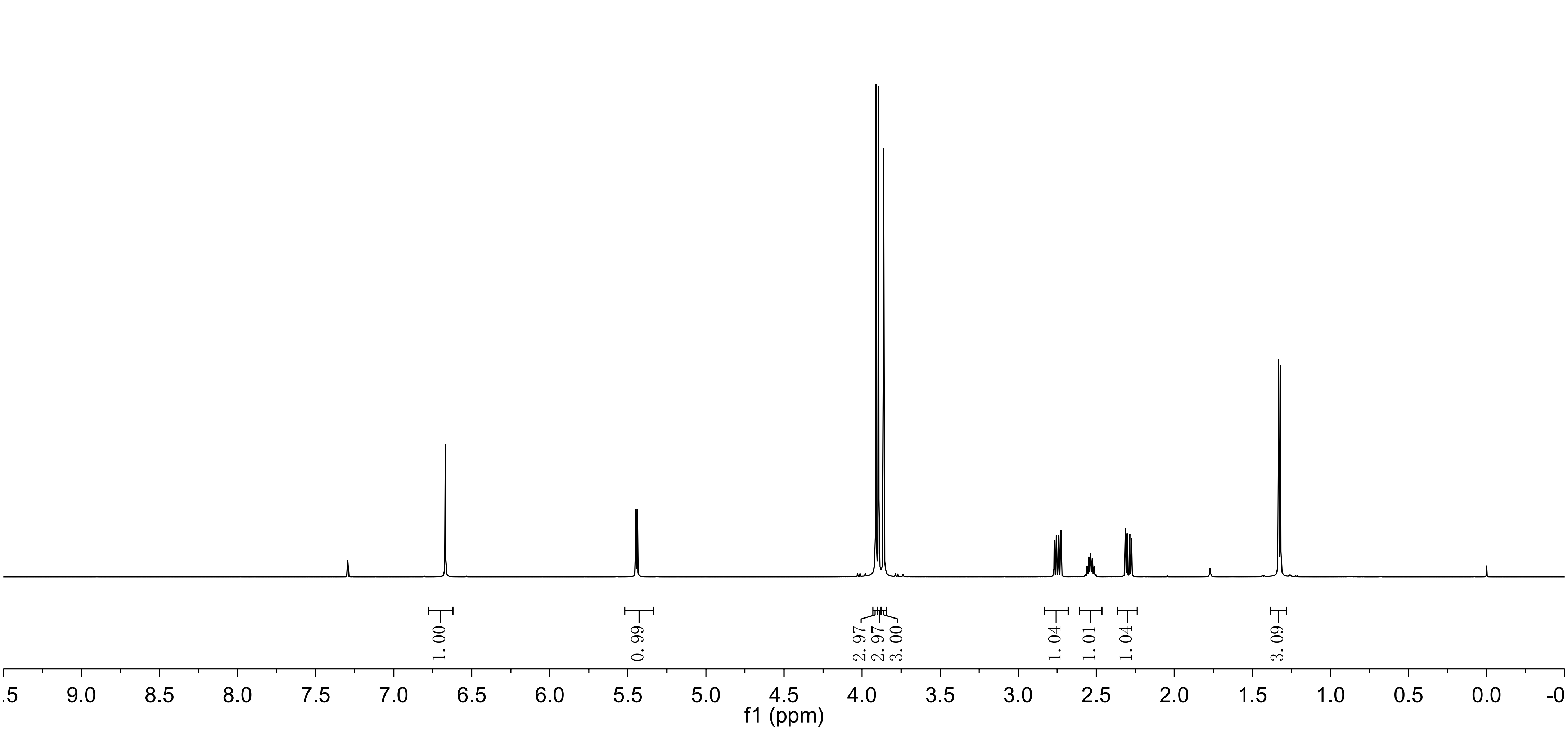

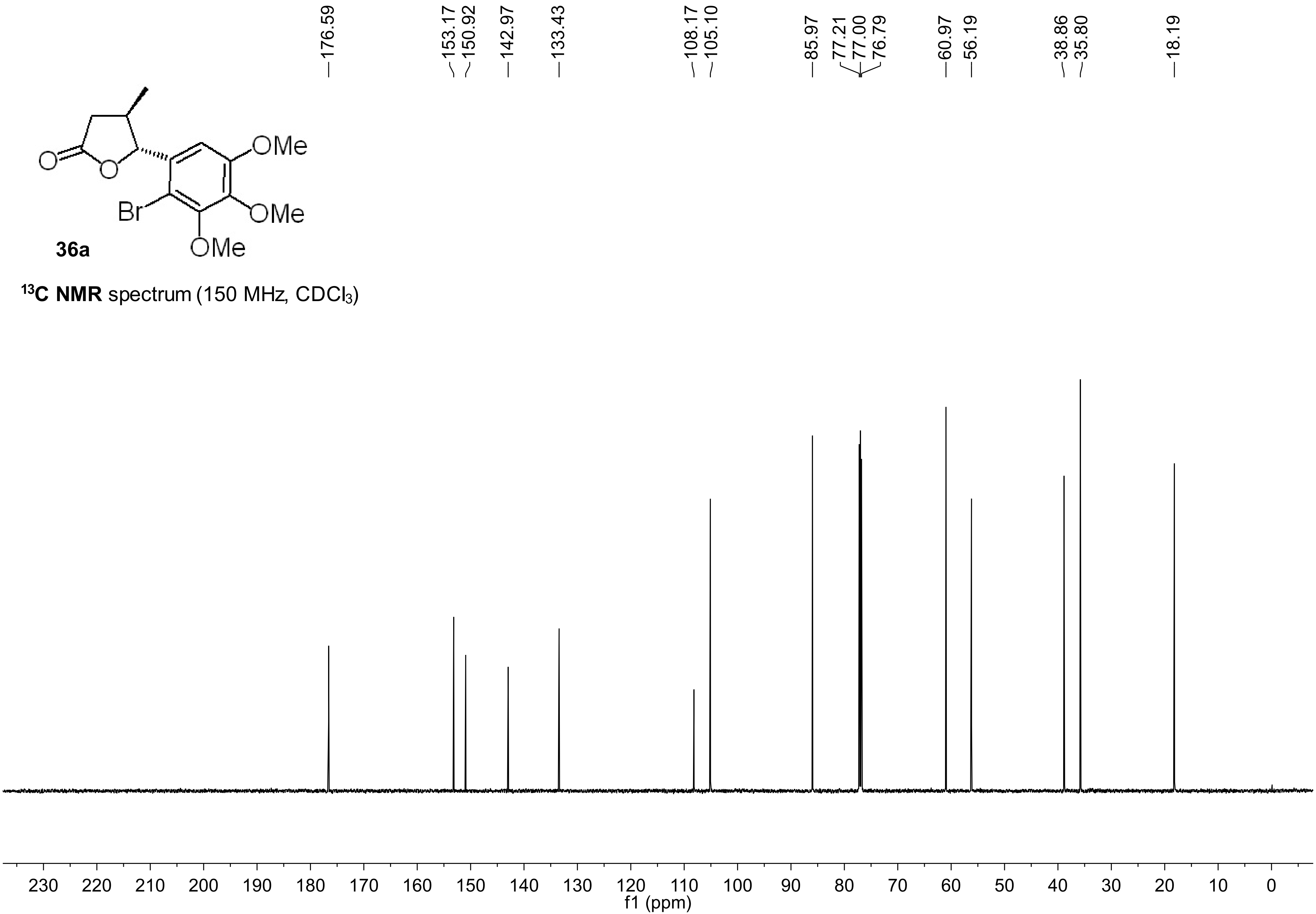

36a

^{13}C NMR spectrum (150 MHz, $CDCl_3$)

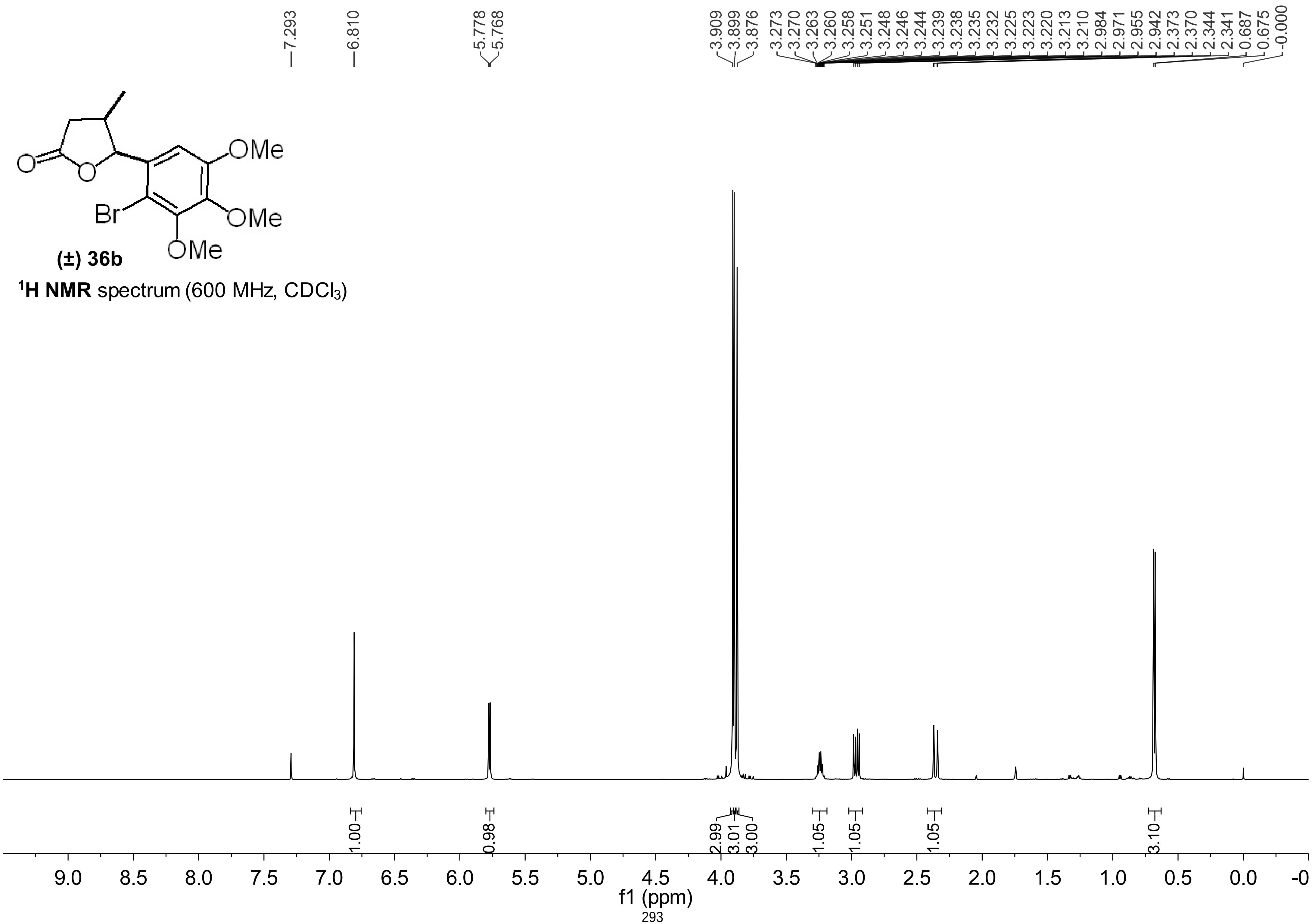
7.293
6.810
5.778
5.768
3.909
3.899
3.876
3.273
3.270
3.263
3.260
3.258
3.251
3.248
3.246
3.244
3.239
3.238
3.235
3.232
3.225
3.223
3.220
3.213
3.210
2.984
2.971
2.955
2.942
2.373
2.370
2.344
2.341
0.687
0.675
-0.000
OMe
OMe
OMe
Br
(±) 36b
1H NMR spectrum (600 MHz, CDCl3)
1.00
0.98
2.99
3.01
3.00
1.05
1.05
1.05
3.10
9.0
8.5
8.0
7.5
7.0
6.5
6.0
5.5
5.0
4.5
4.0
3.5
3.0
2.5
2.0
1.5
1.0
0.5
0.0
-0
f1 (ppm)

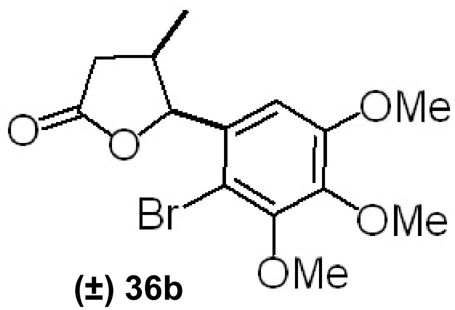

(±) 36b

^{13}C NMR spectrum (150 MHz, $CDCl_3$)

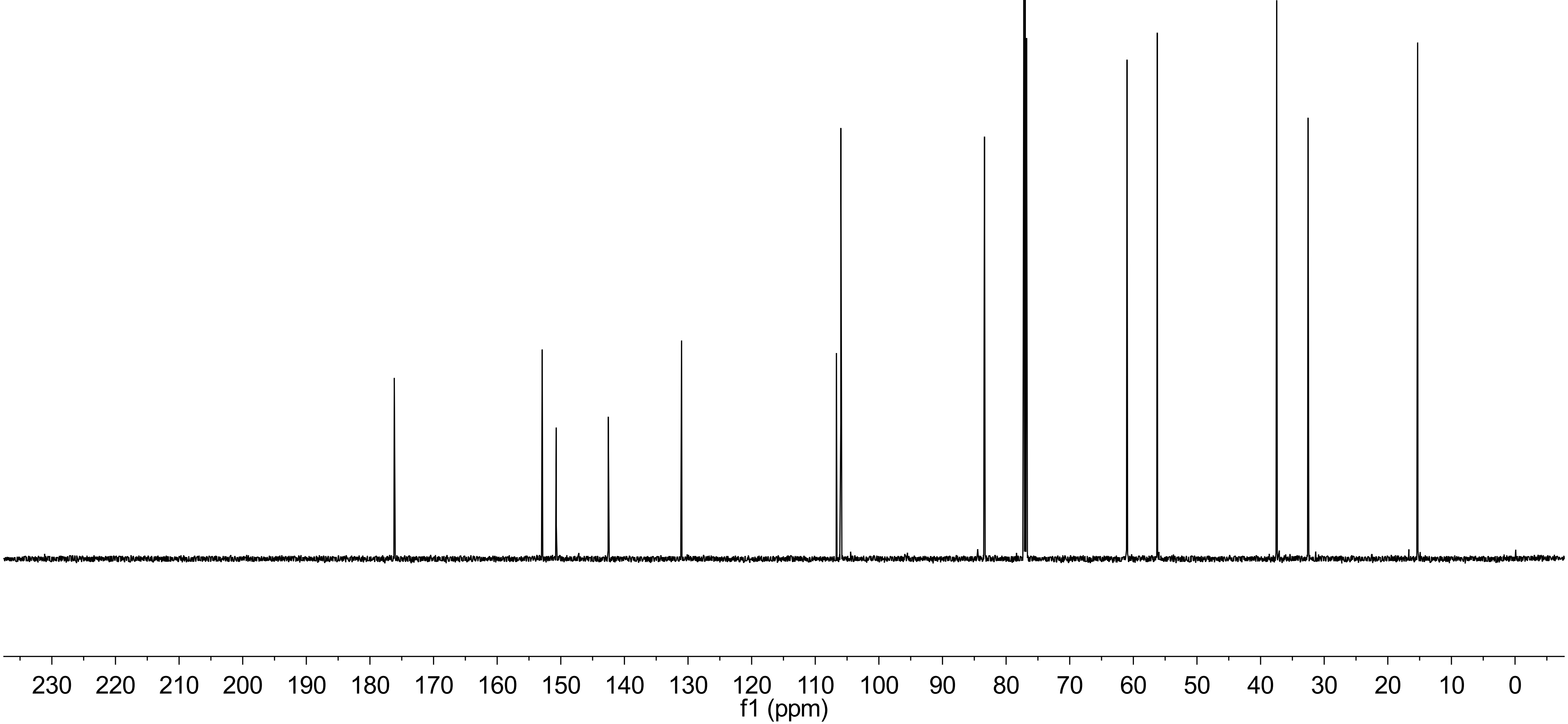

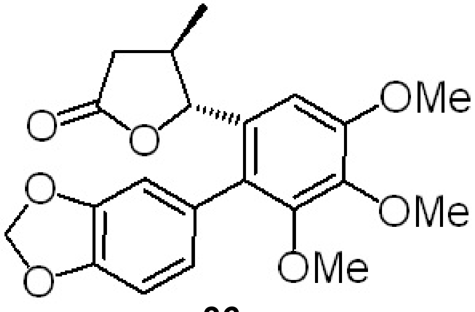

36

^{1}H NMR spectrum (400 MHz, $CDCl_3$)

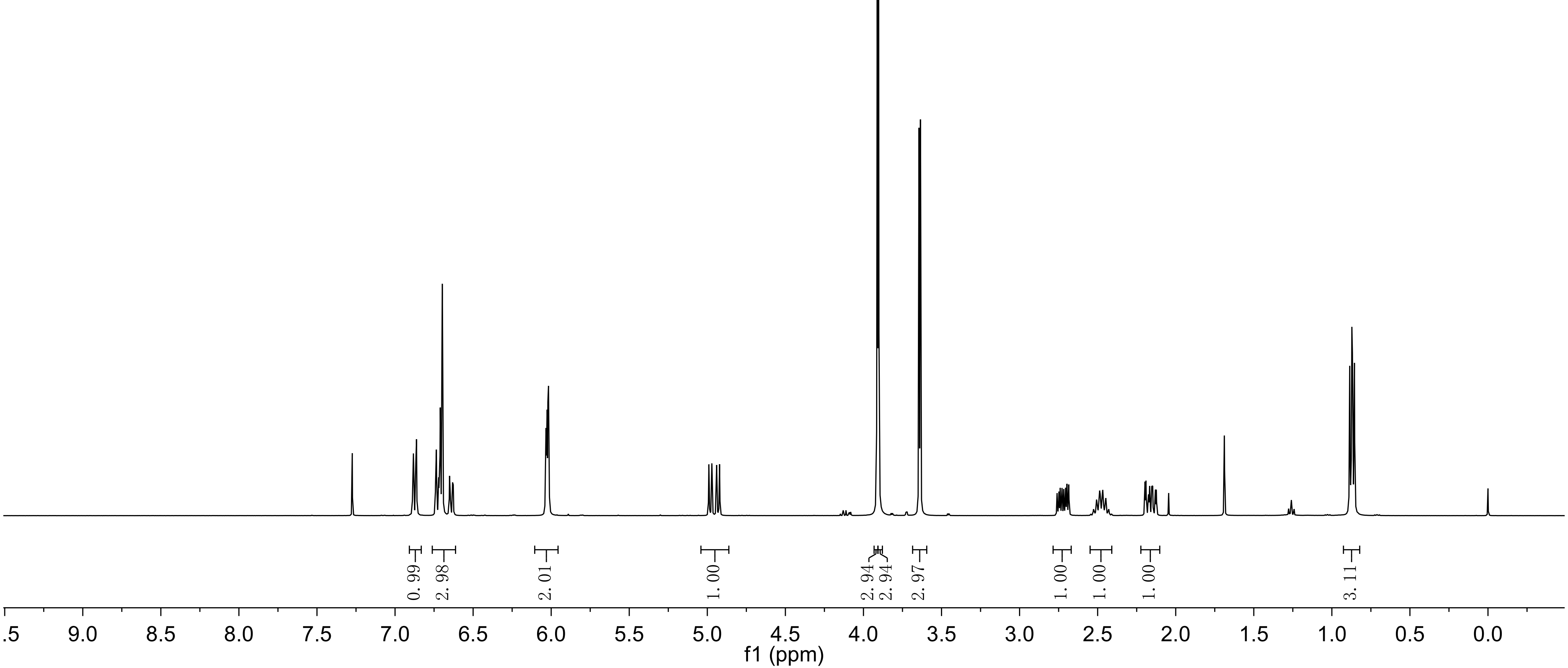

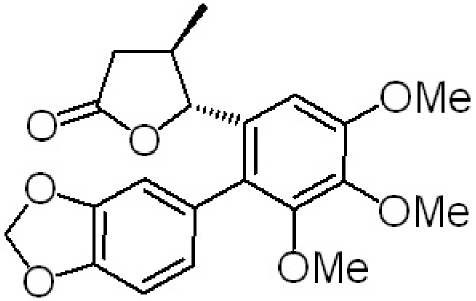

36

^{13}C NMR spectrum (100 MHz, $CDCl_3$)

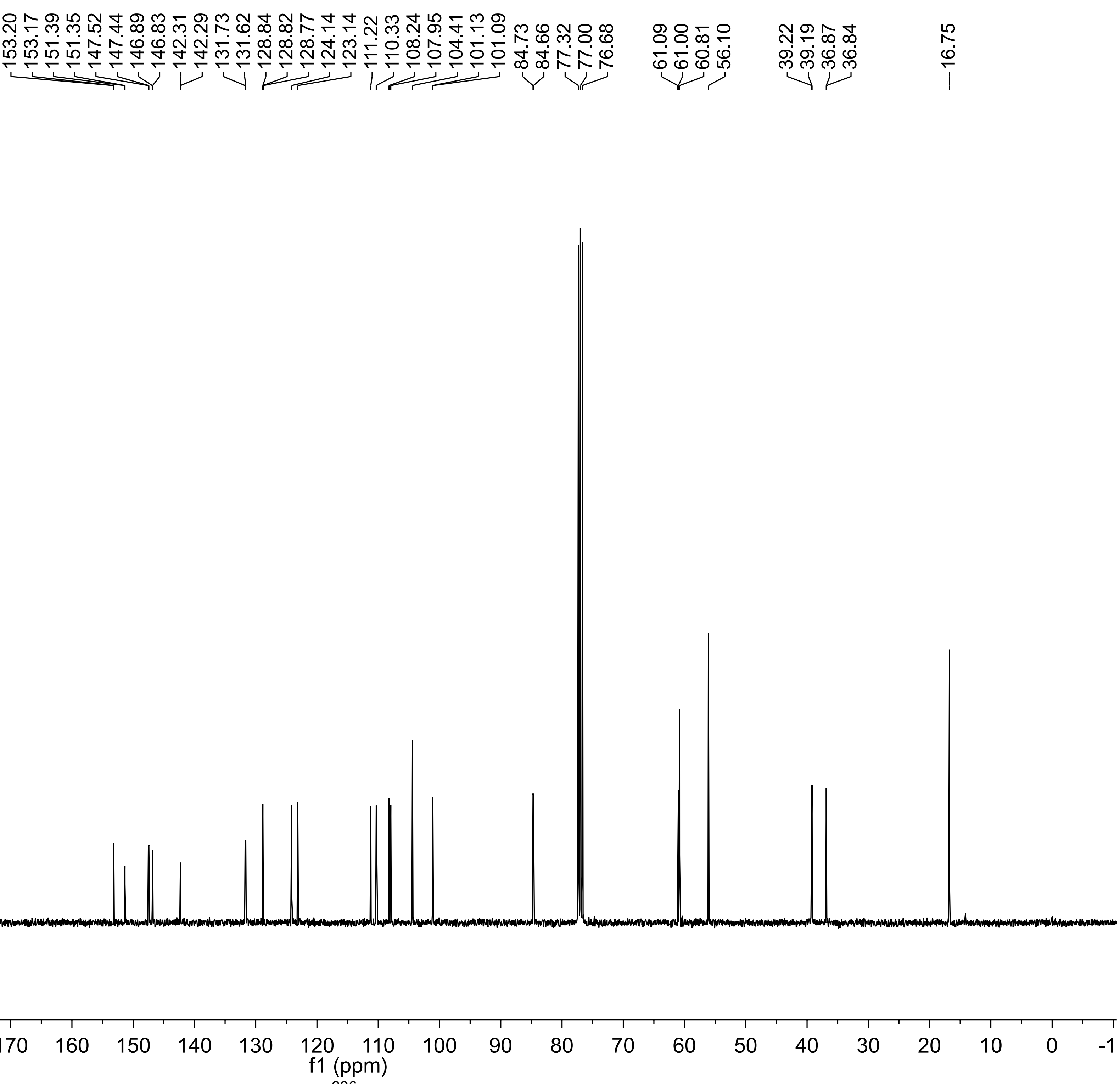

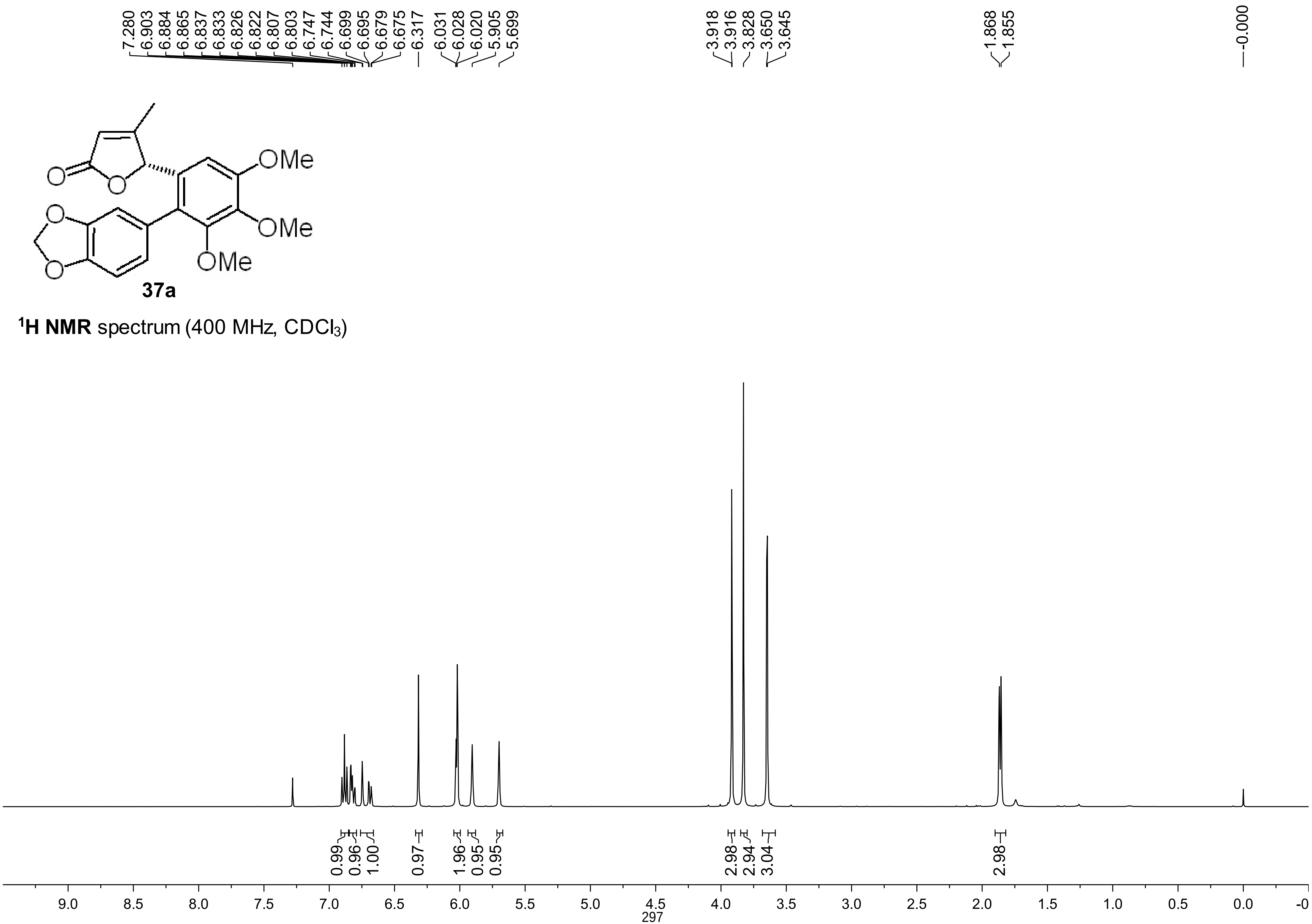

37a

¹H NMR spectrum (400 MHz, $CDCl_3$)

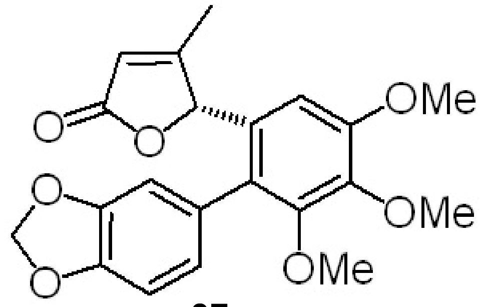

37a

^{13}C NMR spectrum (100 MHz, $CDCl_3$)

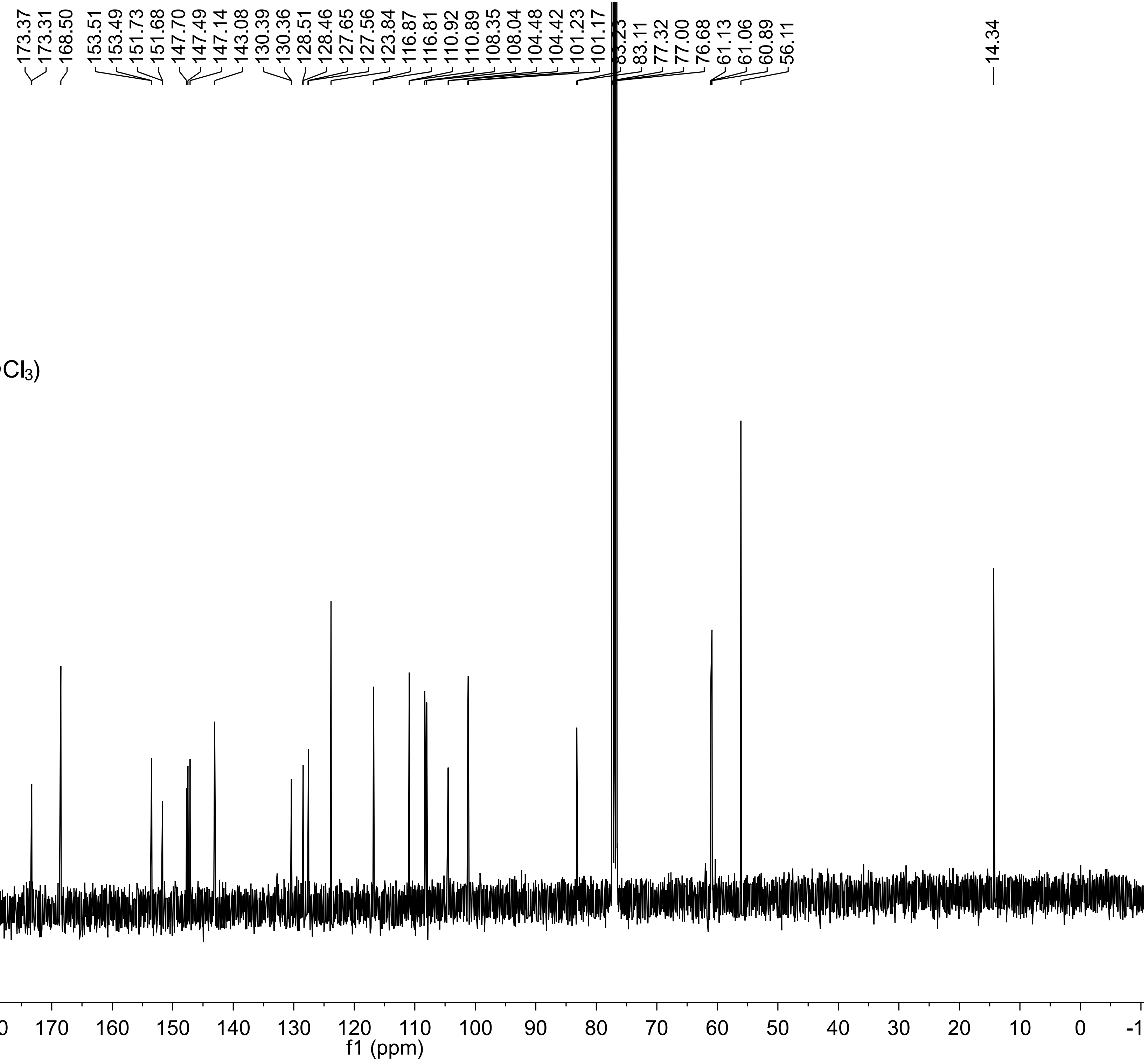

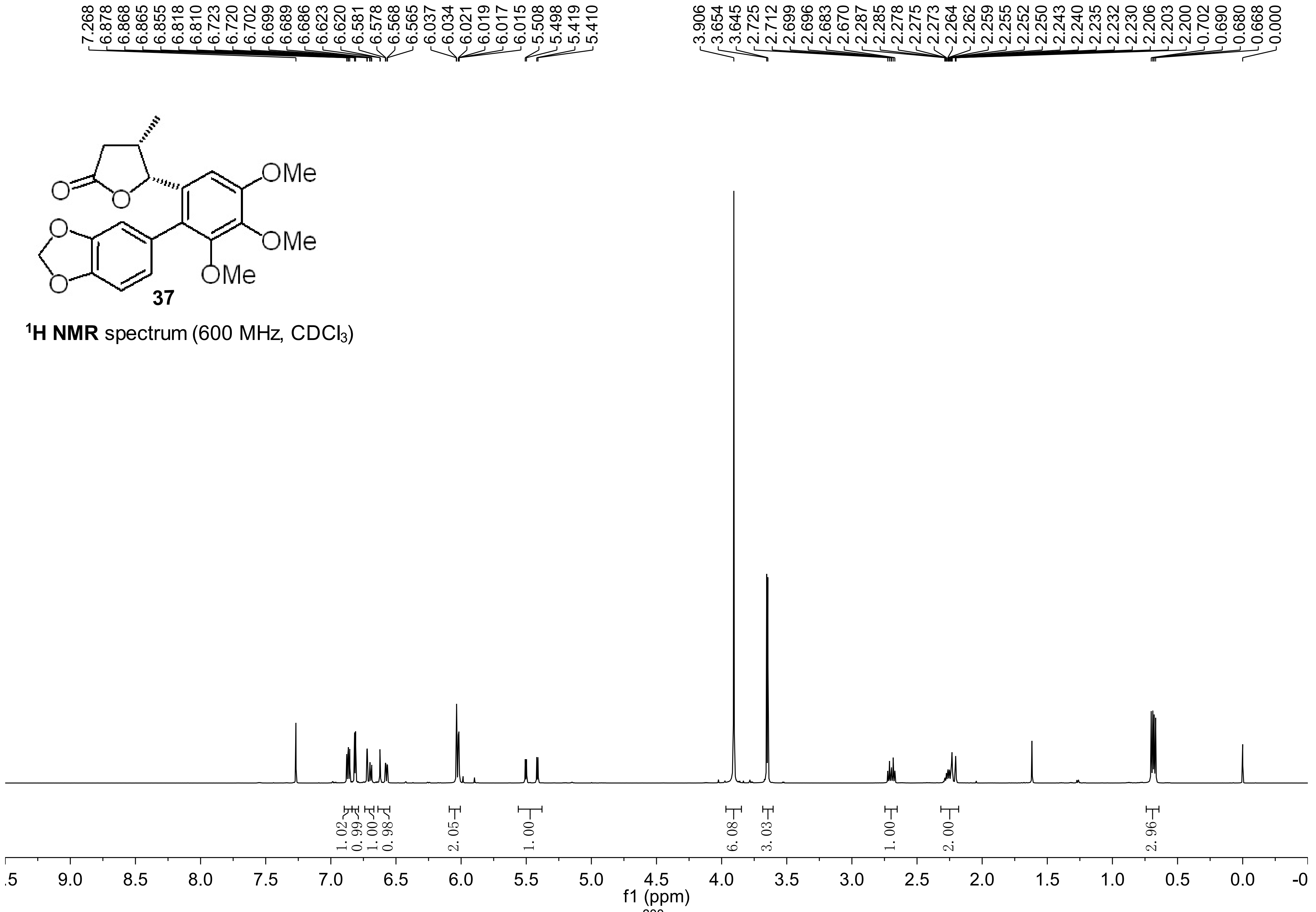
7.268 6.878 6.868 6.865 6.855 6.818 6.810 6.723 6.720 6.702 6.699 6.689 6.686 6.623 6.620 6.581 6.578 6.568 6.565 6.037 6.034 6.021 6.019 6.017 6.015 5.508 5.498 5.419 5.410
3.906 3.654 3.645 2.725 2.712 2.699 2.696 2.683 2.670 2.287 2.285 2.278 2.275 2.273 2.264 2.262 2.259 2.255 2.252 2.250 2.243 2.240 2.235 2.232 2.230 2.206 2.203 2.200 0.702 0.690 0.680 0.668 0.000
OMe
OMe
OMe
O
O
O
O
37
1H NMR spectrum (600 MHz, CDCl3)
1.02 0.99 1.00 0.98 2.05 1.00 6.08 3.03 1.00 2.00 2.96
.5 9.0 8.5 8.0 7.5 7.0 6.5 6.0 5.5 5.0 4.5 4.0 3.5 3.0 2.5 2.0 1.5 1.0 0.5 0.0 -0
f1 (ppm)

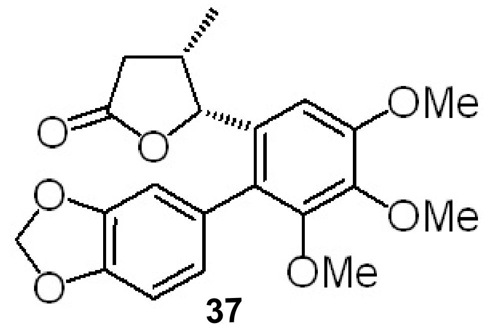

37

^{13}C NMR spectrum (150 MHz, $CDCl_3$)

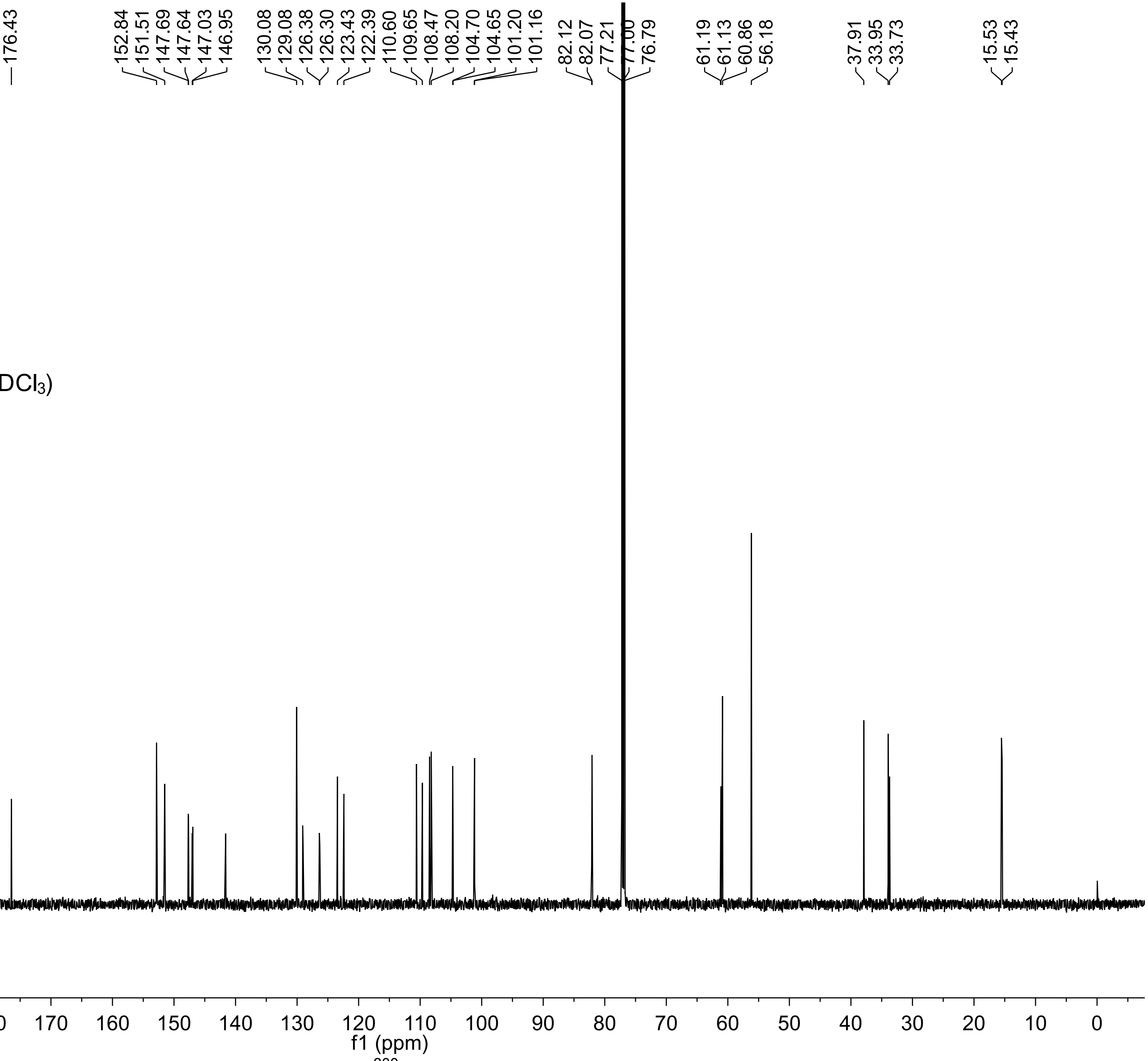

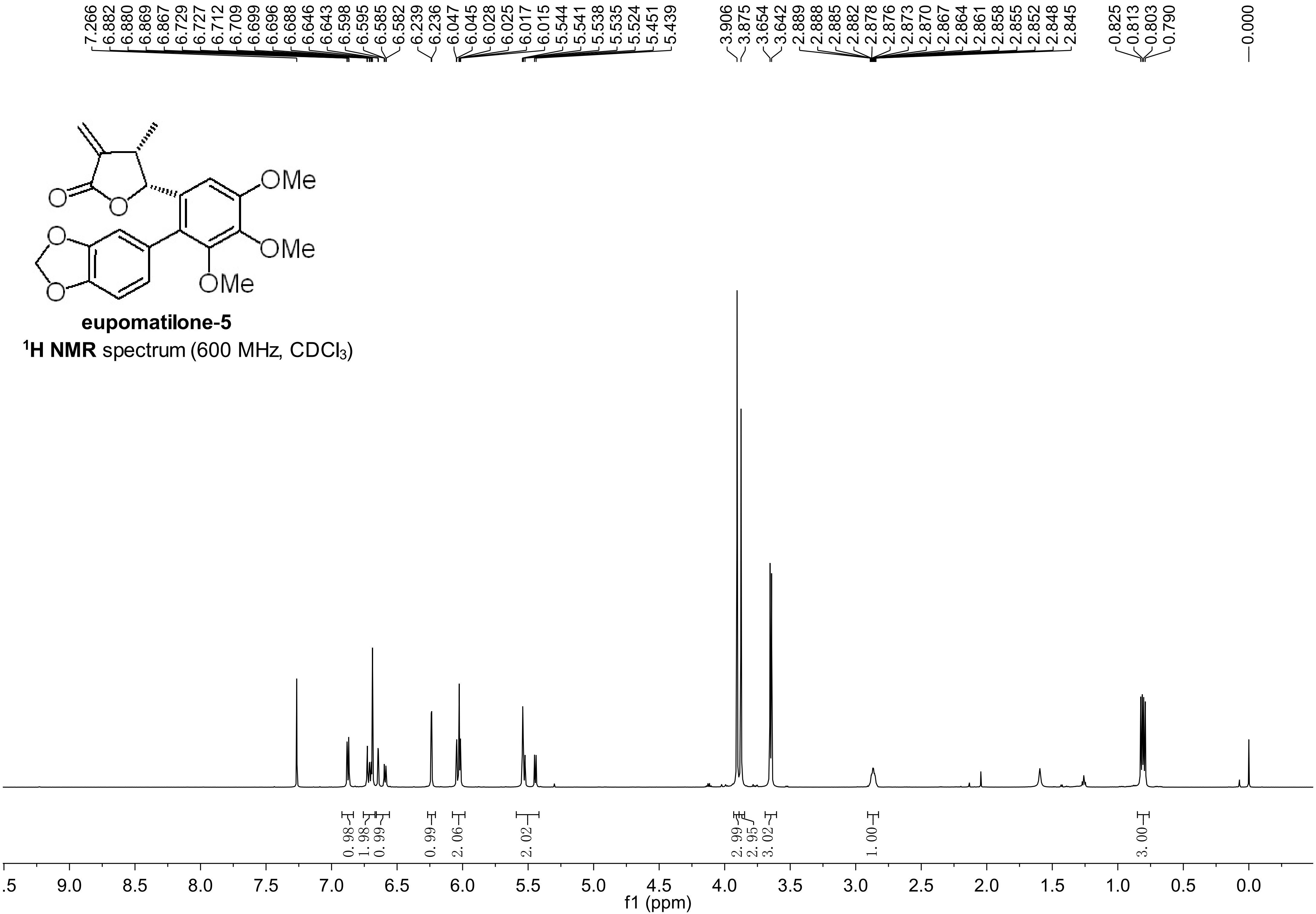

eupomatilone-5

^{1}H NMR spectrum (600 MHz, $CDCl_3$)

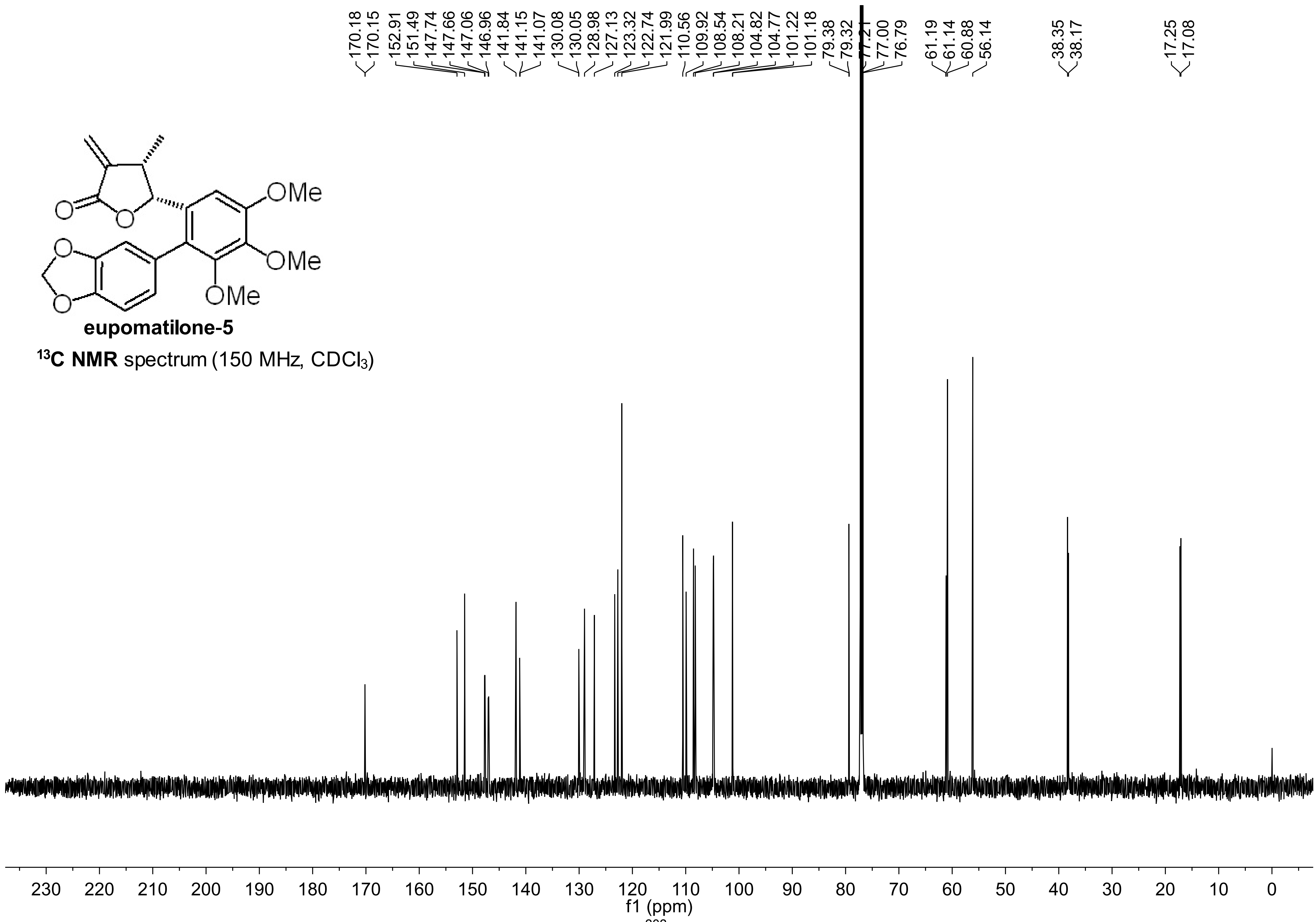
170.18
170.15
152.91
151.49
147.74
147.66
147.06
146.96
141.84
141.15
141.07
130.08
130.05
128.98
127.13
123.32
122.74
121.99
110.56
109.92
108.54
108.21
104.82
104.77
101.22
101.18
79.38
79.32
77.21
77.00
76.79
61.19
61.14
60.88
56.14
38.35
38.17
17.25
17.08
OMe
OMe
OMe
O
O
O
O
eupomatilone-5
^{13}C NMR spectrum (150 MHz, $CDCl_3$)
230 220 210 200 190 180 170 160 150 140 130 120 110 100 90 80 70 60 50 40 30 20 10 0
f1 (ppm)

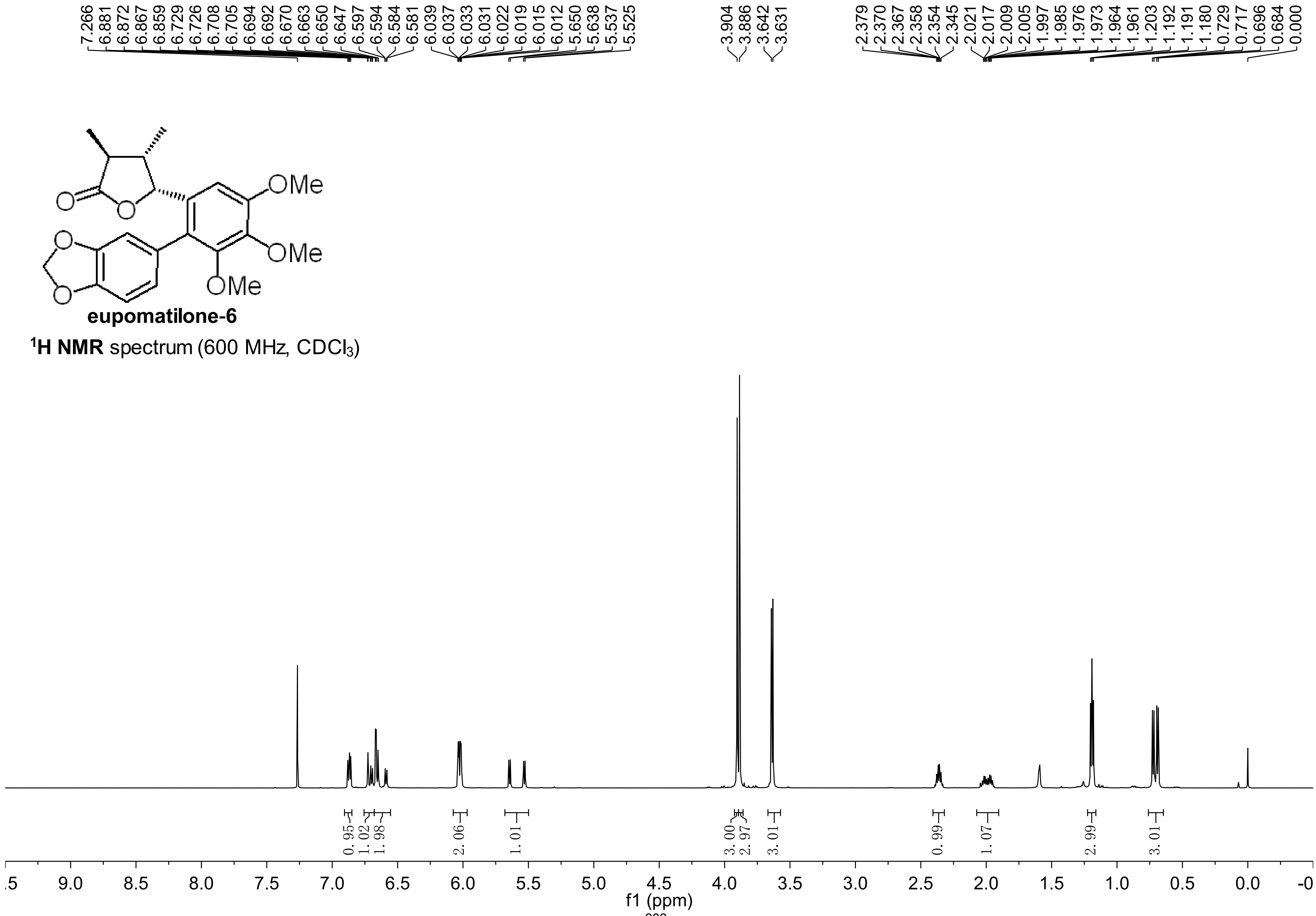
7.266 6.881 6.872 6.867 6.859 6.729 6.726 6.708 6.705 6.694 6.692 6.670 6.663 6.650 6.647 6.597 6.594 6.584 6.581 6.039 6.037 6.033 6.031 6.022 6.019 6.015 6.012 5.650 5.638 5.537 5.525
3.904 3.886 3.642 3.631
2.379 2.370 2.367 2.358 2.354 2.345 2.021 2.017 2.009 2.005 1.997 1.985 1.976 1.973 1.964 1.961 1.203 1.192 1.191 1.180 0.729 0.717 0.696 0.684 0.000
OMe
OMe
OMe
eupomatilone-6
1H NMR spectrum (600 MHz, CDCl3)
0.95 1.02 1.98 2.06 1.01 3.00 2.97 3.01 0.99 1.07 2.99 3.01
.5 9.0 8.5 8.0 7.5 7.0 6.5 6.0 5.5 5.0 4.5 4.0 3.5 3.0 2.5 2.0 1.5 1.0 0.5 0.0 -0
f1 (ppm)

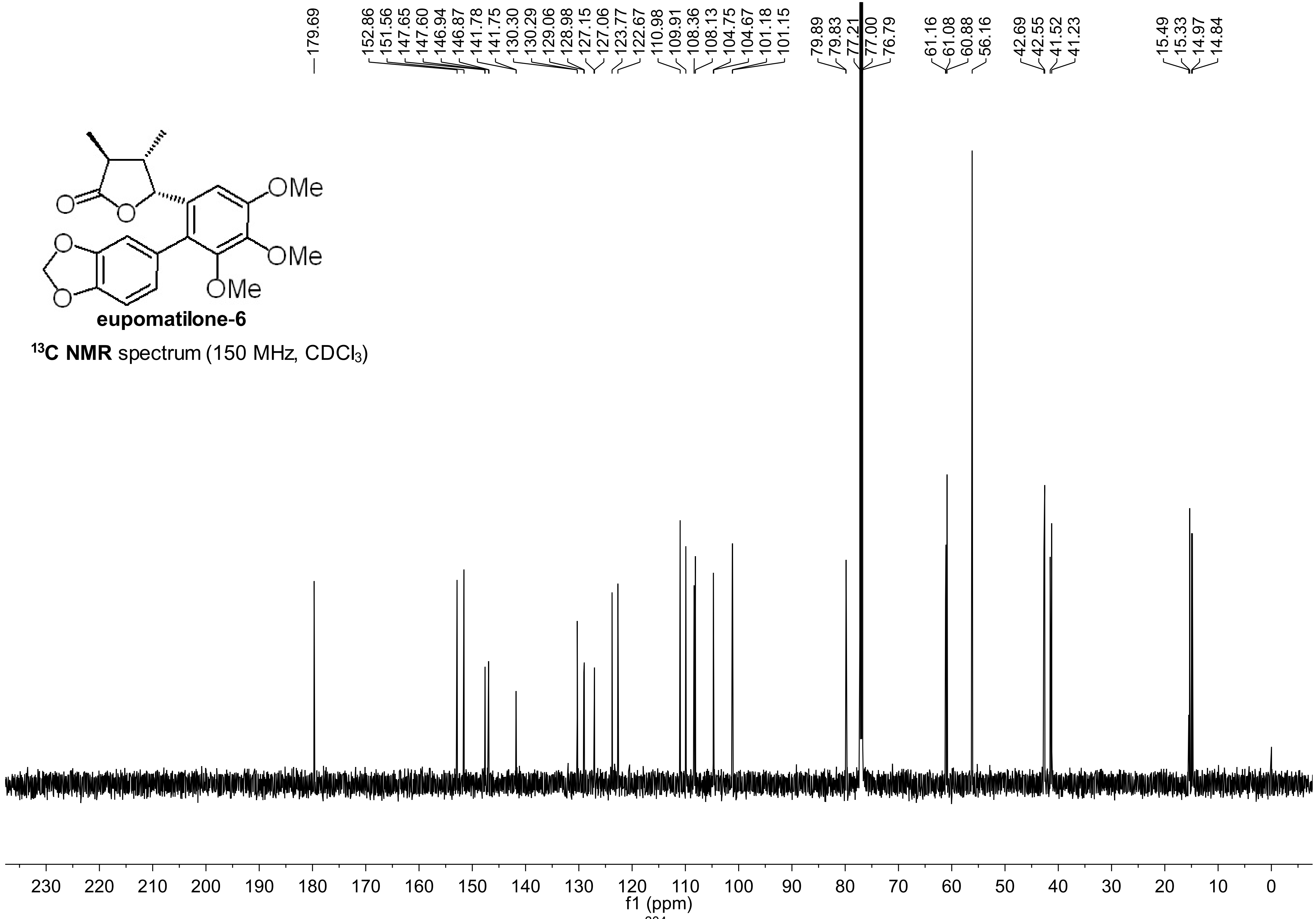

eupomatilone-6

^{13}C NMR spectrum (150 MHz, $CDCl_3$)

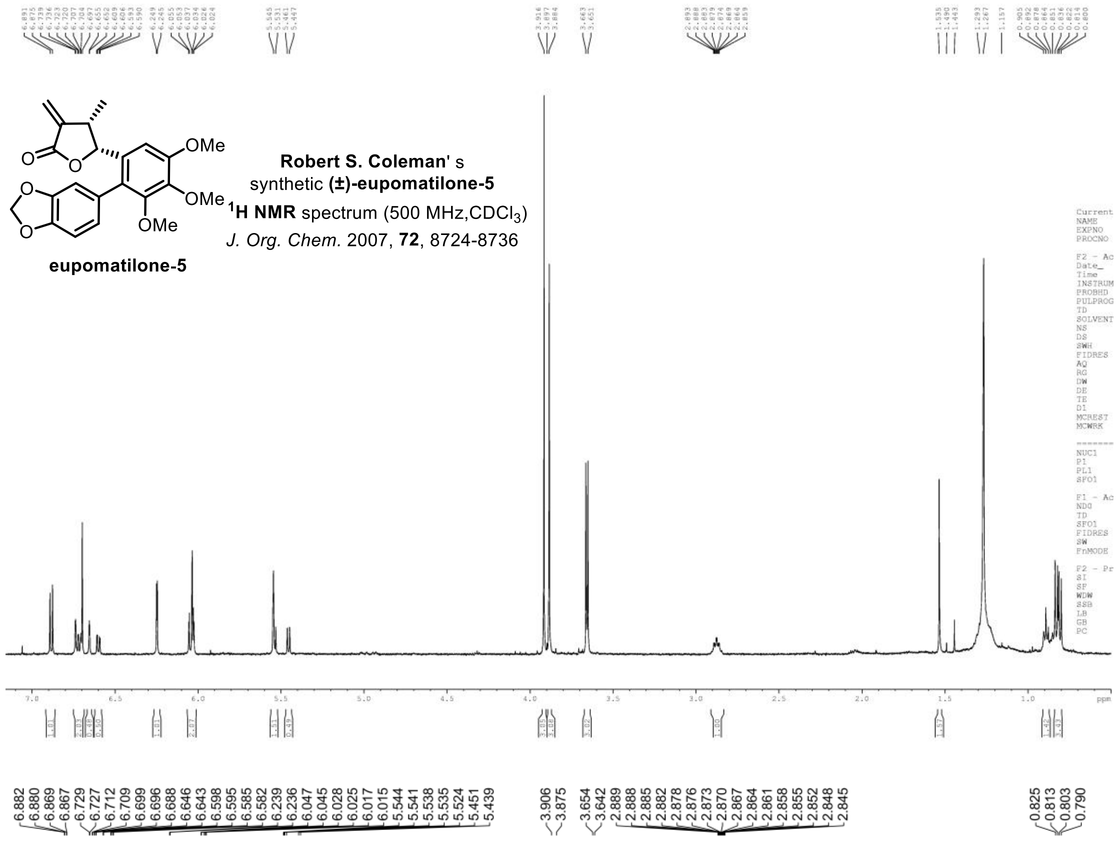
eupomatilone-5
Robert S. Coleman' s
synthetic (±)-eupomatilone-5
1H NMR spectrum (500 MHz,CDCl3)
J. Org. Chem. 2007, 72, 8724-8736
OMe
OMe
OMe

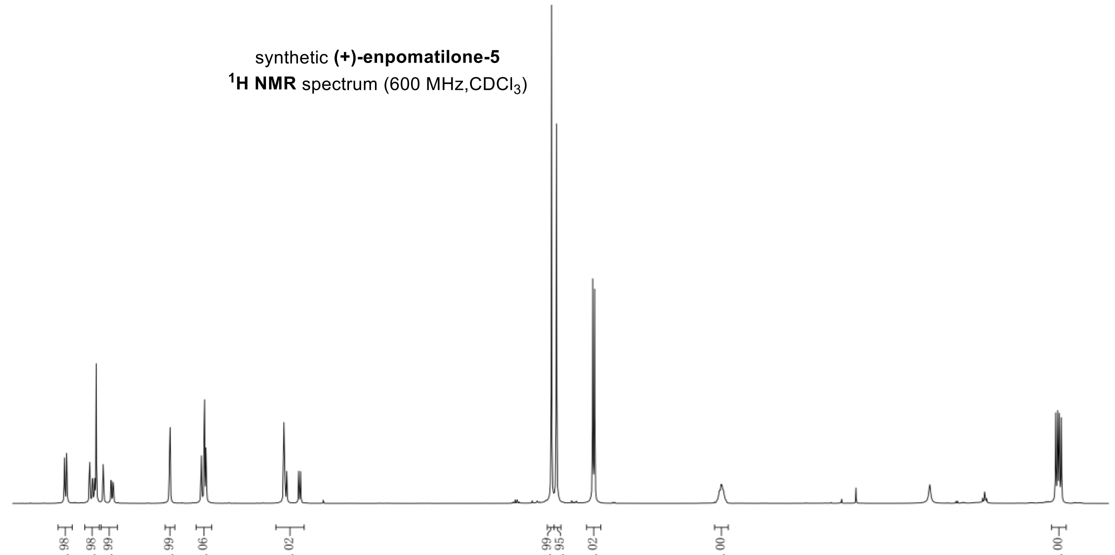
synthetic (+)-enpomatilone-5
1H NMR spectrum (600 MHz,CDCl3)

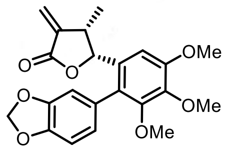

eupomatilone-5

Robert S. Coleman' s
synthetic **(±)-eupomatilone-5**
^{13}C NMR spectrum (125 MHz,$CDCl_3$)
J. Org. Chem. 2007, ***72***, 8724-8736

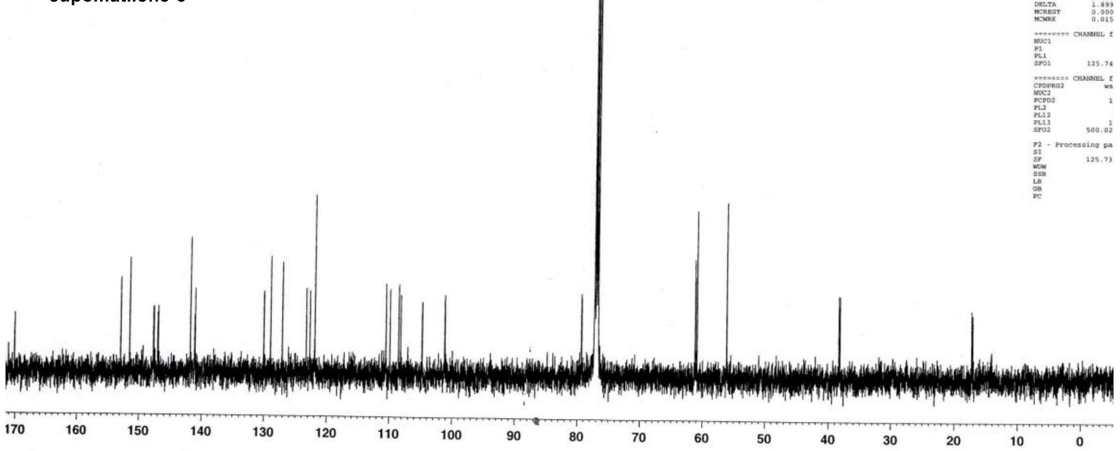

synthetic **(+)-enpomatilone-5**
^{13}C NMR spectrum (150MHz,$CDCl_3$)

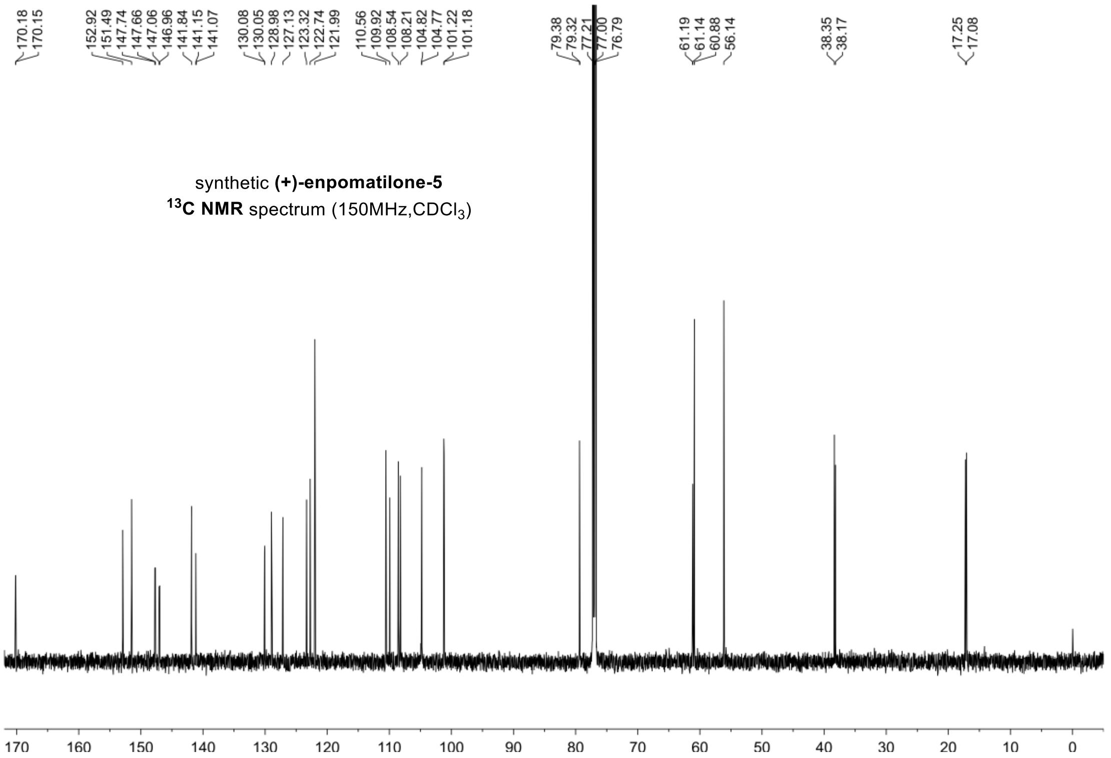

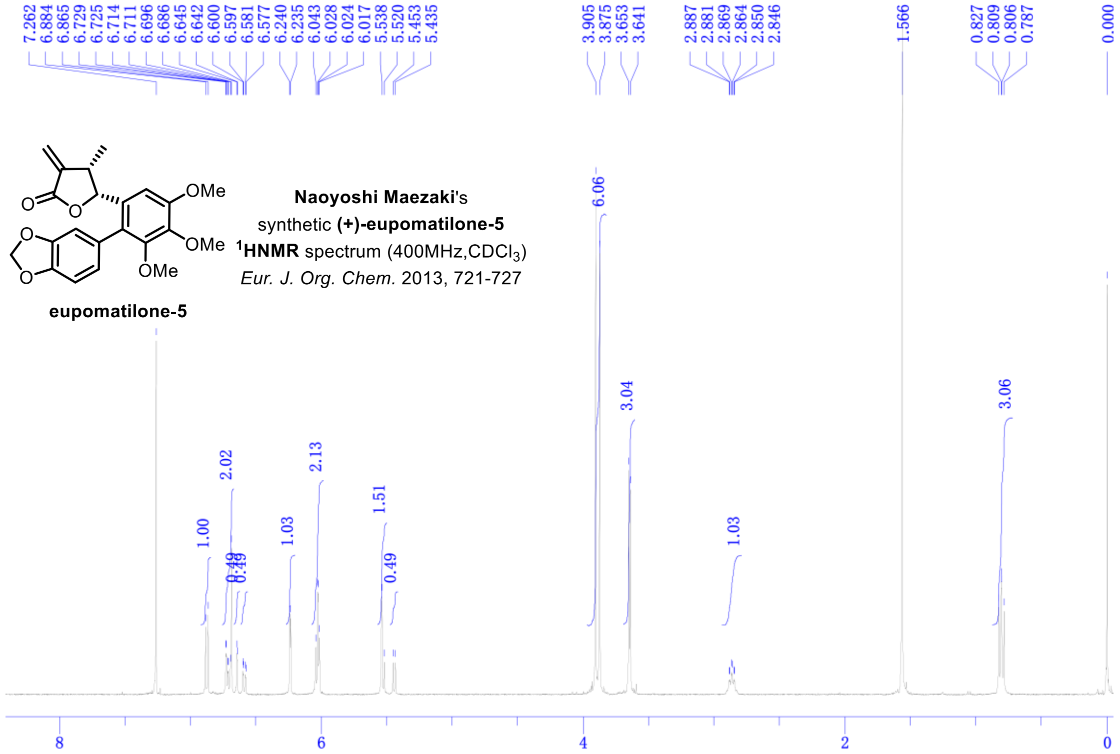
7.262 6.884 6.865 6.729 6.725 6.714 6.711 6.696 6.686 6.645 6.642 6.600 6.597 6.581 6.577 6.240 6.235 6.043 6.028 6.024 6.017 5.538 5.520 5.453 5.435
3.905 3.875 3.653 3.641
2.887 2.881 2.869 2.864 2.850 2.846
1.566
0.827 0.809 0.806 0.787
0.000
OMe
O
O
O
O
OMe
OMe
eupomatilone-5
Naoyoshi Maezaki's
synthetic (+)-eupomatilone-5
1HNMR spectrum (400MHz,CDCl3)
Eur. J. Org. Chem. 2013, 721-727
6.06
3.04
3.06
2.13
2.02
1.51
1.00
1.03
0.49
1.03
8
6
4
2
0

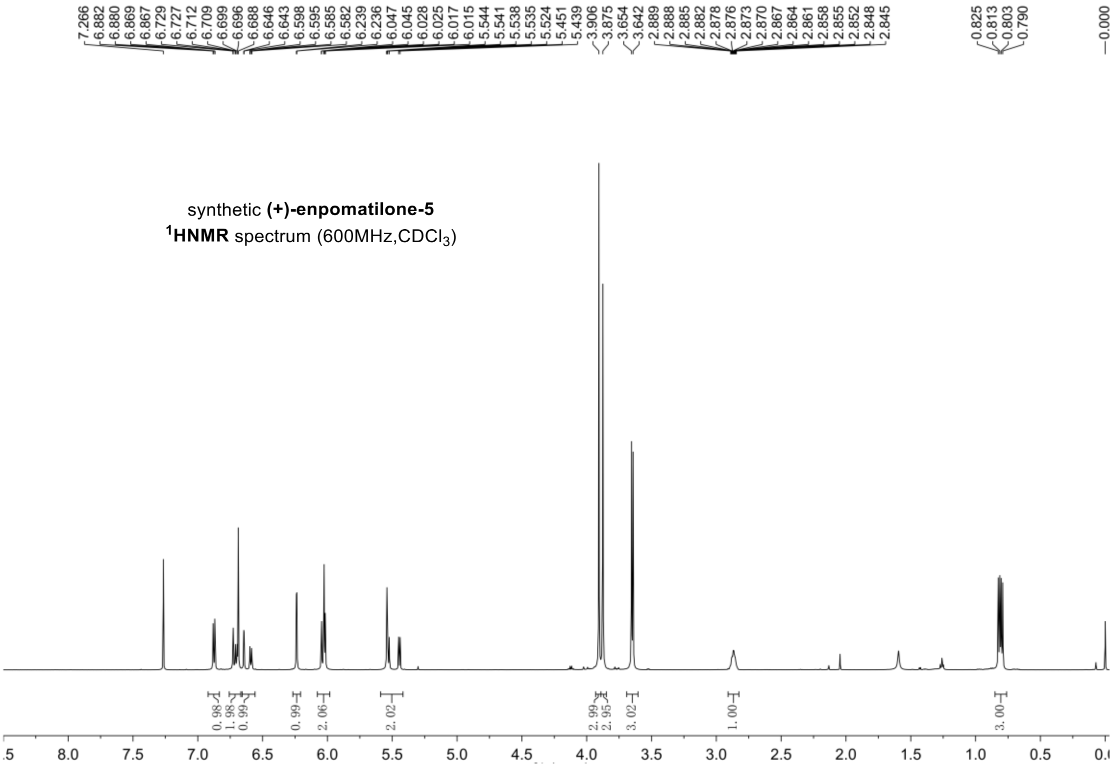
7.266 6.882 6.880 6.869 6.867 6.729 6.727 6.712 6.709 6.699 6.696 6.688 6.646 6.643 6.598 6.595 6.585 6.582 6.239 6.236 6.047 6.045 6.028 6.025 6.017 6.015 5.544 5.541 5.538 5.535 5.524 5.451 5.439
3.906 3.875 3.654 3.642
2.889 2.888 2.885 2.882 2.878 2.876 2.873 2.870 2.867 2.864 2.861 2.858 2.855 2.852 2.848 2.845
0.825 0.813 0.803 0.790
0.000
synthetic (+)-enpomatilone-5
1HNMR spectrum (600MHz,CDCl3)
0.98
1.98
0.99
0.99
2.06
2.02
2.99
2.95
3.02
1.00
3.00
8.0
7.5
7.0
6.5
6.0
5.5
5.0
4.5
4.0
3.5
3.0
2.5
2.0
1.5
1.0
0.5

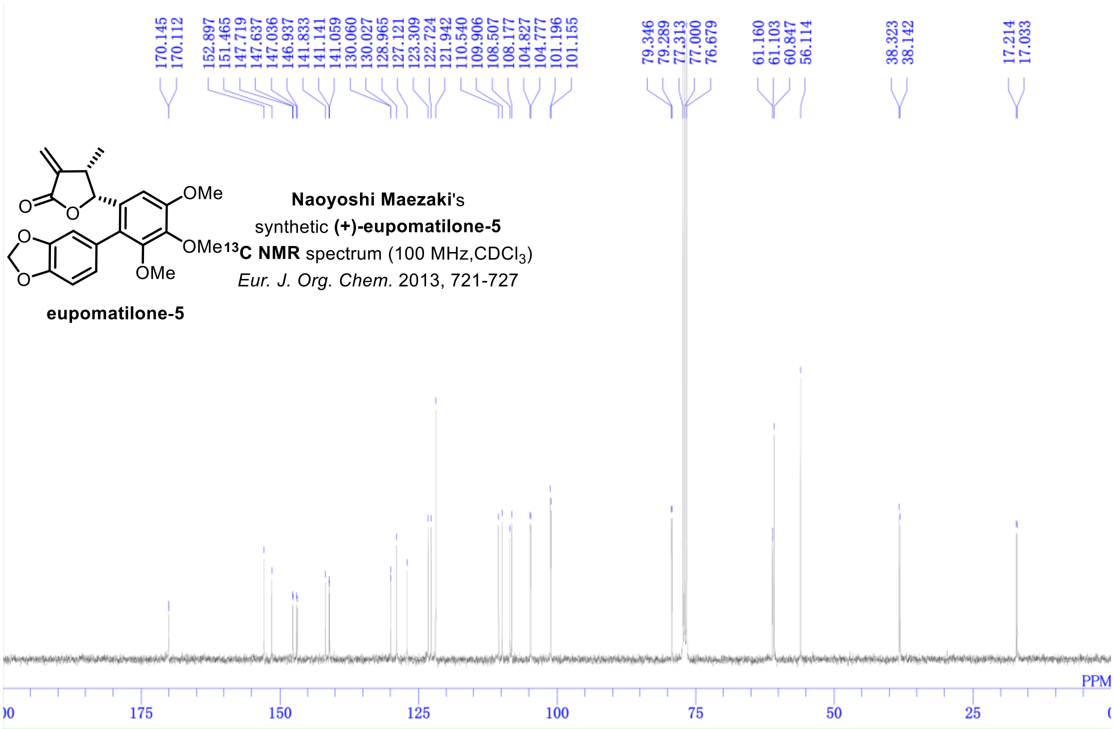
eupomatilone-5
Naoyoshi Maezaki's
synthetic (+)-eupomatilone-5
13C NMR spectrum (100 MHz,CDCl3)
Eur. J. Org. Chem. 2013, 721-727
PPM

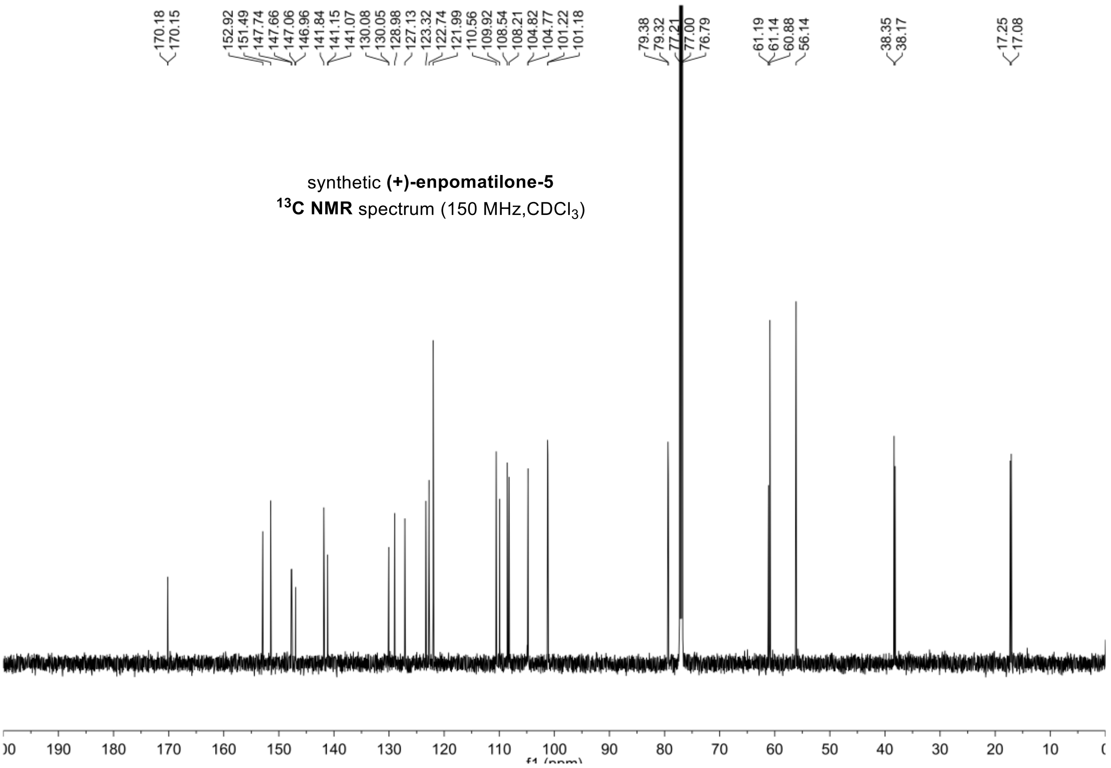
synthetic (+)-enpomatilone-5
13C NMR spectrum (150 MHz,CDCl3)
f1 (ppm)

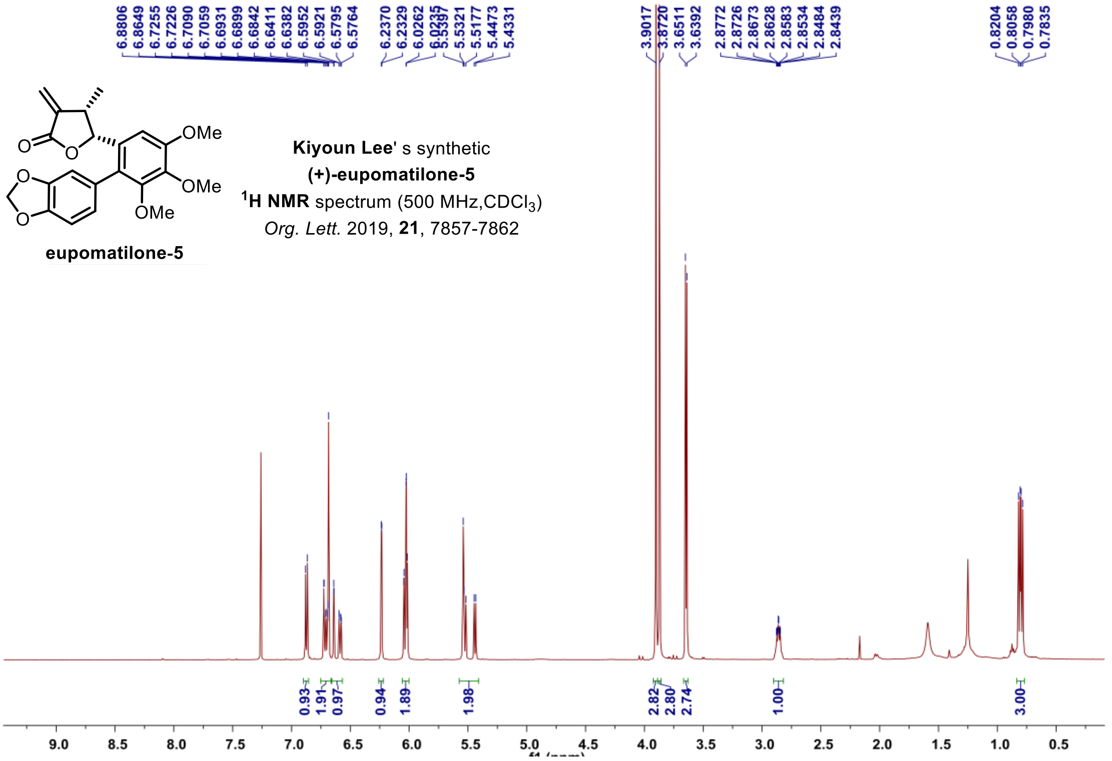
6.8806
6.8649
6.7255
6.7226
6.7090
6.7059
6.6931
6.6899
6.6842
6.6411
6.6382
6.5952
6.5921
6.5795
6.5764
6.2370
6.2329
6.0262
6.0235
5.5397
5.5321
5.5177
5.4473
5.4331
3.9017
3.8720
3.6511
3.6392
2.8772
2.8726
2.8673
2.8628
2.8583
2.8534
2.8484
2.8439
0.8204
0.8058
0.7980
0.7835
OMe
O
O
O
O
OMe
OMe
eupomatilone-5
Kiyoun Lee' s synthetic
(+)-eupomatilone-5
^{1}H NMR spectrum (500 MHz,$CDCl_3$)
Org. Lett. 2019, **21**, 7857-7862
0.93
1.91
0.97
0.94
1.89
1.98
2.82
2.80
2.74
1.00
3.00
9.0
8.5
8.0
7.5
7.0
6.5
6.0
5.5
5.0
4.5
4.0
3.5
3.0
2.5
2.0
1.5
1.0
0.5

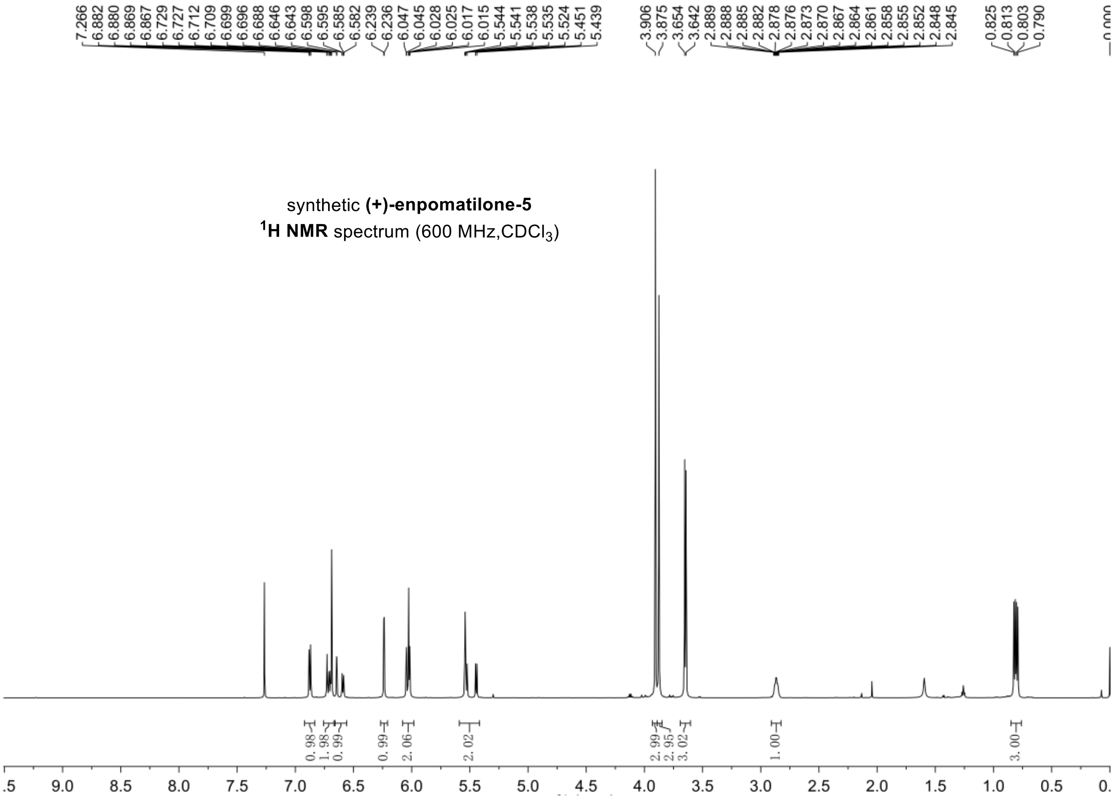
7.266
6.882
6.880
6.869
6.867
6.729
6.727
6.712
6.709
6.699
6.696
6.688
6.646
6.643
6.598
6.595
6.585
6.582
6.239
6.236
6.047
6.045
6.028
6.025
6.017
6.015
5.544
5.541
5.538
5.535
5.524
5.451
5.439
3.906
3.875
3.654
3.642
2.889
2.888
2.885
2.882
2.878
2.876
2.873
2.870
2.867
2.864
2.861
2.858
2.855
2.852
2.848
2.845
0.825
0.813
0.803
0.790
synthetic **(+)-enpomatilone-5**
^{1}H NMR spectrum (600 MHz,$CDCl_3$)
0.98
1.98
0.99
0.99
2.06
2.02
2.99
2.95
3.02
1.00
3.00
.5
9.0
8.5
8.0
7.5
7.0
6.5
6.0
5.5
5.0
4.5
4.0
3.5
3.0
2.5
2.0
1.5
1.0
0.5
0.

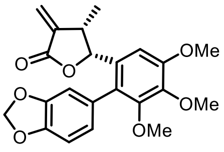

eupomatilone-5

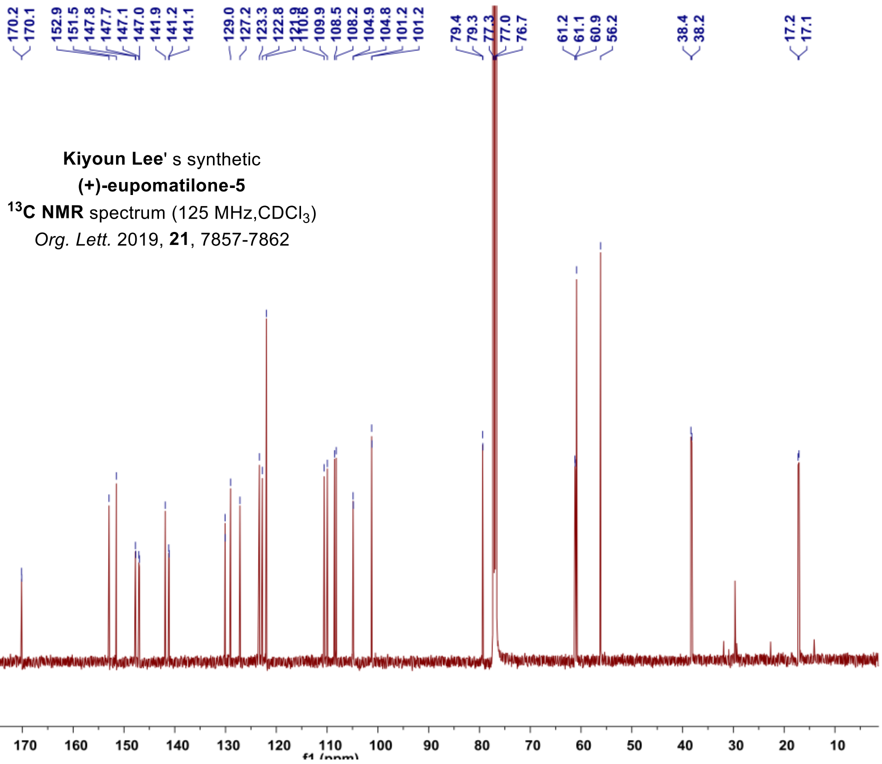

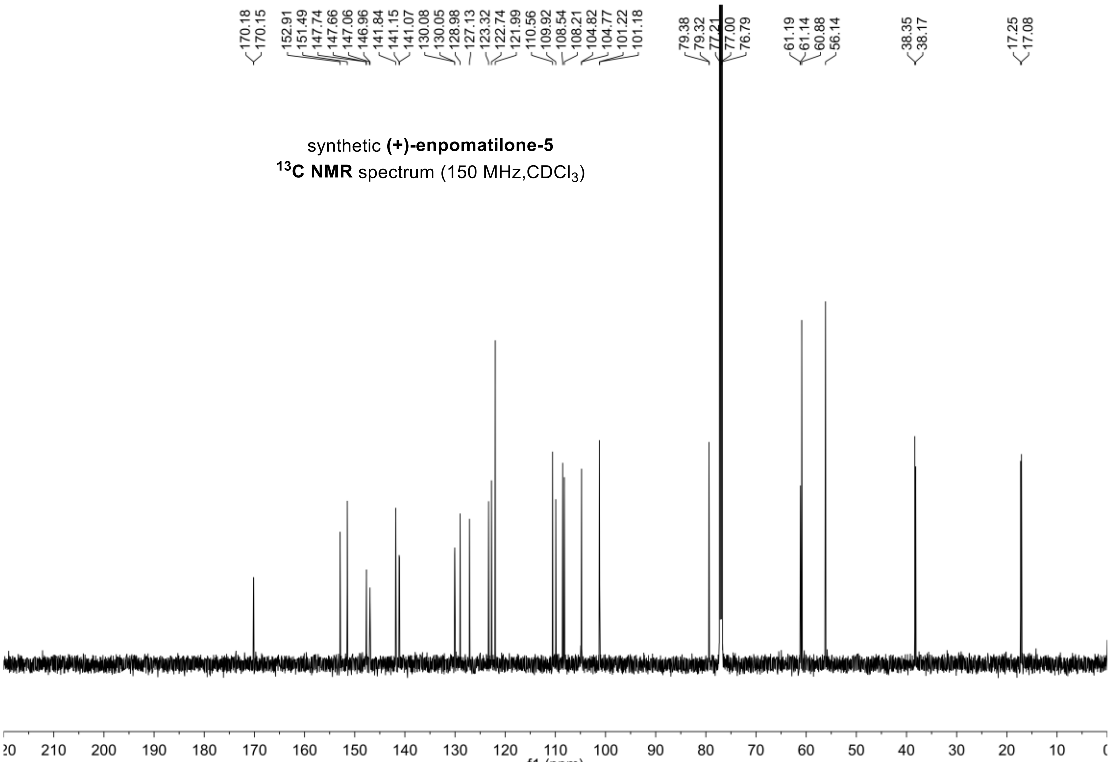

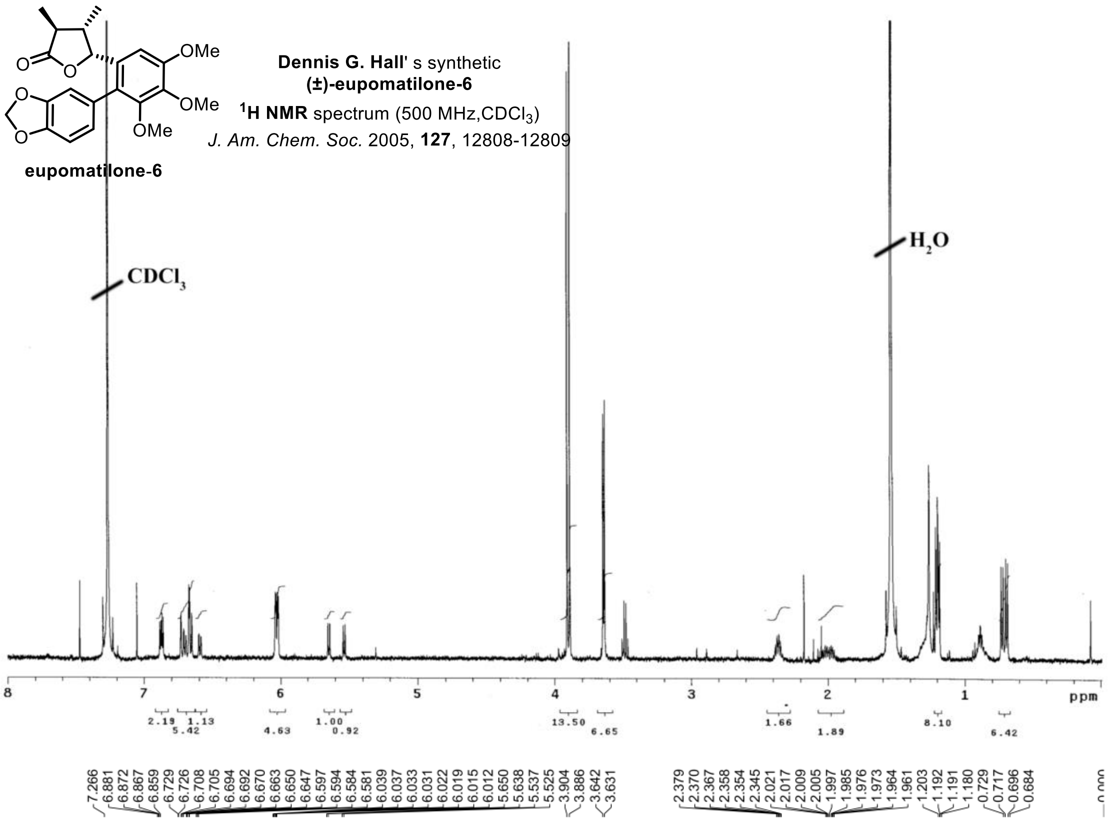

OMe
OMe
OMe
eupomatilone-6
Dennis G. Hall' s synthetic
(±)-eupomatilone-6
¹H NMR spectrum (500 MHz,CDCl3)
J. Am. Chem. Soc. 2005, 127, 12808-12809
CDCl3
H2O
ppm

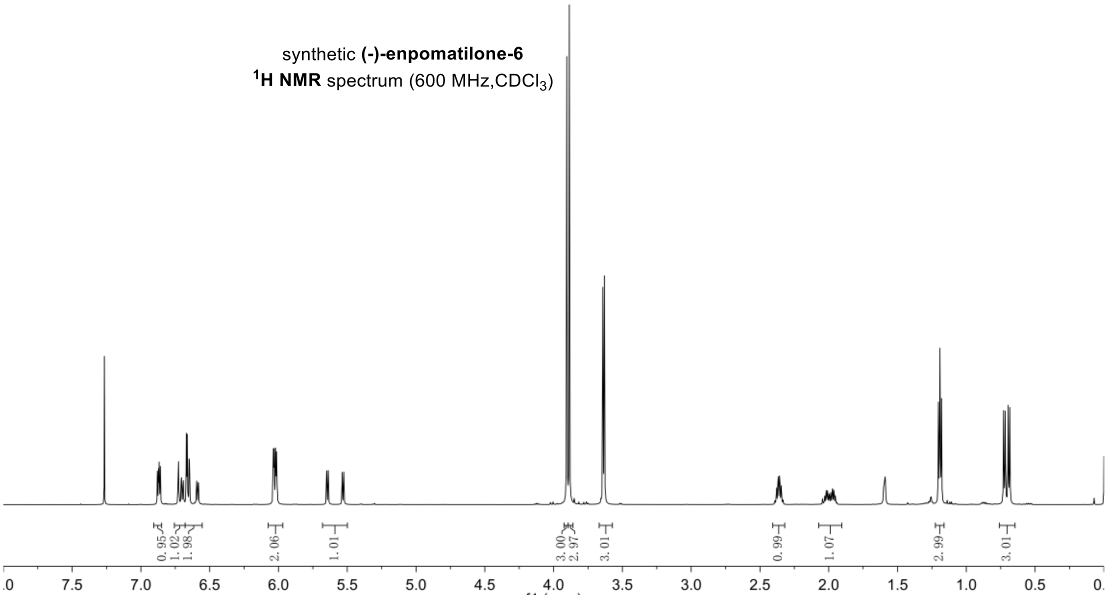

synthetic (-)-enpomatilone-6
¹H NMR spectrum (600 MHz,CDCl3)
f1 (ppm)

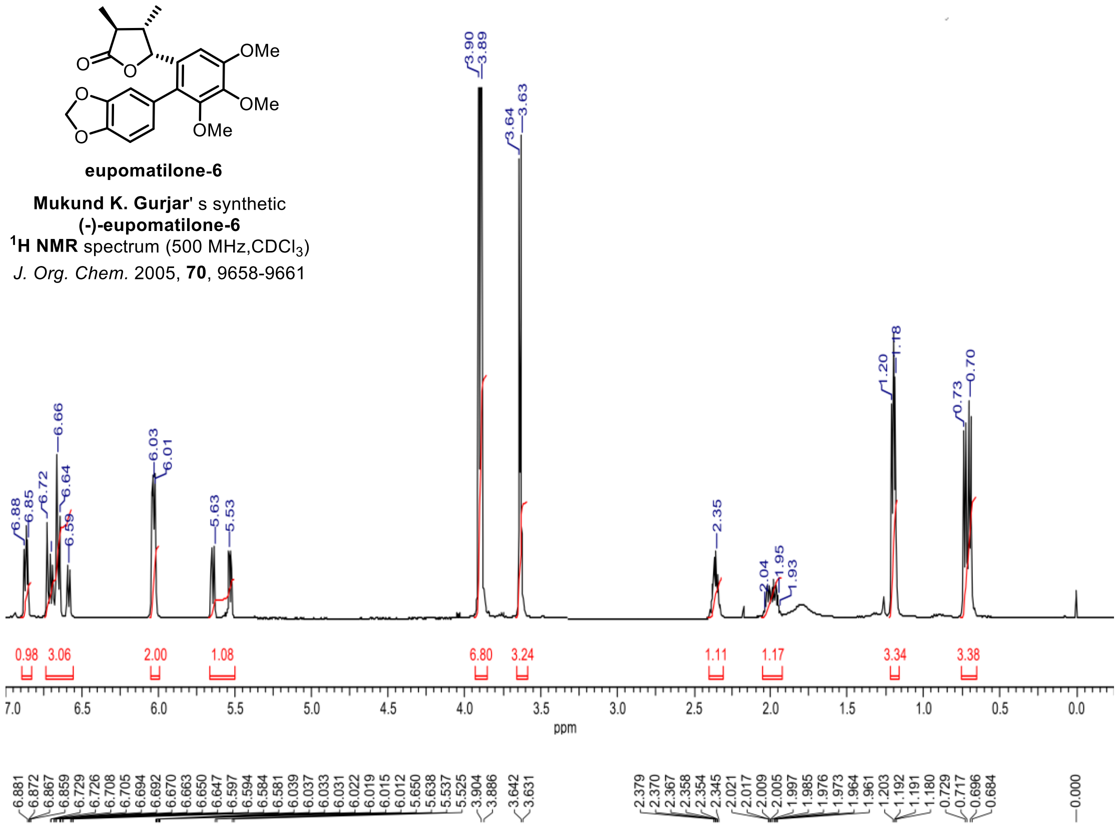
OMe
OMe
OMe
O
O
O
O
eupomatilone-6
Mukund K. Gurjar' s synthetic
(-)-eupomatilone-6
^{1}H NMR spectrum (500 MHz,CDCl$_3$)
J. Org. Chem. 2005, 70, 9658-9661
3.90 3.89
3.64 3.63
6.88 6.85 6.72 6.66 6.64 6.59
6.03 6.01
5.63 5.53
2.35
2.04 1.95 1.93
1.20 1.18
0.73 0.70
0.98 3.06 2.00 1.08 6.80 3.24 1.11 1.17 3.34 3.38
7.0 6.5 6.0 5.5 5.0 4.5 4.0 3.5 3.0 2.5 2.0 1.5 1.0 0.5 0.0
ppm
6.881 6.872 6.867 6.859 6.729 6.726 6.708 6.705 6.694 6.692 6.670 6.663 6.650 6.647 6.597 6.594 6.584 6.581 6.039 6.037 6.033 6.031 6.022 6.019 6.015 6.012 5.650 5.638 5.537 5.525 3.904 3.886 3.642 3.631
2.379 2.370 2.367 2.358 2.354 2.345 2.021 2.017 2.009 2.005 1.997 1.985 1.976 1.973 1.964 1.961 1.203 1.192 1.191 1.180 0.729 0.717 0.696 0.684
0.000

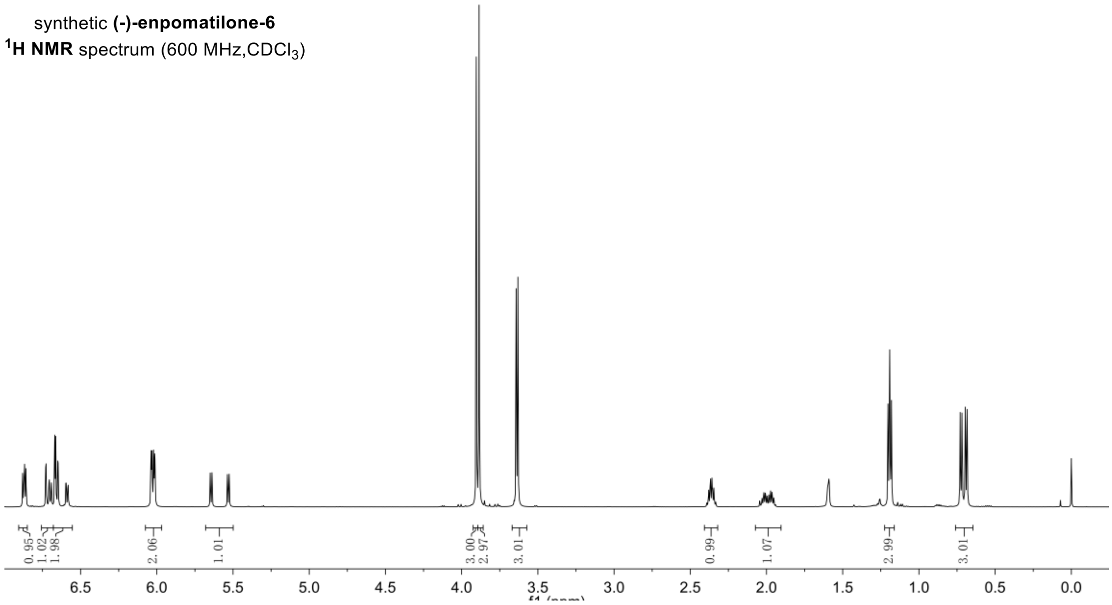
synthetic (-)-enpomatilone-6
^{1}H NMR spectrum (600 MHz,CDCl$_3$)
0.95 1.02 1.98 2.06 1.01 3.00 2.97 3.01 0.99 1.07 2.99 3.01
6.5 6.0 5.5 5.0 4.5 4.0 3.5 3.0 2.5 2.0 1.5 1.0 0.5 0.0

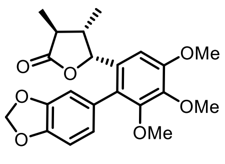

eupomatilone-6

Mukund K. Gurjar' s synthetic
(-)-eupomatilone-6
^{13}**C NMR** spectrum (125 MHz,CDCl$_3$)
J. Org. Chem. 2005, **70**, 9658-9661

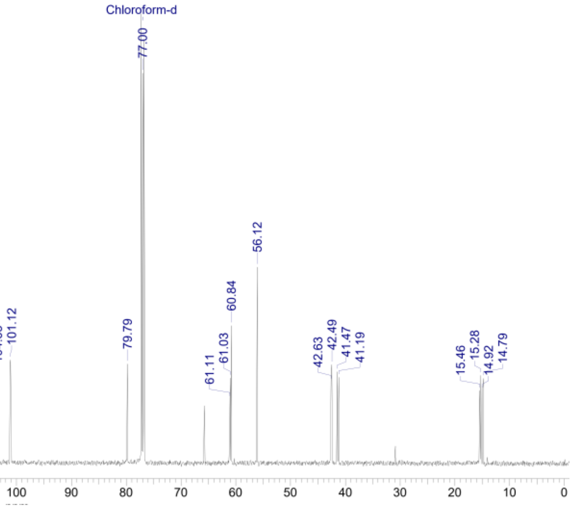

synthetic **(-)-enpomatilone-6**
^{13}**H NMR** spectrum (150 MHz,CDCl$_3$)

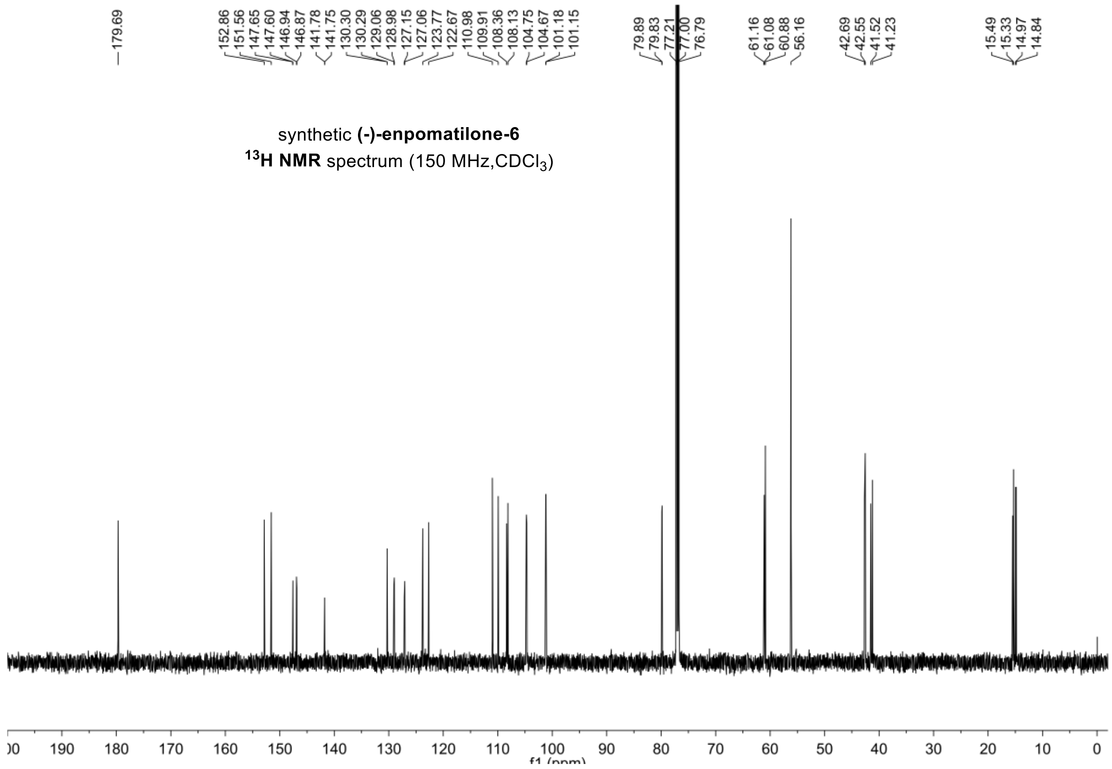

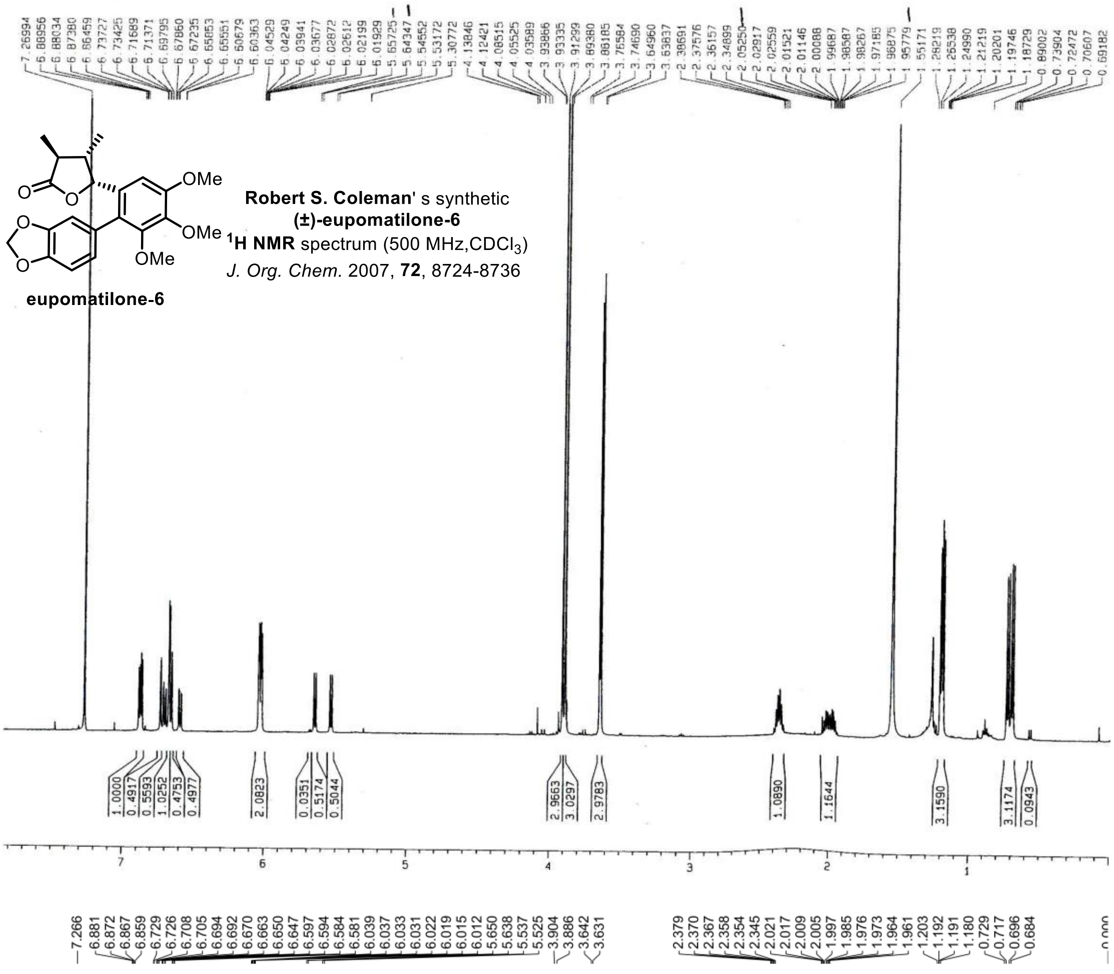
eupomatilone-6
OMe
OMe
OMe
Robert S. Coleman' s synthetic
(±)-eupomatilone-6
^{1}H NMR spectrum (500 MHz,CDCl$_3$)
J. Org. Chem. 2007, 72, 8724-8736

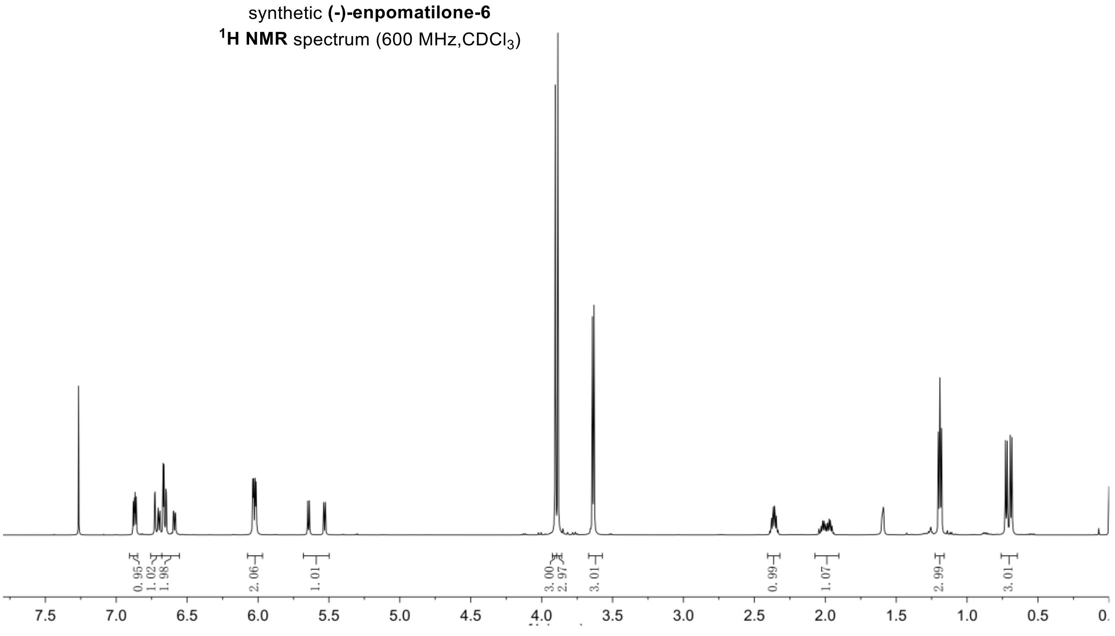
synthetic (-)-enpomatilone-6
^{1}H NMR spectrum (600 MHz,CDCl$_3$)

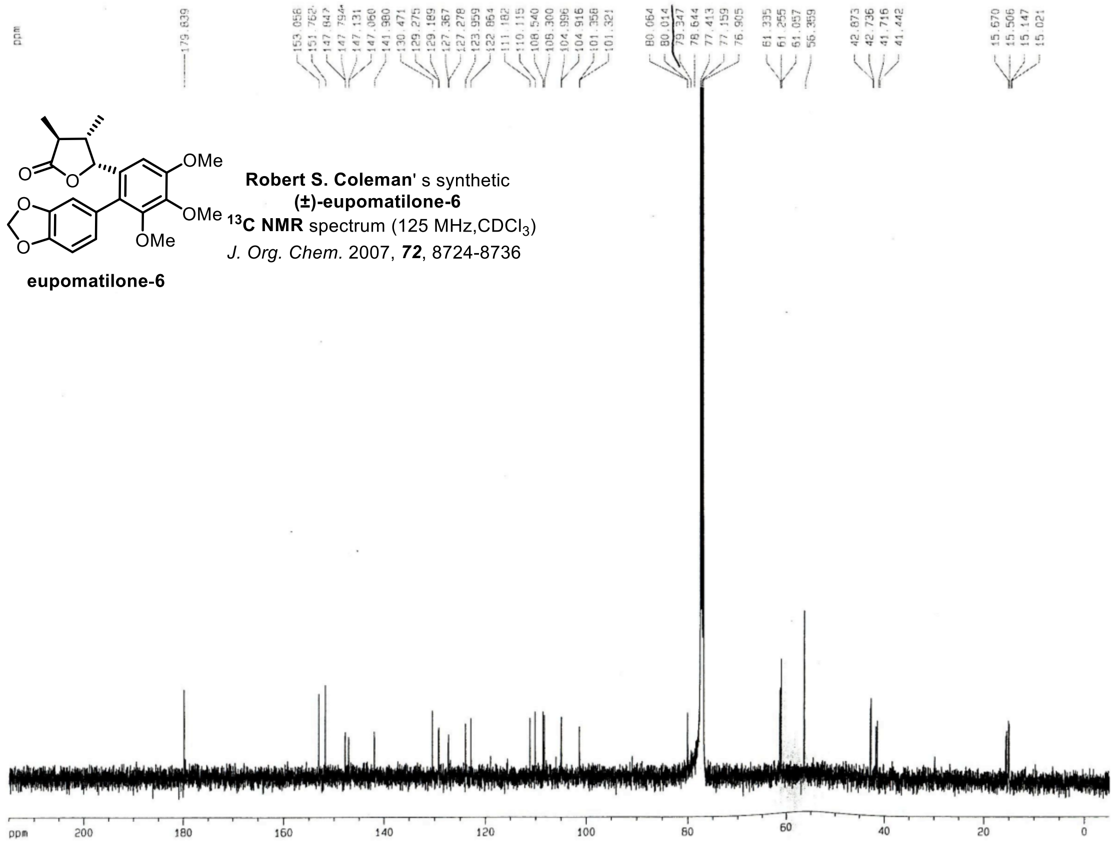

eupomatilone-6

Robert S. Coleman' s synthetic
(±)-eupomatilone-6
^{13}C NMR spectrum (125 MHz,$CDCl_3$)
J. Org. Chem. 2007, ***72***, 8724-8736

synthetic **(-)-enpomatilone-6**
^{13}H NMR spectrum (150 MHz,$CDCl_3$)

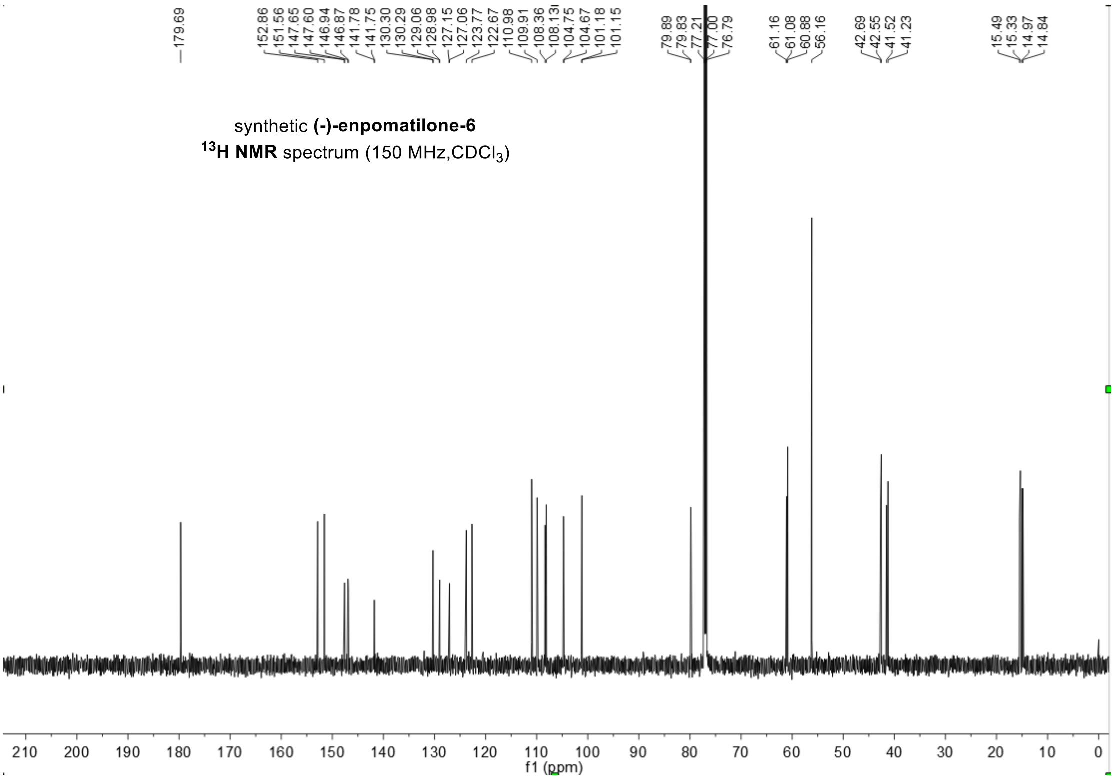

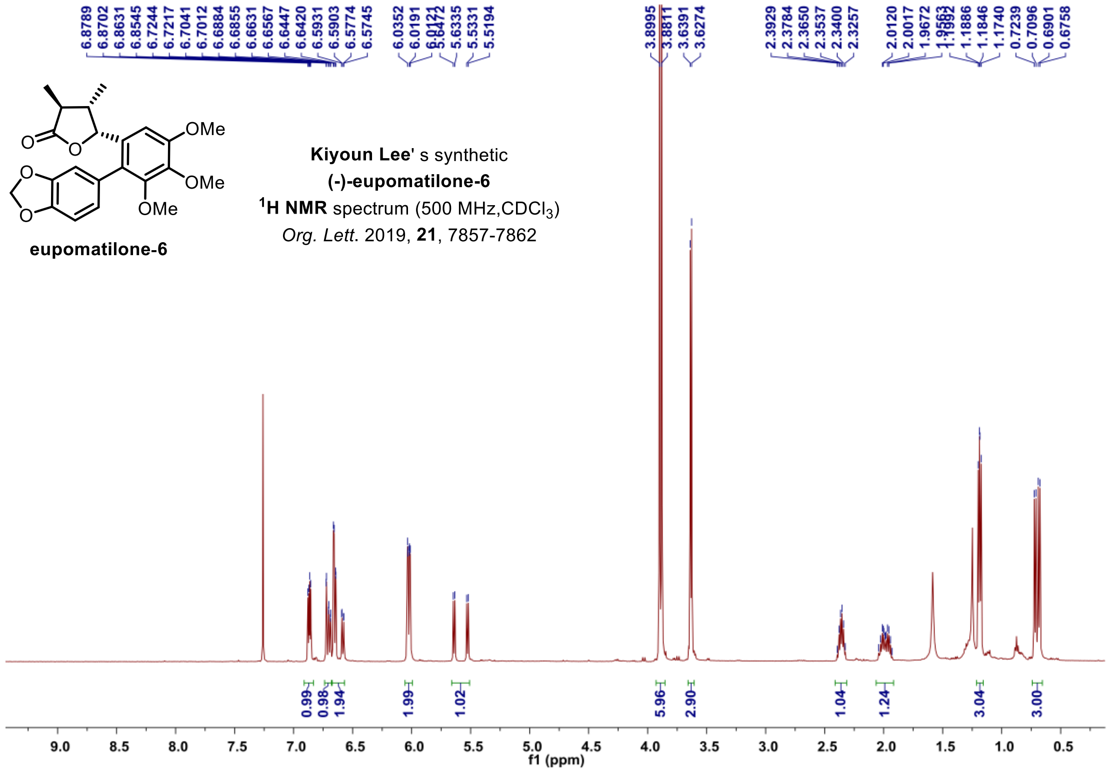

eupomatilone-6

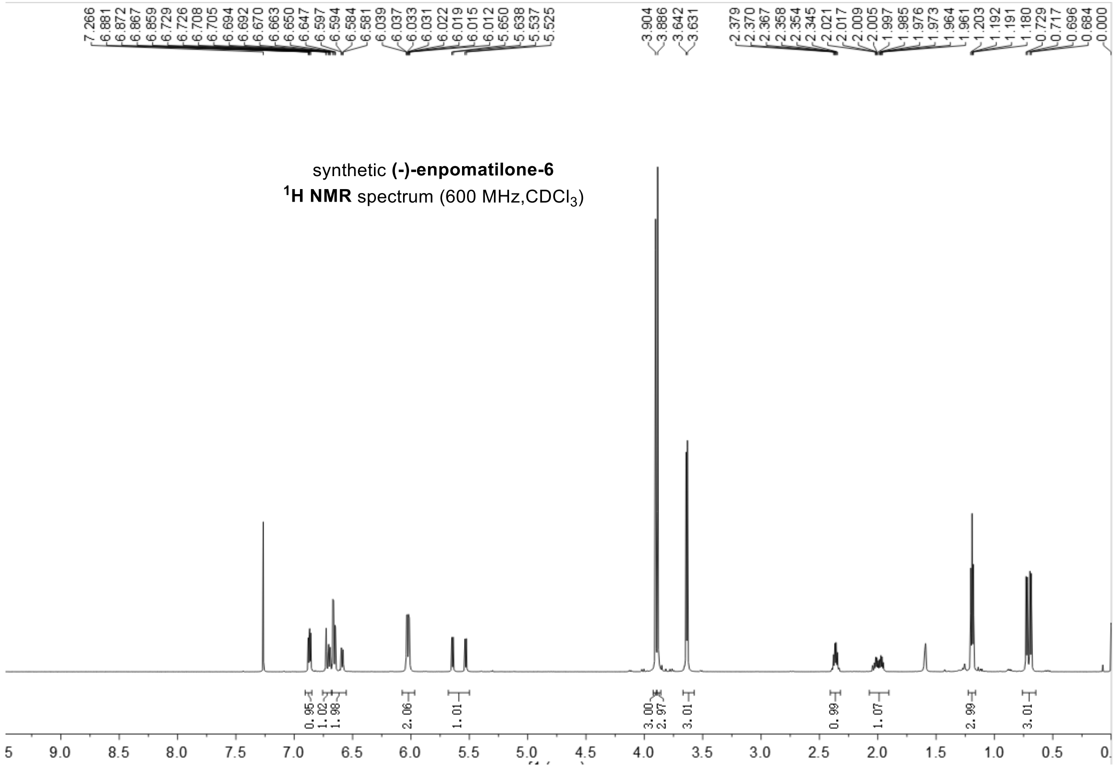

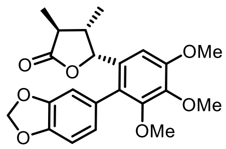

eupomatilone-6

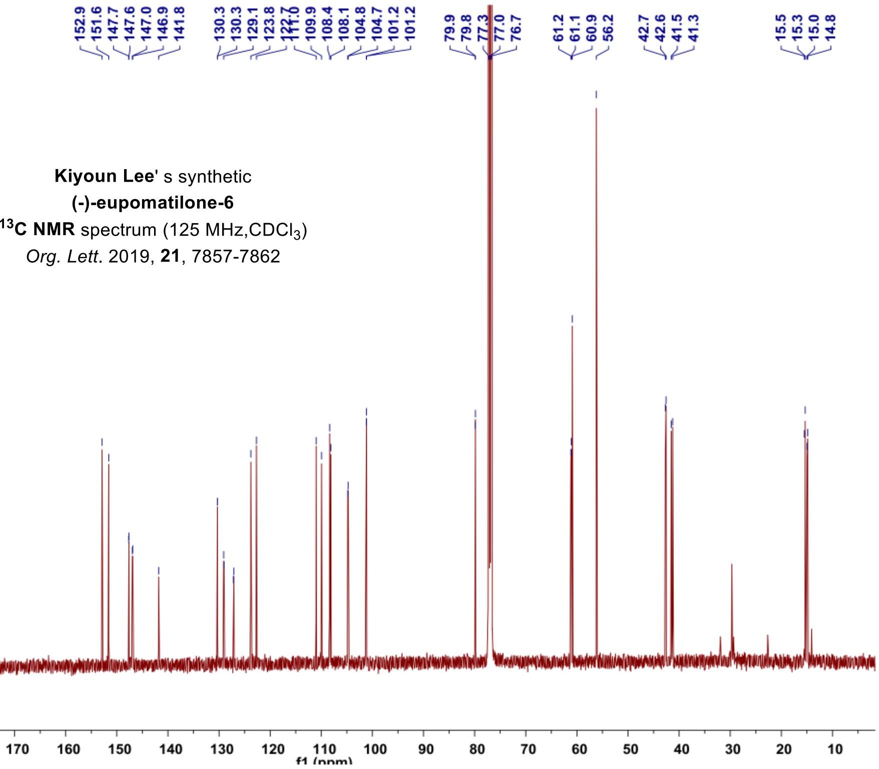

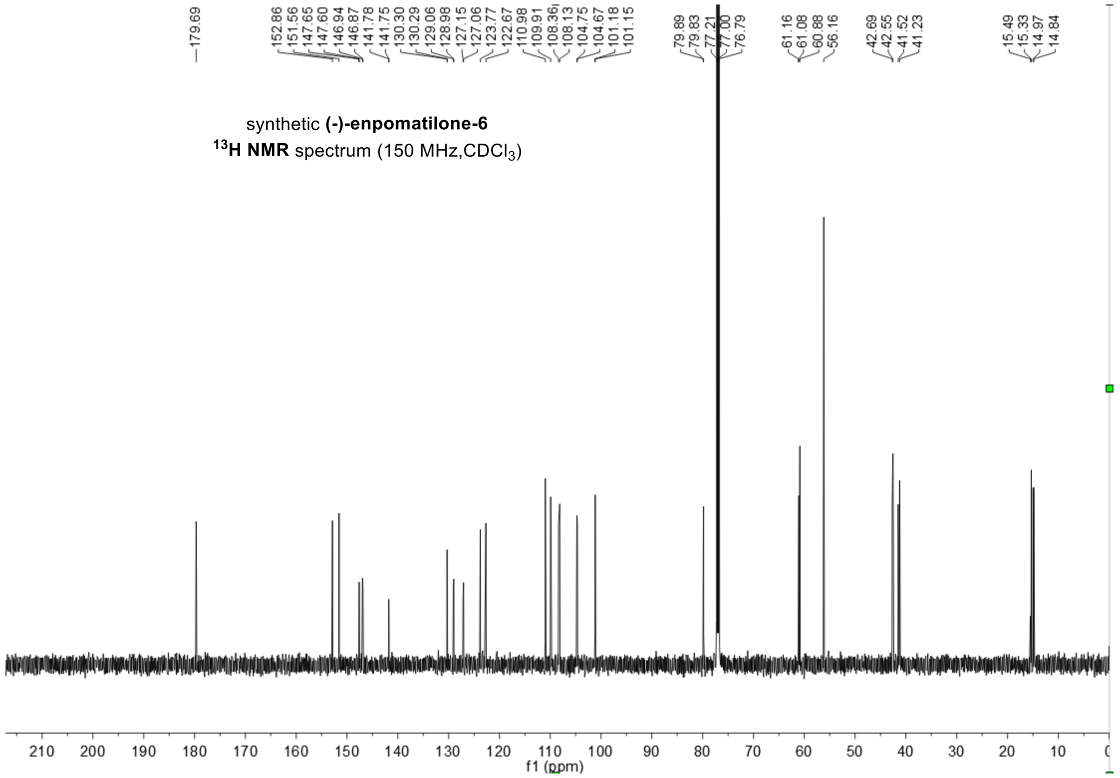

Supplement: SC-013-D2SC02079C-s001 [file SC-013-D2SC02079C-s001.pdf]
